# Gregarine single-cell transcriptomics reveals differential mitochondrial remodeling and adaptation in apicomplexans

**DOI:** 10.1186/s12915-021-01007-2

**Published:** 2021-04-16

**Authors:** Eric D. Salomaki, Kristina X. Terpis, Sonja Rueckert, Michael Kotyk, Zuzana Kotyková Varadínová, Ivan Čepička, Christopher E. Lane, Martin Kolisko

**Affiliations:** 1 Institute of Parasitology, Biology Centre, Czech Academy of Sciences, České Budějovice, Czech Republic; 2 Department of Biological Sciences, University of Rhode Island, Kingston, RI USA; 3 School of Applied Sciences, Edinburgh Napier University, Edinburgh, Scotland, UK; 4 Department of Zoology, Faculty of Science, Charles University in Prague, Prague, Czech Republic; 5 Department of Molecular Biology and Genetics, University of South Bohemia, České Budějovice, Czech Republic

**Keywords:** Mitochondria, Apicomplexa, Anaerobic metabolism, Phylogenomics, Evolution, Eugregarines, Mitosome, Parasitism

## Abstract

**Background:**

Apicomplexa is a diverse phylum comprising unicellular endobiotic animal parasites and contains some of the most well-studied microbial eukaryotes including the devastating human pathogens *Plasmodium falciparum* and *Cryptosporidium hominis*. In contrast, data on the invertebrate-infecting gregarines remains sparse and their evolutionary relationship to other apicomplexans remains obscure. Most apicomplexans retain a highly modified plastid, while their mitochondria remain metabolically conserved. *Cryptosporidium* spp. inhabit an anaerobic host-gut environment and represent the known exception, having completely lost their plastid while retaining an extremely reduced mitochondrion that has lost its genome. Recent advances in single-cell sequencing have enabled the first broad genome-scale explorations of gregarines, providing evidence of differential plastid retention throughout the group. However, little is known about the retention and metabolic capacity of gregarine mitochondria.

**Results:**

Here, we sequenced transcriptomes from five species of gregarines isolated from cockroaches. We combined these data with those from other apicomplexans, performed detailed phylogenomic analyses, and characterized their mitochondrial metabolism. Our results support the placement of *Cryptosporidium* as the earliest diverging lineage of apicomplexans, which impacts our interpretation of evolutionary events within the phylum. By mapping in silico predictions of core mitochondrial pathways onto our phylogeny, we identified convergently reduced mitochondria. These data show that the electron transport chain has been independently lost three times across the phylum, twice within gregarines.

**Conclusions:**

Apicomplexan lineages show variable functional restructuring of mitochondrial metabolism that appears to have been driven by adaptations to parasitism and anaerobiosis. Our findings indicate that apicomplexans are rife with convergent adaptations, with shared features including morphology, energy metabolism, and intracellularity.

## Background

The phylum Apicomplexa is a lineage comprised primarily of unicellular endobiotic parasites of animals and includes pathogens of agriculturally important livestock (e.g., *Eimeria* spp. causing coccidiosis) and humans (e.g., *Plasmodium falciparum* and *Toxoplasma gondii*, the causative agents of malaria and toxoplasmosis, respectively) [1–3]. Due to public health implications, medically important apicomplexans were an early focus for genome sequencing efforts and remain as some of the most well-studied microbial eukaryotes [1, 2, 4]. Apicomplexan organelles were identified as possible targets for drug therapy development and, accordingly, have been the subject of detailed molecular characterization [5–11]. Although most apicomplexans (e.g., *Toxoplasma*, *Babesia*, and *Plasmodium* spp.) retain mitochondria akin to aerobic eukaryotes, they have undergone some restructuring. For example, pyruvate dehydrogenase (PDH) has been replaced by branched chain ketoacid dehydrogenase (BCKDH) and the canonical electron transport chain complex I has been replaced by a single protein that encodes an alternative NADH dehydrogenase (NDH2) [12–14]. *Cryptosporidium* spp. represent the exception, as their adaptation to parasitism and hypoxia within the digestive tract has produced a range of functionally reduced mitochondria, including the complete loss of their mitochondrial genomes [13, 15]. *Cryptosporidium muris* and *C. andersoni* use a common anaerobic pathway to convert pyruvate into acetyl-CoA which is then shuttled to the tricarboxylic acid (TCA) cycle [13]. At the extreme, the mitochondrion-related organelles (MROs) of *C. hominis* and *C. parvum* represent some of the most highly reduced mitochondria known and are no longer capable of ATP generation [13, 16]. The observation of such modifications established interest in the apicomplexan mitochondrion as a model for understanding fundamental questions regarding eukaryotic evolution and organellar retention.

Although apicomplexans comprise some of the most well-studied microbial eukaryotes, the gregarines represent an important exception. Genomic-scale data are scarce from gregarines, despite the importance of their phylogenetic placement for interpretations of evolutionary events within the phylum [17, 18]. Since most of our knowledge on gregarines is derived from microscopic studies and 18S rRNA sequencing [17–21], little is known about the evolution or metabolic potential of gregarine organelles. Recent technical advances in single-cell methods have reduced the barriers to data generation and enabled broad genome-scale explorations of gregarines. In 2019, Janouškovec et al. [22] and Mathur et al. [23] independently published data demonstrating that a “gregarine-type” morphology (conspicuously large single-celled symbionts which infect the intestine, coelom, or reproductive vesicles of invertebrate hosts and possess an attachment apparatus that contains the apical complex at least in the infective stages) has evolved at least three times, highlighting the importance of these understudied lineages to the accurate interpretation of apicomplexan evolution. The only targeted investigation into the mitochondrial metabolism of gregarines was largely speculative, as it was based on the incomplete and highly fragmented *Ascogregarina taiwanensis* genome [24]. As close relatives to the MRO-containing *Cryptosporidium*, gregarines provide an ideal model for exploring mitochondrial functional reduction.

Interestingly, phylogenomic analyses performed by Mathur et al. [23] and Janouškovec et al. [22] produced conflicting topologies for the relationships between gregarines and other apicomplexans. Mathur et al. added data from newly sequenced transcriptomes to a dataset generated by Burki et al. [25], producing a tree that placed gregarines as a sister lineage to *Cryptosporidium* and the other apicomplexans [23]. Using a different set of taxa and an independently derived phylogenomic dataset, the analysis conducted by Janouškovec et al. [22] supports a clade containing *Cryptosporidium* and gregarines as sister to other apicomplexans. These conflicting evolutionary histories have significant impacts on our interpretations of the evolutionary events that include patterns of organellar reduction and transitions to intracellularity. Therefore, these relationships must be resolved before meaningful conclusions on organelle and life history evolution can be made.

In this study, we explored the impact of adaptations to the host environment and parasitism on apicomplexan mitochondrial metabolism, with a particular focus on gregarines. We expanded the available genome-scale data for understudied apicomplexans by generating transcriptomes for five gregarines isolated from terrestrial hosts. These data were combined with all publicly available gregarine data to conduct the most taxonomically rich phylogenomic analyses of Apicomplexa to date. We robustly placed *Cryptosporidium* as the earliest diverging apicomplexan lineage. Within this evolutionary framework, we characterized the previously unknown mitochondrial metabolism of gregarines and provide evidence for multiple independent mitochondrial functional reductions and electron transport chain losses within the Apicomplexa.

## Results and discussion

Transcriptomes were generated for gregarines isolated from five species of cockroaches (Additional file 1: Table S1). Small subunit rRNA (SSU) sequences from the newly sequenced transcriptomes were searched against the NCBI nr database to confirm species identifications of isolated gregarines from different cockroach species. SSU sequences from *Blabericola migrator* (isolated from *Gromphadorhina portentosa*) and *Protomagalhaensia wolfi* (isolated from *Nauphoeta cinerea*) were each > 99.9% similar to published data for these species. The SSU sequence similarity of gregarines from * Polyphaga aegyptiaca* (from here on *Gregarina* sp. Poly) and those from *Pseudoderopeltis* sp. (from here on *Gregarina* sp. Pseudo) were each ~ 96% similar to *Gregarina blattarum* (FJ459741.1). The SSU from gregarines isolated from *Gyna caffrorum* (from here on *Protomagalhaensia* sp. Gyna) was ~ 92% similar to the published SSU from *Protomagalhaensia wolfi* (FJ459758.1).

To explore the evolutionary relationships within the apicomplexans, we reconstructed phylogenies using two different phylogenomic datasets, based on those from previously published studies with conflicting results [22, 23]. Mathur et al. [23] used 39 taxa and 198 genes (out of a possible 248 genes [26]), compared to 74 taxa and 246 genes in our version of the dataset (from here forward called dataset A). Janouškovec et al. [22] utilized 50 taxa and 296 genes (based on original dataset of 339 genes [27]), while we have retained 299 genes and 74 taxa (from here forward called dataset B). Overall, our dataset A (246 genes) and dataset B (299 genes) had 27.72% and 32.02% missing data, respectively.

**Fig. 1:**
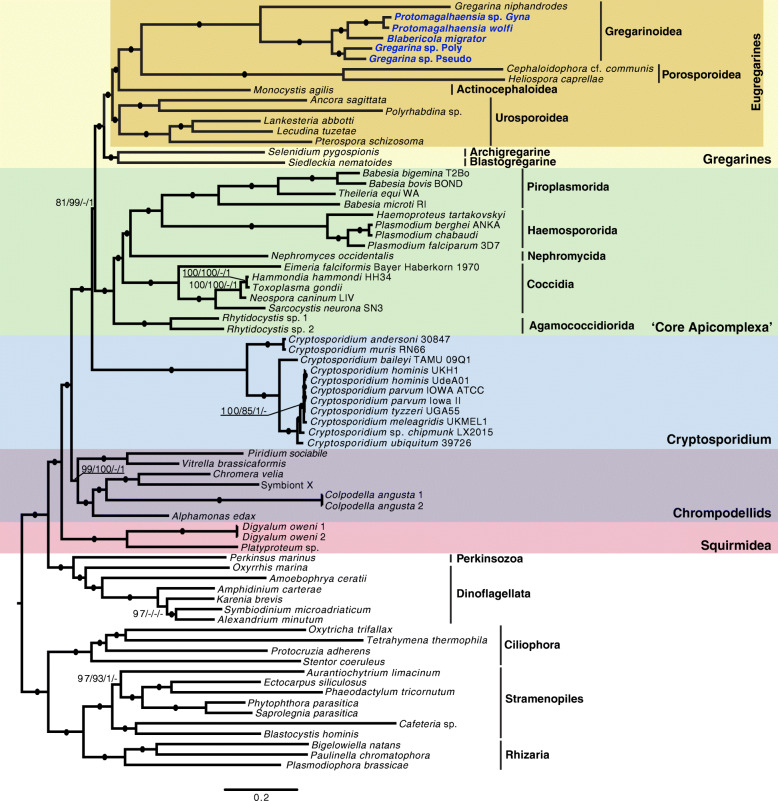
Phylogeny of Apicomplexa. Maximum likelihood phylogeny of apicomplexans as recovered from two independently developed phylogenomic datasets, which both recovered identical topologies. Branch lengths shown are from dataset A. Dataset A was comprised of 246 genes and 63,201 sites, while dataset B was comprised of 299 genes and 89,675 sites. Non-parametric PMSF bootstrap support (BS) values (*n* = 1000) and Phylobayes posterior probabilities (PP) are shown on the branches as follows: dataset A BS/dataset B BS/dataset A PP/dataset B PP. Branches with black circles received maximum support in all analyses. Support values below 80 BS or 0.9 PP are not shown. Gregarines newly sequenced in this study are bolded and colored blue

**Fig. 2:**
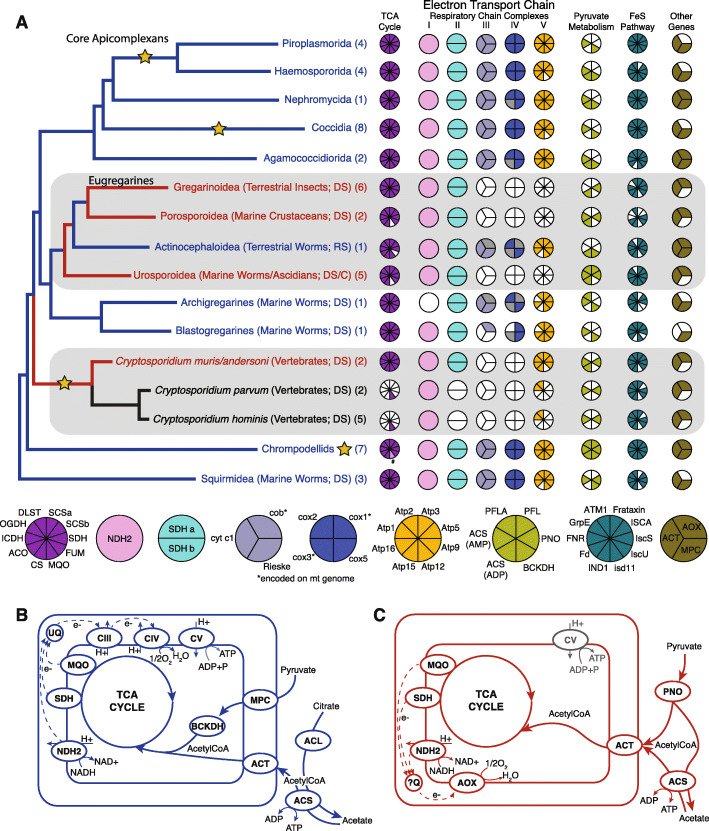
Evolution of apicomplexan mitochondrial metabolism. **a** Cartoon phylogeny of apicomplexans and their close relatives with Coulson plots representing the presence and absence of the tricarboxylic acid cycle (TCA), electron transport chain complexes (ETC), and other genes important for apicomplexan mitochondrial metabolism. The “#” symbol and lighter coloration for MQO in chrompodellids are to signify that they retain malate dehydrogenase rather than malate:quinone oxidoreductase for their TCA cycle. Genes encoded on the mitochondrial genome are indicated with an asterisk. If mitochondrion genome data are absent for a lineage, the corresponding piece of the Coulson plot is colored gray where the gene is presumed to exist due to retention of other genes in the associated complex. Branch names include taxonomy, host information, localization of parasites within the host (DS, digestive system; RS, reproductive system; C, coelom), and the number of genomes and transcriptomes included in each lineage. Gold stars on nodes indicate transitions to an intracellular lifestyle with the star next to the name for Chrompodellids representing an independent event within the clade giving rise to *Piridium sociable*. Branches on the phylogeny are colored according to in silico predictions for mitochondrial metabolism that are similarly colored and shown as **b** representing regular aerobic apicomplexan mitochondria which is depicted in blue and **c** representing an anaerobic mitochondrial metabolism. The functional role of the ETC complex V (ATP synthase) in *Cryptosporidium muris* and *C. andersoni* is shown in gray

Both datasets recovered identical topologies for the relationships between apicomplexans under maximum likelihood (ML) and Bayesian inference (BI) analyses: *Cryptosporidium* is the earliest diverging lineage, with gregarines branching sister to a clade containing Agamococcidiorida, Coccidia, Nephromycida, Haemospororida, and Piroplasmorida (from here on referred to as “core” apicomplexans) (Fig. 1; see Additional file 2: Fig S1, Additional file 3: Fig S2, Additional file 4: Fig S3, Additional file 5: Fig S4, Additional file 6: Fig. S5, Additional file 7: Fig. S6 for individual ML and BI trees). The relationship of gregarines with core apicomplexans was strongly supported by dataset B (99 ML bootstrap (BS), 1.0 BI posterior probability (PP)); however, this clade received lower support from dataset A (81 BS, 0.51 PP). Interestingly, this topology was not recovered by Janouškovec et al. [22] or by Mathur et al. [23] and therefore was the subject of further investigation (see below). The archigregarine *Selenidium pygospionis* and the blastogregarine *Siedleckia nematoides* form a clade branching sister to eugregarines. However, with sequence data available for only one member of each group, this branching pattern may change with additional data. Distinct and fully supported clades of eugregarines branched in correlation to their host organisms, habitat, and the localization of infection within the host (Fig. 2). Although Actinocephaloidea is represented by only a single transcriptomic dataset here, the relationship of Actinocephaloidea and Gregarinoidea has been repeatedly supported with the use of more taxa in phylogenetic analyses of 18S [20], complete rRNA operon [28], and phylogenomic datasets [23]. Janouškovec et al. [22] similarly recovered these groups as sister lineages using three individual actinocephaloids merged into one hybrid dataset in their phylogenomic analysis, but also recovered the similar relationship using all three separately, but with each containing a high amount of missing data. Rather than incorporate numerous fragmented actinocephaloid datasets (e.g., the *Ascogregarina taiwanensis* genome data contains 2.5% complete BUSCO genes) to evaluate this repeatedly established relationship, we selected the highly complete *Monocystis agilis* dataset (86.5% complete or partial BUSCO genes; Additional file 1: Table S1) to represent Actinocephaloidea. In our analyses, we are still able to confidently reconfirm the relationship of Actinocephaloidea and Gregarinoidea as sister groups. The long branches of many of these lineages, combined with a lack of data from numerous families, indicate the need for an increased sampling effort for gregarines from diverse hosts to test whether these co-evolutionary patterns will hold up. However, based on currently available data, it appears that gregarines have transitioned at least twice between marine and terrestrial hosts. Co-evolution of gregarines and their hosts is exemplified in the monophyly of cockroach-infesting gregarines branching as sister to *G. niphandrodes* (from the beetle *Tenebrio molitor*). Furthermore, gregarines originating from Blaberidae cockroaches (*B. migrator*, *P. wolfi*, *Protomagalhaensia* sp. Gyna) form a clade, excluding those from other cockroach families Blattidae and Corydiidae (*Gregarina* sp. Poly, *Gregarina* sp. Pseudo). However, more data are needed to draw meaningful conclusions.

**Fig. 3:**
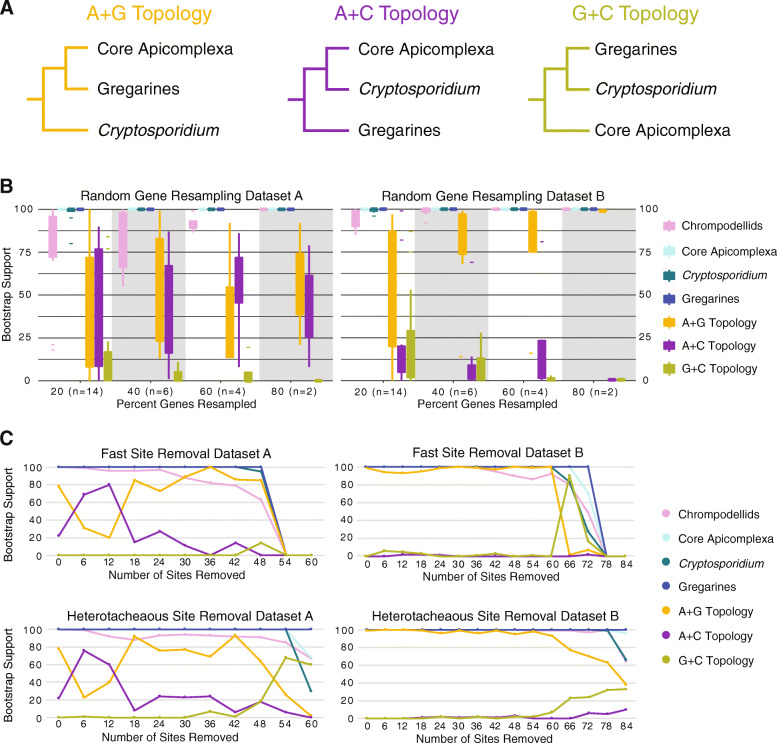
Effects of removing fast evolving and heterotacheous sites, and random gene subsampling on apicomplexan relationships. **a** Colored schematic of the three possible relationships between *Cryptosporidium* spp., gregarines, and core apicomplexans. A+G (yellow) shows monophyly of core apicomplexans and gregarines, A+C (purple) shows monophyly of core apicomplexans and *Cryptosporidium* spp., and G+C (green) shows monophyly of gregarines and *Cryptosporidium* spp. **b** Box-and-whisker plots showing support for randomly sampled subsets of genes from each total dataset (dataset A on the left and dataset B on the right). Non-parametric bootstrap support (*n* = 100) values are on the *y*-axis and subsample percentage is shown on the *x*-axis. The number of individual datasets required to sample every gene with 95% probability of sampling every gene for each percentage of genes being sampled is shown in parentheses. Each bipartition of interest is shown in a different color with A+G, A+C, and G+C corresponding to the colors in **a**. **c** Graphs plotting support for bipartitions of interest after the stepwise removal of the 6000 fastest evolving sites (top) and 6000 most heterotachous sites (bottom) until all sites are removed from each dataset (dataset A on the left and dataset B on the right). Non-parametric bootstrap support (*n* = 100) values are on the *y*-axis and the number of sites removed, measured in thousands, is shown on the *x*-axis

There are three possible relationships between gregarines, *Cryptosporidium*, and the core apicomplexan clade: (1) monophyly of gregarines and core Apicomplexa (A+G topology; Fig. 3a), (2) monophyly of *Cryptosporidium* and core Apicomplexa (A+C topology; Fig. 3a), and (3) monophyly of gregarines and *Cryptosporidium* (G+C topology; Fig. 3a). Interestingly, Mathur et al. [23] recovered A+C topology and Janouškovec et al. [22] recovered G+C topology, while we recover the A+G topology.

To further explore the support and robustness of the three topologies, we performed several additional analyses on each dataset: (1) gene concordance factors (gCF) based on single genes and on the partitioning scheme, (2) approximately unbiased (AU) topology test, (3) random gene resampling, and (4) identification and removal of genes that could strongly influence a particular topology. The gCF scores for all three topologies were below 10% based on individual genes and below 20% based on the partitioning scheme but were highest for the A+G topology with a score of 15.5% (dataset A) and 18.6% (dataset B) based on the partitioning scheme (Additional file 8: Table S2). However, Minh et al. [29] suggest that nodes with low gCF scores should be the focus of further scrutiny. The AU topology test only rejected the G+C topology based on dataset B (P_*G + C*_ = 0.0313) (Additional file 9: Table S3), further highlighting the difficulty in placing the root of the Apicomplexa. Random gene resampling unsurprisingly showed the highest variability for the smallest datasets (20%) (Fig. 3b). Similar to the gCF and AU tests, support for the G+C topology was lowest in all randomly subsampled gene sets for both datasets A and B. Support for the A+G and A+C topologies were similar across all levels of gene subsampling within dataset A, and support for the A+G topology was dominant in dataset B (Fig. 3b). Genes that strongly influence a particular topology were identified in each dataset by averaging the absolute difference in gene-wise log-likelihood for each pair of the three possible topologies, according to the protocol described by Shen et al. [30]. After removing outlier genes from dataset A (11 genes), the topology switched to the A+C with 86 BS support (100 PMSF bootstrap replicates) while dataset B (13 genes) topology remained the same with 96 BS support.

To further investigate the differences between datasets A and B, we have identified overlapping and unique gene sets. There are 153 genes shared between the two datasets, with 93 genes being unique to dataset A and 146 genes being unique to dataset B (Additional file 10: Table S4). Maximum likelihood trees were reconstructed based on subsets of the shared and unique genes. The trees generated based on the shared genes for both datasets, as well as from genes unique to dataset B, all resulted in the A+G topology (Additional file 11: Table S5). In contrast, the genes unique to dataset A produced a tree with the A+C topology (Additional file 11: Table S5) and are likely the cause of lower support for the A+G topology observed in dataset A.

Since many lineages within the apicomplexans formed relatively long branches, we also tested the possible impact of systematic error (e.g., long branch attraction (LBA) or non-optimal model usage) driving support for any of the topologies. We performed serial fast evolving site removal (FSR) [31, 32] and heterotacheous site removal (HSR) [33], with the 6000 fastest or most heterotacheous sites being removed at each step (Fig. 3c). For dataset A, there is a short rise of support for the A+C topology, which then disappears in favor of the A+G topology in both FSR and HSR analyses (Fig. 3c). There is little support for the G+C topology throughout the FSR and HSR analyses of dataset A (Fig. 3c). For dataset B, support for the A+G topology remains > 99 for both FSR and HSR analyses, while the other two topologies are barely supported (Fig. 3c). Given the switch to the A+C topology support in the concatenation of dataset A unique genes, we conducted fast site removal, removing 3000 sites at each step from this dataset as well. In this analysis, after removing 9000 sites, the topology switched from A+C to A+G (Additional file 12: Fig. S7). To further investigate the role of systematic errors contributing to the observed topologies, we ran phylogenomic analyses using the less complex LG model to strengthen a possible artifactual result (compared to the partitioned LG+C60+F+G used in our previous analyses), followed by FSR analysis under this model. Both datasets A and B received higher support for the A+C topology using the LG model. The FSR analyses on both datasets partially rescued the A+G topology that we recovered under the LG+C60+F+G model (Additional file 13: Fig. S8). These findings strongly suggest that the A+C topology recovered by Mathur et al. [23] was likely influenced by a systematic error. It is very likely that the combination of partitioning with mixture models [34], and significantly increased taxon sampling helped to suppress systematic errors in this study. We were unable to assess the driving force behind the topology recovered by Janouškovec et al. [22]; however, we hypothesize that the differences may lie in our increased taxon sampling and use of data partitioning with complex models [34]. It is also possible that the extended taxon sampling improved our resolution of orthologs [35].

The above results show that dataset A cannot strongly distinguish between the A+G and A+C topologies as the topology switches from A+G with bootstrap support 81 to A+C with bootstrap support 86 after the removal of 11 outlier genes (out of 246). Interestingly, these 11 genes do not strictly prefer A+C topology (Additional file 14: Fig. S9). Both FSR and HSR analyses suggest that the support for the A+C topology in dataset A is at least partially driven by systematic errors. The support for the A+C topology declines as fast evolving sites are removed from dataset A (Fig. 3), the LG model analysis of dataset A (Additional file 13:Fig. S8), and in genes unique to dataset A (Additional file 12: Fig. S7). We conclude that the A+C topology may be an artifact resulting from systematic error; however, we cannot entirely rule out this relationship. The G+C topology was rejected by an AU test for dataset A and received very low support in the absolute majority of our analyses. Based on these results, we consider that A+G topology is most likely correct, given the currently available data. However, it remains possible that improved taxon sampling, especially of novel close relatives of *Cryptosporidium*, or the development of evolutionary models better suited for resolving deep evolutionary radiations could alter support for this topology.

### Implications for apicomplexan evolution

Our topology indicates that evolutionary adaptations to different host environments are ubiquitous throughout the Apicomplexa. Within the gregarines, we observe either two independent transitions from infecting a marine host to a terrestrial host, or a switch from marine hosts to terrestrial, and then back to marine (Fig. 2). Additionally, while the majority of gregarines are described from the digestive system of diverse invertebrate hosts, some Actinocephaloidea reside within the reproductive system of their hosts, requiring adaptation to different host tissues (Fig. 2). Despite the contention between the A+C and A+G relationships observed in our analyses, either topology demonstrates that apicomplexans are prone to transitions between intracellular and extracellular lifestyles. Based on our phylogeny (Fig. 1), we suspect that the apicomplexan ancestor was an extracellular endobiont with intracellularity arising independently on at least three occasions: once in *Cryptosporidium*, again in the ancestor of Coccidia, and a third time in the shared ancestor of Haemospororida and Piroplasmorida. It is important to note that *Cryptosporidium* spp. form a parasitophorous vacuole in the plasma membrane of the host, remaining extracytoplasmic [36], unlike more derived apicomplexan parasites like *Toxoplasma* and *Plasmodium* which invade the cytoplasm of the host cell [37, 38], supporting independent origins of intracellularity. However, alternative scenarios remain possible: the apicomplexan ancestor may have been an intracellular parasite with gregarines and Agamococcidioroida independently becoming extracellular parasites, or a transition to an extracellular lifestyle occurred after *Cryptosporidium* diverged and then returned again to intracellularity in the ancestor of Eimeriorina. Interestingly, *Piridium sociabile*, a parasite that infects the subepithelial connective tissue of whelks [39], was recently placed within the chrompodellids [23]. This placement, which is confirmed here, further demonstrates the propensity of apicomplexans and their close relatives to become intracellular parasites.

#### Apicomplexan mitochondrial metabolism

To explore how parasitism and adaption to an endobiotic lifestyle impacted gregarine mitochondrial functionality, we searched for metabolically important mitochondrial proteins in a database comprised of 53 transcriptomes and genomes from apicomplexans, chrompodellids, and Squirmidea (Additional file 1: Table S1). We identified 146 proteins involved in mitochondrial metabolism present in these lineages (Additional file 15: Table S6, Additional file 16: Fig. S10). When data were available, proteins encoded on the mitochondrial genomes were included in our mitochondrial metabolic reconstruction (Fig. 2). These data revealed striking diversity of metabolic capacity among gregarine lineages and support three independent losses of the electron transport chain within Apicomplexa.

### Pyruvate metabolism

In typical aerobic mitochondrion, pyruvate dehydrogenase (PDH) converts pyruvate into acetyl-CoA that is then oxidized into CO_2_ in the TCA cycle, while also reducing NAD+ to NADH. NADH is then utilized by the electron transport chain to generate ATP by oxidative phosphorylation. In more reduced anaerobic MROs, the conversion of pyruvate into acetyl-CoA is commonly facilitated by pyruvate:ferredoxin oxidoreductase (PFO), pyruvate:NADP oxidoreductase (PNO), and/or pyruvate formate lyase (PFL) [40]. Acetyl-CoA can then be metabolized to acetate, while generating ATP from ADP by substrate-level phosphorylation. *Cryptosporidium hominis* and *C. parvum* both lack most genes involved in the TCA cycle and the ETC and can only produce ATP via substrate-level phosphorylation, for example during glycolysis or metabolism of acetyl-CoA to acetate. Although PDH can be found in many apicomplexan lineages, previous studies demonstrated that apicomplexan PDH is localized to the apicoplast rather than the mitochondrion [41]. Instead, most apicomplexans import pyruvate into the mitochondrion via the mitochondrial pyruvate carrier (MPC) where it is converted to acetyl-CoA by the BCKDH complex which is then shuttled into the TCA cycle (Fig. 2b). In *C. muris*, the enzyme PNO converts pyruvate to acetyl-CoA in the cytosol which is presumably then shuttled into the mitochondrion via the acetyl-CoA transporter [42, 43]. Once in the mitochondrion, acetyl-CoA is utilized by the TCA cycle (Fig. 2c), producing NAD(P)H that is ultimately oxidized by a reduced respiratory chain consisting of NDH2 and alternative oxidase (AOX) [43]. Archigregarines, blastogregarines, and the eugregarine superfamily Actinocephaloidea all maintain the pathway of BCKDH converting pyruvate to acetyl-CoA akin to the aerobically functioning mitochondria in core apicomplexans (Fig. 2b). In contrast, the other three eugregarine lineages lost BCKDH and instead use a PNO-based pyruvate metabolism pathway, akin to MROs described from other anaerobes (Fig. 2c) [44]. PNO is conspicuously missing in the *G. niphandrodes* data. Since no other enzyme that metabolizes pyruvate has been identified in *G. niphandrodes*, the absence of PNO may reflect an incomplete nature of this genome.

Substrate-level phosphorylation generating ATP via ADP-forming acetyl-CoA synthetase (ACS) has been characterized in anaerobic lineages across the eukaryotic tree of life with functionally reduced mitochondria [44, 45]. Investigations into apicomplexan acetyl-CoA synthetase have focused primarily on the AMP-forming ACS found in all well-characterized lineages. This AMP-forming ACS has been described to function in fatty acid transport and the production of acetyl-CoA for protein acetylation and fatty acid elongation [14, 43, 46, 47]. Acetyl-CoA synthetase has also been suggested to function in substrate-level phosphorylation in *C. muris*, but not in any other apicomplexan [13, 44]. Here, in addition to the AMP-forming ACS, we identified an ADP-forming ACS found in all eugregarines as well as with patchy distribution throughout Apicomplexa (Additional file 17: Fig. S11). This ADP-forming ACS has been previously shown to have originated in diverse eukaryotic lineages through multiple lateral gene transfer (LGT) events [48–51] and is known to produce ATP via substrate-level phosphorylation in the cytosol [52, 53]. Known exceptions include *Mastigamoeba balamuthi* (Amoebozoa: Archamoebae) and *Spironucleus salmonicida* (Metamonada: Fornicata), which both have ACS localized to their MROs [49, 50]. In silico predictions of the ADP-forming ACS identified in some apicomplexan lineages suggest it is localized into cytosol, where it can convert acetyl-CoA into acetate while generating ATP [13].

We recovered a complete or near complete TCA cycle for all lineages in this study, with the exception of several *Cryptosporidium* species (Fig. 2a; Additional file 15: Table S6). It has been reported that apicomplexans have replaced malate dehydrogenase (MDH) with malate:quinone oxidoreductase (MQO) [13]. We found that *Platyproteum*, a sister taxon to Apicomplexa and chrompodellids, has both MDH and MQO and Chrompodellids retain solely MDH, suggesting acquisition of MQO by an ancestor of Squirmidea, followed by the retention of MDH in Chrompodellids and MQO in Apicomplexans.

### Multiple independent losses of the electron transport chain

One of the most striking findings is the independent loss of multiple ETC complexes in at least three apicomplexan lineages. Functional reduction of *Cryptosporidium* MROs has been well characterized over the past few decades [13, 16, 54, 55]. Here, we report additional losses in three eugregarine lineages. Although archigregarines and blastogregarines are each represented by only one single-cell transcriptome, we recover a nearly complete electron transport chains for these early branching gregarines, with the few missing genes likely resulting from partial datasets (Fig. 2a). Inferring gene loss from transcriptomic data can be problematic, especially considering that life-stage-specific metabolic fluxes have been characterized for some apicomplexans like *Toxoplasma* and *Plasmodium*, which could cause genes to be absent from the transcriptomes [56–58]. However, given that gregarine life cycles are much less complex than that of the “core Apicomplexa,” and all analyzed datasets are based on sampling of the same life stage (trophozoites), differences between gregarine lineages are very unlikely to result from life-stage-specific metabolic fluxes. Even with these considerations, we observe strong patterns of ETC loss in eugregarines as not a single protein from ETC complexes III–V is recovered from Urosporoidea (*n* = 5), Porosporoidea (*n* = 2), or Gregarinoidea (*n* = 5, plus one genome), which all infect the host digestive system or coelom (Fig. 2a). However, this pattern is rendered non-monophyletic by Actinocephaloidea, which appears to maintain a fully functioning ETC (again, the few unrecovered genes are attributed to partial data). *Monocystis agilis* (Actinocephaloidea) infects the reproductive system of terrestrial worms and may have greater exposure to oxygen allowing it to generate ATP through oxidative phosphorylation, unlike the digestive tract-infecting eugregarines. Accordingly, we suspect the differential ETC loss in Urosporoidea, Porosporoidea, and Gregarinoidea is likely a result of advanced adaptation to the hypoxic conditions found within the digestive tract of their respective hosts. Furthermore, the lack of cytochrome b, cytochrome c oxidase subunit 1, or cytochrome c oxidase subunit 3 from Urosporoidea, Porosporoidea, and Gregarinoidea sequence data suggests a possible loss of the mitochondrial genome in these lineages. Similar patterns of mitochondrial genome loss and adaptation to anaerobiosis have been observed in *Cryptosporidium* and other eukaryotic gut endobionts throughout the tree of life [44, 45, 59–62]. Interestingly, the distribution of prokaryotes within the intestinal tract of the polychaete *Neanthes glandicincta* has shown increased density of aerobic species in the anterior, with anaerobes representing the majority in the posterior of the gut [63]. Accordingly, the retention of a complete ETC capable of oxidative phosphorylation in archigregarines and blastogregarines may be explained by their localization within the host digestive system; however, more data is necessary to test this hypothesis.

In most eukaryotes, ubiquinol (UQ) serves as an electron carrier through the electron transport chain. Curiously, we were unable to identify the majority of the enzymes involved in ubiquinol synthesis in eugregarines (transcriptomes from 13 species and the *G. niphandrodes* genome), with the exception of COQ3 which is conserved in all groups, and COQ6 which is found only in Actinocephaloidea. In few, but varied exceptions, anaerobes will instead use rhodoquinone (RQ) as an electron carrier [45, 64–66]. In aerobic conditions, complex II oxidizes succinate to fumarate, thereby transferring electrons for the reduction of ubiquinone to ubiquinol, while in anaerobic conditions complex II functions in the reverse, regenerating UQ by utilizing electrons from ubiquinol to reduce fumarate to succinate [67]. The lower redox potential of RQ over UQ enables rhodoquinone to transfer electrons via complex II and more favorably reduce fumarate to succinate [67]. However, endogenous production of RQ necessitates a source of UQ [68–70]. Utilization of exogenous UQ has been demonstrated in mice [71], and UQ obtained from bacterial prey has been proposed as the source of UQ which is then converted into RQ by the laterally acquired methyltransferase *rquA* in *Pygsuia* [66]. We were unable to identify *rquA* in any apicomplexans in this study negating this as a possibility for eugregarines (data not shown).

The conservation of COQ3 in eugregarines suggests its involvement in ubiquinol (UQ) production, but its synthesis otherwise remains a mystery. It is possible that eugregarines are utilizing COQ3 for alternative functions or that they may have lost the ability to fully synthesize ubiquinol like *Cryptosporidium ubiquitum* [43] and the presence of COQ3 may represent a final vestige of a fading UQ synthesis pathway. We can think of four possibilities to explain the absence of UQ biosynthesis proteins in eugregarines. (1) Either these proteins have become extremely divergent in eugregarines and were unable to be recovered in our searches; (2) eugregarines have replaced ubiquinol with another Q source, for which the synthesis pathway remains unknown (like RQ in the ciliate *Nyctotherus ovalis* [64]); (3) eugregarines are utilizing COQ3 to methylate exogenously acquired 3-demethylubiquinol-n for the final step of UQ production; or (4) exogenous Q could satisfy the UQ requirement for the complete ETC retained by Actinocephaloidea as shown in UQ-deficient mice [71], although the mechanism for its acquisition remains unclear [72]. The absence of dihydroorotate dehydrogenase (DHOD) in other eugregarines with a reduced ETC, and the additional loss of glycerol-3-phosphate dehydrogenase in Gregarinoidea suggests reduced Q dependency in these lineages (Additional file 15: Table S6). This reduced capacity for ubiquinone biosynthesis likely indicates host dependency for a source of Q in eugregarines. Along with the previously described ETC reductions, these findings support the hypothesis that patterns of reductive mitochondrial evolution in eugregarines were co-mediated by adaptions to parasitism and an anaerobic environment in a host digestive system.

The presence of ATP synthases has been associated with the formation of mitochondrial cristae [73], and a recent study identified 17 ATP synthase proteins that contribute to the curvature of cristae in the *Toxoplasma gondii* mitochondrion [74]. This study specifically implicated ATPTG11 (called ASAP19 by [75] and in Table S6) as essential to the structural architecture of *T. gondii* cristae [74]. Investigations of medically important apicomplexans like *T. gondii* have characterized a link between bioenergetics and cristae formation [74]. However, gregarines complicate our understanding of these structures despite there being relatively few ultrastructural studies compared to their vast diversity. Cristae have been observed in the mitochondria of some archigregarines and blastogregarines [21, 76, 77], which are both lineages that retain a complete ETC (Fig. 2). However, other archigregarines appear to lack cristae or present few structures called blistered vesicles that are connected to the inner membrane of the mitochondrion [78]. Surprisingly, members of lineages lacking an ETC in this study (e.g., Urosporoidea and Gregarinoidea) have also been shown to retain cristae [79–81]. We were unable to identify ATPGT11/ASAP19 in any gregarine lineage. Other proteins linked to cristae architecture [74] were identified only from archigregarines, blastogregarines, and actinocephaloideans (see ATPTG proteins in Additional file 15: Table S6), all of which retain a functional ETC. The presence of cristae in gregarine mitochondria that lack an ETC (particularly complex V—ATP synthase), as well as the lack of cristae in mitochondria of gregarines with a complete ETC, would be of particular interest in future investigations.

### Other mitochondrial pathways

Mitochondrial Fe-S cluster synthesis has been considered the core functional pathway preventing the complete loss of mitochondria/MROs in numerous lineages [54, 82–84]. Accordingly, *Monocercomonoides exilis* (Metamonada: Preaxostyla) is the only eukaryote to have completely lost the organelle and has replaced the mitochondrial ISC pathway with the bacterial SUF system through lateral gene transfer. This laterally acquired SUF system functions in the cytosol in *M. exilis* [83]. The *Pygsuia* ISC pathway was similarly replaced by a prokaryotic SUF system that shares a common origin with the fused SUFCB proteins identified in *Blastocystis* [85]. Additionally, *Mastigamoeba balamuthi* and *Entamoeba histolytica* (Amoebozoa: Archamoebae) have both replaced their ISC with a NIF pathway of prokaryotic origin [86–88]. However, in apicomplexans, even the highly reduced MROs of *C. parvum* and *C. hominis* retain a functional ISC system [54]. Although we did not find the complete suite of genes involved in the ISC pathway in any one gregarine, the overall patterns of gene retention support that this pathway remains functional in all gregarines studied to date. The findings here further support the role of the mitochondrial ISC pathway in the retention of MROs.

*Cryptosporidium* is known to have reduced metabolic capacity and has lost several pathways for amino acid and fatty acid metabolism [43]. Although we are relying primarily on single-cell transcriptomes, we identified much broader patterns of amino acid metabolism in the gregarine mitochondria compared to those of *Cryptosporidium* spp. For example, most *Cryptosporidium* species have lost all genes involved in the glycine cleavage system (GCS) [15]. Only the GCS L protein is retained in *C. andersoni*, *C. muris*, Porosporoidea, and Gregarinoidea; however, this protein is known to be widely involved in energy metabolism and maintaining redox balance, including its role as the E3 component in the BCKDH complex [15, 89]. In archigregarines, blastogregarines, urosporideans, and actinocephaloideans, we identified a complete GCS pathway (Table S6). Aside from the GCS pathway, we found cystathione beta synthase, alanine aminotransferase, glutamate dehydrogenase, and several other proteins involved in amino acid and fatty acid metabolism are conserved across gregarine lineages.

### The role of lateral gene transfer in apicomplexan mitochondrial metabolism

Lateral gene transfer (LGT) has been implicated as an important driver in environmental adaptation with an ever-increasing number of well-supported events occurring in eukaryotes [50, 51, 66, 90–92]. Here, we show that ADP-forming acetyl-CoA synthetase (ACS) has been acquired by the ancestor of chrompodellids and Apicomplexa by LGT from a prokaryotic donor (Additional file 17: Fig. S11). The acquisition of this ADP-forming ACS likely facilitates ATP production via substrate-level phosphorylation in the cytosol, providing flexibility in their metabolic capacity under dynamic environmental conditions, as in *Giardia intestinalis* [45].

We also observe strong support for an independent origin of malic enzyme in the gregarines sequenced for this study. Malic enzyme converts malate to pyruvate which is subsequently converted to acetyl-CoA and can be utilized by the TCA cycle. Seeber et al. [15] proposed that in *C. parvum* and *C. hominis*, the NADH generated, in part by malic enzyme, can act as a proton pump and lead to a simplified respiratory chain comprised of NAD(P) transhydrogenase, NDH2, and AOX. Malic enzyme is widely conserved throughout Apicomplexa; however, it appears to have been lost in the ancestor to Gregarinoidea and remains absent in *G. niphandroides*. Interestingly, the cockroach-infecting gregarines sequenced here have replaced the missing malic enzyme through an LGT from proteobacteria (Additional file 18: Fig. S12).

The ancestor of the Apicomplexa lost the TCA cycle protein MDH, which has been replaced by the acquisition of MQO through an LGT from epsilon-proteobacteria [13, 93]. We identified MQO in all apicomplexan lineages except Urospororidea, and Porospororidea, which are represented solely by single-cell transcriptome data, so we consider its absence inconclusive. Both MDH and MQO have been observed in Squirmidea (*Platyproteum*) and two chrompodellids (*Alphamonas* and *Vitrella*). The enzymes may be functioning independently based on cellular conditions as was observed in *E. coli* [94], or collaboratively as in *Corynebacterium glutamicum* [95]. Combined, these three LGT events represent important adaptations to mitochondrial metabolism in apicomplexans. Future investigations of understudied apicomplexans will help to pinpoint the origins of adaptive LGTs, reveal patterns of organellar evolution and genomic adaptions to specific host environments, and establish the order of transitions between intracellular and extracellular lifestyles, and those between marine and terrestrial hosts.

## Conclusions

Our phylogenomic analyses place *Cryptosporidium* as a sister lineage to the rest of Apicomplexa, with gregarines and core apicomplexans each forming fully supported clades. However, deeper analysis highlights that the branching pattern observed in the early apicomplexan radiation remains highly contentious, and the topology of gregarines forming sister lineage to a clade of *Cryptosporidium* and core apicomplexans cannot be fully refuted. Regardless of whether *Cryptosporidium* or gregarines diverged first, this evolutionary framework suggests that apicomplexans are evolutionarily malleable, with phenotypic adaptations being driven by the host environment. Intracellularity has evolved at least three times independently within the Apicomplexa: once in *Cryptosporidium*, again in Coccidia, and a third time in the ancestor of Haemospororida and Piroplasmorida. Our phylogeny also supports a propensity for lifestyle transitions specifically within gregarines, with at least two transitions between marine and terrestrial hosts, as well as adaptations to infect different host organs. Additionally, based on our finding that *Cryptosporidium* represents an evolutionarily distinct lineage from gregarines, it should no longer be considered part of the Gregarinasina or the Gregarinomorphea (as in Adl et al. [96] or Cavalier-Smith [20]).

We characterized mitochondrial metabolism across the Apicomplexa and observed two distinct modes of metabolism within gregarines. Blastogregarines, archigregarines, and Actinocephaloidea possess mitochondria with a fully functioning electron transport chain, akin to core apicomplexans. All other eugregarines appear to lack a mitochondrial genome and have completely lost complexes III–V of the ETC and BCKDH as part of a switch to anaerobic mitochondrial metabolism. Here, pyruvate is converted to acetyl-CoA by PNO or PFL. Acetyl-CoA can then be shuttled to the organelle via acetyl-CoA transporter, utilized by the TCA cycle, and NADH is then oxidized by a reduced respiratory chain consisting of NDH2 and AOX. Alternatively, acetyl-CoA can be metabolized in the cytosol to acetate by acetyl-CoA synthetase, while generating ATP. The mitochondrial or cytosolic localization of PNO/PFL enzymes and acetyl-CoA will help us to fully understand the metabolism in these gregarine lineages. These two types of gregarine mitochondrial metabolism are not restricted to monophyletic groups and the electron transport chain has been reduced at least three times during apicomplexan evolution, twice within gregarines.

We hypothesize that the observed distribution of mitochondrial reduction within gregarines corresponds with their adaptation to an anaerobic environment within their hosts and dependence on the host itself. Actinocephaloidea, blastogregarines, and archigregarines retained seemingly regular mitochondrial functions, while the other eugregarines have twice independently transitioned to functionally reduced mitochondria. The pattern of adaptation corresponds well with the first hypothetical step toward anaerobiosis—loss of complexes III and IV [62]. This has been also observed in many other gut-inhabiting endobionts across the tree of life, including *Cryptosporidium* [44, 45, 62]*.* Moreover, these eugregarines have completely lost complex V and BCKDH. Instead, they possess PNO, which converts pyruvate to acetyl-CoA in anaerobes [44, 96], and ADP-forming acetyl-CoA synthetase that likely facilitates cytosolic substrate-level phosphorylation, producing acetate as the final metabolic product.

Recent advances in single-cell sequencing have opened the door to genomic characterization of unculturable lineages throughout the tree of life. Unsurprisingly, these studies provide profound insight into the understanding of evolution, ecology, and biochemistry. Gregarines provide a textbook example, and until now, their uncertain placement within Apicomplexa hindered our understanding of evolutionary events within this clade of medically important parasites. Gregarine metabolism has been shaped by adaptions to parasitism and anaerobiosis, and as such, the mitochondrial remodeling observed in distinct gregarine lineages highlights the importance of this group to our understanding of organellar evolution and retention in eukaryotes. Future investigations of under-explored apicomplexans will further help to pinpoint the origins of adaptive LGTs, establish the order of transitions between intra- and extracellular lifestyles, as well as host habitats, and continue to reveal patterns of organellar evolution and genomic adaptions to specific host environments.

## Materials and methods

### Specimen collection, RNA isolation and sequencing, and assembly

Cockroach individuals were obtained from cultures housed in the Department of Zoology, Charles University, Czech Republic. *Gromphadorhina portentosa*, *Gyna caffrorum*, and *Nauphoeta cinerea* originated from laboratory cultures of unknown origin. A colony of *Polyphaga aegyptiaca* was established in 2012 from individuals collected on Corfu, Greece, and a colony of *Pseudoderopeltis* sp. was established from individuals collected in 2013 in Ethiopia. Individual gregarine trophozoites were hand-picked from *G. caffrorum*, *P. aegyptiaca*, *Pseudoderopeltis* sp., and *N. cinerea* on an Olympus IX70 inverted microscope using hand-drawn glass pipettes. The gregarines were washed 3 times in phosphate-buffered saline (PBS) and placed in a 0.2-ml tube containing 2 μl of cell lysis buffer for the SmartSeq2 protocol [97], flash frozen, and stored in a − 80 °C freezer. Single-cell RNA isolation, cDNA generation, and amplification were completed according to the SmartSeq2 protocol with only 11 amplification cycles [97]. Heavily infected individuals of *Gromphadorhina portentosa* and *Polyphaga aegyptiaca* were dissected and the gut contents were emptied into a petri dish with PBS. Under a Meopta DM23 dissecting microscope, 40–60 trophozoites were transferred by pipette to a clean dish of PBS to wash the cells of other gut content and host tissue. Cells were then transferred to a 2-ml tube and total RNA was extracted using the RNAqueous RNA isolation kit (Invitrogen) following the manufacturer protocol. The resulting total RNA from 1 extraction of gregarines from *P. aegyptiaca* (the first isolation of a gregarine from a Polyphagidae cockroach), 2 extractions of *Blabericola migrator* isolated from *G. portentosa*, as well as cDNA independently generated from 3 gregarines from *Pseudoderopeltis* sp., 2 *Gregarina* sp. Poly, 2 gregarines isolated from *Gyna caffrorum*, and 2 *Protomagalhaensia wolfi* isolated from *N. cinerea* were sent to Macrogen for library preparation and sequencing. Nextera XT libraries were generated for all single cells and TruSeq stranded mRNA libraries were generated for total RNA. Libraries were sequenced on an Illumina NexSeq producing 30 million 150 bp paired-end reads per library. The resulting reads were checked for quality using FastQC. Low-quality bases and adapters were removed using Trimmomatic [98]. The remaining reads were assembled using rnaSPAdes v3.13 [99], and Transdecoder was used for ORF prediction and translation. Completeness of all transcriptomes generated in this study, along with all public apicomplexan data used for phylogenomic analyses, was assessed using BUSCO with the Apicomplexa gene set (Additional file 1: Table S1) [100]. Gregarine species identifications were confirmed (> 99% identity) or remain ambiguous (< 99% identity, e.g., *Protomagalhaensia* sp. Gyna) based on BLAST searches of the SSU rRNA sequences recovered from the gregarine transcriptomes and general morphology.

### Phylogenomic dataset construction and analyses

The gene sets used in two independently published phylogenomic studies of gregarines were utilized for our analyses [22, 23]. All publicly available Apicomplexan genomes/transcriptomes [101–105] were considered for initial ortholog selection in each gene set and run through BUSCO [100] to assess completeness (see Additional file 1: Table S1). Prior to assembling phylogenomic datasets, our assemblies were run through Winston Cleaner [106] to identify and highlight likely cases of cross contamination. To further assist in the identification of contamination and paralogs, transcriptomes and genomes spanning the diversity of the eukaryotic tree of life were included in the initial “unparsed” datasets which are available on Figshare [107]. A BLAST database was constructed from all proteins considered for phylogenomic analyses (Additional file 1: Table S1). A representative seed sequence for each gene was used as a query to identify putative orthologs by BLAST search (*e*-value < 1e−10). Up to the top five blast hits for each species in the dataset were combined with their respective genes to form a 246-gene dataset that corresponded to the Burki et al. [25] dataset used by Mathur et al. [23] and the 307-gene dataset from Derrelle et al. [27] used by Janouškovec et al. [22]. For each dataset, the individual genes were filtered for sequencing errors and non-homologous sites using PREQUAL [108], aligned using MAFFT (--globalpair --maxiterate 1000) [109], alignment uncertainty and errors were filtered using DIVVIER (--partial -mincol 4 -divvygap) [110], and the filtered alignments were trimmed of sites comprised of > 80% gaps using trimAL (-gt 0.2) [111]. Trees were constructed from the trimmed alignments using RAxML (PROTGAMMALG with 100 rapid bootstraps) [112]. Each tree was then carefully manually inspected to identify the correct ortholog for each species and remove paralogous sequences and contaminations. In the case of inparalogs (gene duplications strictly within a single organism), the longest sequence or sequence with the shortest branch length was selected as the ortholog. For midparalogs, only the side of the inferred gene duplication that contained the most data (as the total sum of retained sequence length of selected orthologs) based on the tree was used for ortholog selection, even if this led to a loss of a specific taxon for the particular gene. To clarify, if one side of an inferred duplication had 7 taxa that were each 50% complete, while the other side contained 5 taxa that were all 90% complete, the side with 5 taxa was selected. In the event the total amount of retained data was similar for each side a duplication, the side with greater representation of species was selected (e.g., a side with 9 taxa that average 50% complete would be chosen over a side with 7 taxa that average 65% complete). Once orthologs for all taxa were identified, ortholog-only datasets were filtered and aligned according to the previously described methods and were then trimmed using BMGE (-g 0.3) [113]. Final taxon and gene selection were carried out on these alignments with a threshold of 80% missing data to maximize representation in each dataset and to accommodate for the inclusion of important taxa (e.g., *Eimeria falciformis*). Our final phylogenomic datasets were comprised of 246 genes (63,201 sites) (corresponding to Mathur et al. [23]) and 299 genes (89,675 sites) (corresponding to Janouškovec et al. [22]) and 74 taxa. These datasets were once again filtered, aligned, and trimmed in the same manner. The resulting alignments were concatenated into a supermatrix (one for each dataset), partitioned using PartitionFinder2 under the LG+G model [114], and subjected to maximum likelihood analysis in IQtree (LG+C60+F+G) [115] with 1000 PMSF bootstrap replicates. Bayesian inference was conducted in Phylobayes [116] under the CAT-GTR model with 2 chains for each dataset run for 7000 generations each (burnin = 20%; maxdiff = 1 and 0.081 for datasets A and B, respectively). The average absolute difference in gene-wise log-likelihood scores, as calculated in IQtree under the LG+C20+G model, for the A+C, A+G, and G+C topologies was calculated following Shen et al. [30] for each dataset. After removing outlier genes identified in each dataset, maximum likelihood trees were reconstructed in IQtree (LG+C60+F+G) [115] with 100 PMSF bootstrap replicates. Additional datasets comprised of genes unique to dataset A, genes unique to dataset B, and genes shared between the two datasets were also subjected to maximum likelihood analysis in IQtree (LG+C60+F+G). Additionally, maximum likelihood trees for the full datasets were constructed under the LG model were computed using IQtree (LG+F+G) with 1000 ultrafast bootstraps. Single gene datasets including all considered sequences (i.e., including orthologs and paralogs [35];) and ortholog-only datasets are available on figshare [107]. Log files for phylogenomic analyses containing commands and processor information are also available on figshare [117] as recommended by Shen et al. [118].

### Removal of fast evolving and heterotacheous sites, and random gene subsampling

For each of our phylogenomic datasets, PhyloFisher scripts [119] were used for fast site removal, heterotacheous site removal, and random gene subsampling. The fastest evolving sites were sequentially removed in 6000 site chunks generating new alternative datasets at each step until all sites were removed (as in [31, 32]). Similarly, the most heterotacheous sites [33] were removed in a stepwise fashion, 6000 sites at a time, producing iteratively smaller datasets until no further sites could be removed. Genes from our 74 taxon datasets were randomly subsampled in sets of 20%, 40%, 60%, and 80% of the complete dataset, under the default 95% confidence interval setting as in Brown et al. [31]. The dataset comprised of genes unique to dataset A was similarly subjected to fast site removal, with 3000 sites being removed at each step. All datasets generated by these scripts were then subjected to phylogenetic analysis using IQtree under the LG+C60+F+G model with 100 non-parametric bootstrap replicates generated using PMSF and the fast setting in IQtree. In the case of fast evolving site removal under the LG model, 100 rapid bootstraps were generated using RAxML v8 (PROTCATLGF).

### Constrained trees, concordance factors, and topology tests

Both 74 taxon datasets used for the initial phylogenomic analyses, were subjected to constrained maximum likelihood tree reconstruction in IQtree (LG+C60+F+G) using the same partitioning scheme applied in our initial analyses. The overall topologies published in Mathur et al. [23] (i.e., gregarines are sister to other apicomplexans) and Janouškovec et al. [22] (monophyletic gregarines and *Cryptosporidium* spp. as sister to other apicomplexans) were used as constraints. Gene concordance factors (gCF) for the best ML tree for our initial phylogenomic analysis and the constrained topologies were calculated using IQtree [29]. Additionally, the approximately unbiased (AU) test [120] was conducted on the maximum likelihood tree, the constrained trees, and 100 distinct local topologies saved during the initial ML analysis (-wt option in IQtree) as implemented by IQtree [115].

### In silico predictions of mitochondrial metabolism

Genes of interest were identified from previously published studies of anaerobic mitochondria, the published mitochondrial proteome of *Tetrahymena thermophila* [121], and recent publications on mitochondrial metabolism of Apicomplexa [15, 51, 61, 65, 75, 85, 122–124]. Candidate sequences from our gregarine data and other publicly available genome-scale datasets for apicomplexans and their close relatives (Additional file 1: Table S1) were recovered by BLAST using representative seed sequences and collecting hits with *e*-value <1e−5 were extracted and added to the initial datasets for each gene. When available, datasets published by Rotterova et al. [61, 124] were used as starting datasets. Alternatively, datasets for phylogenetic analysis were constructed by using BLAST searches of the seed sequences against nr (max target seqs = 100) to thoroughly sample apicomplexans and other close relatives. These BLAST results were supplemented with sequences selected from gene name search results from the NCBI protein and RefSeq databases to ensure broad eukaryotic and prokaryotic taxonomic sampling and were then clustered using CD-Hit [125] at 75%. Each gene dataset was filtered for sequencing errors and non-homologous sites using PREQUAL, aligned using MAFFT (--localpair --maxiterate 1000), and alignment uncertainties and errors were filtered using DIVVIER (-partial -mincol 4 -divvygap) and single gene trees were constructed using IQtree (LG+C20+F+G) with 100 RAxML rapid bootstraps using constructed under the PROTGAMMALG4X model. Each alignment/tree has been repeatedly inspected by the eye, and sequences were added/removed as necessary. Ultimately, each tree was manually inspected, enabling precise inference of the presence/absence of proteins involved in mitochondrial metabolism in the studied lineages. Prediction of subcellular localization for all sequences identified as apicomplexan homologs in this study was conducted in TargetP v2 [126] and DeepLoc [127]. Files containing all initially considered sequences, final datasets, final alignments, sequences of all selected Apicomplexan homologs, the resulting trees (raw and colored to show selected apicomplexan homologs), and the subcellular localization prediction results for all mitochondrial genes discussed in this study are available on figshare [128].

## Supplementary Information

**Additional file 1: Table S1.** Sources, uses, and BUSCO completeness for data used in this study.

**Additional file 2: Fig. S1.** Maximum likelihood phylogeny of apicomplexans as recovered from dataset A, comprised of 246 genes and 63,201 sites. The final dataset was partitioned under the LG+G model in PartitionFinder2 and then subjected to maximum likelihood analysis implemented in IQtree (LG+C60+F+G). Non-parametric PMSF bootstrap support values (*n* = 1000) are shown on the branches.

**Additional file 3: Fig. S2.** Maximum likelihood phylogeny of apicomplexans as recovered from dataset B, comprised of 299 genes and 89,675 sites. The final dataset was partitioned under the LG+G model in PartitionFinder2 and then subjected to maximum likelihood analysis implemented in IQtree (LG+C60+F+G). Non-parametric PMSF bootstrap support values (n = 1000) are shown on the branches.

**Additional file 4: Fig. S3.** Bayesian inference (BI) consensus tree for apicomplexans as recovered from dataset A, comprised of 246 genes and 63,201 sites. The final dataset was subjected to two chains of BI in Phylobayes for 7000 generations with every second generation sampled and a burnin of 20%. The two chains for dataset A did not converge (maxdiff = 1) and their topologies are independently shown as Figs. S7 and S8. Support values listed on branches are posterior probabilities from the analysis.

**Additional file 5: Fig. S4.** Bayesian inference (BI) tree for chain 1 from dataset A, comprised of 246 genes and 63,201 sites. The final dataset was subjected to two chains of BI in Phylobayes for 7000 generations with every second generation sampled and a burnin of 20%. Support values listed on branches are posterior probabilities from the analysis.

**Additional file 6: Fig. S5.** Bayesian inference (BI) tree for chain 2 from dataset A, comprised of 246 genes and 63,201 sites. The final dataset was subjected to two chains of BI in Phylobayes for 7000 generations with every second generation sampled and a burnin of 20%. Support values listed on branches are posterior probabilities from the analysis.

**Additional file 7: Fig. S6.** Bayesian inference (BI) consensus tree for apicomplexans as recovered from dataset B, comprised of 299 genes and 89,675 sites. The final dataset was subjected to two chains of BI in Phylobayes for 7000 generations with every second generation sampled and a burnin of 20%. The two chains for dataset B converged with maxdiff = 0.081. Support values listed on branches are posterior probabilities from the analysis.

**Additional file 8: Table S2.** Gene concordance factors for possible apicomplexan relationships.

**Additional file 9: Table S3.** Approximately unbiased test scores for possible apicomplexan relationships.

**Additional file 10: Table S4.** Genes unique and overlapping between phylogenomic datasets A and B.

**Additional file 11: Table S5.** Maximum likelihood topologies produced by unique and shared genes from datasets A and B.

**Additional file 12: Fig. S7.** Effects of fast evolving site removal on support for bipartitions of interest in the phylogenomic analyses of genes that are unique to dataset A. Graphs plotting support for bipartitions of interest after the stepwise removal of the 3000 fastest evolving sites until all sites are removed from each dataset (dataset A on the top and dataset B on the bottom). 100 RAxML rapid bootstraps values (PROTCATLGF) are on the y-axis and number of sites removed, measured in thousands, is shown on the x-axis.

**Additional file 13: Fig. S8.** Effects of fast evolving site removal on support for bipartitions of interest in the phylogenomic analyses of each dataset under the less complex LG model. Graphs plotting support for bipartitions of interest after the stepwise removal of the 6000 fastest evolving sites until all sites are removed from each dataset (dataset A on the top and dataset B on the bottom). 100 RAxML rapid bootstraps values (PROTCATLGF) are on the y-axis and number of sites removed, measured in thousands, is shown on the x-axis.

**Additional file 14: Fig. S9.** Box-and-whisker plots showing the difference in gene likelihood scores (**Δ**GLS) dataset A and dataset B. **Δ**GLS values for genes that plot above the upper whisker are shown with the preferred topology of the gene tree (A + C = core apicomplexans with *Cryptosporidium*; A + G = core apicomplexans with gregarines; G + C = gregarines with *Cryptosporidium*).

**Additional file 15: Table S6.** Presence-absence table for genes involved in apicomplexan mitochondrial metabolism.

**Additional file 16: Fig. S10.** Maximum likelihood trees for all mitochondrial genes discussed in this study inferred using IQtree (LG+C20+F+G). Support values are calculated from 100 RAxML rapid bootstraps under the PROTGAMMALG model. Sequences identified as apicomplexan homologs are colored in red, sequences in purple are suspected apicomplexan contamination of a host rather than the target organism.

**Additional file 17: Fig. S11.** Maximum likelihood phylogeny of ADP-forming ACS reconstructed in IQtree (LG+C20+F+G) with support values from 100 non-parametric PMSF bootstrap replicates. Prokaryotes are blue, Apicomplexa, Squirmidea and chrompodellids are colored red, and other eukaryotes are shown in black. The topology shows moderately strong support for a laterally transferred ADP-forming ACS gene of prokaryotic origin to the shared ancestor of chrompodellids and apicomplexans.

**Additional file 18: Fig. S12.** Maximum likelihood phylogeny of malic enzyme reconstructed in IQtree (LG+C20+F+G) with support values from 100 non-parametric PMSF bootstrap replicates. Prokaryotes are blue, Apicomplexa, Squirmidea and chrompodellids are colored red, and other eukaryotes are shown in black. The topology shows strong support for an independent acquisition of malic enzyme by members of the Gregarinoidea through lateral gene transfer from proteobacteria. The malic enzyme sequenced in other eugregarines branches with members of the core apicomplexans, chrompodellids, and Squirmidea.

## Data Availability

All raw read sequencing data have been deposited into NCBI SRA archive under BioProject PRJNA684345 (SRA accessions SRR13276162-SRR13276175). All nucleotide assemblies for newly generated data are deposited in figshare at 10.6084/m9.figshare.13350524.v1. The predicted protein fasta files used in BLAST databases in this study (see Additional file 1: Table S1) are deposited in figshare at 10.6084/m9.figshare.13344884.v1. Single gene datasets for all initially considered sequences and final orthologs used in phylogenomic analyses and a list of putative cross contamination sequences for each gene are deposited in figshare at 10.6084/m9.figshare.13344227.v1. Log files for all phylogenomic analyses, single gene datasets for all initially considered sequences, and final orthologs used in phylogenomic analyses are deposited in figshare at 10.6084/m9.figshare.13927292.v2. Single gene datasets comprised of all initially considered sequences, final datasets, apicomplexan homolog, trees (raw and with apicomplexan homologs colored), and subcellular localizations based on TargetP and DeepLoc for all mitochondrial genes discussed here are deposited in figshare at 10.6084/m9.figshare.13928252.v1 [129].

## References

[1] Gardner MJ, Hall N, Fung E, White O, Berriman M, Hyman RW, Carlton JM, Pain A, Nelson KE, Bowman S, Paulsen IT, James K, Eisen JA, Rutherford K, Salzberg SL, Craig A, Kyes S, Chan MS, Nene V, Shallom SJ, Suh B, Peterson J, Angiuoli S, Pertea M, Allen J, Selengut J, Haft D, Mather MW, Vaidya AB, Martin DMA, Fairlamb AH, Fraunholz MJ, Roos DS, Ralph SA, McFadden GI, Cummings LM, Subramanian GM, Mungall C, Venter JC, Carucci DJ, Hoffman SL, Newbold C, Davis RW, Fraser CM, Barrell B (2002). Genome sequence of the human malaria parasite *Plasmodium falciparum*. Nature..

[2] Xu P, Widmer G, Wang Y, Ozaki LS, Alves JM, Serrano MG, Puiu D, Manque P, Akiyoshi D, Mackey AJ, Pearson WR, Dear PH, Bankier AT, Peterson DL, Abrahamsen MS, Kapur V, Tzipori S, Buck GA (2004). The genome of *Cryptosporidium hominis*. Nature..

[3] del Campo J, Heger TJ, Rodríguez-Martínez R, Worden AZ, Richards TA, Massana R, Keeling PJ (2019). Assessing the diversity and distribution of apicomplexans in host and free-living environments using high-throughput amplicon data and a phylogenetically informed reference framework. Front Microbiol.

[4] Carlton JM, Angiuoli SV, Suh BB, Kooij TW, Pertea M, Silva JC, Ermolaeva MD, Allen JE, Selengut JD, Koo HL, Peterson JD, Pop M, Kosack DS, Shumway MF, Bidwell SL, Shallom SJ, van Aken SE, Riedmuller SB, Feldblyum TV, Cho JK, Quackenbush J, Sedegah M, Shoaibi A, Cummings LM, Florens L, Yates JR, Raine JD, Sinden RE, Harris MA, Cunningham DA, Preiser PR, Bergman LW, Vaidya AB, van Lin LH, Janse CJ, Waters AP, Smith HO, White OR, Salzberg SL, Venter JC, Fraser CM, Hoffman SL, Gardner MJ, Carucci DJ (2002). Genome sequence and comparative analysis of the model rodent malaria parasite *Plasmodium yoelii yoelii*. Nature..

[5] Mather M, Henry K, Vaidya A (2007). Mitochondrial drug targets in apicomplexan parasites. Curr Drug Targets.

[6] Ke H, Mather MW (2017). +Targeting mitochondrial functions as antimalarial regime, what is next?. Curr Clin Microbiol Rep.

[7] Vaidya AB, Mather MW (2009). Mitochondrial evolution and functions in malaria parasites. Annu Rev Microbiol.

[8] Hikosaka K, Kita K, Tanabe K (2013). Diversity of mitochondrial genome structure in the phylum Apicomplexa. Mol Biochem Parasit.

[9] Sheiner L, Vaidya AB, McFadden GI (2013). The metabolic roles of the endosymbiotic organelles of *Toxoplasma* and *Plasmodium* spp. Curr Opin Microbiol.

[10] Lim L, McFadden GI (2010). The evolution, metabolism and functions of the apicoplast. Philos T Roy Soc B..

[11] Ralph SA, D’Ombrain MC, McFadden GI (2001). The apicoplast as an antimalarial drug target. Drug Resist Update.

[12] Biagini GA, Viriyavejakul P, O’Neill PM, Bray PG, Ward SA (2006). Functional characterization and target validation of alternative complex I of *Plasmodium falciparum* mitochondria. Antimicrob Agents Ch.

[13] Mogi T, Kita K (2010). Diversity in mitochondrial metabolic pathways in parasitic protists *Plasmodium* and *Cryptosporidium*. Parasitol Int.

[14] Oppenheim RD, Creek DJ, Macrae JI, Modrzynska KK, Pino P, Limenitakis J, Polonais V, Seeber F, Barrett MP, Billker O, McConville MJ, Soldati-Favre D (2014). BCKDH: the missing link in apicomplexan mitochondrial metabolism is required for full virulence of *Toxoplasma gondii* and *Plasmodium berghei*. Plos Pathog.

[15] Seeber F, Limenitakis J, Soldati-Favre D (2008). Apicomplexan mitochondrial metabolism: a story of gains, losses and retentions. Trends Parasitol.

[16] Henriquez FL, Richards TA, Roberts F, McLeod R, Roberts CW (2005). The unusual mitochondrial compartment of *Cryptosporidium parvum*. Trends Parasitol.

[17] Rueckert S, Betts EL, Tsaousis AD (2019). The symbiotic spectrum: where do the gregarines fit?. Trends Parasitol.

[18] Boisard J, Florent I (2020). Why the –omic future of Apicomplexa should include gregarines. Biol Cell.

[19] Rueckert S, Villette PMAH, Leander BS (2011). Species boundaries in gregarine apicomplexan parasites: a case study-comparison of morphometric and molecular variability in *Lecudina* cf. *tuzetae* (Eugregarinorida, Lecudinidae). J Eukaryot Microbiol.

[20] Cavalier-Smith T (2014). Gregarine site-heterogeneous 18S rDNA trees, revision of gregarine higher classification, and the evolutionary diversification of Sporozoa. Eur J Protistol.

[21] Wakeman KC, Heintzelman MB, Leander BS (2014). Comparative ultrastructure and molecular phylogeny of *Selenidium melongena* n. sp. and *S. terebellae* Ray 1930 demonstrate niche partitioning in marine gregarine parasites (Apicomplexa). Protist..

[22] Janouškovec J, Paskerova GG, Miroliubova TS, Mikhailov KV, Birley T, Aleoshin VV, Simdyanov TG. Apicomplexan-like parasites are polyphyletic and widely but selectively dependent on cryptic plastid organelles. eLife. 2019;8 10.7554/eLife.49662. 10.7554/eLife.49662 PMC6733595 31418692

[23] Mathur V, Kolísko M, Hehenberger E, Irwin NAT, Leander BS, Kristmundsson Á, et al. Multiple independent origins of apicomplexan-like parasites. Curr Biol. 2019;29:2936–2941.e5. 10.1016/j.cub.2019.07.019. 10.1016/j.cub.2019.07.019 31422883

[24] Templeton TJ, Enomoto S, Chen W-J, Huang C-G, Lancto CA, Abrahamsen MS, Zhu G (2010). A genome-sequence survey for *Ascogregarina taiwanensis* supports evolutionary affiliation but metabolic diversity between a gregarine and *Cryptosporidium*. Mol Biol Evol.

[25] Burki F, Kaplan M, Tikhonenkov DV, Zlatogursky V, Minh BQ, Radaykina LV, et al. Untangling the early diversification of eukaryotes: a phylogenomic study of the evolutionary origins of Centrohelida, Haptophyta and Cryptista. P Roy Soc B-Biol Sci. 2016;283:20152802. 10.1098/rspb.2015.2802. 10.1098/rspb.2015.2802 PMC4795036 26817772

[26] Strassert JFH, Jamy M, Mylnikov AP, Tikhonenkov DV, Burki F. New phylogenomic analysis of the enigmatic phylum *Telonemia* further resolves the eukaryote tree of life. Mol Biol Evol. 2019;36:757–65. 10.1093/molbev/msz012. 10.1093/molbev/msz012 PMC6844682 30668767

[27] Derelle R, López-García P, Timpano H, Moreira D (2016). A phylogenomic framework to study the diversity and evolution of Stramenopiles (=Heterokonts). Mol Biol Evol.

[28] Simdyanov TG, Guillou L, Diakin AY, Mikhailov KV, Schrével J, Aleoshin VV (2017). A new view on the morphology and phylogeny of eugregarines suggested by the evidence from the gregarine *Ancora sagittata* (Leuckart, 1860) Labbé, 1899 (Apicomplexa: Eugregarinida). PeerJ..

[29] Minh BQ, Hahn MW, Lanfear R. New methods to calculate concordance factors for phylogenomic datasets. Mol Biol Evol. 2020;37:2727–33. 10.1093/molbev/msaa106. 10.1093/molbev/msaa106 PMC7475031 32365179

[30] Shen X-X, Hittinger CT, Rokas A (2017). Contentious relationships in phylogenomic studies can be driven by a handful of genes. Nat Ecol Evol..

[31] Brown MW, Heiss AA, Kamikawa R, Inagaki Y, Yabuki A, Tice AK, Shiratori T, Ishida KI, Hashimoto T, Simpson AGB, Roger AJ (2018). Phylogenomics places orphan protistan lineages in a novel eukaryotic super-group. Genome Biol Evol.

[32] Heiss AA, Kolisko M, Ekelund F, Brown MW, Roger AJ, Simpson AGB (2018). Combined morphological and phylogenomic re-examination of malawimonads, a critical taxon for inferring the evolutionary history of eukaryotes. Roy Soc Open Sci.

[33] Philippe H, Zhou Y, Brinkmann H, Rodrigue N, Delsuc F (2005). Heterotachy and long-branch attraction in phylogenetics. BMC Evol Biol.

[34] Wang H-C, Susko E, Roger AJ (2019). The relative importance of modeling site pattern heterogeneity versus partition-wise heterotachy in phylogenomic inference. Syst Biol.

[35] Salomaki ED, Eme L, Brown MW, Kolisko M (2020). Releasing uncurated datasets is essential for reproducible phylogenomics. Nat Ecol Evol..

[36] Leitch GJ, He Q (2012). Cryptosporidiosis-an overview. J Biomed Res.

[37] Black MW, Boothroyd JC (2000). Lytic cycle of *Toxoplasma gondii*. Microbiol Mol Biol R..

[38] Baer K, Klotz C, Kappe SHI, Schnieder T, Frevert U (2007). Release of hepatic *Plasmodium yoelii* merozoites into the pulmonary microvasculature. Plos Pathog.

[39] Patten R (1936). Notes on a new protozoon, *Piridium sociabile* n.gen., n.sp., from the foot of *Buccinum undatum*. Parasitology..

[40] Stairs CW, Roger AJ, Hampl V (2011). Eukaryotic pyruvate formate lyase and its activating enzyme were acquired laterally from a Firmicute. Mol Biol Evol.

[41] Foth BJ, Stimmler LM, Handman E, Crabb BS, Hodder AN, McFadden GI (2004). The malaria parasite *Plasmodium falciparum* has only one pyruvate dehydrogenase complex, which is located in the apicoplast: the single, plastidic PDH of *Plasmodium falciparum*. Mol Microbiol.

[42] Ctrnacta V, Ault JG, Stejskal F, Keithly JS. Localization of pyruvate: NADP+ oxidoreductase in sporozoites of *Cryptosporidium parvum*. The J Eukaryot Microbiol. 2006;53:225–31. 10.1111/j.1550-7408.2006.00099.x. 10.1111/j.1550-7408.2006.00099.x 16872290

[43] Liu S, Roellig DM, Guo Y, Li N, Frace MA, Tang K, Zhang L, Feng Y, Xiao L (2016). Evolution of mitosome metabolism and invasion-related proteins in *Cryptosporidium*. BMC Genomics.

[44] Stairs CW, Leger MM, Roger AJ (2015). Diversity and origins of anaerobic metabolism in mitochondria and related organelles. Philos T Roy Soc B.

[45] Muller M, Mentel M, van Hellemond JJ, Henze K, Woehle C, Gould SB, Yu RY, van der Giezen M, Tielens AGM, Martin WF (2012). Biochemistry and evolution of anaerobic energy metabolism in eukaryotes. Microbiol Mol Biol R.

[46] Guo F, Zhang H, Payne HR, Zhu G. Differential gene expression and protein localization of *Cryptosporidium parvum* fatty Acyl-CoA synthetase isoforms. J Eukaryot Microbiol. 2016;63:233–46. 10.1111/jeu.12272. 10.1111/jeu.12272 PMC4775295 26411755

[47] Dubois D, Fernandes S, Amiar S, Dass S, Katris NJ, Botté CY, Yamaryo-Botté Y (2018). *Toxoplasma gondii* acetyl-CoA synthetase is involved in fatty acid elongation (of long fatty acid chains) during tachyzoite life stages. J Lipid Res.

[48] Field J, Rosenthal B, Samuelson J. Early lateral transfer of genes encoding malic enzyme, acetyl-CoA synthetase and alcohol dehydrogenases from anaerobic prokaryotes to *Entamoeba histolytica*. Mol Microbiol. 2000;38:446–55. 10.1046/j.1365-2958.2000.02143.x. 10.1046/j.1365-2958.2000.02143.x 11069669

[49] Jerlström-Hultqvist J, Einarsson E, Xu F, Hjort K, Ek B, Steinhauf D, Hultenby K, Bergquist J, Andersson JO, Svärd SG (2013). Hydrogenosomes in the diplomonad *Spironucleus salmonicida*. Nat Commun.

[50] Nývltová E, Stairs CW, Hrdý I, Rídl J, Mach J, Pačes J, Roger AJ, Tachezy J (2015). Lateral gene transfer and gene duplication played a key role in the evolution of *Mastigamoeba balamuthi* hydrogenosomes. Mol Biol Evol.

[51] Leger MM, Kolisko M, Kamikawa R, Stairs CW, Kume K, Čepička I, Silberman JD, Andersson JO, Xu F, Yabuki A, Eme L, Zhang Q, Takishita K, Inagaki Y, Simpson AGB, Hashimoto T, Roger AJ (2017). Organelles that illuminate the origins of *Trichomonas* hydrogenosomes and *Giardia* mitosomes. Nat Ecol Evol.

[52] Sanchez LB, Müller M. Purification and characterization of the acetate forming enzyme, acetyl-CoA synthetase (ADP-forming) from the amitochondriate protist, *Giardia lamblia*. FEBS Lett. 1996;378:240–4. 10.1016/0014-5793(95)01463-2. 10.1016/0014-5793(95)01463-2 8557109

[53] Tielens AGM, van Grinsven KWA, Henze K, van Hellemond JJ, Martin W (2010). Acetate formation in the energy metabolism of parasitic helminths and protists. Int J Parasitol.

[54] LaGier MJ, Tachezy J, Stejskal F, Kutisova K, Keithly JS (2003). Mitochondrial-type iron–sulfur cluster biosynthesis genes (IscS and IscU) in the apicomplexan *Cryptosporidium parvum*. Microbiology..

[55] Putignani L, Tait A, Smith HV, Horner D, Tovar J, Tetley L (2004). Characterization of a mitochondrion-like organelle in *Cryptosporidium parvum*. Parasitology..

[56] MacRae JI, Dixon MW, Dearnley MK, Chua HH, Chambers JM, Kenny S (2013). Mitochondrial metabolism of sexual and asexual blood stages of the malaria parasite *Plasmodium falciparum*. BMC Biol.

[57] Denton H, Roberts CW, Alexander J, Thong kam-wah, Coombs GH. Enzymes of energy metabolism in the bradyzoites and tachyzoites of *Toxoplasma gondii*. FEMS Microbiol Lett 1996;137:103–108. 10.1111/j.1574-6968.1996.tb08090.x. 10.1111/j.1574-6968.1996.tb08090.x 8935663

[58] Sturm A, Mollard V, Cozijnsen A, Goodman CD, McFadden GI (2015). Mitochondrial ATP synthase is dispensable in blood-stage *Plasmodium berghei* rodent malaria but essential in the mosquito phase. P Natl Acad Sci USA.

[59] Stechmann A, Hamblin K, Pérez-Brocal V, Gaston D, Richmond GS, van der Giezen M, Clark CG, Roger AJ (2008). Organelles in *Blastocystis* that blur the distinction between mitochondria and hydrogenosomes. Curr Biol.

[60] Makiuchi T, Nozaki T (2014). Highly divergent mitochondrion-related organelles in anaerobic parasitic protozoa. Biochimie..

[61] Rotterová J, Salomaki E, Pánek T, Bourland W, Žihala D, Táborský P, Edgcomb VP, Beinart RA, Kolísko M, Čepička I (2020). Genomics of new ciliate lineages provides insight into the evolution of obligate anaerobiosis. Curr Biol.

[62] Gawryluk RMR, Stairs CW. Diversity of electron transport chains in anaerobic protists. BBA-Bioenergetics. 1862;2021:148334. 10.1016/j.bbabio.2020.148334. 10.1016/j.bbabio.2020.148334 33159845

[63] Li M, Yang H, Gu J-D (2009). Phylogenetic diversity and axial distribution of microbes in the intestinal tract of the polychaete *Neanthes glandicincta*. Microb Ecol.

[64] Boxma B, de Graaf RM, van der Staay GWM, van Alen TA, Ricard G, Gabaldón T, van Hoek AHAM, Moon-van der Staay SY, Koopman WJH, van Hellemond JJ, Tielens AGM, Friedrich T, Veenhuis M, Huynen MA, Hackstein JHP (2005). An anaerobic mitochondrion that produces hydrogen. Nature..

[65] Gawryluk RMR, Kamikawa R, Stairs CW, Silberman JD, Brown MW, Roger AJ (2016). The earliest stages of mitochondrial adaptation to low oxygen revealed in a novel rhizarian. Curr Biol.

[66] Stairs CW, Eme L, Muñoz-Gómez SA, Cohen A, Dellaire G, Shepherd JN, Fawcett JP, Roger AJ. Microbial eukaryotes have adapted to hypoxia by horizontal acquisitions of a gene involved in rhodoquinone biosynthesis. eLife. 2018;7 10.7554/eLife.34292. 10.7554/eLife.34292 PMC5953543 29697049

[67] Tielens AGM, Rotte C, van Hellemond JJ, Martin W (2002). Mitochondria as we don’t know them. Trends Biochem Sci.

[68] Lonjers ZT, Dickson EL, Chu T-PT, Kreutz JE, Neacsu FA, Anders KR, Shepherd JN (2012). Identification of a new gene required for the biosynthesis of rhodoquinone in *Rhodospirillum rubrum*. J Bacteriol.

[69] Bernert AC, Jacobs EJ, Reinl SR, Choi CCY, Roberts Buceta PM, Culver JC, et al. Recombinant RquA catalyzes the in vivo conversion of ubiquinone to rhodoquinone in *Escherichia coli* and *Saccharomyces cerevisiae*. BBA-Mol Cell Biol L. 1864;2019:1226–34. 10.1016/j.bbalip.2019.05.007. 10.1016/j.bbalip.2019.05.007 PMC6874216 31121262

[70] Del Borrello S, Lautens M, Dolan K, Tan JH, Davie T, Schertzberg MR, et al. Rhodoquinone biosynthesis in *C. elegans* requires precursors generated by the kynurenine pathway. eLife. 2019;8 10.7554/eLife.48165. 10.7554/eLife.48165 PMC6656428 31232688

[71] Lapointe J, Wang Y, Bigras E, Hekimi S (2012). The submitochondrial distribution of ubiquinone affects respiration in long-lived Mclk1+/− mice. J Cell Biol.

[72] Padilla-López S, Jiménez-Hidalgo M, Martín-Montalvo A, Clarke CF, Navas P, Santos-Ocaña C. Genetic evidence for the requirement of the endocytic pathway in the uptake of coenzyme Q6 in *Saccharomyces cerevisiae*. BBA-Biomembranes. 1788;2009:1238–48. 10.1016/j.bbamem.2009.03.018. 10.1016/j.bbamem.2009.03.018 PMC3070215 19345667

[73] Raven JA (2021). Determinants, and implications, of the shape and size of thylakoids and cristae. J Plant Physiol.

[74] Mühleip A, Kock Flygaard R, Ovciarikova J, Lacombe A, Fernandes P, Sheiner L, Amunts A (2021). ATP synthase hexamer assemblies shape cristae of *Toxoplasma* mitochondria. Nat Commun.

[75] Salunke R, Mourier T, Banerjee M, Pain A, Shanmugam D (2018). Highly diverged novel subunit composition of apicomplexan F-type ATP synthase identified from *Toxoplasma gondii*. Plos Biol.

[76] Kuvardina ON, Simdyanov TG (2002). Fine structure of syzygy in *Selenidium pennatum* (Sporozoa, Archigregarinida). Protistology..

[77] Valigurová A, Vaškovicová N, Diakin A, Paskerova GG, Simdyanov TG, Kováčiková M (2017). Motility in blastogregarines (Apicomplexa): native and drug-induced organisation of *Siedleckia nematoides* cytoskeletal elements. Plos One.

[78] Desportes I, Schrével J (2013). Treatise on zoology - anatomy, taxonomy, biology. The gregarines (2 vols): the early branching Apicomplexa.

[79] Toso MA, Omoto CK. *Gregarina niphandrodes* may lack both a plastid genome and organelle. J Eukaryot Microbiol. 2007;54:66–72. 10.1111/j.1550-7408.2006.00229.x. 10.1111/j.1550-7408.2006.00229.x 17300522

[80] Tronchin G, Schrevel J. Chronologie des modifications ultrastructurales au cours de la croissance de *Gregarina blaberae*. J Protozool. 1977;24:67–82. 10.1111/j.1550-7408.1977.tb05282.x. 10.1111/j.1550-7408.1977.tb05282.x 405485

[81] Landers SC (2002). The fine structure of the gamont of *Pterospora floridiensi*s (Apicomplexa: Eugregarinida). J Eukaryot Microbiol.

[82] Tovar J, León-Avila G, Sánchez LB, Sutak R, Tachezy J, van der Giezen M, Hernández M, Müller M, Lucocq JM (2003). Mitochondrial remnant organelles of *Giardia* function in iron-sulphur protein maturation. Nature..

[83] Karnkowska A, Vacek V, Zubáčová Z, Treitli SC, Petrželková R, Eme L, Novák L, Žárský V, Barlow LD, Herman EK, Soukal P, Hroudová M, Doležal P, Stairs CW, Roger AJ, Eliáš M, Dacks JB, Vlček Č, Hampl V (2016). A eukaryote without a mitochondrial organelle. Curr Biol.

[84] Salomaki ED, Kolisko M (2019). There is treasure everywhere: reductive plastid evolution in Apicomplexa in light of their close relatives. Biomolecules..

[85] Stairs CW, Eme L, Brown MW, Mutsaers C, Susko E, Dellaire G, Soanes DM, van der Giezen M, Roger AJ (2014). A SUF Fe-S cluster biogenesis system in the mitochondrion-related organelles of the anaerobic protist *Pygsuia*. Curr Biol.

[86] Mi-ichi F, Yousuf MA, Nakada-Tsukui K, Nozaki T (2009). Mitosomes in *Entamoeba histolytica* contain a sulfate activation pathway. P Nat Acad Sci USA..

[87] Maralikova B, Ali V, Nakada-Tsukui K, Nozaki T, van der Giezen M, Henze K, Tovar J (2010). Bacterial-type oxygen detoxification and iron-sulfur cluster assembly in amoebal relict mitochondria. Cell Microbiol.

[88] Nyvltova E, Sutak R, Harant K, Sedinova M, Hrdy I, Paces J, Vlcek C, Tachezy J (2013). NIF-type iron-sulfur cluster assembly system is duplicated and distributed in the mitochondria and cytosol of Mastigamoeba balamuthi. P Nat Acad Sci USA..

[89] Babady NE, Pang Y-P, Elpeleg O, Isaya G (2007). Cryptic proteolytic activity of dihydrolipoamide dehydrogenase. P Nat Acad Sci USA.

[90] Husnik F, Nikoh N, Koga R, Ross L, Duncan RP, Fujie M, Tanaka M, Satoh N, Bachtrog D, Wilson ACC, von Dohlen CD, Fukatsu T, McCutcheon JP (2013). Horizontal gene transfer from diverse bacteria to an insect genome enables a tripartite nested mealybug symbiosis. Cell..

[91] Leger MM, Eme L, Hug LA, Roger AJ. Novel hydrogenosomes in the microaerophilic Jakobid *Stygiella incarcerata*. Mol Biol Evol. 2016;33(9):2318–36. 10.1111/j.1550-7408.2002.tb00526.x. 10.1093/molbev/msw103 PMC4989108 27280585

[92] Van Etten J, Bhattacharya D (2020). Horizontal gene transfer in eukaryotes: not if, but how much?. Trends Genet.

[93] Nosenko T, Bhattacharya D (2007). Horizontal gene transfer in chromalveolates. BMC Evol Biol.

[94] van der Rest ME, Frank C, Molenaar D (2000). Functions of the membrane-associated and cytoplasmic malate dehydrogenases in the citric acid cycle of *Escherichia coli*. J Bacteriol.

[95] Molenaar D, van der Rest ME, Drysch A, Yücel R (2000). Functions of the membrane-associated and cytoplasmic malate dehydrogenases in the citric acid cycle of *Corynebacterium glutamicum*. J Bacteriol.

[96] Adl SM, Bass D, Lane CE, Lukeš J, Schoch CL, Smirnov A, et al. Revisions to the Classification, Nomenclature, and Diversity of Eukaryotes. J Eukaryot Microbiol. 2019;66:4–119. 10.1111/jeu.12691 PMC6492006 30257078

[97] Rotte C, Stejskal F, Zhu G, Keithly JS, Martin W (2001). Pyruvate:NADP oxidoreductase from the mitochondrion of *Euglena gracilis* and from the apicomplexan *Cryptosporidium parvum*: a biochemical relic linking pyruvate metabolism in mitochondriate and amitochondriate protists. Mol Biol Evol.

[98] Picelli S, Faridani OR, Björklund ÅK, Winberg G, Sagasser S, Sandberg R (2014). Full-length RNA-seq from single cells using Smart-seq2. Nat Protoc.

[99] Bolger AM, Lohse M, Usadel B (2014). Trimmomatic: a flexible trimmer for Illumina sequence data. Bioinformatics..

[100] Bankevich A, Nurk S, Antipov D, Gurevich AA, Dvorkin M, Kulikov AS, Lesin VM, Nikolenko SI, Pham S, Prjibelski AD, Pyshkin AV, Sirotkin AV, Vyahhi N, Tesler G, Alekseyev MA, Pevzner PA (2012). SPAdes: a new genome assembly algorithm and its applications to single-cell sequencing. J Comput Biol.

[101] Simão FA, Waterhouse RM, Ioannidis P, Kriventseva EV, Zdobnov EM (2015). BUSCO: assessing genome assembly and annotation completeness with single-copy orthologs. Bioinformatics..

[102] Aurrecoechea C, Barreto A, Basenko EY, Brestelli J, Brunk BP, Cade S, Crouch K, Doherty R, Falke D, Fischer S, Gajria B, Harb OS, Heiges M, Hertz-Fowler C, Hu S, Iodice J, Kissinger JC, Lawrence C, Li W, Pinney DF, Pulman JA, Roos DS, Shanmugasundram A, Silva-Franco F, Steinbiss S, Stoeckert CJ, Spruill D, Wang H, Warrenfeltz S, Zheng J (2017). EuPathDB: the eukaryotic pathogen genomics database resource. Nucleic Acids Res.

[103] Multiple independent origins of apicomplexan-like parasites. NCBI Bioproject PRJNA539986. 2019. https://www.ncbi.nlm.nih.gov/bioproject/PRJNA539986/. Accessed 3 Jan 2020.

[104] Janouškovec J, Paskerova GG, Miroliubova TS, Mikhailov KV, Birley T, Aleoshin VV, et al. Transcriptomes of apicomplexan parasites. NCBI Bioproject PRJNA557242. 2019. https://www.ncbi.nlm.nih.gov/bioproject/PRJNA557242/. Accessed 3 Jan 2020.

[105] Sequencing of a metagenome and metatranscriptome from a Nephromyces-enriched renal sac of *Molgula occidentalis*. NCBI Bioproject PRJNA524113. 2019. https://www.ncbi.nlm.nih.gov/bioproject/PRJNA524113/. . Accessed 3 Jan 2020.

[106] Keeling PJ, Burki F, Wilcox HM, Allam B, Allen EE, Amaral-Zettler LA, Armbrust EV, Archibald JM, Bharti AK, Bell CJ, Beszteri B, Bidle KD, Cameron CT, Campbell L, Caron DA, Cattolico RA, Collier JL, Coyne K, Davy SK, Deschamps P, Dyhrman ST, Edvardsen B, Gates RD, Gobler CJ, Greenwood SJ, Guida SM, Jacobi JL, Jakobsen KS, James ER, Jenkins B, John U, Johnson MD, Juhl AR, Kamp A, Katz LA, Kiene R, Kudryavtsev A, Leander BS, Lin S, Lovejoy C, Lynn D, Marchetti A, McManus G, Nedelcu AM, Menden-Deuer S, Miceli C, Mock T, Montresor M, Moran MA, Murray S, Nadathur G, Nagai S, Ngam PB, Palenik B, Pawlowski J, Petroni G, Piganeau G, Posewitz MC, Rengefors K, Romano G, Rumpho ME, Rynearson T, Schilling KB, Schroeder DC, Simpson AGB, Slamovits CH, Smith DR, Smith GJ, Smith SR, Sosik HM, Stief P, Theriot E, Twary SN, Umale PE, Vaulot D, Wawrik B, Wheeler GL, Wilson WH, Xu Y, Zingone A, Worden AZ (2014). The marine microbial eukaryote transcriptome sequencing project (MMETSP): illuminating the functional diversity of eukaryotic life in the oceans through transcriptome sequencing. Plos Biol.

[107] Nenarokov S, Kolisko M. Winston Cleaner. github.com/Seraff/WinstonCleaner. Accessed 8 Aug 2019.

[108] Salomaki ED, Terpis KX, Rueckert S, Kotyk M, Varadínová ZK, Čepička I, et al. Phylogenomic dataset files for: Gregarine single-cell transcriptomics reveals differential mitochondrial remodeling and adaptation in apicomplexans. figshare. 2021. doi: 10.6084/m9.figshare.13344227.v1. 10.1186/s12915-021-01007-2 PMC8051059 33863338

[109] Whelan S, Irisarri I, Burki F (2018). PREQUAL: detecting non-homologous characters in sets of unaligned homologous sequences. Bioinformatics..

[110] Katoh K, Standley DM (2013). MAFFT multiple sequence alignment software version 7: improvements in performance and usability. Mol Biol Evol.

[111] Ali RH, Bogusz M, Whelan S (2019). Identifying clusters of high confidence homologies in multiple sequence alignments. Mol Biol Evol.

[112] Capella-Gutierrez S, Silla-Martinez JM, Gabaldon T (2009). trimAl: a tool for automated alignment trimming in large-scale phylogenetic analyses. Bioinformatics.

[113] Stamatakis A (2014). RAxML version 8: a tool for phylogenetic analysis and post-analysis of large phylogenies. Bioinformatics..

[114] Criscuolo A, Gribaldo S (2010). BMGE (Block Mapping and Gathering with Entropy): a new software for selection of phylogenetic informative regions from multiple sequence alignments. BMC Evol Biol.

[115] Lanfear R, Frandsen PB, Wright AM, Senfeld T, Calcott B. PartitionFinder 2: new methods for selecting partitioned models of evolution for molecular and morphological phylogenetic analyses. Mol Biol Evol. 2016;34:772–3. 10.1093/molbev/msw260. 10.1093/molbev/msw260 28013191

[116] Nguyen L-T, Schmidt HA, von Haeseler A, Minh BQ. IQ-TREE: a fast and effective stochastic algorithm for estimating maximum-likelihood phylogenies. Mol Biol Evol. 2015;32:268–74. 10.1093/molbev/msu300. 10.1093/molbev/msu300 PMC4271533 25371430

[117] Lartillot N, Rodrigue N, Stubbs D, Richer J. PhyloBayes MPI: phylogenetic reconstruction with infinite mixtures of profiles in a parallel environment. Syst Biol 2013;62:611–615, 4, doi: 10.1093/sysbio/syt022. 10.1093/sysbio/syt022 23564032

[118] Salomaki ED, Terpis KX, Rueckert S, Kotyk M, Varadínová ZK, Čepička I, et al. Phylogenomic log files for: Gregarine single-cell transcriptomics reveals differential mitochondrial remodeling and adaptation in apicomplexans. figshare. 2021. doi: 10.6084/m9.figshare.13927292.v2. 10.1186/s12915-021-01007-2 PMC8051059 33863338

[119] Shen X-X, Li Y, Hittinger CT, Chen X, Rokas A (2020). An investigation of irreproducibility in maximum likelihood phylogenetic inference. Nat Commun.

[120] Tice AK, Žihala D, Pánek T, Jones R, Salomaki ED, Nenarokov S, et al. PhyloFisher: a phylogenomic package for resolving deep eukaryotic relationships https://github.com/TheBrownLab/PhyloFisher. Accessed 15 Sept 2019. 10.1371/journal.pbio.3001365 PMC8345874 34358228

[121] Shimodaira H (2002). An approximately unbiased test of phylogenetic tree selection. Syst Biol.

[122] Smith DGS, Gawryluk RMR, Spencer DF, Pearlman RE, Siu KWM, Gray MW (2007). Exploring the mitochondrial proteome of the ciliate protozoon *Tetrahymena thermophila*: direct analysis by tandem mass spectrometry. J Mol Biol.

[123] Leger MM, Kolisko M, Kamikawa R, Stairs CW, Kume K, Čepička I, et al. Data from: Organelles that illuminate the origins of Trichomonas hydrogenosomes and Giardia mitosomes. Dryad. 2018; 10.5061/dryad.34qd7. 10.1038/s41559-017-0092 PMC5411260 28474007

[124] Seidi A, Muellner-Wong LS, Rajendran E, Tjhin ET, Dagley LF, Aw VY, et al. Elucidating the mitochondrial proteome of *Toxoplasma gondii* reveals the presence of a divergent cytochrome c oxidase. eLife. 2018;7 10.7554/eLife.38131. 10.7554/eLife.38131 PMC6156079 30204084

[125] Rotterová J, Salomaki ED, Pánek T, Bourland W, Žihala D, Táborský P, et al. Genomics of new ciliate lineages provides insight into the evolution of obligate anaerobiosis - single gene datasets for phylogenomic analysis of anaerobic ciliates (SAL, Ciliophora), protein datasets for mitochondrial pathways prediction, and mitochondrial genomes. Dryad. 2020; 10.5061/dryad.vx0k6djnm.

[126] Li W, Godzik A (2006). Cd-hit: a fast program for clustering and comparing large sets of protein or nucleotide sequences. Bioinformatics..

[127] Almagro Armenteros JJ, Salvatore M, Emanuelsson O, Winther O, von Heijne G, Elofsson A, Nielsen H (2019). Detecting sequence signals in targeting peptides using deep learning. Life Sci Alliance.

[128] Almagro Armenteros JJ, Sønderby CK, Sønderby SK, Nielsen H, Winther O (2017). DeepLoc: prediction of protein subcellular localization using deep learning. Bioinformatics..

[129] Salomaki ED, Terpis KX, Rueckert S, Kotyk M, Varadínová ZK, Čepička I, et al. Mitochondrial gene dataset files for: Gregarine single-cell transcriptomics reveals differential mitochondrial remodeling and adaptation in apicomplexans. figshare. 2021. doi: 10.6084/m9.figshare.13928252.v1. 10.1186/s12915-021-01007-2 PMC8051059 33863338

