# Supplementary figures and images for: Gregarine single-cell transcriptomics reveals differential mitochondrial remodeling and adaptation in apicomplexans
Source: BMC Biol. 2021 Apr 16;19:77. doi: 10.1186/s12915-021-01007-2 (PMC8051059; doi:10.1186/s12915-021-01007-2)

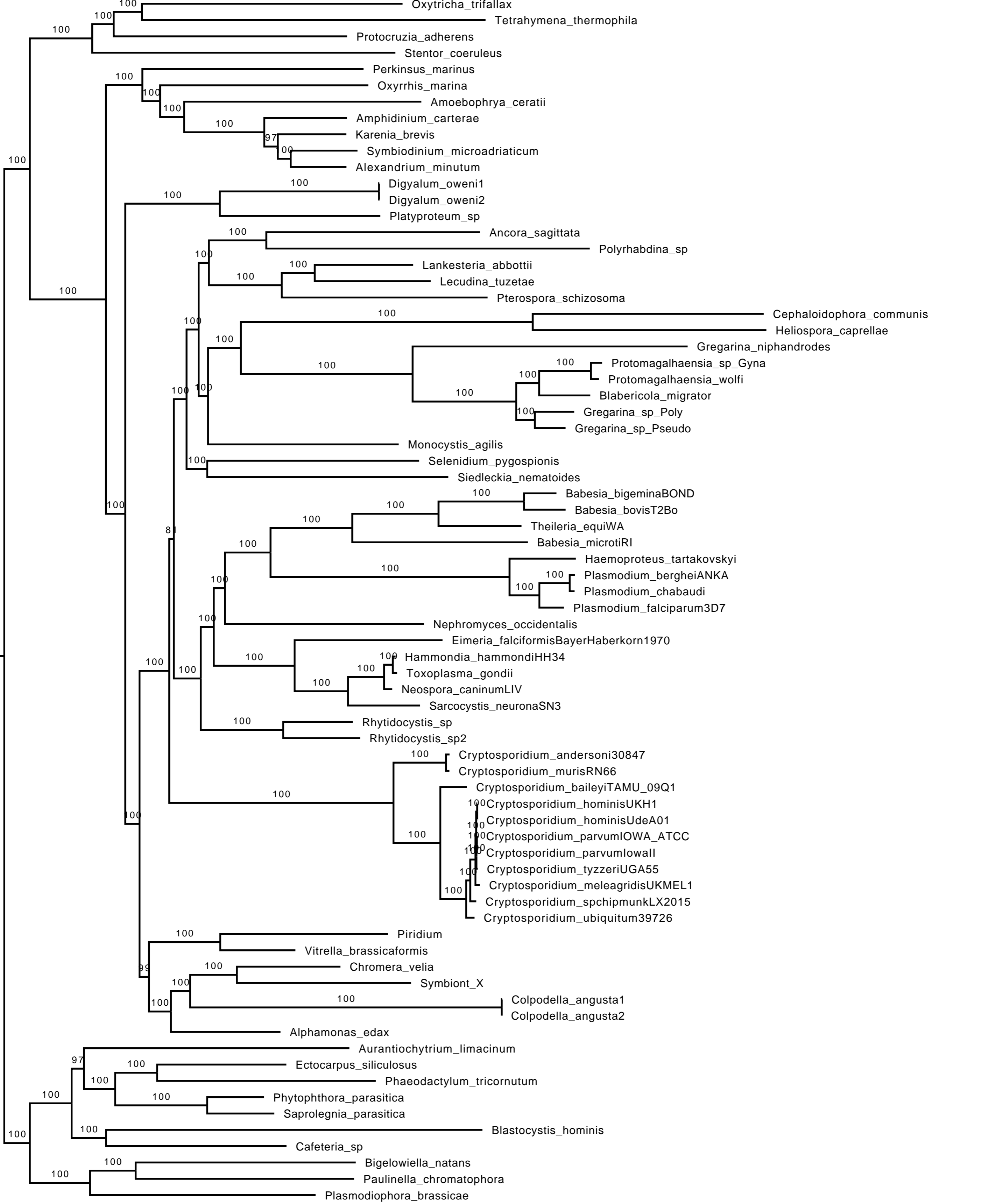

0.2

Supplement: Supplementary file 2 — Additional file 2: Fig. S1. Maximum likelihood phylogeny of apicomplexans as recovered from dataset A, comprised of 246 genes and 63,201 sites. The final dataset was partitioned under the LG+G model in PartitionFinder2 and then subjected to maximum likelihood analysis implemented in IQtree (LG+C60+F+G). Non-parametric PMSF bootstrap support values (n = 1000) are shown on the branches. [file 12915_2021_1007_MOESM2_ESM.pdf]

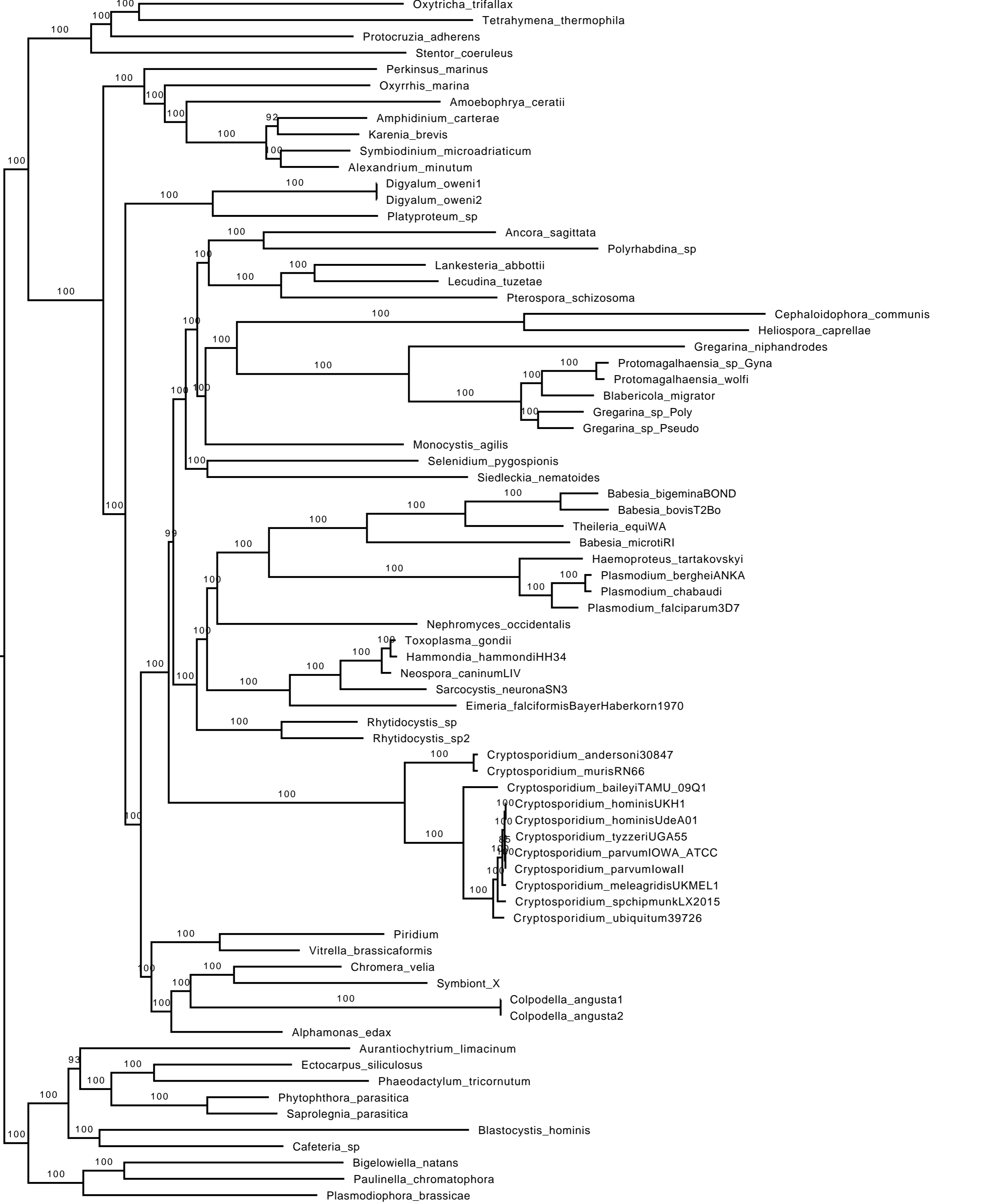

0.2

Supplement: Supplementary file 3 — Additional file 3: Fig. S2. Maximum likelihood phylogeny of apicomplexans as recovered from dataset B, comprised of 299 genes and 89,675 sites. The final dataset was partitioned under the LG+G model in PartitionFinder2 and then subjected to maximum likelihood analysis implemented in IQtree (LG+C60+F+G). Non-parametric PMSF bootstrap support values (n = 1000) are shown on the branches. [file 12915_2021_1007_MOESM3_ESM.pdf]

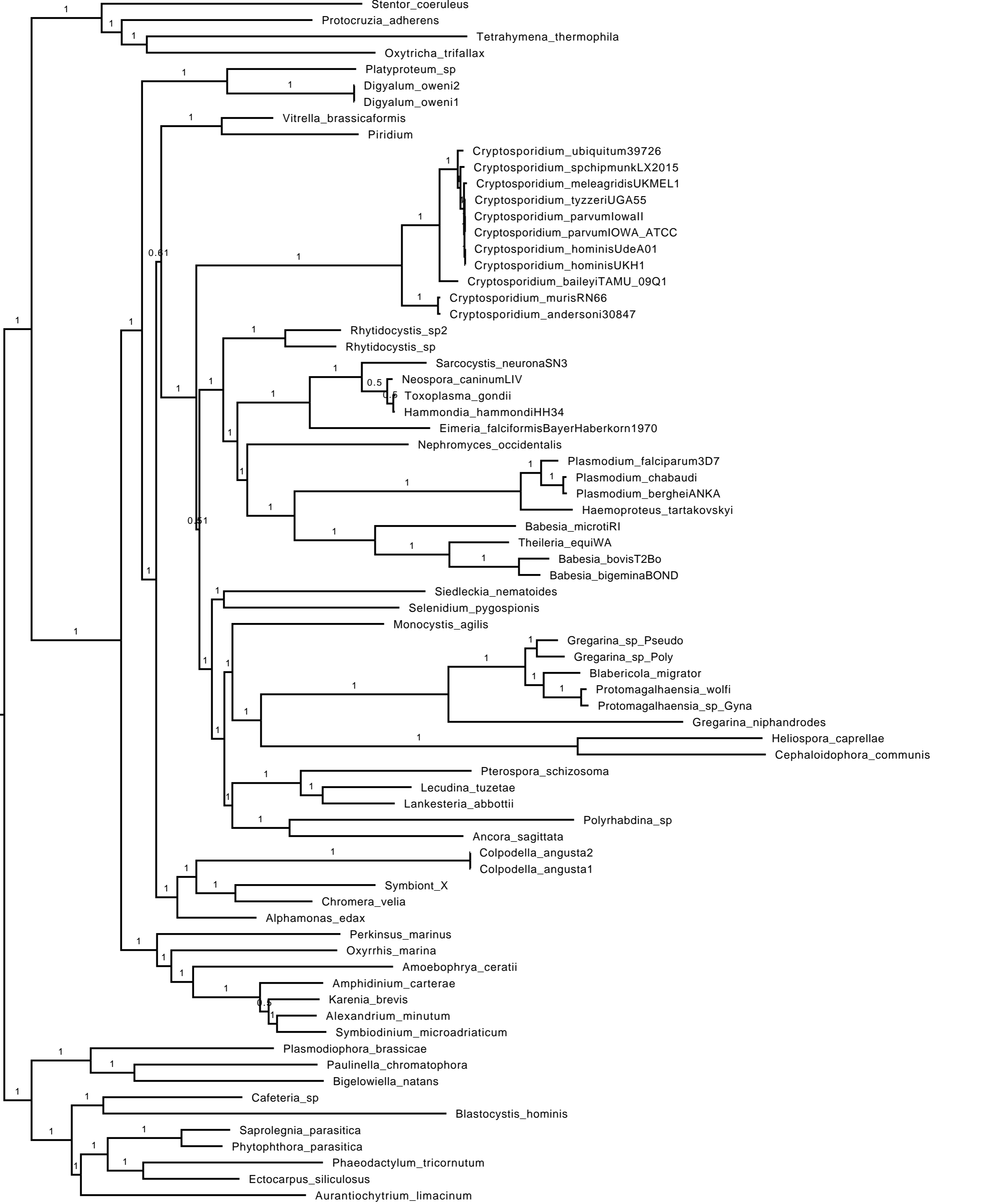

0.3

Supplement: Supplementary file 4 — Additional file 4: Fig. S3. Bayesian inference (BI) consensus tree for apicomplexans as recovered from dataset A, comprised of 246 genes and 63,201 sites. The final dataset was subjected to two chains of BI in Phylobayes for 7000 generations with every second generation sampled and a burnin of 20%. The two chains for dataset A did not converge (maxdiff = 1) and their topologies are independently shown as Figs. S7 and S8. Support values listed on branches are posterior probabilities from the analysis. [file 12915_2021_1007_MOESM4_ESM.pdf]

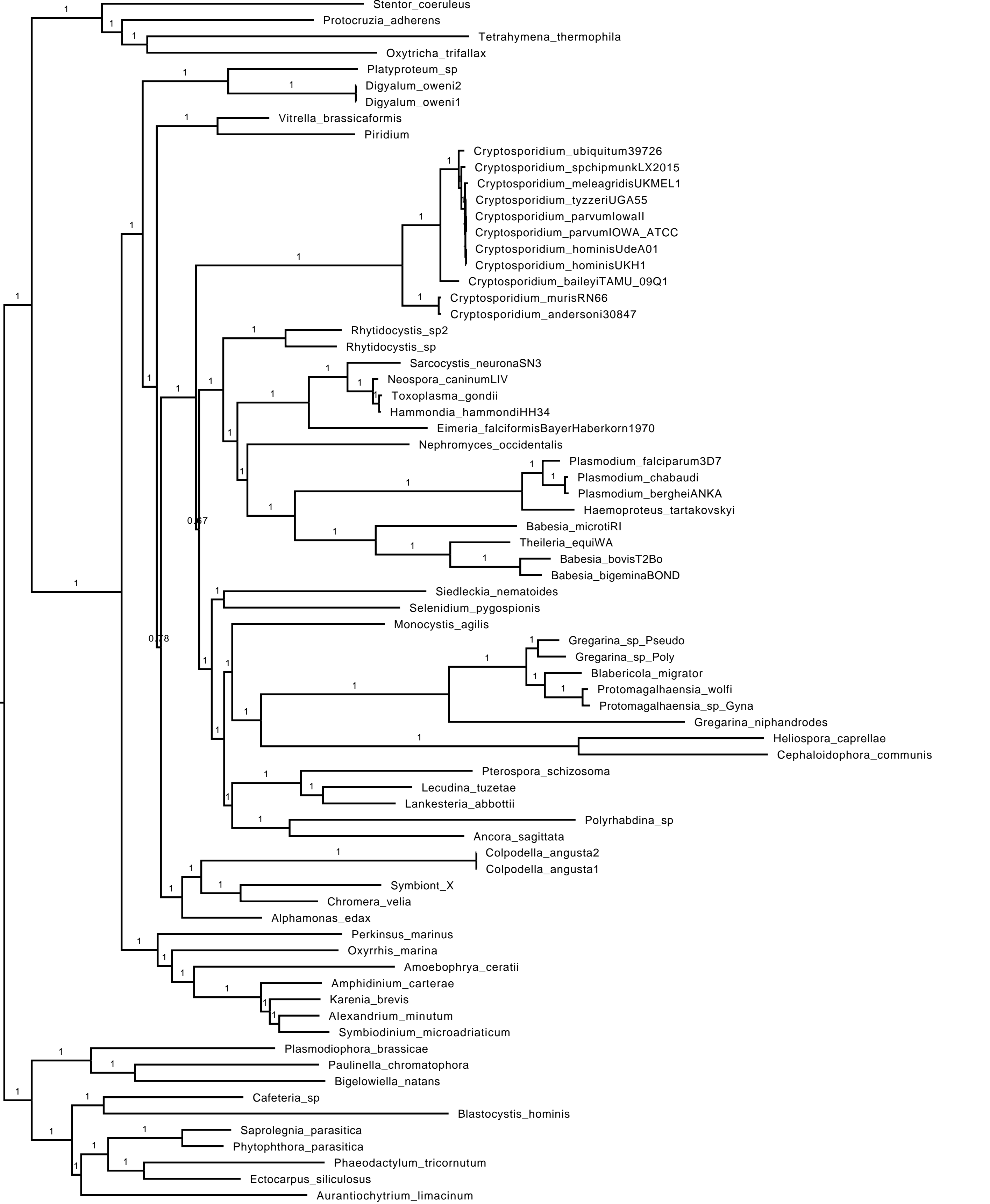

0.3

Supplement: Supplementary file 5 — Additional file 5: Fig. S4. Bayesian inference (BI) tree for chain 1 from dataset A, comprised of 246 genes and 63,201 sites. The final dataset was subjected to two chains of BI in Phylobayes for 7000 generations with every second generation sampled and a burnin of 20%. Support values listed on branches are posterior probabilities from the analysis. [file 12915_2021_1007_MOESM5_ESM.pdf]

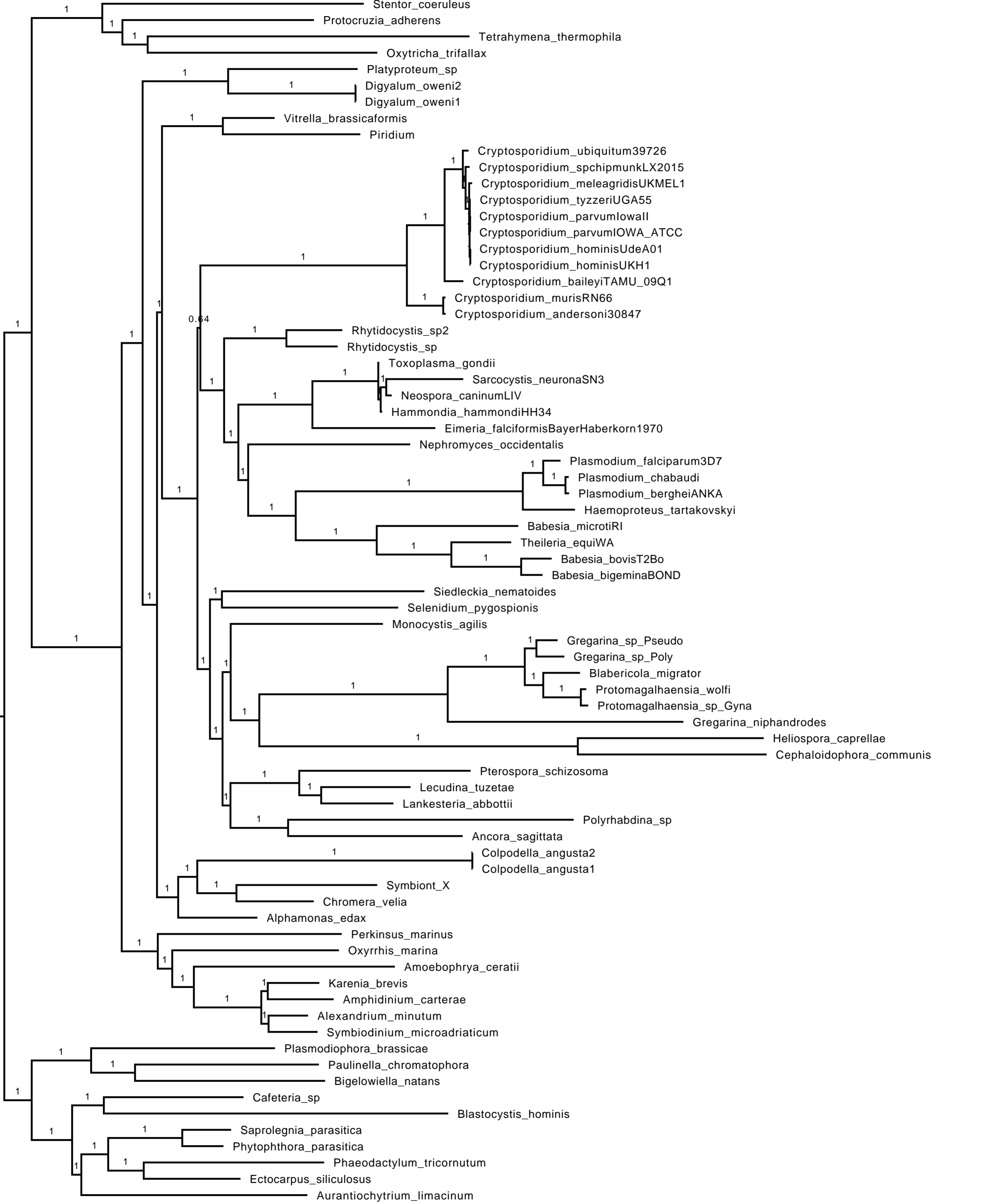

0.3

Supplement: Supplementary file 6 — Additional file 6: Fig. S5. Bayesian inference (BI) tree for chain 2 from dataset A, comprised of 246 genes and 63,201 sites. The final dataset was subjected to two chains of BI in Phylobayes for 7000 generations with every second generation sampled and a burnin of 20%. Support values listed on branches are posterior probabilities from the analysis. [file 12915_2021_1007_MOESM6_ESM.pdf]

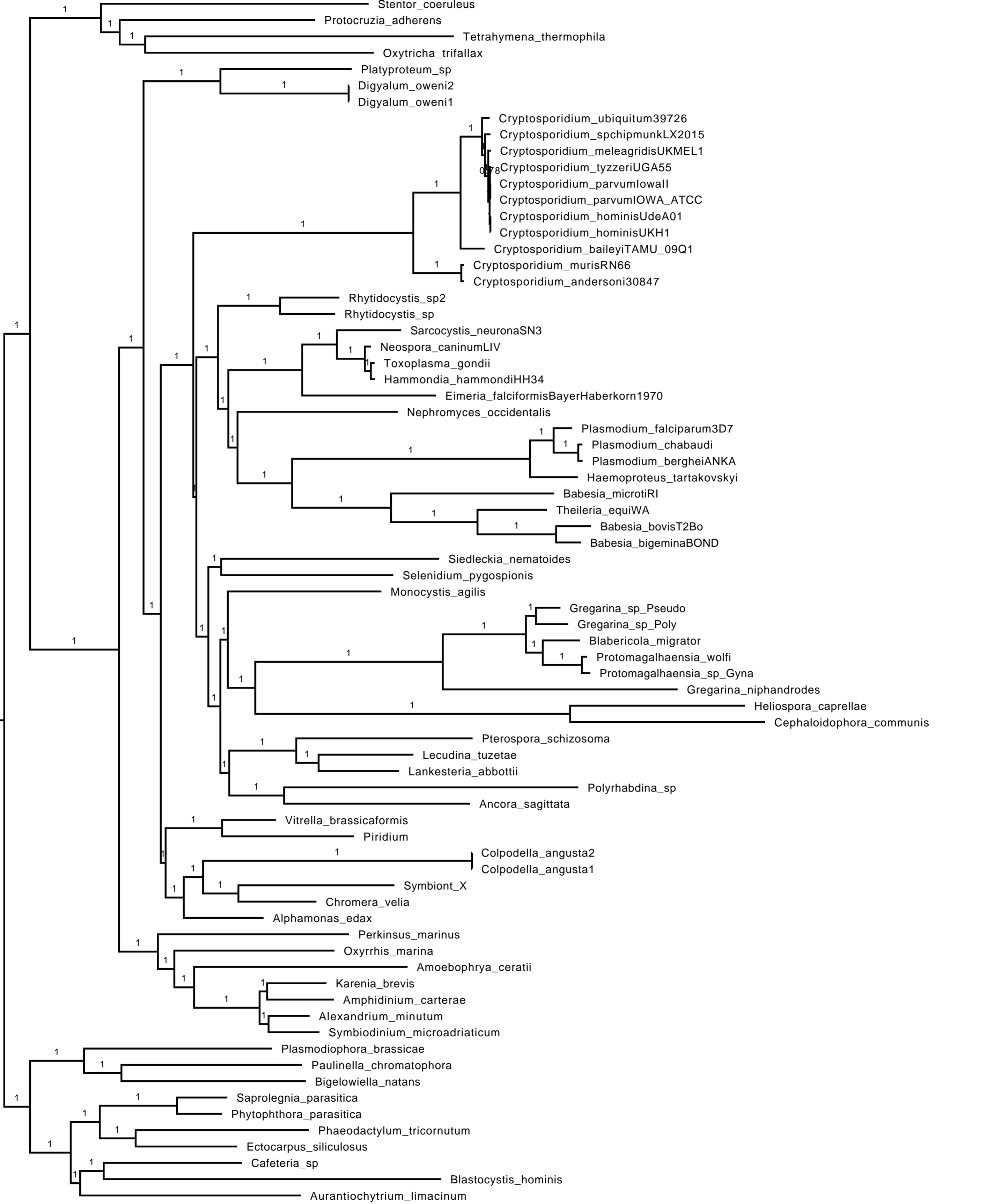

0.3

Supplement: Supplementary file 7 — Additional file 7: Fig. S6. Bayesian inference (BI) consensus tree for apicomplexans as recovered from dataset B, comprised of 299 genes and 89,675 sites. The final dataset was subjected to two chains of BI in Phylobayes for 7000 generations with every second generation sampled and a burnin of 20%. The two chains for dataset B converged with maxdiff = 0.081. Support values listed on branches are posterior probabilities from the analysis. [file 12915_2021_1007_MOESM7_ESM.pdf]

Dataset A Unique Genes

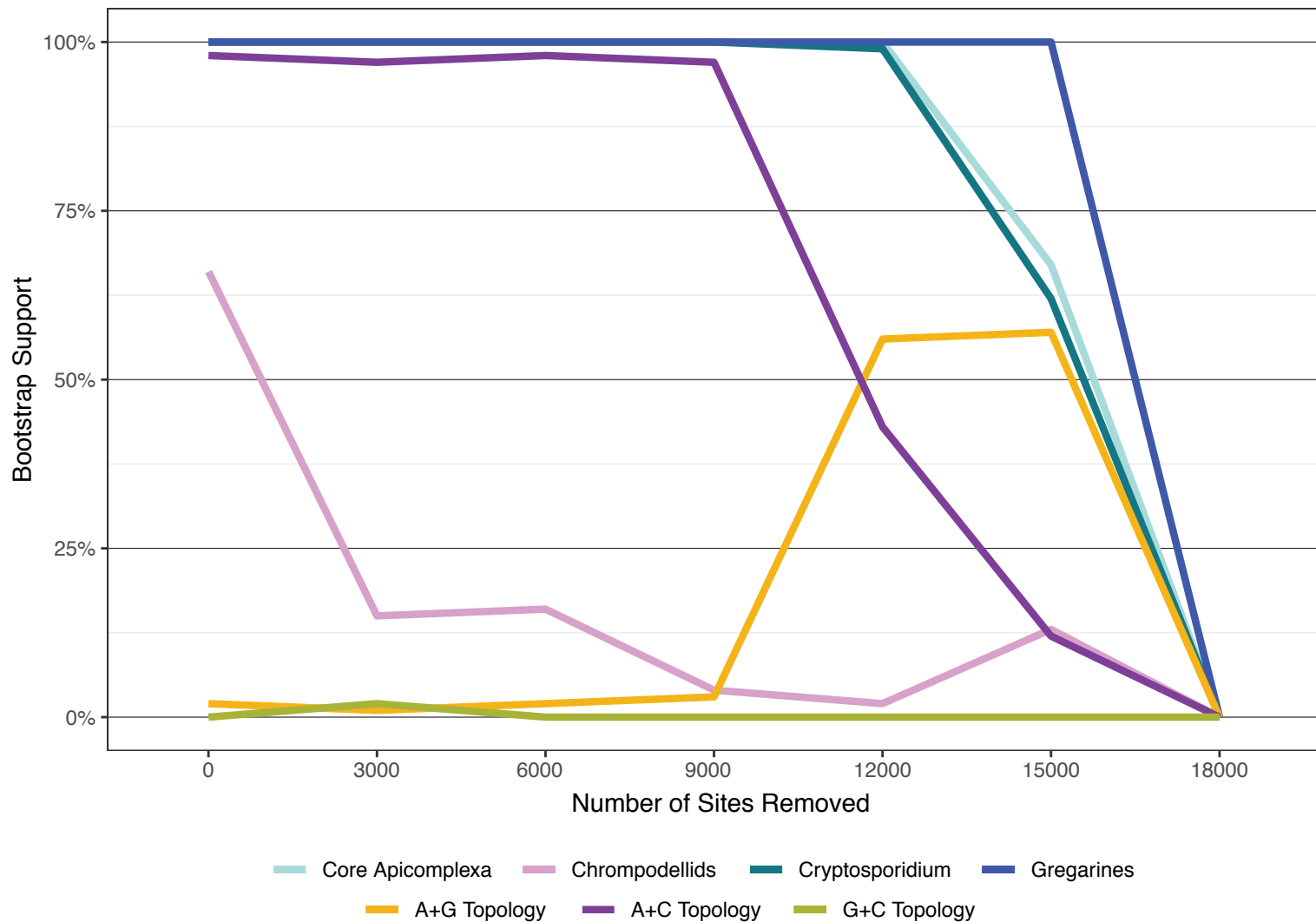

Supplement: Supplementary file 12 — Additional file 12: Fig. S7. Effects of fast evolving site removal on support for bipartitions of interest in the phylogenomic analyses of genes that are unique to dataset A. Graphs plotting support for bipartitions of interest after the stepwise removal of the 3000 fastest evolving sites until all sites are removed from each dataset (dataset A on the top and dataset B on the bottom). 100 RAxML rapid bootstraps values (PROTCATLGF) are on the y-axis and number of sites removed, measured in thousands, is shown on the x-axis. [file 12915_2021_1007_MOESM12_ESM.pdf]

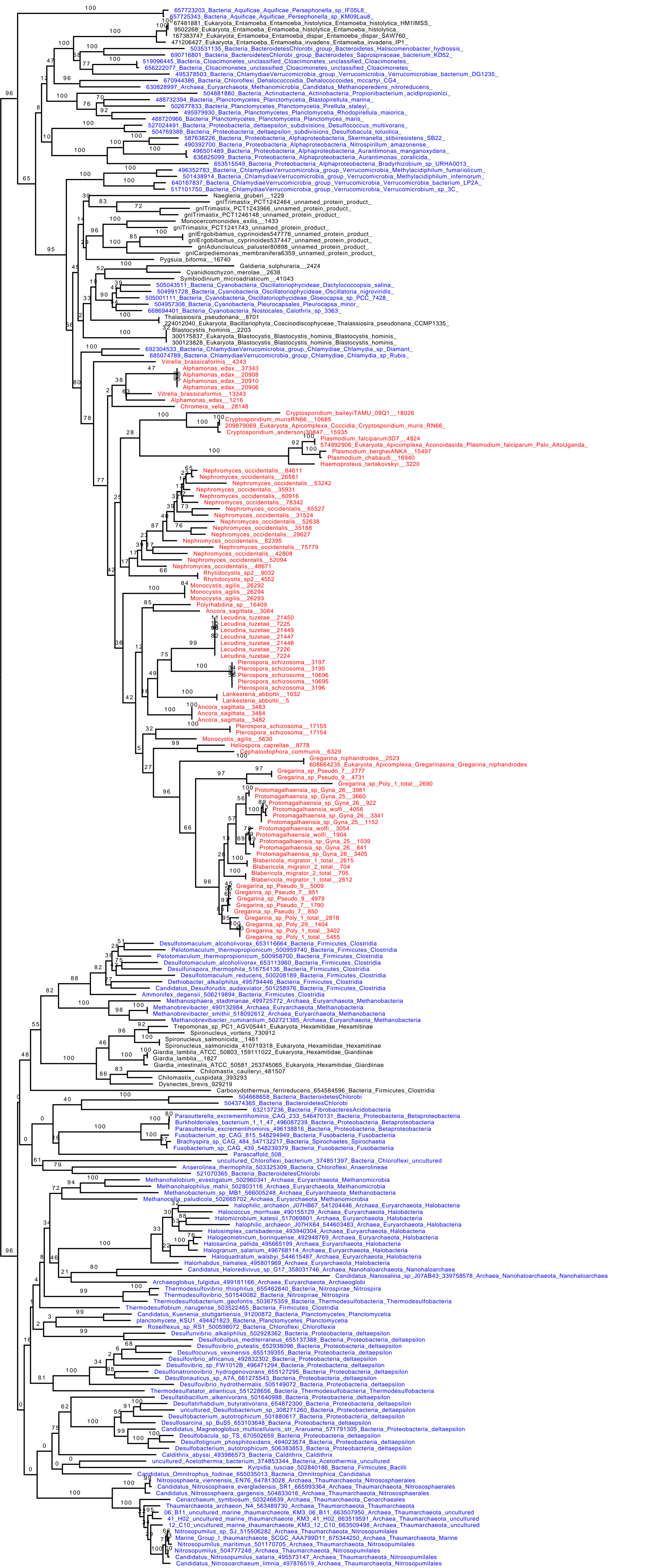

Supplement: Supplementary file 17 — Additional file 17: Fig. S11. Maximum likelihood phylogeny of ADP-forming ACS reconstructed in IQtree (LG+C20+F+G) with support values from 100 non-parametric PMSF bootstrap replicates. Prokaryotes are blue, Apicomplexa, Squirmidea and chrompodellids are colored red, and other eukaryotes are shown in black. The topology shows moderately strong support for a laterally transferred ADP-forming ACS gene of prokaryotic origin to the shared ancestor of chrompodellids and apicomplexans. [file 12915_2021_1007_MOESM17_ESM.pdf]

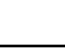

Supplement: Supplementary file 18 — Additional file 18: Fig. S12. Maximum likelihood phylogeny of malic enzyme reconstructed in IQtree (LG+C20+F+G) with support values from 100 non-parametric PMSF bootstrap replicates. Prokaryotes are blue, Apicomplexa, Squirmidea and chrompodellids are colored red, and other eukaryotes are shown in black. The topology shows strong support for an independent acquisition of malic enzyme by members of the Gregarinoidea through lateral gene transfer from proteobacteria. The malic enzyme sequenced in other eugregarines branches with members of the core apicomplexans, chrompodellids, and Squirmidea. [file 12915_2021_1007_MOESM18_ESM.pdf]
