# Supplementary material for: Gregarine single-cell transcriptomics reveals differential mitochondrial remodeling and adaptation in apicomplexans
Source: BMC Biol. 2021 Apr 16;19:77. doi: 10.1186/s12915-021-01007-2 (PMC8051059; doi:10.1186/s12915-021-01007-2)
Supplement: Supplementary file 8 — Additional file 8: Table S2. Gene concordance factors for possible apicomplexan relationships. [file 12915_2021_1007_MOESM8_ESM.docx]

Table S2. Gene concordance factors for based on all individual genes and our partitioning scheme for maximum likelihood trees (IQtree LG+C60+F+G) for the three possible relationships of apicomplexan lineages, where A+G is monophyly of core apicomplexans and gregarines, A+C is monophyly of core apicomplexans and *Cryptosporidium*, and G+C is monophyly of gregarines and *Cryptosporidium*.

|  | A+G | A+C | G+C |
| --- | --- | --- | --- |
| Dataset A All Genes | 6.19% | 4.29% | 0.952% |
| Dataset A Partitions | 15.5% | 13.8% | 6.9% |
| Dataset B All Genes | 6.32% | 5.5% | 0.743% |
| Dataset B Partitions | 18.6% | 7.14% | 12.9% |
