# Supplementary material for: Gregarine single-cell transcriptomics reveals differential mitochondrial remodeling and adaptation in apicomplexans
Source: BMC Biol. 2021 Apr 16;19:77. doi: 10.1186/s12915-021-01007-2 (PMC8051059; doi:10.1186/s12915-021-01007-2)
Supplement: Supplementary file 9 — Additional file 9: Table S3. Approximately unbiased test scores for possible apicomplexan relationships. [file 12915_2021_1007_MOESM9_ESM.docx]

Table S3. P-value scores for the approximately unbiased (AU) test for maximum likelihood trees (IQtree LG+C60+F+G) for the three possible relationships of apicomplexan lineages, where A+G is monophyly of core apicomplexans and gregarines, A+C is monophyly of core apicomplexans and *Cryptosporidium*, and G+C is monophyly of gregarines and *Cryptosporidium*. The ‘+’ indicated the topology was not rejected by the dataset and a ‘-’ indicates the topology was rejected.

|  | A+G | A+C | G+C |
| --- | --- | --- | --- |
| Dataset A | 0.599 + | 0.641 + | 0.0313 - |
| Dataset B | 0.939 + | 0.208 + | 0.172 + |
