# Supplementary material for: Gregarine single-cell transcriptomics reveals differential mitochondrial remodeling and adaptation in apicomplexans
Source: BMC Biol. 2021 Apr 16;19:77. doi: 10.1186/s12915-021-01007-2 (PMC8051059; doi:10.1186/s12915-021-01007-2)
Supplement: Supplementary file 14 — Additional file 14: Fig. S9. Box-and-whisker plots showing the difference in gene likelihood scores (ΔGLS) dataset A and dataset B. ΔGLS values for genes that plot above the upper whisker are shown with the preferred topology of the gene tree (A + C = core apicomplexans with Cryptosporidium; A + G = core apicomplexans with gregarines; G + C = gregarines with Cryptosporidium). [file 12915_2021_1007_MOESM14_ESM.pdf]

Boxplots of Outlier Genes

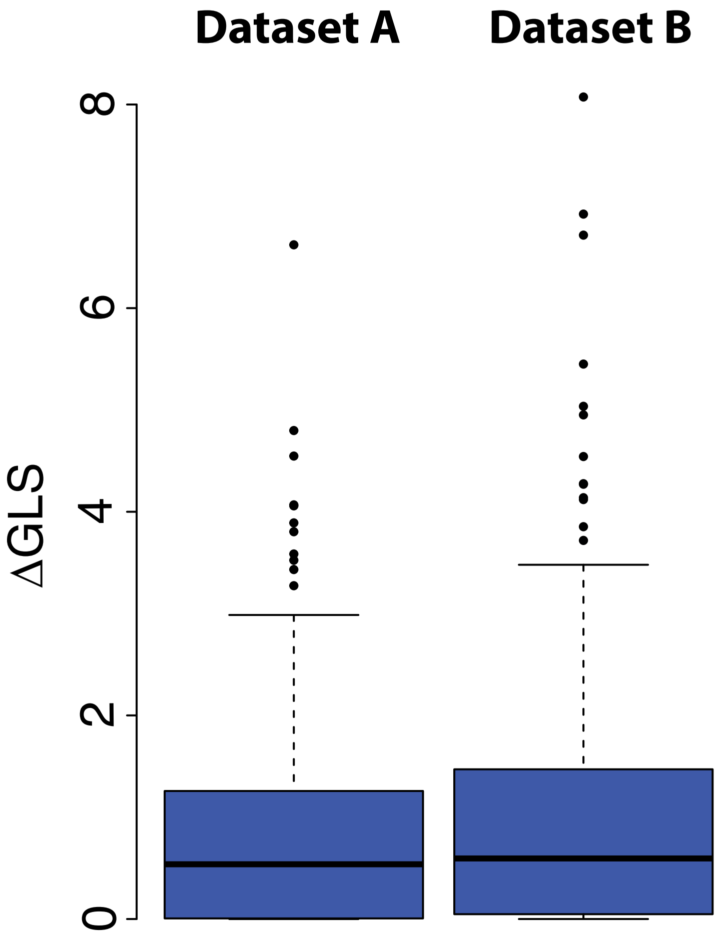

Datasets A Outlier Genes

GENE	ΔGLS	Best Topology
tcp1-gamma	6.62193333	A+C
gpn3	4.79786667	G+C
tcp1-eta	4.54706667	A+C
crfg	4.06766667	G+C
psmc5	4.0582	A+G
tubg	3.89093333	A+C
dimt1l	3.80406667	G+C
tcp1-beta	3.5854	A+G
gdi2	3.52386667	A+C
eif6	3.43253333	G+C
mcm4	3.27373333	A+G
psmd1	2.98626667	G+C

Datasets B Outlier Genes

GENE	ΔGLS	Best Topology
XP_002897020	8.0736	A+C
XP_002904235	6.9236	G+C
XP_002903638	6.717	G+C
XP_002906256	5.4506	G+C
XP_002903063	5.035733333	A+G
XP_0029062	4.951466667	A+C
XP_0029960	4.542533333	G+C
XP_0028992	4.275733333	A+C
XP_0027722	4.270466667	G+C
XP_002895552	4.1382	G+C
XP_0029092	4.119866667	A+G
XP_002997686	3.853266667	A+G
XP_0029053	3.7186	A+G
