# Supplementary material for: Gregarine single-cell transcriptomics reveals differential mitochondrial remodeling and adaptation in apicomplexans
Source: BMC Biol. 2021 Apr 16;19:77. doi: 10.1186/s12915-021-01007-2 (PMC8051059; doi:10.1186/s12915-021-01007-2)
Supplement: Supplementary file 16 — Additional file 16: Fig. S10. Maximum likelihood trees for all mitochondrial genes discussed in this study inferred using IQtree (LG+C20+F+G). Support values are calculated from 100 RAxML rapid bootstraps under the PROTGAMMALG model. Sequences identified as apicomplexan homologs are colored in red, sequences in purple are suspected apicomplexan contamination of a host rather than the target organism. [file 12915_2021_1007_MOESM16_ESM.pdf]

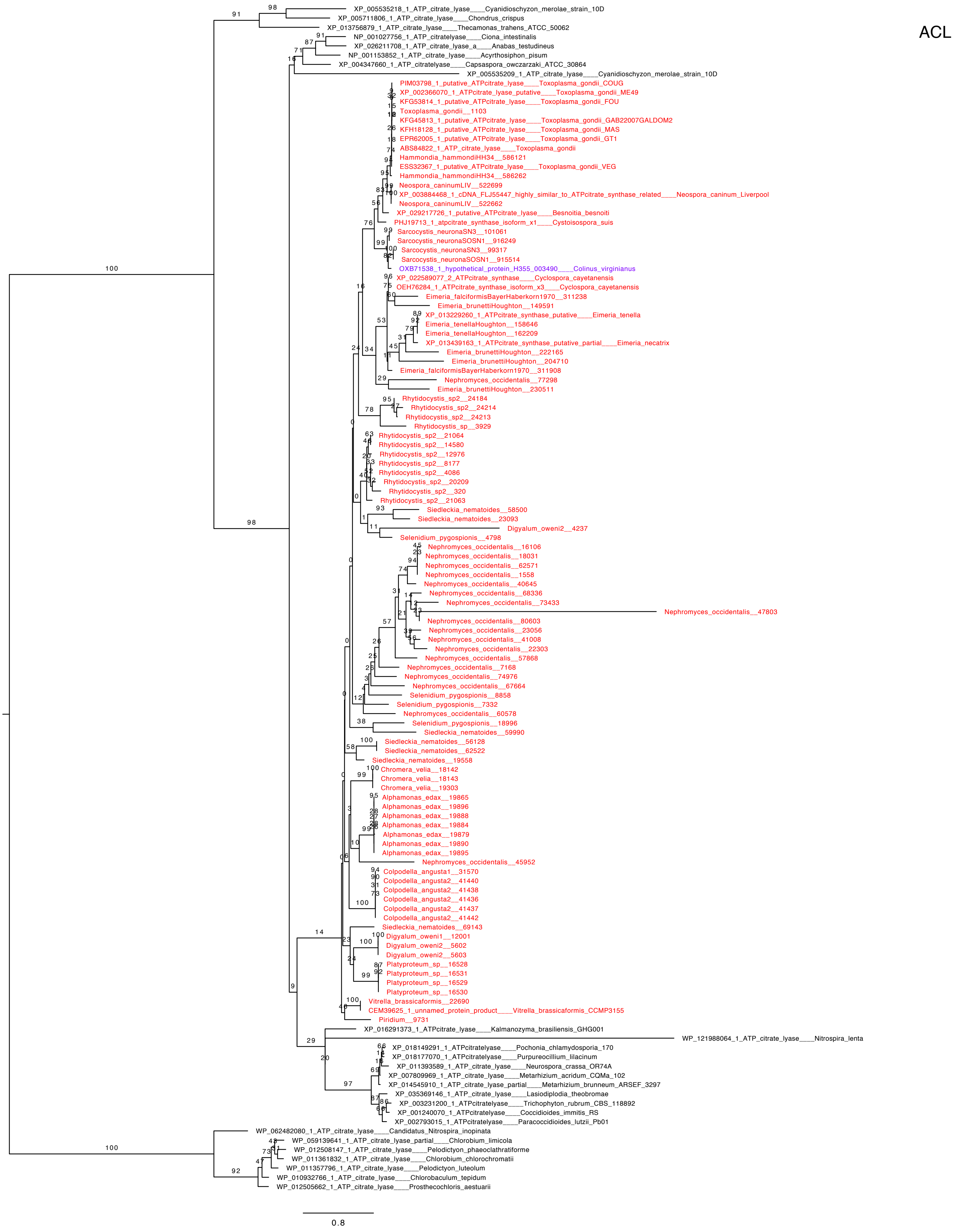

ACL
XP_005535218_1_ATP_citrate_lyase____Cyanidioschyzon_merolae_strain_10D
XP_005711806_1_ATP_citrate_lyase____Chondrus_crispus
XP_013756879_1_ATP_citrate_lyase____Thecamonas_trahens_ATCC_50062
NP_001027756_1_ATP_citratelyase____Ciona_intestinalis
XP_026211708_1_ATP_citrate_lyase_a____Anabas_testudineus
NP_001153852_1_ATP_citrate_lyase____Acyrthosiphon_pisum
XP_004347660_1_ATP_citratelyase____Capsaspora_owczarzaki_ATCC_30864
XP_005535209_1_ATP_citrate_lyase____Cyanidioschyzon_merolae_strain_10D
PIM03798_1_putative_ATPcitrate_lyase____Toxoplasma_gondii_COUG
XP_002366070_1_ATPcitrate_lyase_putative____Toxoplasma_gondii_ME49
KFG53814_1_putative_ATPcitrate_lyase____Toxoplasma_gondii_FOU
Toxoplasma_gondii__1103
KFG45813_1_putative_ATPcitrate_lyase____Toxoplasma_gondii_GAB22007GALDOM2
KFH18128_1_putative_ATPcitrate_lyase____Toxoplasma_gondii_MAS
EPR62005_1_putative_ATPcitrate_lyase____Toxoplasma_gondii_GT1
ABS84822_1_ATP_citrate_lyase____Toxoplasma_gondii
Hammondia_hammondiHH34__586121
ESS32367_1_putative_ATPcitrate_lyase____Toxoplasma_gondii_VEG
Hammondia_hammondiHH34__586262
Neospora_caninumLIV__522699
XP_003884468_1_cDNA_FLJ55447_highly_similar_to_ATPcitrate_synthase_related____Neospora_caninum_Liverpool
Neospora_caninumLIV__522662
XP_029217726_1_putative_ATPcitrate_lyase____Besnoitia_besnoiti
PHJ19713_1_atpcitrate_synthase_isoform_x1____Cystoisospora_suis
Sarcocystis_neuronaSN3__101061
Sarcocystis_neuronaSOSN1__916249
Sarcocystis_neuronaSN3__99317
Sarcocystis_neuronaSOSN1__915514
OXB71538_1_hypothetical_protein_H355_003490____Colinus_virginianus
XP_022589077_2_ATPcitrate_synthase____Cyclospora_cayetanensis
OEH76284_1_ATPcitrate_synthase_isoform_x3____Cyclospora_cayetanensis
Eimeria_falciformisBayerHaberkorn1970__311238
Eimeria_brunettiHoughton__149591
XP_013229260_1_ATPcitrate_synthase_putative____Eimeria_tenella
Eimeria_tenellaHoughton__158646
Eimeria_tenellaHoughton__162209
XP_013439163_1_ATPcitrate_synthase_putative_partial____Eimeria_necatrix
Eimeria_brunettiHoughton__222165
Eimeria_brunettiHoughton__204710
Eimeria_falciformisBayerHaberkorn1970__311908
Nephromyces_occidentalis__77298
Eimeria_brunettiHoughton__230511
Rhytidocystis_sp2__24184
Rhytidocystis_sp2__24214
Rhytidocystis_sp2__24213
Rhytidocystis_sp__3929
Rhytidocystis_sp2__21064
Rhytidocystis_sp2__14580
Rhytidocystis_sp2__12976
Rhytidocystis_sp2__8177
Rhytidocystis_sp2__4086
Rhytidocystis_sp2__20209
Rhytidocystis_sp2__320
Rhytidocystis_sp2__21063
Siedleckia_nematoides__58500
Siedleckia_nematoides__23093
Digyalum_oweni2__4237
Selenidium_pygospionis__4798
Nephromyces_occidentalis__16106
Nephromyces_occidentalis__18031
Nephromyces_occidentalis__62571
Nephromyces_occidentalis__1558
Nephromyces_occidentalis__40645
Nephromyces_occidentalis__68336
Nephromyces_occidentalis__73433
Nephromyces_occidentalis__47803
Nephromyces_occidentalis__80603
Nephromyces_occidentalis__23056
Nephromyces_occidentalis__41008
Nephromyces_occidentalis__22303
Nephromyces_occidentalis__57868
Nephromyces_occidentalis__7168
Nephromyces_occidentalis__74976
Nephromyces_occidentalis__67664
Selenidium_pygospionis__8858
Selenidium_pygospionis__7332
Nephromyces_occidentalis__60578
Selenidium_pygospionis__18996
Siedleckia_nematoides__59990
Siedleckia_nematoides__56128
Siedleckia_nematoides__62522
Siedleckia_nematoides__19558
Chromera_velia__18142
Chromera_velia__18143
Chromera_velia__19303
Alphamonas_edax__19865
Alphamonas_edax__19896
Alphamonas_edax__19888
Alphamonas_edax__19884
Alphamonas_edax__19879
Alphamonas_edax__19890
Alphamonas_edax__19895
Nephromyces_occidentalis__45952
Colpodella_angusta1__31570
Colpodella_angusta2__41440
Colpodella_angusta2__41438
Colpodella_angusta2__41436
Colpodella_angusta2__41437
Colpodella_angusta2__41442
Siedleckia_nematoides__69143
Digyalum_oweni1__12001
Digyalum_oweni2__5602
Digyalum_oweni2__5603
Platyproteum_sp__16528
Platyproteum_sp__16531
Platyproteum_sp__16529
Platyproteum_sp__16530
Vitrella_brassicaformis__22690
CEM39625_1_unnamed_protein_product____Vitrella_brassicaformis_CCMP3155
Piridium__9731
XP_016291373_1_ATPcitrate_lyase____Kalmanozyma_brasiliensis_GHG001
WP_121988064_1_ATP_citrate_lyase____Nitrospira_lenta
XP_018149291_1_ATPcitratelyase____Pochonia_chlamydosporia_170
XP_018177070_1_ATPcitratelyase____Purpureocillium_lilacinum
XP_011393589_1_ATP_citrate_lyase____Neurospora_crassa_OR74A
XP_007809969_1_ATP_citrate_lyase____Metarhizium_acridum_CQMa_102
XP_014545910_1_ATP_citrate_lyase_partial____Metarhizium_brunneum_ARSEF_3297
XP_035369146_1_ATP_citrate_lyase____Lasiodiplodia_theobromae
XP_003231200_1_ATPcitratelyase____Trichophyton_rubrum_CBS_118892
XP_001240070_1_ATPcitratelyase____Coccidioides_immitis_RS
XP_002793015_1_ATPcitratelyase____Paracoccidioides_lutzii_Pb01
WP_062482080_1_ATP_citrate_lyase____Candidatus_Nitrospira_inopinata
WP_059139641_1_ATP_citrate_lyase_partial____Chlorobium_limicola
WP_012508147_1_ATP_citrate_lyase____Pelodictyon_phaeoclathratiforme
WP_011361832_1_ATP_citrate_lyase____Chlorobium_chlorochromatii
WP_011357796_1_ATP_citrate_lyase____Pelodictyon_luteolum
WP_010932766_1_ATP_citrate_lyase____Chlorobaculum_tepidum
WP_012505662_1_ATP_citrate_lyase____Prosthecochloris_aestuarii
0.8

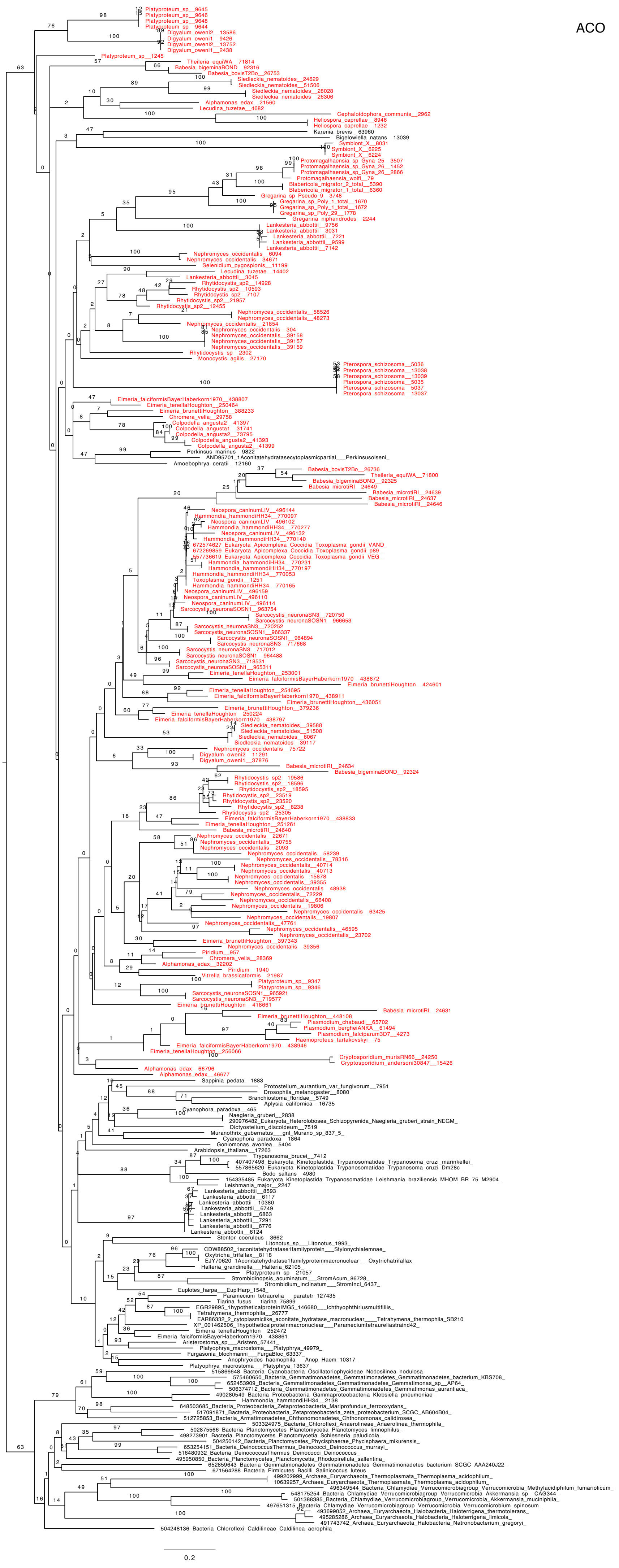

ACO
0.2

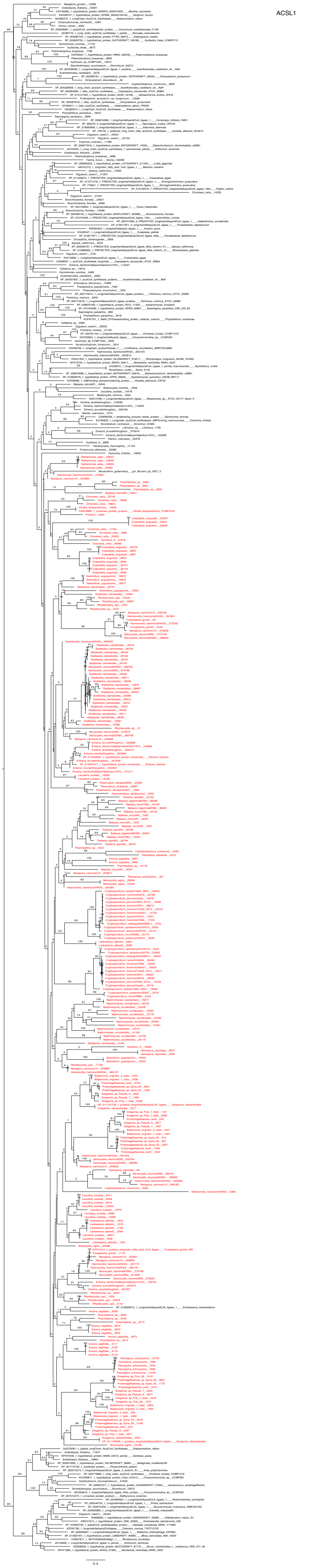
ACSL1
0.3

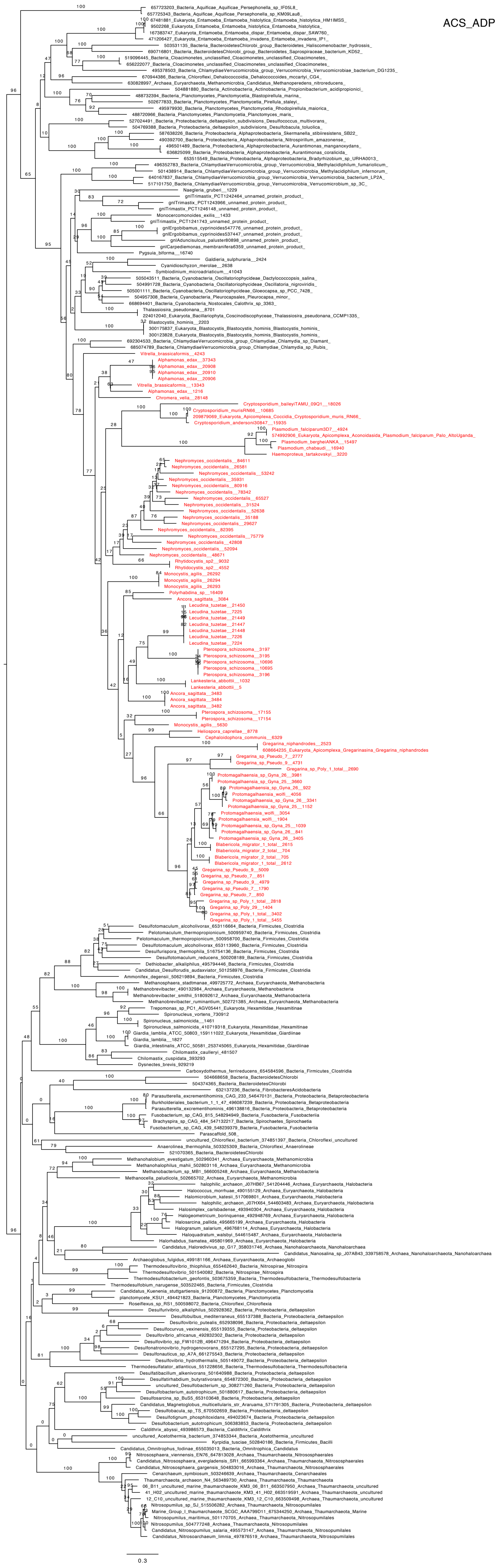
ACS_ADP
0.3

ACS_AMP

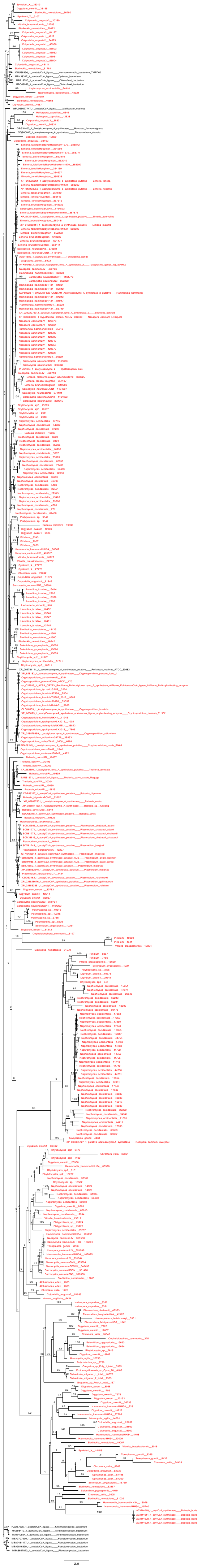

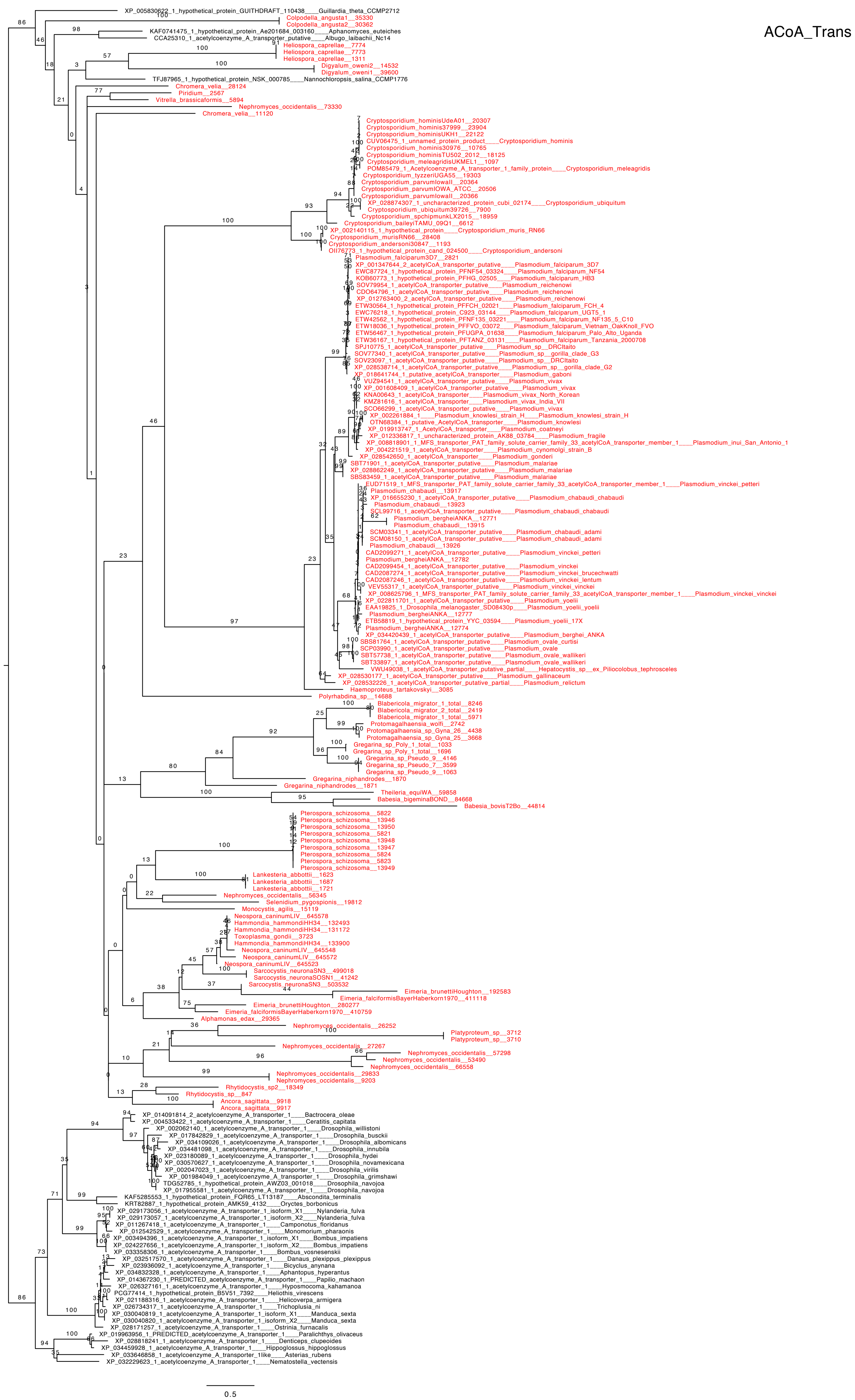
ACoA_Trans
XP_005830622_1_hypothetical_protein_GUITHDRAFT_110438____Guillardia_theta_CCMP2712
Colpodella_angusta1__35330
Colpodella_angusta2__30362
KAF0741475_1_hypothetical_protein_Ae201684_003160____Aphanomyces_euteiches
CCA25310_1_acetylcoenzyme_A_transporter_putative____Albugo_laibachii_Nc14
Heliospora_caprellae__7774
Heliospora_caprellae__7773
Heliospora_caprellae__1311
Digyalum_oweni2__14532
Digyalum_oweni1__39600
TFJ87965_1_hypothetical_protein_NSK_000785____Nannochloropsis_salina_CCMP1776
Chromera_velia__28124
Piridium__2567
Vitrella_brassicaformis__5894
Nephromyces_occidentalis__73330
Chromera_velia__11120
Cryptosporidium_hominisUdeA01__20307
Cryptosporidium_hominis37999__23904
Cryptosporidium_hominisUKH1__22122
CUV06475_1_unnamed_protein_product____Cryptosporidium_hominis
Cryptosporidium_hominis30976__10765
Cryptosporidium_hominisTU502_2012__18125
Cryptosporidium_meleagridisUKMEL1__1097
POM85479_1_Acetylcoenzyme_A_transporter_1_family_protein____Cryptosporidium_meleagridis
Cryptosporidium_tyzzeriUGA55__19303
Cryptosporidium_parvumIowaII__20364
Cryptosporidium_parvumIOWA_ATCC__20506
Cryptosporidium_parvumIowaII__20366
XP_028874307_1_uncharacterized_protein_cubi_02174____Cryptosporidium_ubiquitum
Cryptosporidium_ubiquitum39726__7900
Cryptosporidium_spchipmunkLX2015__18959
Cryptosporidium_baileyiTAMU_09Q1__6612
XP_002140115_1_hypothetical_protein____Cryptosporidium_muris_RN66
Cryptosporidium_murisRN66__28408
Cryptosporidium_andersoni30847__1193
OII76773_1_hypothetical_protein_cand_024500____Cryptosporidium_andersoni
Plasmodium_falciparum3D7__2821
XP_001347644_2_acetylCoA_transporter_putative____Plasmodium_falciparum_3D7
EWC87724_1_hypothetical_protein_PFNF54_03324____Plasmodium_falciparum_NF54
KOB60773_1_hypothetical_protein_PFHG_02505____Plasmodium_falciparum_HB3
SOV79954_1_acetylCoA_transporter_putative____Plasmodium_reichenowi
CDO64796_1_acetylCoA_transporter_putative____Plasmodium_reichenowi
XP_012763400_2_acetylCoA_transporter_putative____Plasmodium_reichenowi
ETW30564_1_hypothetical_protein_PFFCH_02021____Plasmodium_falciparum_FCH_4
EWC76218_1_hypothetical_protein_C923_03144____Plasmodium_falciparum_UGT5_1
ETW42562_1_hypothetical_protein_PFNF135_03221____Plasmodium_falciparum_NF135_5_C10
ETW18036_1_hypothetical_protein_PFFVO_03072____Plasmodium_falciparum_Vietnam_OakKnoll_FVO
ETW56467_1_hypothetical_protein_PFUGPA_01638____Plasmodium_falciparum_Palo_Alto_Uganda
ETW36167_1_hypothetical_protein_PFTANZ_03131____Plasmodium_falciparum_Tanzania_2000708
SPJ10775_1_acetylCoA_transporter_putative____Plasmodium_sp__DRC_Itaito
SOV77340_1_acetylCoA_transporter_putative____Plasmodium_sp__gorilla_clade_G3
SOV23097_1_acetylCoA_transporter_putative____Plasmodium_sp__DRC_Itaito
XP_028538714_1_acetylCoA_transporter_putative____Plasmodium_sp__gorilla_clade_G2
XP_018641744_1_putative_acetylCoA_transporter____Plasmodium_gaboni
VUZ94541_1_acetylCoA_transporter_putative____Plasmodium_vivax
XP_001608409_1_acetylCoA_transporter_putative____Plasmodium_vivax
KNA00643_1_acetylCoA_transporter____Plasmodium_vivax_North_Korean
KMZ81616_1_acetylCoA_transporter____Plasmodium_vivax_India_VII
SCO66299_1_acetylCoA_transporter_putative____Plasmodium_vivax
XP_002261884_1____Plasmodium_knowlesi_strain_H____Plasmodium_knowlesi_strain_H
OTN68384_1_putative_AcetylCoA_transporter____Plasmodium_knowlesi
XP_019913747_1_AcetylCoA_transporter____Plasmodium_coatneyi
XP_012336817_1_uncharacterized_protein_AK88_03784____Plasmodium_fragile
XP_008818901_1_MFS_transporter_PAT_family_solute_carrier_family_33_acetylCoA_transporter_member_1____Plasmodium_inui_San_Antonio_1
XP_004221519_1_acetylCoA_transporter____Plasmodium_cynomolgi_strain_B
XP_028542650_1_acetylCoA_transporter____Plasmodium_gonderi
SBT71901_1_acetylCoA_transporter_putative____Plasmodium_malariae
XP_028862249_1_acetylCoA_transporter_putative____Plasmodium_malariae
SBS83459_1_acetylCoA_transporter_putative____Plasmodium_malariae
EUD71519_1_MFS_transporter_PAT_family_solute_carrier_family_33_acetylCoA_transporter_member_1____Plasmodium_vinckei_petteri
Plasmodium_chabaudi__13917
XP_016655230_1_acetylCoA_transporter_putative____Plasmodium_chabaudi_chabaudi
Plasmodium_chabaudi__13923
SCL99716_1_acetylCoA_transporter_putative____Plasmodium_chabaudi_chabaudi
Plasmodium_bergheiANKA__12771
Plasmodium_chabaudi__13915
SCM03341_1_acetylCoA_transporter_putative____Plasmodium_chabaudi_adami
SCM08150_1_acetylCoA_transporter_putative____Plasmodium_chabaudi_adami
Plasmodium_chabaudi__13926
CAD2099271_1_acetylCoA_transporter_putative____Plasmodium_vinckei_petteri
Plasmodium_bergheiANKA__12782
CAD2099454_1_acetylCoA_transporter_putative____Plasmodium_vinckei
CAD2087274_1_acetylCoA_transporter_putative____Plasmodium_vinckei_brucechwatti
CAD2087246_1_acetylCoA_transporter_putative____Plasmodium_vinckei_lentum
VEV55317_1_acetylCoA_transporter_putative____Plasmodium_vinckei_vinckei
XP_008625796_1_MFS_transporter_PAT_family_solute_carrier_family_33_acetylCoA_transporter_member_1____Plasmodium_vinckei_vinckei
XP_022811701_1_acetylCoA_transporter_putative____Plasmodium_yoelii
EAA19825_1_Drosophila_melanogaster_SD08430p____Plasmodium_yoelii_yoelii
Plasmodium_bergheiANKA__12777
ETB58819_1_hypothetical_protein_YYC_03594____Plasmodium_yoelii_17X
Plasmodium_bergheiANKA__12774
XP_034420439_1_acetylCoA_transporter_putative____Plasmodium_berghei_ANKA
SBS81764_1_acetylCoA_transporter_putative____Plasmodium_ovale_curtisi
SCP03990_1_acetylCoA_transporter_putative____Plasmodium_ovale
SBT57738_1_acetylCoA_transporter_putative____Plasmodium_ovale_wallikeri
SBT33897_1_acetylCoA_transporter_putative____Plasmodium_ovale_wallikeri
VWU49038_1_acetylCoA_transporter_putative_partial____Hepatocystis_sp__ex_Piliocolobus_tephrosceles
XP_028530177_1_acetylCoA_transporter_putative____Plasmodium_gallinaceum
XP_028532226_1_acetylCoA_transporter_putative_partial____Plasmodium_relictum
Haemoproteus_tartakovskyi__3085
Polyrhabdina_sp__14688
Blabericola_migrator_1_total__8246
Blabericola_migrator_2_total__2419
Blabericola_migrator_1_total__5971
Protomagalhaensia_wolfi__2742
Protomagalhaensia_sp_Gyna_26__4438
Protomagalhaensia_sp_Gyna_25__3668
Gregarina_sp_Poly_1_total__1033
Gregarina_sp_Poly_1_total__1696
Gregarina_sp_Pseudo_9__4146
Gregarina_sp_Pseudo_7__3599
Gregarina_sp_Pseudo_9__1063
Gregarina_niphandrodes__1870
Gregarina_niphandrodes__1871
Theileria_equiWA__59858
Babesia_bigeminaBOND__84668
Babesia_bovisT2Bo__44814
Pterospora_schizosoma__5822
Pterospora_schizosoma__13946
Pterospora_schizosoma__13950
Pterospora_schizosoma__5821
Pterospora_schizosoma__13948
Pterospora_schizosoma__13947
Pterospora_schizosoma__5824
Pterospora_schizosoma__5823
Pterospora_schizosoma__13949
Lankesteria_abbottii__1623
Lankesteria_abbottii__1687
Lankesteria_abbottii__1721
Nephromyces_occidentalis__56345
Selenidium_pygospionis__19812
Monocystis_agilis__15119
Neospora_caninumLIV__645578
Hammondia_hammondiHH34__132493
Hammondia_hammondiHH34__131172
Toxoplasma_gondii__3723
Hammondia_hammondiHH34__133900
Neospora_caninumLIV__645548
Neospora_caninumLIV__645572
Neospora_caninumLIV__645523
Sarcocystis_neuronaSN3__499018
Sarcocystis_neuronaSOSN1__41242
Sarcocystis_neuronaSN3__503532
Eimeria_brunettiHoughton__192583
Eimeria_falciformisBayerHaberkorn1970__411118
Eimeria_brunettiHoughton__280277
Eimeria_falciformisBayerHaberkorn1970__410759
Alphamonas_edax__29365
Nephromyces_occidentalis__26252
Platyproteum_sp__3712
Platyproteum_sp__3710
Nephromyces_occidentalis__27267
Nephromyces_occidentalis__57298
Nephromyces_occidentalis__53490
Nephromyces_occidentalis__66558
Nephromyces_occidentalis__29833
Nephromyces_occidentalis__9203
Rhytidocystis_sp2__18349
Rhytidocystis_sp__847
Ancora_sagittata__9918
Ancora_sagittata__9917
XP_014091814_2_acetylcoenzyme_A_transporter_1____Bactrocera_oleae
XP_004533422_1_acetylcoenzyme_A_transporter_1____Ceratitis_capitata
XP_002062140_1_acetylcoenzyme_A_transporter_1____Drosophila_willistoni
XP_017842829_1_acetylcoenzyme_A_transporter_1____Drosophila_busckii
XP_034109026_1_acetylcoenzyme_A_transporter_1____Drosophila_albomicans
XP_034481098_1_acetylcoenzyme_A_transporter_1____Drosophila_innubila
XP_023180089_1_acetylcoenzyme_A_transporter_1____Drosophila_hydei
XP_030570627_1_acetylcoenzyme_A_transporter_1____Drosophila_novamexicana
XP_002047023_1_acetylcoenzyme_A_transporter_1____Drosophila_virilis
XP_001984049_1_acetylcoenzyme_A_transporter_1____Drosophila_grimshawi
TDG52785_1_hypothetical_protein_AWZ03_001018____Drosophila_navojoa
XP_017955581_1_acetylcoenzyme_A_transporter_1____Drosophila_navojoa
KAF5285553_1_hypothetical_protein_FQR65_LT13187____Abscondita_terminalis
KRT82887_1_hypothetical_protein_AMK59_4132____Oryctes_borbonicus
XP_029173056_1_acetylcoenzyme_A_transporter_1_isoform_X1____Nylanderia_fulva
XP_029173057_1_acetylcoenzyme_A_transporter_1_isoform_X2____Nylanderia_fulva
XP_011267418_1_acetylcoenzyme_A_transporter_1____Camponotus_floridanus
XP_012542529_1_acetylcoenzyme_A_transporter_1____Monomorium_pharaonis
XP_003494396_1_acetylcoenzyme_A_transporter_1_isoform_X1____Bombus_impatiens
XP_024227656_1_acetylcoenzyme_A_transporter_1_isoform_X2____Bombus_impatiens
XP_033358306_1_acetylcoenzyme_A_transporter_1____Bombus_vosnesenskii
XP_032517570_1_acetylcoenzyme_A_transporter_1____Danaus_plexippus_plexippus
XP_023936092_1_acetylcoenzyme_A_transporter_1____Bicyclus_anynana
XP_034832328_1_acetylcoenzyme_A_transporter_1____Aphantopus_hyperantus
XP_014367230_1_PREDICTED_acetylcoenzyme_A_transporter_1____Papilio_machaon
XP_026327161_1_acetylcoenzyme_A_transporter_1____Hyposmocoma_kahamanoa
PCG77414_1_hypothetical_protein_B5V51_7392____Heliothis_virescens
XP_021188316_1_acetylcoenzyme_A_transporter_1____Helicoverpa_armigera
XP_026734317_1_acetylcoenzyme_A_transporter_1____Trichoplusia_ni
XP_030040819_1_acetylcoenzyme_A_transporter_1_isoform_X1____Manduca_sexta
XP_030040820_1_acetylcoenzyme_A_transporter_1_isoform_X2____Manduca_sexta
XP_028171257_1_acetylcoenzyme_A_transporter_1____Ostrinia_furnacalis
XP_019963956_1_PREDICTED_acetylcoenzyme_A_transporter_1____Paralichthys_olivaceus
XP_028818241_1_acetylcoenzyme_A_transporter_1____Denticeps_clupeoides
XP_034459928_1_acetylcoenzyme_A_transporter_1____Hippoglossus_hippoglossus
XP_033646858_1_acetylcoenzyme_A_transporter_1like____Asterias_rubens
XP_032229623_1_acetylcoenzyme_A_transporter_1____Nematostella_vectensis
0.5

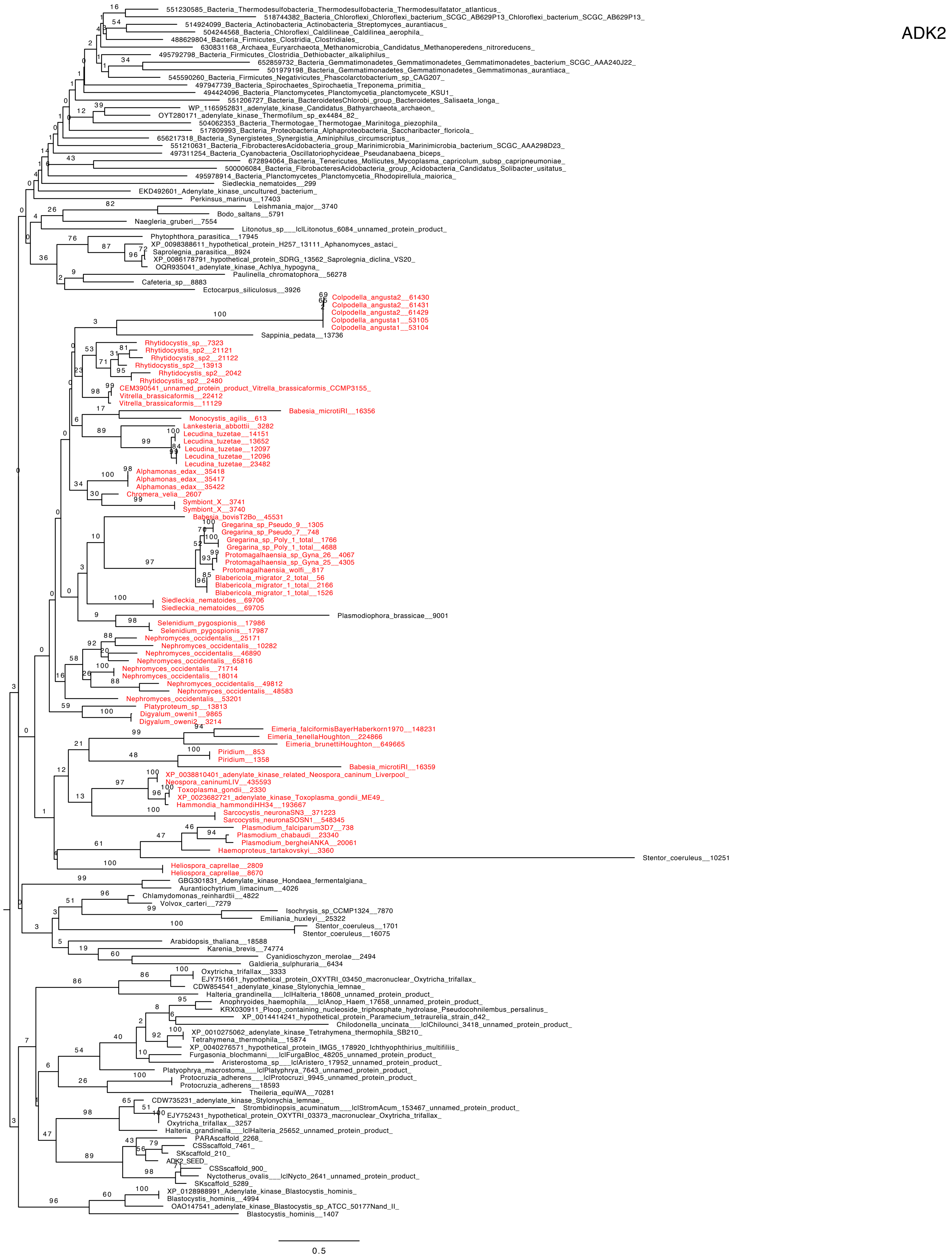
ADK2
551230585_Bacteria_Thermodesulfobacteria_Thermodesulfobacteria_Thermodesulfatator_atlanticus_
518744382_Bacteria_Chloroflexi_Chloroflexi_bacterium_SCGC_AB629P13_Chloroflexi_bacterium_SCGC_AB629P13_
514924099_Bacteria_Actinobacteria_Actinobacteria_Streptomyces_aurantiacus_
504244568_Bacteria_Chloroflexi_Caldilineae_Caldilinea_aerophila_
488629804_Bacteria_Firmicutes_Clostridia_Clostridiales_
630831168_Archaea_Euryarchaeota_Methanomicrobia_Candidatus_Methanoperedens_nitroreducens_
495792798_Bacteria_Firmicutes_Clostridia_Dethiobacter_alkaliphilus_
652859732_Bacteria_Gemmatimonadetes_Gemmatimonadetes_bacterium_SCGC_AAA240J22_
501979198_Bacteria_Gemmatimonadetes_Gemmatimonadetes_Gemmatimonas_aurantiaca_
545590260_Bacteria_Firmicutes_Negativicutes_Phascolarctobacterium_sp_CAG207_
497947739_Bacteria_Spirochaetes_Spirochaetia_Treponema_primitia_
494424096_Bacteria_Planctomycetes_Planctomycetia_planctomycete_KSU1_
551206727_Bacteria_BacteroidetesChlorobi_group_Bacteroidetes_Salisaeta_longa_
WP_1165952831_adenylate_kinase_Candidatus_Bathyarchaeota_archaeon_
OYT280171_adenylate_kinase_Thermofilum_sp_ex4484_82_
504062353_Bacteria_Thermotogae_Thermotogae_Marinitoga_piezophila_
517809993_Bacteria_Proteobacteria_Alphaproteobacteria_Saccharibacter_floricola_
656217318_Bacteria_Synergistetes_Synergistia_Aminiphilus_circumscriptus_
551210631_Bacteria_FibrobacteresAcidobacteria_group_Marinimicrobia_Marinimicrobia_bacterium_SCGC_AAA298D23_
497311254_Bacteria_Cyanobacteria_Oscillatoriophycideae_Pseudanabaena_biceps_
672894064_Bacteria_Tenericutes_Mollicutes_Mycoplasma_capricolum_subsp_capripneumoniae_
500006084_Bacteria_FibrobacteresAcidobacteria_group_Acidobacteria_Solibacter_usitatus_
495978914_Bacteria_Planctomycetes_Planctomycetia_Rhodopirellula_maiorica_
Siedleckia_nematoides__299
EKD492601_Adenylate_kinase_uncultured_bacterium_
Perkinsus_marinus__17403
Leishmania_major__3740
Bodo_saltans__5791
Naegleria_gruberi__7554
Litonotus_sp___lclLitonotus_6084_unnamed_protein_product_
Phytophthora_parasitica__17945
XP_0098388611_hypothetical_protein_H257_13111_Aphanomyces_astaci_
Saprolegnia_parasitica__8924
XP_0086178791_hypothetical_protein_SDRG_13562_Saprolegnia_diclina_VS20_
OQR935041_adenylate_kinase_Achlya_hypogyna_
Paulinella_chromatophora__56278
Cafeteria_sp__8883
Ectocarpus_siliculosus__3926
Colpodella_angusta2__61430
Colpodella_angusta2__61431
Colpodella_angusta2__61429
Colpodella_angusta1__53105
Colpodella_angusta1__53104
Sappinia_pedata__13736
Rhytidocystis_sp__7323
Rhytidocystis_sp2__21121
Rhytidocystis_sp2__21122
Rhytidocystis_sp2__13913
Rhytidocystis_sp2__2042
Rhytidocystis_sp2__2480
CEM390541_unnamed_protein_product_Vitrella_brassicaformis_CCMP3155_
Vitrella_brassicaformis__22412
Vitrella_brassicaformis__11129
Babesia_microtiRI__16356
Monocystis_agilis__613
Lankesteria_abbottii__3282
Lecudina_tuzetae__14151
Lecudina_tuzetae__13652
Lecudina_tuzetae__12097
Lecudina_tuzetae__12096
Lecudina_tuzetae__23482
Alphamonas_edax__35418
Alphamonas_edax__35417
Alphamonas_edax__35422
Chromera_velia__2607
Symbiont_X__3741
Symbiont_X__3740
Babesia_bovisT2Bo__45531
Gregarina_sp_Pseudo_9__1305
Gregarina_sp_Pseudo_7__748
Gregarina_sp_Poly_1_total__1766
Gregarina_sp_Poly_1_total__4688
Protomagalhaensia_sp_Gyna_26__4067
Protomagalhaensia_sp_Gyna_25__4305
Protomagalhaensia_wolfi__817
Blabericola_migrator_2_total__56
Blabericola_migrator_1_total__2166
Blabericola_migrator_1_total__1526
Siedleckia_nematoides__69706
Siedleckia_nematoides__69705
Plasmodiophora_brassicae__9001
Selenidium_pygospionis__17986
Selenidium_pygospionis__17987
Nephromyces_occidentalis__25171
Nephromyces_occidentalis__10282
Nephromyces_occidentalis__46890
Nephromyces_occidentalis__65816
Nephromyces_occidentalis__71714
Nephromyces_occidentalis__18014
Nephromyces_occidentalis__49812
Nephromyces_occidentalis__48583
Nephromyces_occidentalis__53201
Platyproteum_sp__13813
Digyalum_oweni1__9865
Digyalum_oweni2__3214
Eimeria_falciformisBayerHaberkorn1970__148231
Eimeria_tenellaHoughton__224866
Eimeria_brunettiHoughton__649665
Piridium__853
Piridium__1358
Babesia_microtiRI__16359
XP_0038810401_adenylate_kinase_related_Neospora_caninum_Liverpool_
Neospora_caninumLIV__435593
Toxoplasma_gondii__2330
XP_0023682721_adenylate_kinase_Toxoplasma_gondii_ME49_
Hammondia_hammondiHH34__193667
Sarcocystis_neuronaSN3__371223
Sarcocystis_neuronaSOSN1__548345
Plasmodium_falciparum3D7__738
Plasmodium_chabaudi__23340
Plasmodium_bergheiANKA__20061
Haemoproteus_tartakovskyi__3360
Stentor_coeruleus__10251
Heliospora_caprellae__2809
Heliospora_caprellae__8670
GBG301831_Adenylate_kinase_Hondaea_fermentalgiana_
Aurantiochytrium_limacinum__4026
Chlamydomonas_reinhardtii__4822
Volvox_carteri__7279
Isochrysis_sp_CCMP1324__7870
Emiliania_huxleyi__25322
Stentor_coeruleus__1701
Stentor_coeruleus__16075
Arabidopsis_thaliana__18588
Karenia_brevis__74774
Cyanidioschyzon_merolae__2494
Galdieria_sulphuraria__6434
Oxytricha_trifallax__3333
EJY751661_hypothetical_protein_OXYTRI_03450_macronuclear_Oxytricha_trifallax_
CDW854541_adenylate_kinase_Stylonychia_lemnae_
Halteria_grandinella___lclHalteria_18608_unnamed_protein_product_
Anophryoides_haemophila___lclAnop_Haem_17658_unnamed_protein_product_
KRX030911_Ploop_containing_nucleoside_triphosphate_hydrolase_Pseudocohnilembus_persalinus_
XP_0014414241_hypothetical_protein_Paramecium_tetraurelia_strain_d42_
Chilodonella_uncinata___lclChilounci_3418_unnamed_protein_product_
XP_0010275062_adenylate_kinase_Tetrahymena_thermophila_SB210_
Tetrahymena_thermophila__15874
XP_0040276571_hypothetical_protein_IMG5_178920_Ichthyophthirius_multifiliis_
Furgasonia_blochmanni___lclFurgaBloc_48205_unnamed_protein_product_
Aristerostoma_sp___lclAristero_17952_unnamed_protein_product_
Platyophrya_macrostoma___lclPlatyphrya_7643_unnamed_protein_product_
Protocruzia_adherens___lclProtocruzi_9945_unnamed_protein_product_
Protocruzia_adherens__18593
Theileria_equiWA__70281
CDW735231_adenylate_kinase_Stylonychia_lemnae_
Strombidinopsis_acuminatum___lclStromAcum_153467_unnamed_protein_product_
EJY752431_hypothetical_protein_OXYTRI_03373_macronuclear_Oxytricha_trifallax_
Oxytricha_trifallax__3257
Halteria_grandinella___lclHalteria_25652_unnamed_protein_product_
PARAscaffold_2268_
CSSscaffold_7461_
SKscaffold_210_
ADK2_SEED_
CSSscaffold_900_
Nyctotherus_ovalis___lclNycto_2641_unnamed_protein_product_
SKscaffold_5289_
XP_0128988991_Adenylate_kinase_Blastocystis_hominis_
Blastocystis_hominis__4994
OAO147541_adenylate_kinase_Blastocystis_sp_ATCC_50177Nand_II_
Blastocystis_hominis__1407
0.5

ALDH7A1

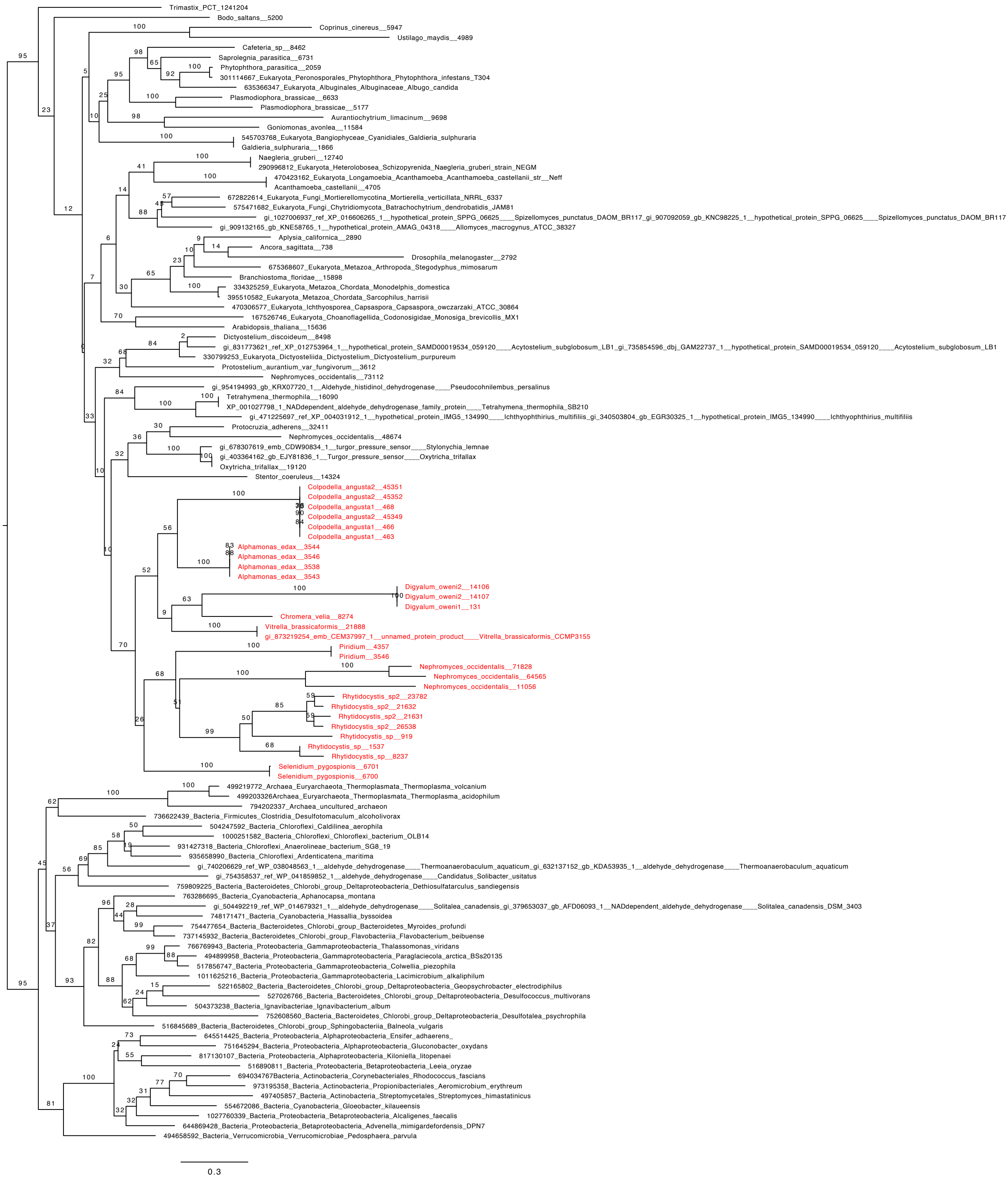
Trimastix_PCT_1241204
Bodo_saltans__5200
Coprinus_cinereus__5947
Ustilago_maydis__4989
Cafeteria_sp__8462
Saprolegnia_parasitica__6731
Phytophthora_parasitica__2059
301114667_Eukaryota_Peronosporales_Phytophthora_Phytophthora_infestans_T304
635366347_Eukaryota_Albuginales_Albuginaceae_Albugo_candida
Plasmodiophora_brassicae__6633
Plasmodiophora_brassicae__5177
Aurantiochytrium_limacinum__9698
Goniomonas_avonlea__11584
545703768_Eukaryota_Bangiophyceae_Cyanidiales_Galdieria_sulphuraria
Galdieria_sulphuraria__1866
Naegleria_gruberi__12740
290996812_Eukaryota_Heterolobosea_Schizopyrenida_Naegleria_gruberi_strain_NEGM
470423162_Eukaryota_Longamoebia_Acanthamoeba_Acanthamoeba_castellanii_str__Neff
Acanthamoeba_castellanii__4705
672822614_Eukaryota_Fungi_Mortierellomycotina_Mortierella_verticillata_NRRL_6337
575471682_Eukaryota_Fungi_Chytridiomycota_Batrachochytrium_dendrobatidis_JAM81
gi_1027006937_ref_XP_016606265_1__hypothetical_protein_SPPG_06625____Spizellomyces_punctatus_DAOM_BR117_gi_907092059_gb_KNC98225_1__hypothetical_protein_SPPG_06625____Spizellomyces_punctatus_DAOM_BR117
gi_909132165_gb_KNE58765_1__hypothetical_protein_AMAG_04318____Allomyces_macrogynus_ATCC_38327
Aplysia_californica__2890
Ancora_sagittata__738
Drosophila_melanogaster__2792
675368607_Eukaryota_Metazoa_Arthropoda_Stegodyphus_mimosarum
Branchiostoma_floridae__15898
334325259_Eukaryota_Metazoa_Chordata_Monodelphis_domestica
395510582_Eukaryota_Metazoa_Chordata_Sarcophilus_harrisii
470306577_Eukaryota_Ichthyosporea_Capsaspora_Capsaspora_owczarzaki_ATCC_30864
167526746_Eukaryota_Choanoflagellida_Codonosigidae_Monosiga_brevicollis_MX1
Arabidopsis_thaliana__15636
Dictyostelium_discoideum__8498
gi_831773621_ref_XP_012753964_1__hypothetical_protein_SAMD00019534_059120____Acytostelium_subglobosum_LB1_gi_735854596_dbj_GAM22737_1__hypothetical_protein_SAMD00019534_059120____Acytostelium_subglobosum_LB1
330799253_Eukaryota_Dictyosteliida_Dictyostelium_Dictyostelium_purpureum
Protostelium_aurantium_var_fungivorum__3612
Nephromyces_occidentalis__73112
gi_954194993_gb_KRX07720_1__Aldehyde_histidinol_dehydrogenase____Pseudocohnilembus_persalinus
Tetrahymena_thermophila__16090
XP_001027798_1_NADdependent_aldehyde_dehydrogenase_family_protein____Tetrahymena_thermophila_SB210
gi_471225697_ref_XP_004031912_1__hypothetical_protein_IMG5_134990____Ichthyophthirius_multifiliis_gi_340503804_gb_EGR30325_1__hypothetical_protein_IMG5_134990____Ichthyophthirius_multifiliis
Protocruzia_adherens__32411
Nephromyces_occidentalis__48674
gi_678307619_emb_CDW90834_1__turgor_pressure_sensor____Stylonychia_lemnae
gi_403364162_gb_EJY81836_1__Turgor_pressure_sensor____Oxytricha_trifallax
Oxytricha_trifallax__19120
Stentor_coeruleus__14324
Colpodella_angusta2__45351
Colpodella_angusta2__45352
Colpodella_angusta1__468
Colpodella_angusta2__45349
Colpodella_angusta1__466
Colpodella_angusta1__463
Alphamonas_edax__3544
Alphamonas_edax__3546
Alphamonas_edax__3538
Alphamonas_edax__3543
Digyalum_oweni2__14106
Digyalum_oweni2__14107
Digyalum_oweni1__131
Chromera_velia__8274
Vitrella_brassicaformis__21888
gi_873219254_emb_CEM37997_1__unnamed_protein_product____Vitrella_brassicaformis_CCMP3155
Piridium__4357
Piridium__3546
Nephromyces_occidentalis__71828
Nephromyces_occidentalis__64565
Nephromyces_occidentalis__11056
Rhytidocystis_sp2__23782
Rhytidocystis_sp2__21632
Rhytidocystis_sp2__21631
Rhytidocystis_sp2__26538
Rhytidocystis_sp__919
Rhytidocystis_sp__1537
Rhytidocystis_sp__8237
Selenidium_pygospionis__6701
Selenidium_pygospionis__6700
499219772_Archaea_Euryarchaeota_Thermoplasmata_Thermoplasma_volcanium
499203326Archaea_Euryarchaeota_Thermoplasmata_Thermoplasma_acidophilum
794202337_Archaea_uncultured_archaeon
736622439_Bacteria_Firmicutes_Clostridia_Desulfotomaculum_alcoholivorax
504247592_Bacteria_Chloroflexi_Caldilinea_aerophila
1000251582_Bacteria_Chloroflexi_Chloroflexi_bacterium_OLB14
931427318_Bacteria_Chloroflexi_Anaerolineae_bacterium_SG8_19
935658990_Bacteria_Chloroflexi_Ardenticatena_maritima
gi_740206629_ref_WP_038048563_1__aldehyde_dehydrogenase____Thermoanaerobaculum_aquaticum_gi_632137152_gb_KDA53935_1__aldehyde_dehydrogenase____Thermoanaerobaculum_aquaticum
gi_754358537_ref_WP_041859852_1__aldehyde_dehydrogenase____Candidatus_Solibacter_usitatus
759809225_Bacteria_Bacteroidetes_Chlorobi_group_Deltaproteobacteria_Dethiosulfatarculus_sandiegensis
763286695_Bacteria_Cyanobacteria_Aphanocapsa_montana
gi_504492219_ref_WP_014679321_1__aldehyde_dehydrogenase____Solitalea_canadensis_gi_379653037_gb_AFD06093_1__NADdependent_aldehyde_dehydrogenase____Solitalea_canadensis_DSM_3403
748171471_Bacteria_Cyanobacteria_Hassallia_byssoidea
754477654_Bacteria_Bacteroidetes_Chlorobi_group_Bacteroidetes_Myroides_profundi
737145932_Bacteria_Bacteroidetes_Chlorobi_group_Flavobacteriia_Flavobacterium_beibuense
766769943_Bacteria_Proteobacteria_Gammaproteobacteria_Thalassomonas_viridans
494899958_Bacteria_Proteobacteria_Gammaproteobacteria_Paraglaciecola_arctica_BSs20135
517856747_Bacteria_Proteobacteria_Gammaproteobacteria_Colwellia_piezophila
1011625216_Bacteria_Proteobacteria_Gammaproteobacteria_Lacimicrobium_alkaliphilum
522165802_Bacteria_Bacteroidetes_Chlorobi_group_Deltaproteobacteria_Geopsychrobacter_electrodiphilus
527026766_Bacteria_Bacteroidetes_Chlorobi_group_Deltaproteobacteria_Desulfococcus_multivorans
504373238_Bacteria_Ignavibacteriae_Ignavibacterium_album
752608560_Bacteria_Bacteroidetes_Chlorobi_group_Deltaproteobacteria_Desulfotalea_psychrophila
516845689_Bacteria_Bacteroidetes_Chlorobi_group_Sphingobacteriia_Balneola_vulgaris
645514425_Bacteria_Proteobacteria_Alphaproteobacteria_Ensifer_adhaerens_
751645294_Bacteria_Proteobacteria_Alphaproteobacteria_Gluconobacter_oxydans
817130107_Bacteria_Proteobacteria_Alphaproteobacteria_Kiloniella_litopenaei
516890811_Bacteria_Proteobacteria_Betaproteobacteria_Leeia_oryzae
694034767Bacteria_Actinobacteria_Corynebacteriales_Rhodococcus_fascians
973195358_Bacteria_Actinobacteria_Propionibacteriales_Aeromicrobium_erythreum
497405857_Bacteria_Actinobacteria_Streptomycetales_Streptomyces_himastatinicus
554672086_Bacteria_Cyanobacteria_Gloeobacter_kilaueensis
1027760339_Bacteria_Proteobacteria_Betaproteobacteria_Alcaligenes_faecalis
644869428_Bacteria_Proteobacteria_Betaproteobacteria_Advenella_mimigardefordensis_DPN7
494658592_Bacteria_Verrucomicrobia_Verrucomicrobiae_Pedosphaera_parvula
0.3

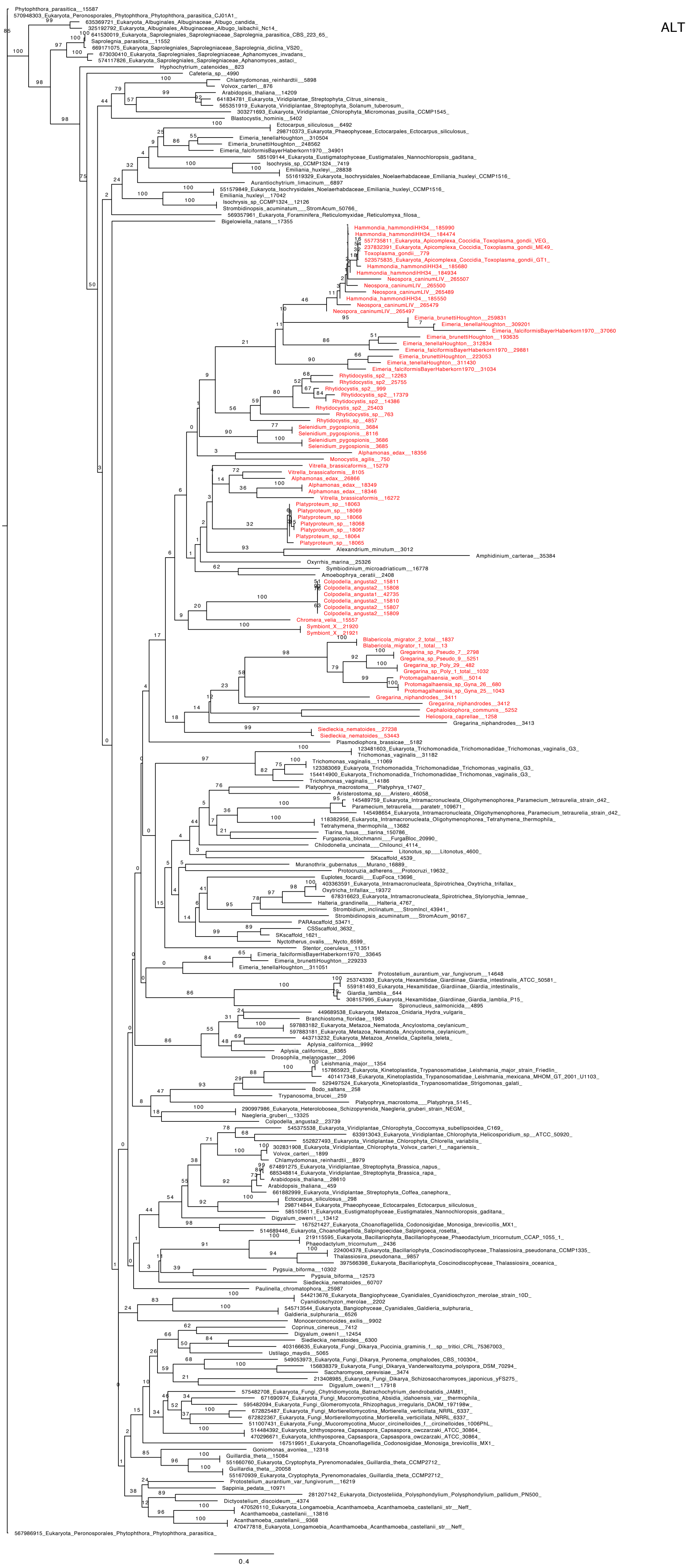

ALT
Phytophthora_parasitica__15587
570948303_Eukaryota_Peronosporales_Phytophthora_Phytophthora_parasitica_CJ01A1_
635369721_Eukaryota_Albuginales_Albuginaceae_Albugo_candida_
325192792_Eukaryota_Albuginales_Albuginaceae_Albugo_laibachii_Nc14_
641530019_Eukaryota_Saprolegniales_Saprolegniaceae_Saprolegnia_parasitica_CBS_223_65_
Saprolegnia_parasitica__11552
669171075_Eukaryota_Saprolegniales_Saprolegniaceae_Saprolegnia_diclina_VS20_
673030410_Eukaryota_Saprolegniales_Saprolegniaceae_Aphanomyces_invadans_
574117826_Eukaryota_Saprolegniales_Saprolegniaceae_Aphanomyces_astaci_
Hyphochytrium_catenoides__823
Cafeteria_sp__4990
Chlamydomonas_reinhardtii__5898
Volvox_carteri__876
Arabidopsis_thaliana__14209
641834781_Eukaryota_Viridiplantae_Streptophyta_Citrus_sinensis_
565351919_Eukaryota_Viridiplantae_Streptophyta_Solanum_tuberosum_
303271693_Eukaryota_Viridiplantae_Chlorophyta_Micromonas_pusilla_CCMP1545_
Blastocystis_hominis__5402
Ectocarpus_siliculosus__6492
298710373_Eukaryota_Phaeophyceae_Ectocarpales_Ectocarpus_siliculosus_
Eimeria_tenellaHoughton__310504
Eimeria_brunettiHoughton__248562
Eimeria_falciformisBayerHaberkorn1970__34901
585109144_Eukaryota_Eustigmatophyceae_Eustigmatales_Nannochloropsis_gaditana_
Isochrysis_sp_CCMP1324__7419
Emiliania_huxleyi__28838
551619329_Eukaryota_Isochrysidales_Noelaerhabdaceae_Emiliania_huxleyi_CCMP1516_
Aurantiochytrium_limacinum__6897
551579849_Eukaryota_Isochrysidales_Noelaerhabdaceae_Emiliania_huxleyi_CCMP1516_
Emiliania_huxleyi__17042
Isochrysis_sp_CCMP1324__12126
Strombidinopsis_acuminatum___StromAcum_50766_
569357961_Eukaryota_Foraminifera_Reticulomyxidae_Reticulomyxa_filosa_
Bigelowiella_natans__17355
Hammondia_hammondiHH34__185990
Hammondia_hammondiHH34__184474
557735811_Eukaryota_Apicomplexa_Coccidia_Toxoplasma_gondii_VEG_
237832391_Eukaryota_Apicomplexa_Coccidia_Toxoplasma_gondii_ME49_
Toxoplasma_gondii__779
523575835_Eukaryota_Apicomplexa_Coccidia_Toxoplasma_gondii_GT1_
Hammondia_hammondiHH34__185680
Hammondia_hammondiHH34__184934
Neospora_caninumLIV__265507
Neospora_caninumLIV__265500
Neospora_caninumLIV__265489
Hammondia_hammondiHH34__185550
Neospora_caninumLIV__265479
Neospora_caninumLIV__265497
Eimeria_brunettiHoughton__259831
Eimeria_tenellaHoughton__309201
Eimeria_falciformisBayerHaberkorn1970__37060
Eimeria_brunettiHoughton__193635
Eimeria_tenellaHoughton__312834
Eimeria_falciformisBayerHaberkorn1970__29881
Eimeria_brunettiHoughton__223053
Eimeria_tenellaHoughton__311430
Eimeria_falciformisBayerHaberkorn1970__31034
Rhytidocystis_sp2__12263
Rhytidocystis_sp2__25755
Rhytidocystis_sp2__999
Rhytidocystis_sp2__17379
Rhytidocystis_sp2__14386
Rhytidocystis_sp2__25403
Rhytidocystis_sp__763
Rhytidocystis_sp__4857
Selenidium_pygospionis__3684
Selenidium_pygospionis__8116
Selenidium_pygospionis__3686
Selenidium_pygospionis__3685
Alphamonas_edax__18356
Monocystis_agilis__750
Vitrella_brassicaformis__15279
Vitrella_brassicaformis__8105
Alphamonas_edax__26866
Alphamonas_edax__18349
Alphamonas_edax__18346
Vitrella_brassicaformis__16272
Platyproteum_sp__18063
Platyproteum_sp__18069
Platyproteum_sp__18066
Platyproteum_sp__18068
Platyproteum_sp__18067
Platyproteum_sp__18064
Platyproteum_sp__18065
Alexandrium_minutum__3012
Amphidinium_carterae__35384
Oxyrrhis_marina__25326
Symbiodinium_microadriaticum__16778
Amoebophrya_ceratii__2408
Colpodella_angusta2__15811
Colpodella_angusta2__15808
Colpodella_angusta1__42735
Colpodella_angusta2__15810
Colpodella_angusta2__15807
Colpodella_angusta2__15809
Chromera_velia__15557
Symbiont_X__21920
Symbiont_X__21921
Blabericola_migrator_2_total__1837
Blabericola_migrator_1_total__13
Gregarina_sp_Pseudo_7__2798
Gregarina_sp_Pseudo_9__5251
Gregarina_sp_Poly_29__482
Gregarina_sp_Poly_1_total__1032
Protomagalhaensia_wolfii__5014
Protomagalhaensia_sp_Gyna_26__680
Protomagalhaensia_sp_Gyna_25__1043
Gregarina_niphandrodes__3411
Gregarina_niphandrodes__3412
Cephaloidophora_communis__5252
Heliospora_caprellae__1256
Gregarina_niphandrodes__3413
Siedleckia_nematoides__27238
Siedleckia_nematoides__53443
Plasmodiophora_brassicae__5182
123481603_Eukaryota_Trichomonadida_Trichomonadidae_Trichomonas_vaginalis_G3_
Trichomonas_vaginalis__31182
Trichomonas_vaginalis__11069
123383069_Eukaryota_Trichomonadida_Trichomonadidae_Trichomonas_vaginalis_G3_
154414900_Eukaryota_Trichomonadida_Trichomonadidae_Trichomonas_vaginalis_G3_
Trichomonas_vaginalis__14186
Platyophrya_macrostoma___Platyphrya_17407_
Aristerostoma_sp___Aristero_46058_
145489759_Eukaryota_Intramacronucleata_Oligohymenophorea_Paramecium_tetraurelia_strain_d42_
Paramecium_tetraurelia___paratetr_109671_
145498654_Eukaryota_Intramacronucleata_Oligohymenophorea_Paramecium_tetraurelia_strain_d42_
118382956_Eukaryota_Intramacronucleata_Oligohymenophorea_Tetrahymena_thermophila_
Tetrahymena_thermophila__13682
Tiarina_fusus___tiarina_150786_
Furgasonia_blochmanni___FurgaBloc_20990_
Chilodonella_uncinata___Chilounci_4114_
Litonotus_sp___Litonotus_4600_
SKscaffold_4539_
Muranothrix_gubernatus___Murano_16889_
Protocruzia_adherens___Protocruzi_19632_
Euplotes_focardii___EupFoca_13696_
403363591_Eukaryota_Intramacronucleata_Spirotrichea_Oxytricha_trifallax_
Oxytricha_trifallax__19372
678316623_Eukaryota_Intramacronucleata_Spirotrichea_Stylonychia_lemnae_
Halteria_grandinella___Halteria_4767_
Strombidium_inclinatum___StromIncl_43941_
Strombidinopsis_acuminatum___StromAcum_90167_
PARAscaffold_53471_
CSSscaffold_3632_
SKscaffold_1621_
Nyctotherus_ovalis___Nycto_6599_
Stentor_coeruleus__11351
Eimeria_falciformisBayerHaberkorn1970__33645
Eimeria_brunettiHoughton__229233
Eimeria_tenellaHoughton__311051
Protostelium_aurantium_var_fungivorum__14648
253743393_Eukaryota_Hexamitidae_Giardiinae_Giardia_intestinalis_ATCC_50581_
559181493_Eukaryota_Hexamitidae_Giardiinae_Giardia_intestinalis_
Giardia_lamblia__644
308157995_Eukaryota_Hexamitidae_Giardiinae_Giardia_lamblia_P15_
Spironucleus_salmonicida__4895
449689538_Eukaryota_Metazoa_Cnidaria_Hydra_vulgaris_
Branchiostoma_floridae__1983
597883182_Eukaryota_Metazoa_Nematoda_Ancylostoma_ceylanicum_
597883181_Eukaryota_Metazoa_Nematoda_Ancylostoma_ceylanicum_
443713232_Eukaryota_Metazoa_Annelida_Capitella_teleta_
Aplysia_californica__9992
Aplysia_californica__8365
Drosophila_melanogaster__2096
Leishmania_major__1354
157865923_Eukaryota_Kinetoplastida_Trypanosomatidae_Leishmania_major_strain_Friedlin_
401417348_Eukaryota_Kinetoplastida_Trypanosomatidae_Leishmania_mexicana_MHOM_GT_2001_U1103_
529497524_Eukaryota_Kinetoplastida_Trypanosomatidae_Strigomonas_galati_
Bodo_saltans__258
Trypanosoma_brucei__259
Platyophrya_macrostoma___Platyphrya_5145_
290997986_Eukaryota_Heterolobosea_Schizopyrenida_Naegleria_gruberi_strain_NEGM_
Naegleria_gruberi__13325
Colpodella_angusta2__23739
545375538_Eukaryota_Viridiplantae_Chlorophyta_Coccomyxa_subellipsoidea_C169_
633913043_Eukaryota_Viridiplantae_Chlorophyta_Helicosporidium_sp__ATCC_50920_
552827493_Eukaryota_Viridiplantae_Chlorophyta_Chlorella_variabilis_
302831908_Eukaryota_Viridiplantae_Chlorophyta_Volvox_carteri_f__nagariensis_
Volvox_carteri__1899
Chlamydomonas_reinhardtii__8979
674891275_Eukaryota_Viridiplantae_Streptophyta_Brassica_napus_
685348814_Eukaryota_Viridiplantae_Streptophyta_Brassica_rapa_
Arabidopsis_thaliana__28610
Arabidopsis_thaliana__459
661882999_Eukaryota_Viridiplantae_Streptophyta_Coffea_canephora_
Ectocarpus_siliculosus__298
298714844_Eukaryota_Phaeophyceae_Ectocarpales_Ectocarpus_siliculosus_
585105611_Eukaryota_Eustigmatophyceae_Eustigmatales_Nannochloropsis_gaditana_
Digyalum_oweni1__13412
167521427_Eukaryota_Choanoflagellida_Codonosigidae_Monosiga_brevicollis_MX1_
514689446_Eukaryota_Choanoflagellida_Salpingoecidae_Salpingoeca_rosetta_
219115595_Eukaryota_Bacillariophyta_Bacillariophyceae_Phaeodactylum_tricornutum_CCAP_1055_1_
Phaeodactylum_tricornutum__2436
224004378_Eukaryota_Bacillariophyta_Coscinodiscophyceae_Thalassiosira_pseudonana_CCMP1335_
Thalassiosira_pseudonana__9857
397566398_Eukaryota_Bacillariophyta_Coscinodiscophyceae_Thalassiosira_oceanica_
Pygsuia_biforma__10302
Pygsuia_biforma__12573
Siedleckia_nematoides__60707
Paulinella_chromatophora__25987
544213676_Eukaryota_Bangiophyceae_Cyanidiales_Cyanidioschyzon_merolae_strain_10D_
Cyanidioschyzon_merolae__2202
545713544_Eukaryota_Bangiophyceae_Cyanidiales_Galdieria_sulphuraria_
Galdieria_sulphuraria__6526
Monocercomonoides_exilis__9902
Coprinus_cinereus__7412
Digyalum_oweni1__12454
Siedleckia_nematoides__6300
406166635_Eukaryota_Fungi_Dikarya_Puccinia_graminis_f__sp__tritici_CRL_75367003_
Ustilago_maydis__5065
549053973_Eukaryota_Fungi_Dikarya_Pyronema_omphalodes_CBS_100304_
156838379_Eukaryota_Fungi_Dikarya_Vanderwaltozyma_polyspora_DSM_70294_
Saccharomyces_cerevisiae__3474
213408985_Eukaryota_Fungi_Dikarya_Schizosaccharomyces_japonicus_yFS275_
Digyalum_oweni1__17918
575482708_Eukaryota_Fungi_Chytridiomycota_Batrachochytrium_dendrobatidis_JAM81_
671690974_Eukaryota_Fungi_Mucoromycotina_Absidia_idahoensis_var__thermophila_
595482094_Eukaryota_Fungi_Glomeromycota_Rhizophagus_irregularis_DAOM_197198w_
672825487_Eukaryota_Fungi_Mortierellomycotina_Mortierella_verticillata_NRRL_6337_
672822367_Eukaryota_Fungi_Mortierellomycotina_Mortierella_verticillata_NRRL_6337_
511007431_Eukaryota_Fungi_Mucoromycotina_Mucor_circinelloides_f__circinelloides_1006PhL_
514484392_Eukaryota_Ichthyosporea_Capsaspora_Capsaspora_owczarzaki_ATCC_30864_
470296671_Eukaryota_Ichthyosporea_Capsaspora_Capsaspora_owczarzaki_ATCC_30864_
167519951_Eukaryota_Choanoflagellida_Codonosigidae_Monosiga_brevicollis_MX1_
Goniomonas_avonlea__12318
Guillardia_theta__15084
551660760_Eukaryota_Cryptophyta_Pyrenomonadales_Guillardia_theta_CCMP2712_
Guillardia_theta__20058
551670939_Eukaryota_Cryptophyta_Pyrenomonadales_Guillardia_theta_CCMP2712_
Protostelium_aurantium_var_fungivorum__16219
Sappinia_pedata__10971
281207142_Eukaryota_Dictyosteliida_Polysphondylium_Polysphondylium_pallidum_PN500_
Dictyostelium_discoideum__4374
470526110_Eukaryota_Longamoebia_Acanthamoeba_Acanthamoeba_castellanii_str__Neff_
Acanthamoeba_castellanii__13816
Acanthamoeba_castellanii__9368
470477818_Eukaryota_Longamoebia_Acanthamoeba_Acanthamoeba_castellanii_str__Neff_
567986915_Eukaryota_Peronosporales_Phytophthora_Phytophthora_parasitica_
0.4

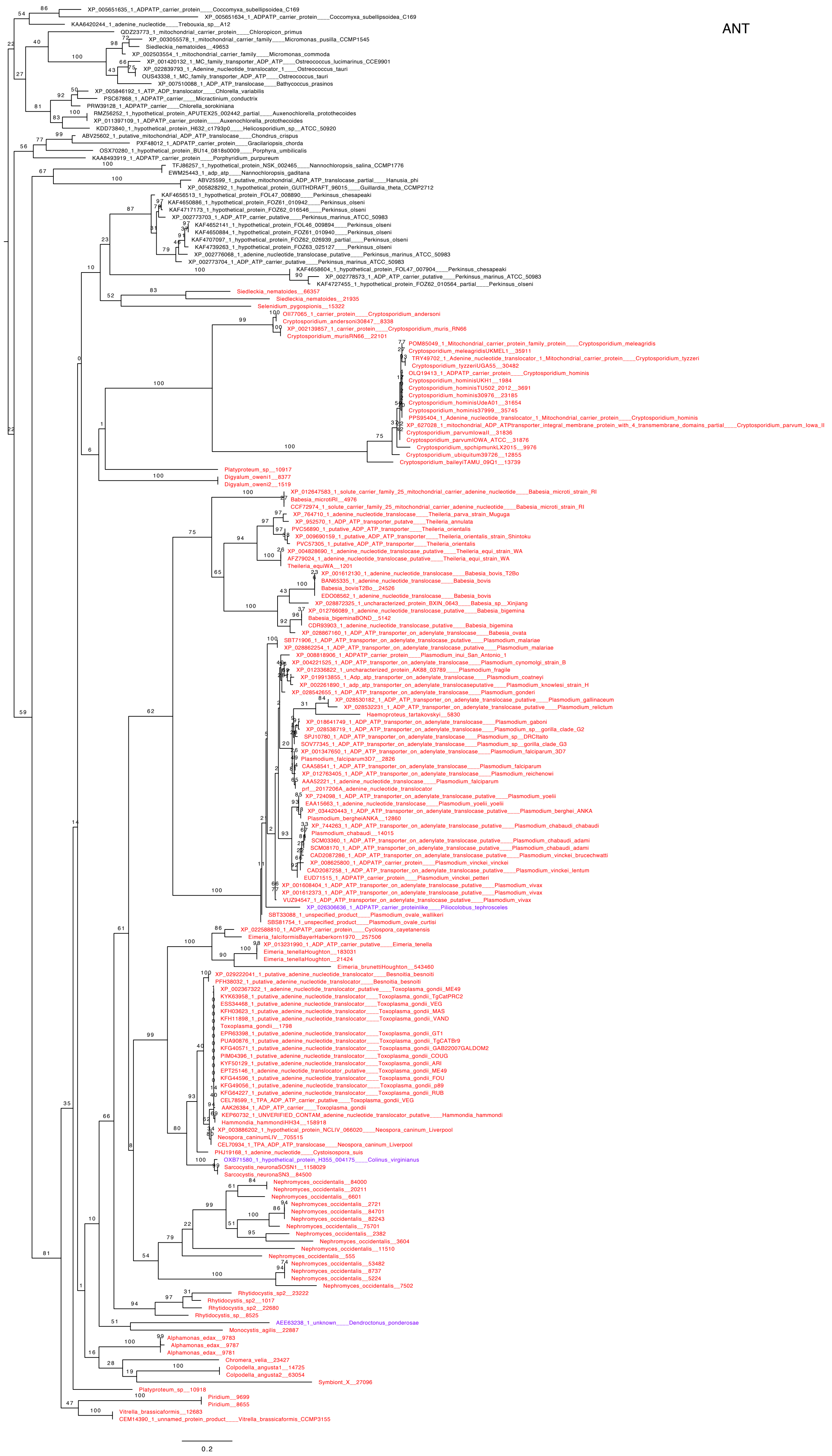
ANT
XP_005651635_1_ADPATP_carrier_protein____Coccomyxa_subellipsoidea_C169
XP_005651634_1_ADPATP_carrier_protein____Coccomyxa_subellipsoidea_C169
KAA6420244_1_adenine_nucleotide____Trebouxia_sp__A12
QDZ23773_1_mitochondrial_carrier_protein____Chloropicon_primus
XP_003055578_1_mitochondrial_carrier_family____Micromonas_pusilla_CCMP1545
Siedleckia_nematoides__49653
XP_002503554_1_mitochondrial_carrier_family____Micromonas_commoda
XP_001420132_1_MC_family_transporter_ADP_ATP____Ostreococcus_lucimarinus_CCE9901
XP_022839793_1_Adenine_nucleotide_translocator_1____Ostreococcus_tauri
OUS43338_1_MC_family_transporter_ADP_ATP____Ostreococcus_tauri
XP_007510088_1_ADP_ATP_translocase____Bathycoccus_prasinos
XP_005846192_1_ATP_ADP_translocator____Chlorella_variabilis
PSC67868_1_ADPATP_carrier____Micractinium_conductrix
PRW39128_1_ADPATP_carrier____Chlorella_sorokiniana
RMZ56252_1_hypothetical_protein_APUTEX25_002442_partial____Auxenochlorella_protothecoides
XP_011397109_1_ADPATP_carrier_protein____Auxenochlorella_protothecoides
KDD73840_1_hypothetical_protein_H632_c1793p0____Helicosporidium_sp__ATCC_50920
ABV25602_1_putative_mitochondrial_ADP_ATP_translocase____Chondrus_crispus
PXF48012_1_ADPATP_carrier_protein____Gracilariopsis_chorda
OSX70280_1_hypothetical_protein_BU14_0818s0009____Porphyra_umbilicalis
KAA8493919_1_ADPATP_carrier_protein____Porphyridium_purpureum
TFJ86257_1_hypothetical_protein_NSK_002465____Nannochloropsis_salina_CCMP1776
EWM25443_1_adp_atp____Nannochloropsis_gaditana
ABV25599_1_putative_mitochondrial_ADP_ATP_translocase_partial____Hanusia_phi
XP_005828292_1_hypothetical_protein_GUITHDRAFT_96015____Guillardia_theta_CCMP2712
KAF4656513_1_hypothetical_protein_FOL47_008890____Perkinsus_chesapeaki
KAF4650886_1_hypothetical_protein_FOZ61_010942____Perkinsus_olseni
KAF4717173_1_hypothetical_protein_FOZ62_016546____Perkinsus_olseni
XP_002773703_1_ADP_ATP_carrier_putative____Perkinsus_marinus_ATCC_50983
KAF4652141_1_hypothetical_protein_FOL46_009894____Perkinsus_olseni
KAF4650884_1_hypothetical_protein_FOZ61_010940____Perkinsus_olseni
KAF4707097_1_hypothetical_protein_FOZ62_026939_partial____Perkinsus_olseni
KAF4739263_1_hypothetical_protein_FOZ63_025127____Perkinsus_olseni
XP_002776068_1_adenine_nucleotide_translocase_putative____Perkinsus_marinus_ATCC_50983
XP_002773704_1_ADP_ATP_carrier_putative____Perkinsus_marinus_ATCC_50983
KAF4658604_1_hypothetical_protein_FOL47_007904____Perkinsus_chesapeaki
XP_002778573_1_ADP_ATP_carrier_putative____Perkinsus_marinus_ATCC_50983
KAF4727455_1_hypothetical_protein_FOZ62_010564_partial____Perkinsus_olseni
Siedleckia_nematoides__66357
Siedleckia_nematoides__21935
Selenidium_pygospionis__15322
OII77065_1_carrier_protein____Cryptosporidium_andersoni
Cryptosporidium_andersoni30847__8338
XP_002139857_1_carrier_protein____Cryptosporidium_muris_RN66
Cryptosporidium_murisRN66__22101
POM85049_1_Mitochondrial_carrier_protein_family_protein____Cryptosporidium_meleagridis
Cryptosporidium_meleagridisUKMEL1__35911
TRY49702_1_Adenine_nucleotide_translocator_1_Mitochondrial_carrier_protein____Cryptosporidium_tyzzeri
Cryptosporidium_tyzzeriUGA55__30482
OLQ19413_1_ADPATP_carrier_protein____Cryptosporidium_hominis
Cryptosporidium_hominisUKH1__1984
Cryptosporidium_hominisTU502_2012__3691
Cryptosporidium_hominis30976__23185
Cryptosporidium_hominisUdeA01__31654
Cryptosporidium_hominis37999__35745
PPS95404_1_Adenine_nucleotide_translocator_1_Mitochondrial_carrier_protein____Cryptosporidium_hominis
XP_627028_1_mitochondrial_ADP_ATPtransporter_integral_membrane_protein_with_4_transmembrane_domains_partial____Cryptosporidium_parvum_Iowa_II
Cryptosporidium_parvumIowaII__31836
Cryptosporidium_parvumIOWA_ATCC__31876
Cryptosporidium_spchipmunkLX2015__9976
Cryptosporidium_ubiquitum39726__12855
Cryptosporidium_baileyiTAMU_09Q1__13739
Platyproteum_sp__10917
Digyalum_oweni1__8377
Digyalum_oweni2__1519
XP_012647583_1_solute_carrier_family_25_mitochondrial_carrier_adenine_nucleotide____Babesia_microti_strain_RI
Babesia_microtiRI__4976
CCF72974_1_solute_carrier_family_25_mitochondrial_carrier_adenine_nucleotide____Babesia_microti_strain_RI
XP_764710_1_adenine_nucleotide_translocase____Theileria_parva_strain_Muguga
XP_952570_1_ADP_ATP_transporter_putatve____Theileria_annulata
PVC56890_1_putative_ADP_ATP_transporter____Theileria_orientalis
XP_009690159_1_putative_ADP_ATP_transporter____Theileria_orientalis_strain_Shintoku
PVC57305_1_putative_ADP_ATP_transporter____Theileria_orientalis
XP_004828690_1_adenine_nucleotide_translocase_putative____Theileria_equi_strain_WA
AFZ79024_1_adenine_nucleotide_translocase_putative____Theileria_equi_strain_WA
Theileria_equiWA__1201
XP_001612130_1_adenine_nucleotide_translocase____Babesia_bovis_T2Bo
BAN65335_1_adenine_nucleotide_translocase____Babesia_bovis
Babesia_bovisT2Bo__24526
EDO08562_1_adenine_nucleotide_translocase____Babesia_bovis
XP_028872325_1_uncharacterized_protein_BXIN_0643____Babesia_sp__Xinjiang
XP_012766089_1_adenine_nucleotide_translocase_putative____Babesia_bigemina
Babesia_bigeminaBOND__5142
CDR93903_1_adenine_nucleotide_translocase_putative____Babesia_bigemina
XP_028867160_1_ADP_ATP_transporter_on_adenylate_translocase____Babesia_ovata
SBT71906_1_ADP_ATP_transporter_on_adenylate_translocase_putative____Plasmodium_malariae
XP_028862254_1_ADP_ATP_transporter_on_adenylate_translocase_putative____Plasmodium_malariae
XP_008818906_1_ADPATP_carrier_protein____Plasmodium_inui_San_Antonio_1
XP_004221525_1_ADP_ATP_transporter_on_adenylate_translocase____Plasmodium_cynomolgi_strain_B
XP_012336822_1_uncharacterized_protein_AK88_03789____Plasmodium_fragile
XP_019913855_1_Adp_atp_transporter_on_adenylate_translocase____Plasmodium_coatneyi
XP_002261890_1_adp_atp_transporter_on_adenylate_translocaseputative____Plasmodium_knowlesi_strain_H
XP_028542655_1_ADP_ATP_transporter_on_adenylate_translocase____Plasmodium_gonderi
XP_028530182_1_ADP_ATP_transporter_on_adenylate_translocase_putative____Plasmodium_gallinaceum
XP_028532231_1_ADP_ATP_transporter_on_adenylate_translocase_putative____Plasmodium_relictum
Haemoproteus_tartakovskyi__5830
XP_018641749_1_ADP_ATP_transporter_on_adenylate_translocase____Plasmodium_gaboni
XP_028538719_1_ADP_ATP_transporter_on_adenylate_translocase____Plasmodium_sp__gorilla_clade_G2
SPJ10780_1_ADP_ATP_transporter_on_adenylate_translocase____Plasmodium_sp__DRC|taito
SOV77345_1_ADP_ATP_transporter_on_adenylate_translocase____Plasmodium_sp__gorilla_clade_G3
XP_001347650_1_ADP_ATP_transporter_on_adenylate_translocase____Plasmodium_falciparum_3D7
Plasmodium_falciparum3D7__2826
CAA58541_1_ADP_ATP_transporter_on_adenylate_translocase____Plasmodium_falciparum
XP_012763405_1_ADP_ATP_transporter_on_adenylate_translocase____Plasmodium_reichenowi
AAA52221_1_adenine_nucleotide_translocase____Plasmodium_falciparum
prf__2017206A_adenine_nucleotide_translocator
XP_724098_1_ADP_ATP_transporter_on_adenylate_translocase_putative____Plasmodium_yoelii
EAA15663_1_adenine_nucleotide_translocase____Plasmodium_yoelii_yoelii
XP_034420443_1_ADP_ATP_transporter_on_adenylate_translocase_putative____Plasmodium_berghei_ANKA
Plasmodium_bergheiANKA__12860
XP_744263_1_ADP_ATP_transporter_on_adenylate_translocase_putative____Plasmodium_chabaudi_chabaudi
Plasmodium_chabaudi__14015
SCM03360_1_ADP_ATP_transporter_on_adenylate_translocase_putative____Plasmodium_chabaudi_adami
SCM08170_1_ADP_ATP_transporter_on_adenylate_translocase_putative____Plasmodium_chabaudi_adami
CAD2087286_1_ADP_ATP_transporter_on_adenylate_translocase_putative____Plasmodium_vinckei_brucechwatti
XP_008625800_1_ADPATP_carrier_protein____Plasmodium_vinckei_vinckei
CAD2087258_1_ADP_ATP_transporter_on_adenylate_translocase_putative____Plasmodium_vinckei_lentum
EUD71515_1_ADPATP_carrier_protein____Plasmodium_vinckei_petteri
XP_001608404_1_ADP_ATP_transporter_on_adenylate_translocase_putative____Plasmodium_vivax
XP_001612373_1_ADP_ATP_transporter_on_adenylate_translocase_putative____Plasmodium_vivax
VUZ94547_1_ADP_ATP_transporter_on_adenylate_translocase_putative____Plasmodium_vivax
XP_026306636_1_ADPATP_carrier_proteinlike____Piliocolobus_tephrosceles
SBT33088_1_unspecified_product____Plasmodium_ovale_wallikeri
SBS81754_1_unspecified_product____Plasmodium_ovale_curtisi
XP_022588810_1_ADPATP_carrier_protein____Cyclospora_cayetanensis
Eimeria_falciformisBayerHaberkorn1970__257506
XP_013231990_1_ADP_ATP_carrier_putative____Eimeria_tenella
Eimeria_tenellaHoughton__183031
Eimeria_tenellaHoughton__21424
Eimeria_brunettiHoughton__543460
XP_029222041_1_putative_adenine_nucleotide_translocator____Besnoitia_besnoiti
PFH38032_1_putative_adenine_nucleotide_translocator____Besnoitia_besnoiti
XP_002367322_1_adenine_nucleotide_translocator_putative____Toxoplasma_gondii_ME49
KYK63958_1_putative_adenine_nucleotide_translocator____Toxoplasma_gondii_TgCatPRC2
ESS34468_1_putative_adenine_nucleotide_translocator____Toxoplasma_gondii_VEG
KFH03623_1_putative_adenine_nucleotide_translocator____Toxoplasma_gondii_MAS
KFH11898_1_putative_adenine_nucleotide_translocator____Toxoplasma_gondii_VAND
Toxoplasma_gondii__1798
EPR63398_1_putative_adenine_nucleotide_translocator____Toxoplasma_gondii_GT1
PUA90876_1_putative_adenine_nucleotide_translocator____Toxoplasma_gondii_TgCATBr9
KFG40571_1_putative_adenine_nucleotide_translocator____Toxoplasma_gondii_GAB22007GALDOM2
PIM04396_1_putative_adenine_nucleotide_translocator____Toxoplasma_gondii_COUG
KYF50129_1_putative_adenine_nucleotide_translocator____Toxoplasma_gondii_ARI
EPT25146_1_adenine_nucleotide_translocator_putative____Toxoplasma_gondii_ME49
KFG44596_1_putative_adenine_nucleotide_translocator____Toxoplasma_gondii_FOU
KFG49056_1_putative_adenine_nucleotide_translocator____Toxoplasma_gondii_p89
KFG64227_1_putative_adenine_nucleotide_translocator____Toxoplasma_gondii_RUB
CEL78599_1_TPA_ADP_ATP_carrier_putative____Toxoplasma_gondii_VEG
AAK26384_1_ADP_ATP_carrier____Toxoplasma_gondii
KEP60732_1_UNVERIFIED_CONTAM_adenine_nucleotide_translocator_putative____Hammondia_hammondi
Hammondia_hammondiHH34__158918
XP_003886202_1_hypothetical_protein_NCLIV_066020____Neospora_caninum_Liverpool
Neospora_caninumLIV__705515
CEL70934_1_TPA_ADP_ATP_translocase____Neospora_caninum_Liverpool
PHJ19168_1_adenine_nucleotide____Cystoisospora_suis
OXB71580_1_hypothetical_protein_H355_004175____Colinus_virginianus
Sarcocystis_neuronaSOSN1__1158029
Sarcocystis_neuronaSN3__84500
Nephromyces_occidentalis__84000
Nephromyces_occidentalis__20211
Nephromyces_occidentalis__6601
Nephromyces_occidentalis__2721
Nephromyces_occidentalis__84701
Nephromyces_occidentalis__82243
Nephromyces_occidentalis__75701
Nephromyces_occidentalis__2382
Nephromyces_occidentalis__3604
Nephromyces_occidentalis__11510
Nephromyces_occidentalis__555
Nephromyces_occidentalis__53482
Nephromyces_occidentalis__8737
Nephromyces_occidentalis__5224
Nephromyces_occidentalis__7502
Rhytidocystis_sp2__23222
Rhytidocystis_sp2__1017
Rhytidocystis_sp2__22680
Rhytidocystis_sp__8525
AEE63238_1_unknown____Dendroctonus_ponderosae
Monocystis_agilis__22887
Alphamonas_edax__9783
Alphamonas_edax__9787
Alphamonas_edax__9781
Chromera_velia__23427
Colpodella_angusta1__14725
Colpodella_angusta2__63054
Symbiont_X__27096
Platyproteum_sp__10918
Piridium__9699
Piridium__8655
Vitrella_brassicaformis__12683
CEM14390_1_unnamed_protein_product____Vitrella_brassicaformis_CCMP3155
0.2

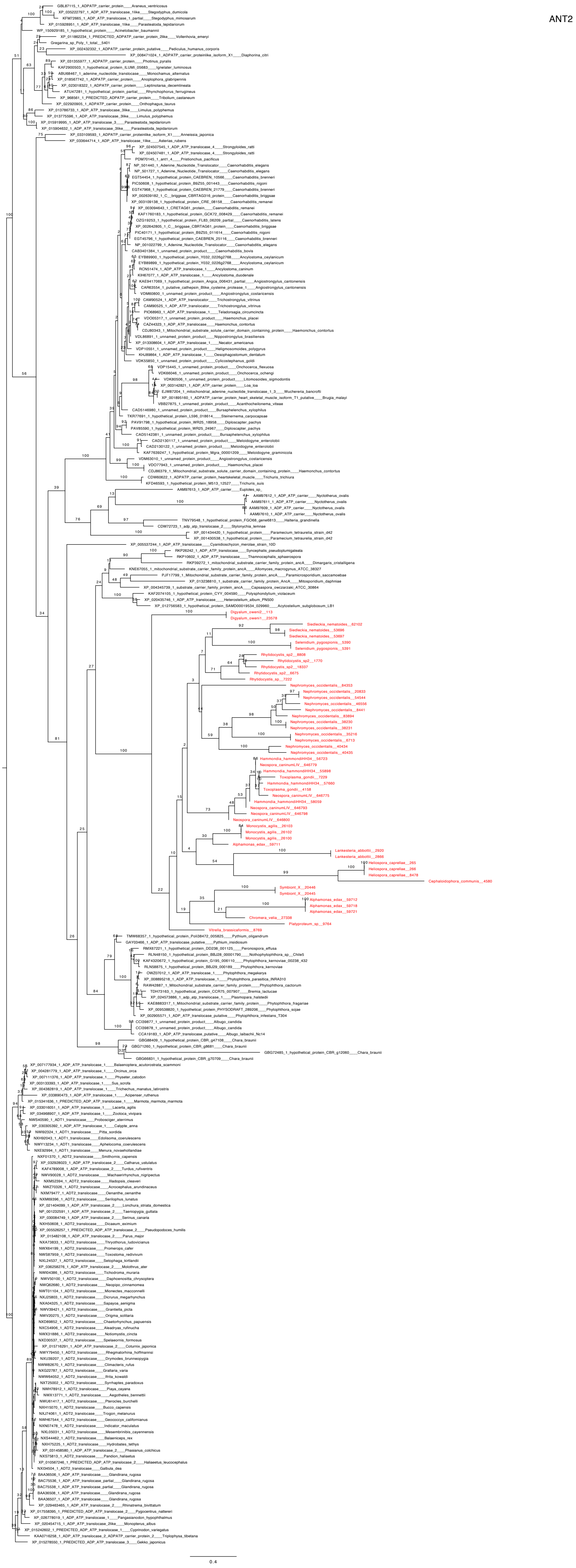
ANT2
0.4

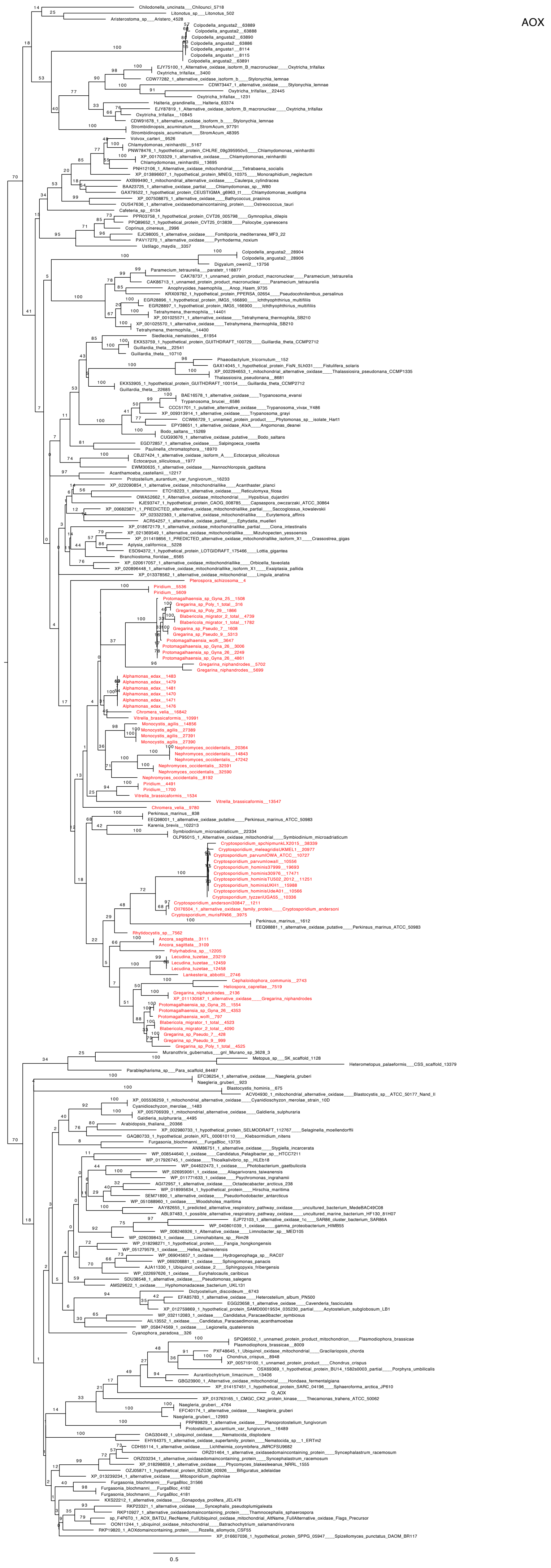
AOX
0.5

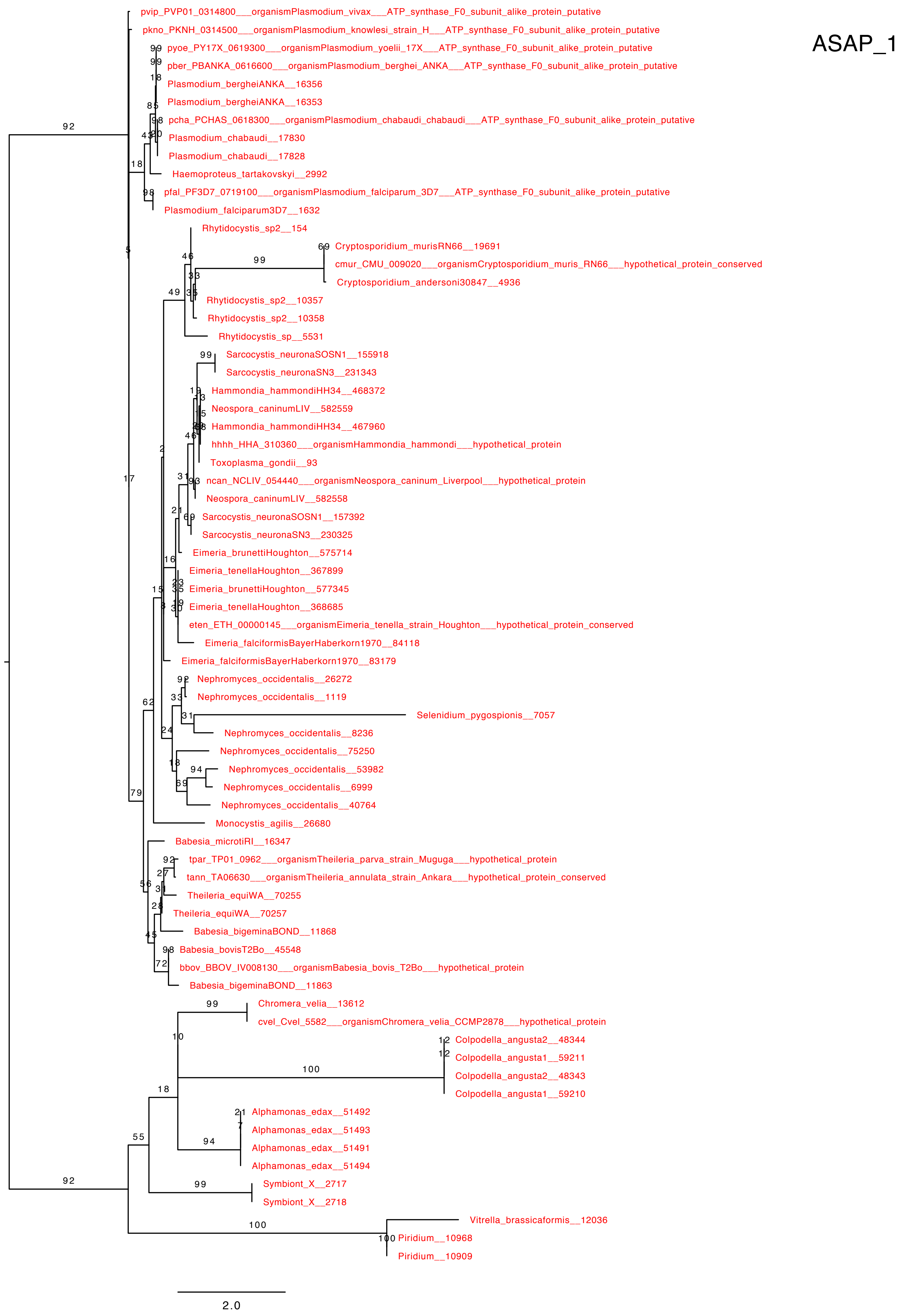

ASAP_1
pvip_PVP01_0314800___organismPlasmodium_vivax___ATP_synthase_F0_subunit_alike_protein_putative
pkno_PKNH_0314500___organismPlasmodium_knowlesi_strain_H___ATP_synthase_F0_subunit_alike_protein_putative
pyoe_PY17X_0619300___organismPlasmodium_yoelii_17X___ATP_synthase_F0_subunit_alike_protein_putative
pber_PBANKA_0616600___organismPlasmodium_berghei_ANKA___ATP_synthase_F0_subunit_alike_protein_putative
Plasmodium_bergheiANKA__16356
Plasmodium_bergheiANKA__16353
pcha_PCHAS_0618300___organismPlasmodium_chabaudi_chabaudi___ATP_synthase_F0_subunit_alike_protein_putative
Plasmodium_chabaudi__17830
Plasmodium_chabaudi__17828
Haemoproteus_tartakovskyi__2992
pfal_PF3D7_0719100___organismPlasmodium_falciparum_3D7___ATP_synthase_F0_subunit_alike_protein_putative
Plasmodium_falciparum3D7__1632
Rhytidocystis_sp2__154
Cryptosporidium_murisRN66__19691
cmur_CMU_009020___organismCryptosporidium_muris_RN66___hypothetical_protein_conserved
Cryptosporidium_andersoni30847__4936
Rhytidocystis_sp2__10357
Rhytidocystis_sp2__10358
Rhytidocystis_sp__5531
Sarcocystis_neuronaSOSN1__155918
Sarcocystis_neuronaSN3__231343
Hammondia_hammondiHH34__468372
Neospora_caninumLIV__582559
Hammondia_hammondiHH34__467960
hhhh_HHA_310360___organismHammondia_hammondi___hypothetical_protein
Toxoplasma_gondii__93
ncan_NCLIV_054440___organismNeospora_caninum_Liverpool___hypothetical_protein
Neospora_caninumLIV__582558
Sarcocystis_neuronaSOSN1__157392
Sarcocystis_neuronaSN3__230325
Eimeria_brunettiHoughton__575714
Eimeria_tenellaHoughton__367899
Eimeria_brunettiHoughton__577345
Eimeria_tenellaHoughton__368685
eten_ETH_00000145___organismEimeria_tenella_strain_Houghton___hypothetical_protein_conserved
Eimeria_falciformisBayerHaberkorn1970__84118
Eimeria_falciformisBayerHaberkorn1970__83179
Nephromyces_occidentalis__26272
Nephromyces_occidentalis__1119
Selenidium_pygospionis__7057
Nephromyces_occidentalis__8236
Nephromyces_occidentalis__75250
Nephromyces_occidentalis__53982
Nephromyces_occidentalis__6999
Nephromyces_occidentalis__40764
Monocystis_agilis__26680
Babesia_microtiRI__16347
tpar_TP01_0962___organismTheileria_parva_strain_Muguga___hypothetical_protein
tann_TA06630___organismTheileria_annulata_strain_Ankara___hypothetical_protein_conserved
Theileria_equiWA__70255
Theileria_equiWA__70257
Babesia_bigeminaBOND__11868
Babesia_bovisT2Bo__45548
bbov_BBOV_IV008130___organismBabesia_bovis_T2Bo___hypothetical_protein
Babesia_bigeminaBOND__11863
Chromera_velia__13612
cvel_Cvel_5582___organismChromera_velia_CCMP2878___hypothetical_protein
Colpodella_angusta2__48344
Colpodella_angusta1__59211
Colpodella_angusta2__48343
Colpodella_angusta1__59210
Alphamonas_edax__51492
Alphamonas_edax__51493
Alphamonas_edax__51491
Alphamonas_edax__51494
Symbiont_X__2717
Symbiont_X__2718
Vitrella_brassicaformis__12036
Piridium__10968
Piridium__10909
2.0

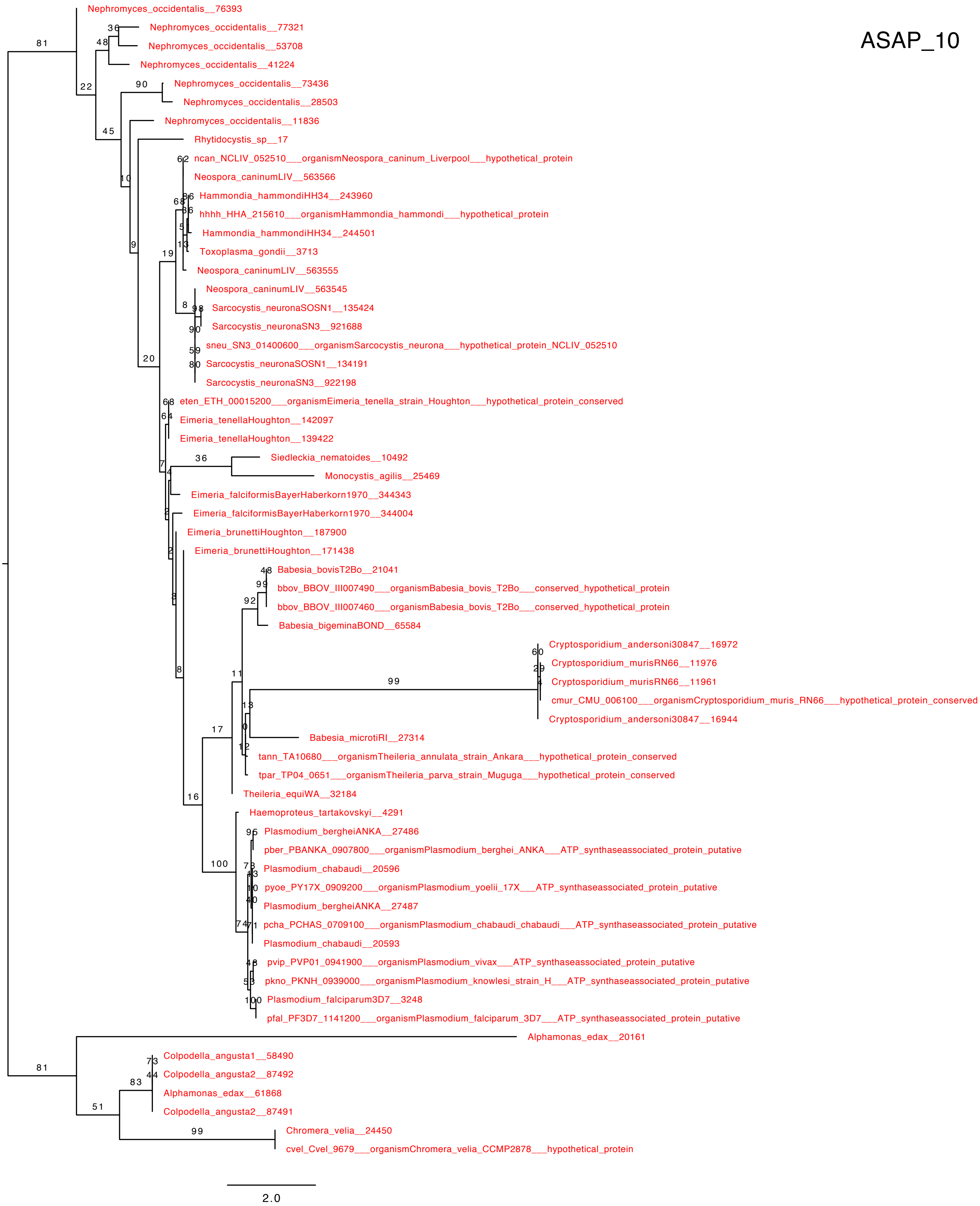

ASAP_10
Nephromyces_occidentalis__76393
Nephromyces_occidentalis__77321
Nephromyces_occidentalis__53708
Nephromyces_occidentalis__41224
Nephromyces_occidentalis__73436
Nephromyces_occidentalis__28503
Nephromyces_occidentalis__11836
Rhytidocystis_sp__17
ncan_NCLIV_052510___organismNeospora_caninum_Liverpool___hypothetical_protein
Neospora_caninumLIV__563566
Hammondia_hammondiHH34__243960
hhhh_HHA_215610___organismHammondia_hammondi___hypothetical_protein
Hammondia_hammondiHH34__244501
Toxoplasma_gondii__3713
Neospora_caninumLIV__563555
Neospora_caninumLIV__563545
Sarcocystis_neuronaSOSN1__135424
Sarcocystis_neuronaSN3__921688
sneu_SN3_01400600___organismSarcocystis_neurona___hypothetical_protein_NCLIV_052510
Sarcocystis_neuronaSOSN1__134191
Sarcocystis_neuronaSN3__922198
eten_ETH_00015200___organismEimeria_tenella_strain_Houghton___hypothetical_protein_conserved
Eimeria_tenellaHoughton__142097
Eimeria_tenellaHoughton__139422
Siedleckia_nematoides__10492
Monocystis_agilis__25469
Eimeria_falciformisBayerHaberkorn1970__344343
Eimeria_falciformisBayerHaberkorn1970__344004
Eimeria_brunettiHoughton__187900
Eimeria_brunettiHoughton__171438
Babesia_bovisT2Bo__21041
bbov_BBOV_III007490___organismBabesia_bovis_T2Bo___conserved_hypothetical_protein
bbov_BBOV_III007460___organismBabesia_bovis_T2Bo___conserved_hypothetical_protein
Babesia_bigeminaBOND__65584
Cryptosporidium_andersoni30847__16972
Cryptosporidium_murisRN66__11976
Cryptosporidium_murisRN66__11961
cmur_CMU_006100___organismCryptosporidium_muris_RN66___hypothetical_protein_conserved
Cryptosporidium_andersoni30847__16944
Babesia_microtiRI__27314
tann_TA10680___organismTheileria_annulata_strain_Ankara___hypothetical_protein_conserved
tpar_TP04_0651___organismTheileria_parva_strain_Muguga___hypothetical_protein_conserved
Theileria_equiWA__32184
Haemoproteus_tartakovskyi__4291
Plasmodium_bergheiANKA__27486
pber_PBANKA_0907800___organismPlasmodium_berghei_ANKA___ATP_synthaseassociated_protein_putative
Plasmodium_chabaudi__20596
pyoe_PY17X_0909200___organismPlasmodium_yoelii_17X___ATP_synthaseassociated_protein_putative
Plasmodium_bergheiANKA__27487
pcha_PCHAS_0709100___organismPlasmodium_chabaudi_chabaudi___ATP_synthaseassociated_protein_putative
Plasmodium_chabaudi__20593
pvip_PVP01_0941900___organismPlasmodium_vivax___ATP_synthaseassociated_protein_putative
pkno_PKNH_0939000___organismPlasmodium_knowlesi_strain_H___ATP_synthaseassociated_protein_putative
Plasmodium_falciparum3D7__3248
pfal_PF3D7_1141200___organismPlasmodium_falciparum_3D7___ATP_synthaseassociated_protein_putative
Alphamonas_edax__20161
Colpodella_angusta1__58490
Colpodella_angusta2__87492
Alphamonas_edax__61868
Colpodella_angusta2__87491
Chromera_velia__24450
cvel_Cvel_9679___organismChromera_velia_CCMP2878___hypothetical_protein
2.0

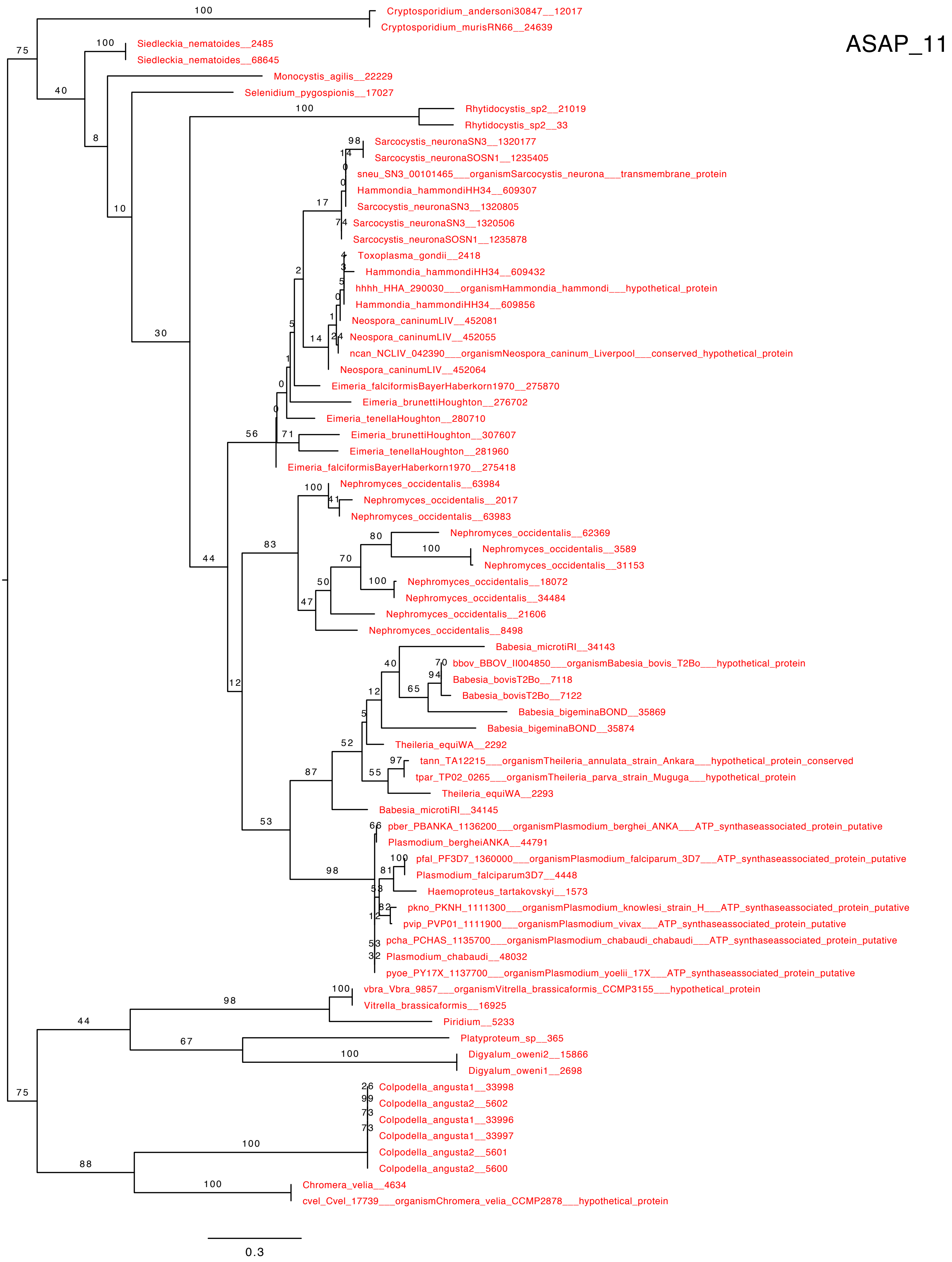
ASAP_11
Cryptosporidium_andersoni30847__12017
Cryptosporidium_murisRN66__24639
Siedleckia_nematoides__2485
Siedleckia_nematoides__68645
Monocystis_agilis__22229
Selenidium_pygospionis__17027
Rhytidocystis_sp2__21019
Rhytidocystis_sp2__33
Sarcocystis_neuronaSN3__1320177
Sarcocystis_neuronaSOSN1__1235405
sneu_SN3_00101465___organismSarcocystis_neurona___transmembrane_protein
Hammondia_hammondiHH34__609307
Sarcocystis_neuronaSN3__1320805
Sarcocystis_neuronaSN3__1320506
Sarcocystis_neuronaSOSN1__1235878
Toxoplasma_gondii__2418
Hammondia_hammondiHH34__609432
hhhh_HHA_290030___organismHammondia_hammondi___hypothetical_protein
Hammondia_hammondiHH34__609856
Neospora_caninumLIV__452081
Neospora_caninumLIV__452055
ncan_NCLIV_042390___organismNeospora_caninum_Liverpool___conserved_hypothetical_protein
Neospora_caninumLIV__452064
Eimeria_falciformisBayerHaberkorn1970__275870
Eimeria_brunettiHoughton__276702
Eimeria_tenellaHoughton__280710
Eimeria_brunettiHoughton__307607
Eimeria_tenellaHoughton__281960
Eimeria_falciformisBayerHaberkorn1970__275418
Nephromyces_occidentalis__63984
Nephromyces_occidentalis__2017
Nephromyces_occidentalis__63983
Nephromyces_occidentalis__62369
Nephromyces_occidentalis__3589
Nephromyces_occidentalis__31153
Nephromyces_occidentalis__18072
Nephromyces_occidentalis__34484
Nephromyces_occidentalis__21606
Nephromyces_occidentalis__8498
Babesia_microtiRI__34143
bbov_BBOV_II004850___organismBabesia_bovis_T2Bo___hypothetical_protein
Babesia_bovisT2Bo__7118
Babesia_bovisT2Bo__7122
Babesia_bigeminaBOND__35869
Babesia_bigeminaBOND__35874
Theileria_equiWA__2292
tann_TA12215___organismTheileria_annulata_strain_Ankara___hypothetical_protein_conserved
tpar_TP02_0265___organismTheileria_parva_strain_Muguga___hypothetical_protein
Theileria_equiWA__2293
Babesia_microtiRI__34145
pber_PBANKA_1136200___organismPlasmodium_berghei_ANKA___ATP_synthaseassociated_protein_putative
Plasmodium_bergheiANKA__44791
pfal_PF3D7_1360000___organismPlasmodium_falciparum_3D7___ATP_synthaseassociated_protein_putative
Plasmodium_falciparum3D7__4448
Haemoproteus_tartakovskyi__1573
pkno_PKNH_1111300___organismPlasmodium_knowlesi_strain_H___ATP_synthaseassociated_protein_putative
pvip_PVP01_1111900___organismPlasmodium_vivax___ATP_synthaseassociated_protein_putative
pcha_PCHAS_1135700___organismPlasmodium_chabaudi_chabaudi___ATP_synthaseassociated_protein_putative
Plasmodium_chabaudi__48032
pyoe_PY17X_1137700___organismPlasmodium_yoelii_17X___ATP_synthaseassociated_protein_putative
vbra_Vbra_9857___organismVitrella_brassicaformis_CCMP3155___hypothetical_protein
Vitrella_brassicaformis__16925
Piridium__5233
Platyproteum_sp__365
Digyalum_oweni2__15866
Digyalum_oweni1__2698
Colpodella_angusta1__33998
Colpodella_angusta2__5602
Colpodella_angusta1__33996
Colpodella_angusta1__33997
Colpodella_angusta2__5601
Colpodella_angusta2__5600
Chromera_velia__4634
cvel_Cvel_17739___organismChromera_velia_CCMP2878___hypothetical_protein
0.3

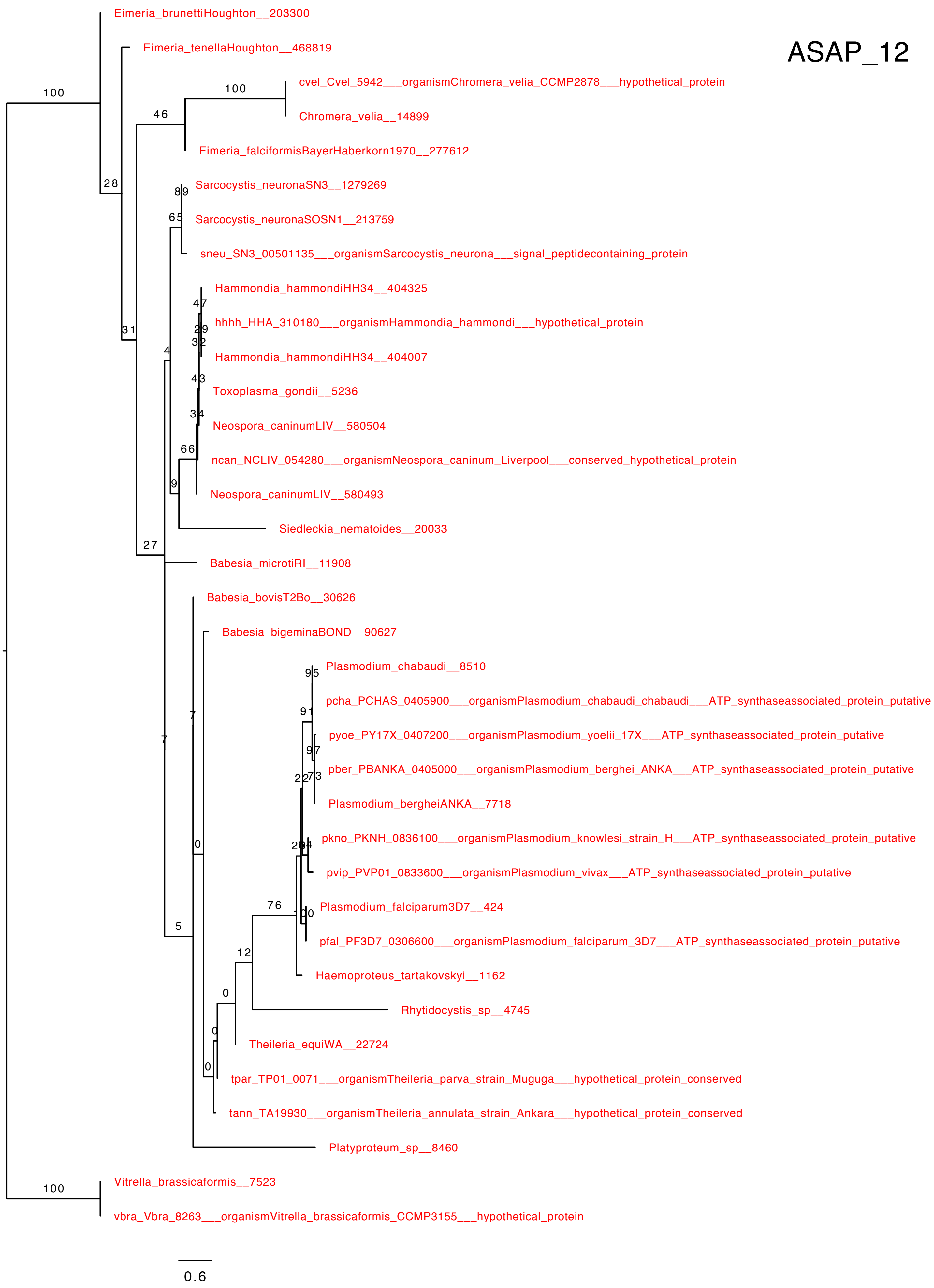

ASAP_12
Eimeria_brunettiHoughton__203300
Eimeria_tenellaHoughton__468819
cvel_Cvel_5942___organismChromera_velia_CCMP2878___hypothetical_protein
Chromera_velia__14899
Eimeria_falciformisBayerHaberkorn1970__277612
Sarcocystis_neuronaSN3__1279269
Sarcocystis_neuronaSOSN1__213759
sneu_SN3_00501135___organismSarcocystis_neurona___signal_peptidecontaining_protein
Hammondia_hammondiHH34__404325
hhhh_HHA_310180___organismHammondia_hammondi___hypothetical_protein
Hammondia_hammondiHH34__404007
Toxoplasma_gondii__5236
Neospora_caninumLIV__580504
ncan_NCLIV_054280___organismNeospora_caninum_Liverpool___conserved_hypothetical_protein
Neospora_caninumLIV__580493
Siedleckia_nematoides__20033
Babesia_microtiRI__11908
Babesia_bovisT2Bo__30626
Babesia_bigeminaBOND__90627
Plasmodium_chabaudi__8510
pcha_PCHAS_0405900___organismPlasmodium_chabaudi_chabaudi___ATP_synthaseassociated_protein_putative
pyoe_PY17X_0407200___organismPlasmodium_yoelii_17X___ATP_synthaseassociated_protein_putative
pber_PBANKA_0405000___organismPlasmodium_berghei_ANKA___ATP_synthaseassociated_protein_putative
Plasmodium_bergheiANKA__7718
pkno_PKNH_0836100___organismPlasmodium_knowlesi_strain_H___ATP_synthaseassociated_protein_putative
pvip_PVP01_0833600___organismPlasmodium_vivax___ATP_synthaseassociated_protein_putative
Plasmodium_falciparum3D7__424
pfal_PF3D7_0306600___organismPlasmodium_falciparum_3D7___ATP_synthaseassociated_protein_putative
Haemoproteus_tartakovskyi__1162
Rhytidocystis_sp__4745
Theileria_equiWA__22724
tpar_TP01_0071___organismTheileria_parva_strain_Muguga___hypothetical_protein_conserved
tann_TA19930___organismTheileria_annulata_strain_Ankara___hypothetical_protein_conserved
Platyproteum_sp__8460
Vitrella_brassicaformis__7523
vbra_Vbra_8263___organismVitrella_brassicaformis_CCMP3155___hypothetical_protein
0.6

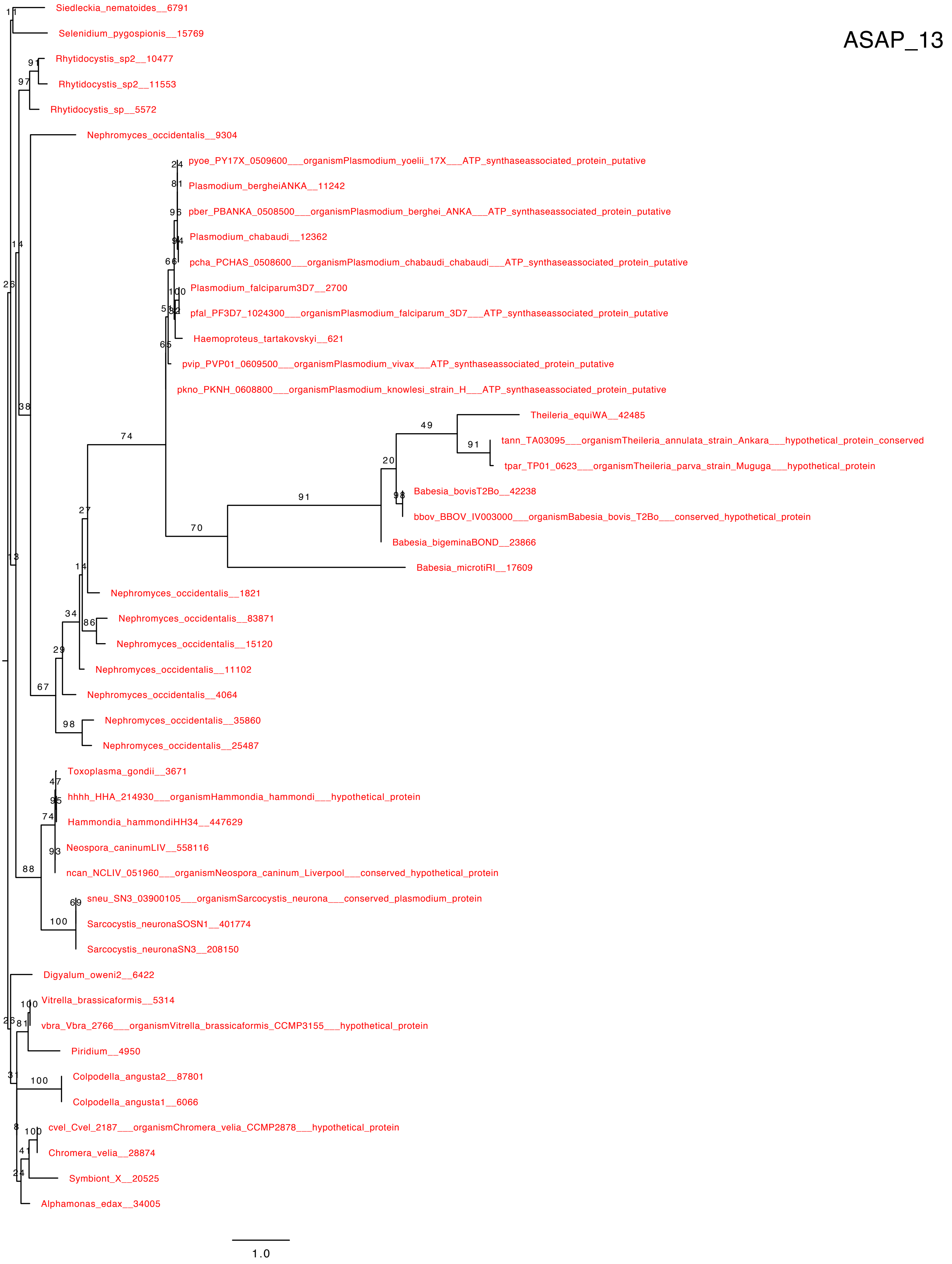
ASAP_13
Siedleckia_nematoides__6791
Selenidium_pygospionis__15769
Rhytidocystis_sp2__10477
Rhytidocystis_sp2__11553
Rhytidocystis_sp__5572
Nephromyces_occidentalis__9304
pyoe_PY17X_0509600___organismPlasmodium_yoelii_17X___ATP_synthaseassociated_protein_putative
Plasmodium_bergheiANKA__11242
pber_PBANKA_0508500___organismPlasmodium_berghei_ANKA___ATP_synthaseassociated_protein_putative
Plasmodium_chabaudi__12362
pcha_PCHAS_0508600___organismPlasmodium_chabaudi_chabaudi___ATP_synthaseassociated_protein_putative
Plasmodium_falciparum3D7__2700
pfal_PF3D7_1024300___organismPlasmodium_falciparum_3D7___ATP_synthaseassociated_protein_putative
Haemoproteus_tartakovskyi__621
pvip_PVP01_0609500___organismPlasmodium_vivax___ATP_synthaseassociated_protein_putative
pkno_PKNH_0608800___organismPlasmodium_knowlesi_strain_H___ATP_synthaseassociated_protein_putative
Theileria_equiWA__42485
tann_TA03095___organismTheileria_annulata_strain_Ankara___hypothetical_protein_conserved
tpar_TP01_0623___organismTheileria_parva_strain_Muguga___hypothetical_protein
Babesia_bovisT2Bo__42238
bbov_BBOV_IV003000___organismBabesia_bovis_T2Bo___conserved_hypothetical_protein
Babesia_bigeminaBOND__23866
Babesia_microtiRI__17609
Nephromyces_occidentalis__1821
Nephromyces_occidentalis__83871
Nephromyces_occidentalis__15120
Nephromyces_occidentalis__11102
Nephromyces_occidentalis__4064
Nephromyces_occidentalis__35860
Nephromyces_occidentalis__25487
Toxoplasma_gondii__3671
hhhh_HHA_214930___organismHammondia_hammondi___hypothetical_protein
Hammondia_hammondiHH34__447629
Neospora_caninumLIV__558116
ncan_NCLIV_051960___organismNeospora_caninum_Liverpool___conserved_hypothetical_protein
sneu_SN3_03900105___organismSarcocystis_neurona___conserved_plasmodium_protein
Sarcocystis_neuronaSOSN1__401774
Sarcocystis_neuronaSN3__208150
Digyalum_oweni2__6422
Vitrella_brassicaformis__5314
vbra_Vbra_2766___organismVitrella_brassicaformis_CCMP3155___hypothetical_protein
Piridium__4950
Colpodella_angusta2__87801
Colpodella_angusta1__6066
cvel_Cvel_2187___organismChromera_velia_CCMP2878___hypothetical_protein
Chromera_velia__28874
Symbiont_X__20525
Alphamonas_edax__34005
1.0

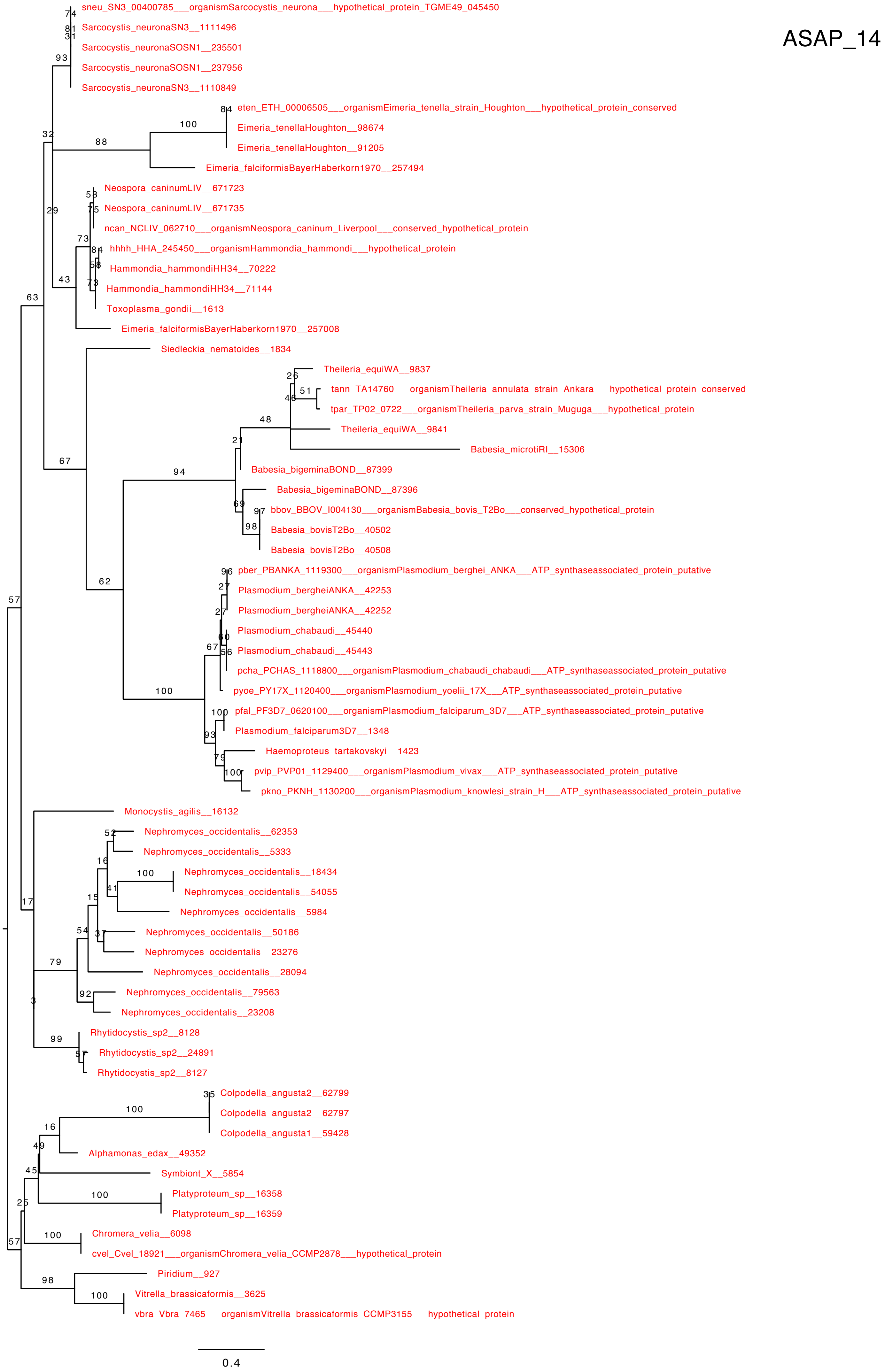

ASAP_14
sneu_SN3_00400785___organismSarcocystis_neurona___hypothetical_protein_TGME49_045450
Sarcocystis_neuronaSN3__1111496
Sarcocystis_neuronaSOSN1__235501
Sarcocystis_neuronaSOSN1__237956
Sarcocystis_neuronaSN3__1110849
eten_ETH_00006505___organismEimeria_tenella_strain_Houghton___hypothetical_protein_conserved
Eimeria_tenellaHoughton__98674
Eimeria_tenellaHoughton__91205
Eimeria_falciformisBayerHaberkorn1970__257494
Neospora_caninumLIV__671723
Neospora_caninumLIV__671735
ncan_NCLIV_062710___organismNeospora_caninum_Liverpool___conserved_hypothetical_protein
hhhh_HHA_245450___organismHammondia_hammondi___hypothetical_protein
Hammondia_hammondiHH34__70222
Hammondia_hammondiHH34__71144
Toxoplasma_gondii__1613
Eimeria_falciformisBayerHaberkorn1970__257008
Siedleckia_nematoides__1834
Theileria_equiWA__9837
tann_TA14760___organismTheileria_annulata_strain_Ankara___hypothetical_protein_conserved
tpar_TP02_0722___organismTheileria_parva_strain_Muguga___hypothetical_protein
Theileria_equiWA__9841
Babesia_microtiRI__15306
Babesia_bigeminaBOND__87399
Babesia_bigeminaBOND__87396
bbov_BBOV_I004130___organismBabesia_bovis_T2Bo___conserved_hypothetical_protein
Babesia_bovisT2Bo__40502
Babesia_bovisT2Bo__40508
pber_PBANKA_1119300___organismPlasmodium_berghei_ANKA___ATP_synthaseassociated_protein_putative
Plasmodium_bergheiANKA__42253
Plasmodium_bergheiANKA__42252
Plasmodium_chabaudi__45440
Plasmodium_chabaudi__45443
pcha_PCHAS_1118800___organismPlasmodium_chabaudi_chabaudi___ATP_synthaseassociated_protein_putative
pyoe_PY17X_1120400___organismPlasmodium_yoelii_17X___ATP_synthaseassociated_protein_putative
pfal_PF3D7_0620100___organismPlasmodium_falciparum_3D7___ATP_synthaseassociated_protein_putative
Plasmodium_falciparum3D7__1348
Haemoproteus_tartakovskyi__1423
pvip_PVP01_1129400___organismPlasmodium_vivax___ATP_synthaseassociated_protein_putative
pkno_PKNH_1130200___organismPlasmodium_knowlesi_strain_H___ATP_synthaseassociated_protein_putative
Monocystis_agilis__16132
Nephromyces_occidentalis__62353
Nephromyces_occidentalis__5333
Nephromyces_occidentalis__18434
Nephromyces_occidentalis__54055
Nephromyces_occidentalis__5984
Nephromyces_occidentalis__50186
Nephromyces_occidentalis__23276
Nephromyces_occidentalis__28094
Nephromyces_occidentalis__79563
Nephromyces_occidentalis__23208
Rhytidocystis_sp2__8128
Rhytidocystis_sp2__24891
Rhytidocystis_sp2__8127
Colpodella_angusta2__62799
Colpodella_angusta2__62797
Colpodella_angusta1__59428
Alphamonas_edax__49352
Symbiont_X__5854
Platyproteum_sp__16358
Platyproteum_sp__16359
Chromera_velia__6098
cvel_Cvel_18921___organismChromera_velia_CCMP2878___hypothetical_protein
Piridium__927
Vitrella_brassicaformis__3625
vbra_Vbra_7465___organismVitrella_brassicaformis_CCMP3155___hypothetical_protein
0.4

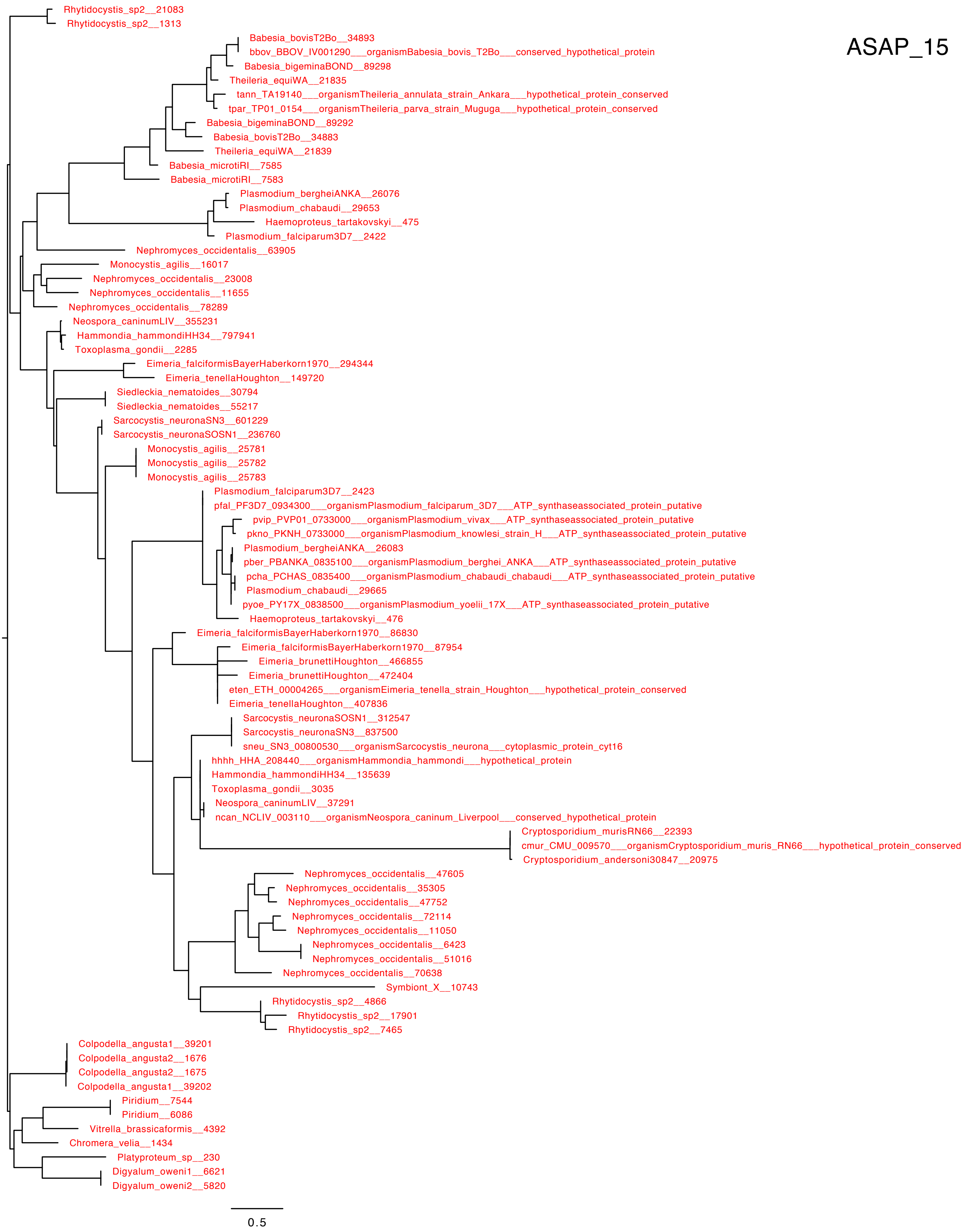
ASAP_15
Rhytidocystis_sp2__21083
Rhytidocystis_sp2__1313
Babesia_bovisT2Bo__34893
bbov_BBOV_IV001290___organismBabesia_bovis_T2Bo___conserved_hypothetical_protein
Babesia_bigeminaBOND__89298
Theileria_equiWA__21835
tann_TA19140___organismTheileria_annulata_strain_Ankara___hypothetical_protein_conserved
tpar_TP01_0154___organismTheileria_parva_strain_Muguga___hypothetical_protein_conserved
Babesia_bigeminaBOND__89292
Babesia_bovisT2Bo__34883
Theileria_equiWA__21839
Babesia_microtiRI__7585
Babesia_microtiRI__7583
Plasmodium_bergheiANKA__26076
Plasmodium_chabaudi__29653
Haemoproteus_tartakovskyi__475
Plasmodium_falciparum3D7__2422
Nephromyces_occidentalis__63905
Monocystis_agilis__16017
Nephromyces_occidentalis__23008
Nephromyces_occidentalis__11655
Nephromyces_occidentalis__78289
Neospora_caninumLIV__355231
Hammondia_hammondiHH34__797941
Toxoplasma_gondii__2285
Eimeria_falciformisBayerHaberkorn1970__294344
Eimeria_tenellaHoughton__149720
Siedleckia_nematoides__30794
Siedleckia_nematoides__55217
Sarcocystis_neuronaSN3__601229
Sarcocystis_neuronaSOSN1__236760
Monocystis_agilis__25781
Monocystis_agilis__25782
Monocystis_agilis__25783
Plasmodium_falciparum3D7__2423
pfal_PF3D7_0934300___organismPlasmodium_falciparum_3D7___ATP_synthaseassociated_protein_putative
pvip_PVP01_0733000___organismPlasmodium_vivax___ATP_synthaseassociated_protein_putative
pkno_PKNH_0733000___organismPlasmodium_knowlesi_strain_H___ATP_synthaseassociated_protein_putative
Plasmodium_bergheiANKA__26083
pber_PBANKA_0835100___organismPlasmodium_berghei_ANKA___ATP_synthaseassociated_protein_putative
pcha_PCHAS_0835400___organismPlasmodium_chabaudi_chabaudi___ATP_synthaseassociated_protein_putative
Plasmodium_chabaudi__29665
pyoe_PY17X_0838500___organismPlasmodium_yoelii_17X___ATP_synthaseassociated_protein_putative
Haemoproteus_tartakovskyi__476
Eimeria_falciformisBayerHaberkorn1970__86830
Eimeria_falciformisBayerHaberkorn1970__87954
Eimeria_brunettiHoughton__466855
Eimeria_brunettiHoughton__472404
eten_ETH_00004265___organismEimeria_tenella_strain_Houghton___hypothetical_protein_conserved
Eimeria_tenellaHoughton__407836
Sarcocystis_neuronaSOSN1__312547
Sarcocystis_neuronaSN3__837500
sneu_SN3_00800530___organismSarcocystis_neurona___cytoplasmic_protein_cyt16
hhhh_HHA_208440___organismHammondia_hammondi___hypothetical_protein
Hammondia_hammondiHH34__135639
Toxoplasma_gondii__3035
Neospora_caninumLIV__37291
ncan_NCLIV_003110___organismNeospora_caninum_Liverpool___conserved_hypothetical_protein
Cryptosporidium_murisRN66__22393
cmur_CMU_009570___organismCryptosporidium_muris_RN66___hypothetical_protein_conserved
Cryptosporidium_andersoni30847__20975
Nephromyces_occidentalis__47605
Nephromyces_occidentalis__35305
Nephromyces_occidentalis__47752
Nephromyces_occidentalis__72114
Nephromyces_occidentalis__11050
Nephromyces_occidentalis__6423
Nephromyces_occidentalis__51016
Nephromyces_occidentalis__70638
Symbiont_X__10743
Rhytidocystis_sp2__4866
Rhytidocystis_sp2__17901
Rhytidocystis_sp2__7465
Colpodella_angusta1__39201
Colpodella_angusta2__1676
Colpodella_angusta2__1675
Colpodella_angusta1__39202
Piridium__7544
Piridium__6086
Vitrella_brassicaformis__4392
Chromera_velia__1434
Platyproteum_sp__230
Digyalum_oweni1__6621
Digyalum_oweni2__5820
0.5

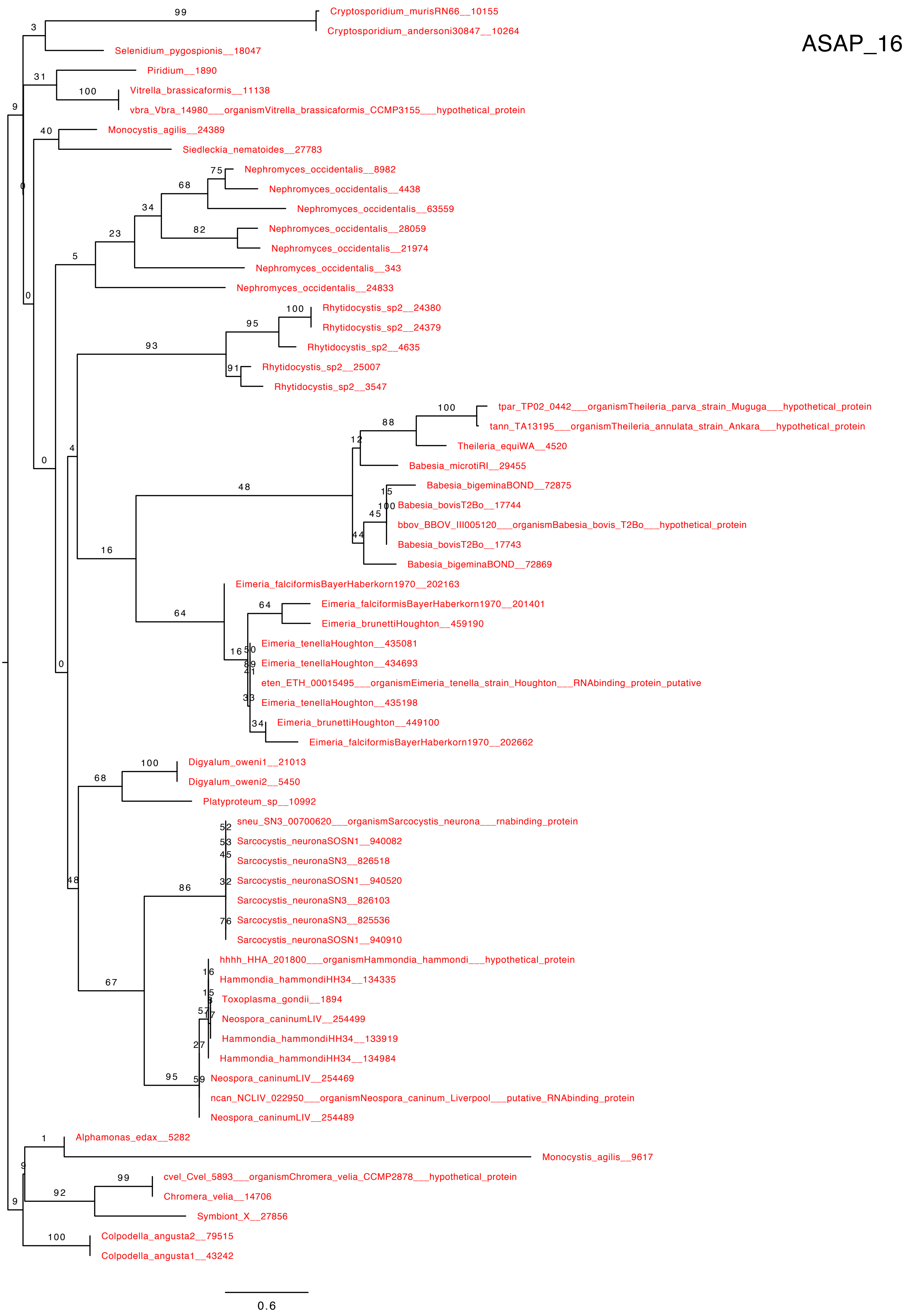
ASAP_16
Cryptosporidium_murisRN66__10155
Cryptosporidium_andersoni30847__10264
Selenidium_pygospionis__18047
Piridium__1890
Vitrella_brassicaformis__11138
vbra_Vbra_14980___organismVitrella_brassicaformis_CCMP3155___hypothetical_protein
Monocystis_agilis__24389
Siedleckia_nematoides__27783
Nephromyces_occidentalis__8982
Nephromyces_occidentalis__4438
Nephromyces_occidentalis__63559
Nephromyces_occidentalis__28059
Nephromyces_occidentalis__21974
Nephromyces_occidentalis__343
Nephromyces_occidentalis__24833
Rhytidocystis_sp2__24380
Rhytidocystis_sp2__24379
Rhytidocystis_sp2__4635
Rhytidocystis_sp2__25007
Rhytidocystis_sp2__3547
tpar_TP02_0442___organismTheileria_parva_strain_Muguga___hypothetical_protein
tann_TA13195___organismTheileria_annulata_strain_Ankara___hypothetical_protein
Theileria_equiWA__4520
Babesia_microtiRI__29455
Babesia_bigeminaBOND__72875
Babesia_bovisT2Bo__17744
bbov_BBOV_III005120___organismBabesia_bovis_T2Bo___hypothetical_protein
Babesia_bovisT2Bo__17743
Babesia_bigeminaBOND__72869
Eimeria_falciformisBayerHaberkorn1970__202163
Eimeria_falciformisBayerHaberkorn1970__201401
Eimeria_brunettiHoughton__459190
Eimeria_tenellaHoughton__435081
Eimeria_tenellaHoughton__434693
eten_ETH_00015495___organismEimeria_tenella_strain_Houghton___RNAbinding_protein_putative
Eimeria_tenellaHoughton__435198
Eimeria_brunettiHoughton__449100
Eimeria_falciformisBayerHaberkorn1970__202662
Digyalum_oweni1__21013
Digyalum_oweni2__5450
Platyproteum_sp__10992
sneu_SN3_00700620___organismSarcocystis_neurona___rnabinding_protein
Sarcocystis_neuronaSOSN1__940082
Sarcocystis_neuronaSN3__826518
Sarcocystis_neuronaSOSN1__940520
Sarcocystis_neuronaSN3__826103
Sarcocystis_neuronaSN3__825536
Sarcocystis_neuronaSOSN1__940910
hhhh_HHA_201800___organismHammondia_hammondi___hypothetical_protein
Hammondia_hammondiHH34__134335
Toxoplasma_gondii__1894
Neospora_caninumLIV__254499
Hammondia_hammondiHH34__133919
Hammondia_hammondiHH34__134984
Neospora_caninumLIV__254469
ncan_NCLIV_022950___organismNeospora_caninum_Liverpool___putative_RNAbinding_protein
Neospora_caninumLIV__254489
Alphamonas_edax__5282
Monocystis_agilis__9617
cvel_Cvel_5893___organismChromera_velia_CCMP2878___hypothetical_protein
Chromera_velia__14706
Symbiont_X__27856
Colpodella_angusta2__79515
Colpodella_angusta1__43242
0.6

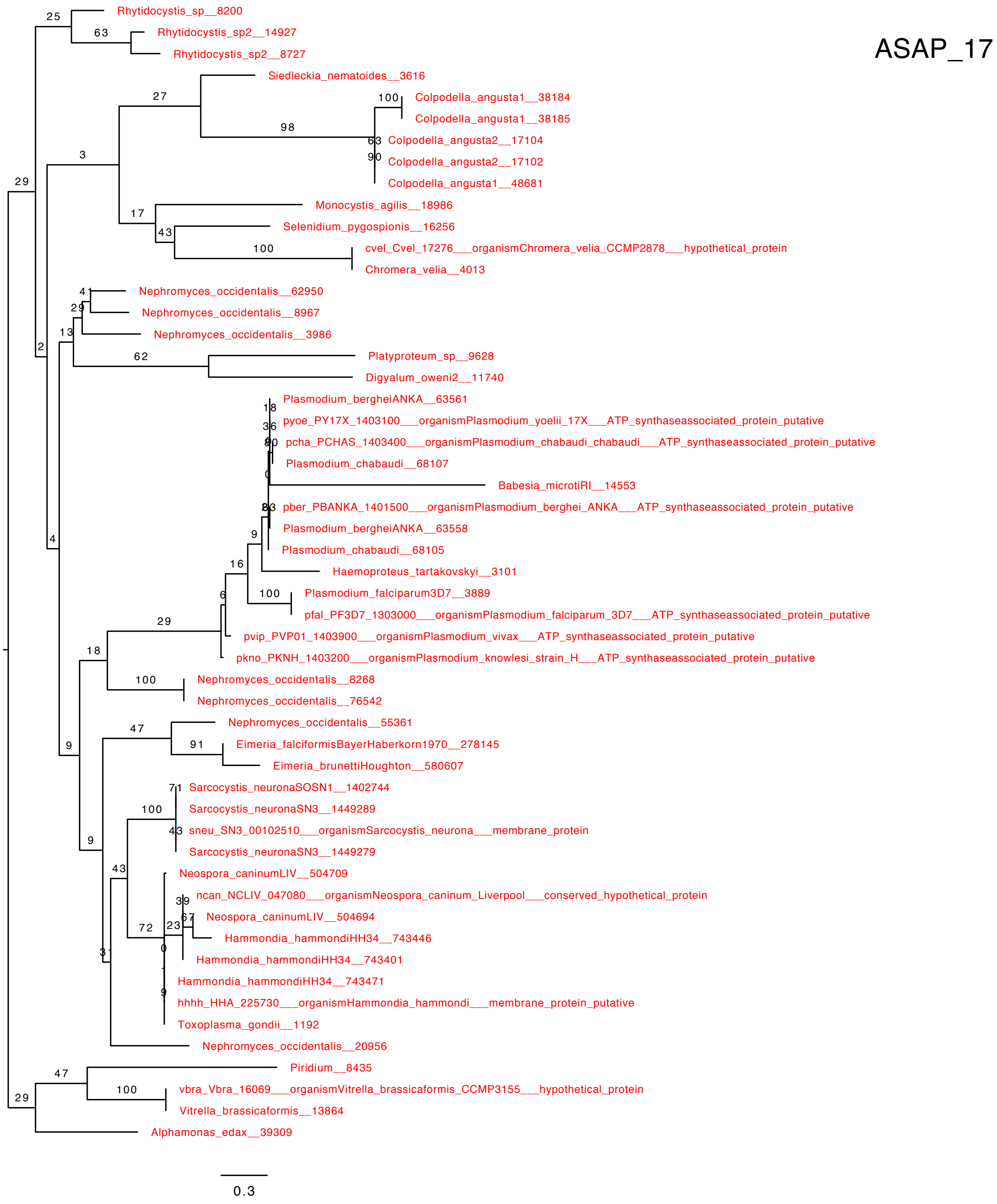
ASAP_17
Rhytidocystis_sp__8200
Rhytidocystis_sp2__14927
Rhytidocystis_sp2__8727
Siedleckia_nematoides__3616
Colpodella_angusta1__38184
Colpodella_angusta1__38185
Colpodella_angusta2__17104
Colpodella_angusta2__17102
Colpodella_angusta1__48681
Monocystis_agilis__18986
Selenidium_pygospionis__16256
cvel_Cvel_17276___organismChromera_velia_CCMP2878___hypothetical_protein
Chromera_velia__4013
Nephromyces_occidentalis__62950
Nephromyces_occidentalis__8967
Nephromyces_occidentalis__3986
Platyproteum_sp__9628
Digyalum_oweni2__11740
Plasmodium_bergheiANKA__63561
pyoe_PY17X_1403100___organismPlasmodium_yoelii_17X___ATP_synthaseassociated_protein_putative
pcha_PCHAS_1403400___organismPlasmodium_chabaudi_chabaudi___ATP_synthaseassociated_protein_putative
Plasmodium_chabaudi__68107
Babesia_microtiRI__14553
pber_PBANKA_1401500___organismPlasmodium_berghei_ANKA___ATP_synthaseassociated_protein_putative
Plasmodium_bergheiANKA__63558
Plasmodium_chabaudi__68105
Haemoproteus_tartakovskyi__3101
Plasmodium_falciparum3D7__3889
pfal_PF3D7_1303000___organismPlasmodium_falciparum_3D7___ATP_synthaseassociated_protein_putative
pvip_PVP01_1403900___organismPlasmodium_vivax___ATP_synthaseassociated_protein_putative
pkno_PKNH_1403200___organismPlasmodium_knowlesi_strain_H___ATP_synthaseassociated_protein_putative
Nephromyces_occidentalis__8268
Nephromyces_occidentalis__76542
Nephromyces_occidentalis__55361
Eimeria_falciformisBayerHaberkorn1970__278145
Eimeria_brunettiHoughton__580607
Sarcocystis_neuronaSOSN1__1402744
Sarcocystis_neuronaSN3__1449289
sneu_SN3_00102510___organismSarcocystis_neurona___membrane_protein
Sarcocystis_neuronaSN3__1449279
Neospora_caninumLIV__504709
ncan_NCLIV_047080___organismNeospora_caninum_Liverpool___conserved_hypothetical_protein
Neospora_caninumLIV__504694
Hammondia_hammondiHH34__743446
Hammondia_hammondiHH34__743401
Hammondia_hammondiHH34__743471
hhhh_HHA_225730___organismHammondia_hammondi___membrane_protein_putative
Toxoplasma_gondii__1192
Nephromyces_occidentalis__20956
Piridium__8435
vbra_Vbra_16069___organismVitrella_brassicaformis_CCMP3155___hypothetical_protein
Vitrella_brassicaformis__13864
Alphamonas_edax__39309
0.3

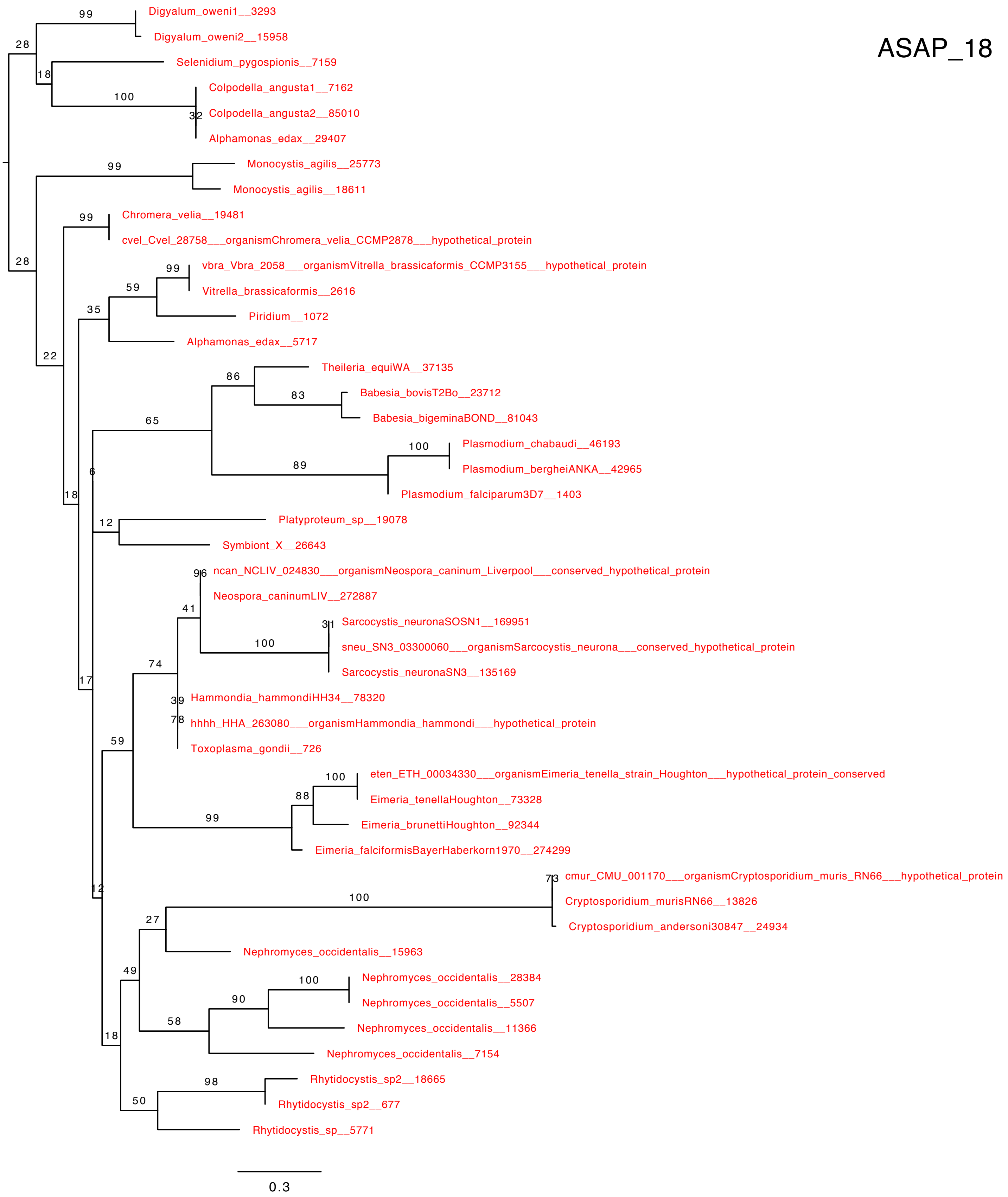
ASAP_18
Digyalum_oweni1__3293
Digyalum_oweni2__15958
Selenidium_pygospionis__7159
Colpodella_angusta1__7162
Colpodella_angusta2__85010
Alphamonas_edax__29407
Monocystis_agilis__25773
Monocystis_agilis__18611
Chromera_velia__19481
cvel_Cvel_28758___organismChromera_velia_CCMP2878___hypothetical_protein
vbra_Vbra_2058___organismVitrella_brassicaformis_CCMP3155___hypothetical_protein
Vitrella_brassicaformis__2616
Piridium__1072
Alphamonas_edax__5717
Theileria_equiWA__37135
Babesia_bovisT2Bo__23712
Babesia_bigeminaBOND__81043
Plasmodium_chabaudi__46193
Plasmodium_bergheiANKA__42965
Plasmodium_falciparum3D7__1403
Platyproteum_sp__19078
Symbiont_X__26643
ncan_NCLIV_024830___organismNeospora_caninum_Liverpool___conserved_hypothetical_protein
Neospora_caninumLIV__272887
Sarcocystis_neuronaSOSN1__169951
sneu_SN3_03300060___organismSarcocystis_neurona___conserved_hypothetical_protein
Sarcocystis_neuronaSN3__135169
Hammondia_hammondiHH34__78320
hhhh_HHA_263080___organismHammondia_hammondi___hypothetical_protein
Toxoplasma_gondii__726
eten_ETH_00034330___organismEimeria_tenella_strain_Houghton___hypothetical_protein_conserved
Eimeria_tenellaHoughton__73328
Eimeria_brunettiHoughton__92344
Eimeria_falciformisBayerHaberkorn1970__274299
cmur_CMU_001170___organismCryptosporidium_muris_RN66___hypothetical_protein
Cryptosporidium_murisRN66__13826
Cryptosporidium_andersoni30847__24934
Nephromyces_occidentalis__15963
Nephromyces_occidentalis__28384
Nephromyces_occidentalis__5507
Nephromyces_occidentalis__11366
Nephromyces_occidentalis__7154
Rhytidocystis_sp2__18665
Rhytidocystis_sp2__677
Rhytidocystis_sp__5771
0.3

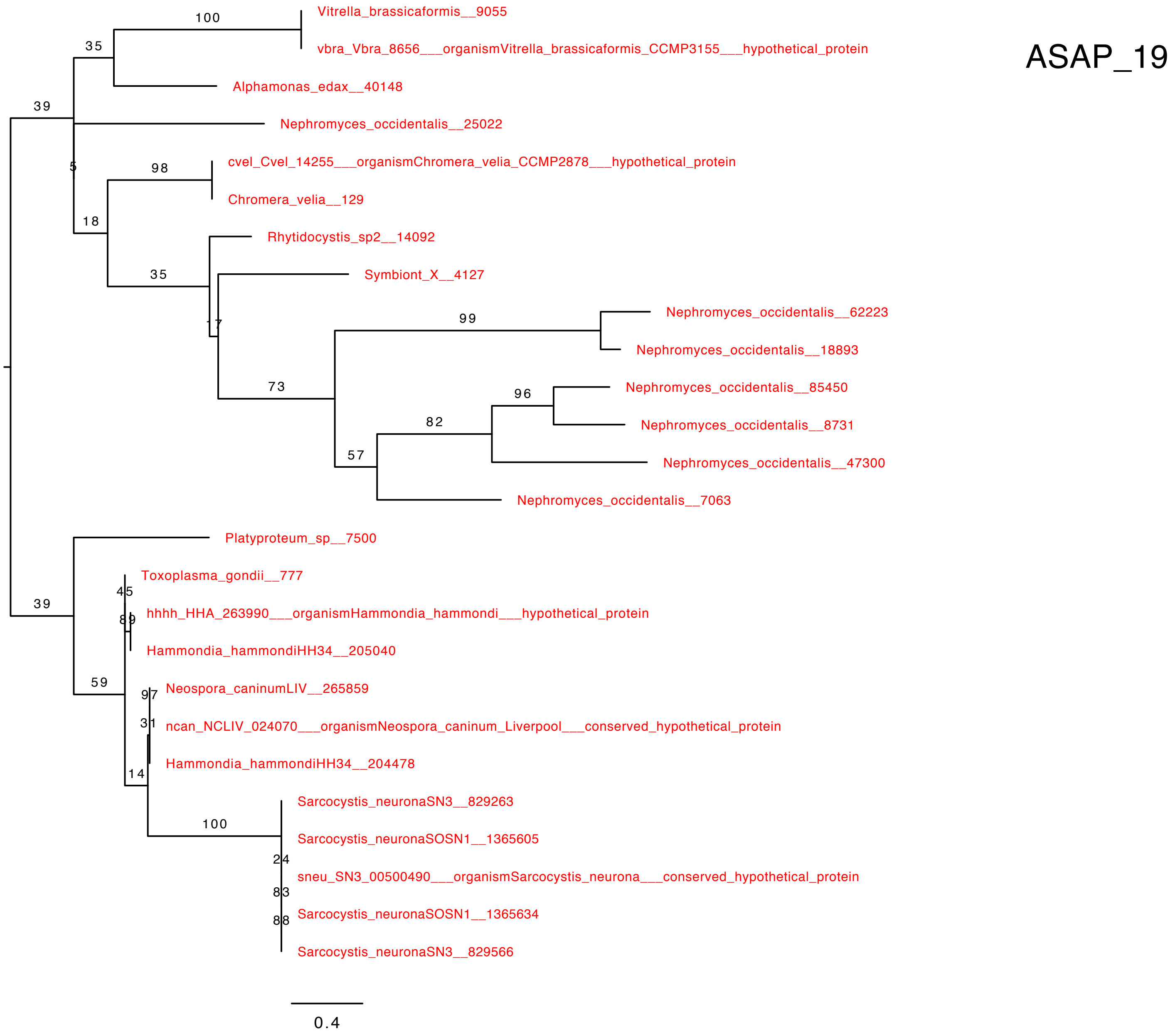

ASAP_19
Vitrella_brassicaformis__9055
vbra_Vbra_8656___organismVitrella_brassicaformis_CCMP3155___hypothetical_protein
Alphamonas_edax__40148
Nephromyces_occidentalis__25022
cvel_Cvel_14255___organismChromera_velia_CCMP2878___hypothetical_protein
Chromera_velia__129
Rhytidocystis_sp2__14092
Symbiont_X__4127
Nephromyces_occidentalis__62223
Nephromyces_occidentalis__18893
Nephromyces_occidentalis__85450
Nephromyces_occidentalis__8731
Nephromyces_occidentalis__47300
Nephromyces_occidentalis__7063
Platyproteum_sp__7500
Toxoplasma_gondii__777
hhhh_HHA_263990___organismHammondia_hammondi___hypothetical_protein
Hammondia_hammondiHH34__205040
Neospora_caninumLIV__265859
ncan_NCLIV_024070___organismNeospora_caninum_Liverpool___conserved_hypothetical_protein
Hammondia_hammondiHH34__204478
Sarcocystis_neuronaSN3__829263
Sarcocystis_neuronaSOSN1__1365605
sneu_SN3_00500490___organismSarcocystis_neurona___conserved_hypothetical_protein
Sarcocystis_neuronaSOSN1__1365634
Sarcocystis_neuronaSN3__829566
100
35
39
5
98
18
35
17
99
73
96
82
57
39
45
89
59
97
31
14
100
24
83
88
0.4

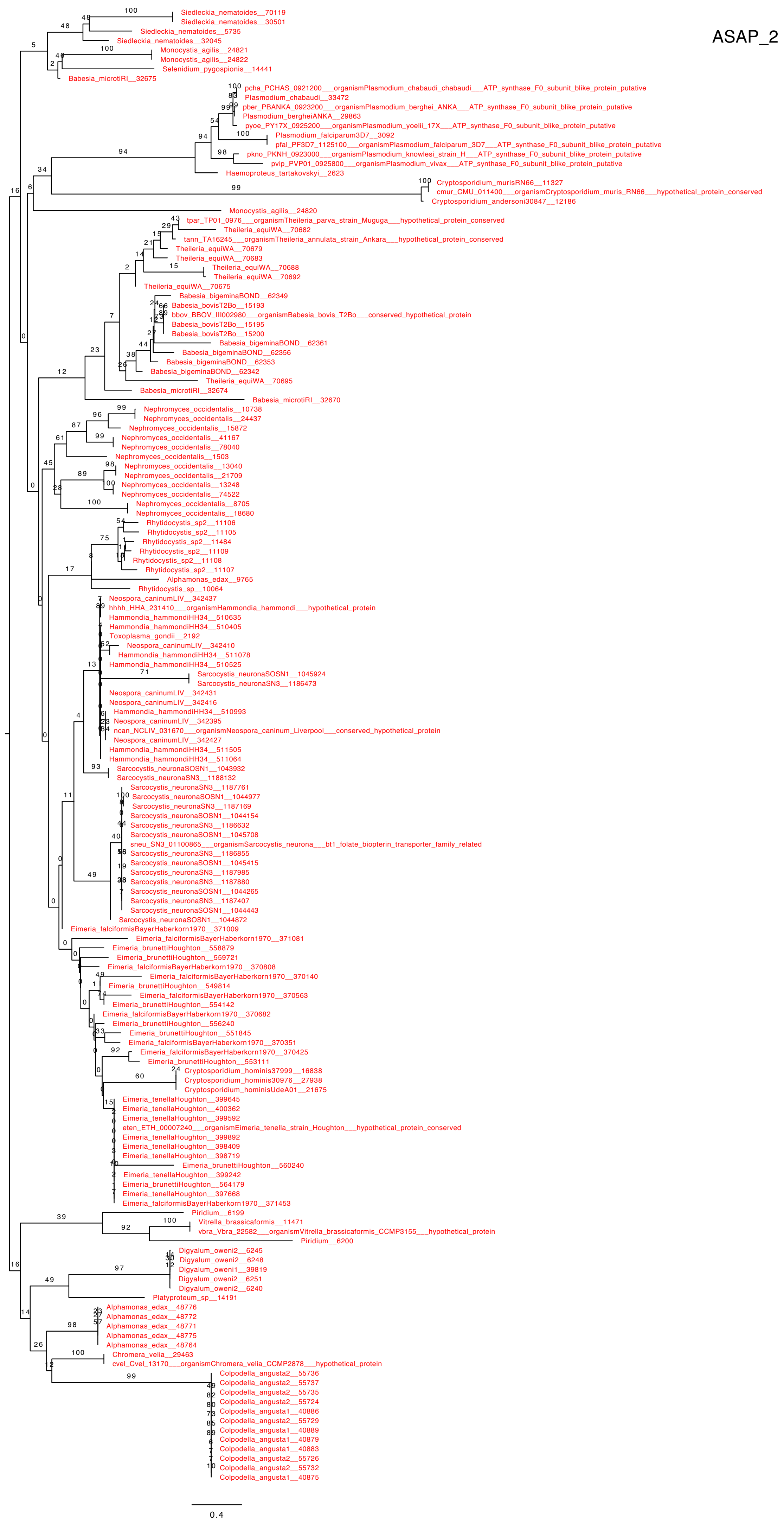
ASAP_2
Siedleckia_nematoides__70119
Siedleckia_nematoides__30501
Siedleckia_nematoides__5735
Siedleckia_nematoides__32045
Monocystis_agilis__24821
Monocystis_agilis__24822
Selenidium_pygospionis__14441
Babesia_microtiRI__32675
pcha_PCHAS_0921200___organismPlasmodium_chabaudi_chabaudi___ATP_synthase_F0_subunit_blike_protein_putative
Plasmodium_chabaudi__33472
pber_PBANKA_0923200___organismPlasmodium_berghei_ANKA___ATP_synthase_F0_subunit_blike_protein_putative
Plasmodium_bergheiANKA__29863
pyoe_PY17X_0925200___organismPlasmodium_yoelii_17X___ATP_synthase_F0_subunit_blike_protein_putative
Plasmodium_falciparum3D7__3092
pfal_PF3D7_1125100___organismPlasmodium_falciparum_3D7___ATP_synthase_F0_subunit_blike_protein_putative
pkno_PKNH_0923000___organismPlasmodium_knowlesi_strain_H___ATP_synthase_F0_subunit_blike_protein_putative
pvip_PVP01_0925800___organismPlasmodium_vivax___ATP_synthase_F0_subunit_blike_protein_putative
Haemoproteus_tartakovskyi__2623
Cryptosporidium_murisRN66__11327
cmur_CMU_011400___organismCryptosporidium_muris_RN66___hypothetical_protein_conserved
Cryptosporidium_andersoni30847__12186
Monocystis_agilis__24820
tpar_TP01_0976___organismTheileria_parva_strain_Muguga___hypothetical_protein_conserved
Theileria_equiWA__70682
tann_TA16245___organismTheileria_annulata_strain_Ankara___hypothetical_protein_conserved
Theileria_equiWA__70679
Theileria_equiWA__70683
Theileria_equiWA__70688
Theileria_equiWA__70692
Theileria_equiWA__70675
Babesia_bigeminaBOND__62349
Babesia_bovisT2Bo__15193
bbov_BBOV_III002980___organismBabesia_bovis_T2Bo___conserved_hypothetical_protein
Babesia_bovisT2Bo__15195
Babesia_bovisT2Bo__15200
Babesia_bigeminaBOND__62361
Babesia_bigeminaBOND__62356
Babesia_bigeminaBOND__62353
Babesia_bigeminaBOND__62342
Theileria_equiWA__70695
Babesia_microtiRI__32674
Babesia_microtiRI__32670
Nephromyces_occidentalis__10738
Nephromyces_occidentalis__24437
Nephromyces_occidentalis__15872
Nephromyces_occidentalis__41167
Nephromyces_occidentalis__78040
Nephromyces_occidentalis__1503
Nephromyces_occidentalis__13040
Nephromyces_occidentalis__21709
Nephromyces_occidentalis__13248
Nephromyces_occidentalis__74522
Nephromyces_occidentalis__8705
Nephromyces_occidentalis__18680
Rhytidocystis_sp2__11106
Rhytidocystis_sp2__11105
Rhytidocystis_sp2__11484
Rhytidocystis_sp2__11109
Rhytidocystis_sp2__11108
Rhytidocystis_sp2__11107
Alphamonas_edax__9765
Rhytidocystis_sp__10064
Neospora_caninumLIV__342437
hhhh_HHA_231410___organismHammondia_hammondi___hypothetical_protein
Hammondia_hammondiHH34__510635
Hammondia_hammondiHH34__510405
Toxoplasma_gondii__2192
Neospora_caninumLIV__342410
Hammondia_hammondiHH34__511078
Hammondia_hammondiHH34__510525
Sarcocystis_neuronaSOSN1__1045924
Sarcocystis_neuronaSN3__1186473
Neospora_caninumLIV__342431
Neospora_caninumLIV__342416
Hammondia_hammondiHH34__510993
Neospora_caninumLIV__342395
ncan_NCLIV_031670___organismNeospora_caninum_Liverpool___conserved_hypothetical_protein
Neospora_caninumLIV__342427
Hammondia_hammondiHH34__511505
Hammondia_hammondiHH34__511064
Sarcocystis_neuronaSOSN1__1043932
Sarcocystis_neuronaSN3__1188132
Sarcocystis_neuronaSN3__1187761
Sarcocystis_neuronaSOSN1__1044977
Sarcocystis_neuronaSN3__1187169
Sarcocystis_neuronaSOSN1__1044154
Sarcocystis_neuronaSN3__1186632
Sarcocystis_neuronaSOSN1__1045708
sneu_SN3_01100865___organismSarcocystis_neurona___bt1_folate_biopterin_transporter_family_related
Sarcocystis_neuronaSN3__1186855
Sarcocystis_neuronaSOSN1__1045415
Sarcocystis_neuronaSN3__1187985
Sarcocystis_neuronaSN3__1187880
Sarcocystis_neuronaSOSN1__1044265
Sarcocystis_neuronaSN3__1187407
Sarcocystis_neuronaSOSN1__1044443
Sarcocystis_neuronaSOSN1__1044872
Eimeria_falciformisBayerHaberkorn1970__371009
Eimeria_falciformisBayerHaberkorn1970__371081
Eimeria_brunettiHoughton__558879
Eimeria_brunettiHoughton__559721
Eimeria_falciformisBayerHaberkorn1970__370808
Eimeria_falciformisBayerHaberkorn1970__370140
Eimeria_brunettiHoughton__549814
Eimeria_falciformisBayerHaberkorn1970__370563
Eimeria_brunettiHoughton__554142
Eimeria_falciformisBayerHaberkorn1970__370682
Eimeria_brunettiHoughton__556240
Eimeria_brunettiHoughton__551845
Eimeria_falciformisBayerHaberkorn1970__370351
Eimeria_falciformisBayerHaberkorn1970__370425
Eimeria_brunettiHoughton__553111
Cryptosporidium_hominis37999__16838
Cryptosporidium_hominis30976__27938
Cryptosporidium_hominisUdeA01__21675
Eimeria_tenellaHoughton__399645
Eimeria_tenellaHoughton__400362
Eimeria_tenellaHoughton__399592
eten_ETH_00007240___organismEimeria_tenella_strain_Houghton___hypothetical_protein_conserved
Eimeria_tenellaHoughton__399892
Eimeria_tenellaHoughton__398409
Eimeria_tenellaHoughton__398719
Eimeria_brunettiHoughton__560240
Eimeria_tenellaHoughton__399242
Eimeria_brunettiHoughton__564179
Eimeria_tenellaHoughton__397668
Eimeria_falciformisBayerHaberkorn1970__371453
Piridium__6199
Vitrella_brassicaformis__11471
vbra_Vbra_22582___organismVitrella_brassicaformis_CCMP3155___hypothetical_protein
Piridium__6200
Digyalum_oweni2__6245
Digyalum_oweni2__6248
Digyalum_oweni1__39819
Digyalum_oweni2__6251
Digyalum_oweni2__6240
Platyproteum_sp__14191
Alphamonas_edax__48776
Alphamonas_edax__48772
Alphamonas_edax__48771
Alphamonas_edax__48775
Alphamonas_edax__48764
Chromera_velia__29463
cvel_Cvel_13170___organismChromera_velia_CCMP2878___hypothetical_protein
Colpodella_angusta2__55736
Colpodella_angusta2__55737
Colpodella_angusta2__55735
Colpodella_angusta2__55724
Colpodella_angusta1__40886
Colpodella_angusta2__55729
Colpodella_angusta1__40889
Colpodella_angusta1__40879
Colpodella_angusta1__40883
Colpodella_angusta2__55726
Colpodella_angusta2__55732
Colpodella_angusta1__40875
0.4

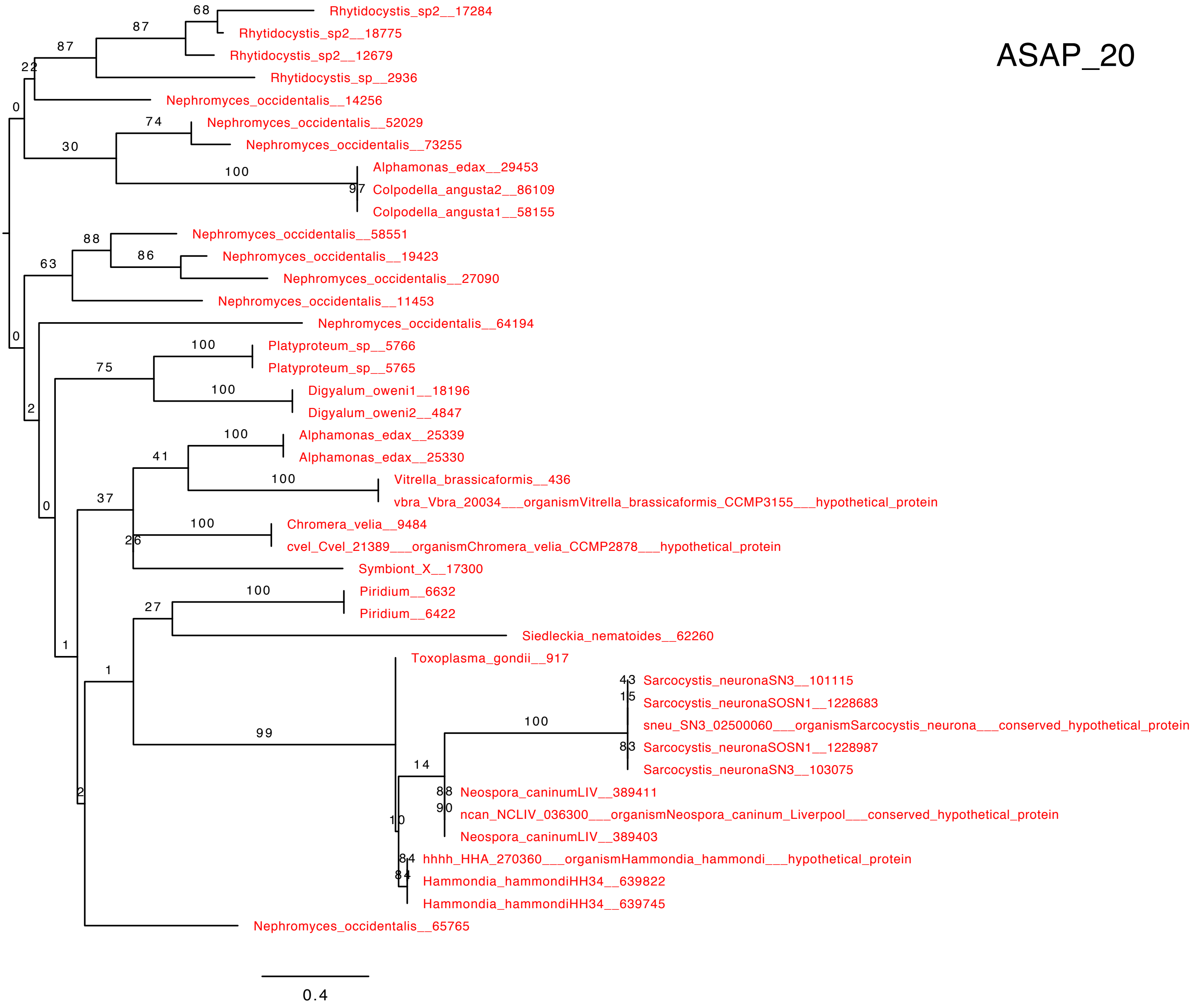

ASAP_20
Rhytidocystis_sp2__17284
Rhytidocystis_sp2__18775
Rhytidocystis_sp2__12679
Rhytidocystis_sp__2936
Nephromyces_occidentalis__14256
Nephromyces_occidentalis__52029
Nephromyces_occidentalis__73255
Alphamonas_edax__29453
Colpodella_angusta2__86109
Colpodella_angusta1__58155
Nephromyces_occidentalis__58551
Nephromyces_occidentalis__19423
Nephromyces_occidentalis__27090
Nephromyces_occidentalis__11453
Nephromyces_occidentalis__64194
Platyproteum_sp__5766
Platyproteum_sp__5765
Digyalum_oweni1__18196
Digyalum_oweni2__4847
Alphamonas_edax__25339
Alphamonas_edax__25330
Vitrella_brassicaformis__436
vbra_Vbra_20034___organismVitrella_brassicaformis_CCMP3155___hypothetical_protein
Chromera_velia__9484
cvel_Cvel_21389___organismChromera_velia_CCMP2878___hypothetical_protein
Symbiont_X__17300
Piridium__6632
Piridium__6422
Siedleckia_nematoides__62260
Toxoplasma_gondii__917
Sarcocystis_neuronaSN3__101115
Sarcocystis_neuronaSOSN1__1228683
sneu_SN3_02500060___organismSarcocystis_neurona___conserved_hypothetical_protein
Sarcocystis_neuronaSOSN1__1228987
Sarcocystis_neuronaSN3__103075
Neospora_caninumLIV__389411
ncan_NCLIV_036300___organismNeospora_caninum_Liverpool___conserved_hypothetical_protein
Neospora_caninumLIV__389403
hhhh_HHA_270360___organismHammondia_hammondi___hypothetical_protein
Hammondia_hammondiHH34__639822
Hammondia_hammondiHH34__639745
Nephromyces_occidentalis__65765
0.4

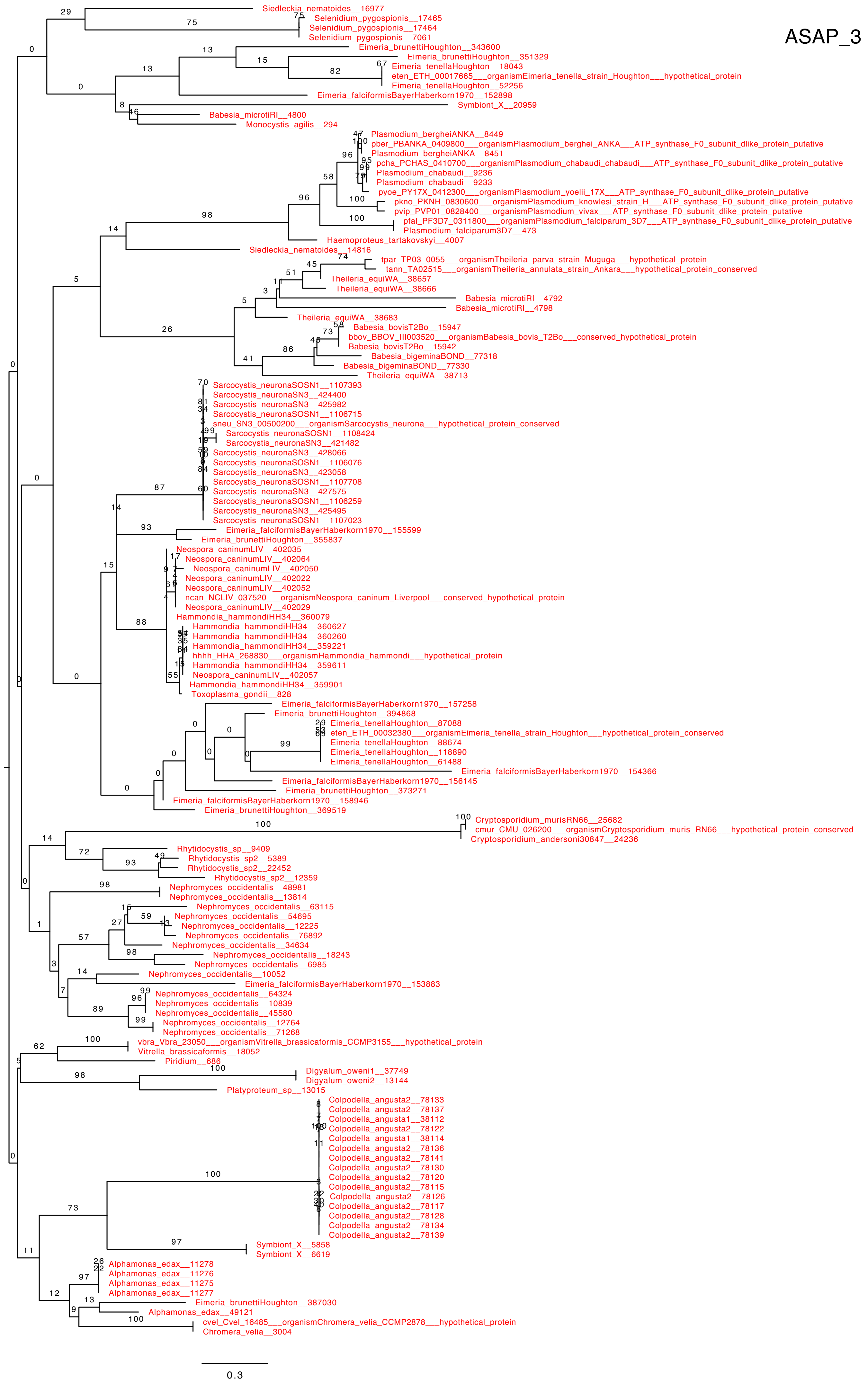
ASAP_3
Siedleckia_nematoides__16977
Selenidium_pygospionis__17465
Selenidium_pygospionis__17464
Selenidium_pygospionis__7061
Eimeria_brunettiHoughton__343600
Eimeria_brunettiHoughton__351329
Eimeria_tenellaHoughton__18043
eten_ETH_00017665___organismEimeria_tenella_strain_Houghton___hypothetical_protein
Eimeria_tenellaHoughton__52256
Eimeria_falciformisBayerHaberkorn1970__152898
Symbiont_X__20959
Babesia_microtiRI__4800
Monocystis_agilis__294
Plasmodium_bergheiANKA__8449
pber_PBANKA_0409800___organismPlasmodium_berghei_ANKA___ATP_synthase_F0_subunit_dlike_protein_putative
Plasmodium_bergheiANKA__8451
pcha_PCHAS_0410700___organismPlasmodium_chabaudi_chabaudi___ATP_synthase_F0_subunit_dlike_protein_putative
Plasmodium_chabaudi__9236
Plasmodium_chabaudi__9233
pyoe_PY17X_0412300___organismPlasmodium_yoelii_17X___ATP_synthase_F0_subunit_dlike_protein_putative
pkno_PKNH_0830600___organismPlasmodium_knowlesi_strain_H___ATP_synthase_F0_subunit_dlike_protein_putative
pvip_PVP01_0828400___organismPlasmodium_vivax___ATP_synthase_F0_subunit_dlike_protein_putative
pfal_PF3D7_0311800___organismPlasmodium_falciparum_3D7___ATP_synthase_F0_subunit_dlike_protein_putative
Plasmodium_falciparum3D7__473
Haemoproteus_tartakovskyi__4007
Siedleckia_nematoides__14816
tpar_TP03_0055___organismTheileria_parva_strain_Muguga___hypothetical_protein
tann_TA02515___organismTheileria_annulata_strain_Ankara___hypothetical_protein_conserved
Theileria_equiWA__38657
Theileria_equiWA__38666
Babesia_microtiRI__4792
Babesia_microtiRI__4798
Theileria_equiWA__38683
Babesia_bovisT2Bo__15947
bbov_BBOV_III003520___organismBabesia_bovis_T2Bo___conserved_hypothetical_protein
Babesia_bovisT2Bo__15942
Babesia_bigeminaBOND__77318
Babesia_bigeminaBOND__77330
Theileria_equiWA__38713
Sarcocystis_neuronaSOSN1__1107393
Sarcocystis_neuronaSN3__424400
Sarcocystis_neuronaSN3__425982
Sarcocystis_neuronaSOSN1__1106715
sneu_SN3_00500200___organismSarcocystis_neurona___hypothetical_protein_conserved
Sarcocystis_neuronaSOSN1__1108424
Sarcocystis_neuronaSN3__421482
Sarcocystis_neuronaSN3__428066
Sarcocystis_neuronaSOSN1__1106076
Sarcocystis_neuronaSN3__423058
Sarcocystis_neuronaSOSN1__1107708
Sarcocystis_neuronaSN3__427575
Sarcocystis_neuronaSOSN1__1106259
Sarcocystis_neuronaSN3__425495
Sarcocystis_neuronaSOSN1__1107023
Eimeria_falciformisBayerHaberkorn1970__155599
Eimeria_brunettiHoughton__355837
Neospora_caninumLIV__402035
Neospora_caninumLIV__402064
Neospora_caninumLIV__402050
Neospora_caninumLIV__402022
Neospora_caninumLIV__402052
ncan_NCLIV_037520___organismNeospora_caninum_Liverpool___conserved_hypothetical_protein
Neospora_caninumLIV__402029
Hammondia_hammondiHH34__360079
Hammondia_hammondiHH34__360627
Hammondia_hammondiHH34__360260
Hammondia_hammondiHH34__359221
hhhh_HHA_268830___organismHammondia_hammondi___hypothetical_protein
Hammondia_hammondiHH34__359611
Neospora_caninumLIV__402057
Hammondia_hammondiHH34__359901
Toxoplasma_gondii__828
Eimeria_falciformisBayerHaberkorn1970__157258
Eimeria_brunettiHoughton__394868
Eimeria_tenellaHoughton__87088
eten_ETH_00032380___organismEimeria_tenella_strain_Houghton___hypothetical_protein_conserved
Eimeria_tenellaHoughton__88674
Eimeria_tenellaHoughton__118890
Eimeria_tenellaHoughton__61488
Eimeria_falciformisBayerHaberkorn1970__154366
Eimeria_falciformisBayerHaberkorn1970__156145
Eimeria_brunettiHoughton__373271
Eimeria_falciformisBayerHaberkorn1970__158946
Eimeria_brunettiHoughton__369519
Cryptosporidium_murisRN66__25682
cmur_CMU_026200___organismCryptosporidium_muris_RN66___hypothetical_protein_conserved
Cryptosporidium_andersoni30847__24236
Rhytidocystis_sp__9409
Rhytidocystis_sp2__5389
Rhytidocystis_sp2__22452
Rhytidocystis_sp2__12359
Nephromyces_occidentalis__48981
Nephromyces_occidentalis__13814
Nephromyces_occidentalis__63115
Nephromyces_occidentalis__54695
Nephromyces_occidentalis__12225
Nephromyces_occidentalis__76892
Nephromyces_occidentalis__34634
Nephromyces_occidentalis__18243
Nephromyces_occidentalis__6985
Nephromyces_occidentalis__10052
Eimeria_falciformisBayerHaberkorn1970__153883
Nephromyces_occidentalis__64324
Nephromyces_occidentalis__10839
Nephromyces_occidentalis__45580
Nephromyces_occidentalis__12764
Nephromyces_occidentalis__71268
vbra_Vbra_23050___organismVitrella_brassicaformis_CCMP3155___hypothetical_protein
Vitrella_brassicaformis__18052
Piridium__686
Digyalum_oweni1__37749
Digyalum_oweni2__13144
Platyproteum_sp__13015
Colpodella_angusta2__78133
Colpodella_angusta2__78137
Colpodella_angusta1__38112
Colpodella_angusta2__78122
Colpodella_angusta1__38114
Colpodella_angusta2__78136
Colpodella_angusta2__78141
Colpodella_angusta2__78130
Colpodella_angusta2__78120
Colpodella_angusta2__78115
Colpodella_angusta2__78126
Colpodella_angusta2__78117
Colpodella_angusta2__78128
Colpodella_angusta2__78134
Colpodella_angusta2__78139
Symbiont_X__5858
Symbiont_X__6619
Alphamonas_edax__11278
Alphamonas_edax__11276
Alphamonas_edax__11275
Alphamonas_edax__11277
Eimeria_brunettiHoughton__387030
Alphamonas_edax__49121
cvel_Cvel_16485___organismChromera_velia_CCMP2878___hypothetical_protein
Chromera_velia__3004
0.3

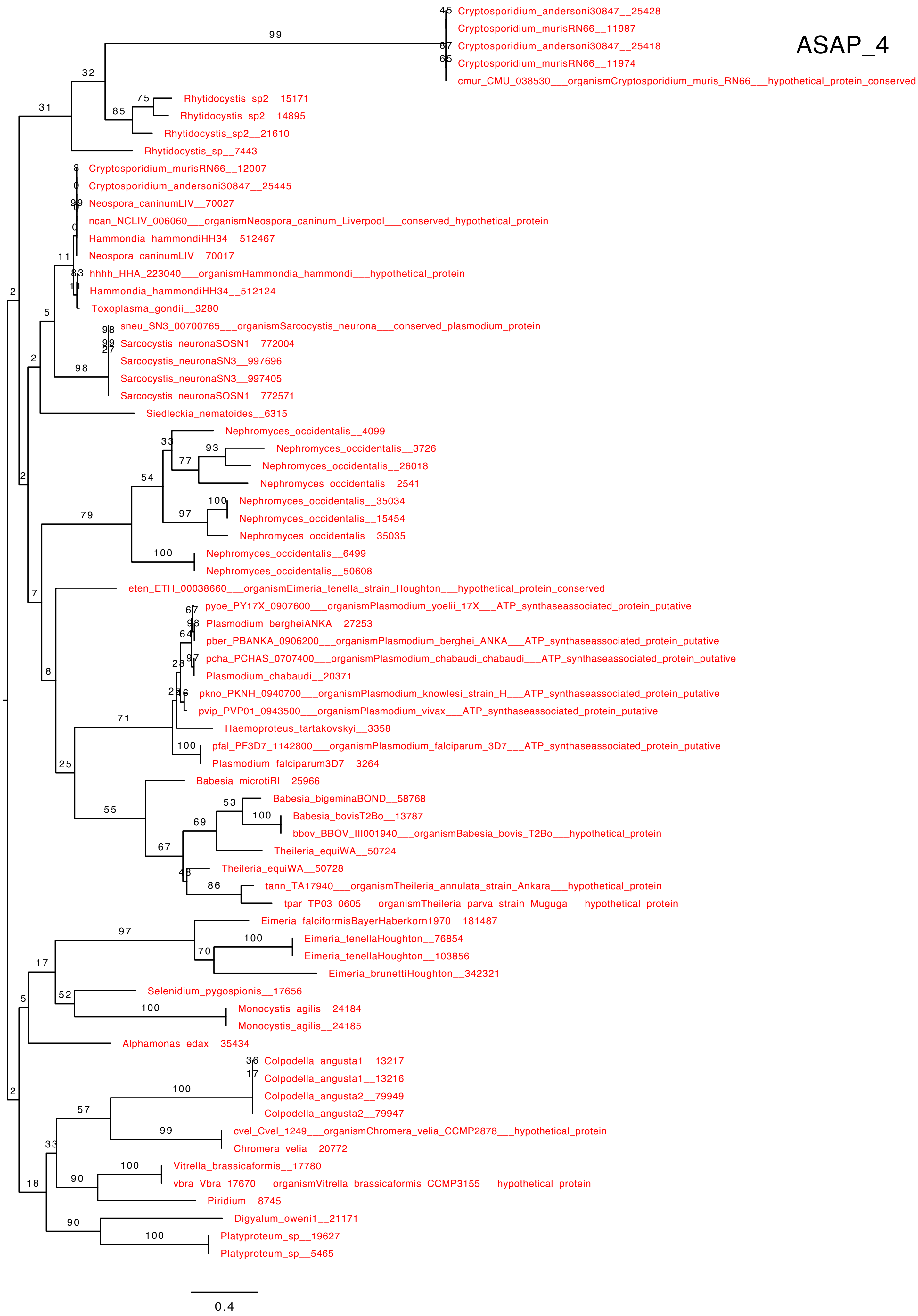
ASAP_4
Cryptosporidium_andersoni30847__25428
Cryptosporidium_murisRN66__11987
Cryptosporidium_andersoni30847__25418
Cryptosporidium_murisRN66__11974
cmur_CMU_038530___organismCryptosporidium_muris_RN66___hypothetical_protein_conserved
Rhytidocystis_sp2__15171
Rhytidocystis_sp2__14895
Rhytidocystis_sp2__21610
Rhytidocystis_sp__7443
Cryptosporidium_murisRN66__12007
Cryptosporidium_andersoni30847__25445
Neospora_caninumLIV__70027
ncan_NCLIV_006060___organismNeospora_caninum_Liverpool___conserved_hypothetical_protein
Hammondia_hammondiHH34__512467
Neospora_caninumLIV__70017
hhhh_HHA_223040___organismHammondia_hammondi___hypothetical_protein
Hammondia_hammondiHH34__512124
Toxoplasma_gondii__3280
sneu_SN3_00700765___organismSarcocystis_neurona___conserved_plasmodium_protein
Sarcocystis_neuronaSOSN1__772004
Sarcocystis_neuronaSN3__997696
Sarcocystis_neuronaSN3__997405
Sarcocystis_neuronaSOSN1__772571
Siedleckia_nematoides__6315
Nephromyces_occidentalis__4099
Nephromyces_occidentalis__3726
Nephromyces_occidentalis__26018
Nephromyces_occidentalis__2541
Nephromyces_occidentalis__35034
Nephromyces_occidentalis__15454
Nephromyces_occidentalis__35035
Nephromyces_occidentalis__6499
Nephromyces_occidentalis__50608
eten_ETH_00038660___organismEimeria_tenella_strain_Houghton___hypothetical_protein_conserved
pyoe_PY17X_0907600___organismPlasmodium_yoelii_17X___ATP_synthaseassociated_protein_putative
Plasmodium_bergheiANKA__27253
pber_PBANKA_0906200___organismPlasmodium_berghei_ANKA___ATP_synthaseassociated_protein_putative
pcha_PCHAS_0707400___organismPlasmodium_chabaudi_chabaudi___ATP_synthaseassociated_protein_putative
Plasmodium_chabaudi__20371
pkno_PKNH_0940700___organismPlasmodium_knowlesi_strain_H___ATP_synthaseassociated_protein_putative
pvip_PVP01_0943500___organismPlasmodium_vivax___ATP_synthaseassociated_protein_putative
Haemoproteus_tartakovskyi__3358
pfal_PF3D7_1142800___organismPlasmodium_falciparum_3D7___ATP_synthaseassociated_protein_putative
Plasmodium_falciparum3D7__3264
Babesia_microtiRI__25966
Babesia_bigeminaBOND__58768
Babesia_bovisT2Bo__13787
bbov_BBOV_III001940___organismBabesia_bovis_T2Bo___hypothetical_protein
Theileria_equiWA__50724
Theileria_equiWA__50728
tann_TA17940___organismTheileria_annulata_strain_Ankara___hypothetical_protein
tpar_TP03_0605___organismTheileria_parva_strain_Muguga___hypothetical_protein
Eimeria_falciformisBayerHaberkorn1970__181487
Eimeria_tenellaHoughton__76854
Eimeria_tenellaHoughton__103856
Eimeria_brunettiHoughton__342321
Selenidium_pygospionis__17656
Monocystis_agilis__24184
Monocystis_agilis__24185
Alphamonas_edax__35434
Colpodella_angusta1__13217
Colpodella_angusta1__13216
Colpodella_angusta2__79949
Colpodella_angusta2__79947
cvel_Cvel_1249___organismChromera_velia_CCMP2878___hypothetical_protein
Chromera_velia__20772
Vitrella_brassicaformis__17780
vbra_Vbra_17670___organismVitrella_brassicaformis_CCMP3155___hypothetical_protein
Piridium__8745
Digyalum_oweni1__21171
Platyproteum_sp__19627
Platyproteum_sp__5465
0.4

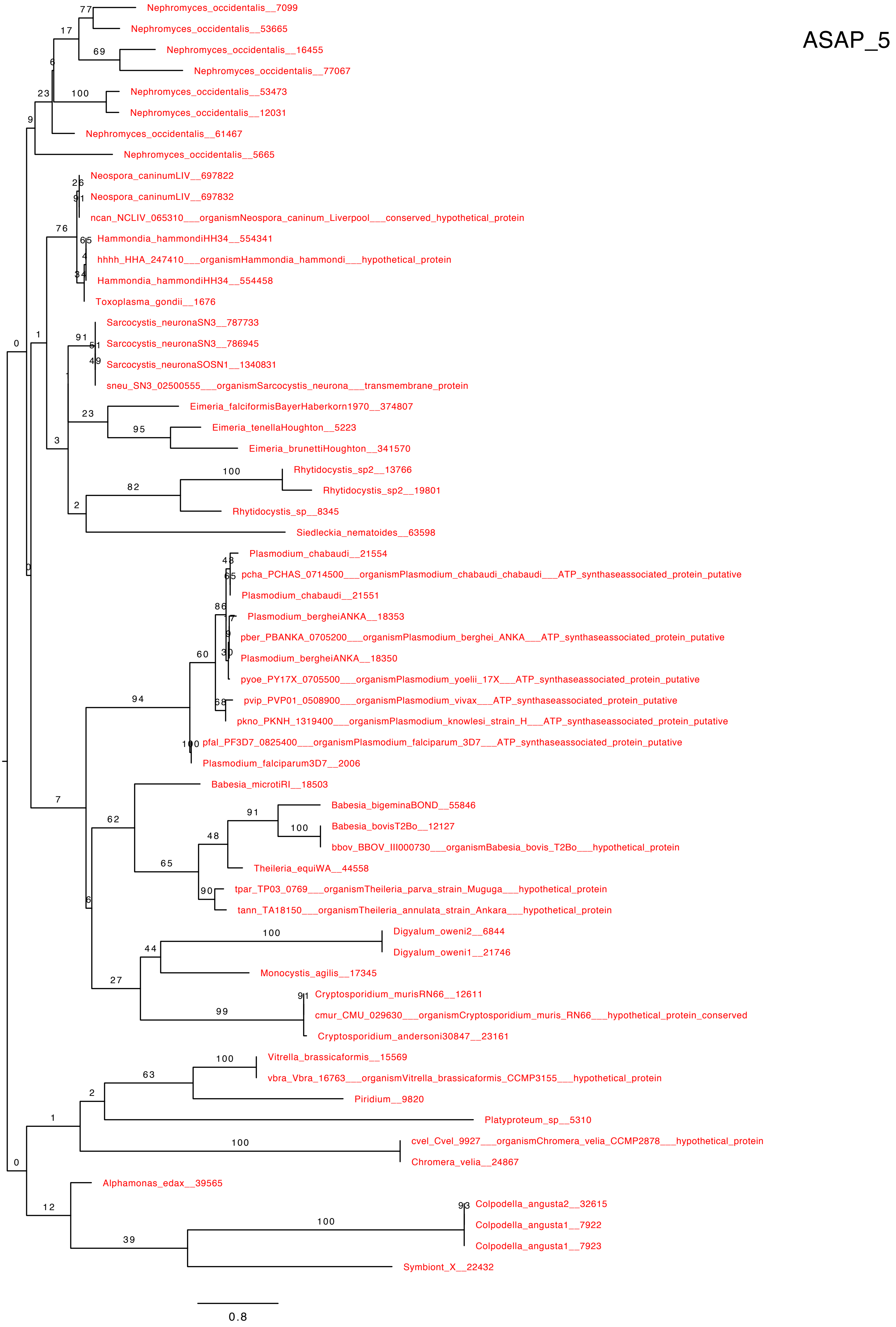
ASAP_5
Nephromyces_occidentalis__7099
Nephromyces_occidentalis__53665
Nephromyces_occidentalis__16455
Nephromyces_occidentalis__77067
Nephromyces_occidentalis__53473
Nephromyces_occidentalis__12031
Nephromyces_occidentalis__61467
Nephromyces_occidentalis__5665
Neospora_caninumLIV__697822
Neospora_caninumLIV__697832
ncan_NCLIV_065310___organismNeospora_caninum_Liverpool___conserved_hypothetical_protein
Hammondia_hammondiHH34__554341
hhhh_HHA_247410___organismHammondia_hammondi___hypothetical_protein
Hammondia_hammondiHH34__554458
Toxoplasma_gondii__1676
Sarcocystis_neuronaSN3__787733
Sarcocystis_neuronaSN3__786945
Sarcocystis_neuronaSOSN1__1340831
sneu_SN3_02500555___organismSarcocystis_neurona___transmembrane_protein
Eimeria_falciformisBayerHaberkorn1970__374807
Eimeria_tenellaHoughton__5223
Eimeria_brunettiHoughton__341570
Rhytidocystis_sp2__13766
Rhytidocystis_sp2__19801
Rhytidocystis_sp__8345
Siedleckia_nematoides__63598
Plasmodium_chabaudi__21554
pcha_PCHAS_0714500___organismPlasmodium_chabaudi_chabaudi___ATP_synthaseassociated_protein_putative
Plasmodium_chabaudi__21551
Plasmodium_bergheiANKA__18353
pber_PBANKA_0705200___organismPlasmodium_berghei_ANKA___ATP_synthaseassociated_protein_putative
Plasmodium_bergheiANKA__18350
pyoe_PY17X_0705500___organismPlasmodium_yoelii_17X___ATP_synthaseassociated_protein_putative
pvip_PVP01_0508900___organismPlasmodium_vivax___ATP_synthaseassociated_protein_putative
pkno_PKNH_1319400___organismPlasmodium_knowlesi_strain_H___ATP_synthaseassociated_protein_putative
pfal_PF3D7_0825400___organismPlasmodium_falciparum_3D7___ATP_synthaseassociated_protein_putative
Plasmodium_falciparum3D7__2006
Babesia_microtiRI__18503
Babesia_bigeminaBOND__55846
Babesia_bovisT2Bo__12127
bbov_BBOV_III000730___organismBabesia_bovis_T2Bo___hypothetical_protein
Theileria_equiWA__44558
tpar_TP03_0769___organismTheileria_parva_strain_Muguga___hypothetical_protein
tann_TA18150___organismTheileria_annulata_strain_Ankara___hypothetical_protein
Digyalum_oweni2__6844
Digyalum_oweni1__21746
Monocystis_agilis__17345
Cryptosporidium_murisRN66__12611
cmur_CMU_029630___organismCryptosporidium_muris_RN66___hypothetical_protein_conserved
Cryptosporidium_andersoni30847__23161
Vitrella_brassicaformis__15569
vbra_Vbra_16763___organismVitrella_brassicaformis_CCMP3155___hypothetical_protein
Piridium__9820
Platyproteum_sp__5310
cvel_Cvel_9927___organismChromera_velia_CCMP2878___hypothetical_protein
Chromera_velia__24867
Alphamonas_edax__39565
Colpodella_angusta2__32615
Colpodella_angusta1__7922
Colpodella_angusta1__7923
Symbiont_X__22432
0.8

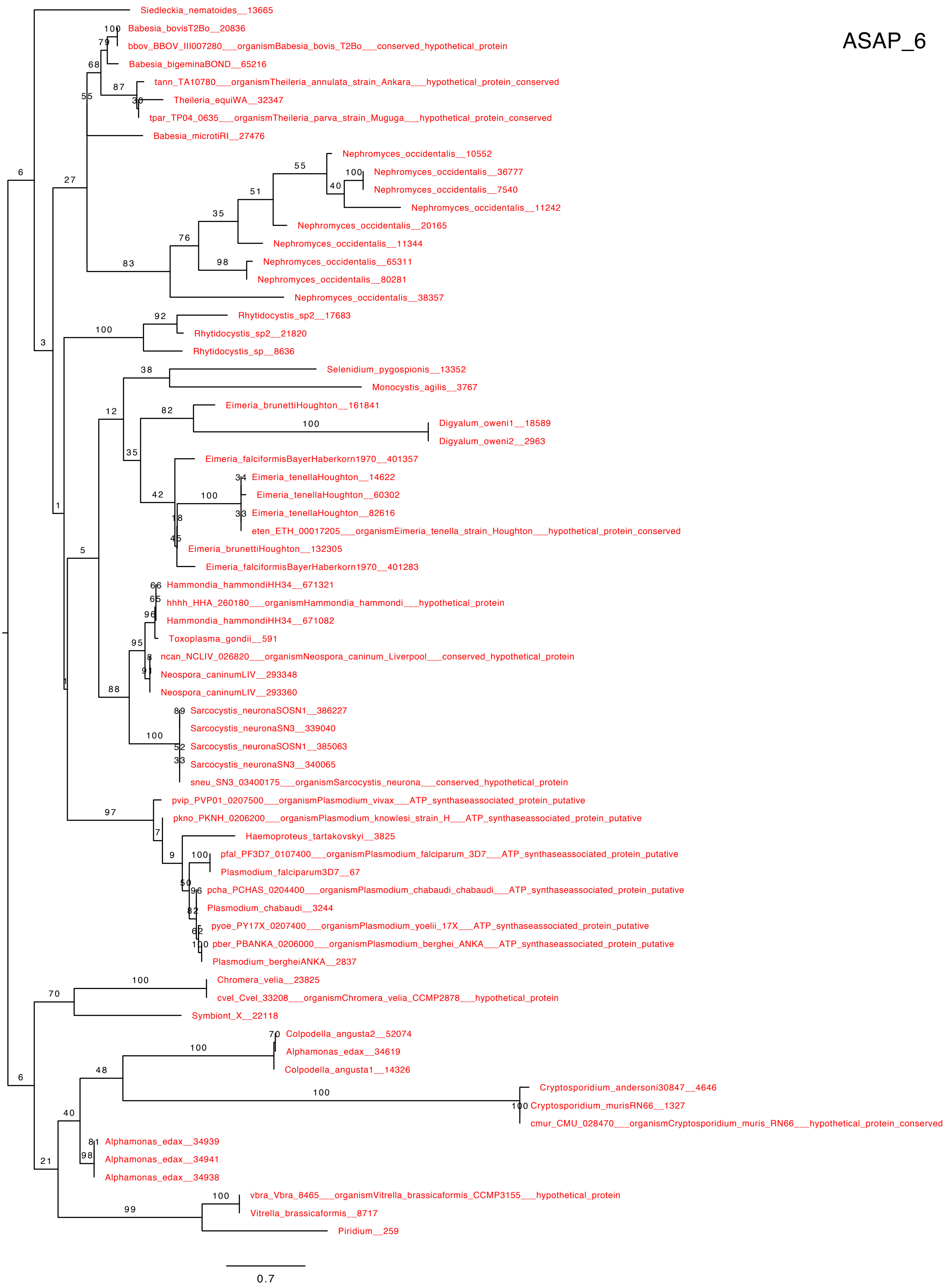
ASAP_6
Siedleckia_nematoides__13665
Babesia_bovisT2Bo__20836
bbov_BBOV_III007280___organismBabesia_bovis_T2Bo___conserved_hypothetical_protein
Babesia_bigeminaBOND__65216
tann_TA10780___organismTheileria_annulata_strain_Ankara___hypothetical_protein_conserved
Theileria_equiWA__32347
tpar_TP04_0635___organismTheileria_parva_strain_Muguga___hypothetical_protein_conserved
Babesia_microtiRI__27476
Nephromyces_occidentalis__10552
Nephromyces_occidentalis__36777
Nephromyces_occidentalis__7540
Nephromyces_occidentalis__11242
Nephromyces_occidentalis__20165
Nephromyces_occidentalis__11344
Nephromyces_occidentalis__65311
Nephromyces_occidentalis__80281
Nephromyces_occidentalis__38357
Rhytidocystis_sp2__17683
Rhytidocystis_sp2__21820
Rhytidocystis_sp__8636
Selenidium_pygospionis__13352
Monocystis_agilis__3767
Eimeria_brunettiHoughton__161841
Digyalum_oweni1__18589
Digyalum_oweni2__2963
Eimeria_falciformisBayerHaberkorn1970__401357
Eimeria_tenellaHoughton__14622
Eimeria_tenellaHoughton__60302
Eimeria_tenellaHoughton__82616
eten_ETH_00017205___organismEimeria_tenella_strain_Houghton___hypothetical_protein_conserved
Eimeria_brunettiHoughton__132305
Eimeria_falciformisBayerHaberkorn1970__401283
Hammondia_hammondiHH34__671321
hhhh_HHA_260180___organismHammondia_hammondi___hypothetical_protein
Hammondia_hammondiHH34__671082
Toxoplasma_gondii__591
ncan_NCLIV_026820___organismNeospora_caninum_Liverpool___conserved_hypothetical_protein
Neospora_caninumLIV__293348
Neospora_caninumLIV__293360
Sarcocystis_neuronaSOSN1__386227
Sarcocystis_neuronaSN3__339040
Sarcocystis_neuronaSOSN1__385063
Sarcocystis_neuronaSN3__340065
sneu_SN3_03400175___organismSarcocystis_neurona___conserved_hypothetical_protein
pvip_PVP01_0207500___organismPlasmodium_vivax___ATP_synthaseassociated_protein_putative
pkno_PKNH_0206200___organismPlasmodium_knowlesi_strain_H___ATP_synthaseassociated_protein_putative
Haemoproteus_tartakovskyi__3825
pfal_PF3D7_0107400___organismPlasmodium_falciparum_3D7___ATP_synthaseassociated_protein_putative
Plasmodium_falciparum3D7__67
pcha_PCHAS_0204400___organismPlasmodium_chabaudi_chabaudi___ATP_synthaseassociated_protein_putative
Plasmodium_chabaudi__3244
pyoe_PY17X_0207400___organismPlasmodium_yoelii_17X___ATP_synthaseassociated_protein_putative
pber_PBANKA_0206000___organismPlasmodium_berghei_ANKA___ATP_synthaseassociated_protein_putative
Plasmodium_bergheiANKA__2837
Chromera_velia__23825
cvel_Cvel_33208___organismChromera_velia_CCMP2878___hypothetical_protein
Symbiont_X__22118
Colpodella_angusta2__52074
Alphamonas_edax__34619
Colpodella_angusta1__14326
Cryptosporidium_andersoni30847__4646
Cryptosporidium_murisRN66__1327
cmur_CMU_028470___organismCryptosporidium_muris_RN66___hypothetical_protein_conserved
Alphamonas_edax__34939
Alphamonas_edax__34941
Alphamonas_edax__34938
vbra_Vbra_8465___organismVitrella_brassicaformis_CCMP3155___hypothetical_protein
Vitrella_brassicaformis__8717
Piridium__259
0.7

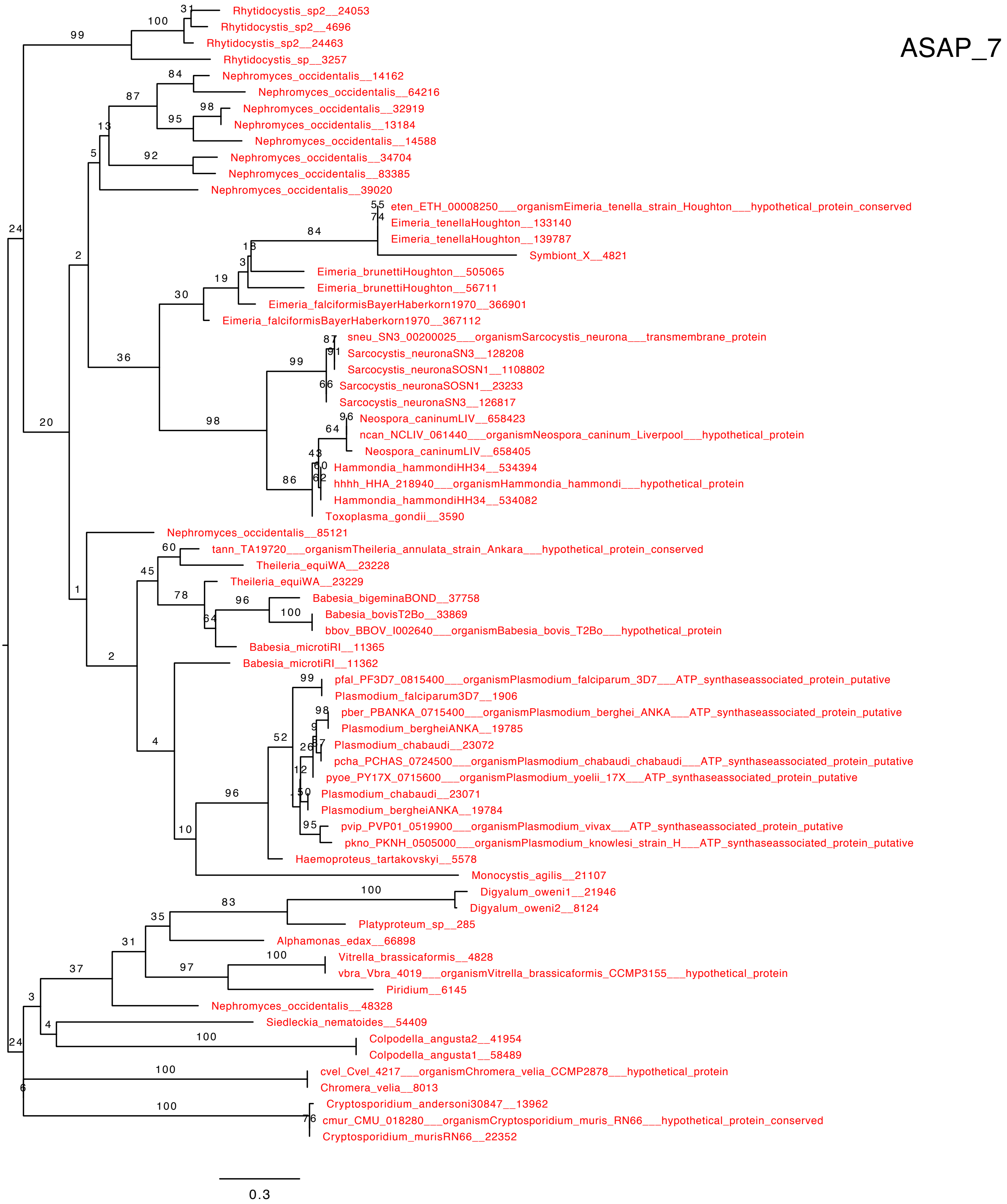
ASAP_7
Rhytidocystis_sp2__24053
Rhytidocystis_sp2__4696
Rhytidocystis_sp2__24463
Rhytidocystis_sp__3257
Nephromyces_occidentalis__14162
Nephromyces_occidentalis__64216
Nephromyces_occidentalis__32919
Nephromyces_occidentalis__13184
Nephromyces_occidentalis__14588
Nephromyces_occidentalis__34704
Nephromyces_occidentalis__83385
Nephromyces_occidentalis__39020
eten_ETH_00008250___organismEimeria_tenella_strain_Houghton___hypothetical_protein_conserved
Eimeria_tenellaHoughton__133140
Eimeria_tenellaHoughton__139787
Symbiont_X__4821
Eimeria_brunettiHoughton__505065
Eimeria_brunettiHoughton__56711
Eimeria_falciformisBayerHaberkorn1970__366901
Eimeria_falciformisBayerHaberkorn1970__367112
sneu_SN3_00200025___organismSarcocystis_neurona___transmembrane_protein
Sarcocystis_neuronaSN3__128208
Sarcocystis_neuronaSOSN1__1108802
Sarcocystis_neuronaSOSN1__23233
Sarcocystis_neuronaSN3__126817
Neospora_caninumLIV__658423
ncan_NCLIV_061440___organismNeospora_caninum_Liverpool___hypothetical_protein
Neospora_caninumLIV__658405
Hammondia_hammondiHH34__534394
hhhh_HHA_218940___organismHammondia_hammondi___hypothetical_protein
Hammondia_hammondiHH34__534082
Toxoplasma_gondii__3590
Nephromyces_occidentalis__85121
tann_TA19720___organismTheileria_annulata_strain_Ankara___hypothetical_protein_conserved
Theileria_equiWA__23228
Theileria_equiWA__23229
Babesia_bigeminaBOND__37758
Babesia_bovisT2Bo__33869
bbov_BBOV_I002640___organismBabesia_bovis_T2Bo___hypothetical_protein
Babesia_microtiRI__11365
Babesia_microtiRI__11362
pfal_PF3D7_0815400___organismPlasmodium_falciparum_3D7___ATP_synthaseassociated_protein_putative
Plasmodium_falciparum3D7__1906
pber_PBANKA_0715400___organismPlasmodium_berghei_ANKA___ATP_synthaseassociated_protein_putative
Plasmodium_bergheiANKA__19785
Plasmodium_chabaudi__23072
pcha_PCHAS_0724500___organismPlasmodium_chabaudi_chabaudi___ATP_synthaseassociated_protein_putative
pyoe_PY17X_0715600___organismPlasmodium_yoelii_17X___ATP_synthaseassociated_protein_putative
Plasmodium_chabaudi__23071
Plasmodium_bergheiANKA__19784
pvip_PVP01_0519900___organismPlasmodium_vivax___ATP_synthaseassociated_protein_putative
pkno_PKNH_0505000___organismPlasmodium_knowlesi_strain_H___ATP_synthaseassociated_protein_putative
Haemoproteus_tartakovskyi__5578
Monocystis_agilis__21107
Digyalum_oweni1__21946
Digyalum_oweni2__8124
Platyproteum_sp__285
Alphamonas_edax__66898
Vitrella_brassicaformis__4828
vbra_Vbra_4019___organismVitrella_brassicaformis_CCMP3155___hypothetical_protein
Piridium__6145
Nephromyces_occidentalis__48328
Siedleckia_nematoides__54409
Colpodella_angusta2__41954
Colpodella_angusta1__58489
cvel_Cvel_4217___organismChromera_velia_CCMP2878___hypothetical_protein
Chromera_velia__8013
Cryptosporidium_andersoni30847__13962
cmur_CMU_018280___organismCryptosporidium_muris_RN66___hypothetical_protein_conserved
Cryptosporidium_murisRN66__22352
0.3

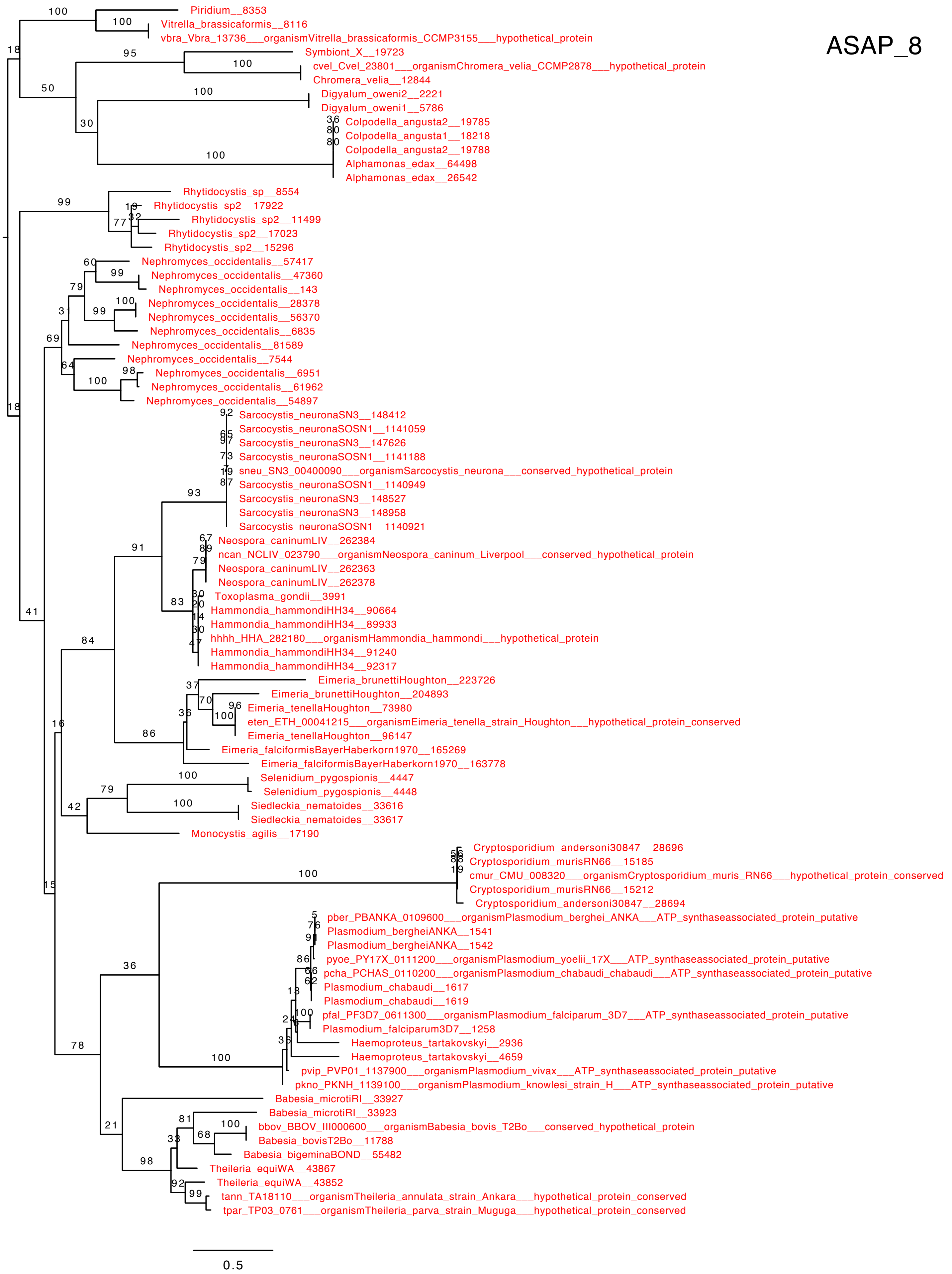
ASAP_8
Piridium__8353
Vitrella_brassicaformis__8116
vbra_Vbra_13736___organismVitrella_brassicaformis_CCMP3155___hypothetical_protein
Symbiont_X__19723
cvel_Cvel_23801___organismChromera_velia_CCMP2878___hypothetical_protein
Chromera_velia__12844
Digyalum_oweni2__2221
Digyalum_oweni1__5786
Colpodella_angusta2__19785
Colpodella_angusta1__18218
Colpodella_angusta2__19788
Alphamonas_edax__64498
Alphamonas_edax__26542
Rhytidocystis_sp__8554
Rhytidocystis_sp2__17922
Rhytidocystis_sp2__11499
Rhytidocystis_sp2__17023
Rhytidocystis_sp2__15296
Nephromyces_occidentalis__57417
Nephromyces_occidentalis__47360
Nephromyces_occidentalis__143
Nephromyces_occidentalis__28378
Nephromyces_occidentalis__56370
Nephromyces_occidentalis__6835
Nephromyces_occidentalis__81589
Nephromyces_occidentalis__7544
Nephromyces_occidentalis__6951
Nephromyces_occidentalis__61962
Nephromyces_occidentalis__54897
Sarcocystis_neuronaSN3__148412
Sarcocystis_neuronaSOSN1__1141059
Sarcocystis_neuronaSN3__147626
Sarcocystis_neuronaSOSN1__1141188
sneu_SN3_00400090___organismSarcocystis_neurona___conserved_hypothetical_protein
Sarcocystis_neuronaSOSN1__1140949
Sarcocystis_neuronaSN3__148527
Sarcocystis_neuronaSN3__148958
Sarcocystis_neuronaSOSN1__1140921
Neospora_caninumLIV__262384
ncan_NCLIV_023790___organismNeospora_caninum_Liverpool___conserved_hypothetical_protein
Neospora_caninumLIV__262363
Neospora_caninumLIV__262378
Toxoplasma_gondii__3991
Hammondia_hammondiHH34__90664
Hammondia_hammondiHH34__89933
hhhh_HHA_282180___organismHammondia_hammondi___hypothetical_protein
Hammondia_hammondiHH34__91240
Hammondia_hammondiHH34__92317
Eimeria_brunettiHoughton__223726
Eimeria_brunettiHoughton__204893
Eimeria_tenellaHoughton__73980
eten_ETH_00041215___organismEimeria_tenella_strain_Houghton___hypothetical_protein_conserved
Eimeria_tenellaHoughton__96147
Eimeria_falciformisBayerHaberkorn1970__165269
Eimeria_falciformisBayerHaberkorn1970__163778
Selenidium_pygospionis__4447
Selenidium_pygospionis__4448
Siedleckia_nematoides__33616
Siedleckia_nematoides__33617
Monocystis_agilis__17190
Cryptosporidium_andersoni30847__28696
Cryptosporidium_murisRN66__15185
cmur_CMU_008320___organismCryptosporidium_muris_RN66___hypothetical_protein_conserved
Cryptosporidium_murisRN66__15212
Cryptosporidium_andersoni30847__28694
pber_PBANKA_0109600___organismPlasmodium_berghei_ANKA___ATP_synthaseassociated_protein_putative
Plasmodium_bergheiANKA__1541
Plasmodium_bergheiANKA__1542
pyoe_PY17X_0111200___organismPlasmodium_yoelii_17X___ATP_synthaseassociated_protein_putative
pcha_PCHAS_0110200___organismPlasmodium_chabaudi_chabaudi___ATP_synthaseassociated_protein_putative
Plasmodium_chabaudi__1617
Plasmodium_chabaudi__1619
pfal_PF3D7_0611300___organismPlasmodium_falciparum_3D7___ATP_synthaseassociated_protein_putative
Plasmodium_falciparum3D7__1258
Haemoproteus_tartakovskyi__2936
Haemoproteus_tartakovskyi__4659
pvip_PVP01_1137900___organismPlasmodium_vivax___ATP_synthaseassociated_protein_putative
pkno_PKNH_1139100___organismPlasmodium_knowlesi_strain_H___ATP_synthaseassociated_protein_putative
Babesia_microtiRI__33927
Babesia_microtiRI__33923
bbov_BBOV_III000600___organismBabesia_bovis_T2Bo___conserved_hypothetical_protein
Babesia_bovisT2Bo__11788
Babesia_bigeminaBOND__55482
Theileria_equiWA__43867
Theileria_equiWA__43852
tann_TA18110___organismTheileria_annulata_strain_Ankara___hypothetical_protein_conserved
tpar_TP03_0761___organismTheileria_parva_strain_Muguga___hypothetical_protein_conserved
0.5

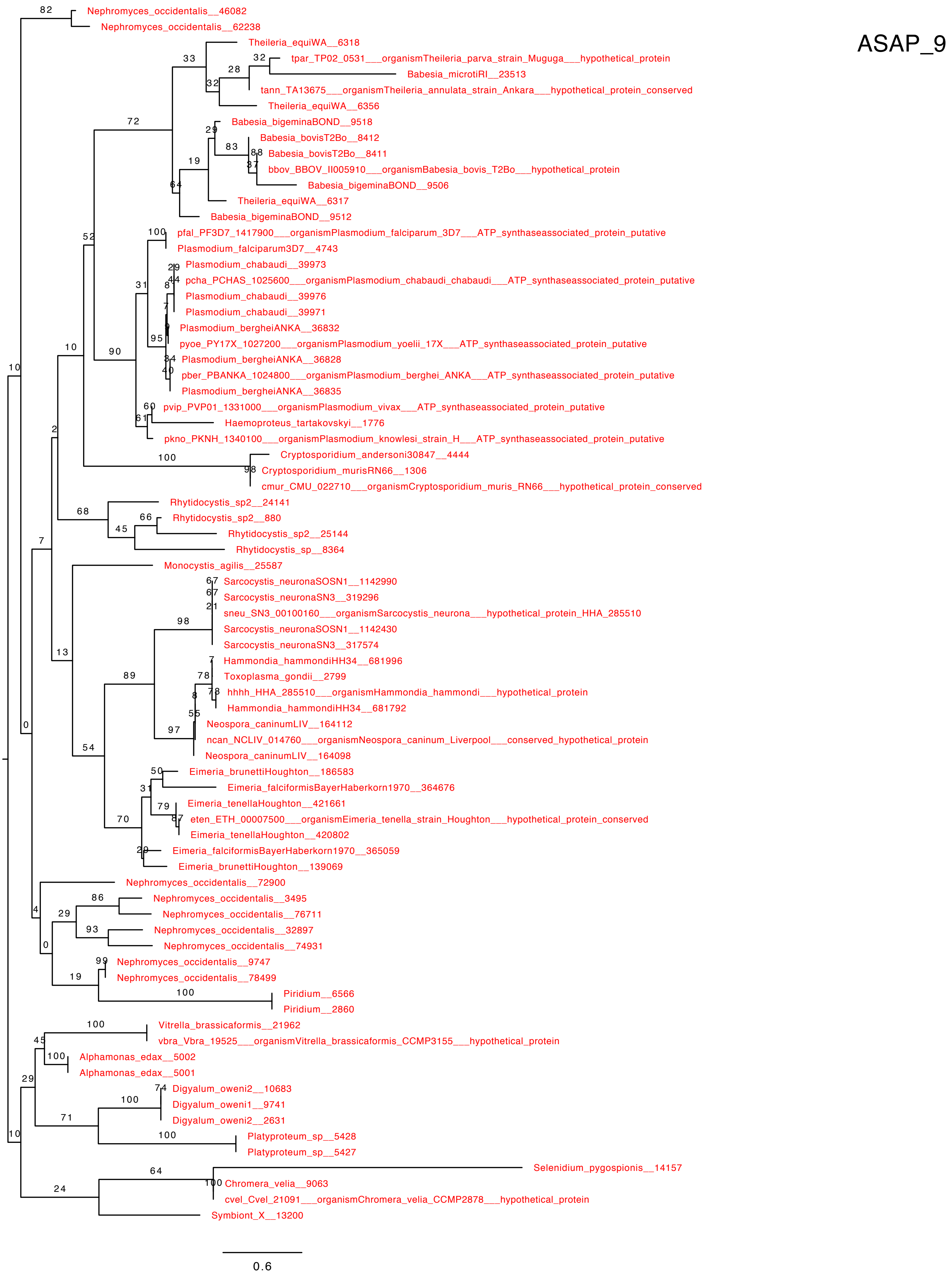
ASAP_9
Nephromyces_occidentalis__46082
Nephromyces_occidentalis__62238
Theileria_equiWA__6318
tpar_TP02_0531___organismTheileria_parva_strain_Muguga___hypothetical_protein
Babesia_microtiRI__23513
tann_TA13675___organismTheileria_annulata_strain_Ankara___hypothetical_protein_conserved
Theileria_equiWA__6356
Babesia_bigeminaBOND__9518
Babesia_bovisT2Bo__8412
Babesia_bovisT2Bo__8411
bbov_BBOV_II005910___organismBabesia_bovis_T2Bo___hypothetical_protein
Babesia_bigeminaBOND__9506
Theileria_equiWA__6317
Babesia_bigeminaBOND__9512
pfal_PF3D7_1417900___organismPlasmodium_falciparum_3D7___ATP_synthaseassociated_protein_putative
Plasmodium_falciparum3D7__4743
Plasmodium_chabaudi__39973
pcha_PCHAS_1025600___organismPlasmodium_chabaudi_chabaudi___ATP_synthaseassociated_protein_putative
Plasmodium_chabaudi__39976
Plasmodium_chabaudi__39971
Plasmodium_bergheiANKA__36832
pyoe_PY17X_1027200___organismPlasmodium_yoelii_17X___ATP_synthaseassociated_protein_putative
Plasmodium_bergheiANKA__36828
pber_PBANKA_1024800___organismPlasmodium_berghei_ANKA___ATP_synthaseassociated_protein_putative
Plasmodium_bergheiANKA__36835
pvip_PVP01_1331000___organismPlasmodium_vivax___ATP_synthaseassociated_protein_putative
Haemoproteus_tartakovskyi__1776
pkno_PKNH_1340100___organismPlasmodium_knowlesi_strain_H___ATP_synthaseassociated_protein_putative
Cryptosporidium_andersoni30847__4444
Cryptosporidium_murisRN66__1306
cmur_CMU_022710___organismCryptosporidium_muris_RN66___hypothetical_protein_conserved
Rhytidocystis_sp2__24141
Rhytidocystis_sp2__880
Rhytidocystis_sp2__25144
Rhytidocystis_sp__8364
Monocystis_agilis__25587
Sarcocystis_neuronaSOSN1__1142990
Sarcocystis_neuronaSN3__319296
sneu_SN3_00100160___organismSarcocystis_neurona___hypothetical_protein_HHA_285510
Sarcocystis_neuronaSOSN1__1142430
Sarcocystis_neuronaSN3__317574
Hammondia_hammondiHH34__681996
Toxoplasma_gondii__2799
hhhh_HHA_285510___organismHammondia_hammondi___hypothetical_protein
Hammondia_hammondiHH34__681792
Neospora_caninumLIV__164112
ncan_NCLIV_014760___organismNeospora_caninum_Liverpool___conserved_hypothetical_protein
Neospora_caninumLIV__164098
Eimeria_brunettiHoughton__186583
Eimeria_falciformisBayerHaberkorn1970__364676
Eimeria_tenellaHoughton__421661
eten_ETH_00007500___organismEimeria_tenella_strain_Houghton___hypothetical_protein_conserved
Eimeria_tenellaHoughton__420802
Eimeria_falciformisBayerHaberkorn1970__365059
Eimeria_brunettiHoughton__139069
Nephromyces_occidentalis__72900
Nephromyces_occidentalis__3495
Nephromyces_occidentalis__76711
Nephromyces_occidentalis__32897
Nephromyces_occidentalis__74931
Nephromyces_occidentalis__9747
Nephromyces_occidentalis__78499
Piridium__6566
Piridium__2860
Vitrella_brassicaformis__21962
vbra_Vbra_19525___organismVitrella_brassicaformis_CCMP3155___hypothetical_protein
Alphamonas_edax__5002
Alphamonas_edax__5001
Digyalum_oweni2__10683
Digyalum_oweni1__9741
Digyalum_oweni2__2631
Platyproteum_sp__5428
Platyproteum_sp__5427
Selenidium_pygospionis__14157
Chromera_velia__9063
cvel_Cvel_21091___organismChromera_velia_CCMP2878___hypothetical_protein
Symbiont_X__13200
0.6

AST

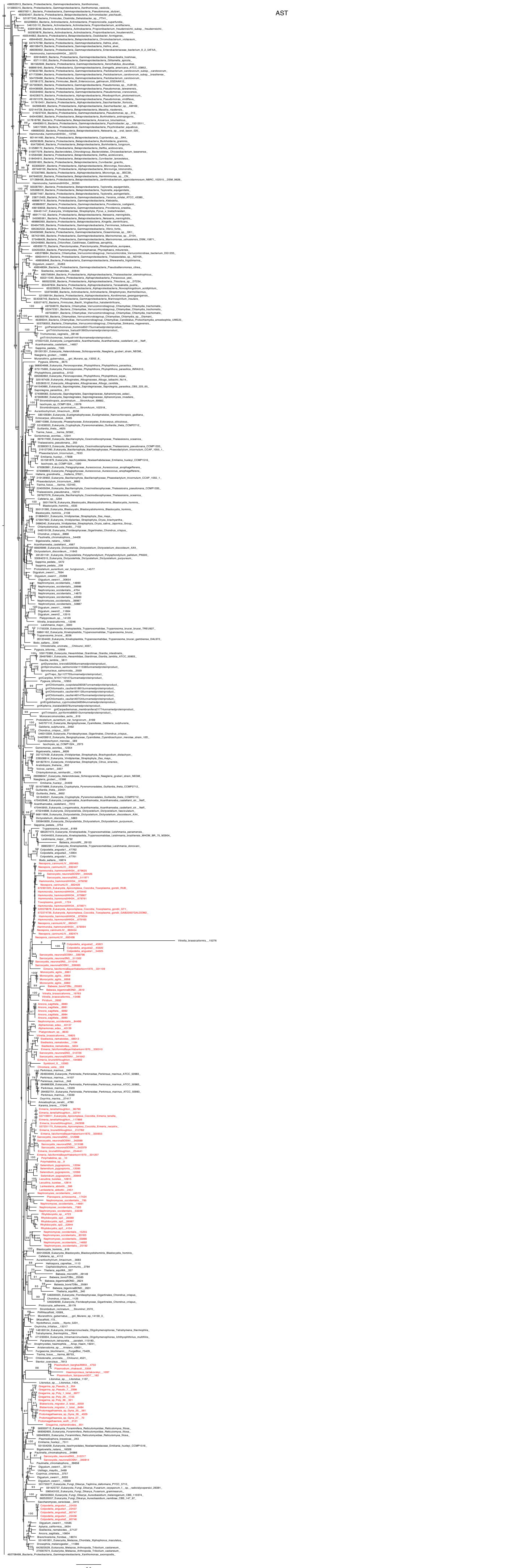

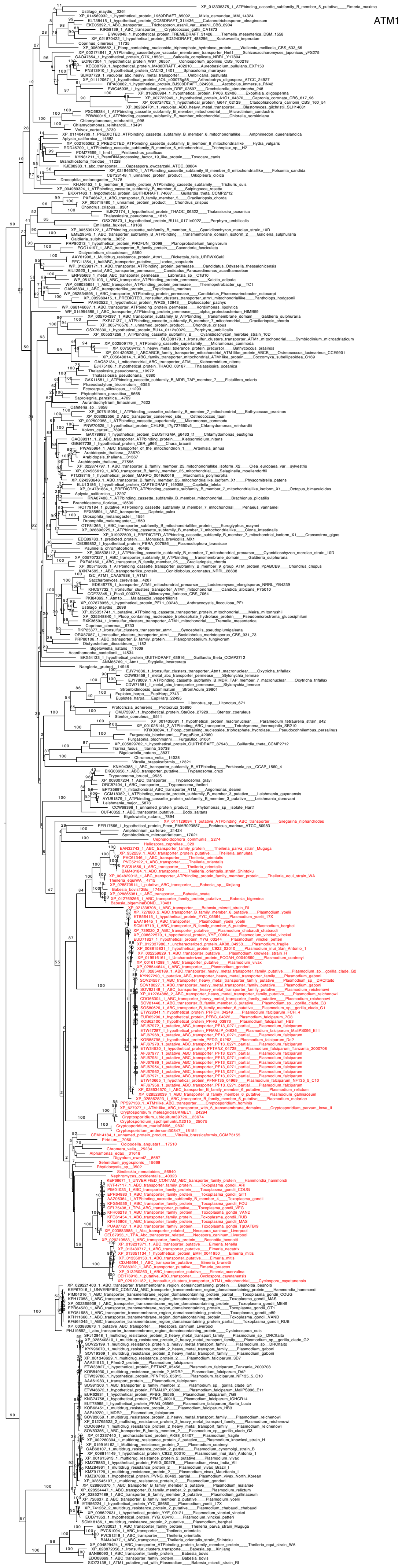
ATM1
0.8

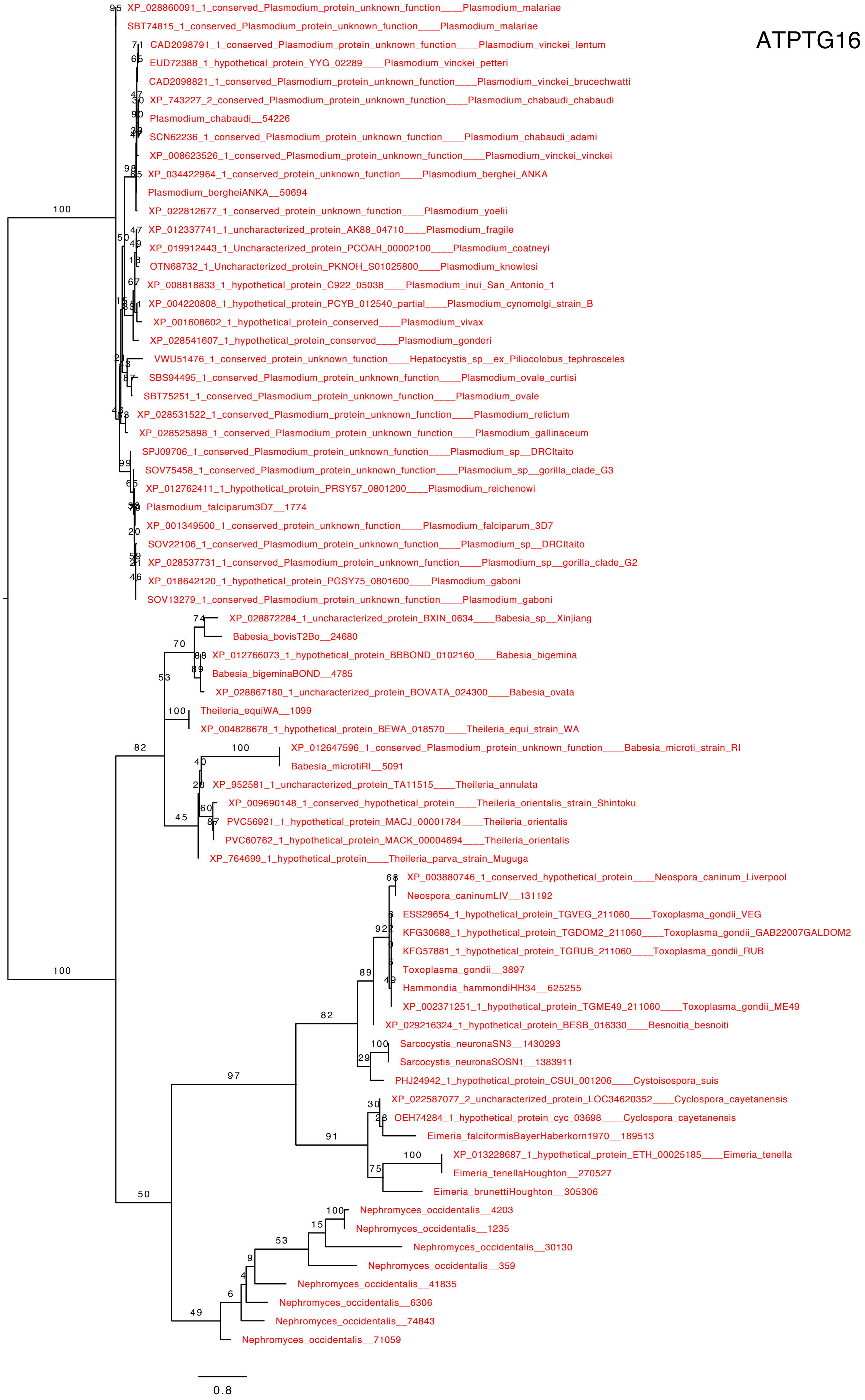
ATPTG16
XP_028860091_1_conserved_Plasmodium_protein_unknown_function____Plasmodium_malariae
SBT74815_1_conserved_Plasmodium_protein_unknown_function____Plasmodium_malariae
CAD2098791_1_conserved_Plasmodium_protein_unknown_function____Plasmodium_vinckei_lentum
EUD72388_1_hypothetical_protein_YYG_02289____Plasmodium_vinckei_petteri
CAD2098821_1_conserved_Plasmodium_protein_unknown_function____Plasmodium_vinckei_brucechwatti
XP_743227_2_conserved_Plasmodium_protein_unknown_function____Plasmodium_chabaudi_chabaudi
Plasmodium_chabaudi__54226
SCN62236_1_conserved_Plasmodium_protein_unknown_function____Plasmodium_chabaudi_adami
XP_008623526_1_conserved_Plasmodium_protein_unknown_function____Plasmodium_vinckei_vinckei
XP_034422964_1_conserved_protein_unknown_function____Plasmodium_berghei_ANKA
Plasmodium_bergheiANKA__50694
XP_022812677_1_conserved_protein_unknown_function____Plasmodium_yoelii
XP_012337741_1_uncharacterized_protein_AK88_04710____Plasmodium_fragile
XP_019912443_1_Uncharacterized_protein_PCOAH_00002100____Plasmodium_coatneyi
OTN68732_1_Uncharacterized_protein_PKNOH_S01025800____Plasmodium_knowlesi
XP_008818833_1_hypothetical_protein_C922_05038____Plasmodium_inui_San_Antonio_1
XP_004220808_1_hypothetical_protein_PCYB_012540_partial____Plasmodium_cynomolgi_strain_B
XP_001608602_1_hypothetical_protein_conserved____Plasmodium_vivax
XP_028541607_1_hypothetical_protein_conserved____Plasmodium_gonderi
VWU51476_1_conserved_protein_unknown_function____Hepatocystis_sp__ex_Piliocolobus_tephrosceles
SBS94495_1_conserved_Plasmodium_protein_unknown_function____Plasmodium_ovale_curtisi
SBT75251_1_conserved_Plasmodium_protein_unknown_function____Plasmodium_ovale
XP_028531522_1_conserved_Plasmodium_protein_unknown_function____Plasmodium_relictum
XP_028525898_1_conserved_Plasmodium_protein_unknown_function____Plasmodium_gallinaceum
SPJ09706_1_conserved_Plasmodium_protein_unknown_function____Plasmodium_sp__DRCItaito
SOV75458_1_conserved_Plasmodium_protein_unknown_function____Plasmodium_sp__gorilla_clade_G3
XP_012762411_1_hypothetical_protein_PRSY57_0801200____Plasmodium_reichenowi
Plasmodium_falciparum3D7__1774
XP_001349500_1_conserved_protein_unknown_function____Plasmodium_falciparum_3D7
SOV22106_1_conserved_Plasmodium_protein_unknown_function____Plasmodium_sp__DRCItaito
XP_028537731_1_conserved_Plasmodium_protein_unknown_function____Plasmodium_sp__gorilla_clade_G2
XP_018642120_1_hypothetical_protein_PGSY75_0801600____Plasmodium_gaboni
SOV13279_1_conserved_Plasmodium_protein_unknown_function____Plasmodium_gaboni
XP_028872284_1_uncharacterized_protein_BXIN_0634____Babesia_sp__Xinjiang
Babesia_bovisT2Bo__24680
XP_012766073_1_hypothetical_protein_BBBOND_0102160____Babesia_bigemina
Babesia_bigeminaBOND__4785
XP_028867180_1_uncharacterized_protein_BOVATA_024300____Babesia_ovata
Theileria_equiWA__1099
XP_004828678_1_hypothetical_protein_BEWA_018570____Theileria_equi_strain_WA
XP_012647596_1_conserved_Plasmodium_protein_unknown_function____Babesia_microti_strain_RI
Babesia_microtiRI__5091
XP_952581_1_uncharacterized_protein_TA11515____Theileria_annulata
XP_009690148_1_conserved_hypothetical_protein____Theileria_orientalis_strain_Shintoku
PVC56921_1_hypothetical_protein_MACJ_00001784____Theileria_orientalis
PVC60762_1_hypothetical_protein_MACK_00004694____Theileria_orientalis
XP_764699_1_hypothetical_protein____Theileria_parva_strain_Muguga
XP_003880746_1_conserved_hypothetical_protein____Neospora_caninum_Liverpool
Neospora_caninumLIV__131192
ESS29654_1_hypothetical_protein_TGVEG_211060____Toxoplasma_gondii_VEG
KFG30688_1_hypothetical_protein_TGDOM2_211060____Toxoplasma_gondii_GAB22007GALDOM2
KFG57881_1_hypothetical_protein_TGRUB_211060____Toxoplasma_gondii_RUB
Toxoplasma_gondii__3897
Hammondia_hammondiHH34__625255
XP_002371251_1_hypothetical_protein_TGME49_211060____Toxoplasma_gondii_ME49
XP_029216324_1_hypothetical_protein_BESB_016330____Besnoitia_besnoiti
Sarcocystis_neuronaSN3__1430293
Sarcocystis_neuronaSOSN1__1383911
PHJ24942_1_hypothetical_protein_CSUI_001206____Cystoisospora_suis
XP_022587077_2_uncharacterized_protein_LOC34620352____Cyclospora_cayetanensis
OEH74284_1_hypothetical_protein_cyc_03698____Cyclospora_cayetanensis
Eimeria_falciformisBayerHaberkorn1970__189513
XP_013228687_1_hypothetical_protein_ETH_00025185____Eimeria_tenella
Eimeria_tenellaHoughton__270527
Eimeria_brunettiHoughton__305306
Nephromyces_occidentalis__4203
Nephromyces_occidentalis__1235
Nephromyces_occidentalis__30130
Nephromyces_occidentalis__359
Nephromyces_occidentalis__41835
Nephromyces_occidentalis__6306
Nephromyces_occidentalis__74843
Nephromyces_occidentalis__71059
0.8

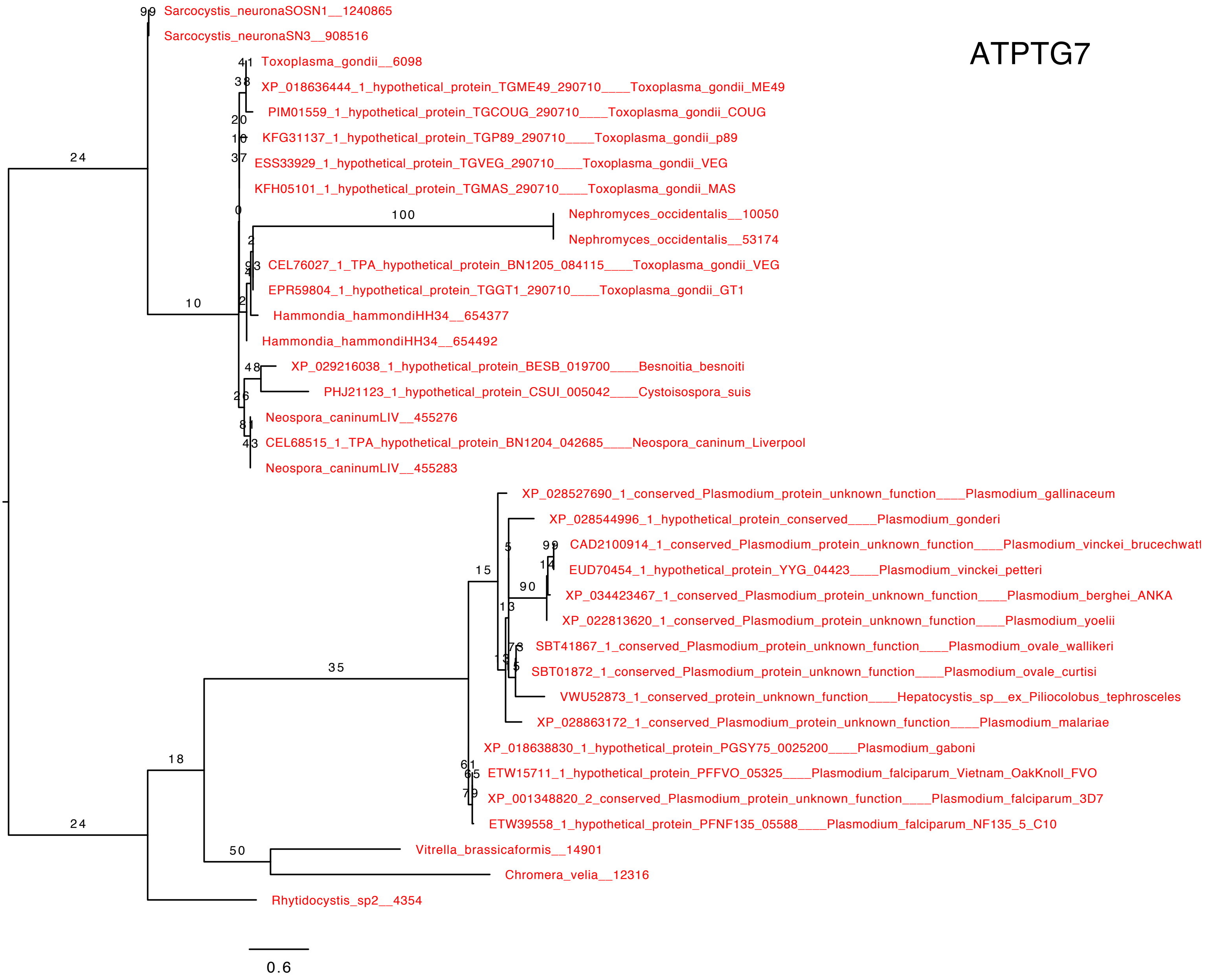
ATPTG7
Sarcocystis_neuronaSOSN1__1240865
Sarcocystis_neuronaSN3__908516
Toxoplasma_gondii__6098
XP_018636444_1_hypothetical_protein_TGME49_290710____Toxoplasma_gondii_ME49
PIM01559_1_hypothetical_protein_TGCOUG_290710____Toxoplasma_gondii_COUG
KFG31137_1_hypothetical_protein_TGP89_290710____Toxoplasma_gondii_p89
ESS33929_1_hypothetical_protein_TGVEG_290710____Toxoplasma_gondii_VEG
KFH05101_1_hypothetical_protein_TGMAS_290710____Toxoplasma_gondii_MAS
Nephromyces_occidentalis__10050
Nephromyces_occidentalis__53174
CEL76027_1_TPA_hypothetical_protein_BN1205_084115____Toxoplasma_gondii_VEG
EPR59804_1_hypothetical_protein_TGGT1_290710____Toxoplasma_gondii_GT1
Hammondia_hammondiHH34__654377
Hammondia_hammondiHH34__654492
XP_029216038_1_hypothetical_protein_BESB_019700____Besnoitia_besnoiti
PHJ21123_1_hypothetical_protein_CSUI_005042____Cystoisospora_suis
Neospora_caninumLIV__455276
CEL68515_1_TPA_hypothetical_protein_BN1204_042685____Neospora_caninum_Liverpool
Neospora_caninumLIV__455283
XP_028527690_1_conserved_Plasmodium_protein_unknown_function____Plasmodium_gallinaceum
XP_028544996_1_hypothetical_protein_conserved____Plasmodium_gonderi
CAD2100914_1_conserved_Plasmodium_protein_unknown_function____Plasmodium_vinckei_brucechwat
EUD70454_1_hypothetical_protein_YYG_04423____Plasmodium_vinckei_petteri
XP_034423467_1_conserved_Plasmodium_protein_unknown_function____Plasmodium_berghei_ANKA
XP_022813620_1_conserved_Plasmodium_protein_unknown_function____Plasmodium_yoelii
SBT41867_1_conserved_Plasmodium_protein_unknown_function____Plasmodium_ovale_wallikeri
SBT01872_1_conserved_Plasmodium_protein_unknown_function____Plasmodium_ovale_curtisi
VWU52873_1_conserved_protein_unknown_function____Hepatocystis_sp__ex_Piliocolobus_tephrosceles
XP_028863172_1_conserved_Plasmodium_protein_unknown_function____Plasmodium_malariae
XP_018638830_1_hypothetical_protein_PGSY75_0025200____Plasmodium_gaboni
ETW15711_1_hypothetical_protein_PFFVO_05325____Plasmodium_falciparum_Vietnam_OakKnoll_FVO
XP_001348820_2_conserved_Plasmodium_protein_unknown_function____Plasmodium_falciparum_3D7
ETW39558_1_hypothetical_protein_PFNF135_05588____Plasmodium_falciparum_NF135_5_C10
Vitrella_brassicaformis__14901
Chromera_velia__12316
Rhytidocystis_sp2__4354
0.6

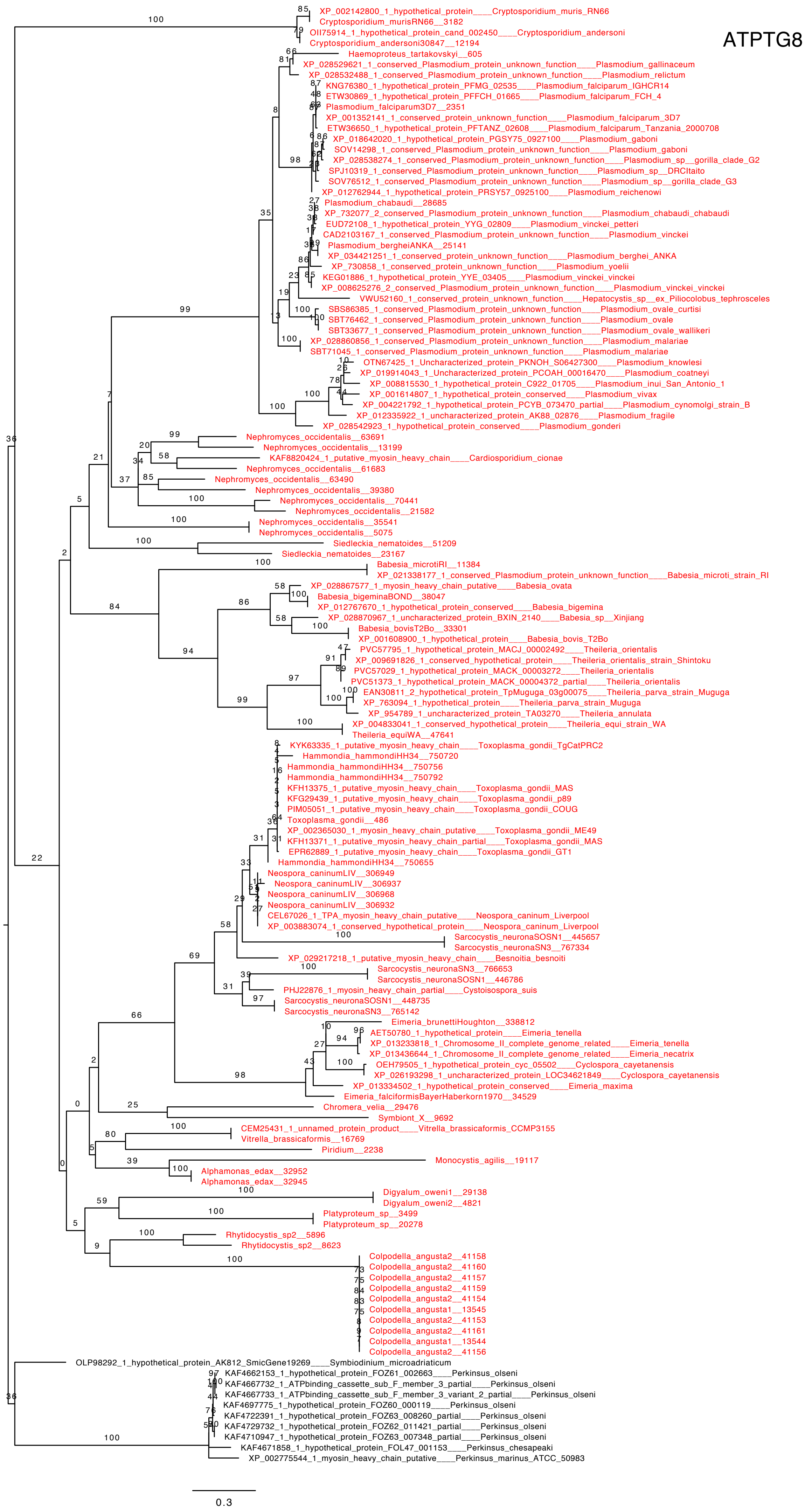
ATPTG8
XP_002142800_1_hypothetical_protein____Cryptosporidium_muris_RN66
Cryptosporidium_murisRN66__3182
OII75914_1_hypothetical_protein_cand_002450____Cryptosporidium_andersoni
Cryptosporidium_andersoni30847__12194
Haemoproteus_tartakovskyi__605
XP_028529621_1_conserved_Plasmodium_protein_unknown_function____Plasmodium_gallinaceum
XP_028532488_1_conserved_Plasmodium_protein_unknown_function____Plasmodium_relictum
KNG76380_1_hypothetical_protein_PFMG_02535____Plasmodium_falciparum_IGHCR14
ETW30869_1_hypothetical_protein_PFFCH_01665____Plasmodium_falciparum_FCH_4
Plasmodium_falciparum3D7__2351
XP_001352141_1_conserved_protein_unknown_function____Plasmodium_falciparum_3D7
ETW36650_1_hypothetical_protein_PFTANZ_02608____Plasmodium_falciparum_Tanzania_2000708
XP_018642020_1_hypothetical_protein_PGSY75_0927100____Plasmodium_gaboni
SOV14298_1_conserved_Plasmodium_protein_unknown_function____Plasmodium_gaboni
XP_028538274_1_conserved_Plasmodium_protein_unknown_function____Plasmodium_sp__gorilla_clade_G2
SPJ10319_1_conserved_Plasmodium_protein_unknown_function____Plasmodium_sp__DRCItaito
SOV76512_1_conserved_Plasmodium_protein_unknown_function____Plasmodium_sp__gorilla_clade_G3
XP_012762944_1_hypothetical_protein_PRSY57_0925100____Plasmodium_reichenowi
Plasmodium_chabaudi__28685
XP_732077_2_conserved_Plasmodium_protein_unknown_function____Plasmodium_chabaudi_chabaudi
EUD72108_1_hypothetical_protein_YYG_02809____Plasmodium_vinckei_petteri
CAD2103167_1_conserved_Plasmodium_protein_unknown_function____Plasmodium_vinckei
Plasmodium_bergheiANKA__25141
XP_034421251_1_conserved_protein_unknown_function____Plasmodium_berghei_ANKA
XP_730858_1_conserved_protein_unknown_function____Plasmodium_yoelii
KEG01886_1_hypothetical_protein_YYE_03405____Plasmodium_vinckei_vinckei
XP_008625276_2_conserved_Plasmodium_protein_unknown_function____Plasmodium_vinckei_vinckei
VWU52160_1_conserved_protein_unknown_function____Hepatocystis_sp__ex_Piliocolobus_tephrosceles
SBS86385_1_conserved_Plasmodium_protein_unknown_function____Plasmodium_ovale_curtisi
SBT76462_1_conserved_Plasmodium_protein_unknown_function____Plasmodium_ovale
SBT33677_1_conserved_Plasmodium_protein_unknown_function____Plasmodium_ovale_wallikeri
XP_028860856_1_conserved_Plasmodium_protein_unknown_function____Plasmodium_malariae
SBT71045_1_conserved_Plasmodium_protein_unknown_function____Plasmodium_malariae
OTN67425_1_Uncharacterized_protein_PKNOH_S06427300____Plasmodium_knowlesi
XP_019914043_1_Uncharacterized_protein_PCOAH_00016470____Plasmodium_coatneyi
XP_008815530_1_hypothetical_protein_C922_01705____Plasmodium_inui_San_Antonio_1
XP_001614807_1_hypothetical_protein____Plasmodium_vivax
XP_004221792_1_hypothetical_protein_PCYB_073470_partial____Plasmodium_cynomolgi_strain_B
XP_012335922_1_uncharacterized_protein_AK88_02876____Plasmodium_fragile
XP_028542923_1_hypothetical_protein_conserved____Plasmodium_gonderi
Nephromyces_occidentalis__63691
Nephromyces_occidentalis__13199
KAF8820424_1_putative_myosin_heavy_chain____Cardiosporidium_cionae
Nephromyces_occidentalis__61683
Nephromyces_occidentalis__63490
Nephromyces_occidentalis__39380
Nephromyces_occidentalis__70441
Nephromyces_occidentalis__21582
Nephromyces_occidentalis__35541
Nephromyces_occidentalis__5075
Siedleckia_nematoides__51209
Siedleckia_nematoides__23167
Babesia_microtiRI__11384
XP_021338177_1_conserved_Plasmodium_protein_unknown_function____Babesia_microti_strain_RI
XP_028867577_1_myosin_heavy_chain_putative____Babesia_ovata
Babesia_bigeminaBOND__38047
XP_012767670_1_hypothetical_protein_conserved____Babesia_bigemina
XP_028870967_1_uncharacterized_protein_BXIN_2140____Babesia_sp__Xinjiang
Babesia_bovisT2Bo__33301
XP_001608900_1_hypothetical_protein____Babesia_bovis_T2Bo
PVC57795_1_hypothetical_protein_MACJ_00002492____Theileria_orientalis
XP_009691826_1_conserved_hypothetical_protein____Theileria_orientalis_strain_Shintoku
PVC57029_1_hypothetical_protein_MACK_00003272____Theileria_orientalis
PVC51373_1_hypothetical_protein_MACK_00004372_partial____Theileria_orientalis
EAN30811_2_hypothetical_protein_TpMuguga_03g00075____Theileria_parva_strain_Muguga
XP_763094_1_hypothetical_protein____Theileria_parva_strain_Muguga
XP_954789_1_uncharacterized_protein_TA03270____Theileria_annulata
XP_004833041_1_conserved_hypothetical_protein____Theileria_equi_strain_WA
Theileria_equiWA__47641
KYK63335_1_putative_myosin_heavy_chain____Toxoplasma_gondii_TgCatPRC2
Hammondia_hammondiHH34__750720
Hammondia_hammondiHH34__750756
Hammondia_hammondiHH34__750792
KFH13375_1_putative_myosin_heavy_chain____Toxoplasma_gondii_MAS
KFG29439_1_putative_myosin_heavy_chain____Toxoplasma_gondii_p89
PIM05051_1_putative_myosin_heavy_chain____Toxoplasma_gondii_COUG
Toxoplasma_gondii__486
XP_002365030_1_myosin_heavy_chain_putative____Toxoplasma_gondii_ME49
KFH13371_1_putative_myosin_heavy_chain_partial____Toxoplasma_gondii_MAS
EPR62889_1_putative_myosin_heavy_chain____Toxoplasma_gondii_GT1
Hammondia_hammondiHH34__750655
Neospora_caninumLIV__306949
Neospora_caninumLIV__306937
Neospora_caninumLIV__306968
Neospora_caninumLIV__306932
CEL67026_1_TPA_myosin_heavy_chain_putative____Neospora_caninum_Liverpool
XP_003883074_1_conserved_hypothetical_protein____Neospora_caninum_Liverpool
Sarcocystis_neuronaSOSN1__445657
Sarcocystis_neuronaSN3__767334
XP_029217218_1_putative_myosin_heavy_chain____Besnoitia_besnoiti
Sarcocystis_neuronaSN3__766653
Sarcocystis_neuronaSOSN1__446786
PHJ22876_1_myosin_heavy_chain_partial____Cystoisospora_suis
Sarcocystis_neuronaSOSN1__448735
Sarcocystis_neuronaSN3__765142
Eimeria_brunettiHoughton__338812
AET50780_1_hypothetical_protein____Eimeria_tenella
XP_013233818_1_Chromosome_II_complete_genome_related____Eimeria_tenella
XP_013436644_1_Chromosome_II_complete_genome_related____Eimeria_necatrix
OEH79505_1_hypothetical_protein_cyc_05502____Cyclospora_cayetanensis
XP_026193298_1_uncharacterized_protein_LOC34621849____Cyclospora_cayetanensis
XP_013334502_1_hypothetical_protein_conserved____Eimeria_maxima
Eimeria_falciformisBayerHaberkorn1970__34529
Chromera_velia__29476
Symbiont_X__9692
CEM25431_1_unnamed_protein_product____Vitrella_brassicaformis_CCMP3155
Vitrella_brassicaformis__16769
Piridium__2238
Monocystis_agilis__19117
Alphamonas_edax__32952
Alphamonas_edax__32945
Digyalum_oweni1__29138
Digyalum_oweni2__4821
Platyproteum_sp__3499
Platyproteum_sp__20278
Rhytidocystis_sp2__5896
Rhytidocystis_sp2__8623
Colpodella_angusta2__41158
Colpodella_angusta2__41160
Colpodella_angusta2__41157
Colpodella_angusta2__41159
Colpodella_angusta2__41154
Colpodella_angusta1__13545
Colpodella_angusta2__41153
Colpodella_angusta2__41161
Colpodella_angusta1__13544
Colpodella_angusta2__41156
OLP98292_1_hypothetical_protein_AK812_SmicGene19269____Symbiodinium_microadriaticum
KAF4662153_1_hypothetical_protein_FOZ61_002663____Perkinsus_olseni
KAF4667732_1_ATPbinding_cassette_sub_F_member_3_partial____Perkinsus_olseni
KAF4667733_1_ATPbinding_cassette_sub_F_member_3_variant_2_partial____Perkinsus_olseni
KAF4697775_1_hypothetical_protein_FOZ60_000119____Perkinsus_olseni
KAF4722391_1_hypothetical_protein_FOZ63_008260_partial____Perkinsus_olseni
KAF4729732_1_hypothetical_protein_FOZ62_011421_partial____Perkinsus_olseni
KAF4710947_1_hypothetical_protein_FOZ63_007348_partial____Perkinsus_olseni
KAF4671858_1_hypothetical_protein_FOL47_001153____Perkinsus_chesapeaki
XP_002775544_1_myosin_heavy_chain_putative____Perkinsus_marinus_ATCC_50983
0.3

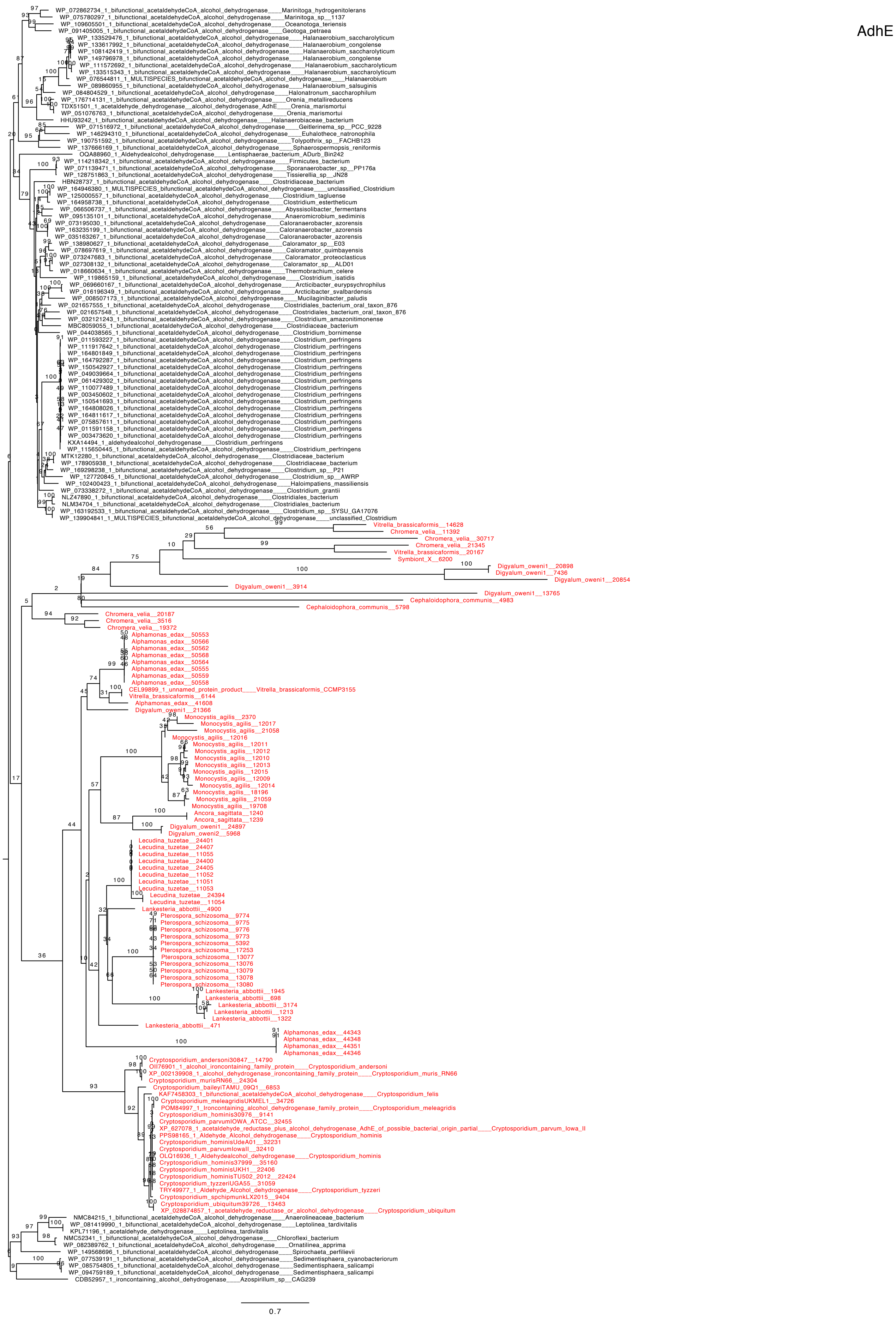

AdhE
WP_072862734_1_bifunctional_acetaldehydeCoA_alcohol_dehydrogenase____Marinitoga_hydrogenitolerans
WP_075780297_1_bifunctional_acetaldehydeCoA_alcohol_dehydrogenase____Marinitoga_sp__1137
WP_109605501_1_bifunctional_acetaldehydeCoA_alcohol_dehydrogenase____Oceanotoga_teriensis
WP_091405005_1_bifunctional_acetaldehydeCoA_alcohol_dehydrogenase____Geotoga_petraea
WP_133529476_1_bifunctional_acetaldehydeCoA_alcohol_dehydrogenase____Halanaerobium_saccharolyticum
WP_133617992_1_bifunctional_acetaldehydeCoA_alcohol_dehydrogenase____Halanaerobium_congolense
WP_108142419_1_bifunctional_acetaldehydeCoA_alcohol_dehydrogenase____Halanaerobium_saccharolyticum
WP_149796978_1_bifunctional_acetaldehydeCoA_alcohol_dehydrogenase____Halanaerobium_congolense
WP_111572692_1_bifunctional_acetaldehydeCoA_alcohol_dehydrogenase____Halanaerobium_saccharolyticum
WP_133515343_1_bifunctional_acetaldehydeCoA_alcohol_dehydrogenase____Halanaerobium_saccharolyticum
WP_076544811_1_MULTISPECIES_bifunctional_acetaldehydeCoA_alcohol_dehydrogenase____Halanaerobium
WP_089860955_1_bifunctional_acetaldehydeCoA_alcohol_dehydrogenase____Halanaerobium_salsuginis
WP_084804529_1_bifunctional_acetaldehydeCoA_alcohol_dehydrogenase____Halonatronum_saccharophilum
WP_176714131_1_bifunctional_acetaldehydeCoA_alcohol_dehydrogenase____Orenia_metallireducens
TDX51501_1_acetaldehyde_dehydrogenase__alcohol_dehydrogenase_AdhE____Orenia_marismortui
WP_051076763_1_bifunctional_acetaldehydeCoA_alcohol_dehydrogenase____Orenia_marismortui
HHU93242_1_bifunctional_acetaldehydeCoA_alcohol_dehydrogenase____Halanaerobiaceae_bacterium
WP_071516972_1_bifunctional_acetaldehydeCoA_alcohol_dehydrogenase____Geitlerinema_sp__PCC_9228
WP_146294310_1_bifunctional_acetaldehydeCoA_alcohol_dehydrogenase____Euhalothece_natronophila
WP_190751592_1_bifunctional_acetaldehydeCoA_alcohol_dehydrogenase____Tolypothrix_sp__FACHB123
WP_137666169_1_bifunctional_acetaldehydeCoA_alcohol_dehydrogenase____Sphaerospermopsis_reniformis
OQA88960_1_Aldehydealcohol_dehydrogenase____Lentisphaerae_bacterium_ADurb_Bin242
WP_114218342_1_bifunctional_acetaldehydeCoA_alcohol_dehydrogenase____Firmicutes_bacterium
WP_071139471_1_bifunctional_acetaldehydeCoA_alcohol_dehydrogenase____Sporanaerobacter_sp__PP176a
WP_128751863_1_bifunctional_acetaldehydeCoA_alcohol_dehydrogenase____Tissierellia_sp__JN28
HBN28737_1_bifunctional_acetaldehydeCoA_alcohol_dehydrogenase____Clostridiaceae_bacterium
WP_164946380_1_MULTISPECIES_bifunctional_acetaldehydeCoA_alcohol_dehydrogenase____unclassified_Clostridium
WP_125000557_1_bifunctional_acetaldehydeCoA_alcohol_dehydrogenase____Clostridium_tagluense
WP_164958738_1_bifunctional_acetaldehydeCoA_alcohol_dehydrogenase____Clostridium_estertheticum
WP_066506737_1_bifunctional_acetaldehydeCoA_alcohol_dehydrogenase____Abyssisolibacter_fermentans
WP_095135101_1_bifunctional_acetaldehydeCoA_alcohol_dehydrogenase____Anaeromicrobium_sediminis
WP_073195030_1_bifunctional_acetaldehydeCoA_alcohol_dehydrogenase____Caloranaerobacter_azorensis
WP_163235199_1_bifunctional_acetaldehydeCoA_alcohol_dehydrogenase____Caloranaerobacter_azorensis
WP_035163267_1_bifunctional_acetaldehydeCoA_alcohol_dehydrogenase____Caloranaerobacter_azorensis
WP_138980627_1_bifunctional_acetaldehydeCoA_alcohol_dehydrogenase____Caloramator_sp__E03
WP_078697619_1_bifunctional_acetaldehydeCoA_alcohol_dehydrogenase____Caloramator_quimbayensis
WP_073247683_1_bifunctional_acetaldehydeCoA_alcohol_dehydrogenase____Caloramator_proteoclasticus
WP_027308132_1_bifunctional_acetaldehydeCoA_alcohol_dehydrogenase____Caloramator_sp__ALD01
WP_018660634_1_bifunctional_acetaldehydeCoA_alcohol_dehydrogenase____Thermobrachium_celere
WP_119865159_1_bifunctional_acetaldehydeCoA_alcohol_dehydrogenase____Clostridium_isatidis
WP_069660167_1_bifunctional_acetaldehydeCoA_alcohol_dehydrogenase____Arcticibacter_eurypsychrophilus
WP_016196349_1_bifunctional_acetaldehydeCoA_alcohol_dehydrogenase____Arcticibacter_svalbardensis
WP_008507173_1_bifunctional_acetaldehydeCoA_alcohol_dehydrogenase____Mucilaginibacter_paludis
WP_021657555_1_bifunctional_acetaldehydeCoA_alcohol_dehydrogenase____Clostridiales_bacterium_oral_taxon_876
WP_021657548_1_bifunctional_acetaldehydeCoA_alcohol_dehydrogenase____Clostridiales_bacterium_oral_taxon_876
WP_032121243_1_bifunctional_acetaldehydeCoA_alcohol_dehydrogenase____Clostridium_amazonitimonense
MBC8059055_1_bifunctional_acetaldehydeCoA_alcohol_dehydrogenase____Clostridiaceae_bacterium
WP_044038565_1_bifunctional_acetaldehydeCoA_alcohol_dehydrogenase____Clostridium_bornimense
WP_011593227_1_bifunctional_acetaldehydeCoA_alcohol_dehydrogenase____Clostridium_perfringens
WP_111917642_1_bifunctional_acetaldehydeCoA_alcohol_dehydrogenase____Clostridium_perfringens
WP_164801849_1_bifunctional_acetaldehydeCoA_alcohol_dehydrogenase____Clostridium_perfringens
WP_164792287_1_bifunctional_acetaldehydeCoA_alcohol_dehydrogenase____Clostridium_perfringens
WP_150542927_1_bifunctional_acetaldehydeCoA_alcohol_dehydrogenase____Clostridium_perfringens
WP_049039664_1_bifunctional_acetaldehydeCoA_alcohol_dehydrogenase____Clostridium_perfringens
WP_061429302_1_bifunctional_acetaldehydeCoA_alcohol_dehydrogenase____Clostridium_perfringens
WP_110077489_1_bifunctional_acetaldehydeCoA_alcohol_dehydrogenase____Clostridium_perfringens
WP_003450602_1_bifunctional_acetaldehydeCoA_alcohol_dehydrogenase____Clostridium_perfringens
WP_150541693_1_bifunctional_acetaldehydeCoA_alcohol_dehydrogenase____Clostridium_perfringens
WP_164808026_1_bifunctional_acetaldehydeCoA_alcohol_dehydrogenase____Clostridium_perfringens
WP_164811617_1_bifunctional_acetaldehydeCoA_alcohol_dehydrogenase____Clostridium_perfringens
WP_075857611_1_bifunctional_acetaldehydeCoA_alcohol_dehydrogenase____Clostridium_perfringens
WP_011591158_1_bifunctional_acetaldehydeCoA_alcohol_dehydrogenase____Clostridium_perfringens
WP_003473620_1_bifunctional_acetaldehydeCoA_alcohol_dehydrogenase____Clostridium_perfringens
KXA14494_1_aldehydealcohol_dehydrogenase____Clostridium_perfringens
WP_115650445_1_bifunctional_acetaldehydeCoA_alcohol_dehydrogenase____Clostridium_perfringens
MTK12280_1_bifunctional_acetaldehydeCoA_alcohol_dehydrogenase____Clostridiaceae_bacterium
WP_178905938_1_bifunctional_acetaldehydeCoA_alcohol_dehydrogenase____Clostridiaceae_bacterium
WP_169298238_1_bifunctional_acetaldehydeCoA_alcohol_dehydrogenase____Clostridium_sp__P21
WP_127720845_1_bifunctional_acetaldehydeCoA_alcohol_dehydrogenase____Clostridium_sp__AWRP
WP_102400423_1_bifunctional_acetaldehydeCoA_alcohol_dehydrogenase____Haloimpatiens_massiliensis
WP_073338272_1_bifunctional_acetaldehydeCoA_alcohol_dehydrogenase____Clostridium_grantii
NLZ47890_1_bifunctional_acetaldehydeCoA_alcohol_dehydrogenase____Clostridiales_bacterium
NLM34704_1_bifunctional_acetaldehydeCoA_alcohol_dehydrogenase____Clostridiales_bacterium
WP_163192533_1_bifunctional_acetaldehydeCoA_alcohol_dehydrogenase____Clostridium_sp__SYSU_GA17076
WP_139904841_1_MULTISPECIES_bifunctional_acetaldehydeCoA_alcohol_dehydrogenase____unclassified_Clostridium
Vitrella_brassicaformis__14628
Chromera_velia__11392
Chromera_velia__30717
Chromera_velia__21345
Vitrella_brassicaformis__20167
Symbiont_X__6200
Digyalum_oweni1__20898
Digyalum_oweni1__7436
Digyalum_oweni1__20854
Digyalum_oweni1__3914
Digyalum_oweni1__13765
Cephaloidophora_communis__4983
Cephaloidophora_communis__5798
Chromera_velia__20187
Chromera_velia__3516
Chromera_velia__19372
Alphamonas_edax__50553
Alphamonas_edax__50566
Alphamonas_edax__50562
Alphamonas_edax__50568
Alphamonas_edax__50564
Alphamonas_edax__50555
Alphamonas_edax__50559
Alphamonas_edax__50558
CEL99899_1_unnamed_protein_product____Vitrella_brassicaformis_CCMP3155
Vitrella_brassicaformis__6144
Alphamonas_edax__41608
Digyalum_oweni1__21366
Monocystis_agilis__2370
Monocystis_agilis__12017
Monocystis_agilis__21058
Monocystis_agilis__12016
Monocystis_agilis__12011
Monocystis_agilis__12012
Monocystis_agilis__12010
Monocystis_agilis__12013
Monocystis_agilis__12015
Monocystis_agilis__12009
Monocystis_agilis__12014
Monocystis_agilis__18196
Monocystis_agilis__21059
Monocystis_agilis__19708
Ancora_sagittata__1240
Ancora_sagittata__1239
Digyalum_oweni1__24897
Digyalum_oweni2__5968
Lecudina_tuzetae__24401
Lecudina_tuzetae__24407
Lecudina_tuzetae__11055
Lecudina_tuzetae__24400
Lecudina_tuzetae__24405
Lecudina_tuzetae__11052
Lecudina_tuzetae__11051
Lecudina_tuzetae__11053
Lecudina_tuzetae__24394
Lecudina_tuzetae__11054
Lankesteria_abbottii__4900
Pterospora_schizosoma__9774
Pterospora_schizosoma__9775
Pterospora_schizosoma__9776
Pterospora_schizosoma__9773
Pterospora_schizosoma__5392
Pterospora_schizosoma__17253
Pterospora_schizosoma__13077
Pterospora_schizosoma__13076
Pterospora_schizosoma__13079
Pterospora_schizosoma__13078
Pterospora_schizosoma__13080
Lankesteria_abbottii__1945
Lankesteria_abbottii__698
Lankesteria_abbottii__3174
Lankesteria_abbottii__1213
Lankesteria_abbottii__1322
Lankesteria_abbottii__471
Alphamonas_edax__44343
Alphamonas_edax__44348
Alphamonas_edax__44351
Alphamonas_edax__44346
Cryptosporidium_andersoni30847__14790
OII76901_1_alcohol_ironcontaining_family_protein____Cryptosporidium_andersoni
XP_002139908_1_alcohol_dehydrogenase_ironcontaining_family_protein____Cryptosporidium_muris_RN66
Cryptosporidium_murisRN66__24304
Cryptosporidium_baileyiTAMU_09Q1__6853
KAF7458303_1_bifunctional_acetaldehydeCoA_alcohol_dehydrogenase____Cryptosporidium_felis
Cryptosporidium_meleagridisUKMEL1__34726
POM84997_1_Ironcontaining_alcohol_dehydrogenase_family_protein____Cryptosporidium_meleagridis
Cryptosporidium_hominis30976__9141
Cryptosporidium_parvumIOWA_ATCC__32455
XP_627078_1_acetaldehyde_reductase_plus_alcohol_dehydrogenase_AdhE_of_possible_bacterial_origin_partial____Cryptosporidium_parvum_Iowa_II
PPS98165_1_Aldehyde_Alcohol_dehydrogenase____Cryptosporidium_hominis
Cryptosporidium_hominisUdeA01__32231
Cryptosporidium_parvumIowaII__32410
OLQ16936_1_Aldehydealcohol_dehydrogenase____Cryptosporidium_hominis
Cryptosporidium_hominis37999__35160
Cryptosporidium_hominisUKH1__22406
Cryptosporidium_hominisTU502_2012__22424
Cryptosporidium_tyzzeriUGA55__31059
TRY49977_1_Aldehyde_Alcohol_dehydrogenase____Cryptosporidium_tyzzeri
Cryptosporidium_spchipmunkLX2015__9404
Cryptosporidium_ubiquitum39726__13463
XP_028874857_1_acetaldehyde_reductase_or_alcohol_dehydrogenase____Cryptosporidium_ubiquitum
NMC84215_1_bifunctional_acetaldehydeCoA_alcohol_dehydrogenase____Anaerolineaceae_bacterium
WP_081419990_1_bifunctional_acetaldehydeCoA_alcohol_dehydrogenase____Leptolinea_tardivitalis
KPL71196_1_acetaldehyde_dehydrogenase____Leptolinea_tardivitalis
NMC52341_1_bifunctional_acetaldehydeCoA_alcohol_dehydrogenase____Chloroflexi_bacterium
WP_082389762_1_bifunctional_acetaldehydeCoA_alcohol_dehydrogenase____Ornatilinea_apprima
WP_149568696_1_bifunctional_acetaldehydeCoA_alcohol_dehydrogenase____Spirochaeta_perfilievii
WP_077539191_1_bifunctional_acetaldehydeCoA_alcohol_dehydrogenase____Sedimentisphaera_cyanobacteriorum
WP_085754805_1_bifunctional_acetaldehydeCoA_alcohol_dehydrogenase____Sedimentisphaera_salicampi
WP_094759189_1_bifunctional_acetaldehydeCoA_alcohol_dehydrogenase____Sedimentisphaera_salicampi
CDB52957_1_ironcontaining_alcohol_dehydrogenase____Azospirillum_sp__CAG239
0.7

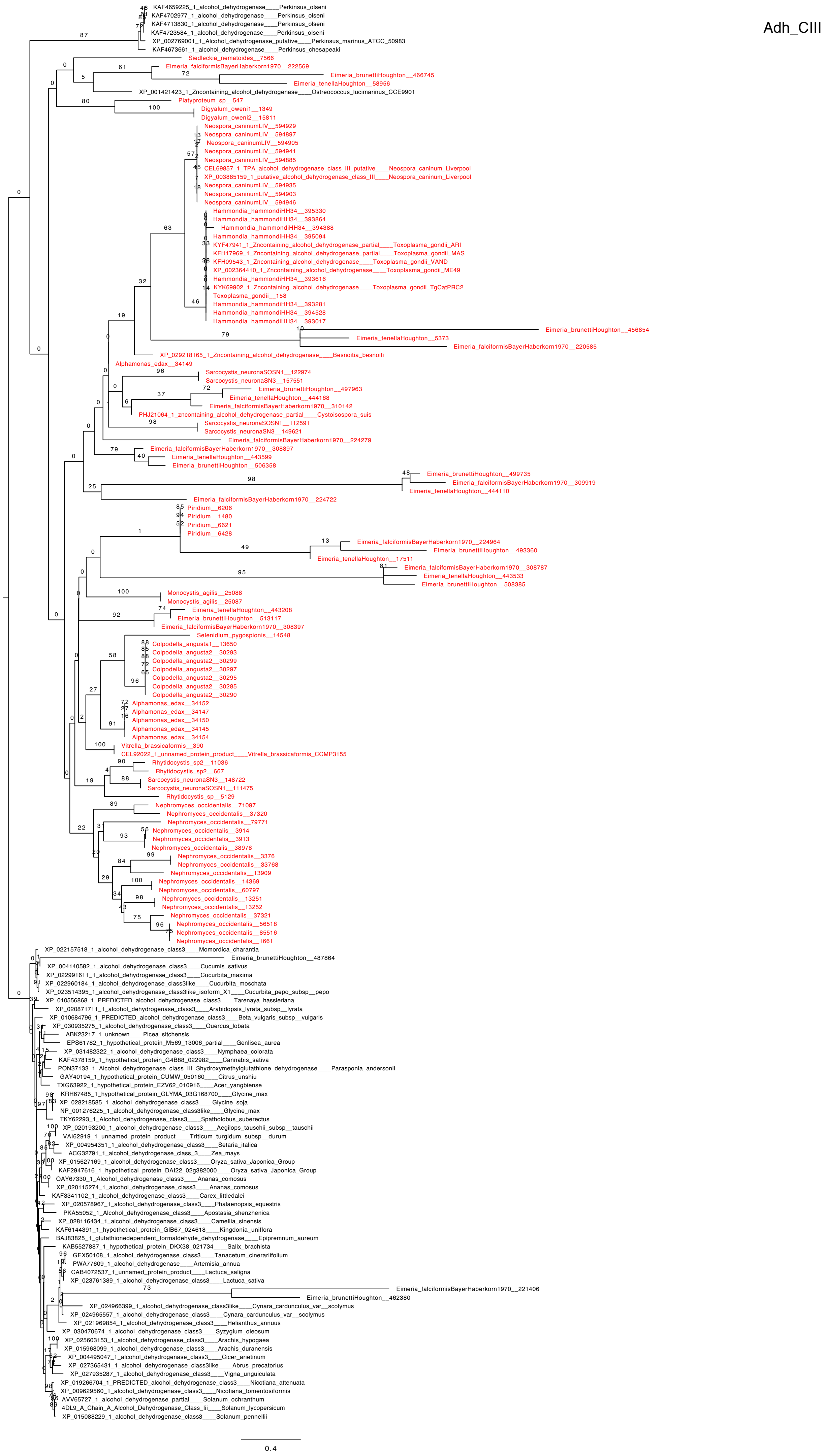
Adh_CIII
KAF4659225_1_alcohol_dehydrogenase____Perkinsus_olseni
KAF4702977_1_alcohol_dehydrogenase____Perkinsus_olseni
KAF4713830_1_alcohol_dehydrogenase____Perkinsus_olseni
KAF4723584_1_alcohol_dehydrogenase____Perkinsus_olseni
XP_002769001_1_Alcohol_dehydrogenase_putative____Perkinsus_marinus_ATCC_50983
KAF4673661_1_alcohol_dehydrogenase____Perkinsus_chesapeaki
Siedleckia_nematoides__7566
Eimeria_falciformisBayerHaberkorn1970__222569
Eimeria_brunettiHoughton__466745
Eimeria_tenellaHoughton__58956
XP_001421423_1_Zncontaining_alcohol_dehydrogenase____Ostreococcus_lucimarinus_CCE9901
Platyproteum_sp__547
Digyalum_oweni1__1349
Digyalum_oweni2__15811
Neospora_caninumLIV__594929
Neospora_caninumLIV__594897
Neospora_caninumLIV__594905
Neospora_caninumLIV__594941
Neospora_caninumLIV__594885
CEL69857_1_TPA_alcohol_dehydrogenase_class_III_putative____Neospora_caninum_Liverpool
XP_003885159_1_putative_alcohol_dehydrogenase_class_III____Neospora_caninum_Liverpool
Neospora_caninumLIV__594935
Neospora_caninumLIV__594903
Neospora_caninumLIV__594946
Hammondia_hammondiHH34__395330
Hammondia_hammondiHH34__393864
Hammondia_hammondiHH34__394388
Hammondia_hammondiHH34__395094
KYF47941_1_Zncontaining_alcohol_dehydrogenase_partial____Toxoplasma_gondii_ARI
KFH17969_1_Zncontaining_alcohol_dehydrogenase_partial____Toxoplasma_gondii_MAS
KFH09543_1_Zncontaining_alcohol_dehydrogenase____Toxoplasma_gondii_VAND
XP_002364410_1_Zncontaining_alcohol_dehydrogenase____Toxoplasma_gondii_ME49
Hammondia_hammondiHH34__393616
KYK69902_1_Zncontaining_alcohol_dehydrogenase____Toxoplasma_gondii_TgCatPRC2
Toxoplasma_gondii__158
Hammondia_hammondiHH34__393281
Hammondia_hammondiHH34__394528
Hammondia_hammondiHH34__393017
Eimeria_brunettiHoughton__456854
Eimeria_tenellaHoughton__5373
Eimeria_falciformisBayerHaberkorn1970__220585
XP_029218165_1_Zncontaining_alcohol_dehydrogenase____Besnoitia_besnoiti
Alphamonas_edax__34149
Sarcocystis_neuronaSOSN1__122974
Sarcocystis_neuronaSN3__157551
Eimeria_brunettiHoughton__497963
Eimeria_tenellaHoughton__444168
Eimeria_falciformisBayerHaberkorn1970__310142
PHJ21064_1_zncontaining_alcohol_dehydrogenase_partial____Cystoisospora_suis
Sarcocystis_neuronaSOSN1__112591
Sarcocystis_neuronaSN3__149621
Eimeria_falciformisBayerHaberkorn1970__224279
Eimeria_falciformisBayerHaberkorn1970__308897
Eimeria_tenellaHoughton__443599
Eimeria_brunettiHoughton__506358
Eimeria_brunettiHoughton__499735
Eimeria_falciformisBayerHaberkorn1970__309919
Eimeria_tenellaHoughton__444110
Eimeria_falciformisBayerHaberkorn1970__224722
Piridium__6206
Piridium__1480
Piridium__6621
Piridium__6428
Eimeria_falciformisBayerHaberkorn1970__224964
Eimeria_brunettiHoughton__493360
Eimeria_tenellaHoughton__17511
Eimeria_falciformisBayerHaberkorn1970__308787
Eimeria_tenellaHoughton__443533
Eimeria_brunettiHoughton__508385
Monocystis_agilis__25088
Monocystis_agilis__25087
Eimeria_tenellaHoughton__443208
Eimeria_brunettiHoughton__513117
Eimeria_falciformisBayerHaberkorn1970__308397
Selenidium_pygospionis__14548
Colpodella_angusta1__13650
Colpodella_angusta2__30293
Colpodella_angusta2__30299
Colpodella_angusta2__30297
Colpodella_angusta2__30295
Colpodella_angusta2__30285
Colpodella_angusta2__30290
Alphamonas_edax__34152
Alphamonas_edax__34147
Alphamonas_edax__34150
Alphamonas_edax__34145
Alphamonas_edax__34154
Vitrella_brassicaformis__390
CEL92022_1_unnamed_protein_product____Vitrella_brassicaformis_CCMP3155
Rhytidocystis_sp2__11036
Rhytidocystis_sp2__667
Sarcocystis_neuronaSN3__148722
Sarcocystis_neuronaSOSN1__111475
Rhytidocystis_sp__5129
Nephromyces_occidentalis__71097
Nephromyces_occidentalis__37320
Nephromyces_occidentalis__79771
Nephromyces_occidentalis__3914
Nephromyces_occidentalis__3913
Nephromyces_occidentalis__38978
Nephromyces_occidentalis__3376
Nephromyces_occidentalis__33768
Nephromyces_occidentalis__13909
Nephromyces_occidentalis__14369
Nephromyces_occidentalis__60797
Nephromyces_occidentalis__13251
Nephromyces_occidentalis__13252
Nephromyces_occidentalis__37321
Nephromyces_occidentalis__56518
Nephromyces_occidentalis__85516
Nephromyces_occidentalis__1661
XP_022157518_1_alcohol_dehydrogenase_class3____Momordica_charantia
Eimeria_brunettiHoughton__487864
XP_004140582_1_alcohol_dehydrogenase_class3____Cucumis_sativus
XP_022991611_1_alcohol_dehydrogenase_class3____Cucurbita_maxima
XP_022960184_1_alcohol_dehydrogenase_class3like____Cucurbita_moschata
XP_023514395_1_alcohol_dehydrogenase_class3like_isoform_X1____Cucurbita_pepo_subsp__pepo
XP_010556868_1_PREDICTED_alcohol_dehydrogenase_class3____Tarenaya_hassleriana
XP_020871711_1_alcohol_dehydrogenase_class3____Arabidopsis_lyrata_subsp__lyrata
XP_010684796_1_PREDICTED_alcohol_dehydrogenase_class3____Beta_vulgaris_subsp__vulgaris
XP_030935275_1_alcohol_dehydrogenase_class3____Quercus_lobata
ABK23217_1_unknown____Picea_sitchensis
EPS61782_1_hypothetical_protein_M569_13006_partial____Genlisea_aurea
XP_031482322_1_alcohol_dehydrogenase_class3____Nymphaea_colorata
KAF4378159_1_hypothetical_protein_G4B88_022982____Cannabis_sativa
PON37133_1_Alcohol_dehydrogenase_class_III_Shydroxymethylglutathione_dehydrogenase____Parasponia_andersonii
GAY40194_1_hypothetical_protein_CUMW_050160____Citrus_unshiu
TXG63922_1_hypothetical_protein_EZV62_010916____Acer_yangbiense
KRH67485_1_hypothetical_protein_GLYMA_03G168700____Glycine_max
XP_028218585_1_alcohol_dehydrogenase_class3____Glycine_soja
NP_001276225_1_alcohol_dehydrogenase_class3like____Glycine_max
TKY62293_1_Alcohol_dehydrogenase_class3____Spatholobus_suberectus
XP_020193200_1_alcohol_dehydrogenase_class3____Aegilops_tauschii_subsp__tauschii
VAI62919_1_unnamed_protein_product____Triticum_turgidum_subsp__durum
XP_004954351_1_alcohol_dehydrogenase_class3____Setaria_italica
ACG32791_1_alcohol_dehydrogenase_class_3____Zea_mays
XP_015627169_1_alcohol_dehydrogenase_class3____Oryza_sativa_Japonica_Group
KAF2947616_1_hypothetical_protein_DAI22_02g382000____Oryza_sativa_Japonica_Group
OAY67330_1_Alcohol_dehydrogenase_class3____Ananas_comosus
XP_020115274_1_alcohol_dehydrogenase_class3____Ananas_comosus
KAF3341102_1_alcohol_dehydrogenase_class3____Carex_littledalei
XP_020578967_1_alcohol_dehydrogenase_class3____Phalaenopsis_equestris
PKA55052_1_Alcohol_dehydrogenase_class3____Apostasia_shenzhenica
XP_028116434_1_alcohol_dehydrogenase_class3____Camellia_sinensis
KAF6144391_1_hypothetical_protein_GIB67_024618____Kingdonia_uniflora
BAJ83825_1_glutathionedependent_formaldehyde_dehydrogenase____Epipremnum_aureum
KAB5527887_1_hypothetical_protein_DKX38_021734____Salix_brachista
GEX50108_1_alcohol_dehydrogenase_class3____Tanacetum_cinerariifolium
PWA77609_1_alcohol_dehydrogenase____Artemisia_annua
CAB4072537_1_unnamed_protein_product____Lactuca_saligna
XP_023761389_1_alcohol_dehydrogenase_class3____Lactuca_sativa
Eimeria_falciformisBayerHaberkorn1970__221406
Eimeria_brunettiHoughton__462380
XP_024966399_1_alcohol_dehydrogenase_class3like____Cynara_cardunculus_var__scolymus
XP_024965557_1_alcohol_dehydrogenase_class3____Cynara_cardunculus_var__scolymus
XP_021969854_1_alcohol_dehydrogenase_class3____Helianthus_annuus
XP_030470674_1_alcohol_dehydrogenase_class3____Syzygium_oleosum
XP_025603153_1_alcohol_dehydrogenase_class3____Arachis_hypogaea
XP_015968099_1_alcohol_dehydrogenase_class3____Arachis_duranensis
XP_004495047_1_alcohol_dehydrogenase_class3____Cicer_arietinum
XP_027365431_1_alcohol_dehydrogenase_class3like____Abrus_precatorius
XP_027935287_1_alcohol_dehydrogenase_class3____Vigna_unguiculata
XP_019266704_1_PREDICTED_alcohol_dehydrogenase_class3____Nicotiana_attenuata
XP_009629560_1_alcohol_dehydrogenase_class3____Nicotiana_tomentosiformis
AVV65727_1_alcohol_dehydrogenase_partial____Solanum_ochranthum
4DL9_A_Chain_A_Alcohol_Dehydrogenase_Class_Iii____Solanum_lycopersicum
XP_015088229_1_alcohol_dehydrogenase_class3____Solanum_pennellii
0.4

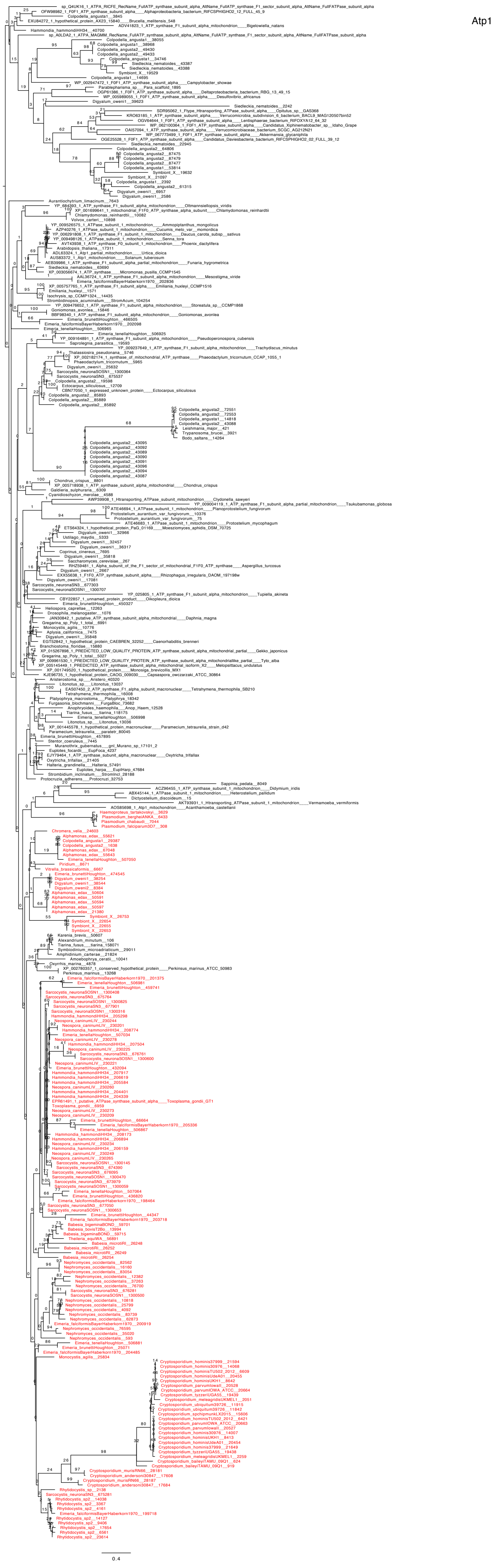
Atp1
0.4

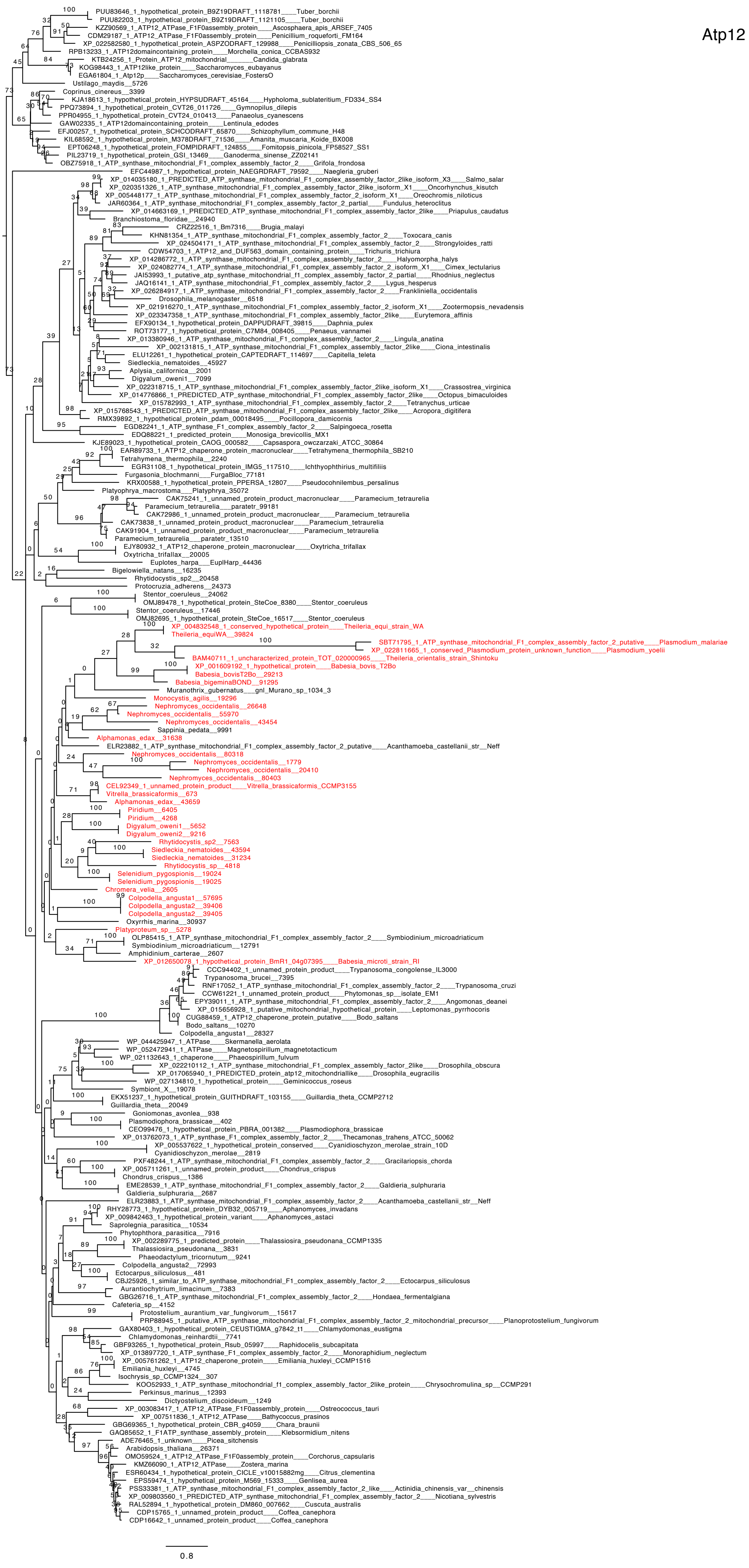
Atp12
0.8

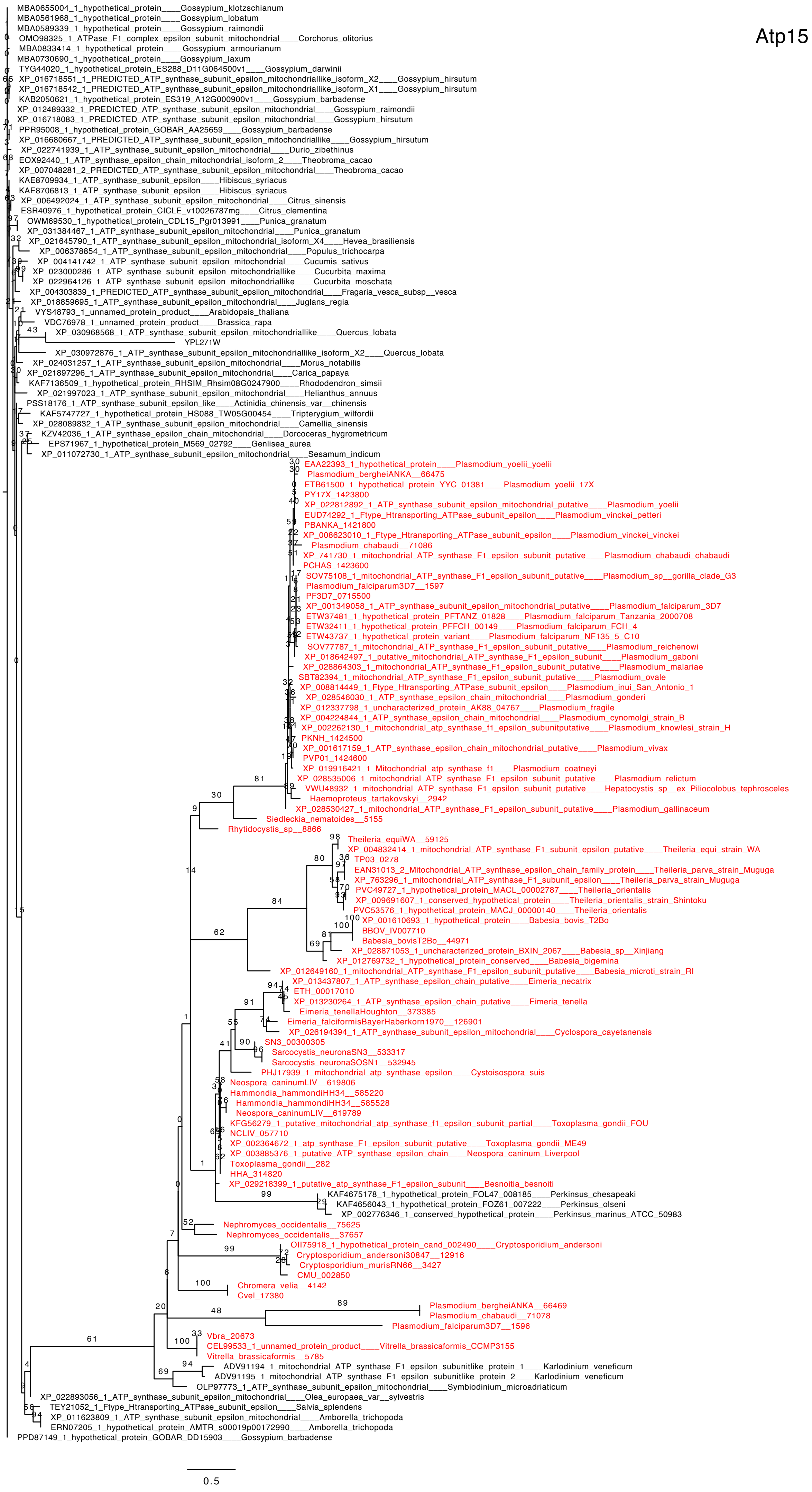
Atp15
MBA0655004_1_hypothetical_protein____Gossypium_klotzschianum
MBA0561968_1_hypothetical_protein____Gossypium_lobatum
MBA0589339_1_hypothetical_protein____Gossypium_raimondii
OMO98325_1_ATPase_F1_complex_epsilon_subunit_mitochondrial____Corchorus_olitorius
MBA0833414_1_hypothetical_protein____Gossypium_armourianum
MBA0730690_1_hypothetical_protein____Gossypium_laxum
TYG44020_1_hypothetical_protein_ES288_D11G064500v1____Gossypium_darwinii
XP_016718551_1_PREDICTED_ATP_synthase_subunit_epsilon_mitochondriallike_isoform_X2____Gossypium_hirsutum
XP_016718542_1_PREDICTED_ATP_synthase_subunit_epsilon_mitochondriallike_isoform_X1____Gossypium_hirsutum
KAB2050621_1_hypothetical_protein_ES319_A12G000900v1____Gossypium_barbadense
XP_012489332_1_PREDICTED_ATP_synthase_subunit_epsilon_mitochondrial____Gossypium_raimondii
XP_016718083_1_PREDICTED_ATP_synthase_subunit_epsilon_mitochondrial____Gossypium_hirsutum
PPR95008_1_hypothetical_protein_GOBAR_AA25659____Gossypium_barbadense
XP_016680667_1_PREDICTED_ATP_synthase_subunit_epsilon_mitochondriallike____Gossypium_hirsutum
XP_022741939_1_ATP_synthase_subunit_epsilon_mitochondrial____Durio_zibethinus
EOX92440_1_ATP_synthase_epsilon_chain_mitochondrial_isoform_2____Theobroma_cacao
XP_007048281_2_PREDICTED_ATP_synthase_subunit_epsilon_mitochondrial____Theobroma_cacao
KAE8709934_1_ATP_synthase_subunit_epsilon____Hibiscus_syriacus
KAE8706813_1_ATP_synthase_subunit_epsilon____Hibiscus_syriacus
XP_006492024_1_ATP_synthase_subunit_epsilon_mitochondrial____Citrus_sinensis
ESR40976_1_hypothetical_protein_CICLE_v10026787mg____Citrus_clementina
OWM69530_1_hypothetical_protein_CDL15_Pgr013991____Punica_granatum
XP_031384467_1_ATP_synthase_subunit_epsilon_mitochondrial____Punica_granatum
XP_021645790_1_ATP_synthase_subunit_epsilon_mitochondrial_isoform_X4____Hevea_brasiliensis
XP_006378854_1_ATP_synthase_subunit_epsilon_mitochondrial____Populus_trichocarpa
XP_004141742_1_ATP_synthase_subunit_epsilon_mitochondrial____Cucumis_sativus
XP_023000286_1_ATP_synthase_subunit_epsilon_mitochondriallike____Cucurbita_maxima
XP_022964126_1_ATP_synthase_subunit_epsilon_mitochondriallike____Cucurbita_moschata
XP_004303839_1_PREDICTED_ATP_synthase_subunit_epsilon_mitochondrial____Fragaria_vesca_subsp__vesca
XP_018859695_1_ATP_synthase_subunit_epsilon_mitochondrial____Juglans_regia
VYS48793_1_unnamed_protein_product____Arabidopsis_thaliana
VDC76978_1_unnamed_protein_product____Brassica_rapa
XP_030968568_1_ATP_synthase_subunit_epsilon_mitochondriallike____Quercus_lobata
YPL271W
XP_030972876_1_ATP_synthase_subunit_epsilon_mitochondriallike_isoform_X2____Quercus_lobata
XP_024031257_1_ATP_synthase_subunit_epsilon_mitochondrial____Morus_notabilis
XP_021897296_1_ATP_synthase_subunit_epsilon_mitochondrial____Carica_papaya
KAF7136509_1_hypothetical_protein_RHSIM_Rhsim08G0247900____Rhododendron_simsii
XP_021997023_1_ATP_synthase_subunit_epsilon_mitochondrial____Helianthus_annuus
PSS18176_1_ATP_synthase_subunit_epsilon_like____Actinidia_chinensis_var__chinensis
KAF5747727_1_hypothetical_protein_HS088_TW05G00454____Tripterygium_wilfordii
XP_028089832_1_ATP_synthase_subunit_epsilon_mitochondrial____Camellia_sinensis
KZV42036_1_ATP_synthase_epsilon_chain_mitochondrial____Dorcoceras_hygrometricum
EPS71967_1_hypothetical_protein_M569_02792____Genlisea_aurea
XP_011072730_1_ATP_synthase_subunit_epsilon_mitochondrial____Sesamum_indicum
EAA22393_1_hypothetical_protein____Plasmodium_yoelii_yoelii
Plasmodium_bergheiANKA__66475
ETB61500_1_hypothetical_protein_YYC_01381____Plasmodium_yoelii_17X
PY17X_1423800
XP_022812892_1_ATP_synthase_subunit_epsilon_mitochondrial_putative____Plasmodium_yoelii
EUD74292_1_Ftype_Htransporting_ATPase_subunit_epsilon____Plasmodium_vinckei_petteri
PBANKA_1421800
XP_008623010_1_Ftype_Htransporting_ATPase_subunit_epsilon____Plasmodium_vinckei_vinckei
Plasmodium_chabaudi__71086
XP_741730_1_mitochondrial_ATP_synthase_F1_epsilon_subunit_putative____Plasmodium_chabaudi_chabaudi
PCHAS_1423600
SOV75108_1_mitochondrial_ATP_synthase_F1_epsilon_subunit_putative____Plasmodium_sp__gorilla_clade_G3
Plasmodium_falciparum3D7__1597
PF3D7_0715500
XP_001349058_1_ATP_synthase_subunit_epsilon_mitochondrial_putative____Plasmodium_falciparum_3D7
ETW37481_1_hypothetical_protein_PFTANZ_01828____Plasmodium_falciparum_Tanzania_2000708
ETW32411_1_hypothetical_protein_PFFCH_00149____Plasmodium_falciparum_FCH_4
ETW43737_1_hypothetical_protein_variant____Plasmodium_falciparum_NF135_5_C10
SOV77787_1_mitochondrial_ATP_synthase_F1_epsilon_subunit_putative____Plasmodium_reichenowi
XP_018642497_1_putative_mitochondrial_ATP_synthase_F1_epsilon_subunit____Plasmodium_gaboni
XP_028864303_1_mitochondrial_ATP_synthase_F1_epsilon_subunit_putative____Plasmodium_malariae
SBT82394_1_mitochondrial_ATP_synthase_F1_epsilon_subunit_putative____Plasmodium_ovale
XP_008814449_1_Ftype_Htransporting_ATPase_subunit_epsilon____Plasmodium_inui_San_Antonio_1
XP_028546030_1_ATP_synthase_epsilon_chain_mitochondrial____Plasmodium_gonderi
XP_012337798_1_uncharacterized_protein_AK88_04767____Plasmodium_fragile
XP_004224844_1_ATP_synthase_epsilon_chain_mitochondrial____Plasmodium_cynomolgi_strain_B
XP_002262130_1_mitochondrial_atp_synthase_f1_epsilon_subunitputative____Plasmodium_knowlesi_strain_H
PKNH_1424500
XP_001617159_1_ATP_synthase_epsilon_chain_mitochondrial_putative____Plasmodium_vivax
PVP01_1424600
XP_019916421_1_Mitochondrial_atp_synthase_f1____Plasmodium_coatneyi
XP_028535006_1_mitochondrial_ATP_synthase_F1_epsilon_subunit_putative____Plasmodium_relictum
VWU48932_1_mitochondrial_ATP_synthase_F1_epsilon_subunit_putative____Hepatocystis_sp__ex_Piliocolobus_tephrosceles
Haemoproteus_tartakovskyi__2942
XP_028530427_1_mitochondrial_ATP_synthase_F1_epsilon_subunit_putative____Plasmodium_gallinaceum
Siedleckia_nematoides__5155
Rhytidocystis_sp__8866
Theileria_equiWA__59125
XP_004832414_1_mitochondrial_ATP_synthase_F1_subunit_epsilon_putative____Theileria_equi_strain_WA
TP03_0278
EAN31013_2_Mitochondrial_ATP_synthase_epsilon_chain_family_protein____Theileria_parva_strain_Muguga
XP_763296_1_mitochondrial_ATP_synthase_F1_subunit_epsilon____Theileria_parva_strain_Muguga
PVC49727_1_hypothetical_protein_MACL_00002787____Theileria_orientalis
XP_009691607_1_conserved_hypothetical_protein____Theileria_orientalis_strain_Shintoku
PVC53576_1_hypothetical_protein_MACJ_00000140____Theileria_orientalis
XP_001610693_1_hypothetical_protein____Babesia_bovis_T2Bo
BBOV_IV007710
Babesia_bovisT2Bo__44971
XP_028871053_1_uncharacterized_protein_BXIN_2067____Babesia_sp__Xinjiang
XP_012769732_1_hypothetical_protein_conserved____Babesia_bigemina
XP_012649160_1_mitochondrial_ATP_synthase_F1_epsilon_subunit_putative____Babesia_microti_strain_RI
XP_013437807_1_ATP_synthase_epsilon_chain_putative____Eimeria_necatrix
ETH_00017010
XP_013230264_1_ATP_synthase_epsilon_chain_putative____Eimeria_tenella
Eimeria_tenellaHoughton__373385
Eimeria_falciformisBayerHaberkorn1970__126901
XP_026194394_1_ATP_synthase_subunit_epsilon_mitochondrial____Cyclospora_cayetanensis
SN3_00300305
Sarcocystis_neuronaSN3__533317
Sarcocystis_neuronaSOSN1__532945
PHJ17939_1_mitochondrial_atp_synthase_epsilon____Cystoisospora_suis
Neospora_caninumLIV__619806
Hammondia_hammondiHH34__585220
Hammondia_hammondiHH34__585528
Neospora_caninumLIV__619789
KFG56279_1_putative_mitochondrial_atp_synthase_f1_epsilon_subunit_partial____Toxoplasma_gondii_FOU
NCLIV_057710
XP_002364672_1_atp_synthase_F1_epsilon_subunit_putative____Toxoplasma_gondii_ME49
XP_003885376_1_putative_ATP_synthase_epsilon_chain____Neospora_caninum_Liverpool
Toxoplasma_gondii__282
HHA_314820
XP_029218399_1_putative_atp_synthase_F1_epsilon_subunit____Besnoitia_besnoiti
KAF4675178_1_hypothetical_protein_FOL47_008185____Perkinsus_chesapeaki
KAF4656043_1_hypothetical_protein_FOZ61_007222____Perkinsus_olseni
XP_002776346_1_conserved_hypothetical_protein____Perkinsus_marinus_ATCC_50983
Nephromyces_occidentalis__75625
Nephromyces_occidentalis__37657
OII75918_1_hypothetical_protein_cand_002490____Cryptosporidium_andersoni
Cryptosporidium_andersoni30847__12916
Cryptosporidium_murisRN66__3427
CMU_002850
Chromera_velia__4142
Cvel_17380
Plasmodium_bergheiANKA__66469
Plasmodium_chabaudi__71078
Plasmodium_falciparum3D7__1596
Vbra_20673
CEL99533_1_unnamed_protein_product____Vitrella_brassicaformis_CCMP3155
Vitrella_brassicaformis__5785
ADV91194_1_mitochondrial_ATP_synthase_F1_epsilon_subunitlike_protein_1____Karlodinium_veneficum
ADV91195_1_mitochondrial_ATP_synthase_F1_epsilon_subunitlike_protein_2____Karlodinium_veneficum
OLP97773_1_ATP_synthase_subunit_epsilon_mitochondrial____Symbiodinium_microadriaticum
XP_022893056_1_ATP_synthase_subunit_epsilon_mitochondrial____Olea_europaea_var__sylvestris
TEY21052_1_Ftype_Htransporting_ATPase_subunit_epsilon____Salvia_splendens
XP_011623809_1_ATP_synthase_subunit_epsilon_mitochondrial____Amborella_trichopoda
ERN07205_1_hypothetical_protein_AMTR_s00019p00172990____Amborella_trichopoda
PPD87149_1_hypothetical_protein_GOBAR_DD15903____Gossypium_barbadense
0.5

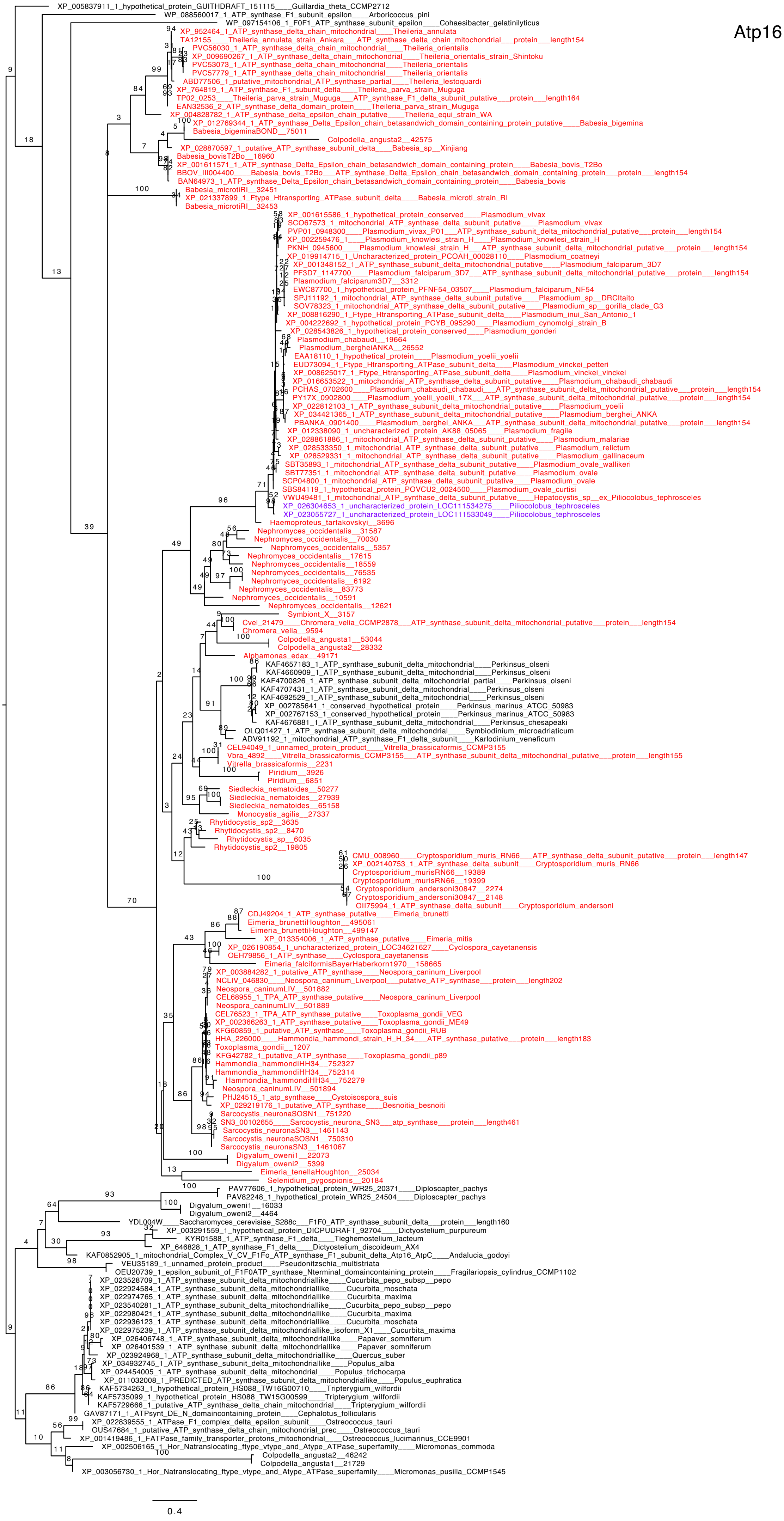
Atp16
XP_005837911_1_hypothetical_protein_GUITHDRAFT_151115____Guillardia_theta_CCMP2712
WP_088560017_1_ATP_synthase_F1_subunit_epsilon____Arboricoccus_pini
WP_097154106_1_F0F1_ATP_synthase_subunit_epsilon____Cohaesibacter_gelatinilyticus
XP_952464_1_ATP_synthase_delta_chain_mitochondrial____Theileria_annulata
TA12155____Theileria_annulata_strain_Ankara___ATP_synthase_delta_chain_mitochondrial___protein___length154
PVC56030_1_ATP_synthase_delta_chain_mitochondrial____Theileria_orientalis
XP_009690267_1_ATP_synthase_delta_chain_mitochondrial____Theileria_orientalis_strain_Shintoku
PVC53073_1_ATP_synthase_delta_chain_mitochondrial____Theileria_orientalis
PVC57779_1_ATP_synthase_delta_chain_mitochondrial____Theileria_orientalis
ABD77506_1_putative_mitochondrial_ATP_synthase_partial____Theileria_lestoquardi
XP_764819_1_ATP_synthase_F1_subunit_delta____Theileria_parva_strain_Muguga
TP02_0253____Theileria_parva_strain_Muguga___ATP_synthase_F1_delta_subunit_putative___protein___length164
EAN32536_2_ATP_synthase_delta_domain_protein____Theileria_parva_strain_Muguga
XP_004828782_1_ATP_synthase_delta_epsilon_chain_putative____Theileria_equi_strain_WA
XP_012769344_1_ATP_synthase_Delta_Epsilon_chain_betasandwich_domain_containing_protein_putative____Babesia_bigemina
Babesia_bigeminaBOND__75011
Colpodella_angusta2__42575
XP_028870597_1_putative_ATP_synthase_subunit_delta____Babesia_sp__Xinjiang
Babesia_bovisT2Bo__16960
XP_001611571_1_ATP_synthase_Delta_Epsilon_chain_betasandwich_domain_containing_protein____Babesia_bovis_T2Bo
BBOV_III004400____Babesia_bovis_T2Bo___ATP_synthase_Delta_Epsilon_chain_betasandwich_domain_containing_protein___protein___length154
BAN64973_1_ATP_synthase_Delta_Epsilon_chain_betasandwich_domain_containing_protein____Babesia_bovis
Babesia_microtiRI__32451
XP_021337899_1_Ftype_Htransporting_ATPase_subunit_delta____Babesia_microti_strain_RI
Babesia_microtiRI__32453
XP_001615586_1_hypothetical_protein_conserved____Plasmodium_vivax
SCO67573_1_mitochondrial_ATP_synthase_delta_subunit_putative____Plasmodium_vivax
PVP01_0948300____Plasmodium_vivax_P01___ATP_synthase_subunit_delta_mitochondrial_putative___protein___length154
XP_002259476_1____Plasmodium_knowlesi_strain_H____Plasmodium_knowlesi_strain_H
PKNH_0945600____Plasmodium_knowlesi_strain_H___ATP_synthase_subunit_delta_mitochondrial_putative___protein___length154
XP_019914715_1_Uncharacterized_protein_PCOAH_00028110____Plasmodium_coatneyi
XP_001348152_1_ATP_synthase_subunit_delta_mitochondrial_putative____Plasmodium_falciparum_3D7
PF3D7_1147700____Plasmodium_falciparum_3D7___ATP_synthase_subunit_delta_mitochondrial_putative___protein___length154
Plasmodium_falciparum3D7__3312
EWC87700_1_hypothetical_protein_PFNF54_03507____Plasmodium_falciparum_NF54
SPJ11192_1_mitochondrial_ATP_synthase_delta_subunit_putative____Plasmodium_sp__DRC1taito
SOV78323_1_mitochondrial_ATP_synthase_delta_subunit_putative____Plasmodium_sp__gorilla_clade_G3
XP_008816290_1_Ftype_Htransporting_ATPase_subunit_delta____Plasmodium_inui_San_Antonio_1
XP_004222692_1_hypothetical_protein_PCYB_095290____Plasmodium_cynomolgi_strain_B
XP_028543826_1_hypothetical_protein_conserved____Plasmodium_gonderi
Plasmodium_chabaudi__19664
Plasmodium_bergheiANKA__26552
EAA18110_1_hypothetical_protein____Plasmodium_yoelii_yoelii
EUD73094_1_Ftype_Htransporting_ATPase_subunit_delta____Plasmodium_vinckei_petteri
XP_008625017_1_Ftype_Htransporting_ATPase_subunit_delta____Plasmodium_vinckei_vinckei
XP_016653522_1_mitochondrial_ATP_synthase_delta_subunit_putative____Plasmodium_chabaudi_chabaudi
PCHAS_0702600____Plasmodium_chabaudi_chabaudi___ATP_synthase_subunit_delta_mitochondrial_putative___protein___length154
PY17X_0902800____Plasmodium_yoelii_yoelii_17X___ATP_synthase_subunit_delta_mitochondrial_putative___protein___length154
XP_022812103_1_ATP_synthase_subunit_delta_mitochondrial_putative____Plasmodium_yoelii
XP_034421365_1_ATP_synthase_subunit_delta_mitochondrial_putative____Plasmodium_berghei_ANKA
PBANKA_0901400____Plasmodium_berghei_ANKA___ATP_synthase_subunit_delta_mitochondrial_putative___protein___length154
XP_012338090_1_uncharacterized_protein_AK88_05065____Plasmodium_fragile
XP_028861886_1_mitochondrial_ATP_synthase_delta_subunit_putative____Plasmodium_malariae
XP_028533350_1_mitochondrial_ATP_synthase_delta_subunit_putative____Plasmodium_relictum
XP_028529331_1_mitochondrial_ATP_synthase_delta_subunit_putative____Plasmodium_gallinaceum
SBT35893_1_mitochondrial_ATP_synthase_delta_subunit_putative____Plasmodium_ovale_wallikeri
SBT77351_1_mitochondrial_ATP_synthase_delta_subunit_putative____Plasmodium_ovale
SCP04800_1_mitochondrial_ATP_synthase_delta_subunit_putative____Plasmodium_ovale
SBS84119_1_hypothetical_protein_POVCU2_0024500____Plasmodium_ovale_curtisi
VWU49481_1_mitochondrial_ATP_synthase_delta_subunit_putative____Hepatocystis_sp__ex_Piliocolobus_tephrosceles
XP_026304653_1_uncharacterized_protein_LOC111534275____Piliocolobus_tephrosceles
XP_023055727_1_uncharacterized_protein_LOC111533049____Piliocolobus_tephrosceles
Haemoproteus_tartakovskyi__3696
Nephromyces_occidentalis__31587
Nephromyces_occidentalis__70030
Nephromyces_occidentalis__5357
Nephromyces_occidentalis__17615
Nephromyces_occidentalis__18559
Nephromyces_occidentalis__76535
Nephromyces_occidentalis__6192
Nephromyces_occidentalis__83773
Nephromyces_occidentalis__10591
Nephromyces_occidentalis__12621
Symbiont_X__3157
Cvel_21479____Chromera_velia_CCMP2878___ATP_synthase_subunit_delta_mitochondrial_putative___protein___length154
Chromera_velia__9594
Colpodella_angusta1__53044
Colpodella_angusta2__28332
Alphamonas_edax__49171
KAF4657183_1_ATP_synthase_subunit_delta_mitochondrial____Perkinsus_olseni
KAF4660909_1_ATP_synthase_subunit_delta_mitochondrial____Perkinsus_olseni
KAF4700826_1_ATP_synthase_subunit_delta_mitochondrial_partial____Perkinsus_olseni
KAF4707431_1_ATP_synthase_subunit_delta_mitochondrial____Perkinsus_olseni
KAF4692529_1_ATP_synthase_subunit_delta_mitochondrial____Perkinsus_olseni
XP_002785641_1_conserved_hypothetical_protein____Perkinsus_marinus_ATCC_50983
XP_002767153_1_conserved_hypothetical_protein____Perkinsus_marinus_ATCC_50983
KAF4676881_1_ATP_synthase_subunit_delta_mitochondrial____Perkinsus_chesapeaki
OLQ01427_1_ATP_synthase_subunit_delta_mitochondrial____Symbiodinium_microadriaticum
ADV91192_1_mitochondrial_ATP_synthase_F1_delta_subunit____Karlodinium_veneficum
CEL94049_1_unnamed_protein_product____Vitrella_brassicaformis_CCMP3155
Vbra_4892____Vitrella_brassicaformis_CCMP3155___ATP_synthase_subunit_delta_mitochondrial_putative___protein___length155
Vitrella_brassicaformis__2231
Piridium__3926
Piridium__6851
Siedleckia_nematoides__50277
Siedleckia_nematoides__27939
Siedleckia_nematoides__65158
Monocystis_agilis__27337
Rhytidocystis_sp2__3635
Rhytidocystis_sp2__8470
Rhytidocystis_sp__6035
Rhytidocystis_sp2__19805
CMU_008960____Cryptosporidium_muris_RN66___ATP_synthase_delta_subunit_putative___protein___length147
XP_002140753_1_ATP_synthase_delta_subunit____Cryptosporidium_muris_RN66
Cryptosporidium_murisRN66__19389
Cryptosporidium_murisRN66__19399
Cryptosporidium_andersoni30847__2274
Cryptosporidium_andersoni30847__2148
OII75994_1_ATP_synthase_delta_subunit____Cryptosporidium_andersoni
CDJ49204_1_ATP_synthase_putative____Eimeria_brunetti
Eimeria_brunettiHoughton__495061
Eimeria_brunettiHoughton__499147
XP_013354006_1_ATP_synthase_putative____Eimeria_mitis
XP_026190854_1_uncharacterized_protein_LOC34621627____Cyclospora_cayetanensis
OEH79856_1_ATP_synthase____Cyclospora_cayetanensis
Eimeria_falciformisBayerHaberkorn1970__158665
XP_003884282_1_putative_ATP_synthase____Neospora_caninum_Liverpool
NCLIV_046830____Neospora_caninum_Liverpool___putative_ATP_synthase___protein___length202
Neospora_caninumLIV__501882
CEL68955_1_TPA_ATP_synthase_putative____Neospora_caninum_Liverpool
Neospora_caninumLIV__501889
CEL76523_1_TPA_ATP_synthase_putative____Toxoplasma_gondii_VEG
XP_002366263_1_ATP_synthase_putative____Toxoplasma_gondii_ME49
KFG60859_1_putative_ATP_synthase____Toxoplasma_gondii_RUB
HHA_226000____Hammondia_hammondi_strain_H_H_34___ATP_synthase_putative___protein___length183
Toxoplasma_gondii__1207
KFG42782_1_putative_ATP_synthase____Toxoplasma_gondii_p89
Hammondia_hammondiHH34__752327
Hammondia_hammondiHH34__752314
Hammondia_hammondiHH34__752279
Neospora_caninumLIV__501894
PHJ24515_1_atp_synthase____Cystoisospora_suis
XP_029219176_1_putative_ATP_synthase____Besnoitia_besnoiti
Sarcocystis_neuronaSOSN1__751220
SN3_00102655____Sarcocystis_neurona_SN3___atp_synthase___protein___length461
Sarcocystis_neuronaSN3__1461143
Sarcocystis_neuronaSOSN1__750310
Sarcocystis_neuronaSN3__1461067
Digyalum_oweni1__22073
Digyalum_oweni2__5399
Eimeria_tenellaHoughton__25034
Selenidium_pygospionis__20184
PAV77606_1_hypothetical_protein_WR25_20371____Diploscapter_pachys
PAV82248_1_hypothetical_protein_WR25_24504____Diploscapter_pachys
Digyalum_oweni1__16033
Digyalum_oweni2__4464
YDL004W____Saccharomyces_cerevisiae_S288c___F1F0_ATP_synthase_subunit_delta___protein___length160
XP_003291559_1_hypothetical_protein_DICPUDRAFT_92704____Dictyostelium_purpureum
KYR01588_1_ATP_synthase_F1_delta____Tieghemostelium_lacteum
XP_646828_1_ATP_synthase_F1_delta____Dictyostelium_discoideum_AX4
KAF0852905_1_mitochondrial_Complex_V_CV_F1Fo_ATP_synthase_F1_subunit_delta_Atp16_AtpC____Andalucia_godoyi
VEU35189_1_unnamed_protein_product____Pseudonitzschia_multistriata
OEU20739_1_epsilon_subunit_of_F1F0ATP_synthase_Nterminal_domaincontaining_protein____Fragilariopsis_cylindrus_CCMP1102
XP_023528709_1_ATP_synthase_subunit_delta_mitochondriallike____Cucurbita_pepo_subsp__pepo
XP_022924584_1_ATP_synthase_subunit_delta_mitochondriallike____Cucurbita_moschata
XP_022974765_1_ATP_synthase_subunit_delta_mitochondriallike____Cucurbita_maxima
XP_023540281_1_ATP_synthase_subunit_delta_mitochondriallike____Cucurbita_pepo_subsp__pepo
XP_022980421_1_ATP_synthase_subunit_delta_mitochondriallike____Cucurbita_maxima
XP_022936123_1_ATP_synthase_subunit_delta_mitochondriallike____Cucurbita_moschata
XP_022975239_1_ATP_synthase_subunit_delta_mitochondriallike_isoform_X1____Cucurbita_maxima
XP_026406748_1_ATP_synthase_subunit_delta_mitochondriallike____Papaver_somniferum
XP_026401539_1_ATP_synthase_subunit_delta_mitochondriallike____Papaver_somniferum
XP_023924968_1_ATP_synthase_subunit_delta_mitochondriallike____Quercus_suber
XP_034932745_1_ATP_synthase_subunit_delta_mitochondriallike____Populus_alba
XP_024454005_1_ATP_synthase_subunit_delta_mitochondrial____Populus_trichocarpa
XP_011032008_1_PREDICTED_ATP_synthase_subunit_delta_mitochondriallike____Populus_euphratica
KAF5734263_1_hypothetical_protein_HS088_TW16G00710____Tripterygium_wilfordii
KAF5735099_1_hypothetical_protein_HS088_TW15G00599____Tripterygium_wilfordii
KAF5729666_1_putative_ATP_synthase_delta_chain_mitochondrial____Tripterygium_wilfordii
GAV87171_1_ATPsynt_DE_N_domaincontaining_protein____Cephalotus_follicularis
XP_022839555_1_ATPase_F1_complex_delta_epsilon_subunit____Ostreococcus_tauri
OUS47684_1_putative_ATP_synthase_delta_chain_mitochondrial_prec____Ostreococcus_tauri
XP_001419486_1_FATPase_family_transporter_protons_mitochondrial____Ostreococcus_lucimarinus_CCE9901
XP_002506165_1_Hor_Natranslocating_ftype_vtype_and_Atype_ATPase_superfamily____Micromonas_commoda
Colpodella_angusta2__46242
Colpodella_angusta1__21729
XP_003056730_1_Hor_Natranslocating_ftype_vtype_and_Atype_ATPase_superfamily____Micromonas_pusilla_CCMP1545
0.4

Atp2

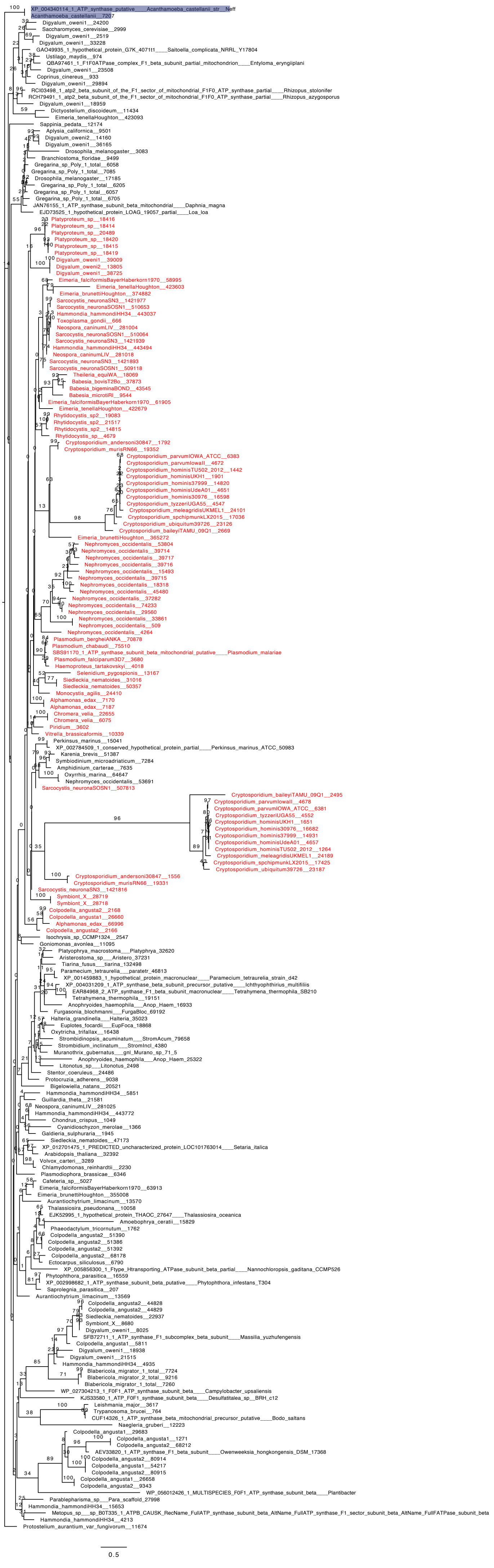

Atp3

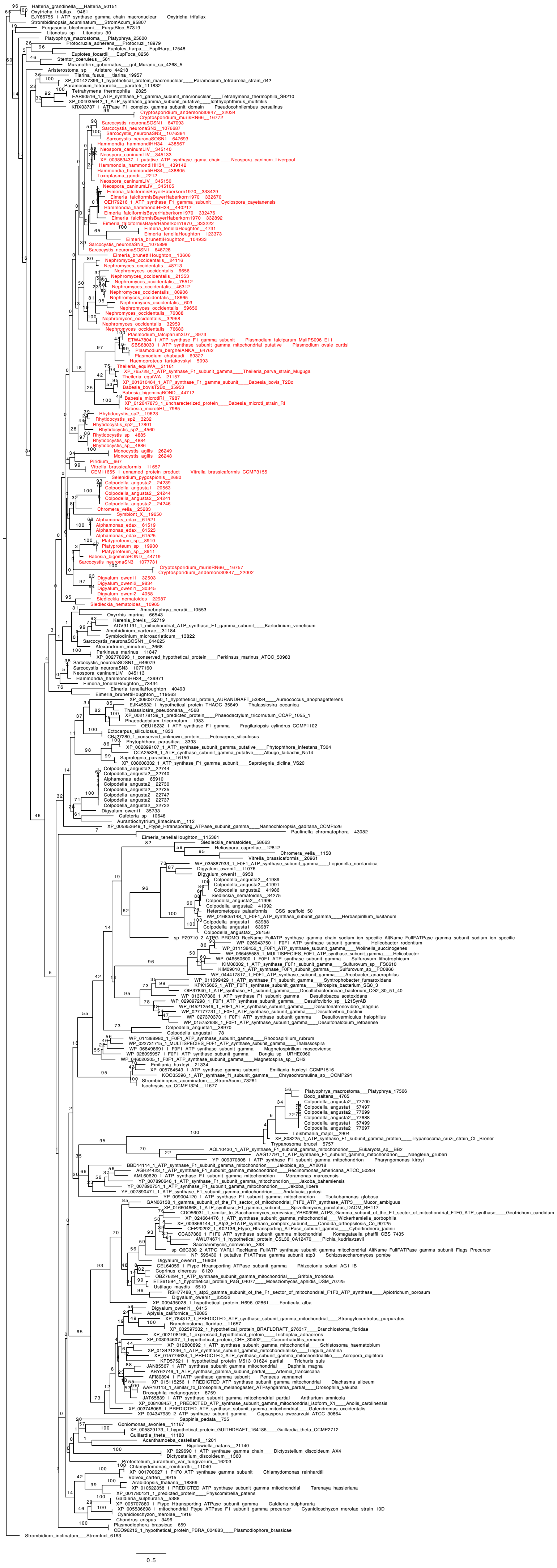

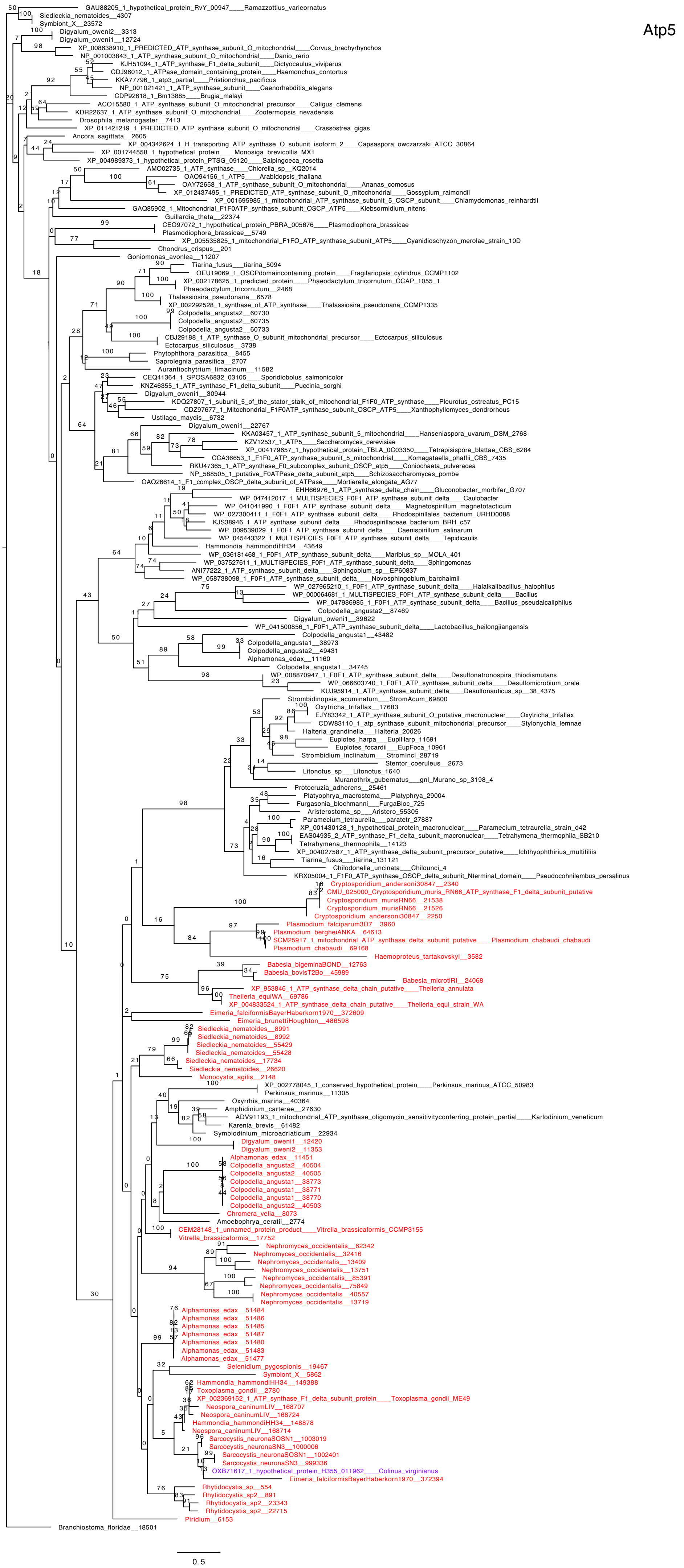

Atp5
GAU88205_1_hypothetical_protein_RvY_00947____Ramazzottius_varieornatus
Siedleckia_nematoides__4307
Symbiont_X__23572
Digyalum_oweni2__3313
Digyalum_oweni1__12724
XP_008638910_1_PREDICTED_ATP_synthase_subunit_O_mitochondrial____Corvus_brachyrhynchos
NP_001003843_1_ATP_synthase_subunit_O_mitochondrial____Danio_rerio
KJH51094_1_ATP_synthase_F1_delta_subunit____Dictyocaulus_viviparus
CDJ96012_1_ATPase_domain_containing_protein____Haemonchus_contortus
KKA77796_1_atp3_partial____Pristionchus_pacificus
NP_001021421_1_ATP_synthase_subunit____Caenorhabditis_elegans
CDP92618_1_Bm13885____Brugia_malayi
ACO15580_1_ATP_synthase_subunit_O_mitochondrial_precursor____Caligus_clemensi
KDR22637_1_ATP_synthase_subunit_O_mitochondrial____Zootermopsis_nevadensis
Drosophila_melanogaster__7413
XP_011421219_1_PREDICTED_ATP_synthase_subunit_O_mitochondrial____Crassostrea_gigas
Ancora_sagittata__2605
XP_004342624_1_H_transporting_ATP_synthase_O_subunit_isoform_2____Capsaspora_owczarzaki_ATCC_30864
XP_001744558_1_hypothetical_protein____Monosiga_brevicollis_MX1
XP_004989373_1_hypothetical_protein_PTSG_09120____Salpingoeca_rosetta
AMO02735_1_ATP_synthase____Chlorella_sp__KQ2014
OAO94156_1_ATP5____Arabidopsis_thaliana
OAY72658_1_ATP_synthase_subunit_O_mitochondrial____Ananas_comosus
XP_012437495_1_PREDICTED_ATP_synthase_subunit_O_mitochondrial____Gossypium_raimondii
XP_001695985_1_mitochondrial_ATP_synthase_subunit_5_OSCP_subunit____Chlamydomonas_reinhardtii
GAQ85902_1_Mitochondrial_F1F0ATP_synthase_subunit_OSCP_ATP5____Klebsormidium_nitens
Guillardia_theta__22374
CEO97072_1_hypothetical_protein_PBRA_005676____Plasmodiophora_brassicae
Plasmodiophora_brassicae__5749
XP_005535825_1_mitochondrial_F1FO_ATP_synthase_subunit_ATP5____Cyanidioschyzon_merolae_strain_10D
Chondrus_crispus__201
Goniomonas_avonlea__11207
Tiarina_fusus__tiarina_5094
OEU19069_1_OSCPdomaincontaining_protein____Fragilariopsis_cylindrus_CCMP1102
XP_002178625_1_predicted_protein____Phaeodactylum_tricornutum_CCAP_1055_1
Phaeodactylum_tricornutum__2468
Thalassiosira_pseudonana__6578
XP_002292528_1_synthase_of_ATP_synthase____Thalassiosira_pseudonana_CCMP1335
Colpodella_angusta2__60730
Colpodella_angusta2__60735
Colpodella_angusta2__60733
CBJ29188_1_ATP_synthase_O_subunit_mitochondrial_precursor____Ectocarpus_siliculosus
Ectocarpus_siliculosus__3738
Phytophthora_parasitica__8455
Saprolegnia_parasitica__2707
Aurantiochytrium_limacinum__11582
CEQ41364_1_SPOSA6832_03105____Sporidiobolus_salmonicolor
KNZ46355_1_ATP_synthase_F1_delta_subunit____Puccinia_sorghi
Digyalum_oweni1__30944
KDQ27807_1_subunit_5_of_the_stator_stalk_of_mitochondrial_F1F0_ATP_synthase____Pleurotus_ostreatus_PC15
CDZ97677_1_Mitochondrial_F1F0ATP_synthase_subunit_OSCP_ATP5____Xanthophyllomyces_dendrorhous
Ustilago_maydis__6732
Digyalum_oweni1__22767
KKA03457_1_ATP_synthase_subunit_5_mitochondrial____Hanseniaspora_uvarum_DSM_2768
KZV12537_1_ATP5____Saccharomyces_cerevisiae
XP_004179657_1_hypothetical_protein_TBLA_0C03350____Tetrapisispora_blattae_CBS_6284
CCA36653_1_F1F0_ATP_synthase_subunit_5_mitochondrial____Komagataella_phaffii_CBS_7435
RKU47365_1_ATP_synthase_F0_subcomplex_subunit_OSCP_atp5____Coniochaeta_pulveracea
NP_588505_1_putative_F0ATPase_delta_subunit_atp5____Schizosaccharomyces_pombe
OAQ26614_1_F1_complex_OSCP_delta_subunit_of_ATPase____Mortierella_elongata_AG77
EHH66976_1_ATP_synthase_delta_chain____Gluconobacter_morbifer_G707
WP_047412017_1_MULTISPECIES_F0F1_ATP_synthase_subunit_delta____Caulobacter
WP_041041990_1_F0F1_ATP_synthase_subunit_delta____Magnetospirillum_magnetotacticum
WP_027300411_1_F0F1_ATP_synthase_subunit_delta____Rhodospirillales_bacterium_URHD0088
KJS38946_1_ATP_synthase_subunit_delta____Rhodospirillaceae_bacterium_BRH_c57
WP_009539029_1_F0F1_ATP_synthase_subunit_delta____Caenispirillum_salinarum
WP_045443322_1_MULTISPECIES_F0F1_ATP_synthase_subunit_delta____Tepidicaulis
Hammondia_hammondiHH34__43649
WP_036181468_1_F0F1_ATP_synthase_subunit_delta____Maribius_sp__MOLA_401
WP_037527611_1_MULTISPECIES_F0F1_ATP_synthase_subunit_delta____Sphingomonas
ANI77222_1_ATP_synthase_subunit_delta____Sphingobium_sp__EP60837
WP_058738098_1_F0F1_ATP_synthase_subunit_delta____Novosphingobium_barchaimii
WP_027965210_1_F0F1_ATP_synthase_subunit_delta____Halalkalibacillus_halophilus
WP_000064681_1_MULTISPECIES_F0F1_ATP_synthase_subunit_delta____Bacillus
WP_047986985_1_F0F1_ATP_synthase_subunit_delta____Bacillus_pseudalcaliphilus
Colpodella_angusta2__87469
Digyalum_oweni1__39622
WP_041500856_1_F0F1_ATP_synthase_subunit_delta____Lactobacillus_heilongjiangensis
Colpodella_angusta1__43482
Colpodella_angusta1__38973
Colpodella_angusta2__49431
Alphamonas_edax__11160
Colpodella_angusta1__34745
WP_008870947_1_F0F1_ATP_synthase_subunit_delta____Desulfonatronospira_thiodismutans
WP_066603740_1_F0F1_ATP_synthase_subunit_delta____Desulfomicrobium_orale
KUJ95914_1_ATP_synthase_subunit_delta____Desulfonauticus_sp__38_4375
Strombidinopsis_acuminatum__StromAcum_69800
Oxytricha_trifallax__17683
EJY83342_1_ATP_synthase_subunit_O_putative_macronuclear____Oxytricha_trifallax
CDW83110_1_atp_synthase_subunit_mitochondrial_precursor____Stylonychia_lemnae
Halteria_grandinella__Halteria_20026
Euplotes_harpa__EuplHarp_11691
Euplotes_focardii__EupFoca_10961
Strombidium_inclinatum__StromIncl_28719
Stentor_coeruleus__2673
Litonotus_sp__Litonotus_1640
Muranothrix_gubernatus__gnl_Murano_sp_3198_4
Protocruzia_adherens__25461
Platyophrya_macrostoma__Platyphrya_29004
Furgasonia_blochmanni__FurgaBloc_725
Aristerostoma_sp__Aristero_55305
Paramecium_tetraurelia__paratetr_27887
XP_001430128_1_hypothetical_protein_macronuclear____Paramecium_tetraurelia_strain_d42
EAS04935_2_ATP_synthase_F1_delta_subunit_macronuclear____Tetrahymena_thermophila_SB210
Tetrahymena_thermophila__14123
XP_004027587_1_ATP_synthase_delta_subunit_precursor_putative____Ichthyophthirius_multifiliis
Tiarina_fusus__tiarina_131121
Chilodonella_uncinata__Chilounci_4
KRX05004_1_F1F0_ATP_synthase_OSCP_delta_subunit_Nterminal_domain____Pseudocohnilembus_persalinus
Cryptosporidium_andersoni30847__2340
CMU_025000_Cryptosporidium_muris_RN66_ATP_synthase_F1_delta_subunit_putative
Cryptosporidium_murisRN66__21538
Cryptosporidium_murisRN66__21526
Cryptosporidium_andersoni30847__2250
Plasmodium_falciparum3D7__3960
Plasmodium_bergheiANKA__64613
SCM25917_1_mitochondrial_ATP_synthase_delta_subunit_putative____Plasmodium_chabaudi_chabaudi
Plasmodium_chabaudi__69168
Haemoproteus_tartakovskyi__3582
Babesia_bigeminaBOND__12763
Babesia_bovisT2Bo__45989
Babesia_microtiRI__24068
XP_953846_1_ATP_synthase_delta_chain_putative____Theileria_annulata
Theileria_equiWA__69786
XP_004833524_1_ATP_synthase_delta_chain_putative____Theileria_equi_strain_WA
Eimeria_falciformisBayerHaberkorn1970__372609
Eimeria_brunettiHoughton__486598
Siedleckia_nematoides__8991
Siedleckia_nematoides__8992
Siedleckia_nematoides__55429
Siedleckia_nematoides__55428
Siedleckia_nematoides__17734
Siedleckia_nematoides__26620
Monocystis_agilis__2148
XP_002778045_1_conserved_hypothetical_protein____Perkinsus_marinus_ATCC_50983
Perkinsus_marinus__11305
Oxyrrhis_marina__40364
Amphidinium_carterae__27630
ADV91193_1_mitochondrial_ATP_synthase_oligomycin_sensitivityconferring_protein_partial____Karlodinium_veneficum
Karenia_brevis__61482
Symbiodinium_microadriaticum__22934
Digyalum_oweni1__12420
Digyalum_oweni2__11353
Alphamonas_edax__11451
Colpodella_angusta2__40504
Colpodella_angusta2__40505
Colpodella_angusta1__38773
Colpodella_angusta1__38771
Colpodella_angusta1__38770
Colpodella_angusta2__40503
Chromera_velia__8073
Amoebophrya_ceratii__2774
CEM28148_1_unnamed_protein_product____Vitrella_brassicaformis_CCMP3155
Vitrella_brassicaformis__17752
Nephromyces_occidentalis__62342
Nephromyces_occidentalis__32416
Nephromyces_occidentalis__13409
Nephromyces_occidentalis__13751
Nephromyces_occidentalis__85391
Nephromyces_occidentalis__75849
Nephromyces_occidentalis__40557
Nephromyces_occidentalis__13719
Alphamonas_edax__51484
Alphamonas_edax__51486
Alphamonas_edax__51485
Alphamonas_edax__51487
Alphamonas_edax__51480
Alphamonas_edax__51483
Alphamonas_edax__51477
Selenidium_pygospionis__19467
Symbiont_X__5862
Hammondia_hammondiHH34__149388
Toxoplasma_gondii__2780
XP_002369152_1_ATP_synthase_F1_delta_subunit_protein____Toxoplasma_gondii_ME49
Neospora_caninumLIV__168707
Neospora_caninumLIV__168724
Hammondia_hammondiHH34__148878
Neospora_caninumLIV__168714
Sarcocystis_neuronaSOSN1__1003019
Sarcocystis_neuronaSN3__1000006
Sarcocystis_neuronaSOSN1__1002401
Sarcocystis_neuronaSN3__999336
OXB71617_1_hypothetical_protein_H355_011962____Colinus_virginianus
Eimeria_falciformisBayerHaberkorn1970__372394
Rhytidocystis_sp__554
Rhytidocystis_sp2__891
Rhytidocystis_sp2__23343
Rhytidocystis_sp2__22715
Piridium__6153
Branchiostoma_floridae__18501
0.5

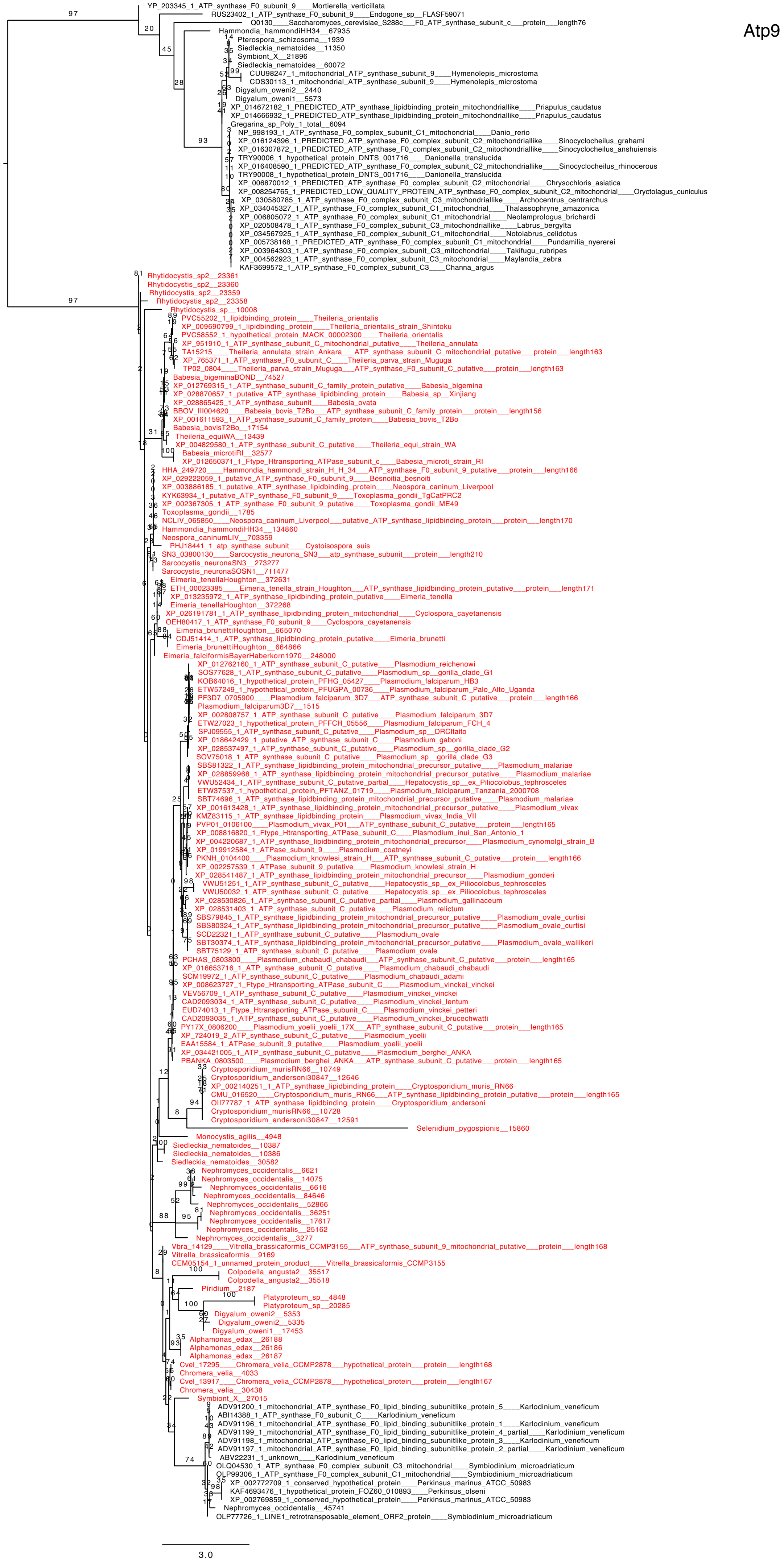
Atp9
YP_203345_1_ATP_synthase_F0_subunit_9____Mortierella_verticillata
RUS23402_1_ATP_synthase_F0_subunit_9____Endogone_sp__FLASF59071
Q0130____Saccharomyces_cerevisiae_S288c___F0_ATP_synthase_subunit_c___protein___length76
Hammondia_hammondiHH34__67935
Pterospora_schizosoma__1939
Siedleckia_nematoides__11350
Symbiont_X__21896
Siedleckia_nematoides__60072
CUU98247_1_mitochondrial_ATP_synthase_subunit_9____Hymenolepis_microstoma
CDS30113_1_mitochondrial_ATP_synthase_subunit_9____Hymenolepis_microstoma
Digyalum_oweni2__2440
Digyalum_oweni1__5573
XP_014672182_1_PREDICTED_ATP_synthase_lipidbinding_protein_mitochondriallike____Priapulus_caudatus
XP_014666932_1_PREDICTED_ATP_synthase_lipidbinding_protein_mitochondriallike____Priapulus_caudatus
Gregarina_sp_Poly_1_total__6094
NP_998193_1_ATP_synthase_F0_complex_subunit_C1_mitochondrial____Danio_rerio
XP_016124396_1_PREDICTED_ATP_synthase_F0_complex_subunit_C2_mitochondriallike____Sinocyclocheilus_grahami
XP_016307872_1_PREDICTED_ATP_synthase_F0_complex_subunit_C2_mitochondriallike____Sinocyclocheilus_anshuiensis
TRY90006_1_hypothetical_protein_DNTS_001716____Danionella_translucida
XP_016408590_1_PREDICTED_ATP_synthase_F0_complex_subunit_C2_mitochondriallike____Sinocyclocheilus_rhinocerous
TRY90008_1_hypothetical_protein_DNTS_001716____Danionella_translucida
XP_006870012_1_PREDICTED_ATP_synthase_F0_complex_subunit_C2_mitochondrial____Chrysochloris_asiatica
XP_008254765_1_PREDICTED_LOW_QUALITY_PROTEIN_ATP_synthase_F0_complex_subunit_C2_mitochondrial____Oryctolagus_cuniculus
XP_030580785_1_ATP_synthase_F0_complex_subunit_C3_mitochondriallike____Archocentrus_centrarchus
XP_034045327_1_ATP_synthase_F0_complex_subunit_C1_mitochondrial____Thalassophryne_amazonica
XP_006805072_1_ATP_synthase_F0_complex_subunit_C1_mitochondrial____Neolamprologus_brichardi
XP_020508478_1_ATP_synthase_F0_complex_subunit_C3_mitochondriallike____Labrus_bergylta
XP_034567925_1_ATP_synthase_F0_complex_subunit_C1_mitochondrial____Notolabrus_celidotus
XP_005738168_1_PREDICTED_ATP_synthase_F0_complex_subunit_C1_mitochondrial____Pundamilia_nyererei
XP_003964303_1_ATP_synthase_F0_complex_subunit_C3_mitochondrial____Takifugu_rubripes
XP_004562923_1_ATP_synthase_F0_complex_subunit_C3_mitochondrial____Maylandia_zebra
KAF3699572_1_ATP_synthase_F0_complex_subunit_C3____Channa_argus
Rhytidocystis_sp2__23361
Rhytidocystis_sp2__23360
Rhytidocystis_sp2__23359
Rhytidocystis_sp2__23358
Rhytidocystis_sp__10008
PVC55202_1_lipidbinding_protein____Theileria_orientalis
XP_009690799_1_lipidbinding_protein____Theileria_orientalis_strain_Shintoku
PVC58552_1_hypothetical_protein_MACK_00002300____Theileria_orientalis
XP_951910_1_ATP_synthase_subunit_C_mitochondrial_putative____Theileria_annulata
TA15215____Theileria_annulata_strain_Ankara___ATP_synthase_subunit_C_mitochondrial_putative___protein___length163
XP_765371_1_ATP_synthase_subunit_C____Theileria_parva_strain_Muguga
TP02_0804____Theileria_parva_strain_Muguga___ATP_synthase_F0_subunit_C_putative___protein___length163
Babesia_bigeminaBOND__74527
XP_012769315_1_ATP_synthase_subunit_C_family_protein_putative____Babesia_bigemina
XP_028870657_1_putative_ATP_synthase_lipidbinding_protein____Babesia_sp__Xinjiang
XP_028865425_1_ATP_synthase_subunit____Babesia_ovata
BBOV_III004620____Babesia_bovis_T2Bo___ATP_synthase_subunit_C_family_protein___protein___length156
XP_001611593_1_ATP_synthase_subunit_C_family_protein____Babesia_bovis_T2Bo
Babesia_bovisT2Bo__17154
Theileria_equiWA__13439
XP_004829580_1_ATP_synthase_subunit_C_putative____Theileria_equi_strain_WA
Babesia_microtiRI__32577
XP_012650371_1_Ftype_Htransporting_ATPase_subunit_c____Babesia_microti_strain_RI
HHA_249720____Hammondia_hammondi_strain_H_H_34___ATP_synthase_F0_subunit_9_putative___protein___length166
XP_029222059_1_putative_ATP_synthase_F0_subunit_9____Besnoitia_besnoiti
XP_003886185_1_putative_ATP_synthase_lipidbinding_protein____Neospora_caninum_Liverpool
KYK63934_1_putative_ATP_synthase_F0_subunit_9____Toxoplasma_gondii_TgCatPRC2
XP_002367305_1_ATP_synthase_F0_subunit_9_putative____Toxoplasma_gondii_ME49
Toxoplasma_gondii__1785
NCLIV_065850____Neospora_caninum_Liverpool___putative_ATP_synthase_lipidbinding_protein___protein___length170
Hammondia_hammondiHH34__134860
Neospora_caninumLIV__703359
PHJ18441_1_atp_synthase_subunit____Cystoisospora_suis
SN3_03800130____Sarcocystis_neurona_SN3___atp_synthase_subunit___protein___length210
Sarcocystis_neuronaSN3__273277
Sarcocystis_neuronaSOSN1__711477
Eimeria_tenellaHoughton__372631
ETH_00023385____Eimeria_tenella_strain_Houghton___ATP_synthase_lipidbinding_protein_putative___protein___length171
XP_013235972_1_ATP_synthase_lipidbinding_protein_putative____Eimeria_tenella
Eimeria_tenellaHoughton__372268
XP_026191781_1_ATP_synthase_lipidbinding_protein_mitochondrial____Cyclospora_cayetanensis
OEH80417_1_ATP_synthase_F0_subunit_9____Cyclospora_cayetanensis
Eimeria_brunettiHoughton__665070
CDJ51414_1_ATP_synthase_lipidbinding_protein_putative____Eimeria_brunetti
Eimeria_brunettiHoughton__664866
Eimeria_falciformisBayerHaberkorn1970__248000
XP_012762160_1_ATP_synthase_subunit_C_putative____Plasmodium_reichenowi
SOS77628_1_ATP_synthase_subunit_C_putative____Plasmodium_sp__gorilla_clade_G1
KOB64016_1_hypothetical_protein_PFHG_05427____Plasmodium_falciparum_HB3
ETW57249_1_hypothetical_protein_PFUGPA_00736____Plasmodium_falciparum_Palo_Alto_Uganda
PF3D7_0705900____Plasmodium_falciparum_3D7___ATP_synthase_subunit_C_putative___protein___length166
Plasmodium_falciparum3D7__1515
XP_002808757_1_ATP_synthase_subunit_C_putative____Plasmodium_falciparum_3D7
ETW27023_1_hypothetical_protein_PFFCH_05556____Plasmodium_falciparum_FCH_4
SPJ09555_1_ATP_synthase_subunit_C_putative____Plasmodium_sp__DRC-Itaito
XP_018642429_1_putative_ATP_synthase_subunit_C____Plasmodium_gaboni
XP_028537497_1_ATP_synthase_subunit_C_putative____Plasmodium_sp__gorilla_clade_G2
SOV75018_1_ATP_synthase_subunit_C_putative____Plasmodium_sp__gorilla_clade_G3
SBS81322_1_ATP_synthase_lipidbinding_protein_mitochondrial_precursor_putative____Plasmodium_malariae
XP_028859968_1_ATP_synthase_lipidbinding_protein_mitochondrial_precursor_putative____Plasmodium_malariae
VWU52434_1_ATP_synthase_subunit_C_putative_partial____Hepatocystis_sp__ex_Piliocolobus_tephrosceles
ETW37537_1_hypothetical_protein_PFTANZ_01719____Plasmodium_falciparum_Tanzania_2000708
SBT74696_1_ATP_synthase_lipidbinding_protein_mitochondrial_precursor_putative____Plasmodium_malariae
XP_001613428_1_ATP_synthase_lipidbinding_protein_mitochondrial_precursor_putative____Plasmodium_vivax
KMZ83115_1_ATP_synthase_lipidbinding_protein____Plasmodium_vivax_India_VII
PVP01_0106100____Plasmodium_vivax_P01___ATP_synthase_subunit_C_putative___protein___length165
XP_008816820_1_Ftype_Htransporting_ATPase_subunit_C____Plasmodium_inui_San_Antonio_1
XP_004220687_1_ATP_synthase_lipidbinding_protein_mitochondrial_precursor____Plasmodium_cynomolgi_strain_B
XP_019912584_1_ATPase_subunit_9____Plasmodium_coatneyi
PKNH_0104400____Plasmodium_knowlesi_strain_H___ATP_synthase_subunit_C_putative___protein___length166
XP_002257539_1_ATPase_subunit_9_putative____Plasmodium_knowlesi_strain_H
XP_028541487_1_ATP_synthase_lipidbinding_protein_mitochondrial_precursor____Plasmodium_gonderi
VWU51251_1_ATP_synthase_subunit_C_putative____Hepatocystis_sp__ex_Piliocolobus_tephrosceles
VWU50032_1_ATP_synthase_subunit_C_putative____Hepatocystis_sp__ex_Piliocolobus_tephrosceles
XP_028530826_1_ATP_synthase_subunit_C_putative_partial____Plasmodium_gallinaceum
XP_028531403_1_ATP_synthase_subunit_C_putative____Plasmodium_relictum
SBS79845_1_ATP_synthase_lipidbinding_protein_mitochondrial_precursor_putative____Plasmodium_ovale_curtisi
SBS80324_1_ATP_synthase_lipidbinding_protein_mitochondrial_precursor_putative____Plasmodium_ovale_curtisi
SCD22321_1_ATP_synthase_subunit_C_putative____Plasmodium_ovale
SBT30374_1_ATP_synthase_lipidbinding_protein_mitochondrial_precursor_putative____Plasmodium_ovale_wallikeri
SBT75129_1_ATP_synthase_subunit_C_putative____Plasmodium_ovale
PCHAS_0803800____Plasmodium_chabaudi_chabaudi___ATP_synthase_subunit_C_putative___protein___length165
XP_016653716_1_ATP_synthase_subunit_C_putative____Plasmodium_chabaudi_chabaudi
SCM19972_1_ATP_synthase_subunit_C_putative____Plasmodium_chabaudi_adami
XP_008623727_1_Ftype_Htransporting_ATPase_subunit_C____Plasmodium_vinckei_vinckei
VEV56709_1_ATP_synthase_subunit_C_putative____Plasmodium_vinckei_vinckei
CAD2093034_1_ATP_synthase_subunit_C_putative____Plasmodium_vinckei_lentum
EUD74013_1_Ftype_Htransporting_ATPase_subunit_C____Plasmodium_vinckei_petteri
CAD2093035_1_ATP_synthase_subunit_C_putative____Plasmodium_vinckei_brucechwatti
PY17X_0806200____Plasmodium_yoelii_yoelii_17X___ATP_synthase_subunit_C_putative___protein___length165
XP_724019_2_ATP_synthase_subunit_C_putative____Plasmodium_yoelii
EAA15584_1_ATPase_subunit_9_putative____Plasmodium_yoelii_yoelii
XP_034421005_1_ATP_synthase_subunit_C_putative____Plasmodium_berghei_ANKA
PBANKA_0803500____Plasmodium_berghei_ANKA___ATP_synthase_subunit_C_putative___protein___length165
Cryptosporidium_murisRN66__10749
Cryptosporidium_andersoni30847__12646
XP_002140251_1_ATP_synthase_lipidbinding_protein____Cryptosporidium_muris_RN66
CMU_016520____Cryptosporidium_muris_RN66___ATP_synthase_lipidbinding_protein_putative___protein___length165
OII77787_1_ATP_synthase_lipidbinding_protein____Cryptosporidium_andersoni
Cryptosporidium_murisRN66__10728
Cryptosporidium_andersoni30847__12591
Selenidium_pygospionis__15860
Monocystis_agilis__4948
Siedleckia_nematoides__10387
Siedleckia_nematoides__10386
Siedleckia_nematoides__30582
Nephromyces_occidentalis__6621
Nephromyces_occidentalis__14075
Nephromyces_occidentalis__6616
Nephromyces_occidentalis__84646
Nephromyces_occidentalis__52866
Nephromyces_occidentalis__36251
Nephromyces_occidentalis__17617
Nephromyces_occidentalis__25162
Nephromyces_occidentalis__3277
Vbra_14129____Vitrella_brassicaformis_CCMP3155___ATP_synthase_subunit_9_mitochondrial_putative___protein___length168
Vitrella_brassicaformis__9169
CEM05154_1_unnamed_protein_product____Vitrella_brassicaformis_CCMP3155
Colpodella_angusta2__35517
Colpodella_angusta2__35518
Piridium__2187
Platyproteum_sp__4848
Platyproteum_sp__20285
Digyalum_oweni2__5353
Digyalum_oweni2__5335
Digyalum_oweni1__17453
Alphamonas_edax__26188
Alphamonas_edax__26186
Alphamonas_edax__26187
Cvel_17295____Chromera_velia_CCMP2878___hypothetical_protein___protein___length168
Chromera_velia__4033
Cvel_13917____Chromera_velia_CCMP2878___hypothetical_protein___protein___length167
Chromera_velia__30438
Symbiont_X__27015
ADV91200_1_mitochondrial_ATP_synthase_F0_lipid_binding_subunitlike_protein_5____Karlodinium_veneficum
ABI14388_1_ATP_synthase_F0_subunit_C____Karlodinium_veneficum
ADV91196_1_mitochondrial_ATP_synthase_F0_lipid_binding_subunitlike_protein_1____Karlodinium_veneficum
ADV91199_1_mitochondrial_ATP_synthase_F0_lipid_binding_subunitlike_protein_4_partial____Karlodinium_veneficum
ADV91198_1_mitochondrial_ATP_synthase_F0_lipid_binding_subunitlike_protein_3____Karlodinium_veneficum
ADV91197_1_mitochondrial_ATP_synthase_F0_lipid_binding_subunitlike_protein_2_partial____Karlodinium_veneficum
ABV22231_1_unknown____Karlodinium_veneficum
OLQ04530_1_ATP_synthase_F0_complex_subunit_C3_mitochondrial____Symbiodinium_microadriaticum
OLP99306_1_ATP_synthase_F0_complex_subunit_C1_mitochondrial____Symbiodinium_microadriaticum
XP_002772709_1_conserved_hypothetical_protein____Perkinsus_marinus_ATCC_50983
KAF4693476_1_hypothetical_protein_FOZ60_010893____Perkinsus_olseni
XP_002769859_1_conserved_hypothetical_protein____Perkinsus_marinus_ATCC_50983
Nephromyces_occidentalis__45741
OLP77726_1_LINE1_retrotransposable_element_ORF2_protein____Symbiodinium_microadriaticum
3.0

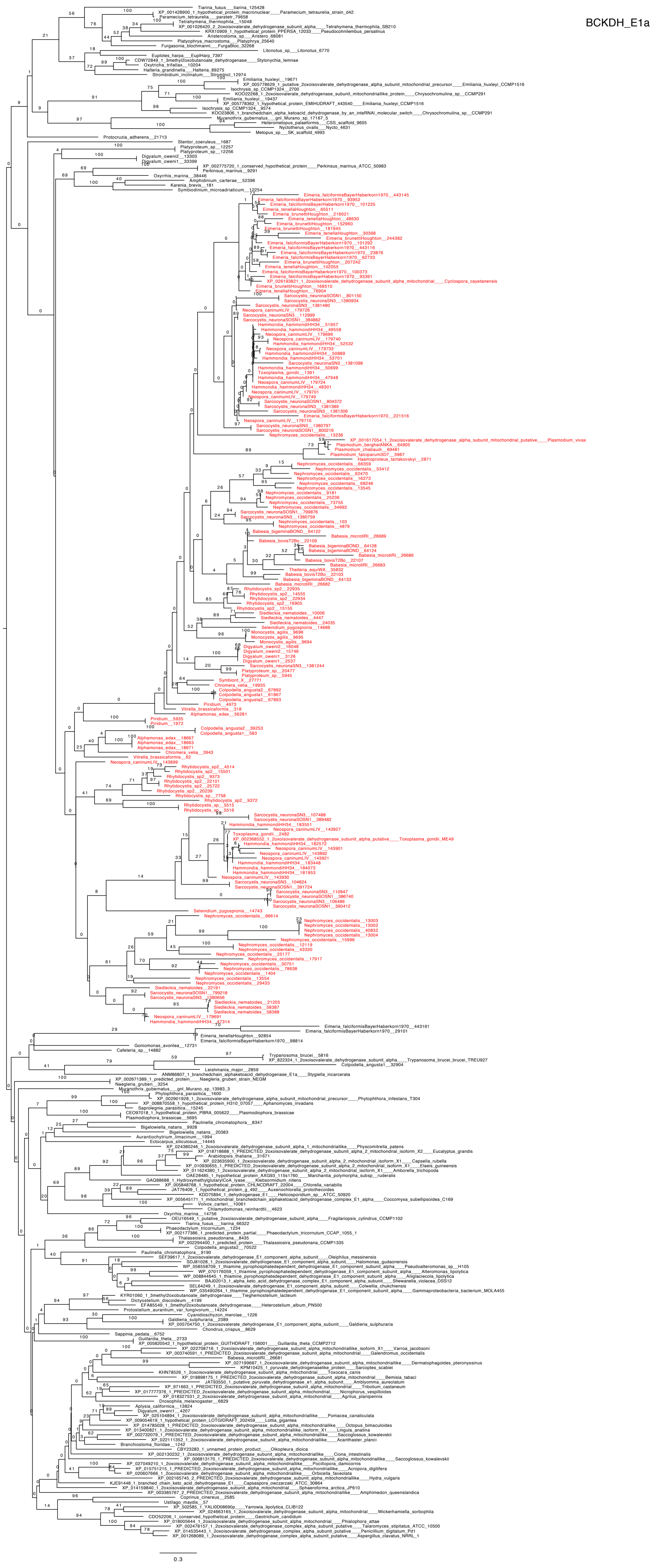
BCKDH_E1a
0.3

BCKDH_E1b

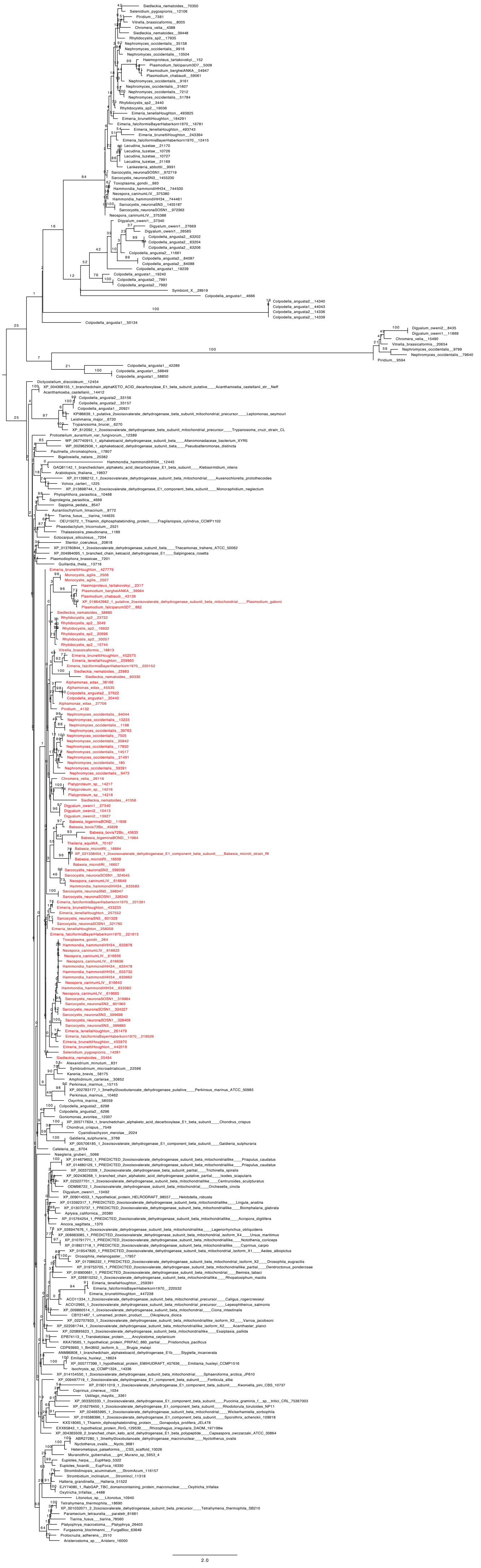

BCKDH_E2

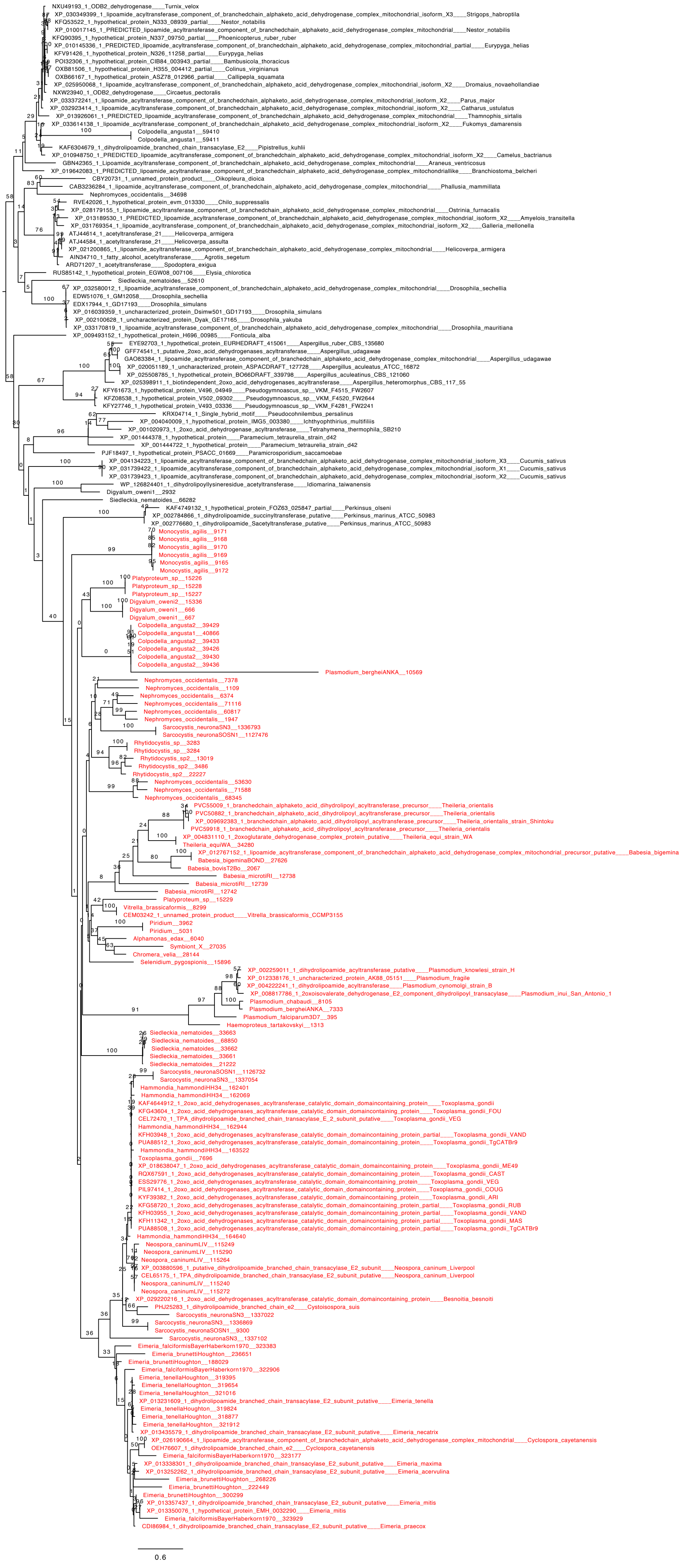
NXU49193_1_ODB2_dehydrogenase____Turnix_velox
XP_030349399_1_lipoamide_acyltransferase_component_of_branchedchain_alphaketo_acid_dehydrogenase_complex_mitochondrial_isoform_X3____Strigops_habroptila
KFQ53522_1_hypothetical_protein_N333_08939_partial____Nestor_notabilis
XP_010017145_1_PREDICTED_lipoamide_acyltransferase_component_of_branchedchain_alphaketo_acid_dehydrogenase_complex_mitochondrial____Nestor_notabilis
KFQ90395_1_hypothetical_protein_N337_09750_partial____Phoenicopterus_ruber_ruber
XP_010145336_1_PREDICTED_lipoamide_acyltransferase_component_of_branchedchain_alphaketo_acid_dehydrogenase_complex_mitochondrial_partial____Eurypyga_helias
KFV91426_1_hypothetical_protein_N326_11258_partial____Eurypyga_helias
POI32306_1_hypothetical_protein_CIB84_003943_partial____Bambusicola_thoracicus
OXB81506_1_hypothetical_protein_H355_004412_partial____Colinus_virginianus
OXB66167_1_hypothetical_protein_ASZ78_012966_partial____Callipepla_squamata
XP_025950068_1_lipoamide_acyltransferase_component_of_branchedchain_alphaketo_acid_dehydrogenase_complex_mitochondrial_isoform_X2____Dromaius_novaehollandiae
NXW23940_1_ODB2_dehydrogenase____Circaetus_pectoralis
XP_033372241_1_lipoamide_acyltransferase_component_of_branchedchain_alphaketo_acid_dehydrogenase_complex_mitochondrial_isoform_X2____Parus_major
XP_032923414_1_lipoamide_acyltransferase_component_of_branchedchain_alphaketo_acid_dehydrogenase_complex_mitochondrial_isoform_X2____Catharus_ustulatus
XP_013926061_1_PREDICTED_lipoamide_acyltransferase_component_of_branchedchain_alphaketo_acid_dehydrogenase_complex_mitochondrial____Thamnophis_sirtalis
XP_033614138_1_lipoamide_acyltransferase_component_of_branchedchain_alphaketo_acid_dehydrogenase_complex_mitochondrial_isoform_X2____Fukomys_damarensis
Colpodella_angusta1__59410
Colpodella_angusta1__59411
KAF6304679_1_dihydrolipoamide_branched_chain_transacylase_E2____Pipistrellus_kuhlii
XP_010948750_1_PREDICTED_lipoamide_acyltransferase_component_of_branchedchain_alphaketo_acid_dehydrogenase_complex_mitochondrial_isoform_X2____Camelus_bactrianus
GBN42365_1_Lipoamide_acyltransferase_component_of_branchedchain_alphaketo_acid_dehydrogenase_complex_mitochondrial____Araneus_ventricosus
XP_019642083_1_PREDICTED_lipoamide_acyltransferase_component_of_branchedchain_alphaketo_acid_dehydrogenase_complex_mitochondriallike____Branchiostoma_belcheri
CBY20731_1_unnamed_protein_product____Oikopleura_dioica
CAB3236284_1_lipoamide_acyltransferase_component_of_branchedchain_alphaketo_acid_dehydrogenase_complex_mitochondrial____Phallusia_mammillata
Nephromyces_occidentalis__34698
RVE42026_1_hypothetical_protein_evm_013330____Chilo_suppressalis
XP_028179155_1_lipoamide_acyltransferase_component_of_branchedchain_alphaketo_acid_dehydrogenase_complex_mitochondrial____Ostrinia_furnacalis
XP_013189530_1_PREDICTED_lipoamide_acyltransferase_component_of_branchedchain_alphaketo_acid_dehydrogenase_complex_mitochondrial_isoform_X2____Amyelois_transitella
XP_031769354_1_lipoamide_acyltransferase_component_of_branchedchain_alphaketo_acid_dehydrogenase_complex_mitochondrial_isoform_X2____Galleria_mellonella
ATJ44614_1_acetyltransferase_21____Helicoverpa_armigera
ATJ44584_1_acetyltransferase_21____Helicoverpa_assulta
XP_021200865_1_lipoamide_acyltransferase_component_of_branchedchain_alphaketo_acid_dehydrogenase_complex_mitochondrial____Helicoverpa_armigera
AIN34710_1_fatty_alcohol_acetyltransferase____Agrotis_segetum
ARD71207_1_acetyltransferase____Spodoptera_exigua
RUS85142_1_hypothetical_protein_EGW08_007106____Elysia_chlorotica
Siedleckia_nematoides__52610
XP_032580012_1_lipoamide_acyltransferase_component_of_branchedchain_alphaketo_acid_dehydrogenase_complex_mitochondrial____Drosophila_sechellia
EDW51076_1_GM12058____Drosophila_sechellia
EDX17944_1_GD17193____Drosophila_simulans
XP_016039359_1_uncharacterized_protein_Dsimw501_GD17193____Drosophila_simulans
XP_002100628_1_uncharacterized_protein_Dyak_GE17165____Drosophila_yakuba
XP_033170819_1_lipoamide_acyltransferase_component_of_branchedchain_alphaketo_acid_dehydrogenase_complex_mitochondrial____Drosophila_mauritiana
XP_009493152_1_hypothetical_protein_H696_00985____Fonticula_alba
EYE92703_1_hypothetical_protein_EURHEDRAFT_415061____Aspergillus_ruber_CBS_135680
GFF74541_1_putative_2oxo_acid_dehydrogenases_acyltransferase____Aspergillus_udagawae
GAO83384_1_lipoamide_acyltransferase_component_of_branchedchain_alphaketo_acid_dehydrogenase_complex_mitochondrial____Aspergillus_udagawae
XP_020051189_1_uncharacterized_protein_ASPACDRAFT_127728____Aspergillus_aculeatus_ATCC_16872
XP_025508785_1_hypothetical_protein_BO66DRAFT_339798____Aspergillus_aculeatinus_CBS_121060
XP_025398911_1_biotindependent_2oxo_acid_dehydrogenases_acyltransferase____Aspergillus_heteromorphus_CBS_117_55
KFY61673_1_hypothetical_protein_V496_04949____Pseudogymnoascus_sp__VKM_F4515_FW2607
KFZ08538_1_hypothetical_protein_V502_09302____Pseudogymnoascus_sp__VKM_F4520_FW2644
KFY27746_1_hypothetical_protein_V493_03336____Pseudogymnoascus_sp__VKM_F4281_FW2241
KRX04714_1_Single_hybrid_motif____Pseudocohnilembus_persalinus
XP_004040009_1_hypothetical_protein_IMG5_003380____Ichthyophthirius_multifiliis
XP_001020973_1_2oxo_acid_dehydrogenase_acyltransferase____Tetrahymena_thermophila_SB210
XP_001444378_1_hypothetical_protein____Paramecium_tetraurelia_strain_d42
XP_001444722_1_hypothetical_protein____Paramecium_tetraurelia_strain_d42
PJF18497_1_hypothetical_protein_PSACC_01669____Paramicrosporidium_saccamoebae
XP_004134223_1_lipoamide_acyltransferase_component_of_branchedchain_alphaketo_acid_dehydrogenase_complex_mitochondrial_isoform_X3____Cucumis_sativus
XP_031739422_1_lipoamide_acyltransferase_component_of_branchedchain_alphaketo_acid_dehydrogenase_complex_mitochondrial_isoform_X1____Cucumis_sativus
XP_031739423_1_lipoamide_acyltransferase_component_of_branchedchain_alphaketo_acid_dehydrogenase_complex_mitochondrial_isoform_X2____Cucumis_sativus
WP_126824401_1_dihydrolipoyllysineresidue_acetyltransferase____Idiomarina_taiwanensis
Digyalum_oweni1__2932
Siedleckia_nematoides__66282
KAF4749132_1_hypothetical_protein_FOZ63_025847_partial____Perkinsus_olseni
XP_002784866_1_dihydrolipoamide_succinyltransferase_putative____Perkinsus_marinus_ATCC_50983
XP_002776680_1_dihydrolipoamide_Sacetyltransferase_putative____Perkinsus_marinus_ATCC_50983
Monocystis_agilis__9171
Monocystis_agilis__9168
Monocystis_agilis__9170
Monocystis_agilis__9169
Monocystis_agilis__9165
Monocystis_agilis__9172
Platyproteum_sp__15226
Platyproteum_sp__15228
Platyproteum_sp__15227
Digyalum_oweni2__15336
Digyalum_oweni1__666
Digyalum_oweni1__667
Colpodella_angusta2__39429
Colpodella_angusta1__40866
Colpodella_angusta2__39433
Colpodella_angusta2__39426
Colpodella_angusta2__39430
Colpodella_angusta2__39436
Plasmodium_bergheiANKA__10569
Nephromyces_occidentalis__7378
Nephromyces_occidentalis__1109
Nephromyces_occidentalis__6374
Nephromyces_occidentalis__71116
Nephromyces_occidentalis__60817
Nephromyces_occidentalis__1947
Sarcocystis_neuronaSN3__1336793
Sarcocystis_neuronaSOSN1__1127476
Rhytidocystis_sp__3283
Rhytidocystis_sp__3284
Rhytidocystis_sp2__13019
Rhytidocystis_sp2__3486
Rhytidocystis_sp2__22227
Nephromyces_occidentalis__53630
Nephromyces_occidentalis__71588
Nephromyces_occidentalis__68345
PVC55009_1_branchedchain_alphaketo_acid_dihydrolipoyl_acyltransferase_precursor____Theileria_orientalis
PVC50882_1_branchedchain_alphaketo_acid_dihydrolipoyl_acyltransferase_precursor____Theileria_orientalis
XP_009692383_1_branchedchain_alphaketo_acid_dihydrolipoyl_acyltransferase_precursor____Theileria_orientalis_strain_Shintoku
PVC59918_1_branchedchain_alphaketo_acid_dihydrolipoyl_acyltransferase_precursor____Theileria_orientalis
XP_004831110_1_2oxoglutarate_dehydrogenase_complex_protein_putative____Theileria_equi_strain_WA
Theileria_equiWA__34280
XP_012767152_1_lipoamide_acyltransferase_component_of_branchedchain_alphaketo_acid_dehydrogenase_complex_mitochondrial_precursor_putative____Babesia_bigemina
Babesia_bigeminaBOND__27626
Babesia_bovisT2Bo__2067
Babesia_microtiRI__12738
Babesia_microtiRI__12739
Babesia_microtiRI__12742
Platyproteum_sp__15229
Vitrella_brassicaformis__8299
CEM03242_1_unnamed_protein_product____Vitrella_brassicaformis_CCMP3155
Piridium__3962
Piridium__5031
Alphamonas_edax__6040
Symbiont_X__27035
Chromera_velia__28144
Selenidium_pygospionis__15896
XP_002259011_1_dihydrolipoamide_acyltransferase_putative____Plasmodium_knowlesi_strain_H
XP_012338176_1_uncharacterized_protein_AK88_05151____Plasmodium_fragile
XP_004222241_1_dihydrolipoamide_acyltransferase____Plasmodium_cynomolgi_strain_B
XP_008817786_1_2oxoisovalerate_dehydrogenase_E2_component_dihydrolipoyl_transacylase____Plasmodium_inui_San_Antonio_1
Plasmodium_chabaudi__8105
Plasmodium_bergheiANKA__7333
Plasmodium_falciparum3D7__395
Haemoproteus_tartakovskyi__1313
Siedleckia_nematoides__33663
Siedleckia_nematoides__68850
Siedleckia_nematoides__33662
Siedleckia_nematoides__33661
Siedleckia_nematoides__21222
Sarcocystis_neuronaSOSN1__1126732
Sarcocystis_neuronaSN3__1337054
Hammondia_hammondiHH34__162401
Hammondia_hammondiHH34__162069
KAF4644912_1_2oxo_acid_dehydrogenases_acyltransferase_catalytic_domain_domaincontaining_protein____Toxoplasma_gondii
KFG43604_1_2oxo_acid_dehydrogenases_acyltransferase_catalytic_domain_domaincontaining_protein____Toxoplasma_gondii_FOU
CEL72470_1_TPA_dihydrolipoamide_branched_chain_transacylase_E_2_subunit_putative____Toxoplasma_gondii_VEG
Hammondia_hammondiHH34__162944
KFH03948_1_2oxo_acid_dehydrogenases_acyltransferase_catalytic_domain_domaincontaining_protein_partial____Toxoplasma_gondii_VAND
PUA88512_1_2oxo_acid_dehydrogenases_acyltransferase_catalytic_domain_domaincontaining_protein____Toxoplasma_gondii_TgCATBr9
Hammondia_hammondiHH34__163522
Toxoplasma_gondii__7696
XP_018636047_1_2oxo_acid_dehydrogenases_acyltransferase_catalytic_domain_domaincontaining_protein____Toxoplasma_gondii_ME49
RQX67591_1_2oxo_acid_dehydrogenases_acyltransferase_catalytic_domain_domaincontaining_protein____Toxoplasma_gondii_CAST
ESS29776_1_2oxo_acid_dehydrogenases_acyltransferase_catalytic_domain_domaincontaining_protein____Toxoplasma_gondii_VEG
PIL97414_1_2oxo_acid_dehydrogenases_acyltransferase_catalytic_domain_domaincontaining_protein____Toxoplasma_gondii_COUG
KYF39382_1_2oxo_acid_dehydrogenases_acyltransferase_catalytic_domain_domaincontaining_protein____Toxoplasma_gondii_ARI
KFG58720_1_2oxo_acid_dehydrogenases_acyltransferase_catalytic_domain_domaincontaining_protein_partial____Toxoplasma_gondii_RUB
KFH03955_1_2oxo_acid_dehydrogenases_acyltransferase_catalytic_domain_domaincontaining_protein_partial____Toxoplasma_gondii_VAND
KFH11342_1_2oxo_acid_dehydrogenases_acyltransferase_catalytic_domain_domaincontaining_protein_partial____Toxoplasma_gondii_MAS
PUA88508_1_2oxo_acid_dehydrogenases_acyltransferase_catalytic_domain_domaincontaining_protein_partial____Toxoplasma_gondii_TgCATBr9
Hammondia_hammondiHH34__164640
Neospora_caninumLIV__115249
Neospora_caninumLIV__115290
Neospora_caninumLIV__115264
XP_003880596_1_putative_dihydrolipoamide_branched_chain_transacylase_E2_subunit____Neospora_caninum_Liverpool
CEL65175_1_TPA_dihydrolipoamide_branched_chain_transacylase_E2_subunit_putative____Neospora_caninum_Liverpool
Neospora_caninumLIV__115240
Neospora_caninumLIV__115272
XP_029220216_1_2oxo_acid_dehydrogenases_acyltransferase_catalytic_domain_domaincontaining_protein____Besnoitia_besnoiti
PHJ25283_1_dihydrolipoamide_branched_chain_e2____Cystoisospora_suis
Sarcocystis_neuronaSN3__1337022
Sarcocystis_neuronaSN3__1336869
Sarcocystis_neuronaSOSN1__9300
Sarcocystis_neuronaSN3__1337102
Eimeria_falciformisBayerHaberkorn1970__323383
Eimeria_brunettiHoughton__236651
Eimeria_brunettiHoughton__188029
Eimeria_falciformisBayerHaberkorn1970__322906
Eimeria_tenellaHoughton__319395
Eimeria_tenellaHoughton__319654
Eimeria_tenellaHoughton__321016
XP_013231609_1_dihydrolipoamide_branched_chain_transacylase_E2_subunit_putative____Eimeria_tenella
Eimeria_tenellaHoughton__319824
Eimeria_tenellaHoughton__318877
Eimeria_tenellaHoughton__321912
XP_013435579_1_dihydrolipoamide_branched_chain_transacylase_E2_subunit_putative____Eimeria_necatrix
XP_026190664_1_lipoamide_acyltransferase_component_of_branchedchain_alphaketo_acid_dehydrogenase_complex_mitochondrial____Cyclospora_cayetanensis
OEH76607_1_dihydrolipoamide_branched_chain_e2____Cyclospora_cayetanensis
Eimeria_falciformisBayerHaberkorn1970__323177
XP_013338301_1_dihydrolipoamide_branched_chain_transacylase_E2_subunit_putative____Eimeria_maxima
XP_013252262_1_dihydrolipoamide_branched_chain_transacylase_E2_subunit_putative____Eimeria_acervulina
Eimeria_brunettiHoughton__268226
Eimeria_brunettiHoughton__222449
Eimeria_brunettiHoughton__300299
XP_013357437_1_dihydrolipoamide_branched_chain_transacylase_E2_subunit_putative____Eimeria_mitis
XP_013350076_1_hypothetical_protein_EMH_0032290____Eimeria_mitis
Eimeria_falciformisBayerHaberkorn1970__323929
CDI86984_1_dihydrolipoamide_branched_chain_transacylase_E2_subunit_putative____Eimeria_praecox
0.6

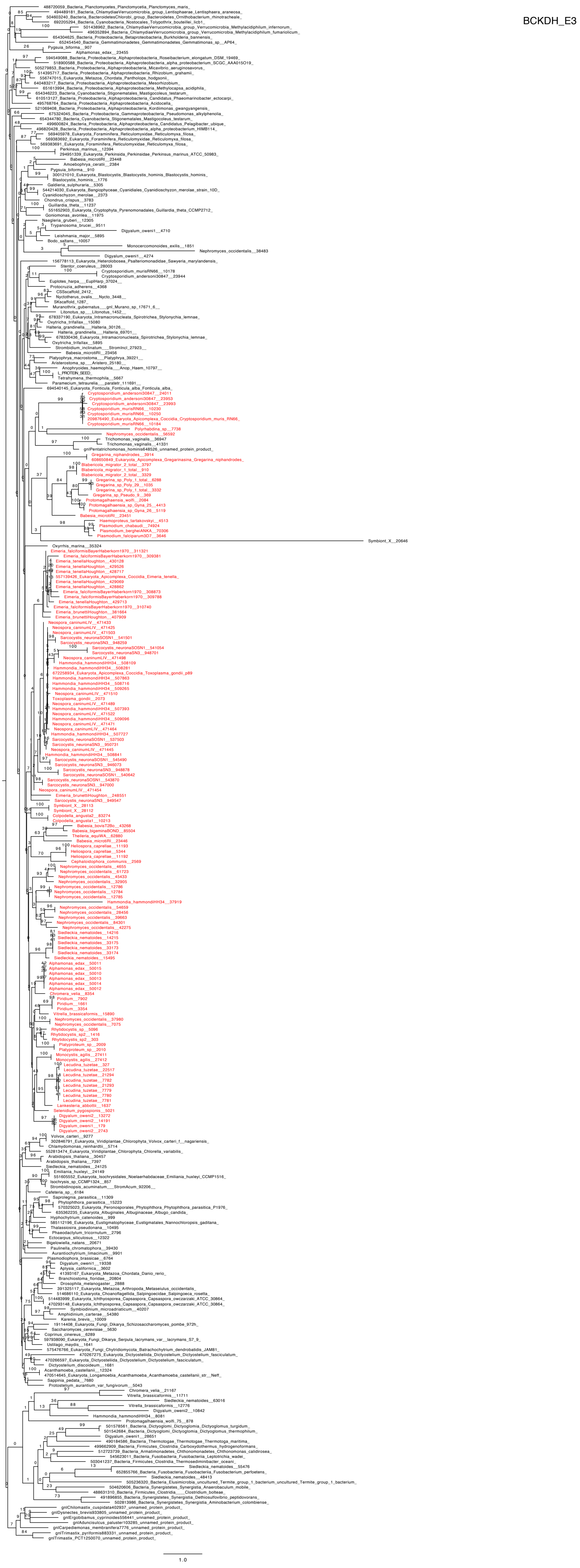
BCKDH_E3
1.0

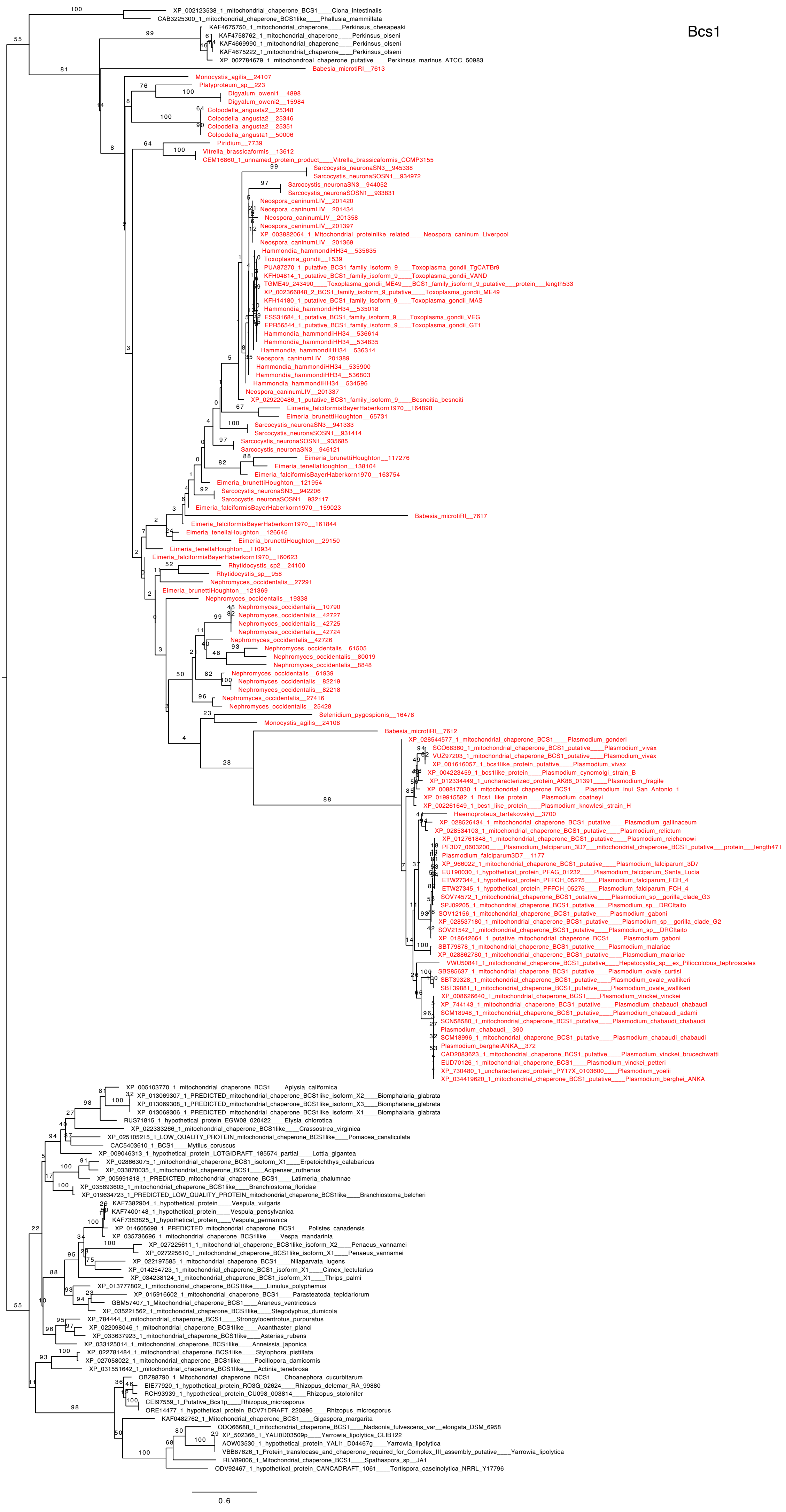

Bcs1
XP_002123538_1_mitochondrial_chaperone_BCS1____Ciona_intestinalis
CAB3225800_1_mitochondrial_chaperone_BCS1like____Phallusia_mammillata
KAF4675750_1_mitochondrial_chaperone____Perkinsus_chesapeaki
KAF4758762_1_mitochondrial_chaperone____Perkinsus_olseni
KAF4669990_1_mitochondrial_chaperone____Perkinsus_olseni
KAF4675222_1_mitochondrial_chaperone____Perkinsus_olseni
XP_002784679_1_mitochondroal_chaperone_putative____Perkinsus_marinus_ATCC_50983
Babesia_microtiRI__7613
Monocystis_agilis__24107
Platyproteum_sp__223
Digyalum_oweni1__4898
Digyalum_oweni2__15984
Colpodella_angusta2__25348
Colpodella_angusta2__25346
Colpodella_angusta2__25351
Colpodella_angusta1__50006
Piridium__7739
Vitrella_brassicaformis__13612
CEM16860_1_unnamed_protein_product____Vitrella_brassicaformis_CCMP3155
Sarcocystis_neuronaSN3__945338
Sarcocystis_neuronaSOSN1__934972
Sarcocystis_neuronaSN3__944052
Sarcocystis_neuronaSOSN1__933831
Neospora_caninumLIV__201420
Neospora_caninumLIV__201434
Neospora_caninumLIV__201358
Neospora_caninumLIV__201397
XP_003882064_1_Mitochondrial_proteinlike_related____Neospora_caninum_Liverpool
Neospora_caninumLIV__201369
Hammondia_hammondiHH34__535635
Toxoplasma_gondii__1539
PUA87270_1_putative_BCS1_family_isoform_9____Toxoplasma_gondii_TgCATBr9
KFH04814_1_putative_BCS1_family_isoform_9____Toxoplasma_gondii_VAND
TGME49_243490____Toxoplasma_gondii_ME49____BCS1_family_isoform_9_putative___protein___length533
XP_002366848_2_BCS1_family_isoform_9_putative____Toxoplasma_gondii_ME49
KFH14180_1_putative_BCS1_family_isoform_9____Toxoplasma_gondii_MAS
Hammondia_hammondiHH34__535018
ESS31684_1_putative_BCS1_family_isoform_9____Toxoplasma_gondii_VEG
EPR56544_1_putative_BCS1_family_isoform_9____Toxoplasma_gondii_GT1
Hammondia_hammondiHH34__536614
Hammondia_hammondiHH34__534835
Hammondia_hammondiHH34__536314
Neospora_caninumLIV__201389
Hammondia_hammondiHH34__535900
Hammondia_hammondiHH34__536803
Hammondia_hammondiHH34__534596
Neospora_caninumLIV__201337
XP_029220486_1_putative_BCS1_family_isoform_9____Besnoitia_besnoiti
Eimeria_falciformisBayerHaberkorn1970__164898
Eimeria_brunettiHoughton__65731
Sarcocystis_neuronaSN3__941333
Sarcocystis_neuronaSOSN1__931414
Sarcocystis_neuronaSOSN1__935685
Sarcocystis_neuronaSN3__946121
Eimeria_brunettiHoughton__117276
Eimeria_tenellaHoughton__138104
Eimeria_falciformisBayerHaberkorn1970__163754
Eimeria_brunettiHoughton__121954
Sarcocystis_neuronaSN3__942206
Sarcocystis_neuronaSOSN1__932117
Eimeria_falciformisBayerHaberkorn1970__159023
Babesia_microtiRI__7617
Eimeria_falciformisBayerHaberkorn1970__161844
Eimeria_tenellaHoughton__126646
Eimeria_brunettiHoughton__29150
Eimeria_tenellaHoughton__110934
Eimeria_falciformisBayerHaberkorn1970__160623
Rhytidocystis_sp2__24100
Rhytidocystis_sp__958
Nephromyces_occidentalis__27291
Eimeria_brunettiHoughton__121369
Nephromyces_occidentalis__19338
Nephromyces_occidentalis__10790
Nephromyces_occidentalis__42727
Nephromyces_occidentalis__42725
Nephromyces_occidentalis__42724
Nephromyces_occidentalis__42726
Nephromyces_occidentalis__61505
Nephromyces_occidentalis__80019
Nephromyces_occidentalis__8848
Nephromyces_occidentalis__61939
Nephromyces_occidentalis__82219
Nephromyces_occidentalis__82218
Nephromyces_occidentalis__27416
Nephromyces_occidentalis__25428
Selenidium_pygospionis__16478
Monocystis_agilis__24108
Babesia_microtiRI__7612
XP_028544577_1_mitochondrial_chaperone_BCS1____Plasmodium_gonderi
SCO68360_1_mitochondrial_chaperone_BCS1_putative____Plasmodium_vivax
VUZ97203_1_mitochondrial_chaperone_BCS1_putative____Plasmodium_vivax
XP_001616057_1_bcs1like_protein_putative____Plasmodium_vivax
XP_004223459_1_bcs1like_protein____Plasmodium_cynomolgi_strain_B
XP_012334449_1_uncharacterized_protein_AK88_01391____Plasmodium_fragile
XP_008817030_1_mitochondrial_chaperone_BCS1____Plasmodium_inui_San_Antonio_1
XP_019915582_1_Bcs1_like_protein____Plasmodium_coatneyi
XP_002261649_1_bcs1_like_protein____Plasmodium_knowlesi_strain_H
Haemoproteus_tartakovskyi__3700
XP_028526434_1_mitochondrial_chaperone_BCS1_putative____Plasmodium_gallinaceum
XP_028534103_1_mitochondrial_chaperone_BCS1_putative____Plasmodium_relictum
XP_012761848_1_mitochondrial_chaperone_BCS1_putative____Plasmodium_reichenowi
PF3D7_0603200____Plasmodium_falciparum_3D7____mitochondrial_chaperone_BCS1_putative___protein___length471
Plasmodium_falciparum3D7__1177
XP_966022_1_mitochondrial_chaperone_BCS1_putative____Plasmodium_falciparum_3D7
EUT90030_1_hypothetical_protein_PFAG_01232____Plasmodium_falciparum_Santa_Lucia
ETW27344_1_hypothetical_protein_PFFCH_05275____Plasmodium_falciparum_FCH_4
ETW27345_1_hypothetical_protein_PFFCH_05276____Plasmodium_falciparum_FCH_4
SOV74572_1_mitochondrial_chaperone_BCS1_putative____Plasmodium_sp__gorilla_clade_G3
SPJ09205_1_mitochondrial_chaperone_BCS1_putative____Plasmodium_sp__DRCItaito
SOV12156_1_mitochondrial_chaperone_BCS1_putative____Plasmodium_gaboni
XP_028537180_1_mitochondrial_chaperone_BCS1_putative____Plasmodium_sp__gorilla_clade_G2
SOV21542_1_mitochondrial_chaperone_BCS1_putative____Plasmodium_sp__DRCItaito
XP_018642664_1_putative_mitochondrial_chaperone_BCS1____Plasmodium_gaboni
SBT79878_1_mitochondrial_chaperone_BCS1_putative____Plasmodium_malariae
XP_028862780_1_mitochondrial_chaperone_BCS1_putative____Plasmodium_malariae
VWU50841_1_mitochondrial_chaperone_BCS1_putative____Hepatocystis_sp__ex_Piliocolobus_tephrosceles
SBS85637_1_mitochondrial_chaperone_BCS1_putative____Plasmodium_ovale_curtisi
SBT39328_1_mitochondrial_chaperone_BCS1_putative____Plasmodium_ovale_wallikeri
SBT39881_1_mitochondrial_chaperone_BCS1_putative____Plasmodium_ovale_wallikeri
XP_008626640_1_mitochondrial_chaperone_BCS1____Plasmodium_vinckei_vinckei
XP_744143_1_mitochondrial_chaperone_BCS1_putative____Plasmodium_chabaudi_chabaudi
SCM18948_1_mitochondrial_chaperone_BCS1_putative____Plasmodium_chabaudi_adami
SCN58580_1_mitochondrial_chaperone_BCS1_putative____Plasmodium_chabaudi_chabaudi
Plasmodium_chabaudi__390
SCM18996_1_mitochondrial_chaperone_BCS1_putative____Plasmodium_chabaudi_chabaudi
Plasmodium_bergheiANKA__372
CAD2083623_1_mitochondrial_chaperone_BCS1_putative____Plasmodium_vinckei_brucechwatti
EUD70126_1_mitochondrial_chaperone_BCS1____Plasmodium_vinckei_petteri
XP_730480_1_uncharacterized_protein_PY17X_0103600____Plasmodium_yoelii
XP_034419620_1_mitochondrial_chaperone_BCS1_putative____Plasmodium_berghei_ANKA
XP_005103770_1_mitochondrial_chaperone_BCS1____Aplysia_californica
XP_013069307_1_PREDICTED_mitochondrial_chaperone_BCS1like_isoform_X2____Biomphalaria_glabrata
XP_013069308_1_PREDICTED_mitochondrial_chaperone_BCS1like_isoform_X3____Biomphalaria_glabrata
XP_013069306_1_PREDICTED_mitochondrial_chaperone_BCS1like_isoform_X1____Biomphalaria_glabrata
RUS71815_1_hypothetical_protein_EGW08_020422____Elysia_chlorotica
XP_022333266_1_mitochondrial_chaperone_BCS1like____Crassostrea_virginica
XP_025105215_1_LOW_QUALITY_PROTEIN_mitochondrial_chaperone_BCS1like____Pomacea_canaliculata
CAC5403610_1_BCS1____Mytilus_coruscus
XP_009046313_1_hypothetical_protein_LOTGIDRAFT_185574_partial____Lottia_gigantea
XP_028663075_1_mitochondrial_chaperone_BCS1_isoform_X1____Erpetoichthys_calabaricus
XP_033870035_1_mitochondrial_chaperone_BCS1____Acipenser_ruthenus
XP_005991818_1_PREDICTED_mitochondrial_chaperone_BCS1____Latimeria_chalumnae
XP_035693603_1_mitochondrial_chaperone_BCS1like____Branchiostoma_floridae
XP_019634723_1_PREDICTED_LOW_QUALITY_PROTEIN_mitochondrial_chaperone_BCS1like____Branchiostoma_belcheri
KAF7382904_1_hypothetical_protein____Vespula_vulgaris
KAF7400148_1_hypothetical_protein____Vespula_pensylvanica
KAF7383825_1_hypothetical_protein____Vespula_germanica
XP_014605698_1_PREDICTED_mitochondrial_chaperone_BCS1____Polistes_canadensis
XP_035736696_1_mitochondrial_chaperone_BCS1like____Vespa_mandarinia
XP_027225611_1_mitochondrial_chaperone_BCS1like_isoform_X2____Penaeus_vannamei
XP_027225610_1_mitochondrial_chaperone_BCS1like_isoform_X1____Penaeus_vannamei
XP_022197585_1_mitochondrial_chaperone_BCS1____Nilaparvata_lugens
XP_014254723_1_mitochondrial_chaperone_BCS1_isoform_X1____Cimex_lectularius
XP_034238124_1_mitochondrial_chaperone_BCS1_isoform_X1____Thrips_palmi
XP_013777802_1_mitochondrial_chaperone_BCS1like____Limulus_polyphemus
XP_015916602_1_mitochondrial_chaperone_BCS1____Parasteatoda_tepidariorum
GBM57407_1_Mitochondrial_chaperone_BCS1____Araneus_ventricosus
XP_035221562_1_mitochondrial_chaperone_BCS1like____Stegodyphus_dumicola
XP_784444_1_mitochondrial_chaperone_BCS1____Strongylocentrotus_purpuratus
XP_022098046_1_mitochondrial_chaperone_BCS1like____Acanthaster_planci
XP_033637923_1_mitochondrial_chaperone_BCS1like____Asterias_rubens
XP_033125014_1_mitochondrial_chaperone_BCS1like____Anneissia_japonica
XP_022781484_1_mitochondrial_chaperone_BCS1like____Stylophora_pistillata
XP_027058022_1_mitochondrial_chaperone_BCS1like____Pocillopora_damicornis
XP_031551642_1_mitochondrial_chaperone_BCS1like____Actinia_tenebrosa
OBZ88790_1_Mitochondrial_chaperone_BCS1____Choanephora_cucurbitarum
EIE77920_1_hypothetical_protein_RO3G_02624____Rhizopus_delemar_RA_99880
RCH93939_1_hypothetical_protein_CU098_003814____Rhizopus_stolonifer
CEI97559_1_Putative_Bcs1p____Rhizopus_microsporus
ORE14477_1_hypothetical_protein_BCV71DRAFT_220896____Rhizopus_microsporus
KAF0482762_1_Mitochondrial_chaperone_BCS1____Gigaspora_margarita
ODQ66688_1_mitochondrial_chaperone_BCS1____Nadsonia_fulvescens_var__elongata_DSM_6958
XP_502366_1_YALI0D03509p____Yarrowia_lipolytica_CLIB122
AOW03530_1_hypothetical_protein_YALI1_D04467g____Yarrowia_lipolytica
VBB87626_1_Protein_translocase_and_chaperone_required_for_Complex_III_assembly_putative____Yarrowia_lipolytica
RLV89006_1_Mitochondrial_chaperone_BCS1____Spathaspora_sp__JA1
ODV92467_1_hypothetical_protein_CANCADRAFT_1061____Tortispora_caseinolytica_NRRL_Y17796
0.6

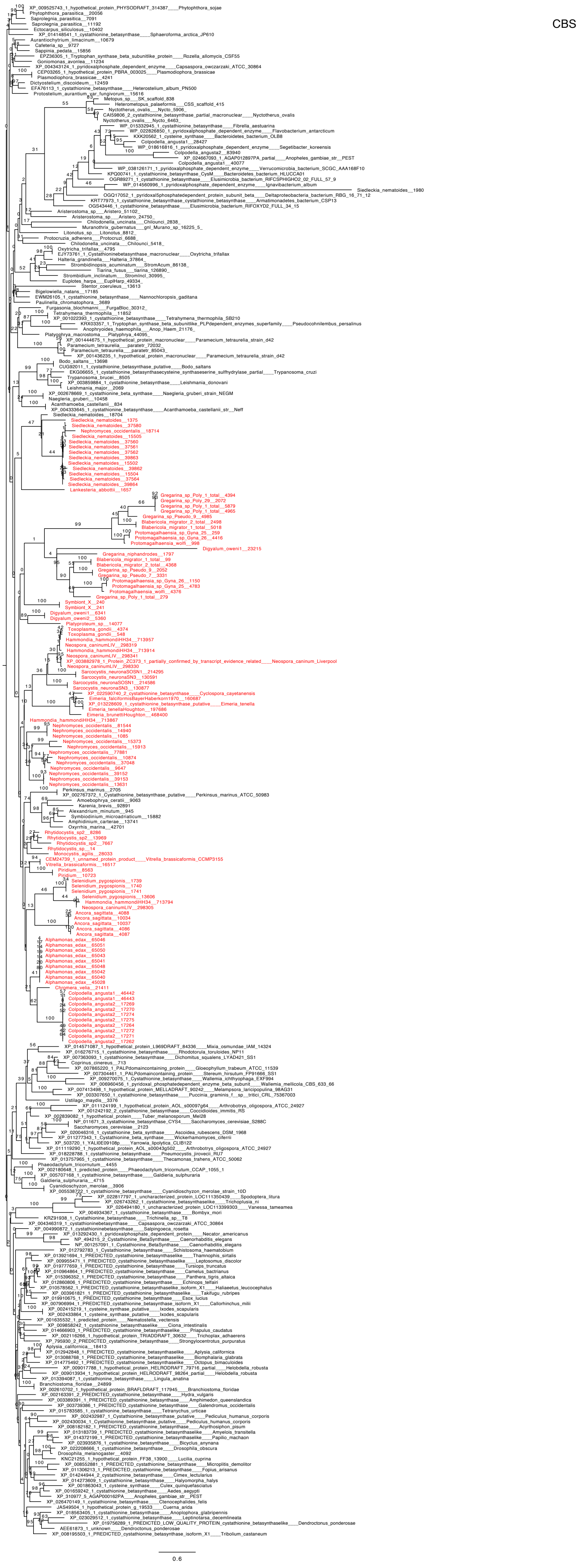
CBS
0.6

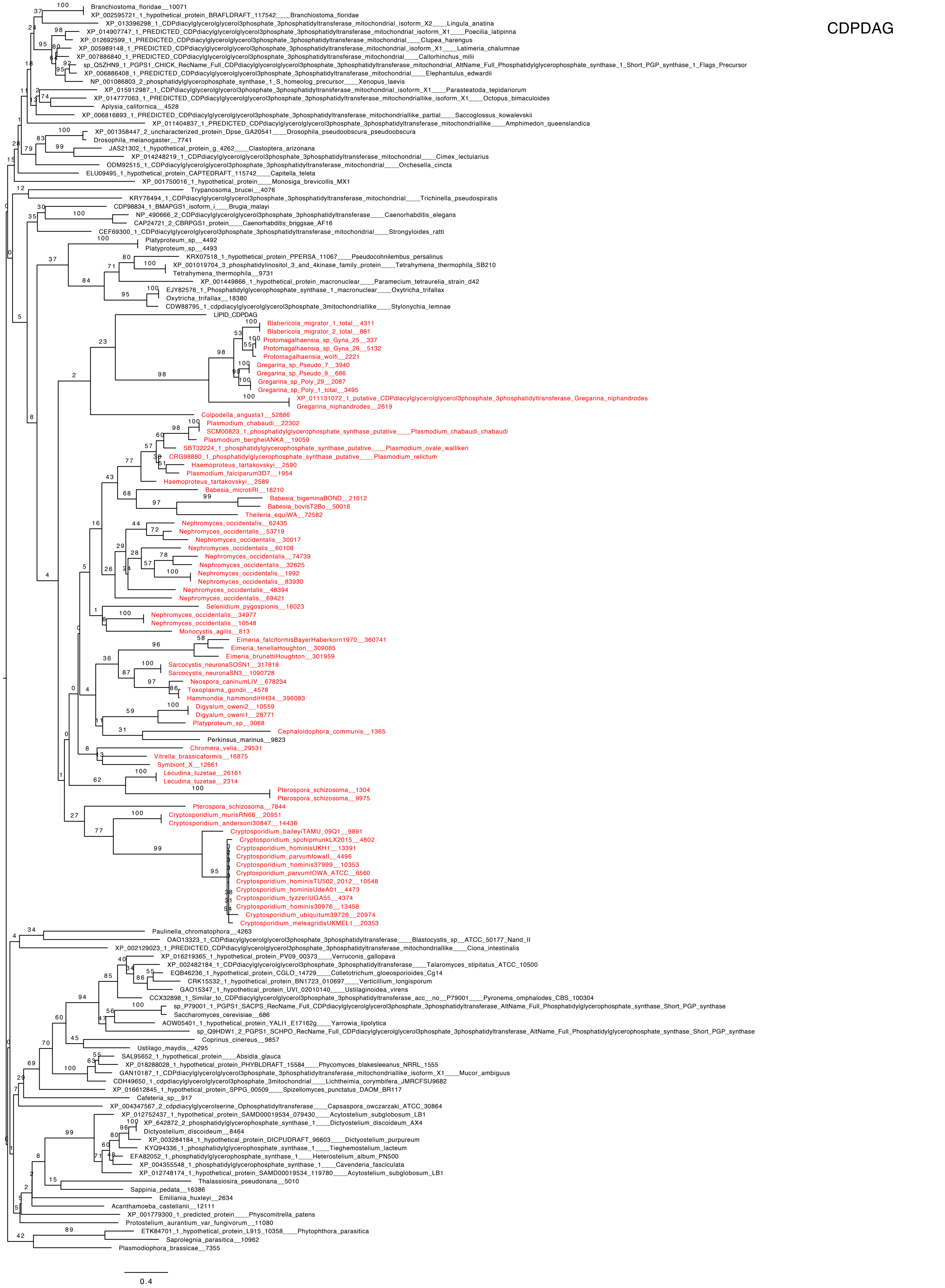
CDPDAG
Branchiostoma_floridae__10071
XP_002595721_1_hypothetical_protein_BRAFLDRAFT_117542____Branchiostoma_floridae
XP_013396298_1_CDPdiacylglycerolglycerol3phosphate_3phosphatidyltransferase_mitochondrial_isoform_X2____Lingula_anatina
XP_014907747_1_PREDICTED_CDPdiacylglycerolglycerol3phosphate_3phosphatidyltransferase_mitochondrial_isoform_X1____Poecilia_latipinna
XP_012692599_1_PREDICTED_CDPdiacylglycerolglycerol3phosphate_3phosphatidyltransferase_mitochondrial____Clupea_harengus
XP_005989148_1_PREDICTED_CDPdiacylglycerolglycerol3phosphate_3phosphatidyltransferase_mitochondrial_isoform_X1____Latimeria_chalumnae
XP_007886840_1_PREDICTED_CDPdiacylglycerolglycerol3phosphate_3phosphatidyltransferase_mitochondrial____Callorhinchus_milii
sp_Q5ZHN9_1_PGPS1_CHICK_RecName_Full_CDPdiacylglycerolglycerol3phosphate_3phosphatidyltransferase_mitochondrial_AltName_Full_Phosphatidylglycerophosphate_synthase_1_Short_PGP_synthase_1_Flags_Precursor
XP_006886408_1_PREDICTED_CDPdiacylglycerolglycerol3phosphate_3phosphatidyltransferase_mitochondrial____Elephantulus_edwardii
NP_001086803_2_phosphatidylglycerophosphate_synthase_1_S_homeolog_precursor____Xenopus_laevis
XP_015912987_1_CDPdiacylglycerolglycerol3phosphate_3phosphatidyltransferase_mitochondrial_isoform_X1____Parasteatoda_tepidariorum
XP_014777063_1_PREDICTED_CDPdiacylglycerolglycerol3phosphate_3phosphatidyltransferase_mitochondriallike_isoform_X1____Octopus_bimaculoides
Aplysia_californica__4528
XP_006816893_1_PREDICTED_CDPdiacylglycerolglycerol3phosphate_3phosphatidyltransferase_mitochondriallike_partial____Saccoglossus_kowalevskii
XP_011404837_1_PREDICTED_CDPdiacylglycerolglycerol3phosphate_3phosphatidyltransferase_mitochondriallike____Amphimedon_queenslandica
XP_001358447_2_uncharacterized_protein_Dpse_GA20541____Drosophila_pseudoobscura_pseudoobscura
Drosophila_melanogaster__7741
JAS21302_1_hypothetical_protein_g_4262____Clastoptera_arizonana
XP_014248219_1_CDPdiacylglycerolglycerol3phosphate_3phosphatidyltransferase_mitochondrial____Cimex_lectularius
ODM92515_1_CDPdiacylglycerolglycerol3phosphate_3phosphatidyltransferase_mitochondrial____Orchesella_cincta
ELU09495_1_hypothetical_protein_CAPTEDRAFT_115742____Capitella_teleta
XP_001750016_1_hypothetical_protein____Monosiga_brevicollis_MX1
Trypanosoma_brucei__4076
KRY76494_1_CDPdiacylglycerolglycerol3phosphate_3phosphatidyltransferase_mitochondrial____Trichinella_pseudospiralis
CDP98834_1_BMAPGS1_isoform_i____Brugia_malayi
NP_490666_2_CDPdiacylglycerolglycerol3phosphate_3phosphatidyltransferase____Caenorhabditis_elegans
CAP24721_2_CBRPGS1_protein____Caenorhabditis_briggsae_AF16
CEF69300_1_CDPdiacylglycerolglycerol3phosphate_3phosphatidyltransferase_mitochondrial____Strongyloides_ratti
Platyproteum_sp__4492
Platyproteum_sp__4493
KRX07518_1_hypothetical_protein_PPERSA_11067____Pseudocohnilembus_persalinus
XP_001019704_3_phosphatidylinositol_3_and_4kinase_family_protein____Tetrahymena_thermophila_SB210
Tetrahymena_thermophila__9731
XP_001449866_1_hypothetical_protein_macronuclear____Paramecium_tetraurelia_strain_d42
EJY82576_1_Phosphatidylglycerophosphate_synthase_1_macronuclear____Oxytricha_trifallax
Oxytricha_trifallax__18380
CDW88795_1_cdpdiacylglycerolglycerol3phosphate_3mitochondriallike____Stylonychia_lemnae
LIPID_CDPDAG
Blabericola_migrator_1_total__4311
Blabericola_migrator_2_total__881
Protomagalhaensia_sp_Gyna_25__337
Protomagalhaensia_sp_Gyna_26__5132
Protomagalhaensia_wolfi__2221
Gregarina_sp_Pseudo_7__3940
Gregarina_sp_Pseudo_9__686
Gregarina_sp_Poly_29__2087
Gregarina_sp_Poly_1_total__3495
XP_011131072_1_putative_CDPdiacylglycerolglycerol3phosphate_3phosphatidyltransferase_Gregarina_niphandrodes
Gregarina_niphandrodes__2619
Colpodella_angusta1__52886
Plasmodium_chabaudi__22302
SCM00823_1_phosphatidylglycerophosphate_synthase_putative____Plasmodium_chabaudi_chabaudi
Plasmodium_bergheiANKA__19059
SBT32224_1_phosphatidylglycerophosphate_synthase_putative____Plasmodium_ovale_wallikeri
CRG98880_1_phosphatidylglycerophosphate_synthase_putative____Plasmodium_relictum
Haemoproteus_tartakovskyi__2590
Plasmodium_falciparum3D7__1954
Haemoproteus_tartakovskyi__2589
Babesia_microtiRI__18210
Babesia_bigeminaBOND__21612
Babesia_bovisT2Bo__50016
Theileria_equiWA__72582
Nephromyces_occidentalis__62435
Nephromyces_occidentalis__53719
Nephromyces_occidentalis__30017
Nephromyces_occidentalis__60108
Nephromyces_occidentalis__74739
Nephromyces_occidentalis__32625
Nephromyces_occidentalis__1992
Nephromyces_occidentalis__83930
Nephromyces_occidentalis__48394
Nephromyces_occidentalis__69421
Selenidium_pygospionis__16023
Nephromyces_occidentalis__34977
Nephromyces_occidentalis__10548
Monocystis_agilis__813
Eimeria_falciformisBayerHaberkorn1970__360741
Eimeria_tenellaHoughton__309085
Eimeria_brunettiHoughton__301959
Sarcocystis_neuronaSOSN1__317818
Sarcocystis_neuronaSN3__1090728
Neospora_caninumLIV__678234
Toxoplasma_gondii__4578
Hammondia_hammondiHH34__396083
Digyalum_oweni2__10559
Digyalum_oweni1__28771
Platyproteum_sp__3068
Cephaloidophora_communis__1365
Perkinsus_marinus__9823
Chromera_velia__29531
Vitrella_brassicaformis__16875
Symbiont_X__12661
Lecudina_tuzetae__26161
Lecudina_tuzetae__2314
Pterospora_schizosoma__1304
Pterospora_schizosoma__9975
Pterospora_schizosoma__7844
Cryptosporidium_murisRN66__20951
Cryptosporidium_andersoni30847__14436
Cryptosporidium_baileyiTAMU_09Q1__9891
Cryptosporidium_spchipmunkLX2015__4802
Cryptosporidium_hominisUKH1__13391
Cryptosporidium_parvumIowaII__4496
Cryptosporidium_hominis37999__10353
Cryptosporidium_parvumIOWA_ATCC__6560
Cryptosporidium_hominisTU502_2012__10548
Cryptosporidium_hominisUdeA01__4473
Cryptosporidium_tyzzeriUGA55__4374
Cryptosporidium_hominis30976__13458
Cryptosporidium_ubiquitum39726__20974
Cryptosporidium_meleagridisUKMEL1__20353
Paulinella_chromatophora__4263
OAO13323_1_CDPdiacylglycerolglycerol3phosphate_3phosphatidyltransferase____Blastocystis_sp__ATCC_50177_Nand_II
XP_002129023_1_PREDICTED_CDPdiacylglycerolglycerol3phosphate_3phosphatidyltransferase_mitochondriallike____Ciona_intestinalis
XP_016219365_1_hypothetical_protein_PV09_00373____Verruconis_gallopava
XP_002482184_1_CDPdiacylglycerolglycerol3phosphate_3phosphatidyltransferase____Talaromyces_stipitatus_ATCC_10500
EQB46236_1_hypothetical_protein_CGLO_14729____Colletotrichum_gloeosporioides_Cg14
CRK15532_1_hypothetical_protein_BN1723_010697____Verticillium_longisporum
GAO15347_1_hypothetical_protein_UVI_02010140____Ustilaginoidea_virens
CCX32898_1_Similar_to_CDPdiacylglycerolglycerol3phosphate_3phosphatidyltransferase_acc__no__P79001____Pyronema_omphalodes_CBS_100304
sp_P79001_1_PGPS1_SACPS_RecName_Full_CDPdiacylglycerolglycerol3phosphate_3phosphatidyltransferase_AltName_Full_Phosphatidylglycerophosphate_synthase_Short_PGP_synthase
Saccharomyces_cerevisiae__686
AOW05401_1_hypothetical_protein_YALI1_E17162g____Yarrowia_lipolytica
sp_Q9HDW1_2_PGPS1_SCHPO_RecName_Full_CDPdiacylglycerolglycerol3phosphate_3phosphatidyltransferase_AltName_Full_Phosphatidylglycerophosphate_synthase_Short_PGP_synthase
Coprinus_cinereus__9857
Ustilago_maydis__4295
SAL95652_1_hypothetical_protein____Absidia_glauca
XP_018288028_1_hypothetical_protein_PHYBLDRAFT_15584____Phycomyces_blakesleeanus_NRRL_1555
GAN10187_1_CDPdiacylglycerolglycerol3phosphate_3phosphatidyltransferase_mitochondriallike_isoform_X1____Mucor_ambiguus
CDH49650_1_cdpdiacylglycerolglycerol3phosphate_3mitochondrial____Lichtheimia_corymbifera_JMRCFSU9682
XP_016612845_1_hypothetical_protein_SPPG_00509____Spizellomyces_punctatus_DAOM_BR117
Cafeteria_sp__917
XP_004347567_2_cdpdiacylglycerolserine_Ophosphatidyltransferase____Capsaspora_owczarzaki_ATCC_30864
XP_012752437_1_hypothetical_protein_SAMD00019534_079430____Acytostelium_subglobosum_LB1
XP_642872_2_phosphatidylglycerophosphate_synthase_1____Dictyostelium_discoideum_AX4
Dictyostelium_discoideum__8464
XP_003284184_1_hypothetical_protein_DICPUDRAFT_96603____Dictyostelium_purpureum
KYQ94336_1_phosphatidylglycerophosphate_synthase_1____Tieghemostelium_lacteum
EFA82052_1_phosphatidylglycerophosphate_synthase_1____Heterostelium_album_PN500
XP_004355548_1_phosphatidylglycerophosphate_synthase_1____Cavenderia_fasciculata
XP_012748174_1_hypothetical_protein_SAMD00019534_119780____Acytostelium_subglobosum_LB1
Thalassiosira_pseudonana__5010
Sappinia_pedata__16386
Emiliania_huxleyi__2634
Acanthamoeba_castellanii__12111
XP_001779300_1_predicted_protein____Physcomitrella_patens
Protostelium_aurantium_var_fungivorum__11080
ETK84701_1_hypothetical_protein_L915_10358____Phytophthora_parasitica
Saprolegnia_parasitica__10962
Plasmodiophora_brassicae__7355
0.4

COQ1

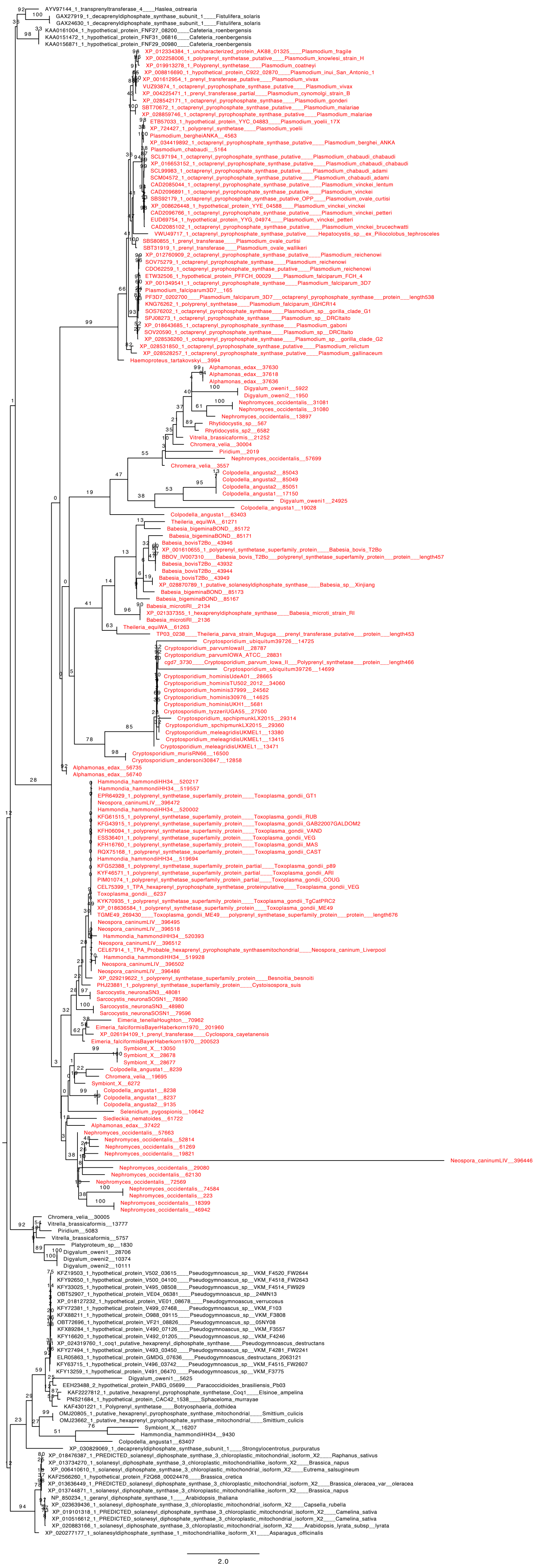

AYV97144_1_transprenyltransferase_4____Haslea_ostrearia
GAX27919_1_decaprenyldiphosphate_synthase_subunit_1____Fistulifera_solaris
GAX24630_1_decaprenyldiphosphate_synthase_subunit_1____Fistulifera_solaris
KAA0161004_1_hypothetical_protein_FNF27_08200____Cafeteria_roenbergensis
KAA0151472_1_hypothetical_protein_FNF31_06816____Cafeteria_roenbergensis
KAA0156871_1_hypothetical_protein_FNF29_00980____Cafeteria_roenbergensis
XP_012334384_1_uncharacterized_protein_AK88_01325____Plasmodium_fragile
XP_002258006_1_polyprenyl_synthetase_putative____Plasmodium_knowlesi_strain_H
XP_019913278_1_Polyprenyl_synthetase____Plasmodium_coatneyi
XP_008816690_1_hypothetical_protein_C922_02870____Plasmodium_inui_San_Antonio_1
XP_001612954_1_prenyl_transferase_putative____Plasmodium_vivax
VUZ93874_1_octaprenyl_pyrophosphate_synthase_putative____Plasmodium_vivax
XP_004225471_1_prenyl_transferase_partial____Plasmodium_cynomolgi_strain_B
XP_028542171_1_octaprenyl_pyrophosphate_synthase____Plasmodium_gonderi
SBT70672_1_octaprenyl_pyrophosphate_synthase_putative____Plasmodium_malariae
XP_028859746_1_octaprenyl_pyrophosphate_synthase_putative____Plasmodium_malariae
ETB57033_1_hypothetical_protein_YYC_04883____Plasmodium_yoelii_17X
XP_724427_1_polyprenyl_synthetase____Plasmodium_yoelii
Plasmodium_bergheiANKA__4563
XP_034419892_1_octaprenyl_pyrophosphate_synthase_putative____Plasmodium_berghei_ANKA
Plasmodium_chabaudi__5164
SCL97194_1_octaprenyl_pyrophosphate_synthase_putative____Plasmodium_chabaudi_chabaudi
XP_016653152_1_octaprenyl_pyrophosphate_synthase_putative____Plasmodium_chabaudi_chabaudi
SCL99983_1_octaprenyl_pyrophosphate_synthase_putative____Plasmodium_chabaudi_adami
SCM04572_1_octaprenyl_pyrophosphate_synthase_putative____Plasmodium_chabaudi_adami
CAD2085044_1_octaprenyl_pyrophosphate_synthase_putative____Plasmodium_vinckei_lentum
CAD2096891_1_octaprenyl_pyrophosphate_synthase_putative____Plasmodium_vinckei
SBS92179_1_octaprenyl_pyrophosphate_synthase_putative_OPP____Plasmodium_ovale_curtisi
XP_008626448_1_hypothetical_protein_YYE_04588____Plasmodium_vinckei_vinckei
CAD2096766_1_octaprenyl_pyrophosphate_synthase_putative____Plasmodium_vinckei_petteri
EUD69754_1_hypothetical_protein_YYG_04974____Plasmodium_vinckei_petteri
CAD2085102_1_octaprenyl_pyrophosphate_synthase_putative____Plasmodium_vinckei_brucechwatti
VWU49717_1_octaprenyl_pyrophosphate_synthase_putative____Hepatocystis_sp__ex_Piliocolobus_tephrosceles
SBS80855_1_prenyl_transferase____Plasmodium_ovale_curtisi
SBT31919_1_prenyl_transferase____Plasmodium_ovale_wallikeri
XP_012760909_2_octaprenyl_pyrophosphate_synthase_putative____Plasmodium_reichenowi
SOV75279_1_octaprenyl_pyrophosphate_synthase____Plasmodium_reichenowi
CDO62259_1_octaprenyl_pyrophosphate_synthase_putative____Plasmodium_reichenowi
ETW32506_1_hypothetical_protein_PFFCH_00029____Plasmodium_falciparum_FCH_4
XP_001349541_1_octaprenyl_pyrophosphate_synthase____Plasmodium_falciparum_3D7
Plasmodium_falciparum3D7__165
PF3D7_0202700____Plasmodium_falciparum_3D7___octaprenyl_pyrophosphate_synthase___protein___length538
KNG76262_1_polyprenyl_synthetase____Plasmodium_falciparum_IGHCR14
SOS76202_1_octaprenyl_pyrophosphate_synthase____Plasmodium_sp__gorilla_clade_G1
SPJ08273_1_octaprenyl_pyrophosphate_synthase____Plasmodium_sp__DRC-Itaito
XP_018643685_1_octaprenyl_pyrophosphate_synthase____Plasmodium_gaboni
SOV20590_1_octaprenyl_pyrophosphate_synthase____Plasmodium_sp__DRC-Itaito
XP_028536260_1_octaprenyl_pyrophosphate_synthase____Plasmodium_sp__gorilla_clade_G2
XP_028531850_1_octaprenyl_pyrophosphate_synthase_putative____Plasmodium_relictum
XP_028528257_1_octaprenyl_pyrophosphate_synthase_putative____Plasmodium_gallinaceum
Haemoproteus_tartakovskyi__3994
Alphamonas_edax__37630
Alphamonas_edax__37618
Alphamonas_edax__37636
Digyalum_oweni1__5922
Digyalum_oweni2__1950
Nephromyces_occidentalis__31081
Nephromyces_occidentalis__31080
Nephromyces_occidentalis__13897
Rhytidocystis_sp__567
Rhytidocystis_sp2__6582
Vitrella_brassicaformis__21252
Chromera_velia__30004
Piridium__2019
Nephromyces_occidentalis__57699
Chromera_velia__3557
Colpodella_angusta2__85043
Colpodella_angusta2__85049
Colpodella_angusta2__85051
Colpodella_angusta1__17150
Digyalum_oweni1__24925
Colpodella_angusta1__19028
Colpodella_angusta1__63403
Theileria_equiWA__61271
Babesia_bigeminaBOND__85172
Babesia_bigeminaBOND__85171
Babesia_bovisT2Bo__43946
XP_001610655_1_polyprenyl_synthetase_superfamily_protein____Babesia_bovis_T2Bo
BBOV_IV007310____Babesia_bovis_T2Bo___polyprenyl_synthetase_superfamily_protein___protein___length457
Babesia_bovisT2Bo__43932
Babesia_bovisT2Bo__43944
Babesia_bovisT2Bo__43949
XP_028870789_1_putative_solanesyldiphosphate_synthase____Babesia_sp__Xinjiang
Babesia_bigeminaBOND__85173
Babesia_bigeminaBOND__85167
Babesia_microtiRI__2134
XP_021337355_1_hexaprenyldiphosphate_synthase____Babesia_microti_strain_RI
Babesia_microtiRI__2136
Theileria_equiWA__61263
TP03_0238____Theileria_parva_strain_Muguga___prenyl_transferase_putative___protein___length453
Cryptosporidium_ubiquitum39726__14725
Cryptosporidium_parvumIowaII__28787
Cryptosporidium_parvumIOWA_ATCC__28831
cgd7_3730____Cryptosporidium_parvum_Iowa_II___Polyprenyl_synthetase___protein___length466
Cryptosporidium_ubiquitum39726__14699
Cryptosporidium_hominisUdeA01__28665
Cryptosporidium_hominisTU502_2012__34060
Cryptosporidium_hominis37999__24562
Cryptosporidium_hominis30976__14625
Cryptosporidium_hominisUKH1__5681
Cryptosporidium_tyzzeriUGA55__27500
Cryptosporidium_spchipmunkLX2015__29314
Cryptosporidium_spchipmunkLX2015__29360
Cryptosporidium_meleagridisUKMEL1__13380
Cryptosporidium_meleagridisUKMEL1__13415
Cryptosporidium_meleagridisUKMEL1__13471
Cryptosporidium_murisRN66__16500
Cryptosporidium_andersoni30847__12858
Alphamonas_edax__56735
Alphamonas_edax__56740
Hammondia_hammondiHH34__520217
Hammondia_hammondiHH34__519557
EPR64929_1_polyprenyl_synthetase_superfamily_protein____Toxoplasma_gondii_GT1
Neospora_caninumLIV__396472
Hammondia_hammondiHH34__520002
KFG61515_1_polyprenyl_synthetase_superfamily_protein____Toxoplasma_gondii_RUB
KFG43915_1_polyprenyl_synthetase_superfamily_protein____Toxoplasma_gondii_GAB2-2007-GAL-DOM2
KFH06094_1_polyprenyl_synthetase_superfamily_protein____Toxoplasma_gondii_VAND
ESS36401_1_polyprenyl_synthetase_superfamily_protein____Toxoplasma_gondii_VEG
KFH16760_1_polyprenyl_synthetase_superfamily_protein____Toxoplasma_gondii_MAS
RQX75168_1_polyprenyl_synthetase_superfamily_protein____Toxoplasma_gondii_CAST
Hammondia_hammondiHH34__519694
KFG52388_1_polyprenyl_synthetase_superfamily_protein_partial____Toxoplasma_gondii_p89
KYF46571_1_polyprenyl_synthetase_superfamily_protein_partial____Toxoplasma_gondii_ARI
PIM01074_1_polyprenyl_synthetase_superfamily_protein_partial____Toxoplasma_gondii_COUG
CEL75399_1_TPA_hexaprenyl_pyrophosphate_synthetase_proteinputative____Toxoplasma_gondii_VEG
Toxoplasma_gondii__6237
KYK70935_1_polyprenyl_synthetase_superfamily_protein____Toxoplasma_gondii_TgCatPRC2
XP_018636584_1_polyprenyl_synthetase_superfamily_protein____Toxoplasma_gondii_ME49
TGME49_269430____Toxoplasma_gondii_ME49___polyprenyl_synthetase_superfamily_protein___protein___length676
Neospora_caninumLIV__396495
Neospora_caninumLIV__396518
Hammondia_hammondiHH34__520393
Neospora_caninumLIV__396512
CEL67914_1_TPA_Probable_hexaprenyl_pyrophosphate_synthasemitochondrial____Neospora_caninum_Liverpool
Hammondia_hammondiHH34__519928
Neospora_caninumLIV__396502
Neospora_caninumLIV__396486
XP_029219622_1_polyprenyl_synthetase_superfamily_protein____Besnoitia_besnoiti
PHJ23881_1_polyprenyl_synthetase_superfamily_protein____Cystoisospora_suis
Sarcocystis_neuronaSN3__48081
Sarcocystis_neuronaSOSN1__78590
Sarcocystis_neuronaSN3__48980
Sarcocystis_neuronaSOSN1__79596
Eimeria_tenellaHoughton__70962
Eimeria_falciformisBayerHaberkorn1970__201960
XP_026194109_1_prenyl_transferase____Cyclospora_cayetanensis
Eimeria_falciformisBayerHaberkorn1970__200523
Symbiont_X__13050
Symbiont_X__28678
Symbiont_X__28677
Colpodella_angusta1__8239
Chromera_velia__19695
Symbiont_X__6272
Colpodella_angusta1__8238
Colpodella_angusta1__8237
Colpodella_angusta2__9135
Selenidium_pygospionis__10642
Siedleckia_nematoides__61722
Alphamonas_edax__37422
Nephromyces_occidentalis__57663
Nephromyces_occidentalis__52814
Nephromyces_occidentalis__61269
Nephromyces_occidentalis__19821
Neospora_caninumLIV__396446
Nephromyces_occidentalis__29080
Nephromyces_occidentalis__62130
Nephromyces_occidentalis__72569
Nephromyces_occidentalis__74584
Nephromyces_occidentalis__223
Nephromyces_occidentalis__18399
Nephromyces_occidentalis__46942
Chromera_velia__30005
Vitrella_brassicaformis__13777
Piridium__5083
Vitrella_brassicaformis__5757
Platyproteum_sp__1830
Digyalum_oweni1__28706
Digyalum_oweni2__10374
Digyalum_oweni2__10111
KFZ19503_1_hypothetical_protein_V502_03615____Pseudogymnoascus_sp__VKM_F-4520_FW-2644
KFY92650_1_hypothetical_protein_V500_04100____Pseudogymnoascus_sp__VKM_F-4518_FW-2643
KFY33025_1_hypothetical_protein_V495_08508____Pseudogymnoascus_sp__VKM_F-4514_FW-929
OBT52907_1_hypothetical_protein_VE04_06381____Pseudogymnoascus_sp__24MN13
XP_018127232_1_hypothetical_protein_VE01_08678____Pseudogymnoascus_verrucosus
KFY72381_1_hypothetical_protein_V499_07468____Pseudogymnoascus_sp__VKM_F-103
KFX88211_1_hypothetical_protein_O988_09115____Pseudogymnoascus_sp__VKM_F-3808
OBT72696_1_hypothetical_protein_VF21_08826____Pseudogymnoascus_sp__05NY08
KFX89284_1_hypothetical_protein_V490_07126____Pseudogymnoascus_sp__VKM_F-3557
KFY16620_1_hypothetical_protein_V492_01205____Pseudogymnoascus_sp__VKM_F-4246
XP_024319760_1_coq1_putative_hexaprenyl_diphosphate_synthase____Pseudogymnoascus_destructans
KFY27494_1_hypothetical_protein_V493_03450____Pseudogymnoascus_sp__VKM_F-4281_FW-2241
ELR05863_1_hypothetical_protein_GMDG_07636____Pseudogymnoascus_destructans_20631-21
KFY63715_1_hypothetical_protein_V496_03742____Pseudogymnoascus_sp__VKM_F-4515_FW-2607
KFY13259_1_hypothetical_protein_V491_06470____Pseudogymnoascus_sp__VKM_F-3775
Digyalum_oweni1__5625
EEH23488_2_hypothetical_protein_PABG_05699____Paracoccidioides_brasiliensis_Pb03
KAF2227812_1_putative_hexaprenyl_pyrophosphate_synthetase_Coq1____Elsinoe_ampelina
PNS21684_1_hypothetical_protein_CAC42_1538____Sphaceloma_murrayae
KAF4301221_1_Polyprenyl_synthetase____Botryosphaeria_dothidea
OMJ20805_1_putative_hexaprenyl_pyrophosphate_synthase_mitochondrial____Smittium_culicis
OMJ23662_1_putative_hexaprenyl_pyrophosphate_synthase_mitochondrial____Smittium_culicis
Symbiont_X__16207
Hammondia_hammondiHH34__9430
Colpodella_angusta1__63407
XP_030829069_1_decaprenyldiphosphate_synthase_subunit_1____Strongylocentrotus_purpuratus
XP_018476387_1_PREDICTED_solanesyl_diphosphate_synthase_3_chloroplastic_mitochondrial_isoform_X2____Raphanus_sativus
XP_013734270_1_solanesyl_diphosphate_synthase_3_chloroplastic_mitochondrial_isoform_X2____Brassica_napus
XP_006410610_1_solanesyl_diphosphate_synthase_3_chloroplastic_mitochondrial_isoform_X2____Eutrema_salsugineum
KAF2566260_1_hypothetical_protein_F2Q68_00024476____Brassica_cretica
XP_013636449_1_PREDICTED_solanesyl_diphosphate_synthase_3_chloroplastic_mitochondrial_isoform_X2____Brassica_oleracea_var__oleracea
XP_013744871_1_solanesyl_diphosphate_synthase_3_chloroplastic_mitochondriallike_isoform_X2____Brassica_napus
NP_850234_1_geranyl_diphosphate_synthase_1____Arabidopsis_thaliana
XP_023639436_1_solanesyl_diphosphate_synthase_3_chloroplastic_mitochondrial_isoform_X2____Capsella_rubella
XP_019101318_1_PREDICTED_solanesyl_diphosphate_synthase_3_chloroplastic_mitochondrial_isoform_X2____Camelina_sativa
XP_010516612_1_PREDICTED_solanesyl_diphosphate_synthase_3_chloroplastic_mitochondrial_isoform_X2____Camelina_sativa
XP_020883166_1_solanesyl_diphosphate_synthase_3_chloroplastic_mitochondrial_isoform_X2____Arabidopsis_lyrata_subsp__lyrata
XP_020277177_1_solanesyldiphosphate_synthase_1_mitochondriallike_isoform_X1____Asparagus_officinalis
2.0

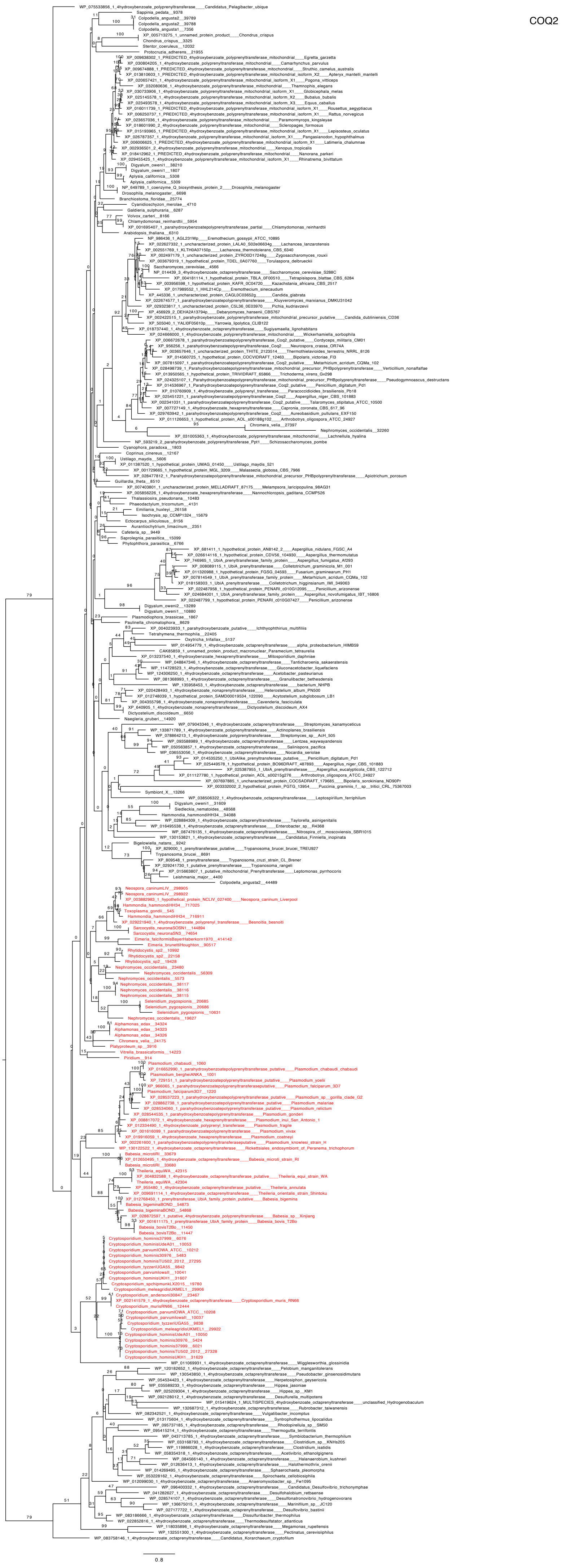
COQ2
0.8

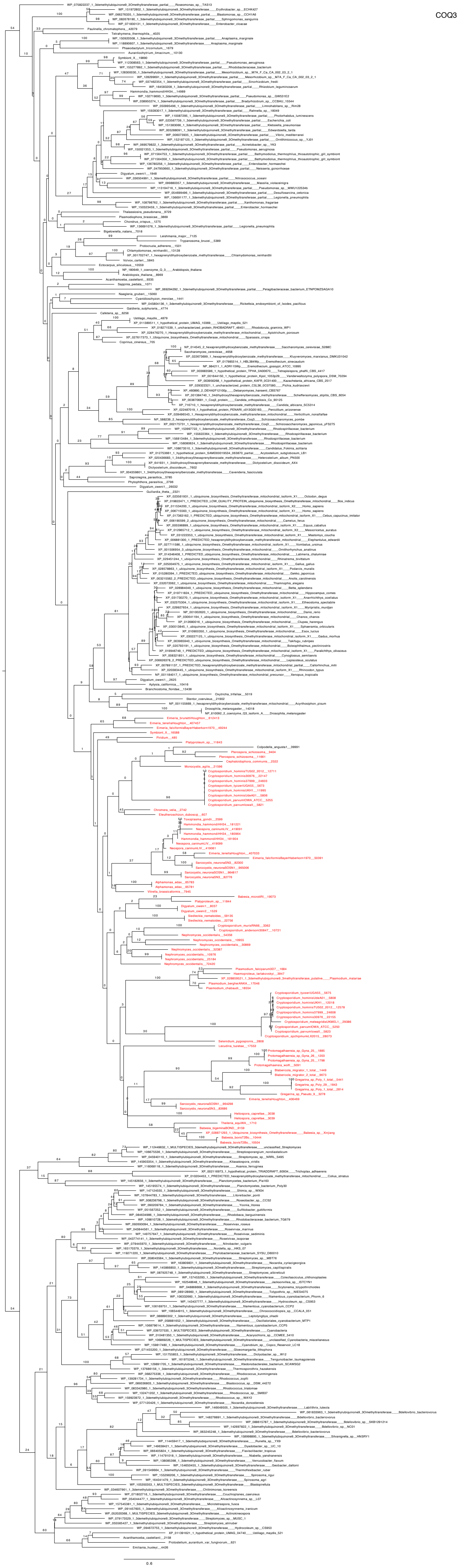
COQ3

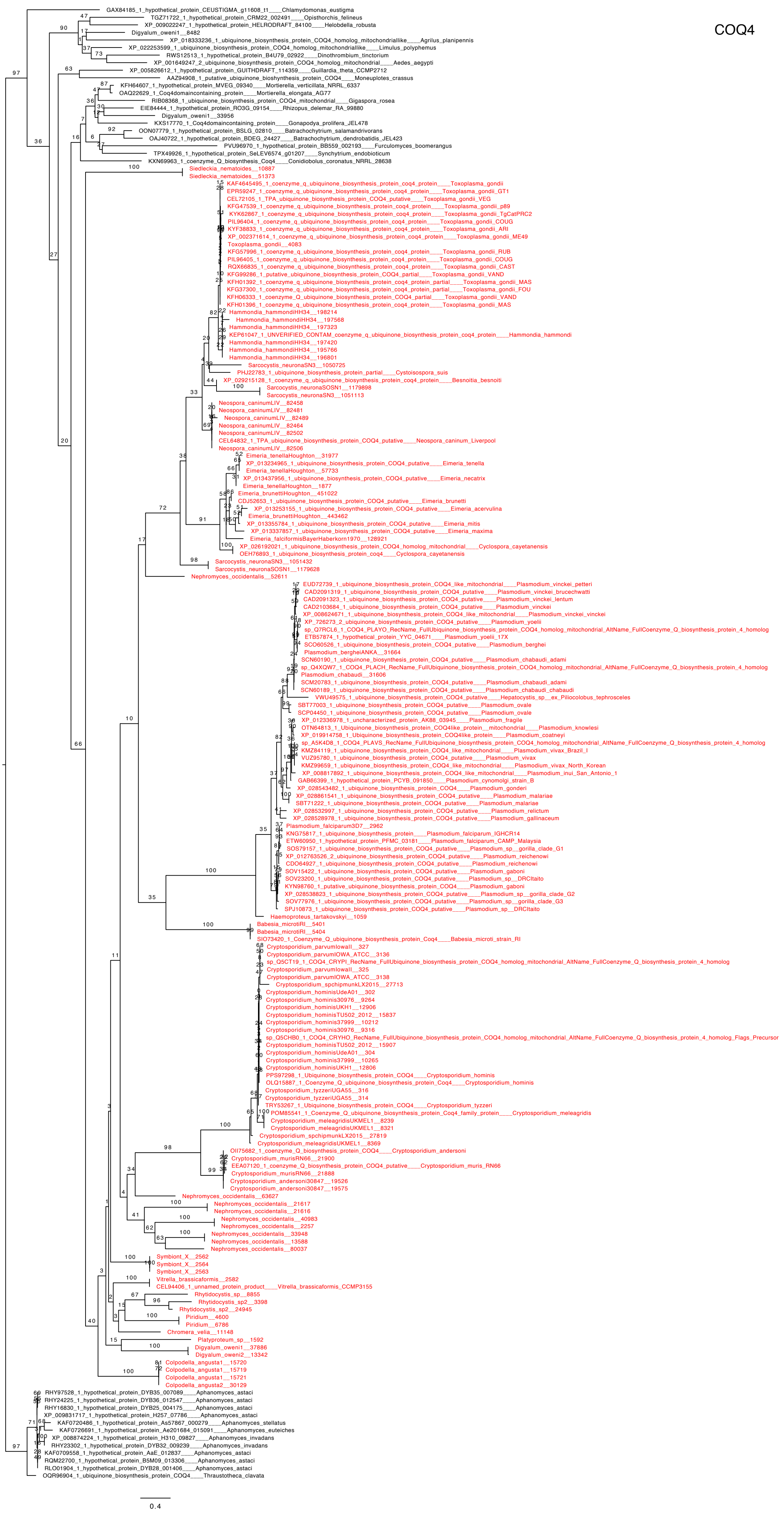
COQ4
GAX84185_1_hypothetical_protein_CEUSTIGMA_g11608_t1____Chlamydomonas_eustigma
TGZ71722_1_hypothetical_protein_CRM22_002491____Opisthorchis_felineus
XP_009022247_1_hypothetical_protein_HELRODRAFT_84100____Helobdella_robusta
Digyalum_oweni1__8482
XP_018333236_1_ubiquinone_biosynthesis_protein_COQ4_homolog_mitochondriallike____Agrilus_planipennis
XP_022253599_1_ubiquinone_biosynthesis_protein_COQ4_homolog_mitochondriallike____Limulus_polyphemus
RWS12513_1_hypothetical_protein_B4U79_02922____Dinothrombium_tinctorium
XP_001649247_2_ubiquinone_biosynthesis_protein_COQ4_homolog_mitochondrial____Aedes_aegypti
XP_005826612_1_hypothetical_protein_GUITHDRAFT_114359____Guillardia_theta_CCMP2712
AAZ94908_1_putative_ubiquinone_biosynthesis_protein_COQ4____Moneuplotes_crassus
KFH64607_1_hypothetical_protein_MVEG_09340____Mortierella_verticillata_NRRL_6337
OAQ22629_1_Coq4domaincontaining_protein____Mortierella_elongata_AG77
RIB08368_1_ubiquinone_biosynthesis_protein_COQ4_mitochondrial____Gigaspora_rosea
EIE84444_1_hypothetical_protein_RO3G_09154____Rhizopus_delemar_RA_99880
Digyalum_oweni1__33956
KXS17770_1_Coq4domaincontaining_protein____Gonapodya_prolifera_JEL478
OON07779_1_hypothetical_protein_BSLG_02810____Batrachochytrium_salamandrivorans
OAJ40722_1_hypothetical_protein_BDEG_24427____Batrachochytrium_dendrobatidis_JEL423
PVU96970_1_hypothetical_protein_BB559_002193____Furculomyces_boomerangus
TPX49926_1_hypothetical_protein_SeLEV6574_g01207____Synchytrium_endobioticum
KXN69963_1_coenzyme_Q_biosynthesis_Coq4____Conidiobolus_coronatus_NRRL_28638
Siedleckia_nematoides__10887
Siedleckia_nematoides__51373
KAF4645495_1_coenzyme_q_ubiquinone_biosynthesis_protein_coq4_protein____Toxoplasma_gondii
EPR59247_1_coenzyme_q_ubiquinone_biosynthesis_protein_coq4_protein____Toxoplasma_gondii_GT1
CEL72105_1_TPA_ubiquinone_biosynthesis_protein_COQ4_putative____Toxoplasma_gondii_VEG
KFG47539_1_coenzyme_q_ubiquinone_biosynthesis_protein_coq4_protein____Toxoplasma_gondii_p89
KYK62867_1_coenzyme_q_ubiquinone_biosynthesis_protein_coq4_protein____Toxoplasma_gondii_TgCatPRC2
PIL96404_1_coenzyme_q_ubiquinone_biosynthesis_protein_coq4_protein____Toxoplasma_gondii_COUG
KYF38833_1_coenzyme_q_ubiquinone_biosynthesis_protein_coq4_protein____Toxoplasma_gondii_ARI
XP_002371614_1_coenzyme_q_ubiquinone_biosynthesis_protein_coq4_protein____Toxoplasma_gondii_ME49
Toxoplasma_gondii__4083
KFG57996_1_coenzyme_q_ubiquinone_biosynthesis_protein_coq4_protein____Toxoplasma_gondii_RUB
PIL96405_1_coenzyme_q_ubiquinone_biosynthesis_protein_coq4_protein____Toxoplasma_gondii_COUG
RQX66835_1_coenzyme_q_ubiquinone_biosynthesis_protein_coq4_protein____Toxoplasma_gondii_CAST
KFG99286_1_putative_ubiquinone_biosynthesis_protein_COQ4_partial____Toxoplasma_gondii_VAND
KFH01392_1_coenzyme_q_ubiquinone_biosynthesis_protein_coq4_protein_partial____Toxoplasma_gondii_MAS
KFG37300_1_coenzyme_q_ubiquinone_biosynthesis_protein_coq4_protein_partial____Toxoplasma_gondii_FOU
KFH06333_1_coenzyme_Q_ubiquinone_biosynthesis_protein_COQ4_partial____Toxoplasma_gondii_VAND
KFH01396_1_coenzyme_q_ubiquinone_biosynthesis_protein_coq4_protein____Toxoplasma_gondii_MAS
Hammondia_hammondiHH34__198214
Hammondia_hammondiHH34__197568
Hammondia_hammondiHH34__197323
KEP61047_1_UNVERIFIED_CONTAM_coenzyme_q_ubiquinone_biosynthesis_protein_coq4_protein____Hammondia_hammondi
Hammondia_hammondiHH34__197420
Hammondia_hammondiHH34__195766
Hammondia_hammondiHH34__196801
Sarcocystis_neuronaSN3__1050725
PHJ22783_1_ubiquinone_biosynthesis_protein_partial____Cystoisospora_suis
XP_029215128_1_coenzyme_q_ubiquinone_biosynthesis_protein_coq4_protein____Besnoitia_besnoiti
Sarcocystis_neuronaSOSN1__1179898
Sarcocystis_neuronaSN3__1051113
Neospora_caninumLIV__82458
Neospora_caninumLIV__82481
Neospora_caninumLIV__82489
Neospora_caninumLIV__82464
Neospora_caninumLIV__82502
CEL64832_1_TPA_ubiquinone_biosynthesis_protein_COQ4_putative____Neospora_caninum_Liverpool
Neospora_caninumLIV__82506
Eimeria_tenellaHoughton__31977
XP_013234965_1_ubiquinone_biosynthesis_protein_COQ4_putative____Eimeria_tenella
Eimeria_tenellaHoughton__57733
XP_013437956_1_ubiquinone_biosynthesis_protein_COQ4_putative____Eimeria_necatrix
Eimeria_tenellaHoughton__1877
Eimeria_brunettiHoughton__451022
CDJ52653_1_ubiquinone_biosynthesis_protein_COQ4_putative____Eimeria_brunetti
XP_013253155_1_ubiquinone_biosynthesis_protein_COQ4_putative____Eimeria_acervulina
Eimeria_brunettiHoughton__443462
XP_013355784_1_ubiquinone_biosynthesis_protein_COQ4_putative____Eimeria_mitis
XP_013337857_1_ubiquinone_biosynthesis_protein_COQ4_putative____Eimeria_maxima
Eimeria_falciformisBayerHaberkorn1970__128921
XP_026192021_1_ubiquinone_biosynthesis_protein_COQ4_homolog_mitochondrial____Cyclospora_cayetanensis
OEH78893_1_ubiquinone_biosynthesis_protein_coq4____Cyclospora_cayetanensis
Sarcocystis_neuronaSN3__1051432
Sarcocystis_neuronaSOSN1__1179628
Nephromyces_occidentalis__52611
EUD72739_1_ubiquinone_biosynthesis_protein_COQ4_like_mitochondrial____Plasmodium_vinckei_petteri
CAD2091319_1_ubiquinone_biosynthesis_protein_COQ4_putative____Plasmodium_vinckei_brucechwatti
CAD2091323_1_ubiquinone_biosynthesis_protein_COQ4_putative____Plasmodium_vinckei_lentum
CAD2103684_1_ubiquinone_biosynthesis_protein_COQ4_putative____Plasmodium_vinckei
XP_008824671_1_ubiquinone_biosynthesis_protein_COQ4_like_mitochondrial____Plasmodium_vinckei_vinckei
XP_726273_2_ubiquinone_biosynthesis_protein_COQ4_putative____Plasmodium_yoelii
sp_Q7RCL6_1_COQ4_PLAYO_RecName_FullUbiquinone_biosynthesis_protein_COQ4_homolog_mitochondrial_AltName_FullCoenzyme_Q_biosynthesis_protein_4_homolog
ETB57874_1_hypothetical_protein_YYC_04671____Plasmodium_yoelii_17X
SCO60526_1_ubiquinone_biosynthesis_protein_COQ4_putative____Plasmodium_berghei
Plasmodium_bergheiANKA__31664
SCN60190_1_ubiquinone_biosynthesis_protein_COQ4_putative____Plasmodium_chabaudi_adami
sp_Q4XQW7_1_COQ4_PLACH_RecName_FullUbiquinone_biosynthesis_protein_COQ4_homolog_mitochondrial_AltName_FullCoenzyme_Q_biosynthesis_protein_4_homolog
Plasmodium_chabaudi__31606
SCM20783_1_ubiquinone_biosynthesis_protein_COQ4_putative____Plasmodium_chabaudi_adami
SCN60189_1_ubiquinone_biosynthesis_protein_COQ4_putative____Plasmodium_chabaudi_chabaudi
VWU49575_1_ubiquinone_biosynthesis_protein_COQ4_putative____Hepatocystis_sp__ex_Piliocolobus_tephrosceles
SBT77003_1_ubiquinone_biosynthesis_protein_COQ4_putative____Plasmodium_ovale
SCP04450_1_ubiquinone_biosynthesis_protein_COQ4_putative____Plasmodium_ovale
XP_012336978_1_uncharacterized_protein_AK88_03945____Plasmodium_fragile
OTN64813_1_Ubiquinone_biosynthesis_protein_COQ4like_protein__mitochondrial____Plasmodium_knowlesi
XP_019914758_1_Ubiquinone_biosynthesis_protein_COQ4like_protein____Plasmodium_coatneyi
sp_A5K4D8_1_COQ4_PLAVS_RecName_FullUbiquinone_biosynthesis_protein_COQ4_homolog_mitochondrial_AltName_FullCoenzyme_Q_biosynthesis_protein_4_homolog
KMZ84119_1_ubiquinone_biosynthesis_protein_COQ4_like_mitochondrial____Plasmodium_vivax_Brazil_I
VUZ95780_1_ubiquinone_biosynthesis_protein_COQ4_putative____Plasmodium_vivax
KMZ99659_1_ubiquinone_biosynthesis_protein_COQ4_like_mitochondrial____Plasmodium_vivax_North_Korean
XP_008817892_1_ubiquinone_biosynthesis_protein_COQ4_like_mitochondrial____Plasmodium_inui_San_Antonio_1
GAB66399_1_hypothetical_protein_PCYB_091850____Plasmodium_cynomolgi_strain_B
XP_028543482_1_ubiquinone_biosynthesis_protein_COQ4____Plasmodium_gonderi
XP_028861541_1_ubiquinone_biosynthesis_protein_COQ4_putative____Plasmodium_malariae
SBT71222_1_ubiquinone_biosynthesis_protein_COQ4_putative____Plasmodium_malariae
XP_028532997_1_ubiquinone_biosynthesis_protein_COQ4_putative____Plasmodium_relictum
XP_028528978_1_ubiquinone_biosynthesis_protein_COQ4_putative____Plasmodium_gallinaceum
Plasmodium_falciparum3D7__2962
KNG75817_1_ubiquinone_biosynthesis_protein____Plasmodium_falciparum_IGHCR14
ETW60950_1_hypothetical_protein_PFMC_03181____Plasmodium_falciparum_CAMP_Malaysia
SOS79157_1_ubiquinone_biosynthesis_protein_COQ4_putative____Plasmodium_sp__gorilla_clade_G1
XP_012763526_2_ubiquinone_biosynthesis_protein_COQ4_putative____Plasmodium_reichenowi
CDO64927_1_ubiquinone_biosynthesis_protein_COQ4_putative____Plasmodium_reichenowi
SOV15422_1_ubiquinone_biosynthesis_protein_COQ4_putative____Plasmodium_gaboni
SOV23200_1_ubiquinone_biosynthesis_protein_COQ4_putative____Plasmodium_sp__DRC1taito
KYN98760_1_putative_ubiquinone_biosynthesis_protein_COQ4____Plasmodium_gaboni
XP_028538823_1_ubiquinone_biosynthesis_protein_COQ4_putative____Plasmodium_sp__gorilla_clade_G2
SOV77976_1_ubiquinone_biosynthesis_protein_COQ4_putative____Plasmodium_sp__gorilla_clade_G3
SPJ10873_1_ubiquinone_biosynthesis_protein_COQ4_putative____Plasmodium_sp__DRC1taito
Haemoproteus_tartakovskyi__1059
Babesia_microtiRI__5401
Babesia_microtiRI__5404
SIO73420_1_Coenzyme_Q_ubiquinone_biosynthesis_protein_Coq4____Babesia_microti_strain_RI
Cryptosporidium_parvumIowaII__327
Cryptosporidium_parvumIOWA_ATCC__3136
sp_Q5CT19_1_COQ4_CRYPI_RecName_FullUbiquinone_biosynthesis_protein_COQ4_homolog_mitochondrial_AltName_FullCoenzyme_Q_biosynthesis_protein_4_homolog
Cryptosporidium_parvumIowaII__325
Cryptosporidium_parvumIOWA_ATCC__3138
Cryptosporidium_spchipmunkLX2015__27713
Cryptosporidium_hominisUdeA01__302
Cryptosporidium_hominis30976__9264
Cryptosporidium_hominisUKH1__12906
Cryptosporidium_hominisTU502_2012__15837
Cryptosporidium_hominis37999__10212
Cryptosporidium_hominis30976__9316
sp_Q5CHB0_1_COQ4_CRYHO_RecName_FullUbiquinone_biosynthesis_protein_COQ4_homolog_mitochondrial_AltName_FullCoenzyme_Q_biosynthesis_protein_4_homolog_Flags_Precursor
Cryptosporidium_hominisTU502_2012__15907
Cryptosporidium_hominisUdeA01__304
Cryptosporidium_hominis37999__10265
Cryptosporidium_hominisUKH1__12806
PPS97298_1_Ubiquinone_biosynthesis_protein_COQ4____Cryptosporidium_hominis
OLQ15887_1_Coenzyme_Q_ubiquinone_biosynthesis_protein_Coq4____Cryptosporidium_hominis
Cryptosporidium_tyzzeriUGA55__316
Cryptosporidium_tyzzeriUGA55__314
TRY53267_1_Ubiquinone_biosynthesis_protein_COQ4____Cryptosporidium_tyzzeri
POM85541_1_Coenzyme_Q_ubiquinone_biosynthesis_protein_Coq4_family_protein____Cryptosporidium_meleagridis
Cryptosporidium_meleagridisUKMEL1__8239
Cryptosporidium_meleagridisUKMEL1__8321
Cryptosporidium_spchipmunkLX2015__27819
Cryptosporidium_meleagridisUKMEL1__8369
OII75682_1_coenzyme_Q_biosynthesis_protein_COQ4____Cryptosporidium_andersoni
Cryptosporidium_murisRN66__21900
EEA07120_1_coenzyme_Q_biosynthesis_protein_COQ4_putative____Cryptosporidium_muris_RN66
Cryptosporidium_murisRN66__21888
Cryptosporidium_andersoni30847__19526
Cryptosporidium_andersoni30847__19575
Nephromyces_occidentalis__63627
Nephromyces_occidentalis__21617
Nephromyces_occidentalis__21616
Nephromyces_occidentalis__40983
Nephromyces_occidentalis__2257
Nephromyces_occidentalis__33948
Nephromyces_occidentalis__13588
Nephromyces_occidentalis__80037
Symbiont_X__2562
Symbiont_X__2564
Symbiont_X__2563
Vitrella_brassicaformis__2582
CEL94406_1_unnamed_protein_product____Vitrella_brassicaformis_CCMP3155
Rhytidocystis_sp__8855
Rhytidocystis_sp2__3398
Rhytidocystis_sp2__24945
Piridium__4600
Piridium__6786
Chromera_velia__11148
Platyproteum_sp__1592
Digyalum_oweni1__37886
Digyalum_oweni2__13342
Colpodella_angusta1__15720
Colpodella_angusta1__15719
Colpodella_angusta1__15721
Colpodella_angusta2__30129
RHY97528_1_hypothetical_protein_DYB35_007089____Aphanomyces_astaci
RHY24225_1_hypothetical_protein_DYB36_012547____Aphanomyces_astaci
RHY16830_1_hypothetical_protein_DYB25_004175____Aphanomyces_astaci
XP_009831717_1_hypothetical_protein_H257_07786____Aphanomyces_astaci
KAF0720486_1_hypothetical_protein_As57867_000279____Aphanomyces_stellatus
KAF0726691_1_hypothetical_protein_Ae201684_015091____Aphanomyces_euteiches
XP_008874224_1_hypothetical_protein_H310_09827____Aphanomyces_invadans
RHY23302_1_hypothetical_protein_DYB32_009239____Aphanomyces_invadans
KAF0709558_1_hypothetical_protein_AaE_012837____Aphanomyces_astaci
RQM22700_1_hypothetical_protein_B5M09_013306____Aphanomyces_astaci
RLO01904_1_hypothetical_protein_DYB28_001406____Aphanomyces_astaci
OQR96904_1_ubiquinone_biosynthesis_protein_COQ4____Thraustotheca_clavata
0.4

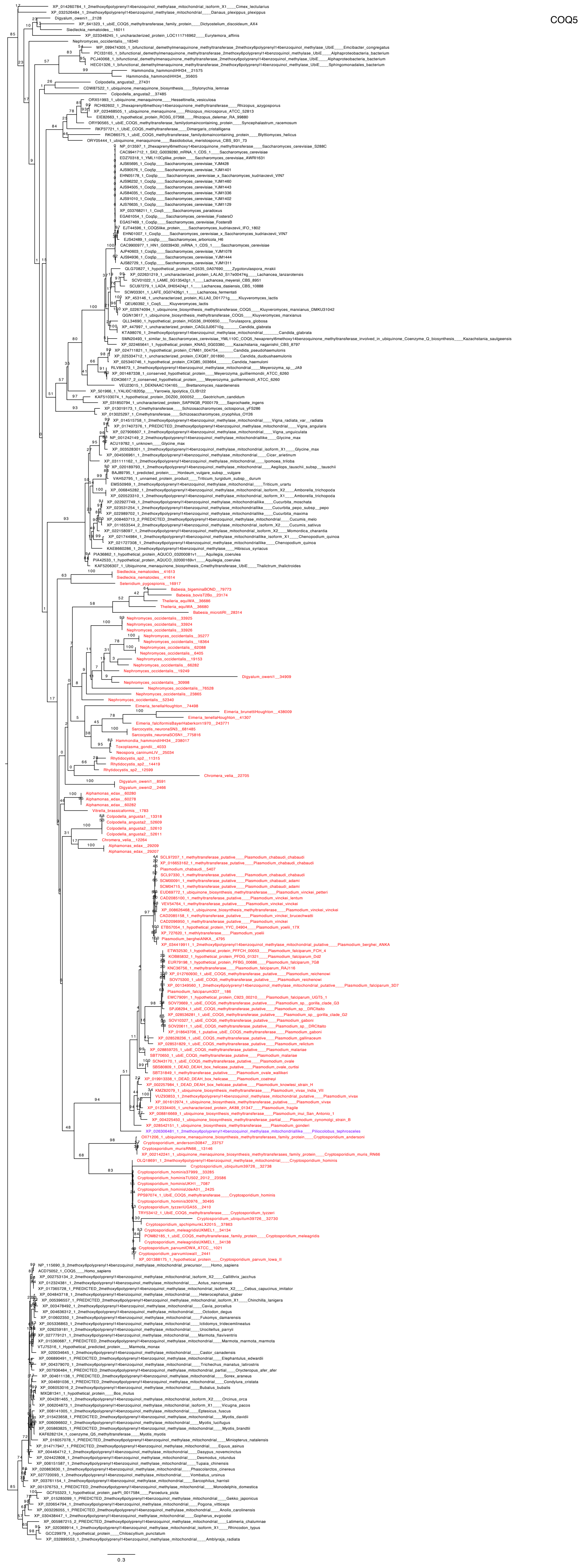
COQ5
0.3

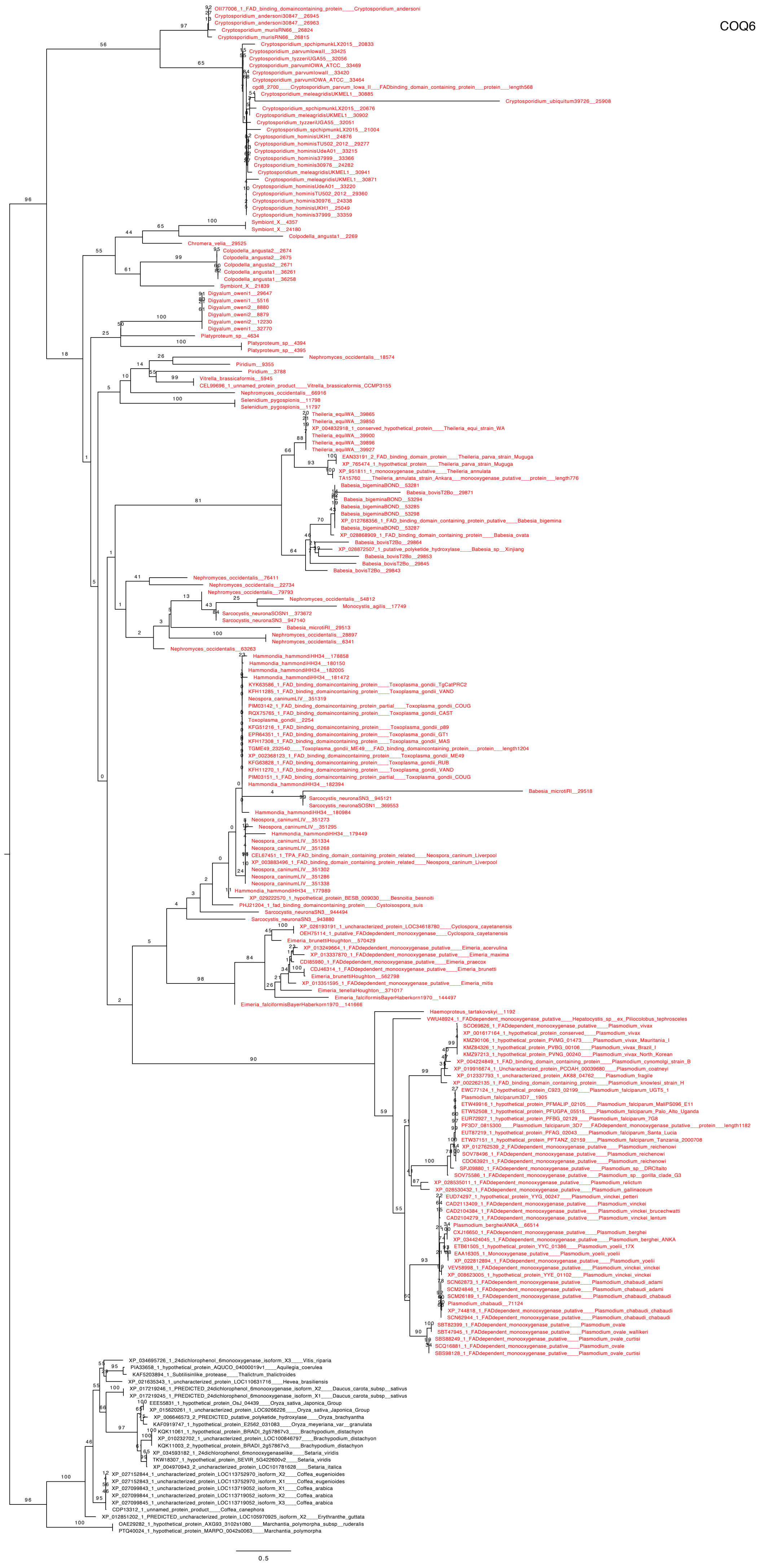
COQ6
0.5

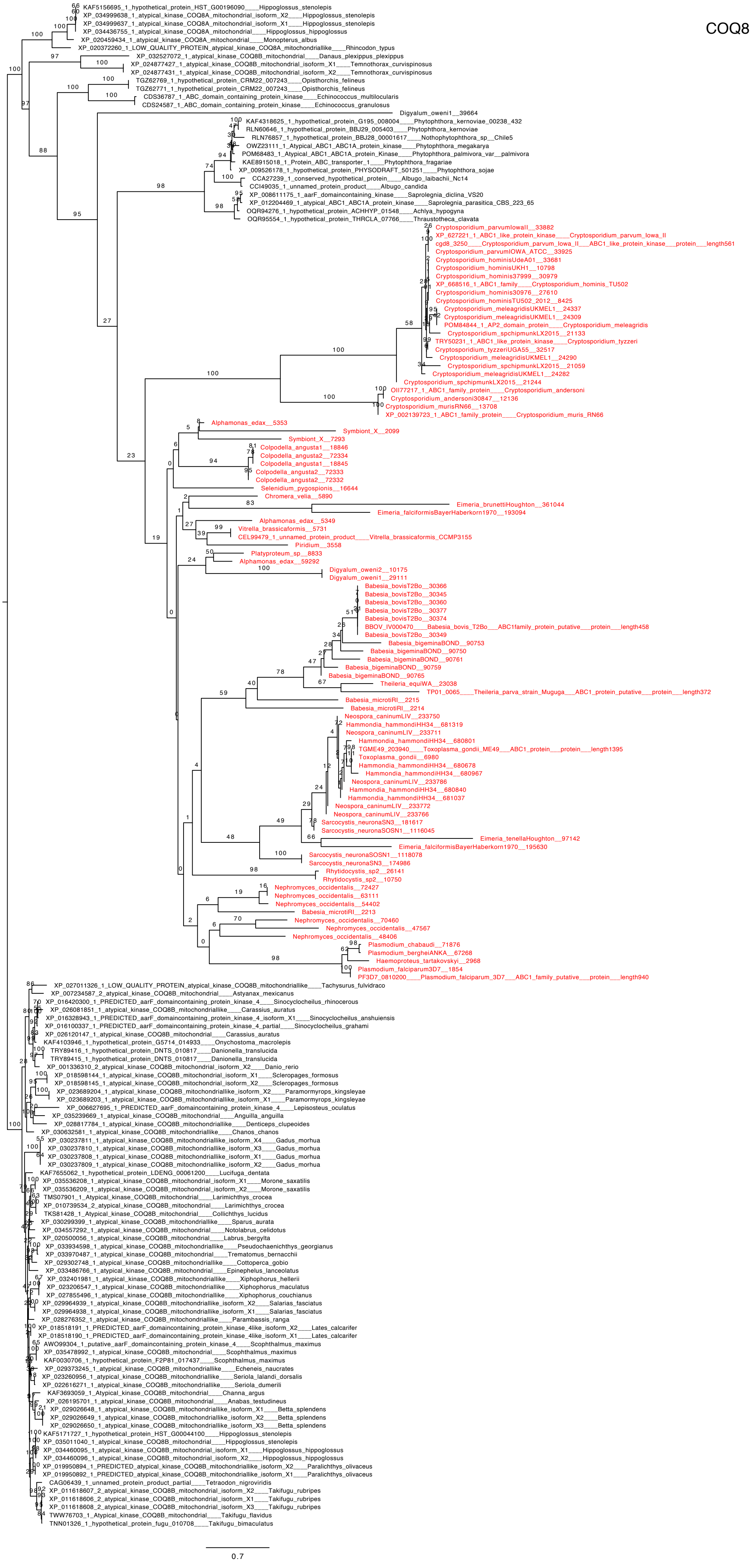
COQ8
KAF5156695_1_hypothetical_protein_HST_G00196090____Hippoglossus_stenolepis
XP_034999638_1_atypical_kinase_COQ8A_mitochondrial_isoform_X2____Hippoglossus_stenolepis
XP_034999637_1_atypical_kinase_COQ8A_mitochondrial_isoform_X1____Hippoglossus_stenolepis
XP_034436755_1_atypical_kinase_COQ8A_mitochondrial____Hippoglossus_hippoglossus
XP_020459434_1_atypical_kinase_COQ8A_mitochondrial____Monopterus_albus
XP_020372260_1_LOW_QUALITY_PROTEIN_atypical_kinase_COQ8A_mitochondriallike____Rhincodon_typus
XP_032527072_1_atypical_kinase_COQ8B_mitochondrial____Danaus_plexippus_plexippus
XP_024877427_1_atypical_kinase_COQ8B_mitochondrial_isoform_X1____Temnothorax_curvispinosus
XP_024877431_1_atypical_kinase_COQ8B_mitochondrial_isoform_X2____Temnothorax_curvispinosus
TGZ62769_1_hypothetical_protein_CRM22_007243____Opisthorchis_felineus
TGZ62771_1_hypothetical_protein_CRM22_007243____Opisthorchis_felineus
CDS36787_1_ABC_domain_containing_protein_kinase____Echinococcus_multilocularis
CDS24587_1_ABC_domain_containing_protein_kinase____Echinococcus_granulosus
Digyalum_oweni1__39664
KAF4318625_1_hypothetical_protein_G195_008004____Phytophthora_kernoviae_00238_432
RLN60646_1_hypothetical_protein_BBJ29_005403____Phytophthora_kernoviae
RLN76857_1_hypothetical_protein_BBJ28_00001617____Nothophytophthora_sp__Chile5
OWZ23111_1_Atypical_ABC1_ABC1A_protein_kinase____Phytophthora_megakarya
POM68483_1_Atypical_ABC1_ABC1A_protein_Kinase____Phytophthora_palmivora_var__palmivora
KAE8915018_1_Protein_ABC_transporter_1____Phytophthora_fragariae
XP_009526178_1_hypothetical_protein_PHYSODRAFT_501251____Phytophthora_sojae
CCA27239_1_conserved_hypothetical_protein____Albugo_laibachii_Nc14
CCI49035_1_unnamed_protein_product____Albugo_candida
XP_008611175_1_aarF_domaincontaining_kinase____Saprolegnia_diclina_VS20
XP_012204469_1_atypical_ABC1_ABC1A_protein_kinase____Saprolegnia_parasitica_CBS_223_65
OQR94276_1_hypothetical_protein_ACHHYP_01548____Achlya_hypogyna
OQR95554_1_hypothetical_protein_THRCLA_07766____Thraustotheca_clavata
Cryptosporidium_parvumIowaII__33882
XP_627221_1_ABC1_like_protein_kinase____Cryptosporidium_parvum_Iowa_II
cgd8_3250____Cryptosporidium_parvum_Iowa_II___ABC1_like_protein_kinase___protein___length561
Cryptosporidium_parvumIOWA_ATCC__33925
Cryptosporidium_hominisUdeA01__33681
Cryptosporidium_hominisUKH1__10798
Cryptosporidium_hominis37999__30979
XP_668516_1_ABC1_family____Cryptosporidium_hominis_TU502
Cryptosporidium_hominis30976__27610
Cryptosporidium_hominisTU502_2012__8425
Cryptosporidium_meleagridisUKMEL1__24337
Cryptosporidium_meleagridisUKMEL1__24309
POM84844_1_AP2_domain_protein____Cryptosporidium_meleagridis
Cryptosporidium_spchipmunkLX2015__21133
TRY50231_1_ABC1_like_protein_kinase____Cryptosporidium_tyzzeri
Cryptosporidium_tyzzeriUGA55__32517
Cryptosporidium_meleagridisUKMEL1__24290
Cryptosporidium_spchipmunkLX2015__21059
Cryptosporidium_meleagridisUKMEL1__24282
Cryptosporidium_spchipmunkLX2015__21244
OII77217_1_ABC1_family_protein____Cryptosporidium_andersoni
Cryptosporidium_andersoni30847__12136
Cryptosporidium_murisRN66__13708
XP_002139723_1_ABC1_family_protein____Cryptosporidium_muris_RN66
Alphamonas_edax__5353
Symbiont_X__2099
Symbiont_X__7293
Colpodella_angusta1__18846
Colpodella_angusta2__72334
Colpodella_angusta1__18845
Colpodella_angusta2__72333
Colpodella_angusta2__72332
Selenidium_pygospionis__16644
Chromera_velia__5890
Eimeria_brunettiHoughton__361044
Eimeria_falciformisBayerHaberkorn1970__193094
Alphamonas_edax__5349
Vitrella_brassicaformis__5731
CEL99479_1_unnamed_protein_product____Vitrella_brassicaformis_CCMP3155
Piridium__3558
Platyproteum_sp__8833
Alphamonas_edax__59292
Digyalum_oweni2__10175
Digyalum_oweni1__29111
Babesia_bovisT2Bo__30366
Babesia_bovisT2Bo__30345
Babesia_bovisT2Bo__30360
Babesia_bovisT2Bo__30377
Babesia_bovisT2Bo__30374
BBOV_IV000470____Babesia_bovis_T2Bo___ABC1family_protein_putative___protein___length458
Babesia_bovisT2Bo__30349
Babesia_bigeminaBOND__90753
Babesia_bigeminaBOND__90750
Babesia_bigeminaBOND__90761
Babesia_bigeminaBOND__90759
Babesia_bigeminaBOND__90765
Theileria_equiWA__23038
TP01_0065____Theileria_parva_strain_Muguga___ABC1_protein_putative___protein___length372
Babesia_microtiRI__2215
Babesia_microtiRI__2214
Neospora_caninumLIV__233750
Hammondia_hammondiHH34__681319
Neospora_caninumLIV__233711
Hammondia_hammondiHH34__680801
TGME49_203940____Toxoplasma_gondii_ME49___ABC1_protein___protein___length1395
Toxoplasma_gondii__6980
Hammondia_hammondiHH34__680678
Hammondia_hammondiHH34__680967
Neospora_caninumLIV__233786
Hammondia_hammondiHH34__680840
Hammondia_hammondiHH34__681037
Neospora_caninumLIV__233772
Neospora_caninumLIV__233766
Sarcocystis_neuronaSN3__181617
Sarcocystis_neuronaSOSN1__1116045
Eimeria_tenellaHoughton__97142
Eimeria_falciformisBayerHaberkorn1970__195630
Sarcocystis_neuronaSOSN1__1118078
Sarcocystis_neuronaSN3__174986
Rhytidocystis_sp2__26141
Rhytidocystis_sp2__10750
Nephromyces_occidentalis__72427
Nephromyces_occidentalis__63111
Nephromyces_occidentalis__54402
Babesia_microtiRI__2213
Nephromyces_occidentalis__70460
Nephromyces_occidentalis__47567
Nephromyces_occidentalis__48406
Plasmodium_chabaudi__71876
Plasmodium_bergheiANKA__67268
Haemoproteus_tartakovskyi__2968
Plasmodium_falciparum3D7__1854
PF3D7_0810200____Plasmodium_falciparum_3D7___ABC1_family_putative___protein___length940
XP_027011326_1_LOW_QUALITY_PROTEIN_atypical_kinase_COQ8B_mitochondriallike____Tachysurus_fulvidraco
XP_007234587_2_atypical_kinase_COQ8B_mitochondrial____Astyanax_mexicanus
XP_016420300_1_PREDICTED_aarF_domaincontaining_protein_kinase_4____Sinocyclocheilus_rhinocerous
XP_026081851_1_atypical_kinase_COQ8B_mitochondriallike____Carassius_auratus
XP_016328943_1_PREDICTED_aarF_domaincontaining_protein_kinase_4_isoform_X1____Sinocyclocheilus_anshuiensis
XP_016100337_1_PREDICTED_aarF_domaincontaining_protein_kinase_4_partial____Sinocyclocheilus_grahami
XP_026120147_1_atypical_kinase_COQ8B_mitochondrial____Carassius_auratus
KAF4103946_1_hypothetical_protein_G5714_014933____Onychostoma_macrolepis
TRY89416_1_hypothetical_protein_DNTS_010817____Danionella_translucida
TRY89415_1_hypothetical_protein_DNTS_010817____Danionella_translucida
XP_001336310_2_atypical_kinase_COQ8B_mitochondrial_isoform_X2____Danio_rerio
XP_018598144_1_atypical_kinase_COQ8B_mitochondrial_isoform_X1____Scleropages_formosus
XP_018598145_1_atypical_kinase_COQ8B_mitochondrial_isoform_X2____Scleropages_formosus
XP_023689204_1_atypical_kinase_COQ8B_mitochondriallike_isoform_X2____Paramormyrops_kingsleyae
XP_023689203_1_atypical_kinase_COQ8B_mitochondriallike_isoform_X1____Paramormyrops_kingsleyae
XP_006627695_1_PREDICTED_aarF_domaincontaining_protein_kinase_4____Lepisosteus_oculatus
XP_035239669_1_atypical_kinase_COQ8B_mitochondrial____Anguilla_anguilla
XP_028817784_1_atypical_kinase_COQ8B_mitochondriallike____Denticeps_clupeoides
XP_030632581_1_atypical_kinase_COQ8B_mitochondriallike____Chanos_chanos
XP_030237811_1_atypical_kinase_COQ8B_mitochondriallike_isoform_X4____Gadus_morhua
XP_030237810_1_atypical_kinase_COQ8B_mitochondriallike_isoform_X3____Gadus_morhua
XP_030237808_1_atypical_kinase_COQ8B_mitochondriallike_isoform_X1____Gadus_morhua
XP_030237809_1_atypical_kinase_COQ8B_mitochondriallike_isoform_X2____Gadus_morhua
KAF7655062_1_hypothetical_protein_LDENG_00061200____Lucifuga_dentata
XP_035536208_1_atypical_kinase_COQ8B_mitochondrial_isoform_X1____Morone_saxatilis
XP_035536209_1_atypical_kinase_COQ8B_mitochondrial_isoform_X2____Morone_saxatilis
TMS07901_1_Atypical_kinase_COQ8B_mitochondrial____Larimichthys_crocea
XP_010739534_2_atypical_kinase_COQ8B_mitochondrial____Larimichthys_crocea
TKS81428_1_Atypical_kinase_COQ8B_mitochondrial____Collichthys_lucidus
XP_030299399_1_atypical_kinase_COQ8B_mitochondriallike____Sparus_aurata
XP_034557292_1_atypical_kinase_COQ8B_mitochondrial____Notolabrus_celidotus
XP_020500056_1_atypical_kinase_COQ8B_mitochondrial____Labrus_bergylta
XP_033934598_1_atypical_kinase_COQ8B_mitochondriallike____Pseudochaenichthys_georgianus
XP_033970487_1_atypical_kinase_COQ8B_mitochondrial____Trematomus_bernacchii
XP_029302748_1_atypical_kinase_COQ8B_mitochondriallike____Cottoperca_gobio
XP_033486766_1_atypical_kinase_COQ8B_mitochondrial____Epinephelus_lanceolatus
XP_032401981_1_atypical_kinase_COQ8B_mitochondriallike____Xiphophorus_hellerii
XP_023206547_1_atypical_kinase_COQ8B_mitochondriallike____Xiphophorus_maculatus
XP_027855496_1_atypical_kinase_COQ8B_mitochondriallike____Xiphophorus_couchianus
XP_029964939_1_atypical_kinase_COQ8B_mitochondriallike_isoform_X2____Salarias_fasciatus
XP_029964938_1_atypical_kinase_COQ8B_mitochondriallike_isoform_X1____Salarias_fasciatus
XP_028276352_1_atypical_kinase_COQ8B_mitochondriallike____Parambassis_ranga
XP_018518191_1_PREDICTED_aarF_domaincontaining_protein_kinase_4like_isoform_X2____Lates_calcarifer
XP_018518190_1_PREDICTED_aarF_domaincontaining_protein_kinase_4like_isoform_X1____Lates_calcarifer
AWO99304_1_putative_aarF_domaincontaining_protein_kinase_4____Scophthalmus_maximus
XP_035478992_1_atypical_kinase_COQ8B_mitochondrial____Scophthalmus_maximus
KAF0030706_1_hypothetical_protein_F2P81_017437____Scophthalmus_maximus
XP_029373245_1_atypical_kinase_COQ8B_mitochondriallike____Echeneis_naucrates
XP_023260956_1_atypical_kinase_COQ8B_mitochondriallike____Seriola_lalandi_dorsalis
XP_022616271_1_atypical_kinase_COQ8B_mitochondriallike____Seriola_dumerili
KAF3693059_1_Atypical_kinase_COQ8B_mitochondrial____Channa_argus
XP_026195701_1_atypical_kinase_COQ8B_mitochondrial____Anabas_testudineus
XP_029026648_1_atypical_kinase_COQ8B_mitochondriallike_isoform_X1____Betta_splendens
XP_029026649_1_atypical_kinase_COQ8B_mitochondriallike_isoform_X2____Betta_splendens
XP_029026650_1_atypical_kinase_COQ8B_mitochondriallike_isoform_X3____Betta_splendens
KAF5171727_1_hypothetical_protein_HST_G00044100____Hippoglossus_stenolepis
XP_035011040_1_atypical_kinase_COQ8B_mitochondrial____Hippoglossus_stenolepis
XP_034460095_1_atypical_kinase_COQ8B_mitochondrial_isoform_X1____Hippoglossus_hippoglossus
XP_034460096_1_atypical_kinase_COQ8B_mitochondrial_isoform_X2____Hippoglossus_hippoglossus
XP_019950894_1_PREDICTED_atypical_kinase_COQ8B_mitochondriallike_isoform_X2____Paralichthys_olivaceus
XP_019950892_1_PREDICTED_atypical_kinase_COQ8B_mitochondriallike_isoform_X1____Paralichthys_olivaceus
CAG06439_1_unnamed_protein_product_partial____Tetraodon_nigroviridis
XP_011618607_2_atypical_kinase_COQ8B_mitochondrial_isoform_X2____Takifugu_rubripes
XP_011618606_2_atypical_kinase_COQ8B_mitochondrial_isoform_X1____Takifugu_rubripes
XP_011618608_2_atypical_kinase_COQ8B_mitochondrial_isoform_X3____Takifugu_rubripes
TWW76703_1_Atypical_kinase_COQ8B_mitochondrial____Takifugu_flavidus
TNN01326_1_hypothetical_protein_fugu_010708____Takifugu_bimaculatus
0.7

CS

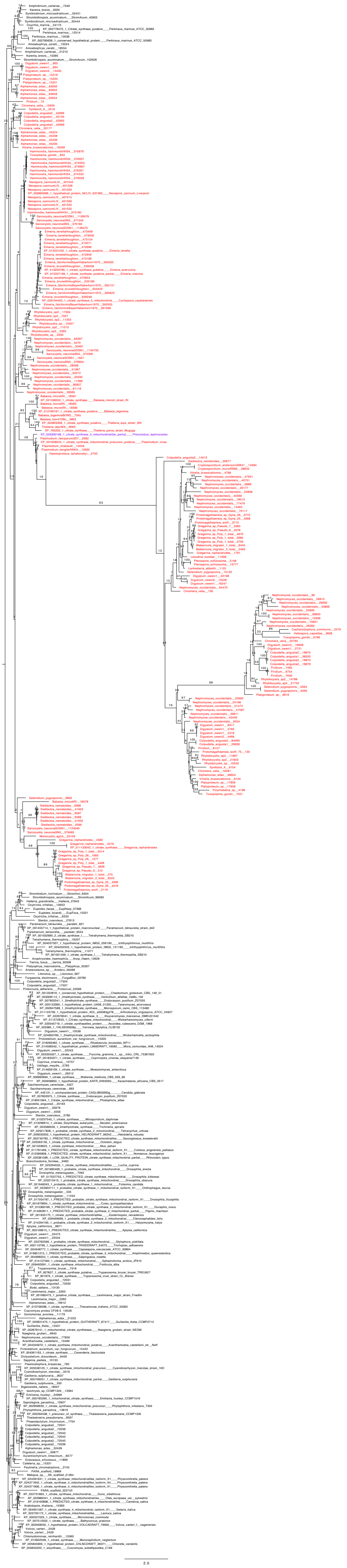

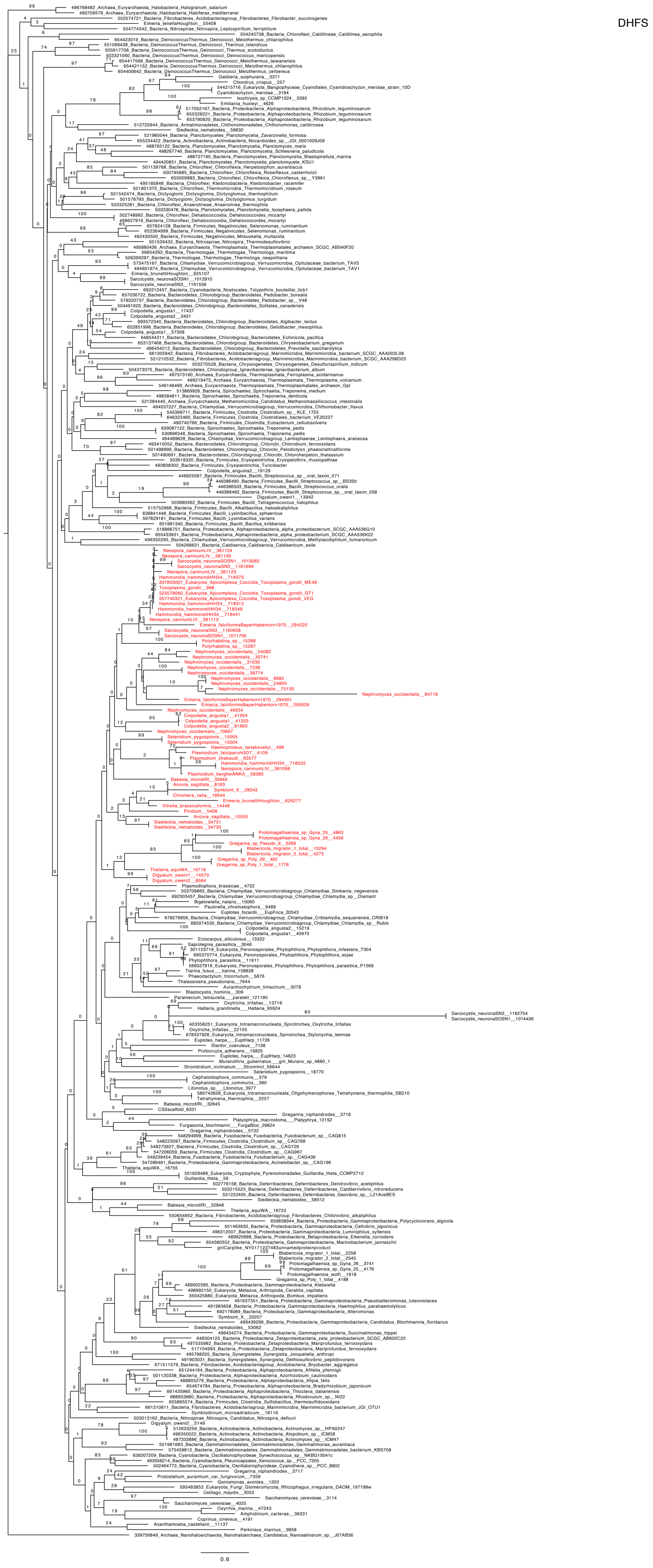
DHFS
0.6

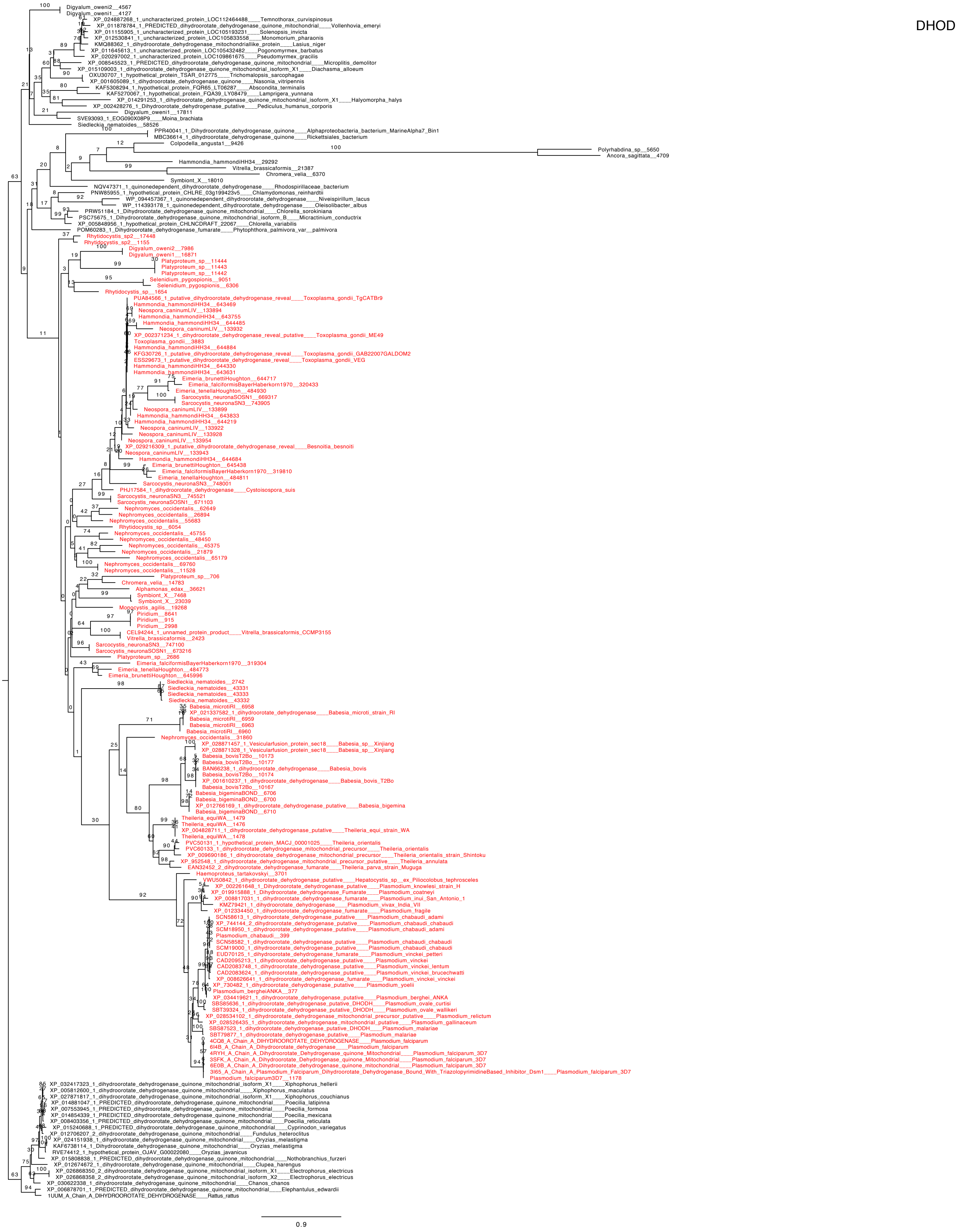
DHOD
Digyalum_oweni2__4567
Digyalum_oweni1__4127
XP_024887268_1_uncharacterized_protein_LOC112464488____Temnothorax_curvispinosus
XP_011878784_1_PREDICTED_dihydroorotate_dehydrogenase_quinone_mitochondrial____Vollenhovia_emeryi
XP_011155905_1_uncharacterized_protein_LOC105193231____Solenopsis_invicta
XP_012530841_1_uncharacterized_protein_LOC105833558____Monomorium_pharaonis
KMQ88362_1_dihydroorotate_dehydrogenase_mitochondriallike_protein____Lasius_niger
XP_011645613_1_uncharacterized_protein_LOC105432482____Pogonomyrmex_barbatus
XP_020297002_1_uncharacterized_protein_LOC109861675____Pseudomyrmex_gracilis
XP_008545523_1_PREDICTED_dihydroorotate_dehydrogenase_quinone_mitochondrial____Microplitis_demolitor
XP_015109003_1_dihydroorotate_dehydrogenase_quinone_mitochondrial_isoform_X1____Diachasma_alloeum
OXU30707_1_hypothetical_protein_TSAR_012775____Trichomalopsis_sarcophagae
XP_001605089_1_dihydroorotate_dehydrogenase_quinone____Nasonia_vitripennis
KAF5308294_1_hypothetical_protein_FQR65_LT06287____Abscondita_terminalis
KAF5270067_1_hypothetical_protein_FQA39_LY08479____Lamprigera_yunnana
XP_014291253_1_dihydroorotate_dehydrogenase_quinone_mitochondrial_isoform_X1____Halyomorpha_halys
XP_002428276_1_Dihydroorotate_dehydrogenase_putative____Pediculus_humanus_corporis
Digyalum_oweni1__17811
SVE93093_1_EOG090X08P9____Moina_brachiata
Siedleckia_nematoides__58526
PPR40041_1_Dihydroorotate_dehydrogenase_quinone____Alphaproteobacteria_bacterium_MarineAlpha7_Bin1
MBC36614_1_dihydroorotate_dehydrogenase_quinone____Rickettsiales_bacterium
Colpodella_angusta1__9426
Polyrhabdina_sp__5650
Ancora_sagittata__4709
Hammondia_hammondiHH34__29292
Vitrella_brassicaformis__21387
Chromera_velia__6370
Symbiont_X__18010
NQV47371_1_quinonedependent_dihydroorotate_dehydrogenase____Rhodospirillaceae_bacterium
PNW85955_1_hypothetical_protein_CHLRE_03g199423v5____Chlamydomonas_reinhardtii
WP_094457367_1_quinonedependent_dihydroorotate_dehydrogenase____Niveispirillum_lacus
WP_114393178_1_quinonedependent_dihydroorotate_dehydrogenase____Oleiolibacter_albus
PRW51184_1_Dihydroorotate_dehydrogenase_quinone_mitochondrial____Chlorella_sorokiniana
PSC75675_1_Dihydroorotate_dehydrogenase_quinone_mitochondrial_isoform_B____Micractinium_conductrix
XP_005848956_1_hypothetical_protein_CHLNCDRAFT_22067____Chlorella_variabilis
POM60283_1_Dihydroorotate_dehydrogenase_fumarate____Phytophthora_palmivora_var__palmivora
Rhytidocystis_sp2__17448
Rhytidocystis_sp2__1155
Digyalum_oweni2__7986
Digyalum_oweni1__16871
Platyproteum_sp__11444
Platyproteum_sp__11443
Platyproteum_sp__11442
Selenidium_pygospionis__9051
Selenidium_pygospionis__6306
Rhytidocystis_sp__1654
PUA84566_1_putative_dihydroorotate_dehydrogenase_reveal____Toxoplasma_gondii_TgCATBr9
Hammondia_hammondiHH34__643469
Neospora_caninumLIV__133894
Hammondia_hammondiHH34__643755
Hammondia_hammondiHH34__644485
Neospora_caninumLIV__133932
XP_002371234_1_dihydroorotate_dehydrogenase_reveal_putative____Toxoplasma_gondii_ME49
Toxoplasma_gondii__3883
Hammondia_hammondiHH34__644884
KFG30726_1_putative_dihydroorotate_dehydrogenase_reveal____Toxoplasma_gondii_GAB22007GALDOM2
ESS29673_1_putative_dihydroorotate_dehydrogenase_reveal____Toxoplasma_gondii_VEG
Hammondia_hammondiHH34__644330
Hammondia_hammondiHH34__643631
Eimeria_brunettiHoughton__644717
Eimeria_falciformisBayerHaberkorn1970__320433
Eimeria_tenellaHoughton__484930
Sarcocystis_neuronaSOSN1__669317
Sarcocystis_neuronaSN3__743905
Neospora_caninumLIV__133899
Hammondia_hammondiHH34__643833
Hammondia_hammondiHH34__644219
Neospora_caninumLIV__133922
Neospora_caninumLIV__133928
Neospora_caninumLIV__133954
XP_029216309_1_putative_dihydroorotate_dehydrogenase_reveal____Besnoitia_besnoiti
Neospora_caninumLIV__133943
Hammondia_hammondiHH34__644684
Eimeria_brunettiHoughton__645438
Eimeria_falciformisBayerHaberkorn1970__319810
Eimeria_tenellaHoughton__484811
Sarcocystis_neuronaSN3__748001
PHJ17584_1_dihydroorotate_dehydrogenase____Cystoisospora_suis
Sarcocystis_neuronaSN3__745521
Sarcocystis_neuronaSOSN1__671103
Nephromyces_occidentalis__62649
Nephromyces_occidentalis__26894
Nephromyces_occidentalis__55683
Rhytidocystis_sp__6054
Nephromyces_occidentalis__45755
Nephromyces_occidentalis__48450
Nephromyces_occidentalis__45375
Nephromyces_occidentalis__21879
Nephromyces_occidentalis__65179
Nephromyces_occidentalis__69760
Nephromyces_occidentalis__11528
Platyproteum_sp__706
Chromera_velia__14783
Alphamonas_edax__36621
Symbiont_X__7468
Symbiont_X__23039
Monocystis_agilis__19268
Piridium__8641
Piridium__915
Piridium__2998
CEL94244_1_unnamed_protein_product____Vitrella_brassicaformis_CCMP3155
Vitrella_brassicaformis__2423
Sarcocystis_neuronaSN3__747100
Sarcocystis_neuronaSOSN1__673216
Platyproteum_sp__2686
Eimeria_falciformisBayerHaberkorn1970__319304
Eimeria_tenellaHoughton__484773
Eimeria_brunettiHoughton__645996
Siedleckia_nematoides__2742
Siedleckia_nematoides__43331
Siedleckia_nematoides__43333
Siedleckia_nematoides__43332
Babesia_microtiRI__6958
XP_021337582_1_dihydroorotate_dehydrogenase____Babesia_microti_strain_RI
Babesia_microtiRI__6959
Babesia_microtiRI__6963
Babesia_microtiRI__6960
Nephromyces_occidentalis__31860
XP_028871457_1_Vesicularfusion_protein_sec18____Babesia_sp__Xinjiang
XP_028871328_1_Vesicularfusion_protein_sec18____Babesia_sp__Xinjiang
Babesia_bovisT2Bo__10173
Babesia_bovisT2Bo__10177
BAN66238_1_dihydroorotate_dehydrogenase____Babesia_bovis
Babesia_bovisT2Bo__10174
XP_001610237_1_dihydroorotate_dehydrogenase____Babesia_bovis_T2Bo
Babesia_bovisT2Bo__10167
Babesia_bigeminaBOND__6706
Babesia_bigeminaBOND__6700
XP_012766169_1_dihydroorotate_dehydrogenase_putative____Babesia_bigemina
Babesia_bigeminaBOND__6710
Theileria_equiWA__1479
Theileria_equiWA__1476
XP_004828711_1_dihydroorotate_dehydrogenase_putative____Theileria_equi_strain_WA
Theileria_equiWA__1478
PVC50131_1_hypothetical_protein_MACJ_00001025____Theileria_orientalis
PVC60133_1_dihydroorotate_dehydrogenase_mitochondrial_precursor____Theileria_orientalis
XP_009690186_1_dihydroorotate_dehydrogenase_mitochondrial_precursor____Theileria_orientalis_strain_Shintoku
XP_952548_1_dihydroorotate_dehydrogenase_mitochondrial_precursor_putative____Theileria_annulata
EAN32452_2_dihydroorotate_dehydrogenase_fumarate____Theileria_parva_strain_Muguga
Haemoproteus_tartakovskyi__3701
VWU50842_1_dihydroorotate_dehydrogenase_putative____Hepatocystis_sp__ex_Piliocolobus_tephrosceles
XP_002261848_1_Dihydroorotate_dehydrogenase_putative____Plasmodium_knowlesi_strain_H
XP_019915888_1_Dihydroorotate_dehydrogenase_Fumarate____Plasmodium_coatneyi
XP_008817031_1_dihydroorotate_dehydrogenase_fumarate____Plasmodium_inui_San_Antonio_1
KMZ79421_1_dihydroorotate_dehydrogenase____Plasmodium_vivax_India_VII
XP_012334450_1_dihydroorotate_dehydrogenase_fumarate____Plasmodium_fragile
SCN58613_1_dihydroorotate_dehydrogenase_putative____Plasmodium_chabaudi_adami
XP_744144_2_dihydroorotate_dehydrogenase_putative____Plasmodium_chabaudi_chabaudi
SCM18950_1_dihydroorotate_dehydrogenase_putative____Plasmodium_chabaudi_adami
Plasmodium_chabaudi__399
SCN58582_1_dihydroorotate_dehydrogenase_putative____Plasmodium_chabaudi_chabaudi
SCM19000_1_dihydroorotate_dehydrogenase_putative____Plasmodium_chabaudi_chabaudi
EUD70125_1_dihydroorotate_dehydrogenase_fumarate____Plasmodium_vinckei_petteri
CAD2095213_1_dihydroorotate_dehydrogenase_putative____Plasmodium_vinckei
CAD2083748_1_dihydroorotate_dehydrogenase_putative____Plasmodium_vinckei_lentum
CAD2083624_1_dihydroorotate_dehydrogenase_putative____Plasmodium_vinckei_brucechwatti
XP_008626641_1_dihydroorotate_dehydrogenase_fumarate____Plasmodium_vinckei_vinckei
XP_730482_1_dihydroorotate_dehydrogenase_putative____Plasmodium_yoelii
Plasmodium_bergheiANKA__377
XP_034419621_1_dihydroorotate_dehydrogenase_putative____Plasmodium_berghei_ANKA
SBS85636_1_dihydroorotate_dehydrogenase_putative_DHODH____Plasmodium_ovale_curtisi
SBT39324_1_dihydroorotate_dehydrogenase_putative_DHODH____Plasmodium_ovale_wallikeri
XP_028534102_1_dihydroorotate_dehydrogenase_mitochondrial_precursor_putative____Plasmodium_relictum
XP_028526435_1_dihydroorotate_dehydrogenase_mitochondrial_putative____Plasmodium_gallinaceum
SBS87523_1_dihydroorotate_dehydrogenase_putative_DHODH____Plasmodium_malariae
SBT79877_1_dihydroorotate_dehydrogenase_putative____Plasmodium_malariae
4CQ8_A_Chain_A_DIHYDROOROTATE_DEHYDROGENASE____Plasmodium_falciparum
6I4B_A_Chain_A_Dihydroorotate_dehydrogenase____Plasmodium_falciparum
4RYH_A_Chain_A_Dihydroorotate_Dehydrogenase_quinone_Mitochondrial____Plasmodium_falciparum_3D7
3SFK_A_Chain_A_Dihydroorotate_Dehydrogenase_quinone_Mitochondrial____Plasmodium_falciparum_3D7
6E0B_A_Chain_A_Dihydroorotate_dehydrogenase_quinone_mitochondrial____Plasmodium_falciparum_3D7
3I65_A_Chain_A_Plasmodium_Falciparum_Dihydroorotate_Dehydrogenase_Bound_With_TriazolopyrimidineBased_Inhibitor_Dsm1____Plasmodium_falciparum_3D7
Plasmodium_falciparum3D7__1178
XP_032417323_1_dihydroorotate_dehydrogenase_quinone_mitochondrial_isoform_X1____Xiphophorus_hellerii
XP_005812600_1_dihydroorotate_dehydrogenase_quinone_mitochondrial____Xiphophorus_maculatus
XP_027871817_1_dihydroorotate_dehydrogenase_quinone_mitochondrial_isoform_X1____Xiphophorus_couchianus
XP_014881047_1_PREDICTED_dihydroorotate_dehydrogenase_quinone_mitochondrial____Poecilia_latipinna
XP_007553945_1_PREDICTED_dihydroorotate_dehydrogenase_quinone_mitochondrial____Poecilia_formosa
XP_014854339_1_PREDICTED_dihydroorotate_dehydrogenase_quinone_mitochondrial____Poecilia_mexicana
XP_008403356_1_PREDICTED_dihydroorotate_dehydrogenase_quinone_mitochondrial____Poecilia_reticulata
XP_015240688_1_PREDICTED_dihydroorotate_dehydrogenase_quinone_mitochondrial____Cyprinodon_variegatus
XP_012706207_2_dihydroorotate_dehydrogenase_quinone_mitochondrial____Fundulus_heteroclitus
XP_024151938_1_dihydroorotate_dehydrogenase_quinone_mitochondrial____Oryzias_melastigma
KAF6738114_1_Dihydroorotate_dehydrogenase_quinone_mitochondrial____Oryzias_melastigma
RVE74412_1_hypothetical_protein_OJAV_G00022080____Oryzias_javanicus
XP_015808838_1_PREDICTED_dihydroorotate_dehydrogenase_quinone_mitochondrial____Nothobranchius_furzeri
XP_012674672_1_dihydroorotate_dehydrogenase_quinone_mitochondrial____Clupea_harengus
XP_026868350_2_dihydroorotate_dehydrogenase_quinone_mitochondrial_isoform_X1____Electrophorus_electricus
XP_026868358_2_dihydroorotate_dehydrogenase_quinone_mitochondrial_isoform_X2____Electrophorus_electricus
XP_030622338_1_dihydroorotate_dehydrogenase_quinone_mitochondrial____Chanos_chanos
XP_006878701_1_PREDICTED_dihydroorotate_dehydrogenase_quinone_mitochondrial____Elephantulus_edwardii
1UUM_A_Chain_A_DIHYDROOROTATE_DEHYDROGENASE____Rattus_rattus
0.9

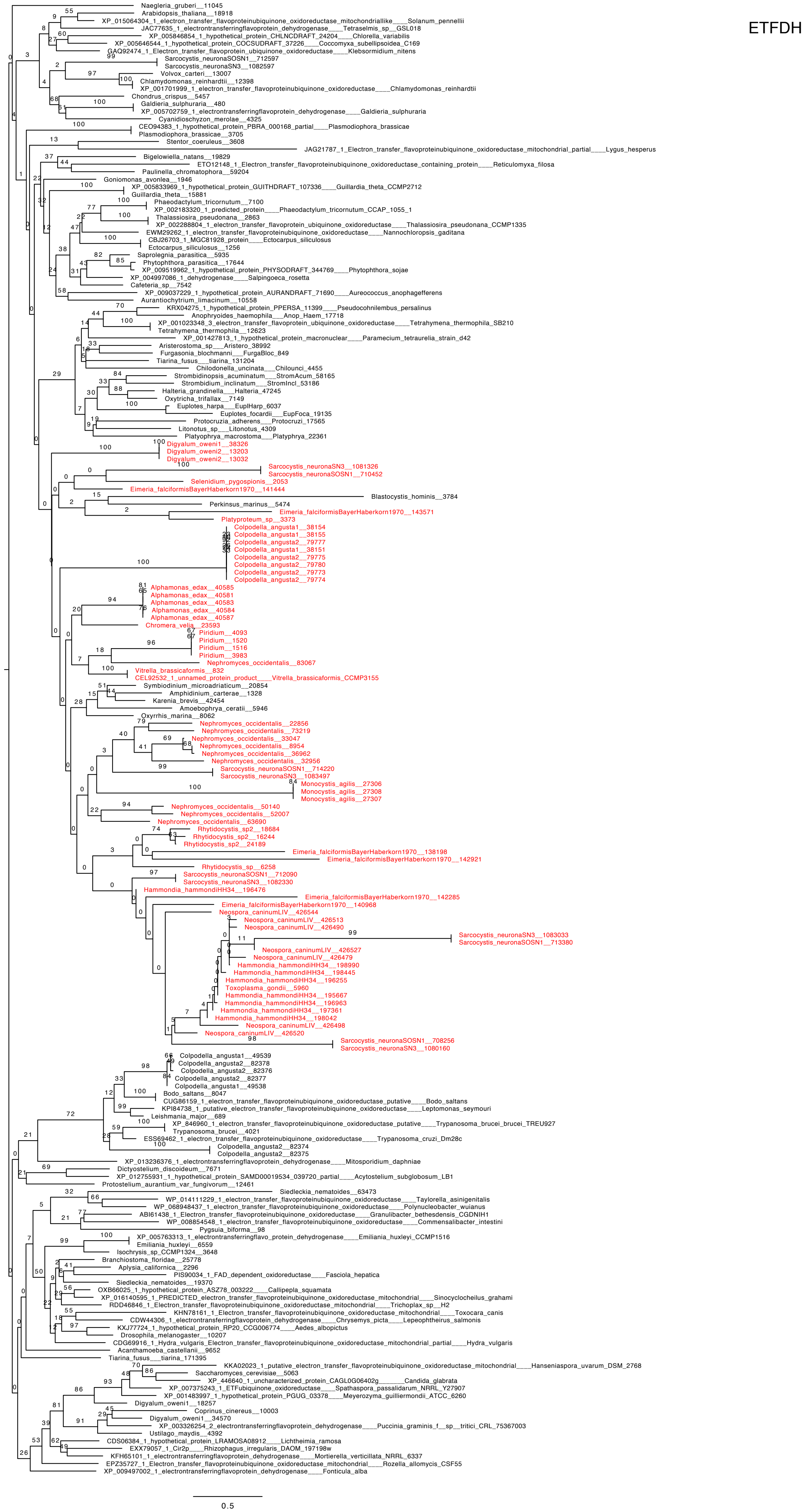

ETFDH
Naegleria_gruberi__11045
Arabidopsis_thaliana__18918
XP_015064304_1_electron_transfer_flavoproteinubiquinone_oxidoreductase_mitochondriallike____Solanum_pennellii
JAC77635_1_electrontransferringflavoprotein_dehydrogenase____Tetraselmis_sp__GSL018
XP_005846854_1_hypothetical_protein_CHLNCDRAFT_24204____Chlorella_variabilis
XP_005646544_1_hypothetical_protein_COCSUDRAFT_37226____Coccomyxa_subellipsoidea_C169
GAQ92474_1_Electron_transfer_flavoprotein_ubiquinone_oxidoreductase____Klebsormidium_nitens
Sarcocystis_neuronaSOSN1__712597
Sarcocystis_neuronaSN3__1082597
Volvox_carteri__13007
Chlamydomonas_reinhardtii__12398
XP_001701999_1_electron_transfer_flavoproteinubiquinone_oxidoreductase____Chlamydomonas_reinhardtii
Chondrus_crispus__5457
Galdieria_sulphuraria__480
XP_005702759_1_electrontransferringflavoprotein_dehydrogenase____Galdieria_sulphuraria
Cyanidioschyzon_merolae__4325
CEO94383_1_hypothetical_protein_PBRA_000168_partial____Plasmodiophora_brassicae
Plasmodiophora_brassicae__3705
Stentor_coeruleus__3608
JAG21787_1_Electron_transfer_flavoproteinubiquinone_oxidoreductase_mitochondrial_partial____Lygus_hesperus
Bigelowiella_natans__19829
ETO12148_1_Electron_transfer_flavoproteinubiquinone_oxidoreductase_containing_protein____Reticulomyxa_filosa
Paulinella_chromatophora__59204
Goniomonas_avonlea__1946
XP_005833969_1_hypothetical_protein_GUITHDRAFT_107336____Guillardia_theta_CCMP2712
Guillardia_theta__15881
Phaeodactylum_tricornutum__7100
XP_002183320_1_predicted_protein____Phaeodactylum_tricornutum_CCAP_1055_1
Thalassiosira_pseudonana__2863
XP_002288804_1_electron_transfer_flavoprotein_ubiquinone_oxidoreductase____Thalassiosira_pseudonana_CCMP1335
EWM29262_1_electron_transfer_flavoproteinubiquinone_oxidoreductase____Nannochloropsis_gaditana
CBJ26703_1_MGC81928_protein____Ectocarpus_siliculosus
Ectocarpus_siliculosus__1256
Saprolegnia_parasitica__5935
Phytophthora_parasitica__17644
XP_009519962_1_hypothetical_protein_PHYSODRAFT_344769____Phytophthora_sojae
XP_004997086_1_dehydrogenase____Salpingoeca_rosetta
Cafeteria_sp__7542
XP_009037229_1_hypothetical_protein_AURANDRAFT_71690____Aureococcus_anophagefferens
Aurantiochytrium_limacinum__10558
KRX04275_1_hypothetical_protein_PPERSA_11399____Pseudocohnilembus_persalinus
Anophryoides_haemophila___Anop_Haem_17718
XP_001023348_3_electron_transfer_flavoprotein_ubiquinone_oxidoreductase____Tetrahymena_thermophila_SB210
Tetrahymena_thermophila__12623
XP_001427813_1_hypothetical_protein_macronuclear____Paramecium_tetraurelia_strain_d42
Aristerostoma_sp___Aristero_38992
Furgasonia_blochmanni___FurgaBloc_849
Tiarina_fusus___tiarina_131204
Chilodonella_uncinata___Chilounci_4455
Strombidinopsis_acuminatum___StromAcum_58165
Strombidium_inclinatum___StromIncl_53186
Halteria_grandinella___Halteria_47245
Oxytricha_trifallax__7149
Euplotes_harpa___EuplHarp_6037
Euplotes_focardii___EupFoca_19135
Protocruzia_adherens___Protocruzi_17565
Litonotus_sp___Litonotus_4309
Platyophrya_macrostoma___Platyphrya_22361
Digyalum_oweni1__38326
Digyalum_oweni2__13203
Digyalum_oweni2__13032
Sarcocystis_neuronaSN3__1081326
Sarcocystis_neuronaSOSN1__710452
Selenidium_pygospionis__2053
Eimeria_falciformisBayerHaberkorn1970__141444
Blastocystis_hominis__3784
Perkinsus_marinus__5474
Eimeria_falciformisBayerHaberkorn1970__143571
Platyproteum_sp__3373
Colpodella_angusta1__38154
Colpodella_angusta1__38155
Colpodella_angusta2__79777
Colpodella_angusta1__38151
Colpodella_angusta2__79775
Colpodella_angusta2__79780
Colpodella_angusta2__79773
Colpodella_angusta2__79774
Alphamonas_edax__40585
Alphamonas_edax__40581
Alphamonas_edax__40583
Alphamonas_edax__40584
Alphamonas_edax__40587
Chromera_velia__23593
Piridium__4093
Piridium__1520
Piridium__1516
Piridium__3983
Nephromyces_occidentalis__83067
Vitrella_brassicaformis__832
CEL92532_1_unnamed_protein_product____Vitrella_brassicaformis_CCMP3155
Symbiodinium_microadriaticum__20854
Amphidinium_carterae__1328
Karenia_brevis__42454
Amoebophrya_ceratii__5946
Oxyrrhis_marina__8062
Nephromyces_occidentalis__22856
Nephromyces_occidentalis__73219
Nephromyces_occidentalis__33047
Nephromyces_occidentalis__8954
Nephromyces_occidentalis__36962
Nephromyces_occidentalis__32956
Sarcocystis_neuronaSOSN1__714220
Sarcocystis_neuronaSN3__1083497
Monocystis_agilis__27306
Monocystis_agilis__27308
Monocystis_agilis__27307
Nephromyces_occidentalis__50140
Nephromyces_occidentalis__52007
Nephromyces_occidentalis__63690
Rhytidocystis_sp2__18684
Rhytidocystis_sp2__16244
Rhytidocystis_sp2__24189
Eimeria_falciformisBayerHaberkorn1970__138198
Eimeria_falciformisBayerHaberkorn1970__142921
Rhytidocystis_sp__6258
Sarcocystis_neuronaSOSN1__712090
Sarcocystis_neuronaSN3__1082330
Hammondia_hammondiHH34__196476
Eimeria_falciformisBayerHaberkorn1970__142285
Eimeria_falciformisBayerHaberkorn1970__140968
Neospora_caninumLIV__426544
Neospora_caninumLIV__426513
Neospora_caninumLIV__426490
Sarcocystis_neuronaSN3__1083033
Sarcocystis_neuronaSOSN1__713380
Neospora_caninumLIV__426527
Neospora_caninumLIV__426479
Hammondia_hammondiHH34__198990
Hammondia_hammondiHH34__198445
Hammondia_hammondiHH34__196255
Toxoplasma_gondii__5960
Hammondia_hammondiHH34__195667
Hammondia_hammondiHH34__196963
Hammondia_hammondiHH34__197361
Hammondia_hammondiHH34__198042
Neospora_caninumLIV__426498
Neospora_caninumLIV__426520
Sarcocystis_neuronaSOSN1__708256
Sarcocystis_neuronaSN3__1080160
Colpodella_angusta1__49539
Colpodella_angusta2__82378
Colpodella_angusta2__82376
Colpodella_angusta2__82377
Colpodella_angusta1__49538
Bodo_saltans__8047
CUG86159_1_electron_transfer_flavoproteinubiquinone_oxidoreductase_putative____Bodo_saltans
KPI84738_1_putative_electron_transfer_flavoproteinubiquinone_oxidoreductase____Leptomonas_seymouri
Leishmania_major__689
XP_846960_1_electron_transfer_flavoproteinubiquinone_oxidoreductase_putative____Trypanosoma_brucei_brucei_TREU927
Trypanosoma_brucei__4021
ESS69462_1_electron_transfer_flavoproteinubiquinone_oxidoreductase____Trypanosoma_cruzi_Dm28c
Colpodella_angusta2__82374
Colpodella_angusta2__82375
XP_013236376_1_electrontransferringflavoprotein_dehydrogenase____Mitosporidium_daphniae
Dictyostelium_discoideum__7671
XP_012755931_1_hypothetical_protein_SAMD00019534_039720_partial____Acytostelium_subglobosum_LB1
Protostelium_aurantium_var_fungivorum__12461
Siedleckia_nematoides__63473
WP_014111229_1_electron_transfer_flavoproteinubiquinone_oxidoreductase____Taylorella_asinigenitalis
WP_068948437_1_electron_transfer_flavoproteinubiquinone_oxidoreductase____Polynucleobacter_wuianus
ABI61438_1_Electron_transfer_flavoproteinubiquinone_oxidoreductase____Granulibacter_bethesdensis_CGDNIH1
WP_008854548_1_electron_transfer_flavoproteinubiquinone_oxidoreductase____Commensalibacter_intestini
Pygsuia_biforma__98
XP_005763313_1_electrontransferringflavo_protein_dehydrogenase____Emiliania_huxleyi_CCMP1516
Emiliania_huxleyi__6559
Isochrysis_sp_CCMP1324__3648
Branchiostoma_floridae__25778
Aplysia_californica__2296
PIS90034_1_FAD_dependent_oxidoreductase____Fasciola_hepatica
Siedleckia_nematoides__19370
OXB66025_1_hypothetical_protein_ASZ78_003222____Callipepla_squamata
XP_016140595_1_PREDICTED_electron_transfer_flavoproteinubiquinone_oxidoreductase_mitochondrial____Sinocyclocheilus_grahami
RDD46846_1_Electron_transfer_flavoproteinubiquinone_oxidoreductase_mitochondrial____Trichoplax_sp__H2
KHN78161_1_Electron_transfer_flavoproteinubiquinone_oxidoreductase_mitochondrial____Toxocara_canis
CDW44306_1_electrontransferringflavoprotein_dehydrogenase____Chrysemys_picta____Lepeophtheirus_salmonis
KXJ77724_1_hypothetical_protein_RP20_CCG006774____Aedes_albopictus
Drosophila_melanogaster__10207
CDG69916_1_Hydra_vulgaris_Electron_transfer_flavoproteinubiquinone_oxidoreductase_mitochondrial_partial____Hydra_vulgaris
Acanthamoeba_castellanii__9652
Tiarina_fusus___tiarina_171395
KKA02023_1_putative_electron_transfer_flavoproteinubiquinone_oxidoreductase_mitochondrial____Hanseniaspora_uvarum_DSM_2768
Saccharomyces_cerevisiae__5063
XP_446640_1_uncharacterized_protein_CAGL0G06402g_______Candida_glabrata
XP_007375243_1_ETFubiquinone_oxidoreductase____Spathaspora_passalidarum_NRRL_Y27907
XP_001483997_1_hypothetical_protein_PGUG_03378____Meyerozyma_guilliermondii_ATCC_6260
Digyalum_oweni1__18257
Coprinus_cinereus__10003
Digyalum_oweni1__34570
XP_003326254_2_electrontransferringflavoprotein_dehydrogenase____Puccinia_graminis_f__sp__tritici_CRL_75367003
Ustilago_maydis__4392
CDS06384_1_hypothetical_protein_LRAMOSA08912____Lichtheimia_ramosa
EXX79057_1_Cir2p____Rhizophagus_irregularis_DAOM_197198w
KFH65101_1_electrontransferringflavoprotein_dehydrogenase____Mortierella_verticillata_NRRL_6337
EPZ35727_1_Electron_transfer_flavoproteinubiquinone_oxidoreductase_mitochondrial____Rozella_allomycis_CSF55
XP_009497002_1_electrontransferringflavoprotein_dehydrogenase____Fonticula_alba
0.5

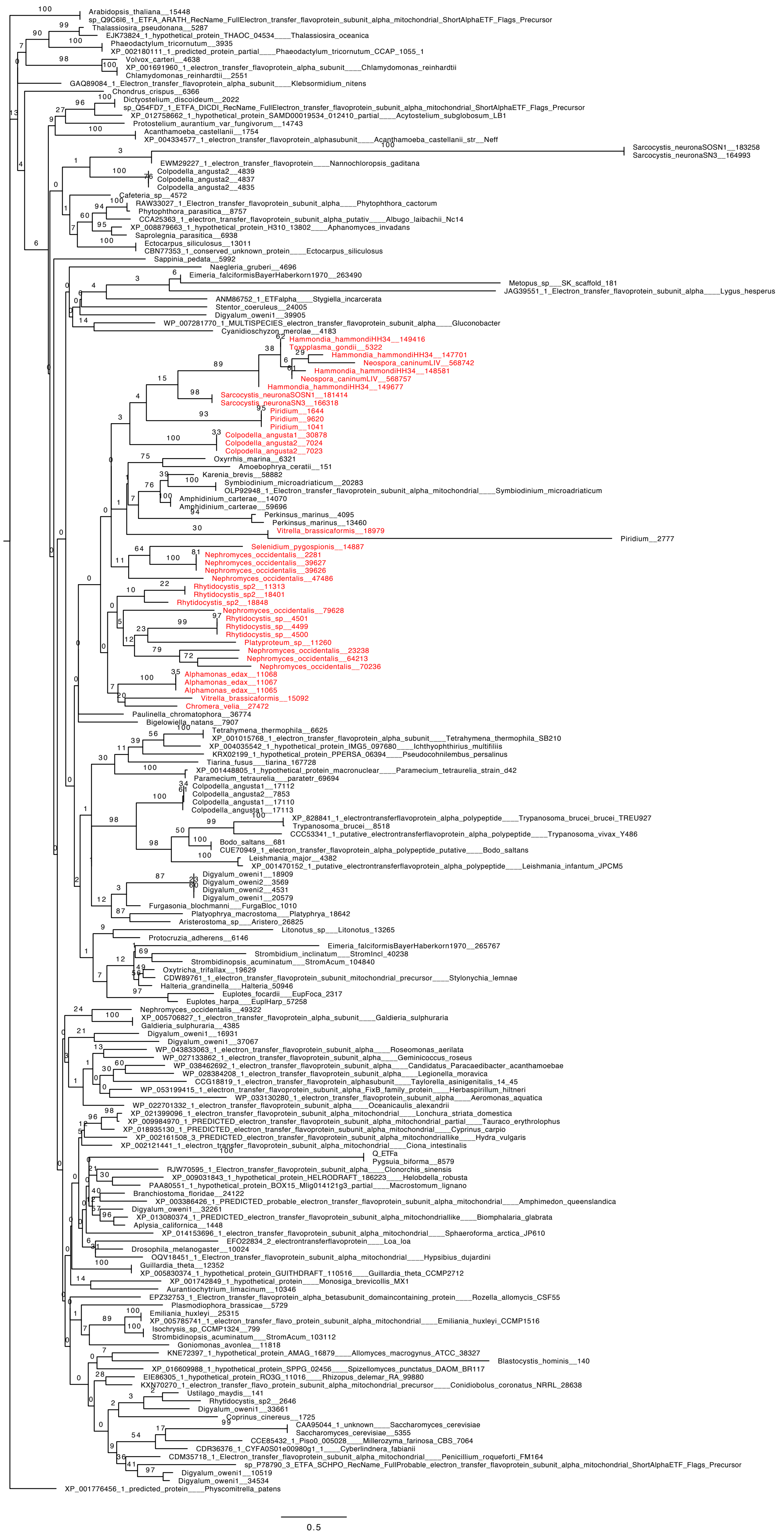
ETFa
Arabidopsis_thaliana__15448
sp_Q9C6I6_1_ETFA_ARATH_RecName_FullElectron_transfer_flavoprotein_subunit_alpha_mitochondrial_ShortAlphaETF_Flags_Precursor
Thalassiosira_pseudonana__5287
EJK73824_1_hypothetical_protein_THAOC_04534____Thalassiosira_oceanica
Phaeodactylum_tricornutum__3935
XP_002180111_1_predicted_protein_partial____Phaeodactylum_tricornutum_CCAP_1055_1
Volvox_carteri__4638
XP_001691960_1_electron_transfer_flavoprotein_alpha_subunit____Chlamydomonas_reinhardtii
Chlamydomonas_reinhardtii__2551
GAQ89084_1_Electron_transfer_flavoprotein_alpha_subunit____Klebsormidium_nitens
Chondrus_crispus__6366
Dictyostelium_discoideum__2022
sp_Q54FD7_1_ETFA_DICDI_RecName_FullElectron_transfer_flavoprotein_subunit_alpha_mitochondrial_ShortAlphaETF_Flags_Precursor
XP_012758662_1_hypothetical_protein_SAMD00019534_012410_partial____Acytostelium_subglobosum_LB1
Protostelium_aurantium_var_fungivorum__14743
Acanthamoeba_castellanii__1754
XP_004334577_1_electron_transfer_flavoprotein_alphasubunit____Acanthamoeba_castellanii_str__Neff
Sarcocystis_neuronaSOSN1__183258
Sarcocystis_neuronaSN3__164993
EWM29227_1_electron_transfer_flavoprotein____Nannochloropsis_gaditana
Colpodella_angusta2__4839
Colpodella_angusta2__4837
Colpodella_angusta2__4835
Cafeteria_sp__4572
RAW33027_1_Electron_transfer_flavoprotein_subunit_alpha____Phytophthora_cactorum
Phytophthora_parasitica__8757
CCA25363_1_electron_transfer_flavoprotein_subunit_alpha_putativ____Albugo_laibachii_Nc14
XP_008879663_1_hypothetical_protein_H310_13802____Aphanomyces_invadans
Saprolegnia_parasitica__6938
Ectocarpus_siliculosus__13011
CBN77353_1_conserved_unknown_protein____Ectocarpus_siliculosus
Sappinia_pedata__5992
Naegleria_gruberi__4696
Eimeria_falciformisBayerHaberkorn1970__263490
Metopus_sp___SK_scaffold_181
JAG39551_1_Electron_transfer_flavoprotein_subunit_alpha____Lygus_hesperus
ANM86752_1_ETFalpha____Stygiella_incarcerata
Stentor_coeruleus__24005
Digyalum_oweni1__39905
WP_007281770_1_MULTISPECIES_electron_transfer_flavoprotein_subunit_alpha____Gluconobacter
Cyanidioschyzon_merolae__4183
Hammondia_hammondiHH34__149416
Toxoplasma_gondii__5322
Hammondia_hammondiHH34__147701
Neospora_caninumLIV__568742
Hammondia_hammondiHH34__148581
Neospora_caninumLIV__568757
Hammondia_hammondiHH34__149677
Sarcocystis_neuronaSOSN1__181414
Sarcocystis_neuronaSN3__166318
Piridium__1644
Piridium__9620
Piridium__1041
Colpodella_angusta1__30878
Colpodella_angusta2__7024
Colpodella_angusta2__7023
Oxyrrhis_marina__6321
Amoebophrya_ceratii__151
Karenia_brevis__58882
Symbiodinium_microadriaticum__20283
OLP92948_1_Electron_transfer_flavoprotein_subunit_alpha_mitochondrial____Symbiodinium_microadriaticum
Amphidinium_carterae__14070
Amphidinium_carterae__59696
Perkinsus_marinus__4095
Perkinsus_marinus__13460
Vitrella_brassicaformis__18979
Piridium__2777
Selenidium_pygospionis__14887
Nephromyces_occidentalis__2281
Nephromyces_occidentalis__39627
Nephromyces_occidentalis__39626
Nephromyces_occidentalis__47486
Rhytidocystis_sp2__11313
Rhytidocystis_sp2__18401
Rhytidocystis_sp2__18848
Nephromyces_occidentalis__79628
Rhytidocystis_sp__4501
Rhytidocystis_sp__4499
Rhytidocystis_sp__4500
Platyproteum_sp__11260
Nephromyces_occidentalis__23238
Nephromyces_occidentalis__64213
Nephromyces_occidentalis__70236
Alphamonas_edax__11068
Alphamonas_edax__11067
Alphamonas_edax__11065
Vitrella_brassicaformis__15092
Chromera_velia__27472
Paulinella_chromatophora__36774
Bigelowiella_natans__7907
Tetrahymena_thermophila__6625
XP_001015768_1_electron_transfer_flavoprotein_alpha_subunit____Tetrahymena_thermophila_SB210
XP_004035542_1_hypothetical_protein_IMG5_097680____Ichthyophthirius_multifiliis
KRX02199_1_hypothetical_protein_PPERSA_06394____Pseudocohnilembus_persalinus
Tiarina_fusus___tiarina_167728
XP_001448805_1_hypothetical_protein_macronuclear____Paramecium_tetraurelia_strain_d42
Paramecium_tetraurelia___paratetr_69694
Colpodella_angusta1__17112
Colpodella_angusta2__7853
Colpodella_angusta1__17110
Colpodella_angusta1__17113
XP_828841_1_electrontransferflavoprotein_alpha_polypeptide____Trypanosoma_brucei_brucei_TREU927
Trypanosoma_brucei__8518
CCC53341_1_putative_electrontransferflavoprotein_alpha_polypeptide____Trypanosoma_vivax_Y486
Bodo_saltans__681
CUE70949_1_electron_transfer_flavoprotein_alpha_polypeptide_putative____Bodo_saltans
Leishmania_major__4382
XP_001470152_1_putative_electrontransferflavoprotein_alpha_polypeptide____Leishmania_infantum_JPCM5
Digyalum_oweni1__18909
Digyalum_oweni2__3569
Digyalum_oweni2__4531
Digyalum_oweni1__20579
Furgasonia_blochmanni___FurgaBloc_1010
Platyophrya_macrostoma___Platyphrya_18642
Aristerostoma_sp___Aristero_26825
Litonotus_sp___Litonotus_13265
Protocruzia_adherens__6146
Eimeria_falciformisBayerHaberkorn1970__265767
Strombidium_inclinatum___StromIncl_40238
Strombidinopsis_acuminatum___StromAcum_104840
Oxytricha_trifallax__19629
CDW89761_1_electron_transfer_flavoprotein_subunit_mitochondrial_precursor____Stylonychia_lemnae
Halteria_grandinella___Halteria_50946
Euplotes_focardii___EupFoca_2317
Euplotes_harpa___EuplHarp_57258
Nephromyces_occidentalis__49322
XP_005706827_1_electron_transfer_flavoprotein_alpha_subunit____Galdieria_sulphuraria
Galdieria_sulphuraria__4385
Digyalum_oweni1__16931
Digyalum_oweni1__37067
WP_043833063_1_electron_transfer_flavoprotein_subunit_alpha____Roseomonas_aerilata
WP_027133862_1_electron_transfer_flavoprotein_subunit_alpha____Geminicoccus_roseus
WP_038462692_1_electron_transfer_flavoprotein_subunit_alpha____Candidatus_Paracaedibacter_acanthamoebae
WP_028384208_1_electron_transfer_flavoprotein_subunit_alpha____Legionella_moravica
CCG18819_1_electron_transfer_flavoprotein_alphasubunit____Taylorella_asinigenitalis_14_45
WP_053199415_1_electron_transfer_flavoprotein_subunit_alpha_FixB_family_protein____Herbaspirillum_hiltneri
WP_033130280_1_electron_transfer_flavoprotein_subunit_alpha____Aeromonas_aquatica
WP_022701332_1_electron_transfer_flavoprotein_subunit_alpha____Oceanicaulis_alexandrii
XP_021399096_1_electron_transfer_flavoprotein_subunit_alpha_mitochondrial____Lonchura_striata_domestica
XP_009984970_1_PREDICTED_electron_transfer_flavoprotein_subunit_alpha_mitochondrial_partial____Tauraco_erythrolophus
XP_018935130_1_PREDICTED_electron_transfer_flavoprotein_subunit_alpha_mitochondrial____Cyprinus_carpio
XP_002161508_3_PREDICTED_electron_transfer_flavoprotein_subunit_alpha_mitochondriallike____Hydra_vulgaris
XP_002121441_1_electron_transfer_flavoprotein_subunit_alpha_mitochondrial____Ciona_intestinalis
Q_ETFa
Pygsuia_biforma__8579
RJW70595_1_Electron_transfer_flavoprotein_subunit_alpha____Clonorchis_sinensis
XP_009031843_1_hypothetical_protein_HELRODRAFT_186223____Helobdella_robusta
PAA80551_1_hypothetical_protein_BOX15_Mlig014121g3_partial____Macrostomum_lignano
Branchiostoma_floridae__24122
XP_003386426_1_PREDICTED_probable_electron_transfer_flavoprotein_subunit_alpha_mitochondrial____Amphimedon_queenslandica
Digyalum_oweni1__32261
XP_013080374_1_PREDICTED_electron_transfer_flavoprotein_subunit_alpha_mitochondriallike____Biomphalaria_glabrata
Aplysia_californica__1448
XP_014153696_1_electron_transfer_flavoprotein_subunit_alpha_mitochondrial____Sphaeroforma_arctica_JP610
EFO22834_2_electrontransferflavoprotein____Loa_loa
Drosophila_melanogaster__10024
OQV18451_1_Electron_transfer_flavoprotein_subunit_alpha_mitochondrial____Hypsibius_dujardini
Guillardia_theta__12352
XP_005830374_1_hypothetical_protein_GUITHDRAFT_110516____Guillardia_theta_CCMP2712
XP_001742849_1_hypothetical_protein____Monosiga_brevicollis_MX1
Aurantiochytrium_limacinum__10346
EPZ32753_1_Electron_transfer_flavoprotein_alpha_betasubunit_domaincontaining_protein____Rozella_allomycis_CSF55
Plasmodiophora_brassicae__5729
Emiliania_huxleyi__25315
XP_005785741_1_electron_transfer_flavo_protein_subunit_alpha_mitochondrial____Emiliania_huxleyi_CCMP1516
Isochrysis_sp_CCMP1324__799
Strombidinopsis_acuminatum___StromAcum_103112
Goniomonas_avonlea__11818
KNE72397_1_hypothetical_protein_AMAG_16879____Allomyces_macrogynus_ATCC_38327
Blastocystis_hominis__140
XP_016609988_1_hypothetical_protein_SPPG_02456____Spizellomyces_punctatus_DAOM_BR117
EIE86305_1_hypothetical_protein_RO3G_11016____Rhizopus_delemar_RA_99880
KXN70270_1_electron_transfer_flavo_protein_subunit_alpha_mitochondrial_precursor____Conidiobolus_coronatus_NRRL_28638
Ustilago_maydis__141
Rhytidocystis_sp2__2646
Digyalum_oweni1__33661
Coprinus_cinereus__1725
CAA95044_1_unknown____Saccharomyces_cerevisiae
Saccharomyces_cerevisiae__5355
CCE85432_1_Piso0_005028____Millerozyma_farinosa_CBS_7064
CDR36376_1_CYFA0S01e00980g1_1____Cyberlindnera_fabianii
CDM35718_1_Electron_transfer_flavoprotein_subunit_alpha_mitochondrial____Penicillium_roqueforti_FM164
sp_P78790_3_ETFA_SCHPO_RecName_FullProbable_electron_transfer_flavoprotein_subunit_alpha_mitochondrial_ShortAlphaETF_Flags_Precursor
Digyalum_oweni1__10519
Digyalum_oweni1__34534
XP_001776456_1_predicted_protein____Physcomitrella_patens
0.5

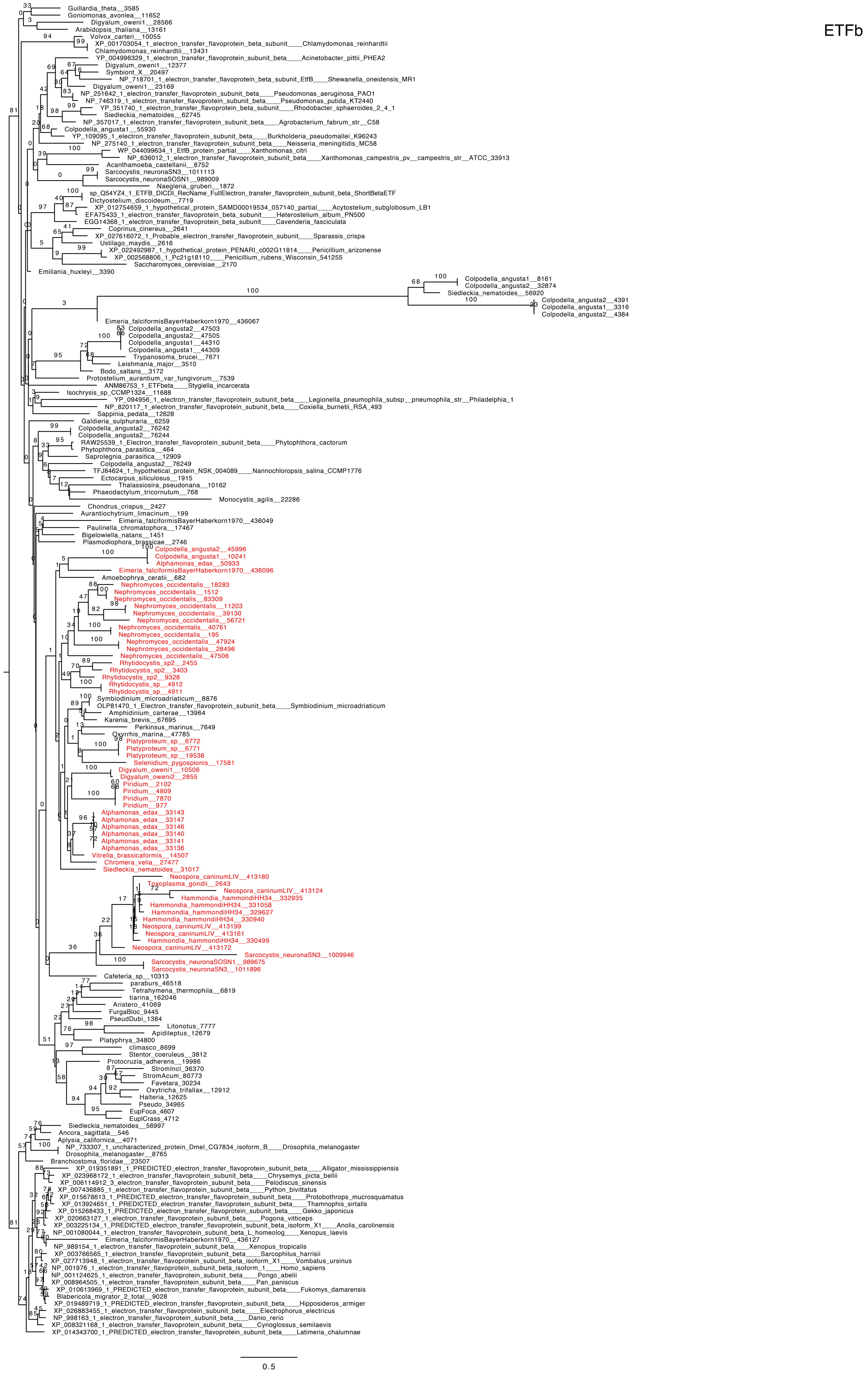
ETFb
0.5

FKBP

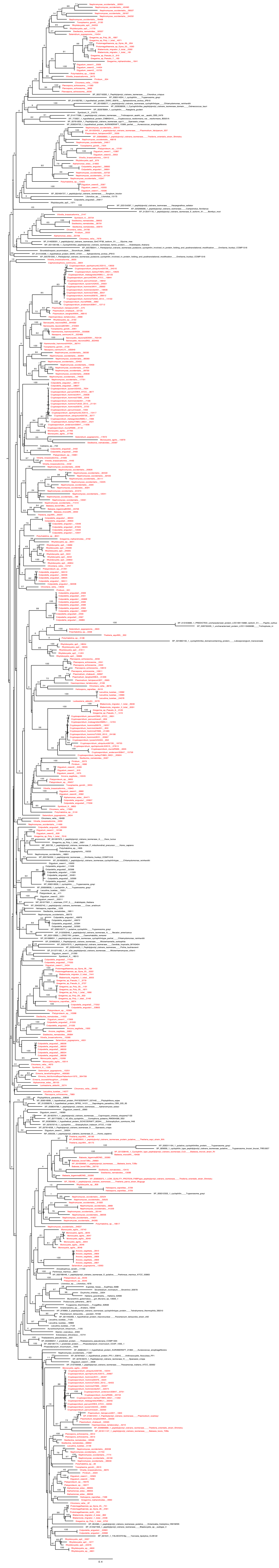

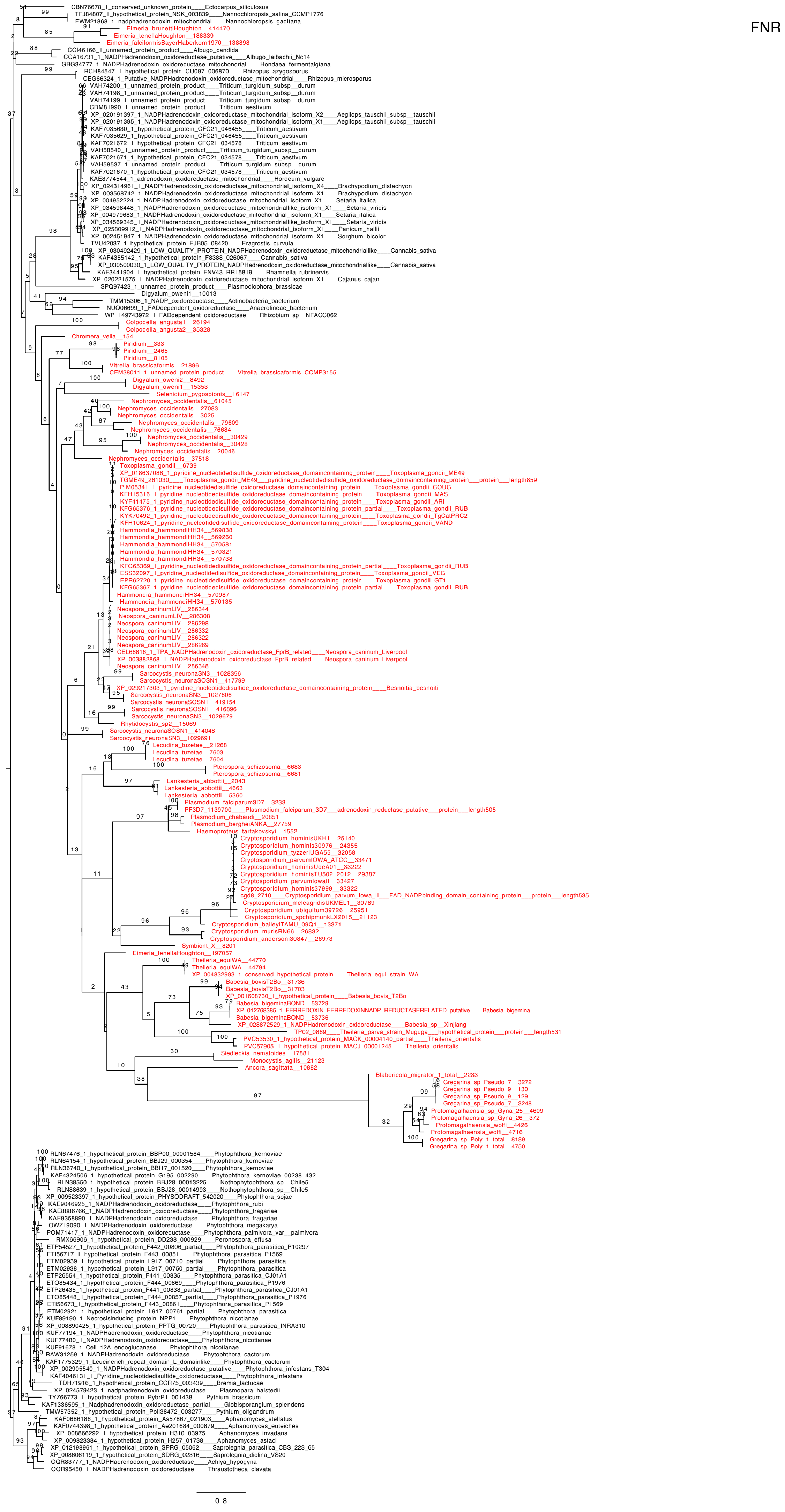
FNR
0.8

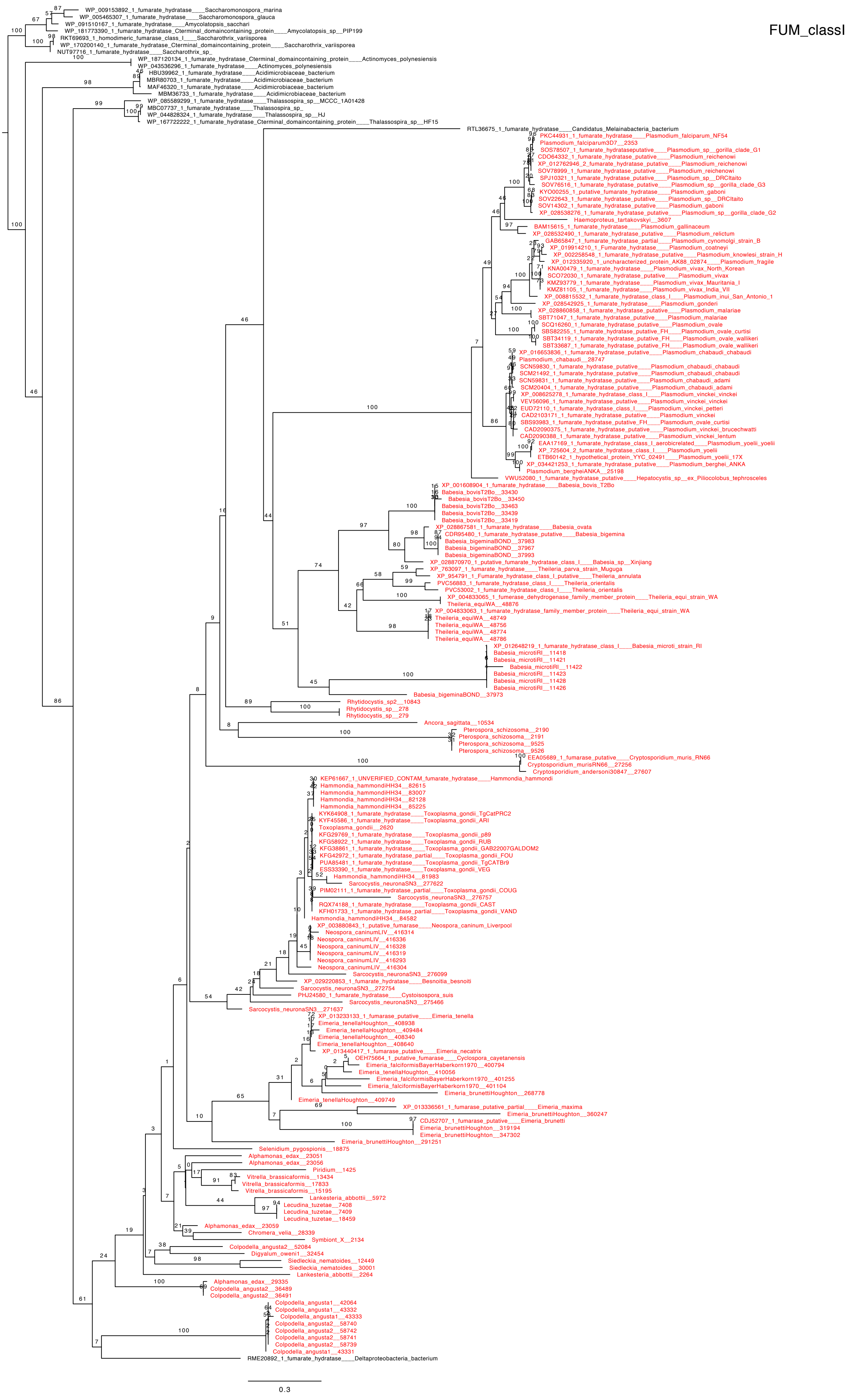

FUM_classI
WP_009153892_1_fumarate_hydratase____Saccharomonospora_marina
WP_005465307_1_fumarate_hydratase____Saccharomonospora_glauca
WP_091510167_1_fumarate_hydratase____Amycolatopsis_sacchari
WP_181773390_1_fumarate_hydratase_Cterminal_domaincontaining_protein____Amycolatopsis_sp__PIP199
RKT69693_1_homodimeric_fumarase_class_I____Saccharothrix_variisporea
WP_170200140_1_fumarate_hydratase_Cterminal_domaincontaining_protein____Saccharothrix_variisporea
NUT97716_1_fumarate_hydratase____Saccharothrix_sp_
WP_187120134_1_fumarate_hydratase_Cterminal_domaincontaining_protein____Actinomyces_polynesiensis
WP_043536296_1_fumarate_hydratase____Actinomyces_polynesiensis
HBU39962_1_fumarate_hydratase____Acidimicrobiaceae_bacterium
MBR80703_1_fumarate_hydratase____Acidimicrobiaceae_bacterium
MAF46320_1_fumarate_hydratase____Acidimicrobiaceae_bacterium
MBM36733_1_fumarate_hydratase____Acidimicrobiaceae_bacterium
WP_085589299_1_fumarate_hydratase____Thalassospira_sp__MCCC_1A01428
MBC07737_1_fumarate_hydratase____Thalassospira_sp_
WP_044828324_1_fumarate_hydratase____Thalassospira_sp__HJ
WP_167722222_1_fumarate_hydratase_Cterminal_domaincontaining_protein____Thalassospira_sp__HF15
RTL36675_1_fumarate_hydratase____Candidatus_Melainabacteria_bacterium
PKC44931_1_fumarate_hydratase____Plasmodium_falciparum_NF54
Plasmodium_falciparum3D7__2353
SOS78507_1_fumarate_hydrataseputative____Plasmodium_sp__gorilla_clade_G1
CDO64332_1_fumarate_hydratase_putative____Plasmodium_reichenowi
XP_012762946_2_fumarate_hydratase_putative____Plasmodium_reichenowi
SOV78999_1_fumarate_hydratase_putative____Plasmodium_reichenowi
SPJ10321_1_fumarate_hydratase_putative____Plasmodium_sp__DRC-Itaito
SOV76516_1_fumarate_hydratase_putative____Plasmodium_sp__gorilla_clade_G3
KYO00255_1_putative_fumarate_hydratase____Plasmodium_gaboni
SOV22643_1_fumarate_hydratase_putative____Plasmodium_sp__DRC-Itaito
SOV14302_1_fumarate_hydratase_putative____Plasmodium_gaboni
XP_028538276_1_fumarate_hydratase_putative____Plasmodium_sp__gorilla_clade_G2
Haemoproteus_tartakovskyi__3607
BAM15615_1_fumarate_hydratase____Plasmodium_gallinaceum
XP_028532490_1_fumarate_hydratase_putative____Plasmodium_relictum
GAB65847_1_fumarate_hydratase_partial____Plasmodium_cynomolgi_strain_B
XP_019914210_1_Fumarate_hydratase____Plasmodium_coatneyi
XP_002258548_1_fumarate_hydratase_putative____Plasmodium_knowlesi_strain_H
XP_012335920_1_uncharacterized_protein_AK88_02874____Plasmodium_fragile
KNA00479_1_fumarate_hydratase____Plasmodium_vivax_North_Korean
SCO72030_1_fumarate_hydratase_putative____Plasmodium_vivax
KMZ93779_1_fumarate_hydratase____Plasmodium_vivax_Mauritania_I
KMZ81105_1_fumarate_hydratase____Plasmodium_vivax_India_VII
XP_008815532_1_fumarate_hydratase_class_I____Plasmodium_inui_San_Antonio_1
XP_028542925_1_fumarate_hydratase____Plasmodium_gonderi
XP_028860858_1_fumarate_hydratase_putative____Plasmodium_malariae
SBT71047_1_fumarate_hydratase_putative____Plasmodium_malariae
SCQ16260_1_fumarate_hydratase_putative____Plasmodium_ovale
SBS82255_1_fumarate_hydratase_putative_FH____Plasmodium_ovale_curtisi
SBT34119_1_fumarate_hydratase_putative_FH____Plasmodium_ovale_wallikeri
SBT33687_1_fumarate_hydratase_putative_FH____Plasmodium_ovale_wallikeri
XP_016653836_1_fumarate_hydratase_putative____Plasmodium_chabaudi_chabaudi
Plasmodium_chabaudi__28747
SCN59830_1_fumarate_hydratase_putative____Plasmodium_chabaudi_chabaudi
SCM21492_1_fumarate_hydratase_putative____Plasmodium_chabaudi_chabaudi
SCN59831_1_fumarate_hydratase_putative____Plasmodium_chabaudi_adami
SCM20404_1_fumarate_hydratase_putative____Plasmodium_chabaudi_adami
XP_008625278_1_fumarate_hydratase_class_I____Plasmodium_vinckei_vinckei
VEV56096_1_fumarate_hydratase_putative____Plasmodium_vinckei_vinckei
EUD72110_1_fumarate_hydratase_class_I____Plasmodium_vinckei_petteri
CAD2103171_1_fumarate_hydratase_putative____Plasmodium_vinckei
SBS93983_1_fumarate_hydratase_putative_FH____Plasmodium_ovale_curtisi
CAD2090375_1_fumarate_hydratase_putative____Plasmodium_vinckei_brucechwatti
CAD2090388_1_fumarate_hydratase_putative____Plasmodium_vinckei_lentum
EAA17169_1_fumarate_hydratase_class_I_aerobicrelated____Plasmodium_yoelii_yoelii
XP_725604_2_fumarate_hydratase_class_I____Plasmodium_yoelii
ETB60142_1_hypothetical_protein_YYC_02491____Plasmodium_yoelii_17X
XP_034421253_1_fumarate_hydratase_putative____Plasmodium_berghei_ANKA
Plasmodium_bergheiANKA__25198
VWU52080_1_fumarate_hydratase_putative____Hepatocystis_sp__ex_Piliocolobus_tephrosceles
XP_001608904_1_fumarate_hydratase____Babesia_bovis_T2Bo
Babesia_bovisT2Bo__33430
Babesia_bovisT2Bo__33450
Babesia_bovisT2Bo__33463
Babesia_bovisT2Bo__33439
Babesia_bovisT2Bo__33419
XP_028867581_1_fumarate_hydratase____Babesia_ovata
CDR95480_1_fumarate_hydratase_putative____Babesia_bigemina
Babesia_bigeminaBOND__37983
Babesia_bigeminaBOND__37967
Babesia_bigeminaBOND__37993
XP_028870970_1_putative_fumarate_hydratase_class_I____Babesia_sp__Xinjiang
XP_763097_1_fumarate_hydratase____Theileria_parva_strain_Muguga
XP_954791_1_Fumarate_hydratase_class_I_putative____Theileria_annulata
PVC56883_1_fumarate_hydratase_class_I____Theileria_orientalis
PVC53002_1_fumarate_hydratase_class_I____Theileria_orientalis
XP_004833065_1_fumerase_dehydrogenase_family_member_protein____Theileria_equi_strain_WA
Theileria_equiWA__48876
XP_004833063_1_fumarate_hydratase_family_member_protein____Theileria_equi_strain_WA
Theileria_equiWA__48749
Theileria_equiWA__48756
Theileria_equiWA__48774
Theileria_equiWA__48786
XP_012648219_1_fumarate_hydratase_class_I____Babesia_microti_strain_RI
Babesia_microtiRI__11418
Babesia_microtiRI__11421
Babesia_microtiRI__11422
Babesia_microtiRI__11423
Babesia_microtiRI__11428
Babesia_microtiRI__11426
Babesia_bigeminaBOND__37973
Rhytidocystis_sp2__10843
Rhytidocystis_sp__278
Rhytidocystis_sp__279
Ancora_sagittata__10534
Pterospora_schizosoma__2190
Pterospora_schizosoma__2191
Pterospora_schizosoma__9525
Pterospora_schizosoma__9526
EEA05689_1_fumarase_putative____Cryptosporidium_muris_RN66
Cryptosporidium_murisRN66__27256
Cryptosporidium_andersoni30847__27607
KEP61667_1_UNVERIFIED_CONTAM_fumarate_hydratase____Hammondia_hammondi
Hammondia_hammondiHH34__82615
Hammondia_hammondiHH34__83007
Hammondia_hammondiHH34__82128
Hammondia_hammondiHH34__85225
KYK64908_1_fumarate_hydratase____Toxoplasma_gondii_TgCatPRC2
KYF45586_1_fumarate_hydratase____Toxoplasma_gondii_ARI
Toxoplasma_gondii__2620
KFG29769_1_fumarate_hydratase____Toxoplasma_gondii_p89
KFG58922_1_fumarate_hydratase____Toxoplasma_gondii_RUB
KFG38861_1_fumarate_hydratase____Toxoplasma_gondii_GAB22007GALDOM2
KFG42972_1_fumarate_hydratase_partial____Toxoplasma_gondii_FOU
PUA85481_1_fumarate_hydratase____Toxoplasma_gondii_TgCATBr9
ESS33390_1_fumarate_hydratase____Toxoplasma_gondii_VEG
Hammondia_hammondiHH34__81983
Sarcocystis_neuronaSN3__277622
PIM02111_1_fumarate_hydratase_partial____Toxoplasma_gondii_COUG
Sarcocystis_neuronaSN3__276757
RQX74188_1_fumarate_hydratase____Toxoplasma_gondii_CAST
KFH01733_1_fumarate_hydratase_partial____Toxoplasma_gondii_VAND
Hammondia_hammondiHH34__84582
XP_003880843_1_putative_fumarase____Neospora_caninum_Liverpool
Neospora_caninumLIV__416314
Neospora_caninumLIV__416336
Neospora_caninumLIV__416328
Neospora_caninumLIV__416319
Neospora_caninumLIV__416293
Neospora_caninumLIV__416304
Sarcocystis_neuronaSN3__276099
XP_029220853_1_fumarate_hydratase____Besnoitia_besnoiti
Sarcocystis_neuronaSN3__272754
PHJ24580_1_fumarate_hydratase____Cystoisospora_suis
Sarcocystis_neuronaSN3__275466
Sarcocystis_neuronaSN3__271637
XP_013233133_1_fumarase_putative____Eimeria_tenella
Eimeria_tenellaHoughton__408938
Eimeria_tenellaHoughton__409484
Eimeria_tenellaHoughton__408340
Eimeria_tenellaHoughton__408640
XP_013440417_1_fumarase_putative____Eimeria_necatrix
OEH75664_1_putative_fumarase____Cyclospora_cayetanensis
Eimeria_falciformisBayerHaberkorn1970__400794
Eimeria_tenellaHoughton__410056
Eimeria_falciformisBayerHaberkorn1970__401255
Eimeria_falciformisBayerHaberkorn1970__401104
Eimeria_brunettiHoughton__268778
Eimeria_tenellaHoughton__409749
XP_013336561_1_fumarase_putative_partial____Eimeria_maxima
Eimeria_brunettiHoughton__360247
CDJ52707_1_fumarase_putative____Eimeria_brunetti
Eimeria_brunettiHoughton__319194
Eimeria_brunettiHoughton__347302
Eimeria_brunettiHoughton__291251
Selenidium_pygospionis__18875
Alphamonas_edax__23051
Alphamonas_edax__23056
Piridium__1425
Vitrella_brassicaformis__13434
Vitrella_brassicaformis__17833
Vitrella_brassicaformis__15195
Lankesteria_abbottii__5972
Lecudina_tuzetae__7408
Lecudina_tuzetae__7409
Lecudina_tuzetae__18459
Alphamonas_edax__23059
Chromera_velia__28339
Symbiont_X__2134
Colpodella_angusta2__52084
Digyalum_oweni1__32454
Siedleckia_nematoides__12449
Siedleckia_nematoides__30001
Lankesteria_abbottii__2264
Alphamonas_edax__29335
Colpodella_angusta2__36489
Colpodella_angusta2__36491
Colpodella_angusta1__42064
Colpodella_angusta1__43332
Colpodella_angusta1__43333
Colpodella_angusta2__58740
Colpodella_angusta2__58742
Colpodella_angusta2__58741
Colpodella_angusta2__58739
Colpodella_angusta1__43331
RME20892_1_fumarate_hydratase____Deltaproteobacteria_bacterium
0.3

FUM_classII

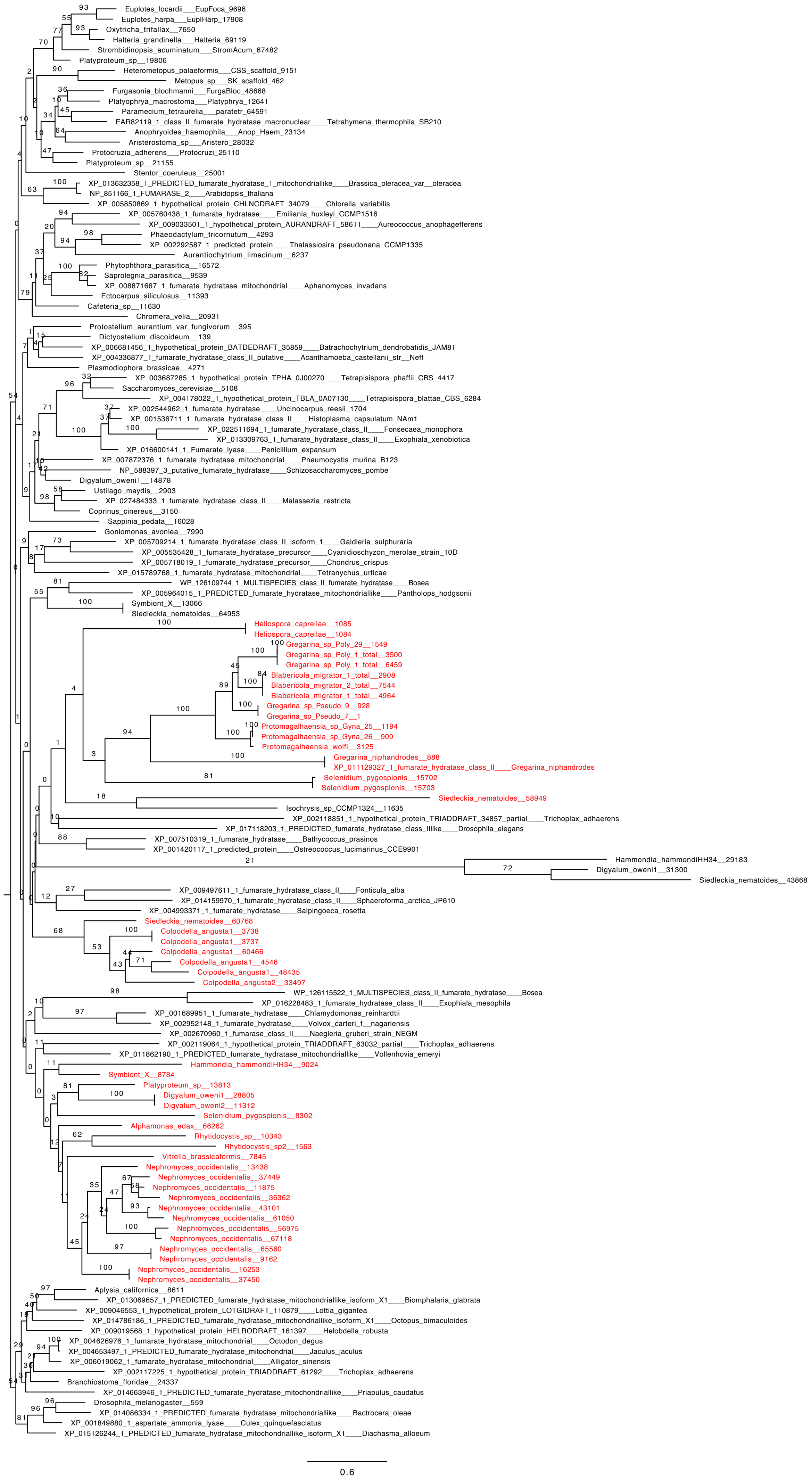
Euplotes_focardii___EupFoca_9696
Euplotes_harpa___EuplHarp_17908
Oxytricha_trifallax__7650
Halteria_grandinella___Halteria_69119
Strombidinopsis_acuminatum___StromAcum_67482
Platyproteum_sp__19806
Heterometopus_palaeformis___CSS_scaffold_9151
Metopus_sp___SK_scaffold_462
Furgasonia_blochmanni___FurgaBloc_48668
Platyophrya_macrostoma___Platyphrya_12641
Paramecium_tetraurelia___paratetr_64591
EAR82119_1_class_II_fumarate_hydratase_macronuclear____Tetrahymena_thermophila_SB210
Anophryoides_haemophila___Anop_Haem_23134
Aristerostoma_sp___Aristero_28032
Protocruzia_adherens___Protocruzi_25110
Platyproteum_sp__21155
Stentor_coeruleus__25001
XP_013632358_1_PREDICTED_fumarate_hydratase_1_mitochondriallike____Brassica_oleracea_var__oleracea
NP_851166_1_FUMARASE_2____Arabidopsis_thaliana
XP_005850869_1_hypothetical_protein_CHLNCDRAFT_34079____Chlorella_variabilis
XP_005760438_1_fumarate_hydratase____Emiliania_huxleyi_CCMP1516
XP_009033501_1_hypothetical_protein_AURANDRAFT_58611____Aureococcus_anophagefferens
Phaeodactylum_tricornutum__4293
XP_002292587_1_predicted_protein____Thalassiosira_pseudonana_CCMP1335
Aurantiochytrium_limacinum__6237
Phytophthora_parasitica__16572
Saprolegnia_parasitica__9539
XP_008871667_1_fumarate_hydratase_mitochondrial____Aphanomyces_invadans
Ectocarpus_siliculosus__11393
Cafeteria_sp__11630
Chromera_velia__20931
Protostelium_aurantium_var_fungivorum__395
Dictyostelium_discoideum__139
XP_006681456_1_hypothetical_protein_BATDEDRAFT_35859____Batrachochytrium_dendrobatidis_JAM81
XP_004336877_1_fumarate_hydratase_class_II_putative____Acanthamoeba_castellanii_str__Neff
Plasmodiophora_brassicae__4271
XP_003687285_1_hypothetical_protein_TPHA_0J00270____Tetrapisispora_phaffii_CBS_4417
Saccharomyces_cerevisiae__5108
XP_004178022_1_hypothetical_protein_TBLA_0A07130____Tetrapisispora_blattae_CBS_6284
XP_002544962_1_fumarate_hydratase____Uncinocarpus_reesii_1704
XP_001536711_1_fumarate_hydratase_class_II____Histoplasma_capsulatum_NAm1
XP_022511694_1_fumarate_hydratase_class_II____Fonsecaea_monophora
XP_013309763_1_fumarate_hydratase_class_II____Exophiala_xenobiotica
XP_016600141_1_Fumarate_lyase____Penicillium_expansum
XP_007872376_1_fumarate_hydratase_mitochondrial____Pneumocystis_murina_B123
NP_588397_3_putative_fumarate_hydratase____Schizosaccharomyces_pombe
Digyalum_oweni1__14878
Ustilago_maydis__2903
XP_027484333_1_fumarate_hydratase_class_II____Malassezia_restricta
Coprinus_cinereus__3150
Sappinia_pedata__16028
Goniomonas_avonlea__7990
XP_005709214_1_fumarate_hydratase_class_II_isoform_1____Galdieria_sulphuraria
XP_005535428_1_fumarate_hydratase_precursor____Cyanidioschyzon_merolae_strain_10D
XP_005718019_1_fumarate_hydratase_precursor____Chondrus_crispus
XP_015789768_1_fumarate_hydratase_mitochondrial____Tetranychus_urticae
WP_126109744_1_MULTISPECIES_class_II_fumarate_hydratase____Bosea
XP_005964015_1_PREDICTED_fumarate_hydratase_mitochondriallike____Pantholops_hodgsonii
Symbiont_X__13066
Siedleckia_nematoides__64953
Heliospora_caprellae__1085
Heliospora_caprellae__1084
Gregarina_sp_Poly_29__1549
Gregarina_sp_Poly_1_total__3500
Gregarina_sp_Poly_1_total__6459
Blabericola_migrator_1_total__2908
Blabericola_migrator_2_total__7544
Blabericola_migrator_1_total__4964
Gregarina_sp_Pseudo_9__928
Gregarina_sp_Pseudo_7__1
Protomagalhaensia_sp_Gyna_25__1194
Protomagalhaensia_sp_Gyna_26__909
Protomagalhaensia_wolfi__3125
Gregarina_niphandrodes__888
XP_011129327_1_fumarate_hydratase_class_II____Gregarina_niphandrodes
Selenidium_pygospionis__15702
Selenidium_pygospionis__15703
Siedleckia_nematoides__58949
Isochrysis_sp_CCMP1324__11635
XP_002118851_1_hypothetical_protein_TRIADDRAFT_34857_partial____Trichoplax_adhaerens
XP_017118203_1_PREDICTED_fumarate_hydratase_class_IIlike____Drosophila_elegans
XP_007510319_1_fumarate_hydratase____Bathycoccus_prasinos
XP_001420117_1_predicted_protein____Ostreococcus_lucimarinus_CCE9901
Hammondia_hammondiHH34__29183
Digyalum_oweni1__31300
Siedleckia_nematoides__43868
XP_009497611_1_fumarate_hydratase_class_II____Fonticula_alba
XP_014159970_1_fumarate_hydratase_class_II____Sphaeroforma_arctica_JP610
XP_004993371_1_fumarate_hydratase____Salpingoeca_rosetta
Siedleckia_nematoides__60768
Colpodella_angusta1__3738
Colpodella_angusta1__3737
Colpodella_angusta1__60466
Colpodella_angusta1__4546
Colpodella_angusta1__48435
Colpodella_angusta2__33497
WP_126115522_1_MULTISPECIES_class_II_fumarate_hydratase____Bosea
XP_016228483_1_fumarate_hydratase_class_II____Exophiala_mesophila
XP_001689951_1_fumarate_hydratase____Chlamydomonas_reinhardtii
XP_002952148_1_fumarate_hydratase____Volvox_carteri_f__nagariensis
XP_002670960_1_fumarase_class_II____Naegleria_gruberi_strain_NEGM
XP_002119064_1_hypothetical_protein_TRIADDRAFT_63032_partial____Trichoplax_adhaerens
XP_011862190_1_PREDICTED_fumarate_hydratase_mitochondriallike____Vollenhovia_emeryi
Hammondia_hammondiHH34__9024
Symbiont_X__8784
Platyproteum_sp__13813
Digyalum_oweni1__28805
Digyalum_oweni2__11312
Selenidium_pygospionis__8302
Alphamonas_edax__66262
Rhytidocystis_sp__10343
Rhytidocystis_sp2__1563
Vitrella_brassicaformis__7845
Nephromyces_occidentalis__13438
Nephromyces_occidentalis__37449
Nephromyces_occidentalis__11875
Nephromyces_occidentalis__36362
Nephromyces_occidentalis__43101
Nephromyces_occidentalis__61050
Nephromyces_occidentalis__56975
Nephromyces_occidentalis__67118
Nephromyces_occidentalis__65560
Nephromyces_occidentalis__9162
Nephromyces_occidentalis__16253
Nephromyces_occidentalis__37450
Aplysia_californica__8611
XP_013069657_1_PREDICTED_fumarate_hydratase_mitochondriallike_isoform_X1____Biomphalaria_glabrata
XP_009046553_1_hypothetical_protein_LOTGIDRAFT_110879____Lottia_gigantea
XP_014786186_1_PREDICTED_fumarate_hydratase_mitochondriallike_isoform_X1____Octopus_bimaculoides
XP_009019568_1_hypothetical_protein_HELRODRAFT_161397____Helobdella_robusta
XP_004626976_1_fumarate_hydratase_mitochondrial____Octodon_degus
XP_004653497_1_PREDICTED_fumarate_hydratase_mitochondrial____Jaculus_jaculus
XP_006019062_1_fumarate_hydratase_mitochondrial____Alligator_sinensis
XP_002117225_1_hypothetical_protein_TRIADDRAFT_61292____Trichoplax_adhaerens
Branchiostoma_floridae__24337
XP_014663946_1_PREDICTED_fumarate_hydratase_mitochondriallike____Priapulus_caudatus
Drosophila_melanogaster__559
XP_014086334_1_PREDICTED_fumarate_hydratase_mitochondriallike____Bactrocera_oleae
XP_001849880_1_aspartate_ammonia_lyase____Culex_quinquefasciatus
XP_015126244_1_PREDICTED_fumarate_hydratase_mitochondriallike_isoform_X1____Diachasma_alloeum
0.6

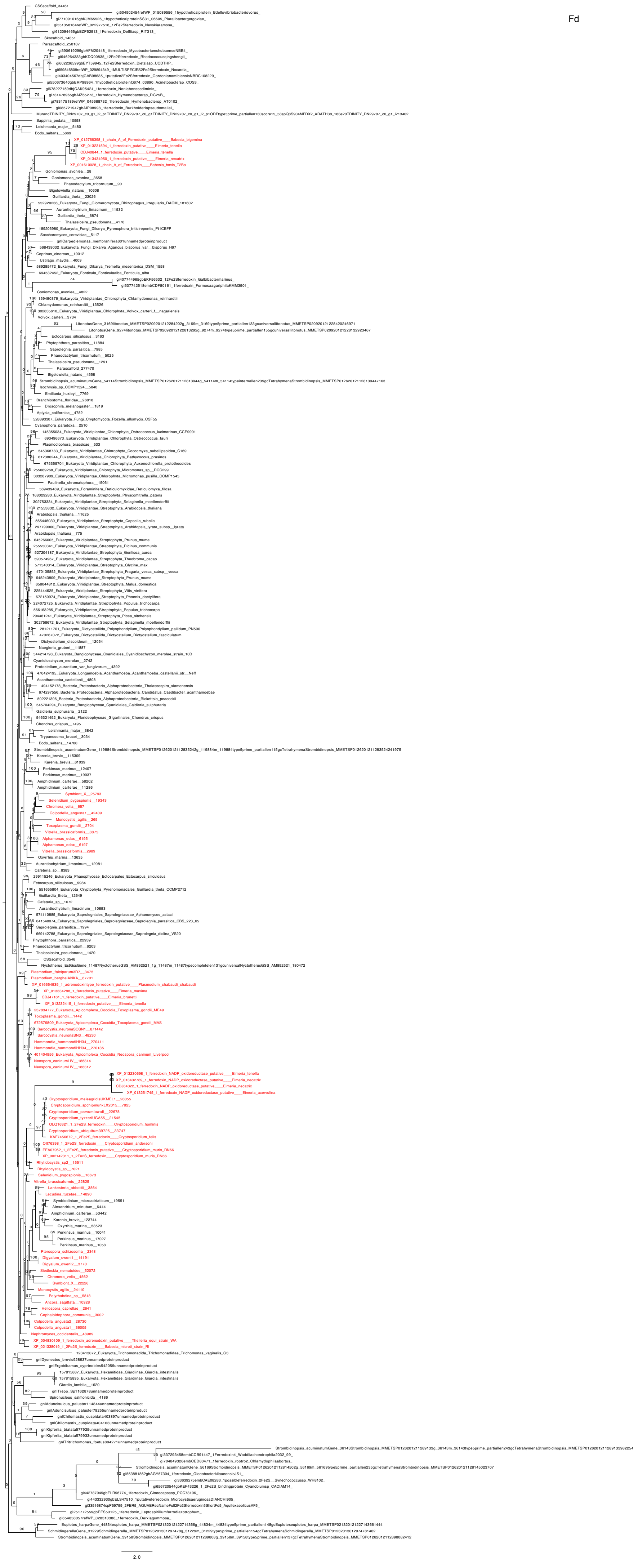
Fd
2.0

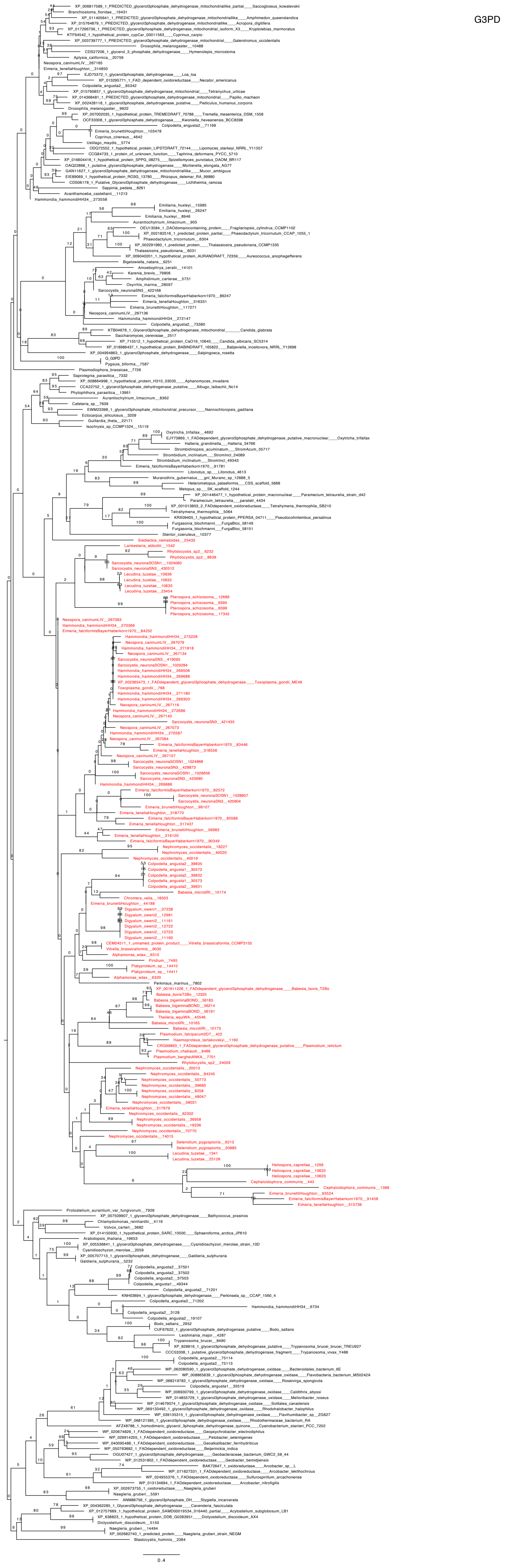
G3PD
0.4

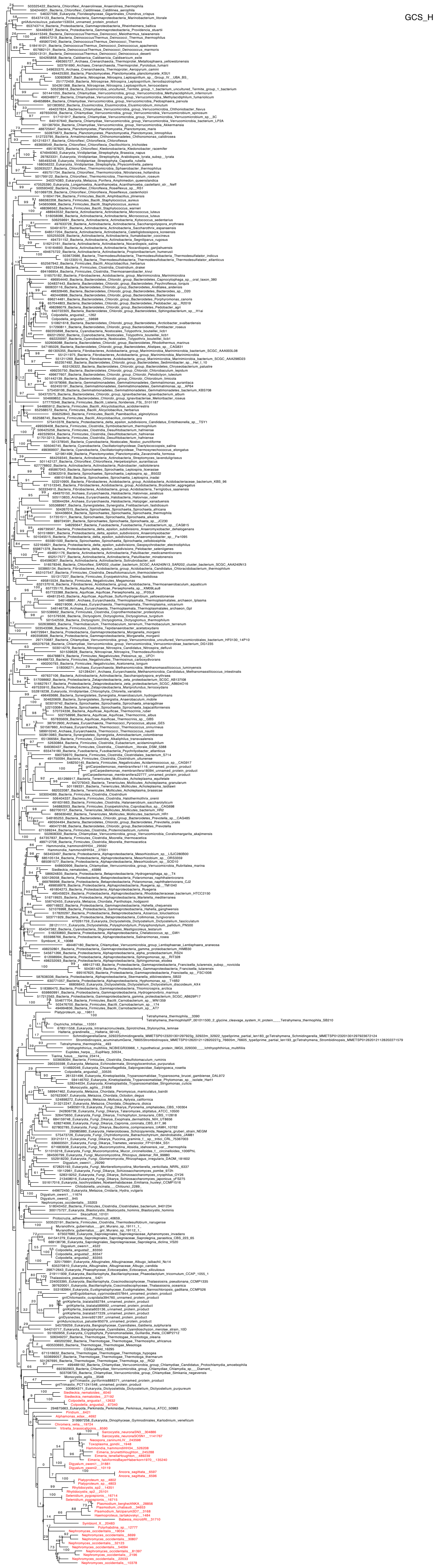
GCS_H
0.5

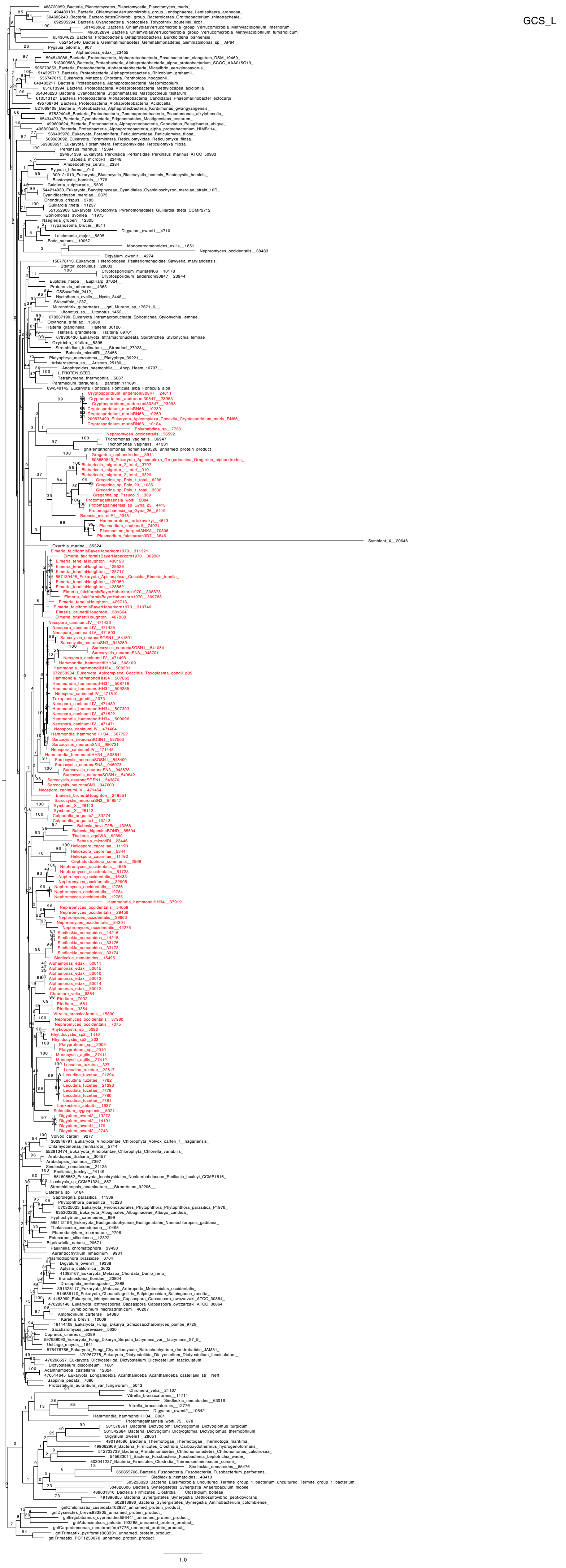
GCS_L
1.0

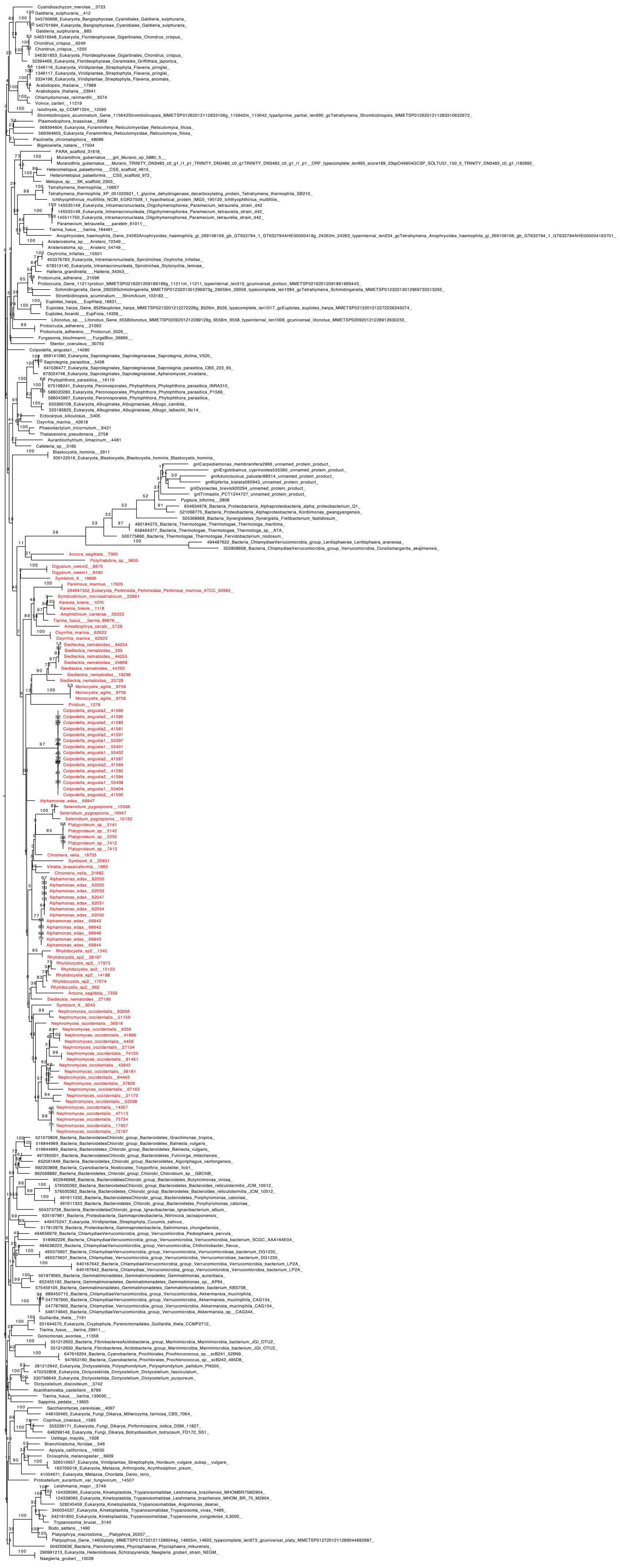

GCS_P

GCS_T

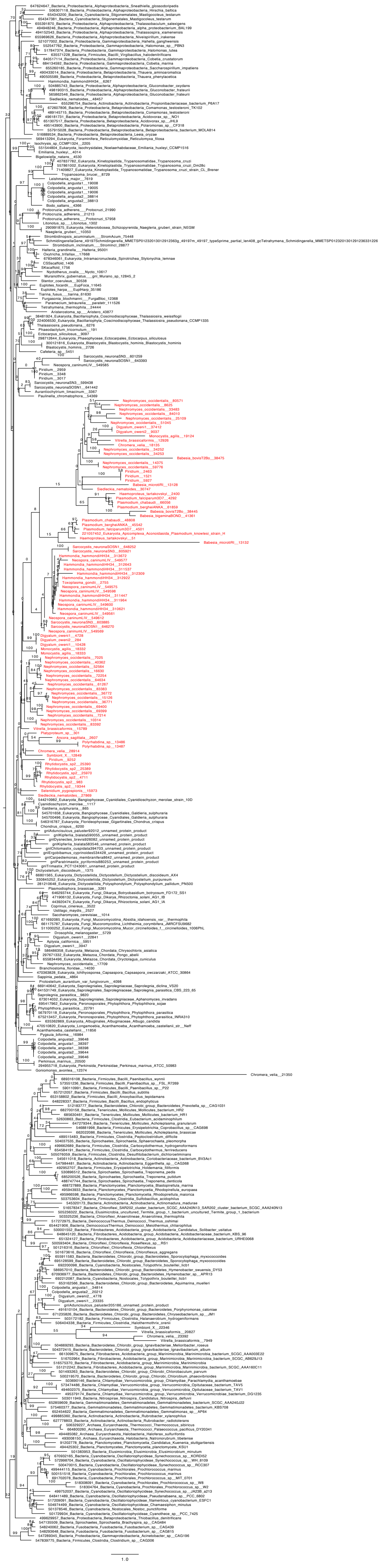

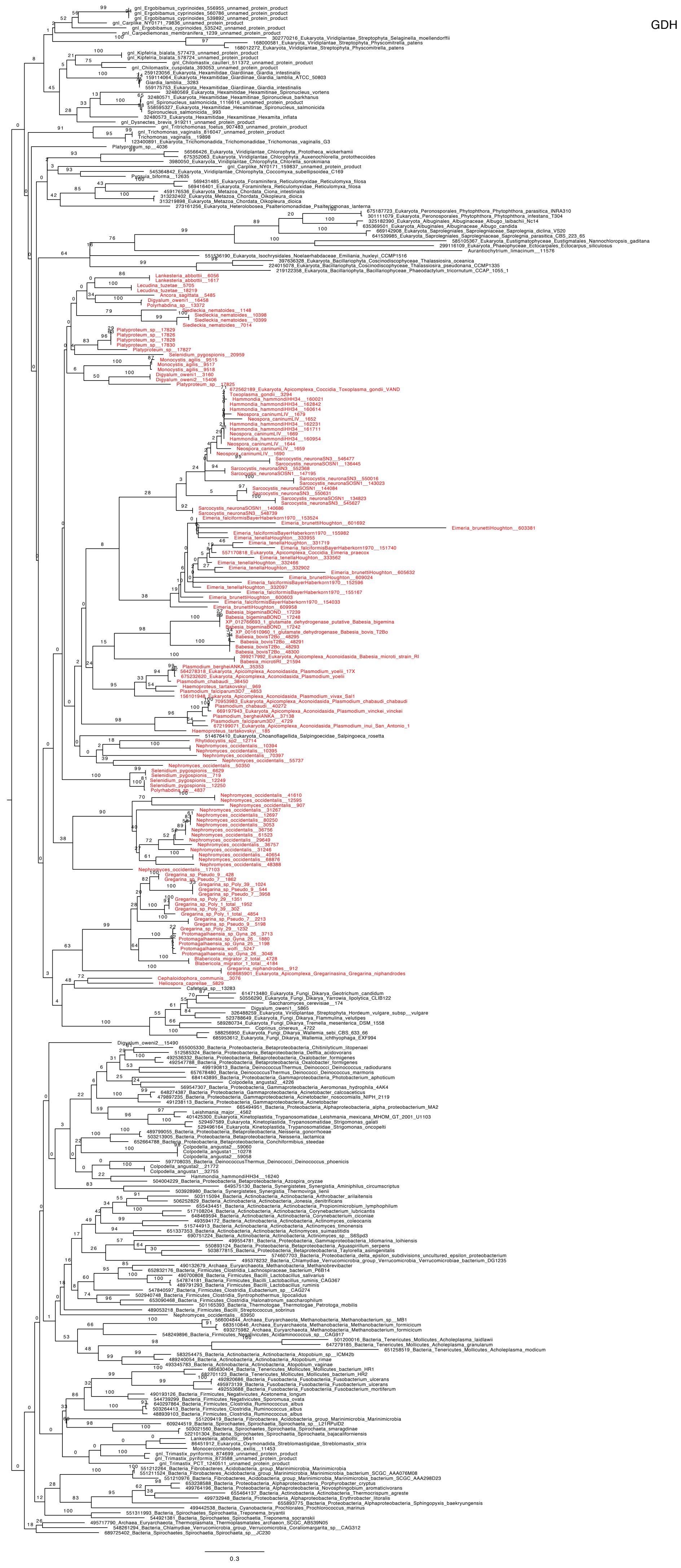
GDH
0.3

GDH2

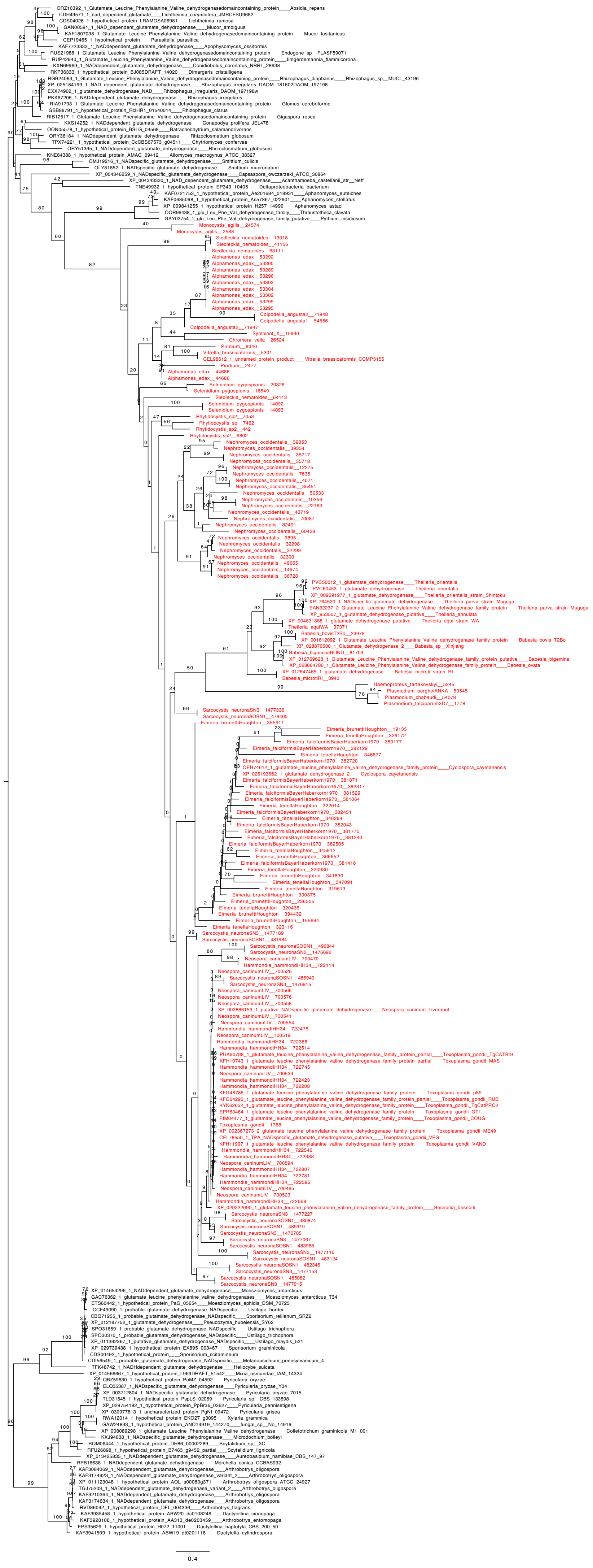

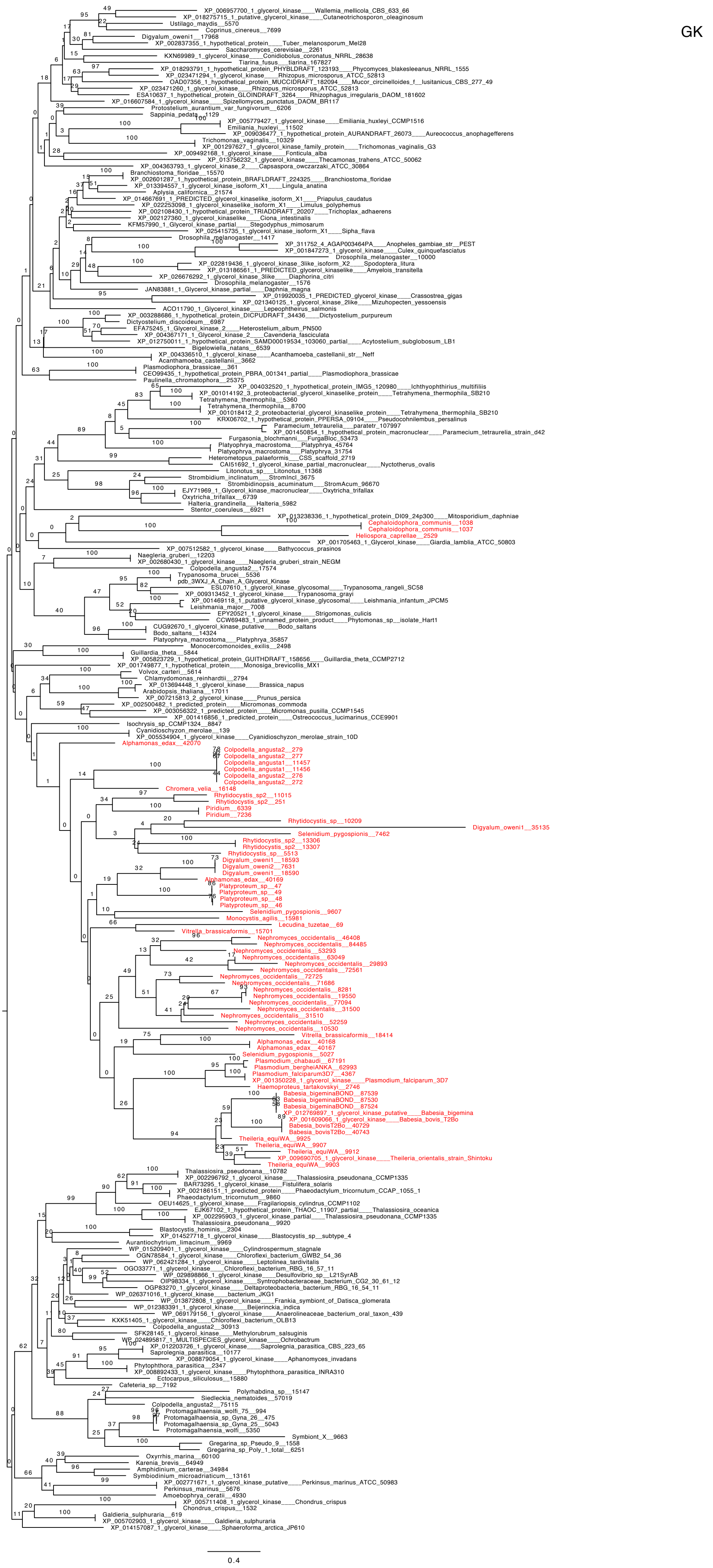
GK
0.4

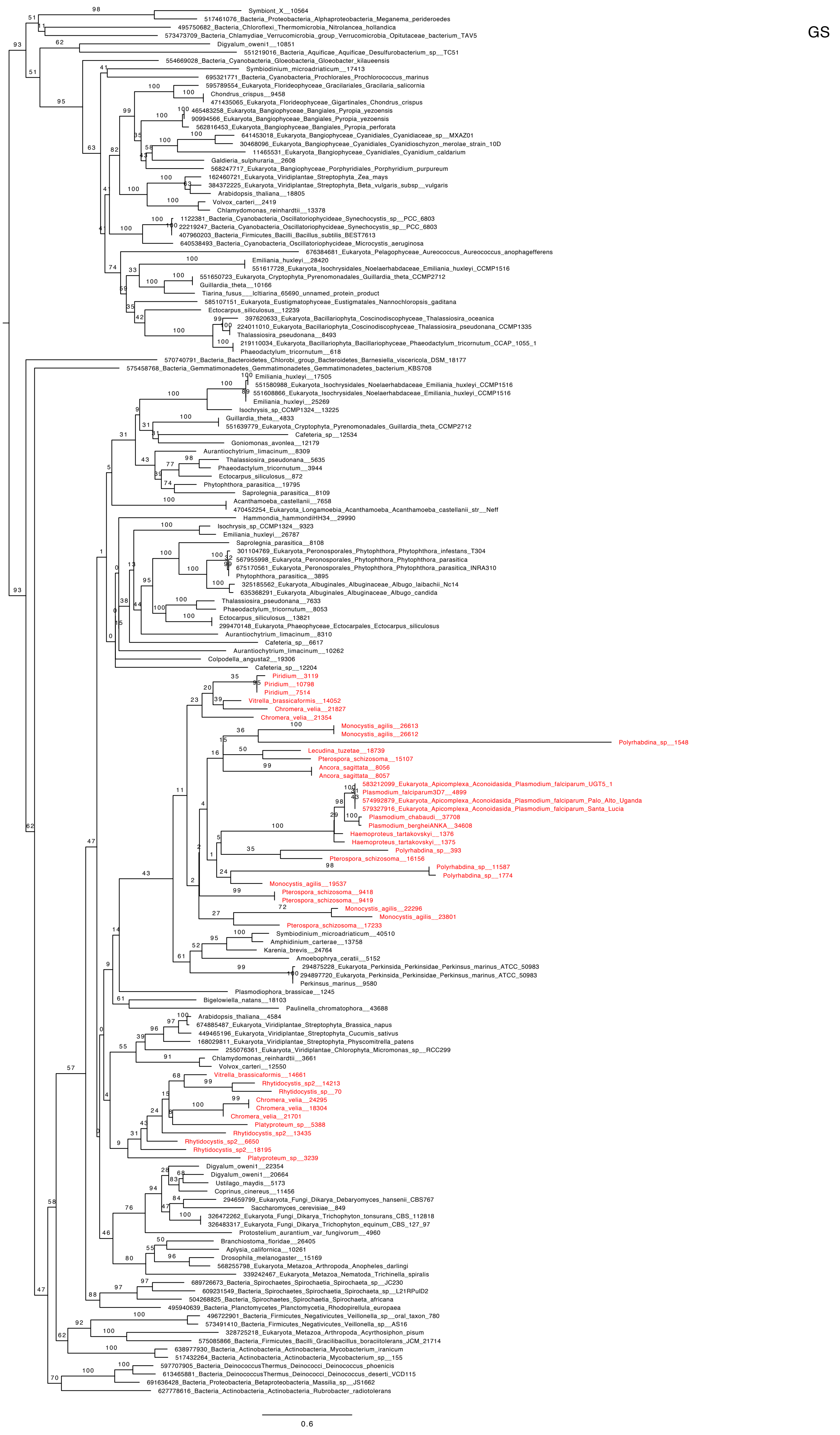

GS
0.6

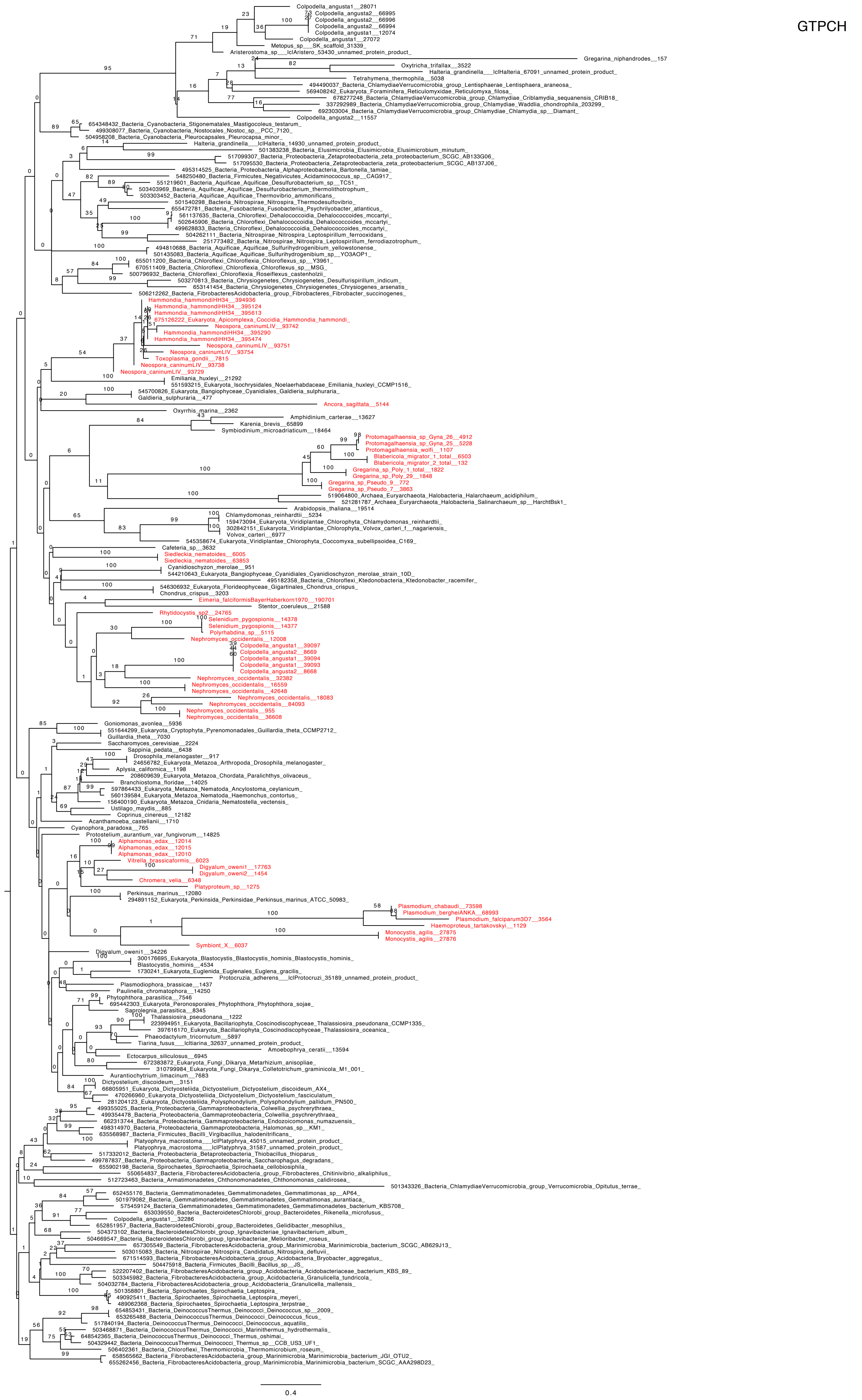
GTPCH
0.4

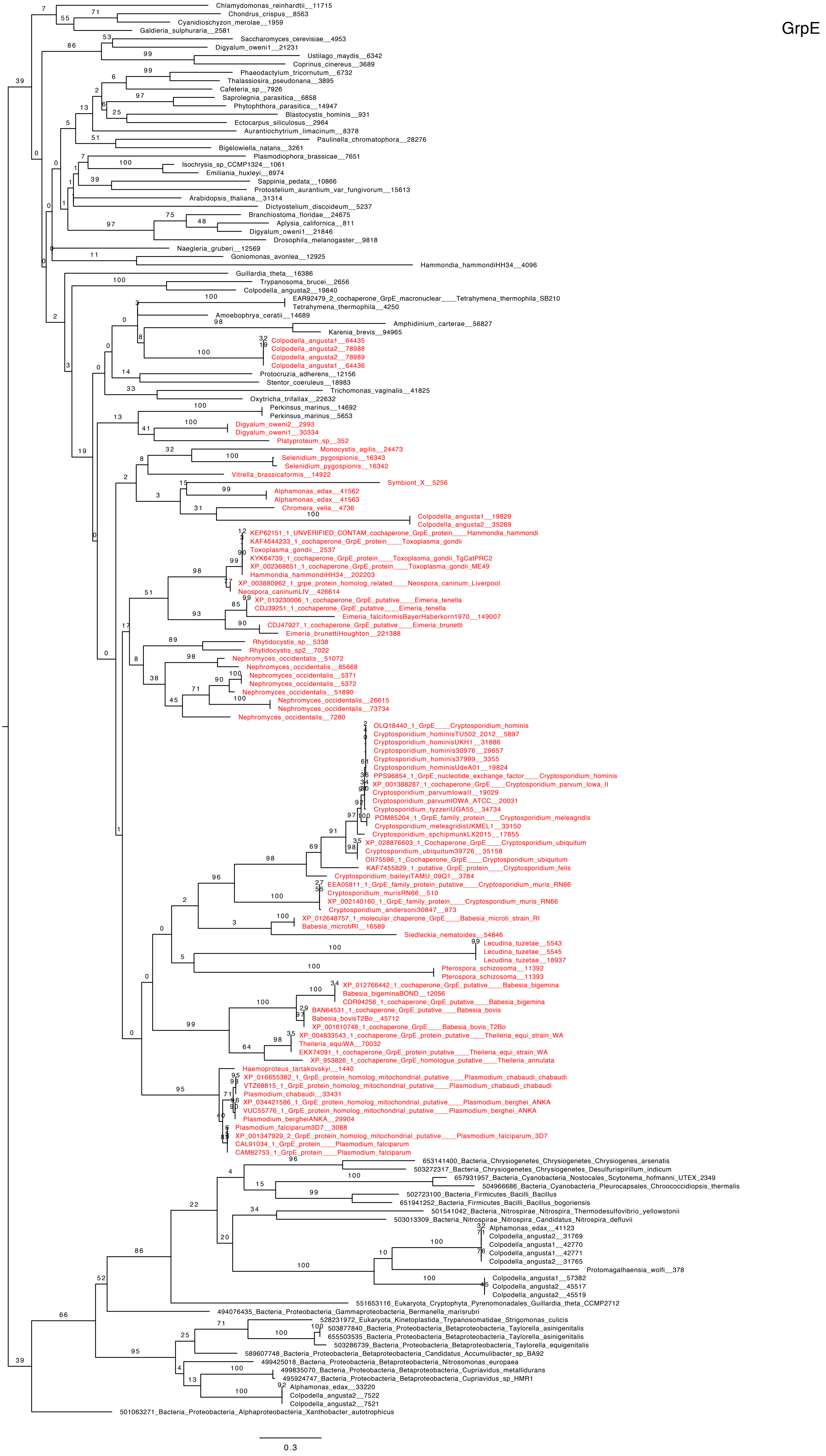
GrpE
Chlamydomonas_reinhardtii__11715
Chondrus_crispus__8563
Cyanidioschyzon_merolae__1959
Galdieria_sulphuraria__2581
Saccharomyces_cerevisiae__4953
Digyalum_oweni1__21231
Ustilago_maydis__6342
Coprinus_cinereus__3689
Phaeodactylum_tricornutum__6732
Thalassiosira_pseudonana__3895
Cafeteria_sp__7926
Saprolegnia_parasitica__6858
Phytophthora_parasitica__14947
Blastocystis_hominis__931
Ectocarpus_siliculosus__2964
Aurantiochytrium_limacinum__8378
Paulinella_chromatophora__28276
Bigelowiella_natans__3261
Plasmodiophora_brassicae__7651
Isochrysis_sp_CCMP1324__1061
Emiliania_huxleyi__8974
Sappinia_pedata__10866
Protostelium_aurantium_var_fungivorum__15613
Arabidopsis_thaliana__31314
Dictyostelium_discoideum__5237
Branchiostoma_floridae__24675
Aplysia_californica__811
Digyalum_oweni1__21846
Drosophila_melanogaster__9818
Naegleria_gruberi__12569
Goniomonas_avonlea__12925
Hammondia_hammondiHH34__4096
Guillardia_theta__16386
Trypanosoma_brucei__2656
Colpodella_angusta2__19840
EAR92479_2_cochaperone_GrpE_macronuclear____Tetrahymena_thermophila_SB210
Tetrahymena_thermophila__4250
Amoebophrya_ceratii__14689
Amphidinium_carterae__56827
Karenia_brevis__94965
Colpodella_angusta1__64435
Colpodella_angusta2__78988
Colpodella_angusta2__78989
Colpodella_angusta1__64436
Protocruzia_adherens__12156
Stentor_coeruleus__18983
Trichomonas_vaginalis__41825
Oxytricha_trifallax__22632
Perkinsus_marinus__14692
Perkinsus_marinus__5653
Digyalum_oweni2__2993
Digyalum_oweni1__30334
Platyproteum_sp__352
Monocystis_agilis__24473
Selenidium_pygospionis__16343
Selenidium_pygospionis__16342
Vitrella_brassicaformis__14922
Symbiont_X__5256
Alphamonas_edax__41562
Alphamonas_edax__41563
Chromera_velia__4736
Colpodella_angusta1__19829
Colpodella_angusta2__35269
KEP62151_1_UNVERIFIED_CONTAM_cochaperone_GrpE_protein____Hammondia_hammondi
KAF4644233_1_cochaperone_GrpE_protein____Toxoplasma_gondii
Toxoplasma_gondii__2537
KYK64739_1_cochaperone_GrpE_protein____Toxoplasma_gondii_TgCatPRC2
XP_002368651_1_cochaperone_GrpE_protein____Toxoplasma_gondii_ME49
Hammondia_hammondiHH34__202203
XP_003880962_1_grpe_protein_homolog_related____Neospora_caninum_Liverpool
Neospora_caninumLIV__426614
XP_013230006_1_cochaperone_GrpE_putative____Eimeria_tenella
CDJ39251_1_cochaperone_GrpE_putative____Eimeria_tenella
Eimeria_falciformisBayerHaberkorn1970__149007
CDJ47927_1_cochaperone_GrpE_putative____Eimeria_brunetti
Eimeria_brunettiHoughton__221388
Rhytidocystis_sp__5338
Rhytidocystis_sp2__7022
Nephromyces_occidentalis__51072
Nephromyces_occidentalis__85668
Nephromyces_occidentalis__5371
Nephromyces_occidentalis__5372
Nephromyces_occidentalis__51890
Nephromyces_occidentalis__26615
Nephromyces_occidentalis__73734
Nephromyces_occidentalis__7280
OLQ18440_1_GrpE____Cryptosporidium_hominis
Cryptosporidium_hominisTU502_2012__5897
Cryptosporidium_hominisUKH1__31886
Cryptosporidium_hominis30976__29657
Cryptosporidium_hominis37999__3355
Cryptosporidium_hominisUdeA01__19824
PPS96854_1_GrpE_nucleotide_exchange_factor____Cryptosporidium_hominis
XP_001388287_1_cochaperone_GrpE____Cryptosporidium_parvum_Iowa_II
Cryptosporidium_parvumIowaII__19029
Cryptosporidium_parvumIOWA_ATCC__20031
Cryptosporidium_tyzzeriUGA55__34734
POM85204_1_GrpE_family_protein____Cryptosporidium_meleagridis
Cryptosporidium_meleagridisUKMEL1__33150
Cryptosporidium_spchipmunkLX2015__17855
XP_028876603_1_Cochaperone_GrpE____Cryptosporidium_ubiquitum
Cryptosporidium_ubiquitum39726__35158
OII75596_1_Cochaperone_GrpE____Cryptosporidium_ubiquitum
KAF7455829_1_putative_GrpE_protein____Cryptosporidium_felis
Cryptosporidium_baileyiTAMU_09Q1__3784
EEA05811_1_GrpE_family_protein_putative____Cryptosporidium_muris_RN66
Cryptosporidium_murisRN66__510
XP_002140160_1_GrpE_family_protein____Cryptosporidium_muris_RN66
Cryptosporidium_andersoni30847__973
XP_012648757_1_molecular_chaperone_GrpE____Babesia_microti_strain_RI
Babesia_microtiRI__16589
Siedleckia_nematoides__54846
Lecudina_tuzetae__5543
Lecudina_tuzetae__5545
Lecudina_tuzetae__18937
Pterospora_schizosoma__11392
Pterospora_schizosoma__11393
XP_012766442_1_cochaperone_GrpE_putative____Babesia_bigemina
Babesia_bigeminaBOND__12056
CDR94256_1_cochaperone_GrpE_putative____Babesia_bigemina
BAN64531_1_cochaperone_GrpE_putative____Babesia_bovis
Babesia_bovisT2Bo__45712
XP_001610748_1_cochaperone_GrpE____Babesia_bovis_T2Bo
XP_004833543_1_cochaperone_GrpE_protein_putative____Theileria_equi_strain_WA
Theileria_equiWA__70032
EKX74091_1_cochaperone_GrpE_protein_putative____Theileria_equi_strain_WA
XP_953826_1_cochaperone_GrpE_homologue_putative____Theileria_annulata
Haemoproteus_tartakovskyi__1440
XP_016655382_1_GrpE_protein_homolog_mitochondrial_putative____Plasmodium_chabaudi_chabaudi
VTZ68815_1_GrpE_protein_homolog_mitochondrial_putative____Plasmodium_chabaudi_chabaudi
Plasmodium_chabaudi__33431
XP_034421586_1_GrpE_protein_homolog_mitochondrial_putative____Plasmodium_berghei_ANKA
VUC55776_1_GrpE_protein_homolog_mitochondrial_putative____Plasmodium_berghei_ANKA
Plasmodium_bergheiANKA__29904
Plasmodium_falciparum3D7__3088
XP_001347929_2_GrpE_protein_homolog_mitochondrial_putative____Plasmodium_falciparum_3D7
CAL91034_1_GrpE_protein____Plasmodium_falciparum
CAM82753_1_GrpE_protein____Plasmodium_falciparum
653141400_Bacteria_Chrysiogenetes_Chrysiogenetes_Chrysiogenes_arsenatis
503272317_Bacteria_Chrysiogenetes_Chrysiogenetes_Desulfurispirillum_indicum
657931957_Bacteria_Cyanobacteria_Nostocales_Scytonema_hofmanni_UTEX_2349
504966686_Bacteria_Cyanobacteria_Pleurocapsales_Chroococcidiopsis_thermalis
502723100_Bacteria_Firmicutes_Bacilli_Bacillus
651941252_Bacteria_Firmicutes_Bacilli_Bacillus_bogoriensis
501541042_Bacteria_Nitrospirae_Nitrospirae_Thermodesulfovibrio_yellowstonii
503013309_Bacteria_Nitrospirae_Nitrospira_Candidatus_Nitrospira_defluvii
Alphamonas_edax__41123
Colpodella_angusta2__31769
Colpodella_angusta1__42770
Colpodella_angusta1__42771
Colpodella_angusta2__31765
Protomagalhaensia_wolfi__378
Colpodella_angusta1__57382
Colpodella_angusta2__45517
Colpodella_angusta2__45519
551653116_Eukaryota_Cryptophyta_Pyrenomonadales_Guillardia_theta_CCMP2712
494076435_Bacteria_Proteobacteria_Gammaproteobacteria_Bermanella_marisrubri
528231972_Eukaryota_Kinetoplastida_Trypanosomatidae_Strigomonas_culicis
503877840_Bacteria_Proteobacteria_Betaproteobacteria_Taylorella_asinigenitalis
655503535_Bacteria_Proteobacteria_Betaproteobacteria_Taylorella_asinigenitalis
503286739_Bacteria_Proteobacteria_Betaproteobacteria_Taylorella_equigenitalis
589607748_Bacteria_Proteobacteria_Betaproteobacteria_Candidatus_Accumulibacter_sp_BA92
499425018_Bacteria_Proteobacteria_Betaproteobacteria_Nitrosomonas_europaea
499835070_Bacteria_Proteobacteria_Betaproteobacteria_Cupriavidus_metallidurans
495924747_Bacteria_Proteobacteria_Betaproteobacteria_Cupriavidus_sp_HMR1
Alphamonas_edax__33220
Colpodella_angusta2__7522
Colpodella_angusta2__7521
501063271_Bacteria_Proteobacteria_Alphaproteobacteria_Xanthobacter_autotrophicus
0.3

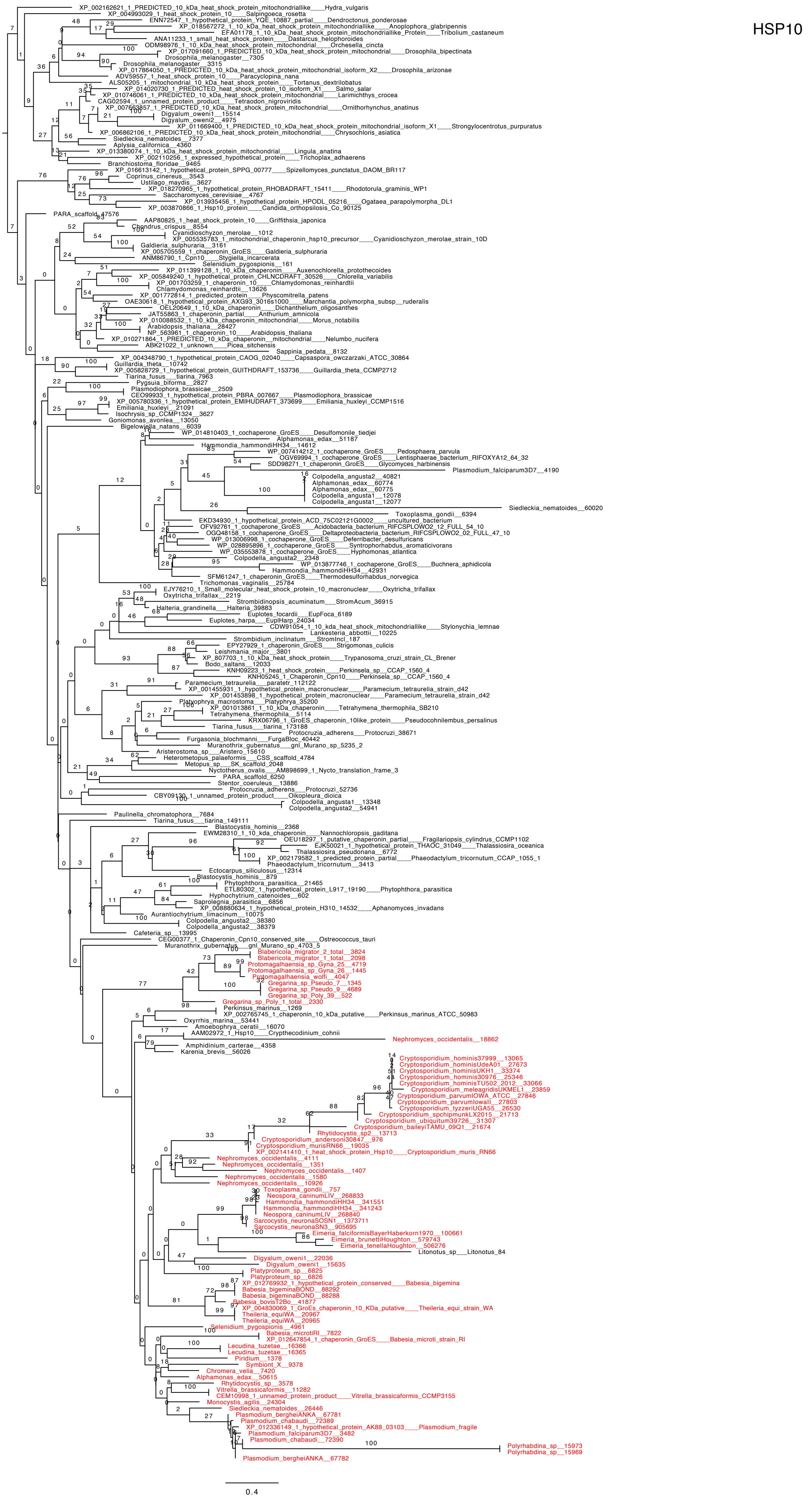
HSP10
XP_002162621_1_PREDICTED_10_kDa_heat_shock_protein_mitochondriallike____Hydra_vulgaris
XP_004993029_1_heat_shock_protein_10____Salpingoeca_rosetta
ENN72547_1_hypothetical_protein_YQE_10887_partial____Dendroctonus_ponderosae
XP_018567272_1_10_kDa_heat_shock_protein_mitochondriallike____Anoplophora_glabripennis
EFA01178_1_10_kDa_heat_shock_protein_mitochondriallike_Protein____Tribolium_castaneum
ANA11233_1_small_heat_shock_protein____Dastarcus_helophoroides
ODM98976_1_10_kDa_heat_shock_protein_mitochondrial____Orchesella_cincta
XP_017091660_1_PREDICTED_10_kDa_heat_shock_protein_mitochondrial____Drosophila_bipectinata
Drosophila_melanogaster__7305
Drosophila_melanogaster__3315
XP_017864050_1_PREDICTED_10_kDa_heat_shock_protein_mitochondrial_isoform_X2____Drosophila_arizonae
ADV59557_1_heat_shock_protein_10____Paracyclopina_nana
ALS05205_1_mitochondrial_10_kDa_heat_shock_protein____Tortanus_dextrilobatus
XP_014020730_1_PREDICTED_heat_shock_protein_10_isoform_X1____Salmo_salar
XP_010746061_1_PREDICTED_10_kDa_heat_shock_protein_mitochondrial____Larimichthys_crocea
CAG02594_1_unnamed_protein_product____Tetraodon_nigroviridis
XP_007663857_1_PREDICTED_10_kDa_heat_shock_protein_mitochondrial____Ornithorhynchus_anatinus
Digyalum_oweni1__15514
Digyalum_oweni2__4975
XP_011669400_1_PREDICTED_10_kDa_heat_shock_protein_mitochondrial_isoform_X1____Strongylocentrotus_purpuratus
XP_006862106_1_PREDICTED_10_kDa_heat_shock_protein_mitochondrial____Chrysochloris_asiatica
Siedleckia_nematoides__7377
Aplysia_californica__4360
XP_013380074_1_10_kDa_heat_shock_protein_mitochondrial____Lingula_anatina
XP_002110256_1_expressed_hypothetical_protein____Trichoplax_adhaerens
Branchiostoma_floridae__9465
XP_016613142_1_hypothetical_protein_SPPG_00777____Spizellomyces_punctatus_DAOM_BR117
Coprinus_cinereus__3543
Ustilago_maydis__3627
XP_018270965_1_hypothetical_protein_RHOBADRAFT_15411____Rhodotorula_graminis_WP1
Saccharomyces_cerevisiae__4767
XP_013935456_1_hypothetical_protein_HPODL_05216____Ogataea_parapolymorpha_DL1
XP_003870866_1_Hsp10_protein____Candida_orthopsilosis_Co_90125
PARA_scaffold_47576
AAP80825_1_heat_shock_protein_10____Griffithsia_japonica
Chondrus_crispus__8554
Cyanidioschyzon_merolae__1012
XP_005535783_1_mitochondrial_chaperonin_hsp10_precursor____Cyanidioschyzon_merolae_strain_10D
Galdieria_sulphuraria__3161
XP_005705559_1_chaperonin_GroES____Galdieria_sulphuraria
ANM86790_1_Cpn10____Stygiella_incarcerata
Selenidium_pygospionis__161
XP_011399128_1_10_kDa_chaperonin____Auxenochlorella_protothecoides
XP_005849240_1_hypothetical_protein_CHLNCDRAFT_30526____Chlorella_variabilis
XP_001703259_1_chaperonin_10____Chlamydomonas_reinhardtii
Chlamydomonas_reinhardtii__13626
XP_001772814_1_predicted_protein____Physcomitrella_patens
OAE30618_1_hypothetical_protein_AXG93_3016s1000____Marchantia_polymorpha_subsp__ruderalis
OEL20649_1_10_kDa_chaperonin____Dichanthelium_oligosanthes
JAT55863_1_chaperonin_partial____Anthurium_amnicola
XP_010088532_1_10_kDa_chaperonin_mitochondrial____Morus_notabilis
Arabidopsis_thaliana__28427
NP_563961_1_chaperonin_10____Arabidopsis_thaliana
XP_010271864_1_PREDICTED_10_kDa_chaperonin__mitochondrial____Nelumbo_nucifera
ABK21022_1_unknown____Picea_sitchensis
Sappinia_pedata__8132
XP_004348790_1_hypothetical_protein_CAOG_02040____Capsaspora_owczarzaki_ATCC_30864
Guillardia_theta__10742
XP_005828729_1_hypothetical_protein_GUITHDRAFT_153736____Guillardia_theta_CCMP2712
Tiarina_fusus___tiarina_7963
Pygsuia_biforma__2827
Plasmodiophora_brassicae__2509
CEO99933_1_hypothetical_protein_PBRA_007667____Plasmodiophora_brassicae
XP_005780336_1_hypothetical_protein_EMIHUDRAFT_373699____Emiliania_huxleyi_CCMP1516
Emiliania_huxleyi__21091
Isochrysis_sp_CCMP1324__3627
Goniomonas_avonlea__13050
Bigelowiella_natans__6039
WP_014810403_1_cochaperone_GroES____Desulfomonile_tiedjei
Alphamonas_edax__51187
Hammondia_hammondiHH34__14612
WP_007414212_1_cochaperone_GroES____Pedosphaera_parvula
OGV69994_1_cochaperone_GroES____Lentisphaerae_bacterium_RIFOXYA12_64_32
SDD98271_1_chaperonin_GroES____Glycomyces_harbinensis
Plasmodium_falciparum3D7__4190
Colpodella_angusta2__40821
Alphamonas_edax__60774
Alphamonas_edax__60775
Colpodella_angusta1__12078
Colpodella_angusta1__12077
Siedleckia_nematoides__60020
Toxoplasma_gondii__6394
EKD34930_1_hypothetical_protein_ACD_75C02121G0002____uncultured_bacterium
OFV92761_1_cochaperone_GroES____Acidobacteria_bacterium_RIFCSPLOWO2_12_FULL_54_10
OGQ48158_1_cochaperone_GroES____Deltaproteobacteria_bacterium_RIFCSPLOWO2_02_FULL_47_10
WP_013006998_1_cochaperone_GroES____Deferribacter_desulfuricans
WP_028895896_1_cochaperone_GroES____Syntrophorhabdus_aromaticivorans
WP_035553878_1_cochaperone_GroES____Hyphomonas_atlantica
Colpodella_angusta2__2348
WP_013877746_1_cochaperone_GroES____Buchnera_aphidicola
Hammondia_hammondiHH34__42931
SFM61247_1_chaperonin_GroES____Thermodesulforhabdus_norvegica
Trichomonas_vaginalis__25784
EJY76210_1_Small_molecular_heat_shock_protein_10_macronuclear____Oxytricha_trifallax
Oxytricha_trifallax__2219
Strombidinopsis_acuminatum___StromAcum_36915
Halteria_grandinella___Halteria_39883
Euplotes_focardii___EupFoca_6189
Euplotes_harpa___EuplHarp_24034
CDW91054_1_10_kda_heat_shock_mitochondriallike____Stylonychia_lemnae
Lankesteria_abbottii__10225
Strombidium_inclinatum___StromIncl_187
EPY27929_1_chaperonin_GroES____Strigomonas_culicis
Leishmania_major__3801
XP_807703_1_10_kDa_heat_shock_protein____Trypanosoma_cruzi_strain_CL_Brener
Bodo_saltans__12033
KNH09223_1_heat_shock_protein____Perkinsela_sp__CCAP_1560_4
KNH05245_1_Chaperonin_Cpn10____Perkinsela_sp__CCAP_1560_4
Paramecium_tetraurelia___paratetr_112122
XP_001455931_1_hypothetical_protein_macronuclear____Paramecium_tetraurelia_strain_d42
XP_001453898_1_hypothetical_protein_macronuclear____Paramecium_tetraurelia_strain_d42
Platyophrya_macrostoma___Platyphrya_35200
XP_001013861_1_10_kDa_chaperonin____Tetrahymena_thermophila_SB210
Tetrahymena_thermophila__5114
KRX06796_1_GroES_chaperonin_10like_protein____Pseudocohnilembus_persalinus
Tiarina_fusus___tiarina_173188
Protocruzia_adherens___Protocruzi_38671
Furgasonia_blochmanni___FurgaBloc_40442
Muranothrix_gubernatus___gnl_Murano_sp_5235_2
Aristerostoma_sp___Aristero_15610
Heterometopus_palaeformis___CSS_scaffold_4784
Metopus_sp___SK_scaffold_2048
Nyctotherus_ovalis___AM898699_1_Nycto_translation_frame_3
PARA_scaffold_6250
Stentor_coeruleus__13886
Protocruzia_adherens___Protocruzi_52736
CBY09130_1_unnamed_protein_product____Oikopleura_dioica
Colpodella_angusta1__13348
Colpodella_angusta2__54941
Paulinella_chromatophora__7684
Tiarina_fusus___tiarina_149111
Blastocystis_hominis__2368
EWM28310_1_10_kda_chaperonin____Nannochloropsis_gaditana
OEU18297_1_putative_chaperonin_partial____Fragilariopsis_cylindrus_CCMP1102
EJK50021_1_hypothetical_protein_THAOC_31049____Thalassiosira_oceanica
Thalassiosira_pseudonana__6772
XP_002179582_1_predicted_protein_partial____Phaeodactylum_tricornutum_CCAP_1055_1
Phaeodactylum_tricornutum__3413
Ectocarpus_siliculosus__12314
Blastocystis_hominis__879
Phytophthora_parasitica__21465
ETL80302_1_hypothetical_protein_L917_19190____Phytophthora_parasitica
Hyphochytrium_catenoides__602
Saprolegnia_parasitica__6856
XP_008880634_1_hypothetical_protein_H310_14532____Aphanomyces_invadans
Aurantiochytrium_limacinum__10075
Colpodella_angusta2__38380
Colpodella_angusta2__38379
Cafeteria_sp__13995
CEG00377_1_Chaperonin_Cpn10_conserved_site____Ostreococcus_tauri
Muranothrix_gubernatus___gnl_Murano_sp_4703_5
Blabericola_migrator_2_total__3824
Blabericola_migrator_1_total__2098
Protomagalhaensia_sp_Gyna_25__4719
Protomagalhaensia_sp_Gyna_26__1445
Protomagalhaensia_wolfi__4047
Gregarina_sp_Pseudo_7__1345
Gregarina_sp_Pseudo_9__4689
Gregarina_sp_Poly_39__522
Gregarina_sp_Poly_1_total__2330
Perkinsus_marinus__1269
XP_002765745_1_chaperonin_10_kDa_putative____Perkinsus_marinus_ATCC_50983
Oxyrrhis_marina__53441
Amoebophrya_ceratii__16070
AAM02972_1_Hsp10____Crypthecodinium_cohnii
Nephromyces_occidentalis__18862
Amphidinium_carterae__4358
Karenia_brevis__56026
Cryptosporidium_hominis37999__13065
Cryptosporidium_hominisUdeA01__27673
Cryptosporidium_hominisUKH1__33374
Cryptosporidium_hominis30976__25346
Cryptosporidium_hominisTU502_2012__33066
Cryptosporidium_meleagridisUKMEL1__23859
Cryptosporidium_parvumIOWA_ATCC__27846
Cryptosporidium_parvumIowaII__27803
Cryptosporidium_tyzzeriUGA55__26530
Cryptosporidium_spchipmunkLX2015__21713
Cryptosporidium_ubiquitum39726__31307
Cryptosporidium_baileyiTAMU_09Q1__21674
Rhytidocystis_sp2__13713
Cryptosporidium_andersoni30847__976
Cryptosporidium_murisRN66__19035
XP_002141410_1_heat_shock_protein_Hsp10____Cryptosporidium_muris_RN66
Nephromyces_occidentalis__4111
Nephromyces_occidentalis__1351
Nephromyces_occidentalis__1407
Nephromyces_occidentalis__1580
Nephromyces_occidentalis__10926
Toxoplasma_gondii__757
Neospora_caninumLIV__268833
Hammondia_hammondiHH34__341551
Hammondia_hammondiHH34__341243
Neospora_caninumLIV__268840
Sarcocystis_neuronaSOSN1__1373711
Sarcocystis_neuronaSN3__905695
Eimeria_falciformisBayerHaberkorn1970__100661
Eimeria_brunettiHoughton__579743
Eimeria_tenellaHoughton__506276
Litonotus_sp___Litonotus_84
Digyalum_oweni1__22036
Digyalum_oweni1__15635
Platyproteum_sp__6825
Platyproteum_sp__6826
XP_012769932_1_hypothetical_protein_conserved____Babesia_bigemina
Babesia_bigeminaBOND__88292
Babesia_bigeminaBOND__88288
Babesia_bovisT2Bo__41877
XP_004830069_1_GroES_chaperonin_10_KDa_putative____Theileria_equi_strain_WA
Theileria_equiWA__20967
Theileria_equiWA__20965
Selenidium_pygospionis__4961
Babesia_microtiRI__7822
XP_012647854_1_chaperonin_GroES____Babesia_microti_strain_RI
Lecudina_tuzetae__16366
Lecudina_tuzetae__16365
Piridium__1378
Symbiont_X__9378
Chromera_velia__7420
Alphamonas_edax__50615
Rhytidocystis_sp__3578
Vitrella_brassicaformis__11282
CEM10998_1_unnamed_protein_product____Vitrella_brassicaformis_CCMP3155
Monocystis_agilis__24304
Siedleckia_nematoides__26446
Plasmodium_bergheiANKA__67781
Plasmodium_chabaudi__72389
XP_012336149_1_hypothetical_protein_AK88_03103____Plasmodium_fragile
Plasmodium_falciparum3D7__3482
Plasmodium_chabaudi__72390
Polyrhabdina_sp__15973
Polyrhabdina_sp__15969
Plasmodium_bergheiANKA__67782
0.4

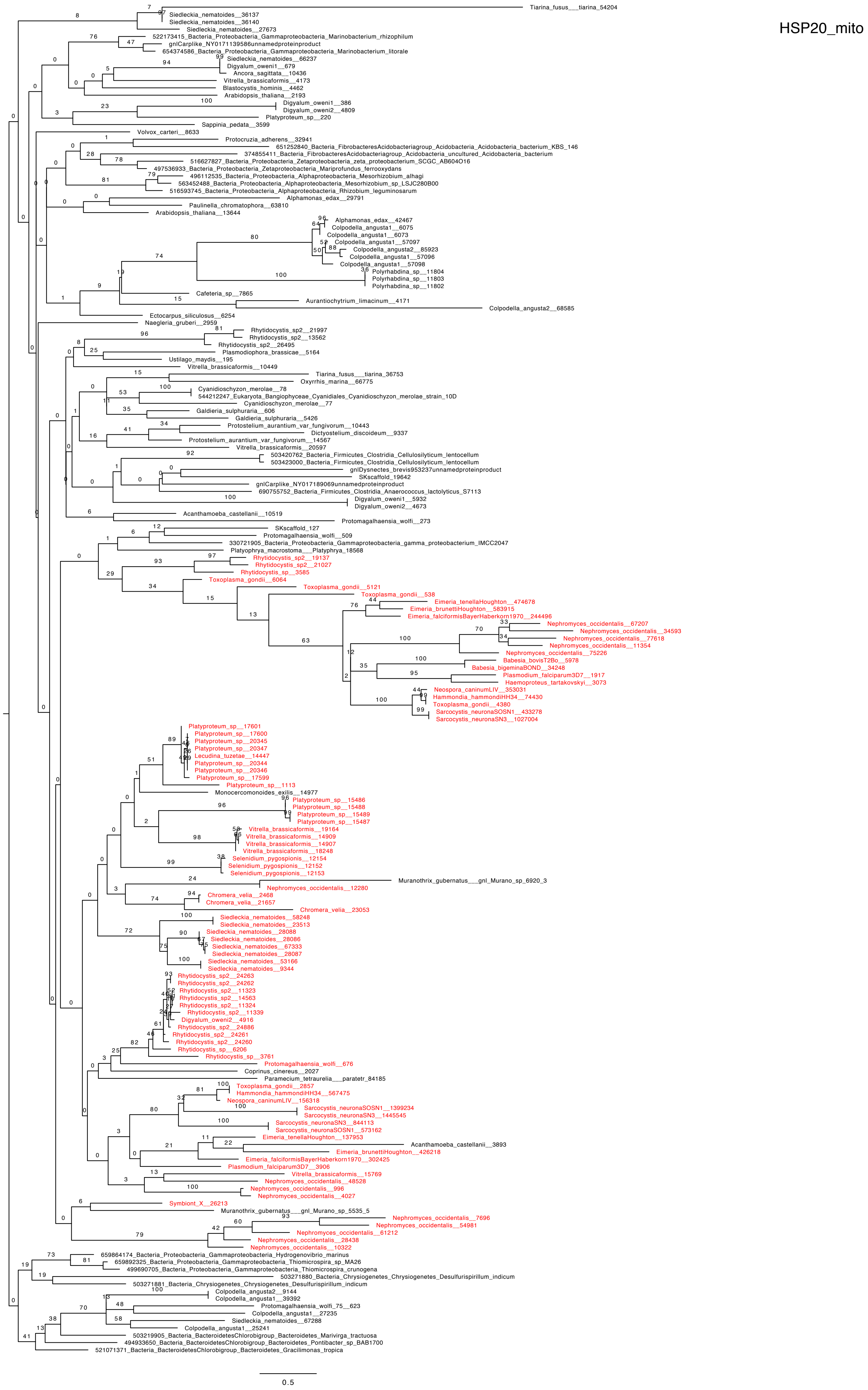

HSP20_mito
Tiarina_fusus___tiarina_54204
Siedleckia_nematoides__36137
Siedleckia_nematoides__36140
Siedleckia_nematoides__27673
522173415_Bacteria_Proteobacteria_Gammaproteobacteria_Marinobacterium_rhizophilum
gnlCarplike_NY0171139586unnamedproteinproduct
654374586_Bacteria_Proteobacteria_Gammaproteobacteria_Marinobacterium_litorale
Siedleckia_nematoides__66237
Digyalum_oweni1__679
Ancora_sagittata__10436
Vitrella_brassicaformis__4173
Blastocystis_hominis__4462
Arabidopsis_thaliana__2193
Digyalum_oweni1__386
Digyalum_oweni2__4809
Platyproteum_sp__220
Sappinia_pedata__3599
Volvox_carteri__8633
Protocruzia_adherens__32941
651252840_Bacteria_FibrobacteresAcidobacteriagroup_Acidobacteria_Acidobacteria_bacterium_KBS_146
374855411_Bacteria_FibrobacteresAcidobacteriagroup_Acidobacteria_uncultured_Acidobacteria_bacterium
516627827_Bacteria_Proteobacteria_Zetaproteobacteria_zeta_proteobacterium_SCGC_AB604O16
497536933_Bacteria_Proteobacteria_Zetaproteobacteria_Mariprofundus_ferrooxydans
496112535_Bacteria_Proteobacteria_Alphaproteobacteria_Mesorhizobium_alhagi
563452488_Bacteria_Proteobacteria_Alphaproteobacteria_Mesorhizobium_sp_LSJC280B00
516593745_Bacteria_Proteobacteria_Alphaproteobacteria_Rhizobium_leguminosarum
Alphamonas_edax__29791
Paulinella_chromatophora__63810
Arabidopsis_thaliana__13644
Alphamonas_edax__42467
Colpodella_angusta1__6075
Colpodella_angusta1__6073
Colpodella_angusta1__57097
Colpodella_angusta2__85923
Colpodella_angusta1__57096
Colpodella_angusta1__57098
Polyrhabdina_sp__11804
Polyrhabdina_sp__11803
Polyrhabdina_sp__11802
Cafeteria_sp__7865
Aurantiochytrium_limacinum__4171
Colpodella_angusta2__68585
Ectocarpus_siliculosus__6254
Naegleria_gruberi__2959
Rhytidocystis_sp2__21997
Rhytidocystis_sp2__13562
Rhytidocystis_sp2__26495
Plasmodiophora_brassicae__5164
Ustilago_maydis__195
Vitrella_brassicaformis__10449
Tiarina_fusus___tiarina_36753
Oxyrrhis_marina__66775
Cyanidioschyzon_merolae__78
544212247_Eukaryota_Bangiophyceae_Cyanidiales_Cyanidioschyzon_merolae_strain_10D
Cyanidioschyzon_merolae__77
Galdieria_sulphuraria__606
Galdieria_sulphuraria__5426
Protostelium_aurantium_var_fungivorum__10443
Dictyostelium_discoideum__9337
Protostelium_aurantium_var_fungivorum__14567
Vitrella_brassicaformis__20597
503420762_Bacteria_Firmicutes_Clostridia_Cellulosilyticum_lentocellum
503423000_Bacteria_Firmicutes_Clostridia_Cellulosilyticum_lentocellum
gnlDysnectes_brevis953237unnamedproteinproduct
SKscaffold_19642
gnlCarplike_NY017189069unnamedproteinproduct
690755752_Bacteria_Firmicutes_Clostridia_Anaerococcus_lactolyticus_S7113
Digyalum_oweni1__5932
Digyalum_oweni2__4673
Acanthamoeba_castellanii__10519
Protomagalhaensia_wolfi__273
SKscaffold_127
Protomagalhaensia_wolfi__509
330721905_Bacteria_Proteobacteria_Gammaproteobacteria_gamma_proteobacterium_IMCC2047
Platyophrya_macrostoma___Platyphrya_18568
Rhytidocystis_sp2__19137
Rhytidocystis_sp2__21027
Rhytidocystis_sp__3585
Toxoplasma_gondii__6064
Toxoplasma_gondii__5121
Toxoplasma_gondii__538
Eimeria_tenellaHoughton__474678
Eimeria_brunettiHoughton__583915
Eimeria_falciformisBayerHaberkorn1970__244496
Nephromyces_occidentalis__67207
Nephromyces_occidentalis__34593
Nephromyces_occidentalis__77618
Nephromyces_occidentalis__11354
Nephromyces_occidentalis__75226
Babesia_bovisT2Bo__5978
Babesia_bigeminaBOND__34248
Plasmodium_falciparum3D7__1917
Haemoproteus_tartakovskyi__3073
Neospora_caninumLIV__353031
Hammondia_hammondiHH34__74430
Toxoplasma_gondii__4380
Sarcocystis_neuronaSOSN1__433278
Sarcocystis_neuronaSN3__1027004
Platyproteum_sp__17601
Platyproteum_sp__17600
Platyproteum_sp__20345
Platyproteum_sp__20347
Lecudina_tuzetae__14447
Platyproteum_sp__20344
Platyproteum_sp__20346
Platyproteum_sp__17599
Platyproteum_sp__1113
Monocercomonoides_exilis__14977
Platyproteum_sp__15486
Platyproteum_sp__15488
Platyproteum_sp__15489
Platyproteum_sp__15487
Vitrella_brassicaformis__19164
Vitrella_brassicaformis__14909
Vitrella_brassicaformis__14907
Vitrella_brassicaformis__18248
Selenidium_pygospionis__12154
Selenidium_pygospionis__12152
Selenidium_pygospionis__12153
Muranothrix_gubernatus___gnl_Murano_sp_6920_3
Nephromyces_occidentalis__12280
Chromera_velia__2468
Chromera_velia__21657
Chromera_velia__23053
Siedleckia_nematoides__58248
Siedleckia_nematoides__23513
Siedleckia_nematoides__28088
Siedleckia_nematoides__28086
Siedleckia_nematoides__67333
Siedleckia_nematoides__28087
Siedleckia_nematoides__53166
Siedleckia_nematoides__9344
Rhytidocystis_sp2__24263
Rhytidocystis_sp2__24262
Rhytidocystis_sp2__11323
Rhytidocystis_sp2__14563
Rhytidocystis_sp2__11324
Rhytidocystis_sp2__11339
Digyalum_oweni2__4916
Rhytidocystis_sp2__24886
Rhytidocystis_sp2__24261
Rhytidocystis_sp2__24260
Rhytidocystis_sp__6206
Rhytidocystis_sp__3761
Protomagalhaensia_wolfi__676
Coprinus_cinereus__2027
Paramecium_tetraurelia___paratetr_84185
Toxoplasma_gondii__2857
Hammondia_hammondiHH34__567475
Neospora_caninumLIV__156318
Sarcocystis_neuronaSOSN1__1399234
Sarcocystis_neuronaSN3__1445545
Sarcocystis_neuronaSN3__844113
Sarcocystis_neuronaSOSN1__573162
Eimeria_tenellaHoughton__137953
Acanthamoeba_castellanii__3893
Eimeria_brunettiHoughton__426218
Eimeria_falciformisBayerHaberkorn1970__302425
Plasmodium_falciparum3D7__3906
Vitrella_brassicaformis__15769
Nephromyces_occidentalis__48528
Nephromyces_occidentalis__996
Nephromyces_occidentalis__4027
Symbiont_X__26213
Muranothrix_gubernatus___gnl_Murano_sp_5535_5
Nephromyces_occidentalis__7696
Nephromyces_occidentalis__54981
Nephromyces_occidentalis__61212
Nephromyces_occidentalis__28438
Nephromyces_occidentalis__10322
659864174_Bacteria_Proteobacteria_Gammaproteobacteria_Hydrogenovibrio_marinus
659892325_Bacteria_Proteobacteria_Gammaproteobacteria_Thiomicrospira_sp_MA26
499690705_Bacteria_Proteobacteria_Gammaproteobacteria_Thiomicrospira_crunogena
503271880_Bacteria_Chrysiogenetes_Chrysiogenetes_Desulfurispirillum_indicum
503271881_Bacteria_Chrysiogenetes_Chrysiogenetes_Desulfurispirillum_indicum
Colpodella_angusta2__9144
Colpodella_angusta1__39392
Protomagalhaensia_wolfi_75__623
Colpodella_angusta1__27235
Siedleckia_nematoides__67288
Colpodella_angusta1__25241
503219905_Bacteria_BacteroidetesChlorobigroup_Bacteroidetes_Marivirga_tractuosa
494933650_Bacteria_BacteroidetesChlorobigroup_Bacteroidetes_Pontibacter_sp_BAB1700
521071371_Bacteria_BacteroidetesChlorobigroup_Bacteroidetes_Gracilimonas_tropica
0.5

HSP40

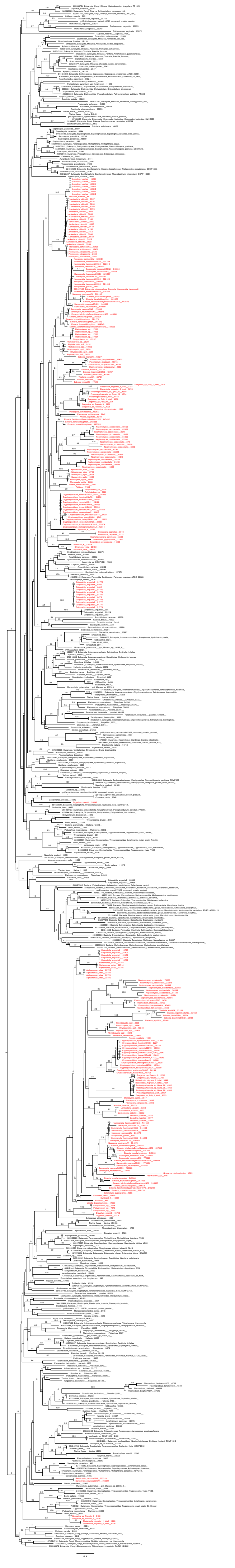

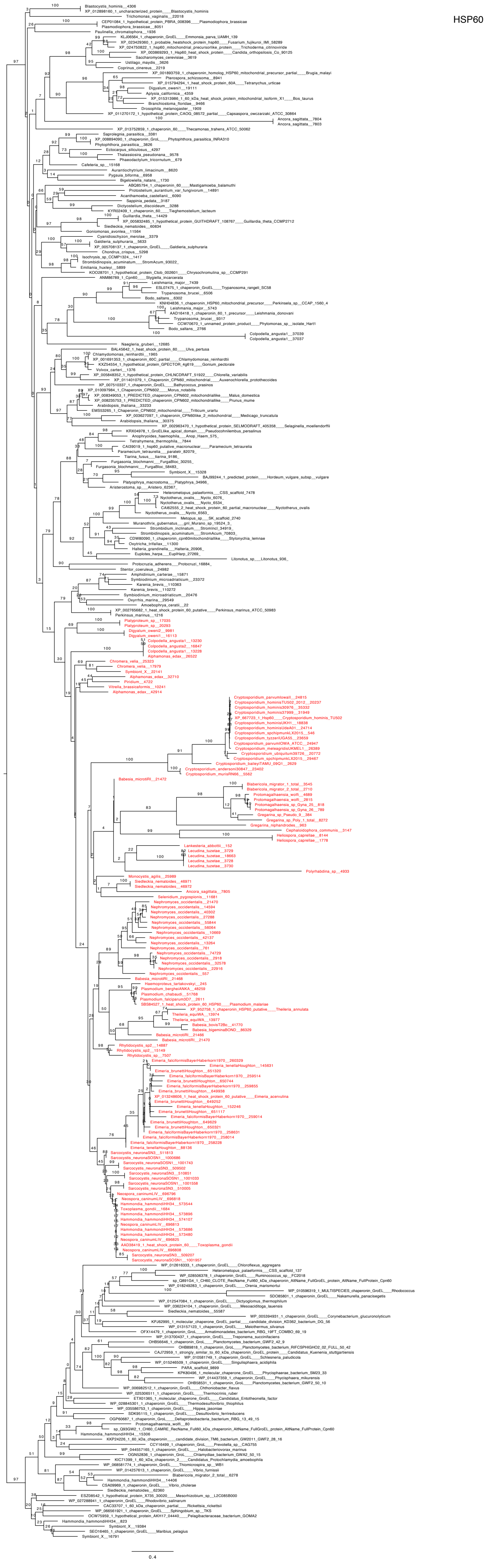
HSP60

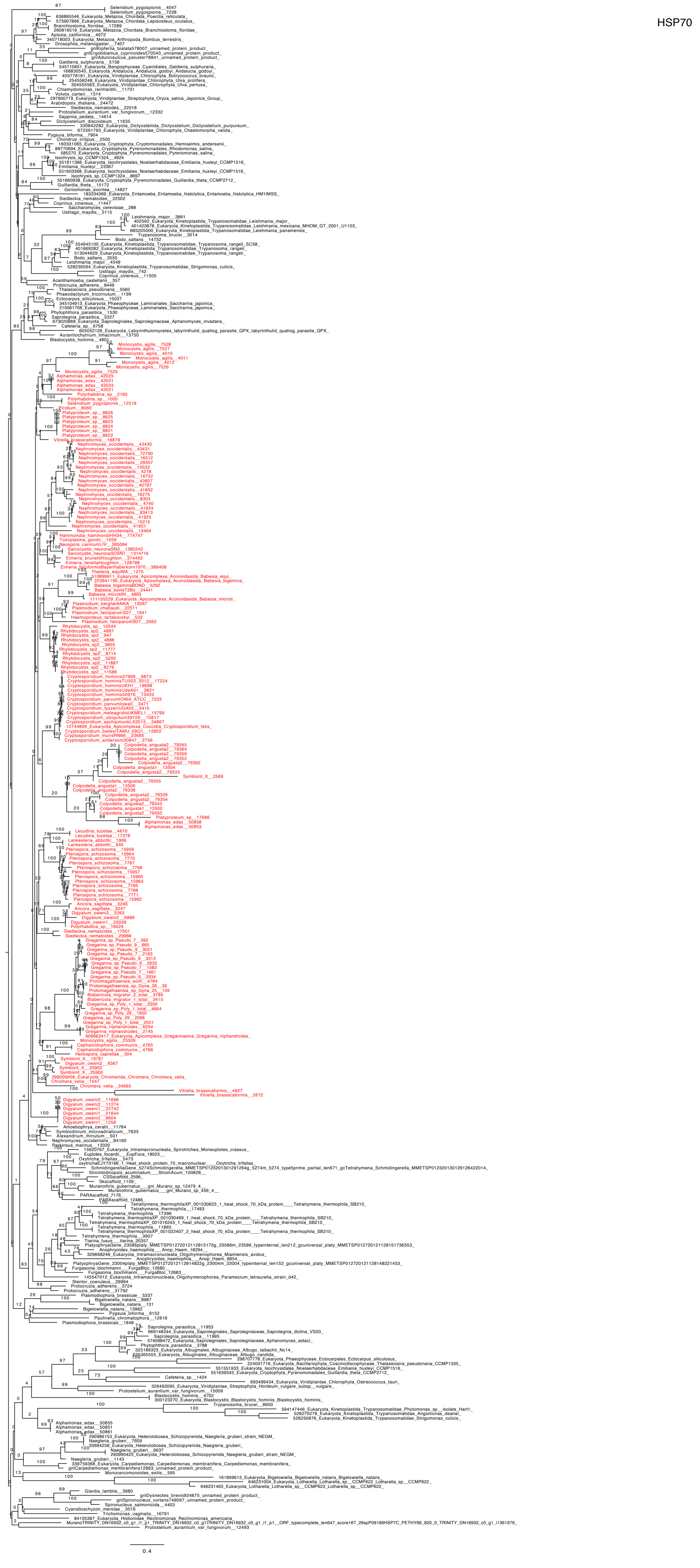
HSP70
0.4

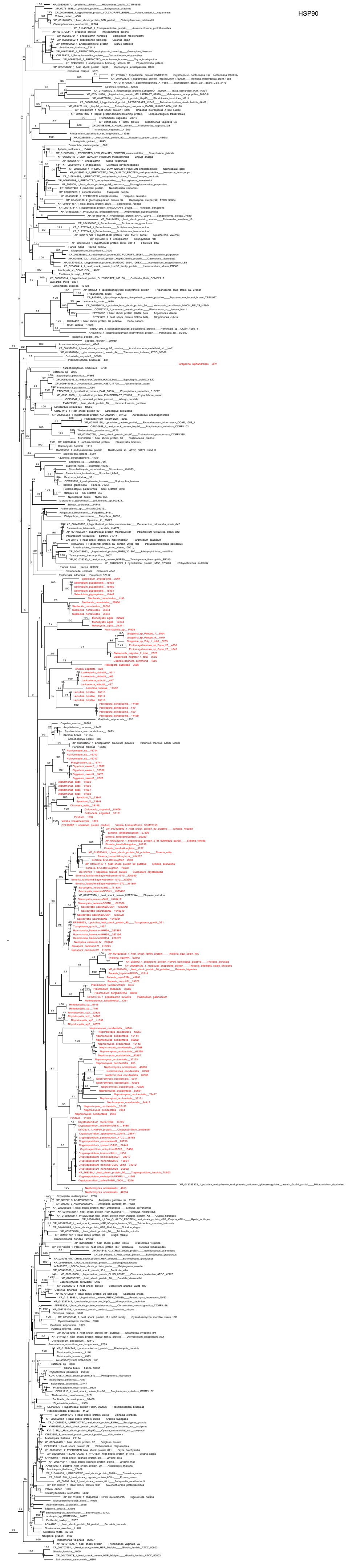
HSP90
0.4

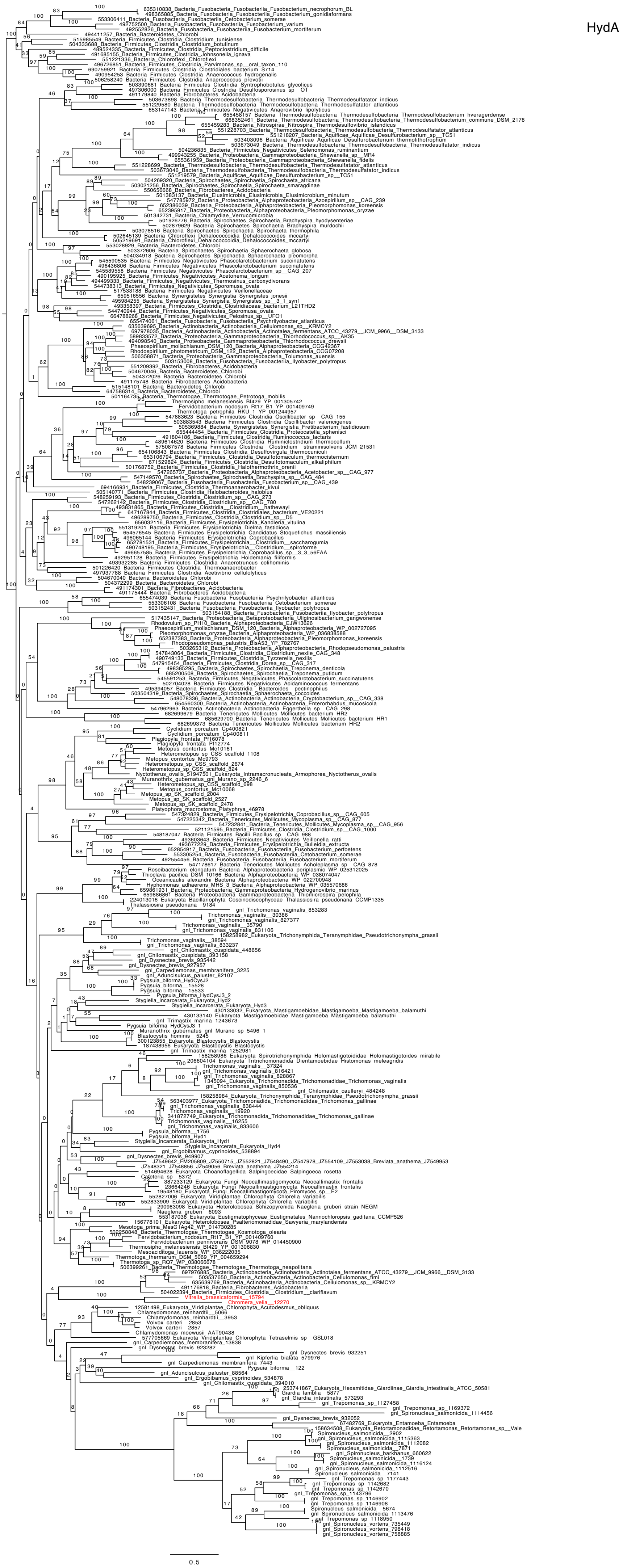

HydA
0.5

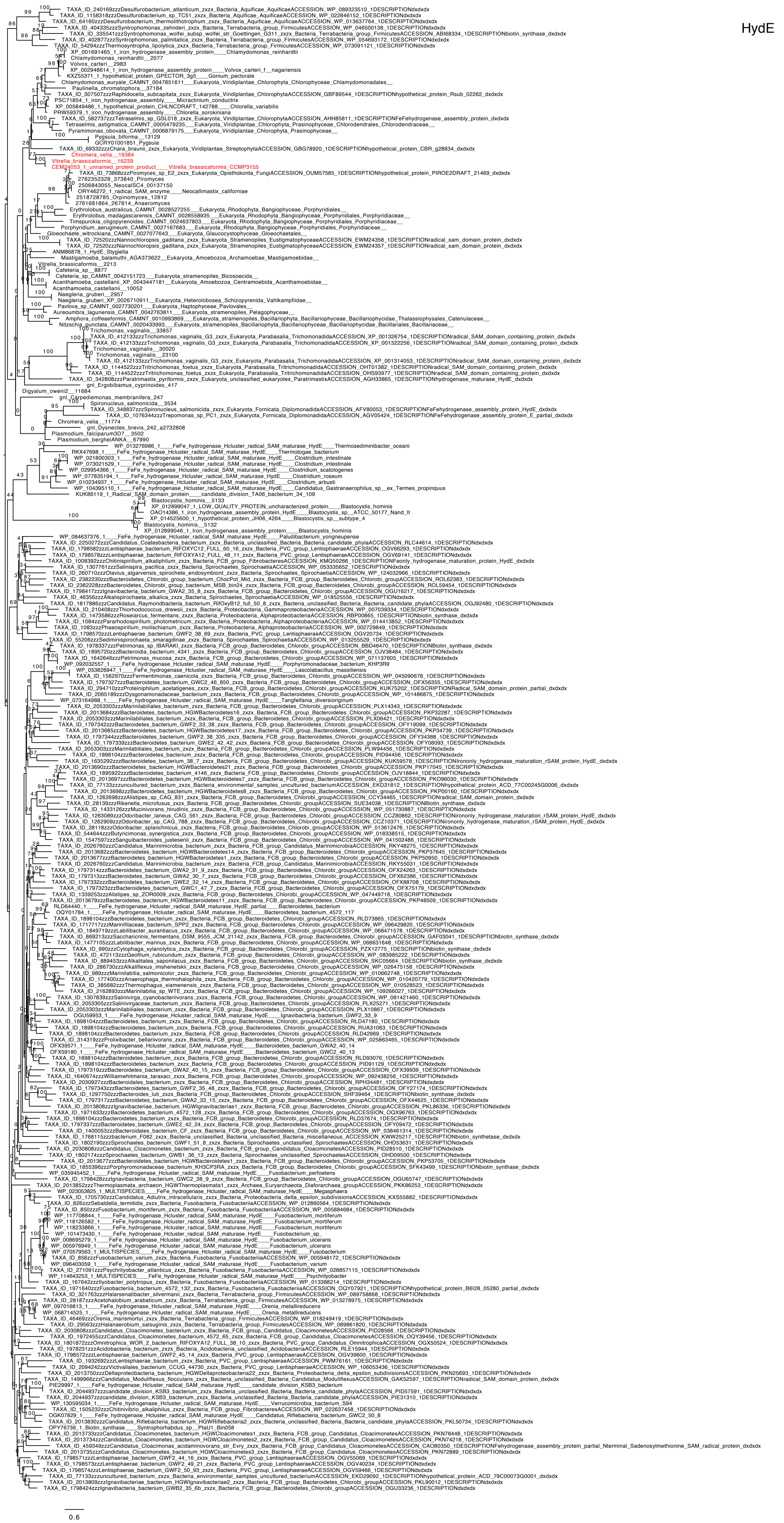
HydE
0.6

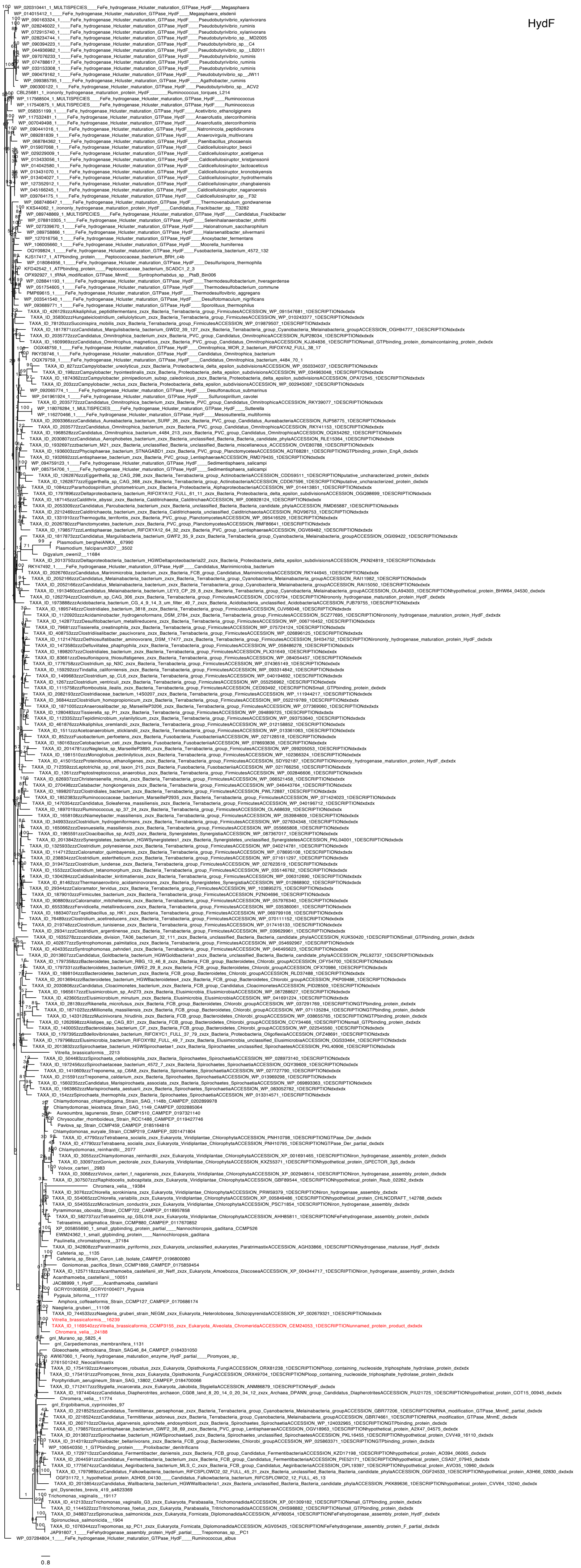
HydF
0.8

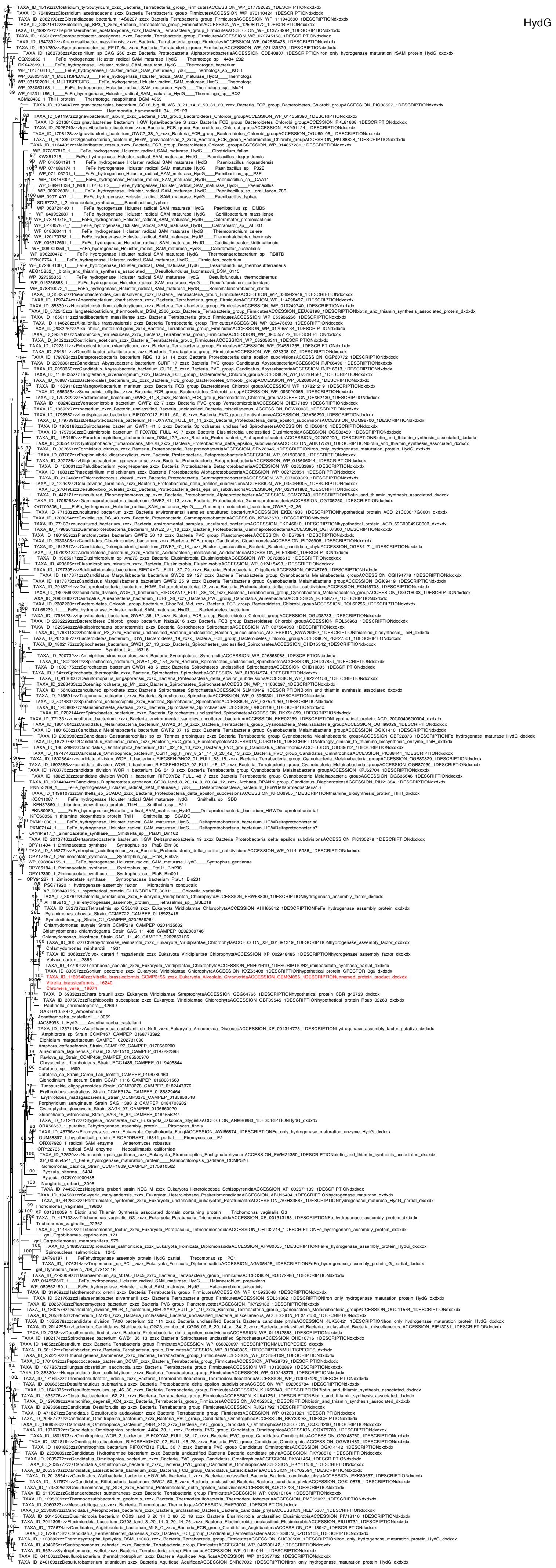
HydG
0.6

ICDH

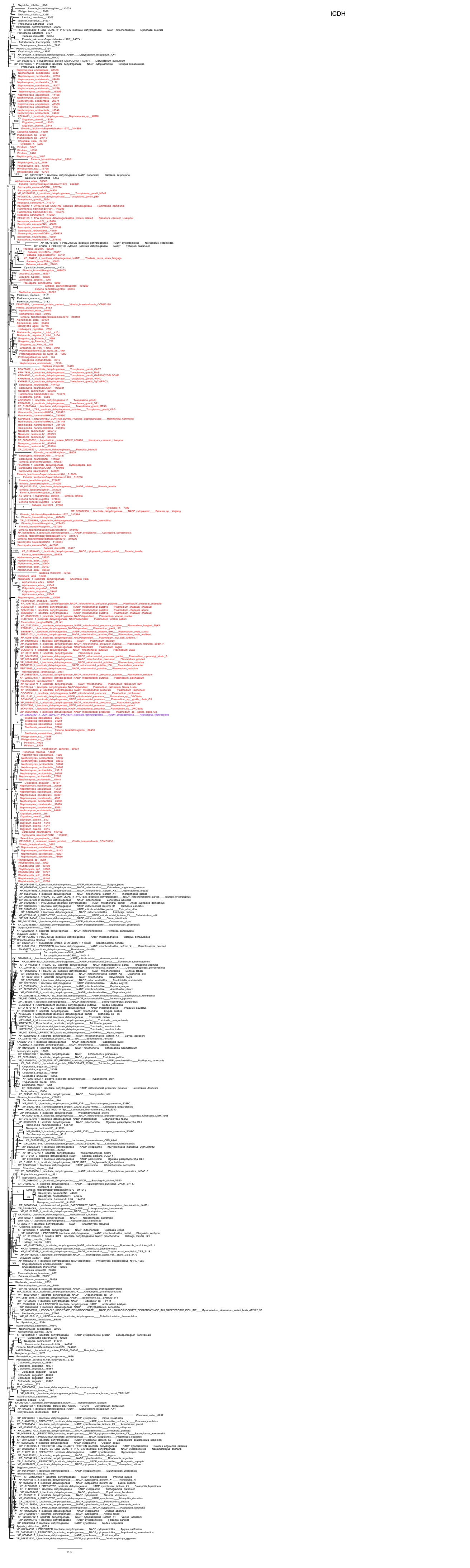

IND1

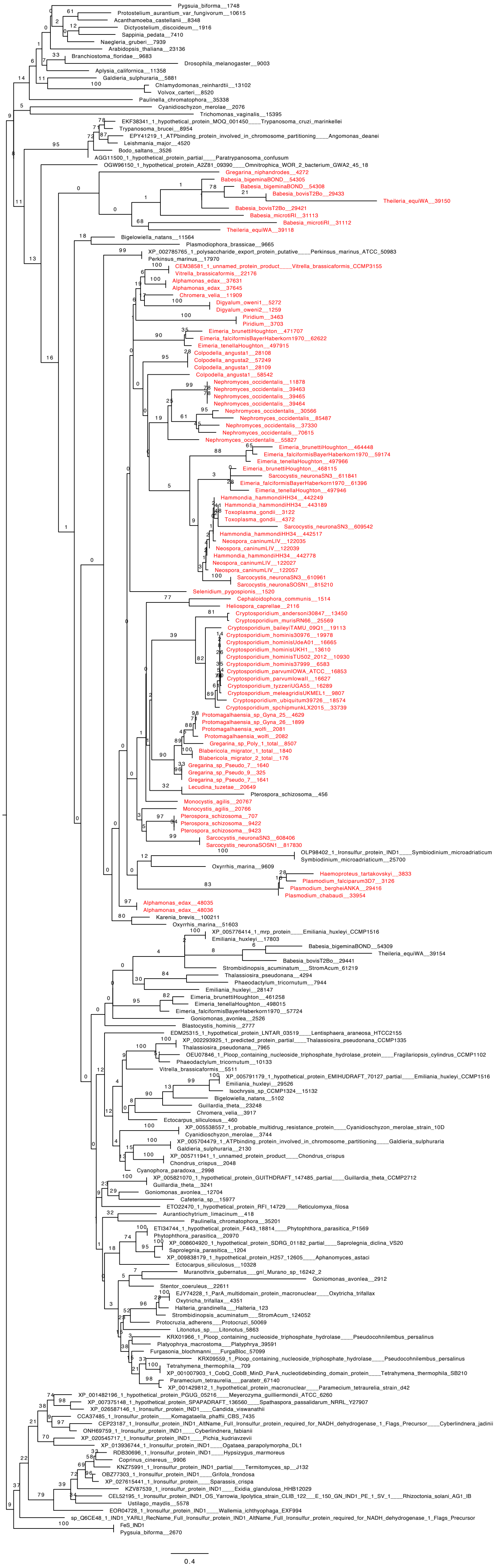
Pygsuia_biforma__1748
Protostelium_aurantium_var_fungivorum__10615
Acanthamoeba_castellanii__8348
Dictyostelium_discoideum__1916
Sappinia_pedata__7410
Naegleria_gruberi__7939
Arabidopsis_thaliana__23136
Branchiostoma_floridae__9683
Drosophila_melanogaster__9003
Aplysia_californica__11358
Galdieria_sulphuraria__5881
Chlamydomonas_reinhardtii__13102
Volvox_carteri__8520
Paulinella_chromatophora__35338
Cyanidioschyzon_merolae__2076
Trichomonas_vaginalis__15395
EKF38341_1_hypothetical_protein_MOQ_001450____Trypanosoma_cruzi_marinkellei
Trypanosoma_brucei__8954
EPY41219_1_ATPbinding_protein_involved_in_chromosome_partitioning____Angomonas_deanei
Leishmania_major__4520
Bodo_saltans__3526
AGG11500_1_hypothetical_protein_partial____Paratrypanosoma_confusum
OGW96150_1_hypothetical_protein_A2Z81_09390____Omnitrophica_WOR_2_bacterium_GWA2_45_18
Gregarina_niphandrodes__4272
Babesia_bigeminaBOND__54305
Babesia_bigeminaBOND__54308
Babesia_bovisT2Bo__29433
Theileria_equiWA__39150
Babesia_bovisT2Bo__29421
Babesia_microtiRI__31113
Babesia_microtiRI__31112
Theileria_equiWA__39118
Bigelowiella_natans__11564
Plasmodiophora_brassicae__9665
XP_002785765_1_polysaccharide_export_protein_putative____Perkinsus_marinus_ATCC_50983
Perkinsus_marinus__17970
CEM38581_1_unnamed_protein_product____Vitrella_brassicaformis_CCMP3155
Vitrella_brassicaformis__22176
Alphamonas_edax__37631
Alphamonas_edax__37645
Chromera_velia__11909
Digyalum_oweni1__5272
Digyalum_oweni2__1259
Piridium__3463
Piridium__3703
Eimeria_brunettiHoughton__471707
Eimeria_falciformisBayerHaberkorn1970__62622
Eimeria_tenellaHoughton__497915
Colpodella_angusta1__28108
Colpodella_angusta2__57249
Colpodella_angusta1__28109
Colpodella_angusta1__58542
Nephromyces_occidentalis__11878
Nephromyces_occidentalis__39463
Nephromyces_occidentalis__39465
Nephromyces_occidentalis__39464
Nephromyces_occidentalis__30566
Nephromyces_occidentalis__85487
Nephromyces_occidentalis__37330
Nephromyces_occidentalis__70615
Nephromyces_occidentalis__55827
Eimeria_brunettiHoughton__464448
Eimeria_falciformisBayerHaberkorn1970__59174
Eimeria_tenellaHoughton__497966
Eimeria_brunettiHoughton__468115
Sarcocystis_neuronaSN3__611841
Eimeria_falciformisBayerHaberkorn1970__61396
Eimeria_tenellaHoughton__497946
Hammondia_hammondiHH34__442249
Hammondia_hammondiHH34__443189
Toxoplasma_gondii__3122
Toxoplasma_gondii__4372
Sarcocystis_neuronaSN3__609542
Hammondia_hammondiHH34__442517
Neospora_caninumLIV__122035
Neospora_caninumLIV__122039
Hammondia_hammondiHH34__442778
Neospora_caninumLIV__122027
Neospora_caninumLIV__122057
Sarcocystis_neuronaSN3__610961
Sarcocystis_neuronaSOSN1__815210
Selenidium_pygospionis__1520
Cephaloidophora_communis__1514
Heliospora_caprellae__2116
Cryptosporidium_andersoni30847__13450
Cryptosporidium_murisRN66__25569
Cryptosporidium_baileyiTAMU_09Q1__19113
Cryptosporidium_hominis30976__19976
Cryptosporidium_hominisUdeA01__16665
Cryptosporidium_hominisUKH1__13610
Cryptosporidium_hominisTU502_2012__10930
Cryptosporidium_hominis37999__6583
Cryptosporidium_parvumIOWA_ATCC__16853
Cryptosporidium_parvumIowaII__16627
Cryptosporidium_tyzzeriUGA55__16289
Cryptosporidium_meleagridisUKMEL1__9807
Cryptosporidium_ubiquitum39726__18574
Cryptosporidium_spchipmunkLX2015__33739
Protomagalhaensia_sp_Gyna_25__4629
Protomagalhaensia_sp_Gyna_26__1899
Protomagalhaensia_wolfi__2081
Protomagalhaensia_wolfi__2082
Gregarina_sp_Poly_1_total__8507
Blabericola_migrator_1_total__1840
Blabericola_migrator_2_total__176
Gregarina_sp_Pseudo_7__1640
Gregarina_sp_Pseudo_9__325
Gregarina_sp_Pseudo_7__1641
Lecudina_tuzetae__20649
Pterospora_schizosoma__456
Monocystis_agilis__20767
Monocystis_agilis__20766
Pterospora_schizosoma__707
Pterospora_schizosoma__9422
Pterospora_schizosoma__9423
Sarcocystis_neuronaSN3__608406
Sarcocystis_neuronaSOSN1__817830
OLP98402_1_Ironsulfur_protein_IND1____Symbiodinium_microadriaticum
Symbiodinium_microadriaticum__25700
Oxyrrhis_marina__9609
Haemoproteus_tartakovskyi__3833
Plasmodium_falciparum3D7__3126
Plasmodium_bergheiANKA__29416
Plasmodium_chabaudi__33954
Alphamonas_edax__48035
Alphamonas_edax__48036
Karenia_brevis__100211
Oxyrrhis_marina__51603
XP_005776414_1_mrp_protein____Emiliania_huxleyi_CCMP1516
Emiliania_huxleyi__17803
Babesia_bigeminaBOND__54309
Theileria_equiWA__39154
Babesia_bovisT2Bo__29441
Strombidinopsis_acuminatum___StromAcum_61219
Thalassiosira_pseudonana__4294
Phaeodactylum_tricornutum__7944
Emiliania_huxleyi__28147
Eimeria_brunettiHoughton__461258
Eimeria_tenellaHoughton__498015
Eimeria_falciformisBayerHaberkorn1970__57724
Goniomonas_avonlea__2526
Blastocystis_hominis__2777
EDM25315_1_hypothetical_protein_LNTAR_03519____Lentisphaera_araneosa_HTCC2155
XP_002293925_1_predicted_protein_partial____Thalassiosira_pseudonana_CCMP1335
Thalassiosira_pseudonana__7965
OEU07846_1_Ploop_containing_nucleoside_triphosphate_hydrolase_protein____Fragilariopsis_cylindrus_CCMP1102
Phaeodactylum_tricornutum__10133
Vitrella_brassicaformis__5511
XP_005791179_1_hypothetical_protein_EMIHUDRAFT_70127_partial____Emiliania_huxleyi_CCMP1516
Emiliania_huxleyi__29526
Isochrysis_sp_CCMP1324__15132
Bigelowiella_natans__5102
Guillardia_theta__23248
Chromera_velia__3917
Ectocarpus_siliculosus__460
XP_005538557_1_probable_multidrug_resistance_protein____Cyanidioschyzon_merolae_strain_10D
Cyanidioschyzon_merolae__3744
XP_005704479_1_ATPbinding_protein_involved_in_chromosome_partitioning____Galdieria_sulphuraria
Galdieria_sulphuraria__2130
XP_005711941_1_unnamed_protein_product____Chondrus_crispus
Chondrus_crispus__2048
Cyanophora_paradoxa__2998
XP_005821070_1_hypothetical_protein_GUITHDRAFT_147485_partial____Guillardia_theta_CCMP2712
Guillardia_theta__3241
Goniomonas_avonlea__12704
Cafeteria_sp__15977
ETO22470_1_hypothetical_protein_RFI_14729____Reticulomyxa_filosa
Aurantiochytrium_limacinum__418
Paulinella_chromatophora__35201
ETI34744_1_hypothetical_protein_F443_18814____Phytophthora_parasitica_P1569
Phytophthora_parasitica__20970
XP_008604920_1_hypothetical_protein_SDRG_01182_partial____Saprolegnia_diclina_VS20
Saprolegnia_parasitica__1204
XP_009838179_1_hypothetical_protein_H257_12605____Aphanomyces_astaci
Ectocarpus_siliculosus__10328
Muranothrix_gubernatus___gnl_Murano_sp_16242_2
Goniomonas_avonlea__2912
Stentor_coeruleus__22611
EJY74228_1_ParA_multidomain_protein_macronuclear____Oxytricha_trifallax
Oxytricha_trifallax__4351
Halteria_grandinella___Halteria_123
Strombidinopsis_acuminatum___StromAcum_124052
Protocruzia_adherens___Protocruzi_50069
Litonotus_sp___Litonotus_5863
KRX01966_1_Ploop_containing_nucleoside_triphosphate_hydrolase____Pseudocohnilembus_persalinus
Platyophrya_macrostoma___Platyphrya_39591
Furgasonia_blochmanni___FurgaBloc_57099
KRX09559_1_Ploop_containing_nucleoside_triphosphate_hydrolase____Pseudocohnilembus_persalinus
Tetrahymena_thermophila__709
XP_001007903_1_CobQ_CobB_MinD_ParA_nucleotidebinding_domain_protein____Tetrahymena_thermophila_SB210
Paramecium_tetraurelia___paratetr_67140
XP_001429812_1_hypothetical_protein_macronuclear____Paramecium_tetraurelia_strain_d42
XP_001482196_1_hypothetical_protein_PGUG_05216____Meyerozyma_guilliermondii_ATCC_6260
XP_007375148_1_hypothetical_protein_SPAPADRAFT_136560____Spathaspora_passalidarum_NRRL_Y27907
XP_026587146_1_Ironsulfur_protein_IND1____Candida_viswanathii
CCA37485_1_Ironsulfur_protein____Komagataella_phaffii_CBS_7435
CEP23187_1_Ironsulfur_protein_IND1_AltName_Full_Ironsulfur_protein_required_for_NADH_dehydrogenase_1_Flags_Precursor____Cyberlindnera_jadinii
ONH69759_1_Ironsulfur_protein_IND1____Cyberlindnera_fabianii
XP_020545717_1_Ironsulfur_protein_IND1____Pichia_kudriavzevii
XP_013936744_1_Ironsulfur_protein_IND1____Ogataea_parapolymorpha_DL1
RDB30696_1_Ironsulfur_protein_IND1____Hypsizygus_marmoreus
Coprinus_cinereus__9906
KNZ75991_1_Ironsulfur_protein_IND1_partial____Termitomyces_sp_J132
OBZ77303_1_Ironsulfur_protein_IND1____Grifola_frondosa
XP_027615441_1_Ironsulfur_protein____Sparassis_crispa
KZV87539_1_Ironsulfur_protein_IND1____Exidia_glandulosa_HHB12029
CEL52195_1_Ironsulfur_protein_IND1_OS_Yarrowia_lipolytica_strain_CLIB_122___E_150_GN_IND1_PE_1_SV_1____Rhizoctonia_solani_AG1_IB
Ustilago_maydis__5578
EOR04728_1_Ironsulfur_protein_IND1____Wallemia_ichthyophaga_EXF994
sp_Q6CE48_1_IND1_YARLI_RecName_Full_Ironsulfur_protein_IND1_AltName_Full_Ironsulfur_protein_required_for_NADH_dehydrogenase_1_Flags_Precursor
FeS_IND1
Pygsuia_biforma__2670
0.4

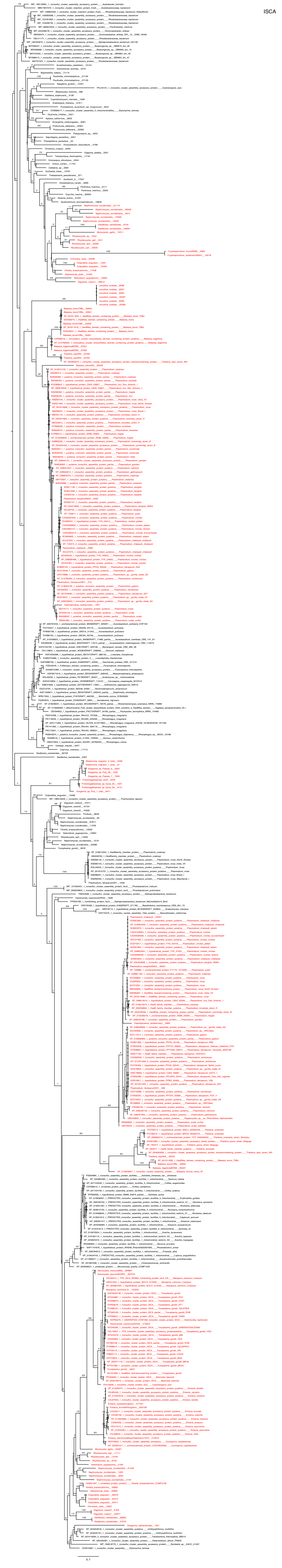
ISCA

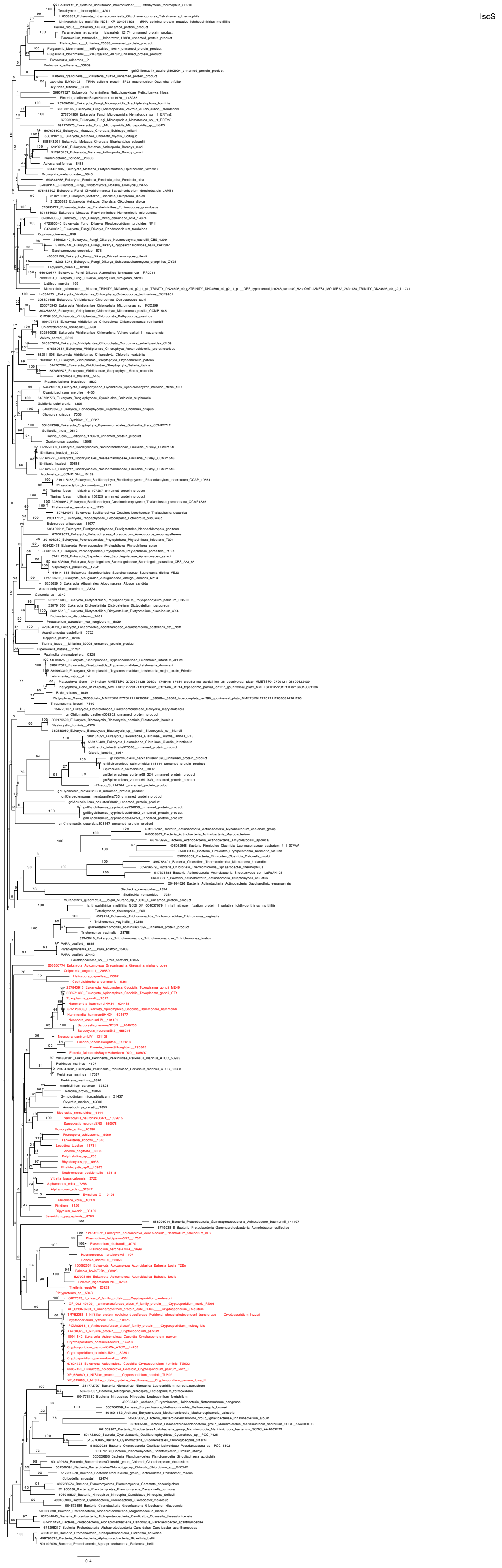
IscS
0.4

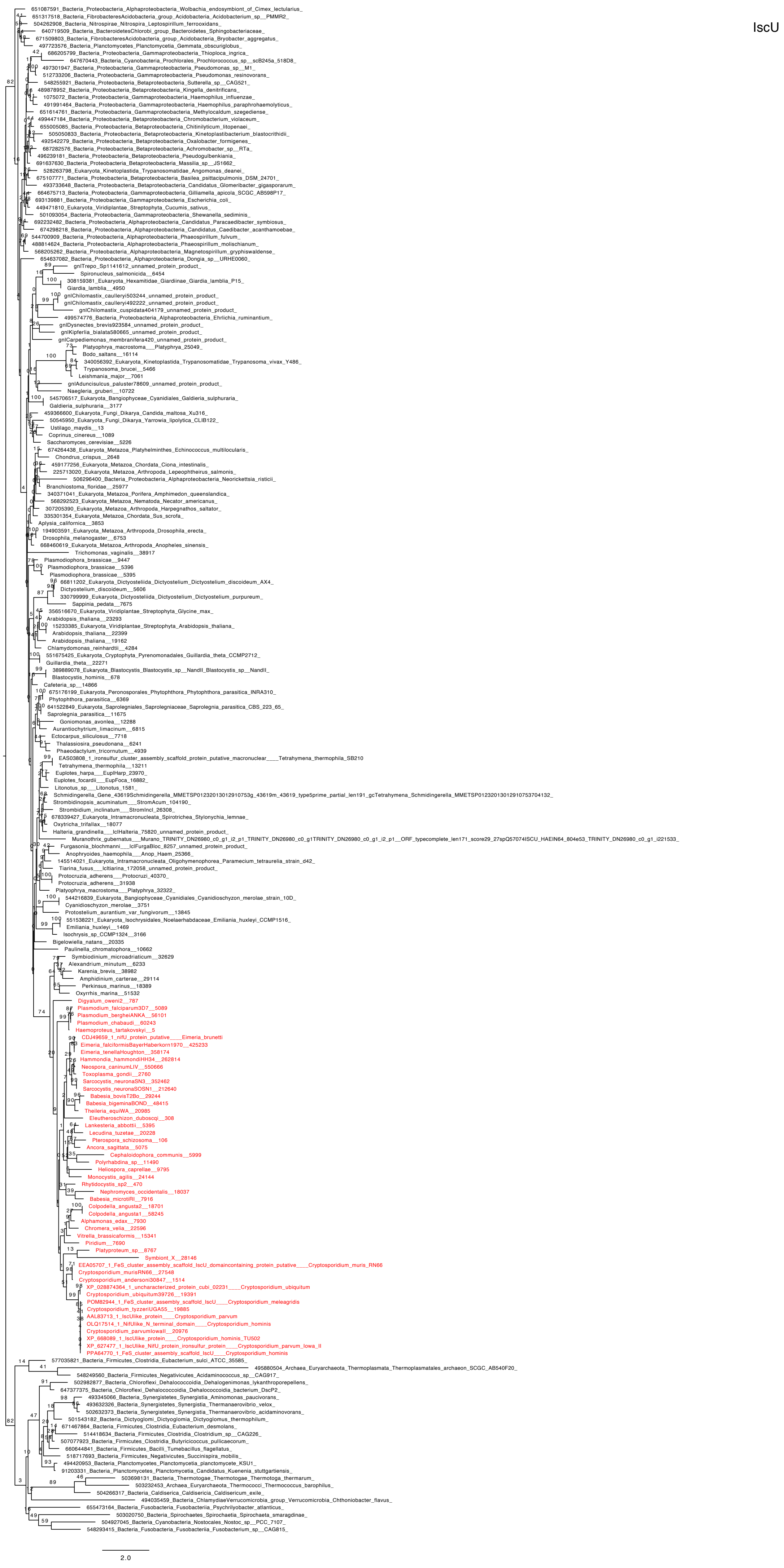
IscU
2.0

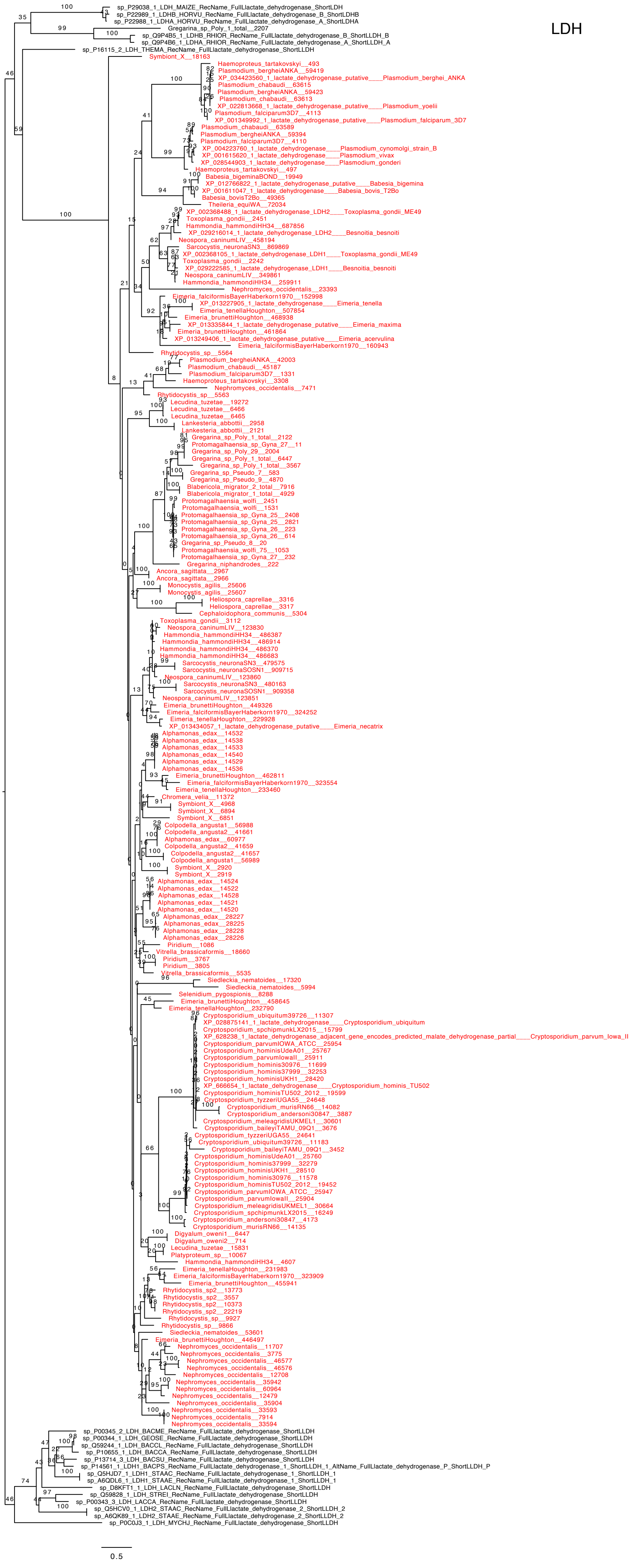
LDH
sp_P29038_1_LDH_MAIZE_RecName_FullLlactate_dehydrogenase_ShortLDH
sp_P22989_1_LDHB_HORVU_RecName_FullLlactate_dehydrogenase_B_ShortLDHB
sp_P22988_1_LDHA_HORVU_RecName_FullLlactate_dehydrogenase_A_ShortLDHA
Gregarina_sp_Poly_1_total__2207
sp_Q9P4B5_1_LDHB_RHIOR_RecName_FullLlactate_dehydrogenase_B_ShortLLDH_B
sp_Q9P4B6_1_LDHA_RHIOR_RecName_FullLlactate_dehydrogenase_A_ShortLLDH_A
sp_P16115_2_LDH_THEMA_RecName_FullLlactate_dehydrogenase_ShortLLDH
Symbiont_X__18163
Haemoproteus_tartakovskyi__493
Plasmodium_bergheiANKA__59419
XP_034423560_1_lactate_dehydrogenase_putative____Plasmodium_berghei_ANKA
Plasmodium_chabaudi__63615
Plasmodium_bergheiANKA__59423
Plasmodium_chabaudi__63613
XP_022813668_1_lactate_dehydrogenase_putative____Plasmodium_yoelii
Plasmodium_falciparum3D7__4113
XP_001349992_1_lactate_dehydrogenase_putative____Plasmodium_falciparum_3D7
Plasmodium_chabaudi__63589
Plasmodium_bergheiANKA__59394
Plasmodium_falciparum3D7__4110
XP_004223760_1_lactate_dehydrogenase____Plasmodium_cynomolgi_strain_B
XP_001615620_1_lactate_dehydrogenase____Plasmodium_vivax
XP_028544903_1_lactate_dehydrogenase____Plasmodium_gonderi
Haemoproteus_tartakovskyi__497
Babesia_bigeminaBOND__19949
XP_012766822_1_lactate_dehydrogenase_putative____Babesia_bigemina
XP_001611047_1_lactate_dehydrogenase____Babesia_bovis_T2Bo
Babesia_bovisT2Bo__49365
Theileria_equiWA__72034
XP_002368488_1_lactate_dehydrogenase_LDH2____Toxoplasma_gondii_ME49
Toxoplasma_gondii__2451
Hammondia_hammondiHH34__687856
XP_029216014_1_lactate_dehydrogenase_LDH2____Besnoitia_besnoiti
Neospora_caninumLIV__458194
Sarcocystis_neuronaSN3__869869
XP_002368105_1_lactate_dehydrogenase_LDH1____Toxoplasma_gondii_ME49
Toxoplasma_gondii__2242
XP_029222585_1_lactate_dehydrogenase_LDH1____Besnoitia_besnoiti
Neospora_caninumLIV__349861
Hammondia_hammondiHH34__259911
Nephromyces_occidentalis__23393
Eimeria_falciformisBayerHaberkorn1970__152998
XP_013227905_1_lactate_dehydrogenase____Eimeria_tenella
Eimeria_tenellaHoughton__507854
Eimeria_brunettiHoughton__468938
XP_013335844_1_lactate_dehydrogenase_putative____Eimeria_maxima
Eimeria_brunettiHoughton__461864
XP_013249406_1_lactate_dehydrogenase_putative____Eimeria_acervulina
Eimeria_falciformisBayerHaberkorn1970__160943
Rhytidocystis_sp__5564
Plasmodium_bergheiANKA__42003
Plasmodium_chabaudi__45187
Plasmodium_falciparum3D7__1331
Haemoproteus_tartakovskyi__3308
Nephromyces_occidentalis__7471
Rhytidocystis_sp__5563
Lecudina_tuzetae__19272
Lecudina_tuzetae__6466
Lecudina_tuzetae__6465
Lankesteria_abbottii__2958
Lankesteria_abbottii__2121
Gregarina_sp_Poly_1_total__2122
Protomagalhaensia_sp_Gyna_27__11
Gregarina_sp_Poly_29__2004
Gregarina_sp_Poly_1_total__6447
Gregarina_sp_Poly_1_total__3567
Gregarina_sp_Pseudo_7__583
Gregarina_sp_Pseudo_9__4870
Blabericola_migrator_2_total__7916
Blabericola_migrator_1_total__4929
Protomagalhaensia_wolfi__2451
Protomagalhaensia_wolfi__1531
Protomagalhaensia_sp_Gyna_25__2408
Protomagalhaensia_sp_Gyna_25__2821
Protomagalhaensia_sp_Gyna_26__223
Protomagalhaensia_sp_Gyna_26__614
Gregarina_sp_Pseudo_8__20
Protomagalhaensia_wolfi_75__1053
Protomagalhaensia_sp_Gyna_27__232
Gregarina_niphandrodes__222
Ancora_sagittata__2967
Ancora_sagittata__2966
Monocystis_agilis__25606
Monocystis_agilis__25607
Heliospora_caprellae__3316
Heliospora_caprellae__3317
Cephaloidophora_communis__5304
Toxoplasma_gondii__3112
Neospora_caninumLIV__123830
Hammondia_hammondiHH34__486387
Hammondia_hammondiHH34__486914
Hammondia_hammondiHH34__486370
Hammondia_hammondiHH34__486683
Sarcocystis_neuronaSN3__479575
Sarcocystis_neuronaSOSN1__909715
Neospora_caninumLIV__123860
Sarcocystis_neuronaSN3__480163
Sarcocystis_neuronaSOSN1__909358
Neospora_caninumLIV__123851
Eimeria_brunettiHoughton__449326
Eimeria_falciformisBayerHaberkorn1970__324252
Eimeria_tenellaHoughton__229928
XP_013434057_1_lactate_dehydrogenase_putative____Eimeria_necatrix
Alphamonas_edax__14532
Alphamonas_edax__14538
Alphamonas_edax__14533
Alphamonas_edax__14540
Alphamonas_edax__14529
Alphamonas_edax__14536
Eimeria_brunettiHoughton__462811
Eimeria_falciformisBayerHaberkorn1970__323554
Eimeria_tenellaHoughton__233460
Chromera_velia__11372
Symbiont_X__4968
Symbiont_X__6894
Symbiont_X__6851
Colpodella_angusta1__56988
Colpodella_angusta2__41661
Alphamonas_edax__60977
Colpodella_angusta2__41659
Colpodella_angusta2__41657
Colpodella_angusta1__56989
Symbiont_X__2920
Symbiont_X__2919
Alphamonas_edax__14524
Alphamonas_edax__14522
Alphamonas_edax__14528
Alphamonas_edax__14521
Alphamonas_edax__14520
Alphamonas_edax__28227
Alphamonas_edax__28225
Alphamonas_edax__28228
Alphamonas_edax__28226
Piridium__1086
Vitrella_brassicaformis__18660
Piridium__3767
Piridium__3805
Vitrella_brassicaformis__5535
Siedleckia_nematoides__17320
Siedleckia_nematoides__5994
Selenidium_pygospionis__8288
Eimeria_brunettiHoughton__458645
Eimeria_tenellaHoughton__232790
Cryptosporidium_ubiquitum39726__11307
XP_028875141_1_lactate_dehydrogenase____Cryptosporidium_ubiquitum
Cryptosporidium_spchipmunkLX2015__15799
XP_628238_1_lactate_dehydrogenase_adjacent_gene_encodes_predicted_malate_dehydrogenase_partial____Cryptosporidium_parvum_Iowa_II
Cryptosporidium_parvumIOWA_ATCC__25954
Cryptosporidium_hominisUdeA01__25767
Cryptosporidium_parvumIowaII__25911
Cryptosporidium_hominis30976__11699
Cryptosporidium_hominis37999__32253
Cryptosporidium_hominisUKH1__28420
XP_666654_1_lactate_dehydrogenase____Cryptosporidium_hominis_TU502
Cryptosporidium_hominisTU502_2012__19599
Cryptosporidium_tyzzeriUGA55__24648
Cryptosporidium_murisRN66__14082
Cryptosporidium_andersoni30847__3887
Cryptosporidium_meleagridisUKMEL1__30601
Cryptosporidium_baileyiTAMU_09Q1__3676
Cryptosporidium_tyzzeriUGA55__24641
Cryptosporidium_ubiquitum39726__11183
Cryptosporidium_baileyiTAMU_09Q1__3452
Cryptosporidium_hominisUdeA01__25760
Cryptosporidium_hominis37999__32279
Cryptosporidium_hominisUKH1__28510
Cryptosporidium_hominis30976__11578
Cryptosporidium_hominisTU502_2012__19452
Cryptosporidium_parvumIOWA_ATCC__25947
Cryptosporidium_parvumIowaII__25904
Cryptosporidium_meleagridisUKMEL1__30664
Cryptosporidium_spchipmunkLX2015__16249
Cryptosporidium_andersoni30847__4173
Cryptosporidium_murisRN66__14135
Digyalum_oweni1__6447
Digyalum_oweni2__714
Lecudina_tuzetae__15831
Platyproteum_sp__10067
Hammondia_hammondiHH34__4607
Eimeria_tenellaHoughton__231983
Eimeria_falciformisBayerHaberkorn1970__323909
Eimeria_brunettiHoughton__455941
Rhytidocystis_sp2__13773
Rhytidocystis_sp2__3557
Rhytidocystis_sp2__10373
Rhytidocystis_sp2__22219
Rhytidocystis_sp__9927
Rhytidocystis_sp__9866
Siedleckia_nematoides__53601
Eimeria_brunettiHoughton__446497
Nephromyces_occidentalis__11707
Nephromyces_occidentalis__3775
Nephromyces_occidentalis__46577
Nephromyces_occidentalis__46576
Nephromyces_occidentalis__12708
Nephromyces_occidentalis__35942
Nephromyces_occidentalis__60964
Nephromyces_occidentalis__12479
Nephromyces_occidentalis__35804
Nephromyces_occidentalis__33593
Nephromyces_occidentalis__7914
Nephromyces_occidentalis__33594
sp_P00345_2_LDH_BACME_RecName_FullLlactate_dehydrogenase_ShortLLDH
sp_P00344_1_LDH_GEOSE_RecName_FullLlactate_dehydrogenase_ShortLLDH
sp_Q59244_1_LDH_BACCL_RecName_FullLlactate_dehydrogenase_ShortLLDH
sp_P10655_1_LDH_BACCA_RecName_FullLlactate_dehydrogenase_ShortLLDH
sp_P13714_3_LDH_BACSU_RecName_FullLlactate_dehydrogenase_ShortLLDH
sp_P14561_1_LDH1_BACPS_RecName_FullLlactate_dehydrogenase_1_ShortLLDH_1_AltName_FullLlactate_dehydrogenase_P_ShortLLDH_P
sp_Q5HJD7_1_LDH1_STAAC_RecName_FullLlactate_dehydrogenase_1_ShortLLDH_1
sp_A6QDL6_1_LDH1_STAAE_RecName_FullLlactate_dehydrogenase_1_ShortLLDH_1
sp_D8KFT1_1_LDH_LACLN_RecName_FullLlactate_dehydrogenase_ShortLLDH
sp_Q59828_1_LDH_STREI_RecName_FullLlactate_dehydrogenase_ShortLLDH
sp_P00343_3_LDH_LACCA_RecName_FullLlactate_dehydrogenase_ShortLLDH
sp_Q5HCV0_1_LDH2_STAAC_RecName_FullLlactate_dehydrogenase_2_ShortLLDH_2
sp_A6QK89_1_LDH2_STAAE_RecName_FullLlactate_dehydrogenase_2_ShortLLDH_2
sp_P0C0J3_1_LDH_MYCHJ_RecName_FullLlactate_dehydrogenase_ShortLLDH
0.5

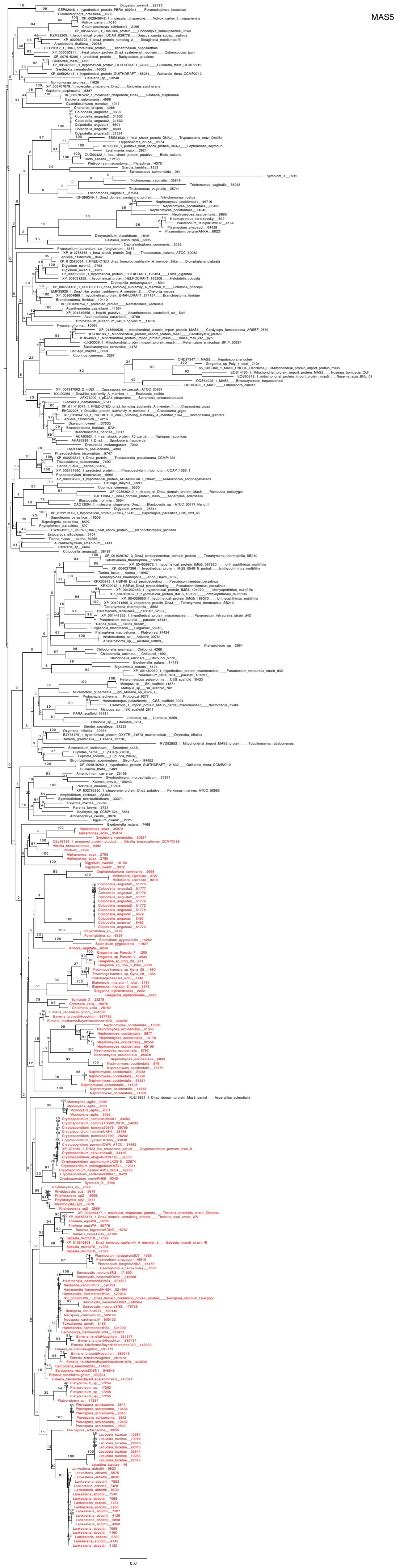
MAS5
0.6

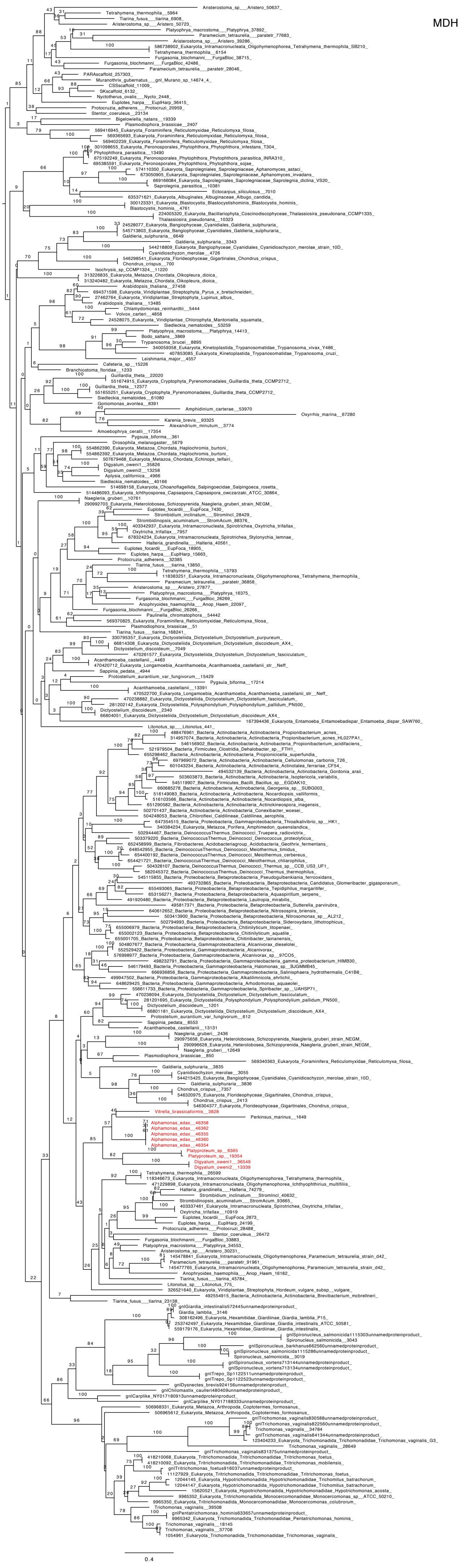
MDH
0.4

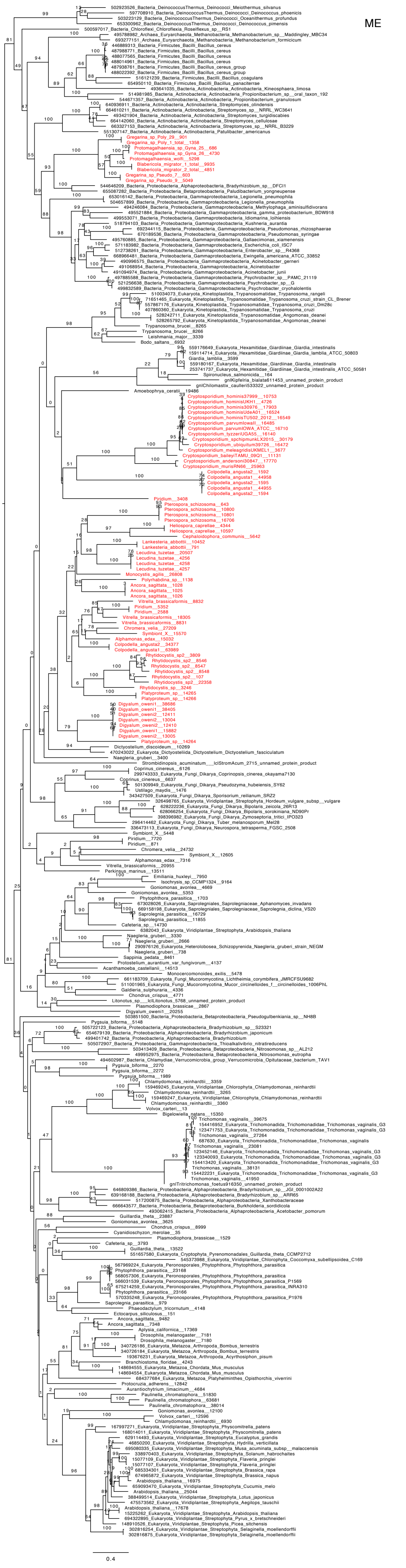
ME
0.4

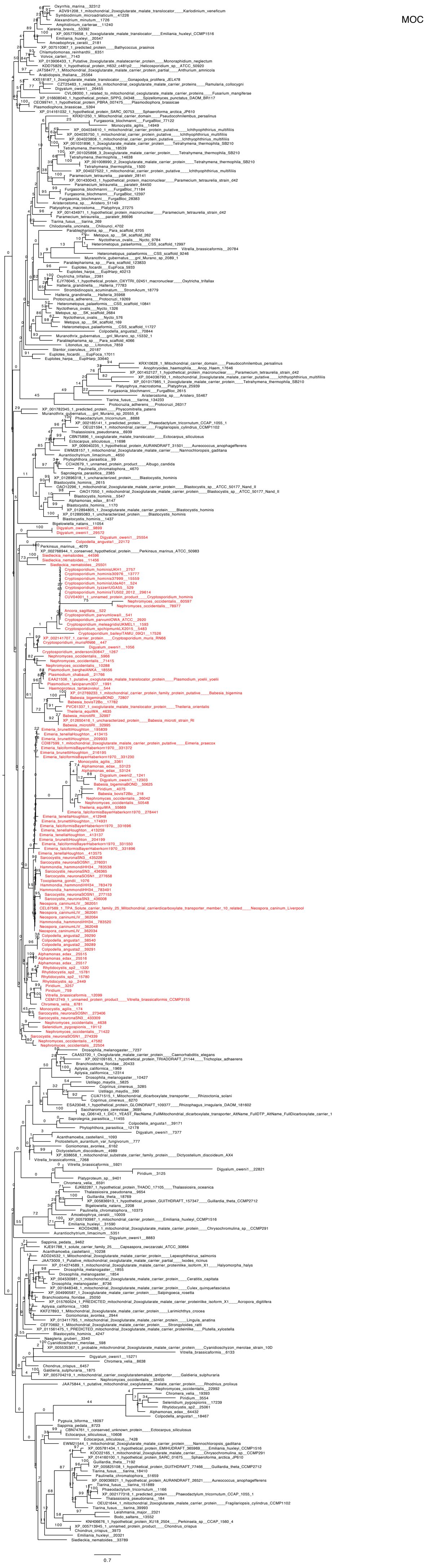
MOC
0.7

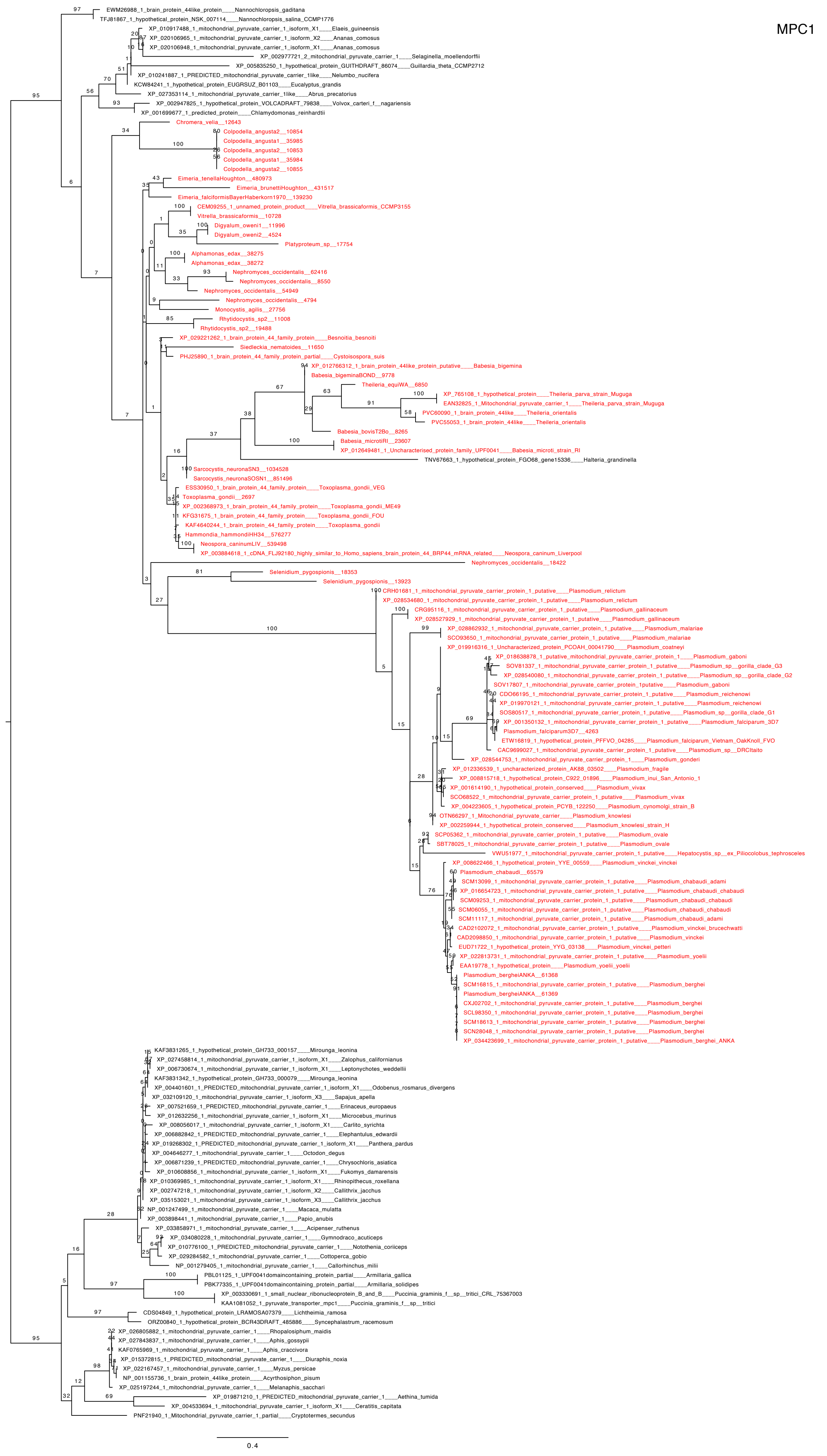
MPC1
EWM26988_1_brain_protein_44like_protein____Nannochloropsis_gaditana
TFJ81867_1_hypothetical_protein_NSK_007114____Nannochloropsis_salina_CCMP1776
XP_010917488_1_mitochondrial_pyruvate_carrier_1_isoform_X1____Elaeis_guineensis
XP_020106965_1_mitochondrial_pyruvate_carrier_1_isoform_X2____Ananas_comosus
XP_020106948_1_mitochondrial_pyruvate_carrier_1_isoform_X1____Ananas_comosus
XP_002977721_2_mitochondrial_pyruvate_carrier_1____Selaginella_moellendorffii
XP_005835250_1_hypothetical_protein_GUITHDRAFT_86074____Guillardia_theta_CCMP2712
XP_010241887_1_PREDICTED_mitochondrial_pyruvate_carrier_1like____Nelumbo_nucifera
KCW84241_1_hypothetical_protein_EUGRSUZ_B01103____Eucalyptus_grandis
XP_027353114_1_mitochondrial_pyruvate_carrier_1like____Abrus_precatorius
XP_002947825_1_hypothetical_protein_VOLCADRAFT_79838____Volvox_carteri_f__nagariensis
XP_001699677_1_predicted_protein____Chlamydomonas_reinhardtii
Chromera_velia__12643
Colpodella_angusta2__10854
Colpodella_angusta1__35985
Colpodella_angusta2__10853
Colpodella_angusta1__35984
Colpodella_angusta2__10855
Eimeria_tenellaHoughton__480973
Eimeria_brunettiHoughton__431517
Eimeria_falciformisBayerHaberkorn1970__139230
CEM09255_1_unnamed_protein_product____Vitrella_brassicaformis_CCMP3155
Vitrella_brassicaformis__10728
Digyalum_oweni1__11996
Digyalum_oweni2__4524
Platyproteum_sp__17754
Alphamonas_edax__38275
Alphamonas_edax__38272
Nephromyces_occidentalis__62416
Nephromyces_occidentalis__8550
Nephromyces_occidentalis__54949
Nephromyces_occidentalis__4794
Monocystis_agilis__27756
Rhytidocystis_sp2__11008
Rhytidocystis_sp2__19488
XP_029221262_1_brain_protein_44_family_protein____Besnoitia_besnoiti
Siedleckia_nematoides__11650
PHJ25890_1_brain_protein_44_family_protein_partial____Cystoisospora_suis
XP_012766312_1_brain_protein_44like_protein_putative____Babesia_bigemina
Babesia_bigeminaBOND__9778
Theileria_equiWA__6850
XP_765108_1_hypothetical_protein____Theileria_parva_strain_Muguga
EAN32825_1_Mitochondrial_pyruvate_carrier_1____Theileria_parva_strain_Muguga
PVC60090_1_brain_protein_44like____Theileria_orientalis
PVC55053_1_brain_protein_44like____Theileria_orientalis
Babesia_bovisT2Bo__8265
Babesia_microtiRI__23607
XP_012649481_1_Uncharacterised_protein_family_UPF0041____Babesia_microti_strain_RI
TNV67663_1_hypothetical_protein_FGO68_gene15336____Halteria_grandinella
Sarcocystis_neuronaSN3__1034528
Sarcocystis_neuronaSOSN1__851496
ESS30950_1_brain_protein_44_family_protein____Toxoplasma_gondii_VEG
Toxoplasma_gondii__2697
XP_002368973_1_brain_protein_44_family_protein____Toxoplasma_gondii_ME49
KFG31675_1_brain_protein_44_family_protein____Toxoplasma_gondii_FOU
KAF4640244_1_brain_protein_44_family_protein____Toxoplasma_gondii
Hammondia_hammondiHH34__576277
Neospora_caninumLIV__539498
XP_003884618_1_cDNA_FLJ92180_highly_similar_to_Homo_sapiens_brain_protein_44_BRP44_mRNA_related____Neospora_caninum_Liverpool
Nephromyces_occidentalis__18422
Selenidium_pygospionis__18353
Selenidium_pygospionis__13923
CRH01681_1_mitochondrial_pyruvate_carrier_protein_1_putative____Plasmodium_relictum
XP_028534680_1_mitochondrial_pyruvate_carrier_protein_1_putative____Plasmodium_relictum
CRG95116_1_mitochondrial_pyruvate_carrier_protein_1_putative____Plasmodium_gallinaceum
XP_028527929_1_mitochondrial_pyruvate_carrier_protein_1_putative____Plasmodium_gallinaceum
XP_028862932_1_mitochondrial_pyruvate_carrier_protein_1_putative____Plasmodium_malariae
SCO93650_1_mitochondrial_pyruvate_carrier_protein_1_putative____Plasmodium_malariae
XP_019916316_1_Uncharacterized_protein_PCOAH_00041790____Plasmodium_coatneyi
XP_018638878_1_putative_mitochondrial_pyruvate_carrier_protein_1____Plasmodium_gaboni
SOV81337_1_mitochondrial_pyruvate_carrier_protein_1_putative____Plasmodium_sp__gorilla_clade_G3
XP_028540080_1_mitochondrial_pyruvate_carrier_protein_1_putative____Plasmodium_sp__gorilla_clade_G2
SOV17807_1_mitochondrial_pyruvate_carrier_protein_1putative____Plasmodium_gaboni
CDO66195_1_mitochondrial_pyruvate_carrier_protein_1_putative____Plasmodium_reichenowi
XP_019970121_1_mitochondrial_pyruvate_carrier_protein_1_putative____Plasmodium_reichenowi
SOS80517_1_mitochondrial_pyruvate_carrier_protein_1_putative____Plasmodium_sp__gorilla_clade_G1
XP_001350132_1_mitochondrial_pyruvate_carrier_protein_1_putative____Plasmodium_falciparum_3D7
Plasmodium_falciparum3D7__4263
ETW16819_1_hypothetical_protein_PFFVO_04285____Plasmodium_falciparum_Vietnam_OakKnoll_FVO
CAC9699027_1_mitochondrial_pyruvate_carrier_protein_1_putative____Plasmodium_sp__DRC1taito
XP_028544753_1_mitochondrial_pyruvate_carrier_protein_1____Plasmodium_gonderi
XP_012336539_1_uncharacterized_protein_AK88_03502____Plasmodium_fragile
XP_008815718_1_hypothetical_protein_C922_01896____Plasmodium_inui_San_Antonio_1
XP_001614190_1_hypothetical_protein_conserved____Plasmodium_vivax
SCO68522_1_mitochondrial_pyruvate_carrier_protein_1_putative____Plasmodium_vivax
XP_004223605_1_hypothetical_protein_PCYB_122250____Plasmodium_cynomolgi_strain_B
OTN66297_1_Mitochondrial_pyruvate_carrier____Plasmodium_knowlesi
XP_002259944_1_hypothetical_protein_conserved____Plasmodium_knowlesi_strain_H
SCP05362_1_mitochondrial_pyruvate_carrier_protein_1_putative____Plasmodium_ovale
SBT78025_1_mitochondrial_pyruvate_carrier_protein_1_putative____Plasmodium_ovale
VWU51977_1_mitochondrial_pyruvate_carrier_protein_1_putative____Hepatocystis_sp__ex_Piliocolobus_tephrosceles
XP_008622466_1_hypothetical_protein_YYE_00559____Plasmodium_vinckei_vinckei
Plasmodium_chabaudi__65579
SCM13099_1_mitochondrial_pyruvate_carrier_protein_1_putative____Plasmodium_chabaudi_adami
XP_016654723_1_mitochondrial_pyruvate_carrier_protein_1_putative____Plasmodium_chabaudi_chabaudi
SCM09253_1_mitochondrial_pyruvate_carrier_protein_1_putative____Plasmodium_chabaudi_chabaudi
SCM06055_1_mitochondrial_pyruvate_carrier_protein_1_putative____Plasmodium_chabaudi_chabaudi
SCM11117_1_mitochondrial_pyruvate_carrier_protein_1_putative____Plasmodium_chabaudi_adami
CAD2102072_1_mitochondrial_pyruvate_carrier_protein_1_putative____Plasmodium_vinckei_brucechwatti
CAD2098850_1_mitochondrial_pyruvate_carrier_protein_1_putative____Plasmodium_vinckei
EUD71722_1_hypothetical_protein_YYG_03138____Plasmodium_vinckei_petteri
XP_022813731_1_mitochondrial_pyruvate_carrier_protein_1_putative____Plasmodium_yoelii
EAA19778_1_hypothetical_protein____Plasmodium_yoelii_yoelii
Plasmodium_bergheiANKA__61368
SCM16815_1_mitochondrial_pyruvate_carrier_protein_1_putative____Plasmodium_berghei
Plasmodium_bergheiANKA__61369
CXJ02702_1_mitochondrial_pyruvate_carrier_protein_1_putative____Plasmodium_berghei
SCL98350_1_mitochondrial_pyruvate_carrier_protein_1_putative____Plasmodium_berghei
SCM18613_1_mitochondrial_pyruvate_carrier_protein_1_putative____Plasmodium_berghei
SCN28048_1_mitochondrial_pyruvate_carrier_protein_1_putative____Plasmodium_berghei
XP_034423699_1_mitochondrial_pyruvate_carrier_protein_1_putative____Plasmodium_berghei_ANKA
KAF3831265_1_hypothetical_protein_GH733_000157____Mirounga_leonina
XP_027458814_1_mitochondrial_pyruvate_carrier_1_isoform_X1____Zalophus_californianus
XP_006730674_1_mitochondrial_pyruvate_carrier_1_isoform_X1____Leptonychotes_weddellii
KAF3831342_1_hypothetical_protein_GH733_000079____Mirounga_leonina
XP_004401601_1_PREDICTED_mitochondrial_pyruvate_carrier_1_isoform_X1____Odobenus_rosmarus_divergens
XP_032109120_1_mitochondrial_pyruvate_carrier_1_isoform_X3____Sapajus_apella
XP_007521659_1_PREDICTED_mitochondrial_pyruvate_carrier_1____Erinaceus_europaeus
XP_012632256_1_mitochondrial_pyruvate_carrier_1_isoform_X1____Microcebus_murinus
XP_008056017_1_mitochondrial_pyruvate_carrier_1_isoform_X1____Carlito_syrichta
XP_006882842_1_PREDICTED_mitochondrial_pyruvate_carrier_1____Elephantulus_edwardii
XP_019268302_1_PREDICTED_mitochondrial_pyruvate_carrier_1_isoform_X1____Panthera_pardus
XP_004646277_1_mitochondrial_pyruvate_carrier_1____Octodon_degus
XP_006871239_1_PREDICTED_mitochondrial_pyruvate_carrier_1____Chrysochloris_asiatica
XP_010608856_1_mitochondrial_pyruvate_carrier_1_isoform_X1____Fukomys_damarensis
XP_010369985_1_mitochondrial_pyruvate_carrier_1_isoform_X1____Rhinopithecus_roxellana
XP_002747218_1_mitochondrial_pyruvate_carrier_1_isoform_X2____Callithrix_jacchus
XP_035153021_1_mitochondrial_pyruvate_carrier_1_isoform_X3____Callithrix_jacchus
NP_001247499_1_mitochondrial_pyruvate_carrier_1____Macaca_mulatta
XP_003898441_1_mitochondrial_pyruvate_carrier_1____Papio_anubis
XP_033858971_1_mitochondrial_pyruvate_carrier_1____Acipenser_ruthenus
XP_034080228_1_mitochondrial_pyruvate_carrier_1____Gymnodraco_acuticeps
XP_010776100_1_PREDICTED_mitochondrial_pyruvate_carrier_1____Notothenia_coriiceps
XP_029284582_1_mitochondrial_pyruvate_carrier_1____Cottoperca_gobio
NP_001279405_1_mitochondrial_pyruvate_carrier_1____Callorhinchus_milii
PBL01125_1_UPF0041domaincontaining_protein_partial____Armillaria_gallica
PBK77335_1_UPF0041domaincontaining_protein_partial____Armillaria_solidipes
XP_003330691_1_small_nuclear_ribonucleoprotein_B_and_B____Puccinia_graminis_f__sp__tritici_CRL_75367003
KAA1081052_1_pyruvate_transporter_mpc1____Puccinia_graminis_f__sp__tritici
CDS04849_1_hypothetical_protein_LRAMOSA07379____Lichtheimia_ramosa
ORZ00840_1_hypothetical_protein_BCR43DRAFT_485886____Syncephalastrum_racemosum
XP_026805882_1_mitochondrial_pyruvate_carrier_1____Rhopalosiphum_maidis
XP_027843837_1_mitochondrial_pyruvate_carrier_1____Aphis_gossypii
KAF0765969_1_mitochondrial_pyruvate_carrier_1____Aphis_craccivora
XP_015372815_1_PREDICTED_mitochondrial_pyruvate_carrier_1____Diuraphis_noxia
XP_022167457_1_mitochondrial_pyruvate_carrier_1____Myzus_persicae
NP_001155736_1_brain_protein_44like_protein____Acyrthosiphon_pisum
XP_025197244_1_mitochondrial_pyruvate_carrier_1____Melanaphis_sacchari
XP_019871210_1_PREDICTED_mitochondrial_pyruvate_carrier_1____Aethina_tumida
XP_004533694_1_mitochondrial_pyruvate_carrier_1_isoform_X1____Ceratitis_capitata
PNF21940_1_Mitochondrial_pyruvate_carrier_1_partial____Cryptotermes_secundus
0.4

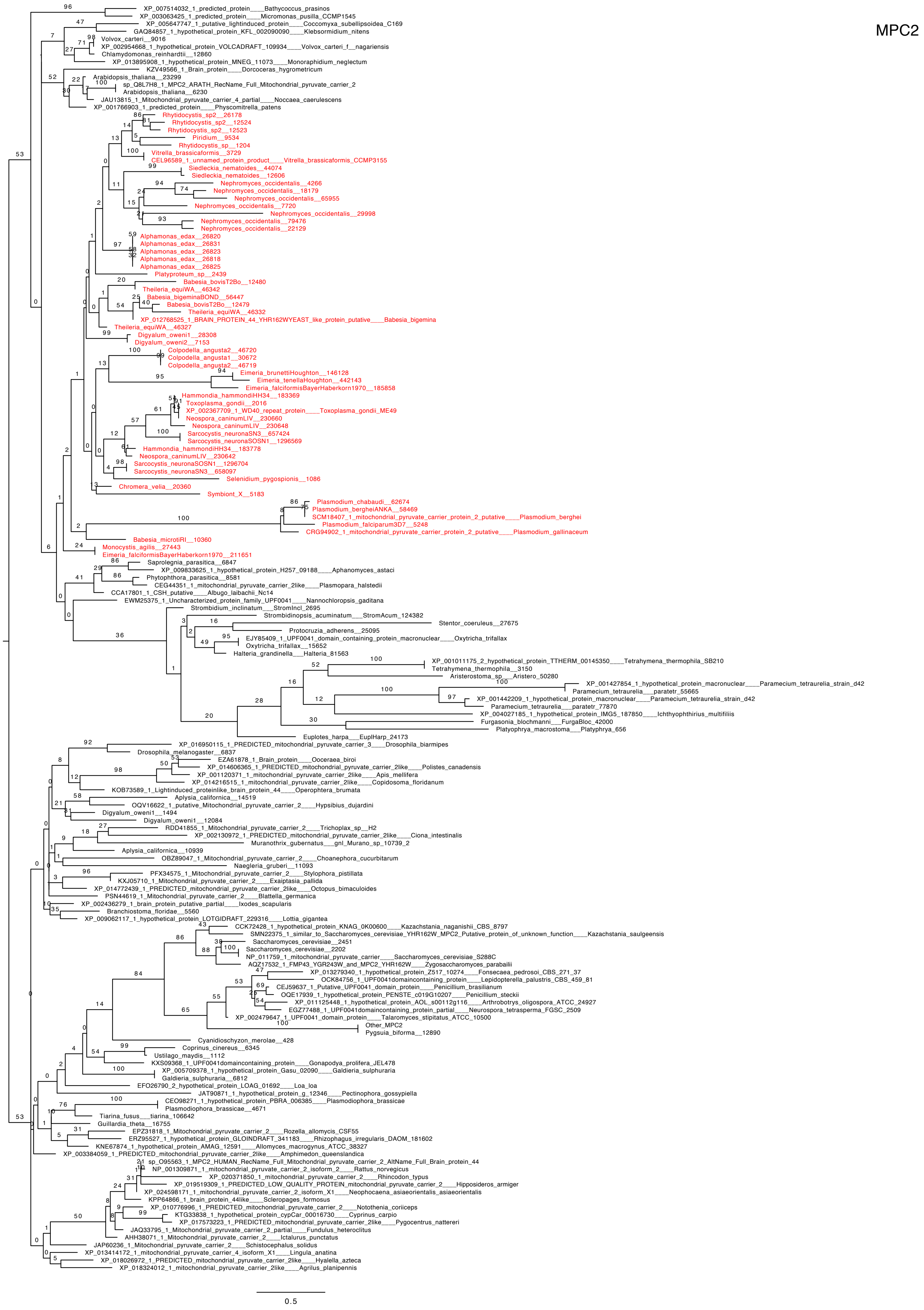
MPC2
XP_007514032_1_predicted_protein____Bathycoccus_prasinos
XP_003063425_1_predicted_protein____Micromonas_pusilla_CCMP1545
XP_005647747_1_putative_lightinduced_protein____Coccomyxa_subellipsoidea_C169
GAQ84857_1_hypothetical_protein_KFL_002090090____Klebsormidium_nitens
Volvox_carteri__9016
XP_002954668_1_hypothetical_protein_VOLCADRAFT_109934____Volvox_carteri_f__nagariensis
Chlamydomonas_reinhardtii__12860
XP_013895908_1_hypothetical_protein_MNEG_11073____Monoraphidium_neglectum
KZV49566_1_Brain_protein____Dorcoceras_hygrometricum
Arabidopsis_thaliana__23299
sp_Q8L7H8_1_MPC2_ARATH_RecName_Full_Mitochondrial_pyruvate_carrier_2
Arabidopsis_thaliana__6230
JAU13815_1_Mitochondrial_pyruvate_carrier_4_partial____Noccaea_caerulescens
XP_001766903_1_predicted_protein____Physcomitrella_patens
Rhytidocystis_sp2__26178
Rhytidocystis_sp2__12524
Rhytidocystis_sp2__12523
Piridium__9534
Rhytidocystis_sp__1204
Vitrella_brassicaformis__3729
CEL96589_1_unnamed_protein_product____Vitrella_brassicaformis_CCMP3155
Siedleckia_nematoides__44074
Siedleckia_nematoides__12606
Nephromyces_occidentalis__4266
Nephromyces_occidentalis__18179
Nephromyces_occidentalis__65955
Nephromyces_occidentalis__7720
Nephromyces_occidentalis__29998
Nephromyces_occidentalis__79476
Nephromyces_occidentalis__22129
Alphamonas_edax__26820
Alphamonas_edax__26831
Alphamonas_edax__26823
Alphamonas_edax__26818
Alphamonas_edax__26825
Platyproteum_sp__2439
Babesia_bovisT2Bo__12480
Theileria_equiWA__46342
Babesia_bigeminaBOND__56447
Babesia_bovisT2Bo__12479
Theileria_equiWA__46332
XP_012768525_1_BRAIN_PROTEIN_44_YHR162WYEAST_like_protein_putative____Babesia_bigemina
Theileria_equiWA__46327
Digyalum_oweni1__28308
Digyalum_oweni2__7153
Colpodella_angusta2__46720
Colpodella_angusta1__30672
Colpodella_angusta2__46719
Eimeria_brunettiHoughton__146128
Eimeria_tenellaHoughton__442143
Eimeria_falciformisBayerHaberkorn1970__185858
Hammondia_hammondiHH34__183369
Toxoplasma_gondii__2016
XP_002367709_1_WD40_repeat_protein____Toxoplasma_gondii_ME49
Neospora_caninumLIV__230660
Neospora_caninumLIV__230648
Sarcocystis_neuronaSN3__657424
Sarcocystis_neuronaSOSN1__1296569
Hammondia_hammondiHH34__183778
Neospora_caninumLIV__230642
Sarcocystis_neuronaSOSN1__1296704
Sarcocystis_neuronaSN3__658097
Selenidium_pygospionis__1086
Chromera_velia__20360
Symbiont_X__5183
Plasmodium_chabaudi__62674
Plasmodium_bergheiANKA__58469
SCM18407_1_mitochondrial_pyruvate_carrier_protein_2_putative____Plasmodium_berghei
Plasmodium_falciparum3D7__5248
CRG94902_1_mitochondrial_pyruvate_carrier_protein_2_putative____Plasmodium_gallinaceum
Babesia_microtiRI__10360
Monocystis_agilis__27443
Eimeria_falciformisBayerHaberkorn1970__211651
Saprolegnia_parasitica__6847
XP_009833625_1_hypothetical_protein_H257_09188____Aphanomyces_astaci
Phytophthora_parasitica__8581
CEG44351_1_mitochondrial_pyruvate_carrier_2like____Plasmopara_halstedii
CCA17801_1_CSH_putative____Albugo_laibachii_Nc14
EWM25375_1_Uncharacterized_protein_family_UPF0041____Nannochloropsis_gaditana
Strombidium_inclinatum___StromIncl_2695
Strombidinopsis_acuminatum___StromAcum_124382
Stentor_coeruleus__27675
Protocruzia_adherens__25095
EJY85409_1_UPF0041_domain_containing_protein_macronuclear____Oxytricha_trifallax
Oxytricha_trifallax__15652
Halteria_grandinella___Halteria_81563
XP_001011175_2_hypothetical_protein_TTHERM_00145350____Tetrahymena_thermophila_SB210
Tetrahymena_thermophila__3150
Aristerostoma_sp___Aristero_50280
XP_001427854_1_hypothetical_protein_macronuclear____Paramecium_tetraurelia_strain_d42
Paramecium_tetraurelia___paratetr_55665
XP_001442209_1_hypothetical_protein_macronuclear____Paramecium_tetraurelia_strain_d42
Paramecium_tetraurelia___paratetr_77870
XP_004027185_1_hypothetical_protein_IMG5_187850____Ichthyophthirius_multifiliis
Furgasonia_blochmanni___FurgaBloc_42000
Platyophrya_macrostoma___Platyphrya_656
Euplotes_harpa___EuplHarp_24173
XP_016950115_1_PREDICTED_mitochondrial_pyruvate_carrier_3____Drosophila_biarmipes
Drosophila_melanogaster__6837
EZA61878_1_Brain_protein____Ooceraea_biroi
XP_014606365_1_PREDICTED_mitochondrial_pyruvate_carrier_2like____Polistes_canadensis
XP_001120371_1_mitochondrial_pyruvate_carrier_2like____Apis_mellifera
XP_014216515_1_mitochondrial_pyruvate_carrier_2like____Copidosoma_floridanum
KOB73589_1_Lightinduced_proteinlike_brain_protein_44____Operophtera_brumata
Aplysia_californica__14519
OQV16622_1_putative_Mitochondrial_pyruvate_carrier_2____Hypsibius_dujardini
Digyalum_oweni1__1494
Digyalum_oweni1__12084
RDD41855_1_Mitochondrial_pyruvate_carrier_2____Trichoplax_sp__H2
XP_002130972_1_PREDICTED_mitochondrial_pyruvate_carrier_2like____Ciona_intestinalis
Muranothrix_gubernatus___gnl_Murano_sp_10739_2
Aplysia_californica__10939
OBZ89047_1_Mitochondrial_pyruvate_carrier_2____Choanephora_cucurbitarum
Naegleria_gruberi__11093
PFX34575_1_Mitochondrial_pyruvate_carrier_2____Stylophora_pistillata
KXJ05710_1_Mitochondrial_pyruvate_carrier_2____Exaiptasia_pallida
XP_014772439_1_PREDICTED_mitochondrial_pyruvate_carrier_2like____Octopus_bimaculoides
PSN44619_1_Mitochondrial_pyruvate_carrier_2____Blattella_germanica
XP_002436279_1_brain_protein_putative_partial____Ixodes_scapularis
Branchiostoma_floridae__5560
XP_009062117_1_hypothetical_protein_LOTGIDRAFT_229316____Lottia_gigantea
CCK72428_1_hypothetical_protein_KNAG_0K00600____Kazachstania_naganishii_CBS_8797
SMN22375_1_similar_to_Saccharomyces_cerevisiae_YHR162W_MPC2_Putative_protein_of_unknown_function____Kazachstania_saulgeensis
Saccharomyces_cerevisiae__2451
Saccharomyces_cerevisiae__2202
NP_011759_1_mitochondrial_pyruvate_carrier____Saccharomyces_cerevisiae_S288C
AQZ17532_1_FMP43_YGR243W_and_MPC2_YHR162W____Zygosaccharomyces_parabailii
XP_013279340_1_hypothetical_protein_Z517_10274____Fonsecaea_pedrosoi_CBS_271_37
OCK84756_1_UPF0041domaincontaining_protein____Lepidopterella_palustris_CBS_459_81
CEJ59637_1_Putative_UPF0041_domain_protein____Penicillium_brasilianum
OQE17939_1_hypothetical_protein_PENSTE_c019G10207____Penicillium_steckii
XP_011125448_1_hypothetical_protein_AOL_s00112g116____Arthrobotrys_oligospora_ATCC_24927
EGZ77488_1_UPF0041domaincontaining_protein_partial____Neurospora_tetrasperma_FGSC_2509
XP_002479647_1_UPF0041_domain_protein____Talaromyces_stipitatus_ATCC_10500
Other_MPC2
Pygsuia_biforma__12890
Cyanidioschyzon_merolae__428
Coprinus_cinereus__6345
Ustilago_maydis__1112
KXS09368_1_UPF0041domaincontaining_protein____Gonapodya_prolifera_JEL478
XP_005709378_1_hypothetical_protein_Gasu_02090____Galdieria_sulphuraria
Galdieria_sulphuraria__6812
EFO26790_2_hypothetical_protein_LOAG_01692____Loa_loa
JAT90871_1_hypothetical_protein_g_12346____Pectinophora_gossypiella
CEO98271_1_hypothetical_protein_PBRA_006385____Plasmodiophora_brassicae
Plasmodiophora_brassicae__4671
Tiarina_fusus___tiarina_106642
Guillardia_theta__16755
EPZ31818_1_Mitochondrial_pyruvate_carrier_2____Rozella_allomycis_CSF55
ERZ95527_1_hypothetical_protein_GLOINDRAFT_341183____Rhizophagus_irregularis_DAOM_181602
KNE67874_1_hypothetical_protein_AMAG_12591____Allomyces_macrogynus_ATCC_38327
XP_003384059_1_PREDICTED_mitochondrial_pyruvate_carrier_2like____Amphimedon_queenslandica
sp_O95563_1_MPC2_HUMAN_RecName_Full_Mitochondrial_pyruvate_carrier_2_AltName_Full_Brain_protein_44
NP_001309871_1_mitochondrial_pyruvate_carrier_2_isoform_2____Rattus_norvegicus
XP_020371850_1_mitochondrial_pyruvate_carrier_2____Rhincodon_typus
XP_019519309_1_PREDICTED_LOW_QUALITY_PROTEIN_mitochondrial_pyruvate_carrier_2____Hipposideros_armiger
XP_024598171_1_mitochondrial_pyruvate_carrier_2_isoform_X1____Neophocaena_asiaeorientalis_asiaeorientalis
KPP64866_1_brain_protein_44like____Scleropages_formosus
XP_010776996_1_PREDICTED_mitochondrial_pyruvate_carrier_2____Notothenia_coriiceps
KTG33838_1_hypothetical_protein_cypCar_00016730____Cyprinus_carpio
XP_017573223_1_PREDICTED_mitochondrial_pyruvate_carrier_2like____Pygocentrus_nattereri
JAQ33795_1_Mitochondrial_pyruvate_carrier_2_partial____Fundulus_heteroclitus
AHH38071_1_Mitochondrial_pyruvate_carrier_2____Ictalurus_punctatus
JAP60236_1_Mitochondrial_pyruvate_carrier_2____Schistocephalus_solidus
XP_013414172_1_mitochondrial_pyruvate_carrier_4_isoform_X1____Lingula_anatina
XP_018026972_1_PREDICTED_mitochondrial_pyruvate_carrier_2like____Hyalella_azteca
XP_018324012_1_mitochondrial_pyruvate_carrier_2like____Agrilus_planipennis
0.5

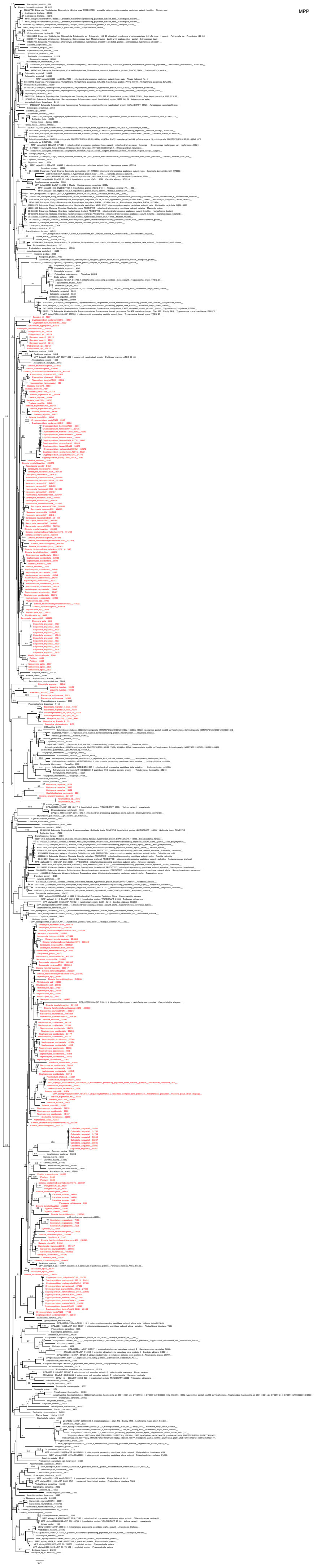
MPP

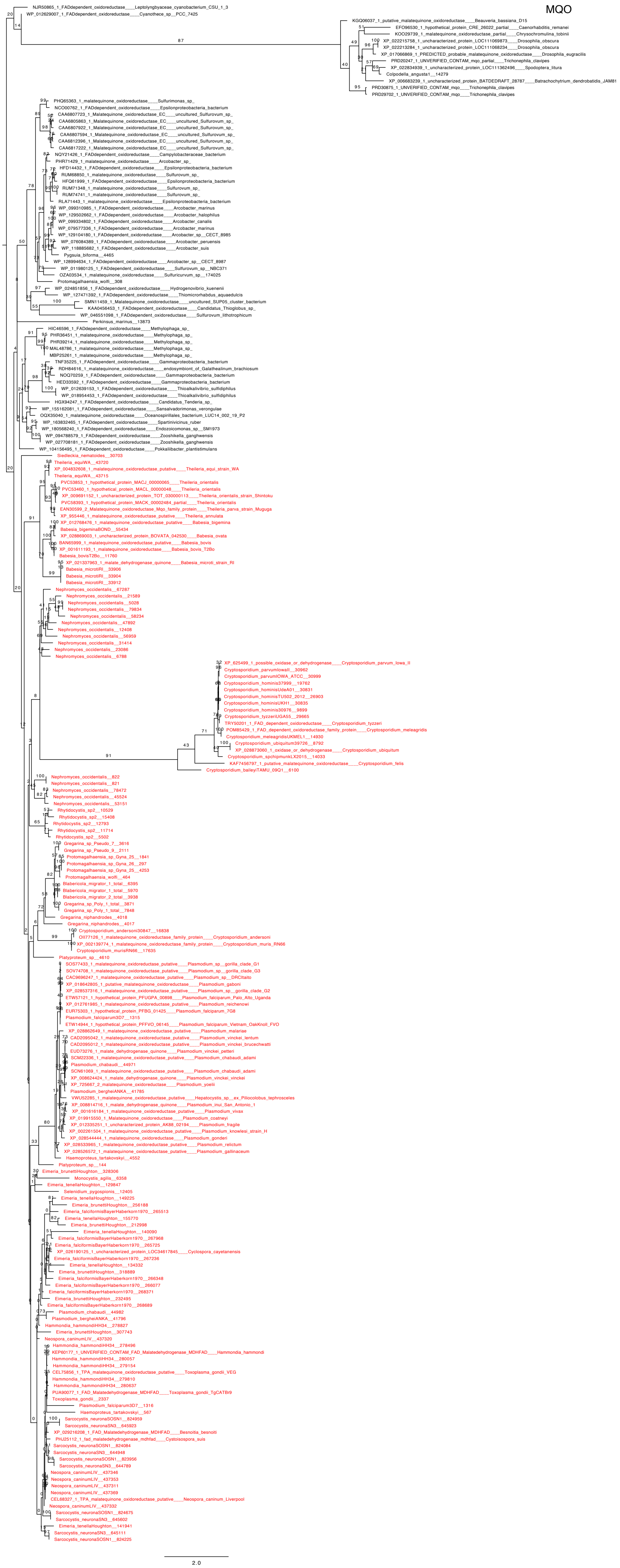
MQO
2.0

NDH2

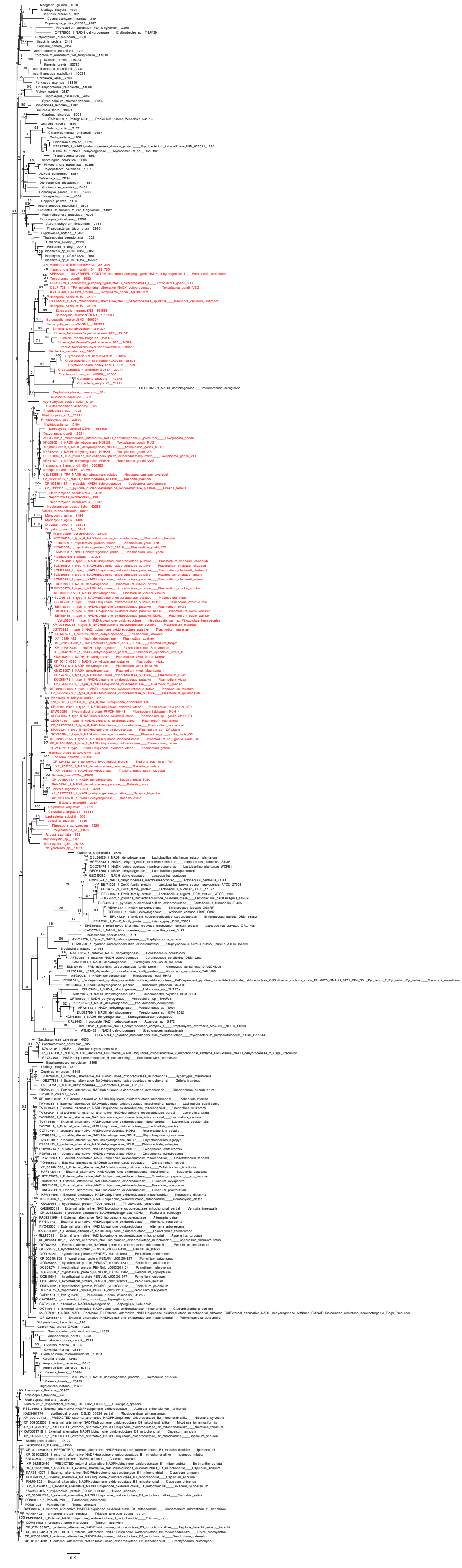

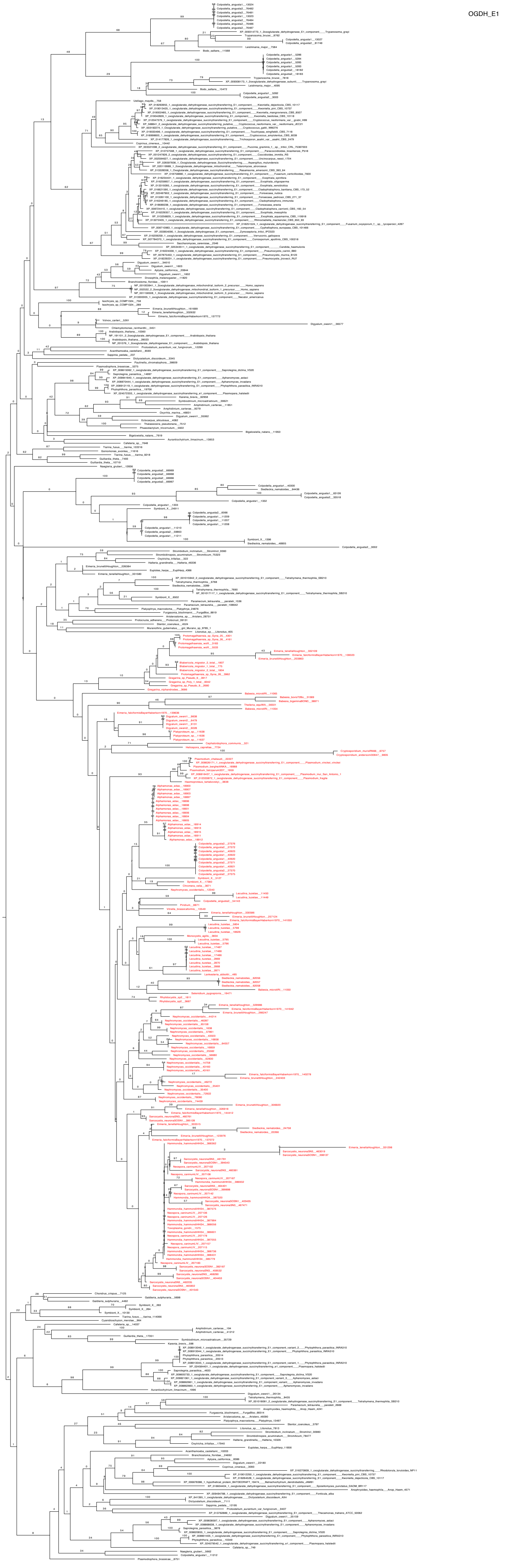
OGDH_E1
0.3

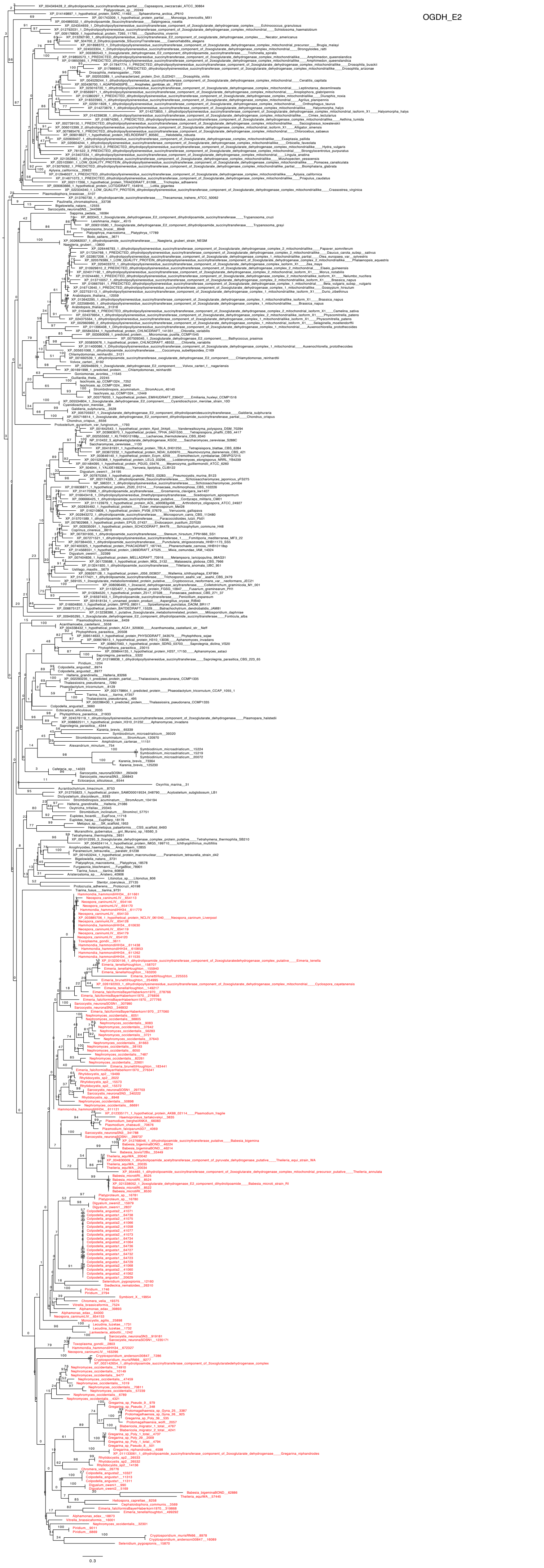
OGDH_E2
0.3

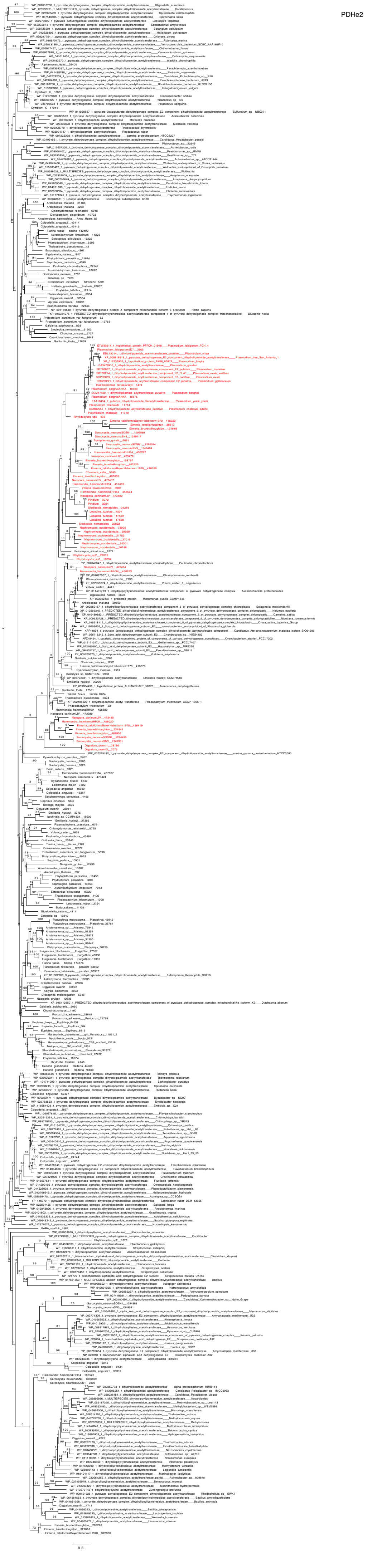
PDHe2
0.6

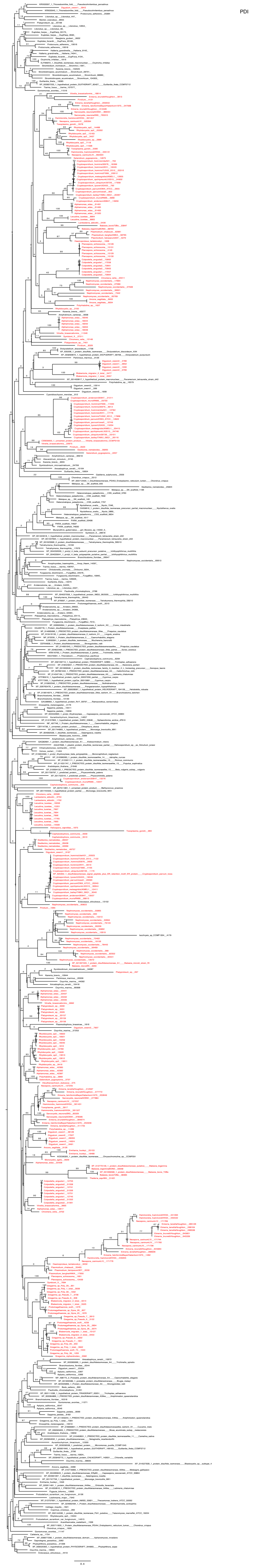
PDI
0.4

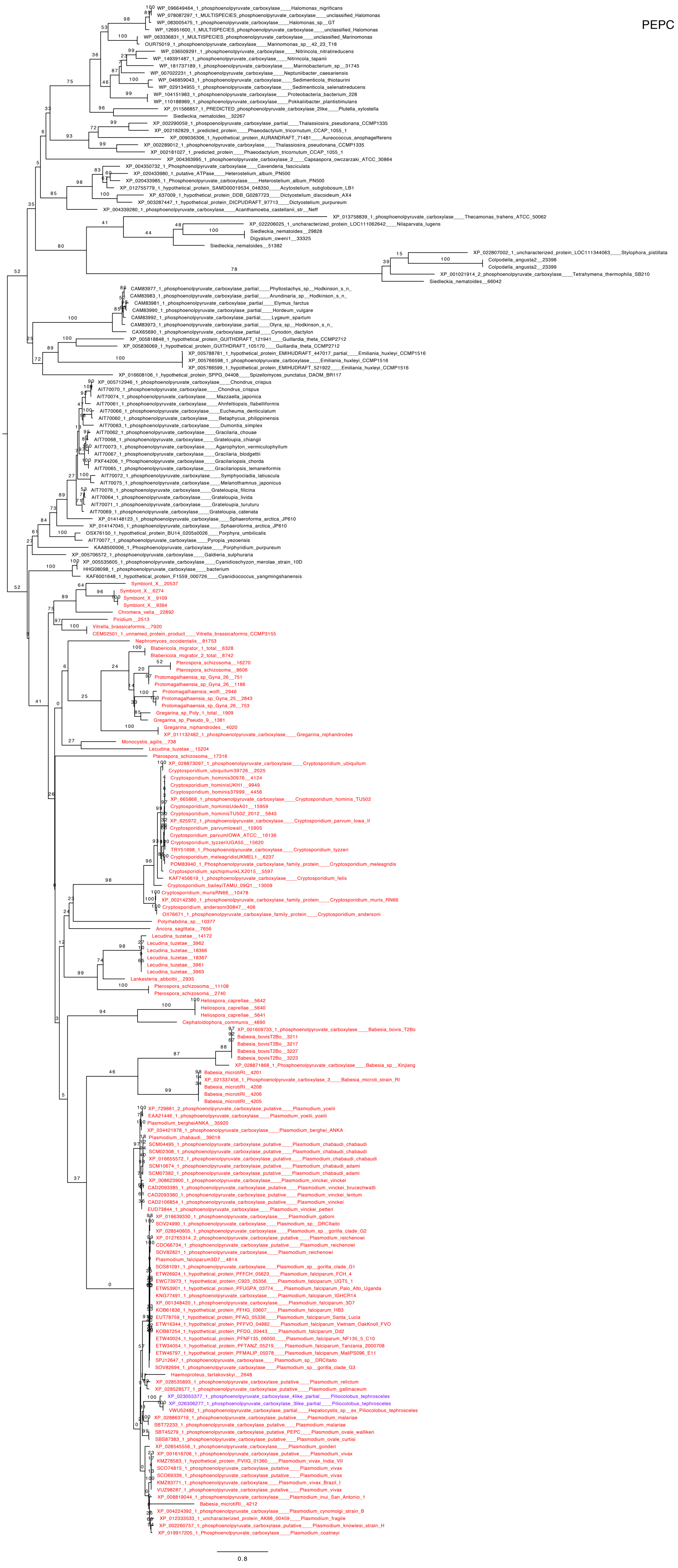
PEPC
0.8

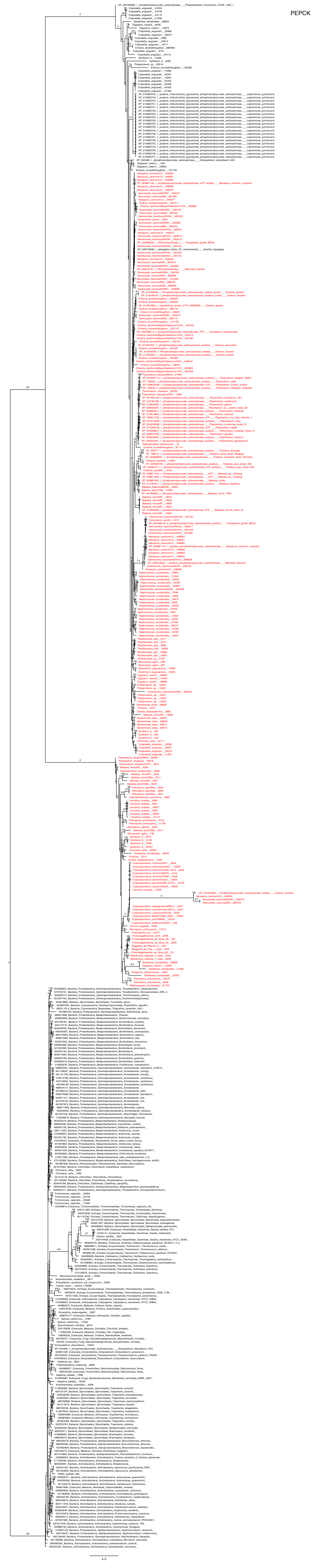
PEPCK
4.0

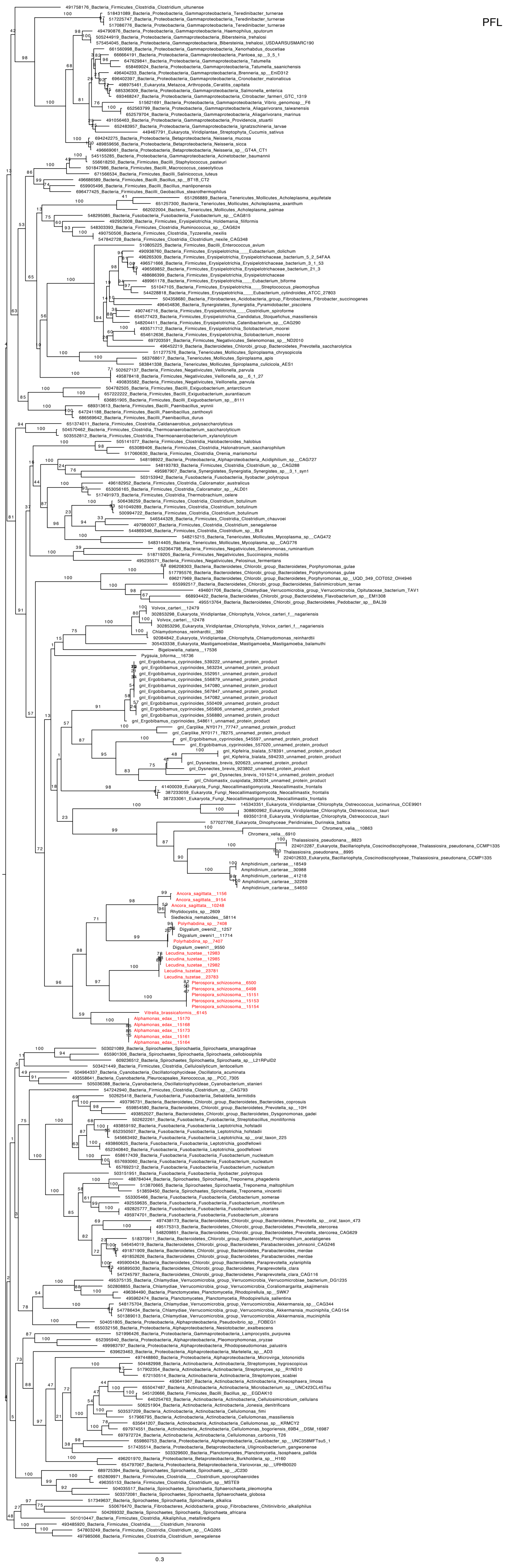
PFL
0.3

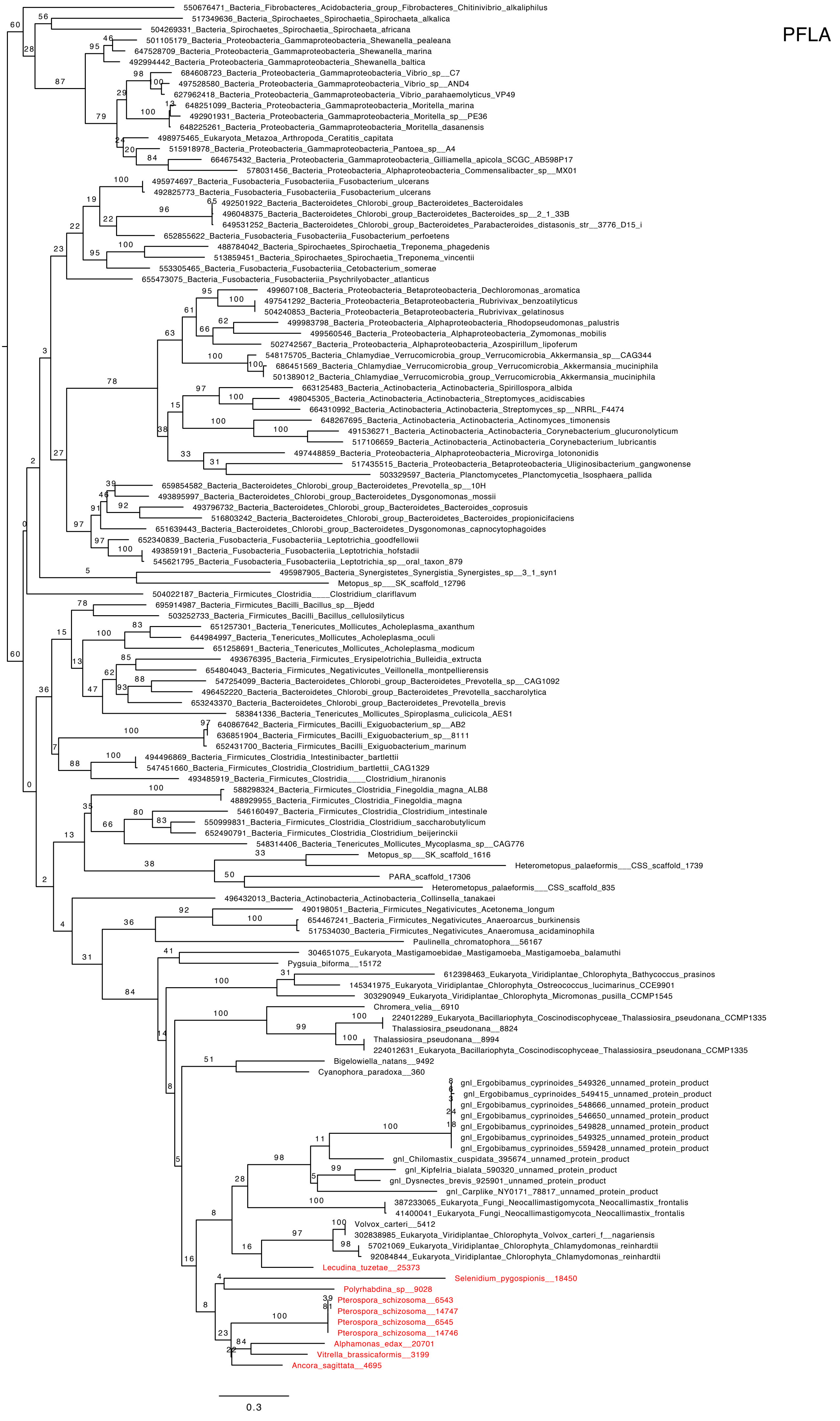
PFLA
550676471_Bacteria_Fibrobacteres_Acidobacteria_group_Fibrobacteres_Chitinivibrio_alkaliphilus
517349636_Bacteria_Spirochaetes_Spirochaetia_Spirochaeta_alkalica
504269331_Bacteria_Spirochaetes_Spirochaetia_Spirochaeta_africana
501105179_Bacteria_Proteobacteria_Gammaproteobacteria_Shewanella_pealeana
647528709_Bacteria_Proteobacteria_Gammaproteobacteria_Shewanella_marina
492994442_Bacteria_Proteobacteria_Gammaproteobacteria_Shewanella_baltica
684608723_Bacteria_Proteobacteria_Gammaproteobacteria_Vibrio_sp__C7
497528580_Bacteria_Proteobacteria_Gammaproteobacteria_Vibrio_sp__AND4
627962418_Bacteria_Proteobacteria_Gammaproteobacteria_Vibrio_parahaemolyticus_VP49
648251099_Bacteria_Proteobacteria_Gammaproteobacteria_Moritella_marina
492901931_Bacteria_Proteobacteria_Gammaproteobacteria_Moritella_sp__PE36
648225261_Bacteria_Proteobacteria_Gammaproteobacteria_Moritella_dasanensis
498975465_Eukaryota_Metazoa_Arthropoda_Ceratitis_capitata
515918978_Bacteria_Proteobacteria_Gammaproteobacteria_Pantoea_sp__A4
664675432_Bacteria_Proteobacteria_Gammaproteobacteria_Gilliamella_apicola_SCGC_AB598P17
578031456_Bacteria_Proteobacteria_Alphaproteobacteria_Commensalibacter_sp__MX01
495974697_Bacteria_Fusobacteria_Fusobacteriia_Fusobacterium_ulcerans
492825773_Bacteria_Fusobacteria_Fusobacteriia_Fusobacterium_ulcerans
492501922_Bacteria_Bacteroidetes_Chlorobi_group_Bacteroidetes_Bacteroidales
496048375_Bacteria_Bacteroidetes_Chlorobi_group_Bacteroidetes_Bacteroides_sp__2_1_33B
649531252_Bacteria_Bacteroidetes_Chlorobi_group_Bacteroidetes_Parabacteroides_distasonis_str__3776_D15_i
652855622_Bacteria_Fusobacteria_Fusobacteriia_Fusobacterium_perfoetens
488784042_Bacteria_Spirochaetes_Spirochaetia_Treponema_phagedenis
513859451_Bacteria_Spirochaetes_Spirochaetia_Treponema_vincentii
553305465_Bacteria_Fusobacteria_Fusobacteriia_Cetobacterium_somerae
655473075_Bacteria_Fusobacteria_Fusobacteriia_Psychrilyobacter_atlanticus
499607108_Bacteria_Proteobacteria_Betaproteobacteria_Dechloromonas_aromatica
497541292_Bacteria_Proteobacteria_Betaproteobacteria_Rubrivivax_benzoatilyticus
504240853_Bacteria_Proteobacteria_Betaproteobacteria_Rubrivivax_gelatinosus
499983798_Bacteria_Proteobacteria_Alphaproteobacteria_Rhodopseudomonas_palustris
499560546_Bacteria_Proteobacteria_Alphaproteobacteria_Zymomonas_mobilis
502742567_Bacteria_Proteobacteria_Alphaproteobacteria_Azospirillum_lipoferum
548175705_Bacteria_Chlamydiae_Verrucomicrobia_group_Verrucomicrobia_Akkermansia_sp__CAG344
686451569_Bacteria_Chlamydiae_Verrucomicrobia_group_Verrucomicrobia_Akkermansia_muciniphila
501389012_Bacteria_Chlamydiae_Verrucomicrobia_group_Verrucomicrobia_Akkermansia_muciniphila
663125483_Bacteria_Actinobacteria_Actinobacteria_Spirillospora_albida
498045305_Bacteria_Actinobacteria_Actinobacteria_Streptomyces_acidiscabies
664310992_Bacteria_Actinobacteria_Actinobacteria_Streptomyces_sp__NRRL_F4474
648267695_Bacteria_Actinobacteria_Actinobacteria_Actinomyces_timonensis
491536271_Bacteria_Actinobacteria_Actinobacteria_Corynebacterium_glucuronolyticum
517106659_Bacteria_Actinobacteria_Actinobacteria_Corynebacterium_lubricantis
497448859_Bacteria_Proteobacteria_Alphaproteobacteria_Microvirga_lotononidis
517435515_Bacteria_Proteobacteria_Betaproteobacteria_Uliginosibacterium_gangwonense
503329597_Bacteria_Planctomycetes_Planctomycetia_Isosphaera_pallida
659854582_Bacteria_Bacteroidetes_Chlorobi_group_Bacteroidetes_Prevotella_sp__10H
493895997_Bacteria_Bacteroidetes_Chlorobi_group_Bacteroidetes_Dysgonomonas_mossii
493796732_Bacteria_Bacteroidetes_Chlorobi_group_Bacteroidetes_Bacteroides_coprosuis
516803242_Bacteria_Bacteroidetes_Chlorobi_group_Bacteroidetes_Bacteroides_propionicifaciens
651639443_Bacteria_Bacteroidetes_Chlorobi_group_Bacteroidetes_Dysgonomonas_capnocytophagoides
652340839_Bacteria_Fusobacteria_Fusobacteriia_Leptotrichia_goodfellowii
493859191_Bacteria_Fusobacteria_Fusobacteriia_Leptotrichia_hofstadii
545621795_Bacteria_Fusobacteria_Fusobacteriia_Leptotrichia_sp__oral_taxon_879
495987905_Bacteria_Synergistetes_Synergistia_Synergistes_sp__3_1_syn1
Metopus_sp___SK_scaffold_12796
504022187_Bacteria_Firmicutes_Clostridia____Clostridium_clariflavum
695914987_Bacteria_Firmicutes_Bacilli_Bacillus_sp__Bjedd
503252733_Bacteria_Firmicutes_Bacilli_Bacillus_cellulosilyticus
651257301_Bacteria_Tenericutes_Mollicutes_Acholeplasma_axanthum
644984997_Bacteria_Tenericutes_Mollicutes_Acholeplasma_oculi
651258691_Bacteria_Tenericutes_Mollicutes_Acholeplasma_modicum
493676395_Bacteria_Firmicutes_Erysipelotrichia_Bulleidia_extructa
654804043_Bacteria_Firmicutes_Negativicutes_Veillonella_montpellierensis
547254099_Bacteria_Bacteroidetes_Chlorobi_group_Bacteroidetes_Prevotella_sp__CAG1092
496452220_Bacteria_Bacteroidetes_Chlorobi_group_Bacteroidetes_Prevotella_saccharolytica
653243370_Bacteria_Bacteroidetes_Chlorobi_group_Bacteroidetes_Prevotella_brevis
583841336_Bacteria_Tenericutes_Mollicutes_Spiroplasma_culicicola_AES1
640867642_Bacteria_Firmicutes_Bacilli_Exiguobacterium_sp__AB2
636851904_Bacteria_Firmicutes_Bacilli_Exiguobacterium_sp__8111
652431700_Bacteria_Firmicutes_Bacilli_Exiguobacterium_marinum
494496869_Bacteria_Firmicutes_Clostridia_Intestinibacter_bartlettii
547451660_Bacteria_Firmicutes_Clostridia_Clostridium_bartlettii_CAG1329
493485919_Bacteria_Firmicutes_Clostridia____Clostridium_hiranonis
588298324_Bacteria_Firmicutes_Clostridia_Finegoldia_magna_ALB8
488929955_Bacteria_Firmicutes_Clostridia_Finegoldia_magna
546160497_Bacteria_Firmicutes_Clostridia_Clostridium_intestinale
550999831_Bacteria_Firmicutes_Clostridia_Clostridium_saccharobutylicum
652490791_Bacteria_Firmicutes_Clostridia_Clostridium_beijerinckii
548314406_Bacteria_Tenericutes_Mollicutes_Mycoplasma_sp__CAG776
Metopus_sp___SK_scaffold_1616
Heterometopus_palaeformis___CSS_scaffold_1739
PARA_scaffold_17306
Heterometopus_palaeformis___CSS_scaffold_835
496432013_Bacteria_Actinobacteria_Actinobacteria_Collinsella_tanakaei
490198051_Bacteria_Firmicutes_Negativicutes_Acetonema_longum
654467241_Bacteria_Firmicutes_Negativicutes_Anaeroarcus_burkinensis
517534030_Bacteria_Firmicutes_Negativicutes_Anaeromusa_acidaminophila
Paulinella_chromatophora__56167
304651075_Eukaryota_Mastigamoebidae_Mastigamoeba_Mastigamoeba_balamuthi
Pygsuia_biforma__15172
612398463_Eukaryota_Viridiplantae_Chlorophyta_Bathycoccus_prasinos
145341975_Eukaryota_Viridiplantae_Chlorophyta_Ostreococcus_lucimarinus_CCE9901
303290949_Eukaryota_Viridiplantae_Chlorophyta_Micromonas_pusilla_CCMP1545
Chromera_velia__6910
224012289_Eukaryota_Bacillariophyta_Coscinodiscophyceae_Thalassiosira_pseudonana_CCMP1335
Thalassiosira_pseudonana__8824
Thalassiosira_pseudonana__8994
224012631_Eukaryota_Bacillariophyta_Coscinodiscophyceae_Thalassiosira_pseudonana_CCMP1335
Bigelowiella_natans__9492
Cyanophora_paradoxa__360
gnl_Ergobibamus_cyprinoides_549326_unnamed_protein_product
gnl_Ergobibamus_cyprinoides_549415_unnamed_protein_product
gnl_Ergobibamus_cyprinoides_548666_unnamed_protein_product
gnl_Ergobibamus_cyprinoides_546650_unnamed_protein_product
gnl_Ergobibamus_cyprinoides_549828_unnamed_protein_product
gnl_Ergobibamus_cyprinoides_549325_unnamed_protein_product
gnl_Ergobibamus_cyprinoides_559428_unnamed_protein_product
gnl_Chilomastix_cuspidata_395674_unnamed_protein_product
gnl_Kipfelria_bialata_590320_unnamed_protein_product
gnl_Dysnectes_brevis_925901_unnamed_protein_product
gnl_Carplike_NY0171_78817_unnamed_protein_product
387233065_Eukaryota_Fungi_Neocallimastigomycota_Neocallimastix_frontalis
41400041_Eukaryota_Fungi_Neocallimastigomycota_Neocallimastix_frontalis
Volvox_carteri__5412
302838985_Eukaryota_Viridiplantae_Chlorophyta_Volvox_carteri_f__nagariensis
57021069_Eukaryota_Viridiplantae_Chlorophyta_Chlamydomonas_reinhardtii
92084844_Eukaryota_Viridiplantae_Chlorophyta_Chlamydomonas_reinhardtii
Lecudina_tuzetae__25373
Selenidium_pygospionis__18450
Polyrhabdina_sp__9028
Pterospora_schizosoma__6543
Pterospora_schizosoma__14747
Pterospora_schizosoma__6545
Pterospora_schizosoma__14746
Alphamonas_edax__20701
Vitrella_brassicaformis__3199
Ancora_sagittata__4695
0.3

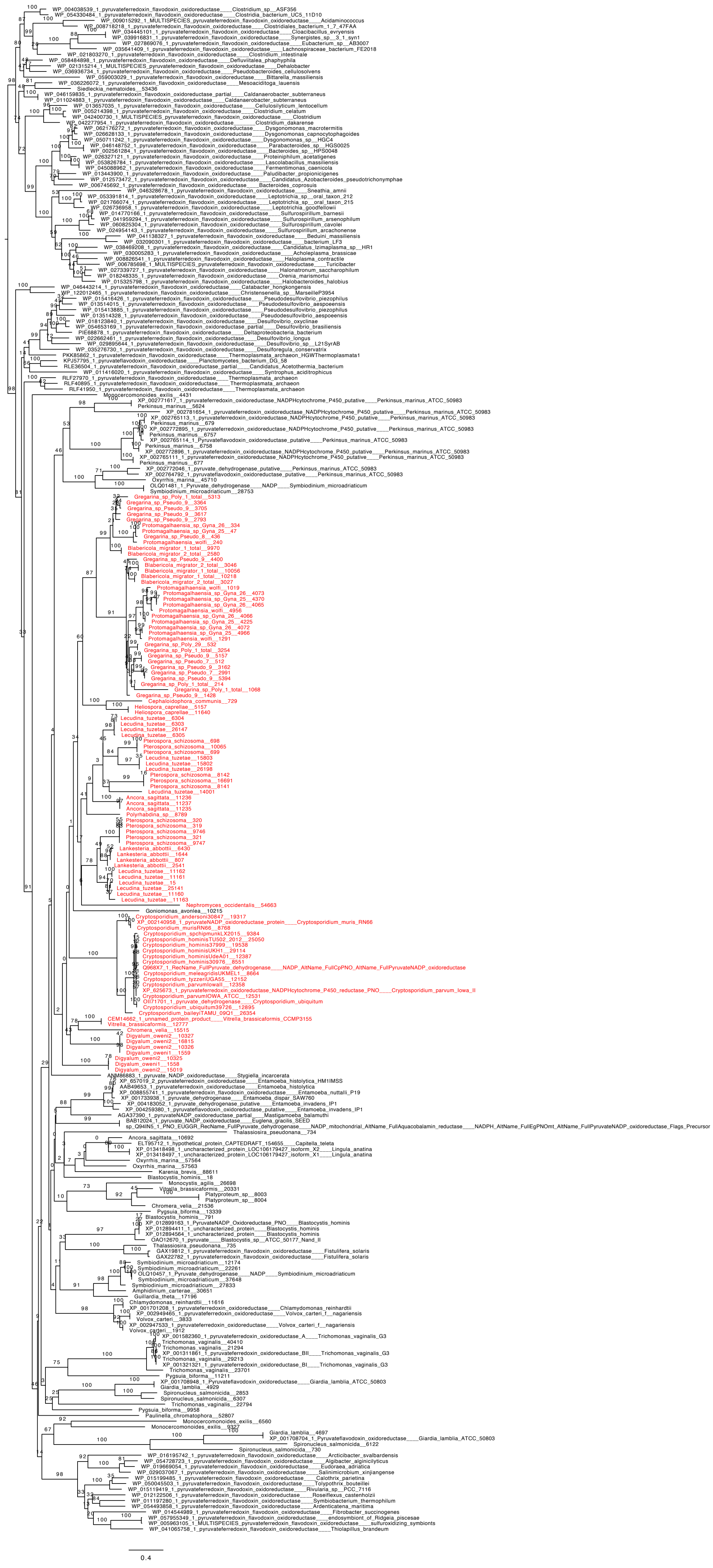
PNO
0.4

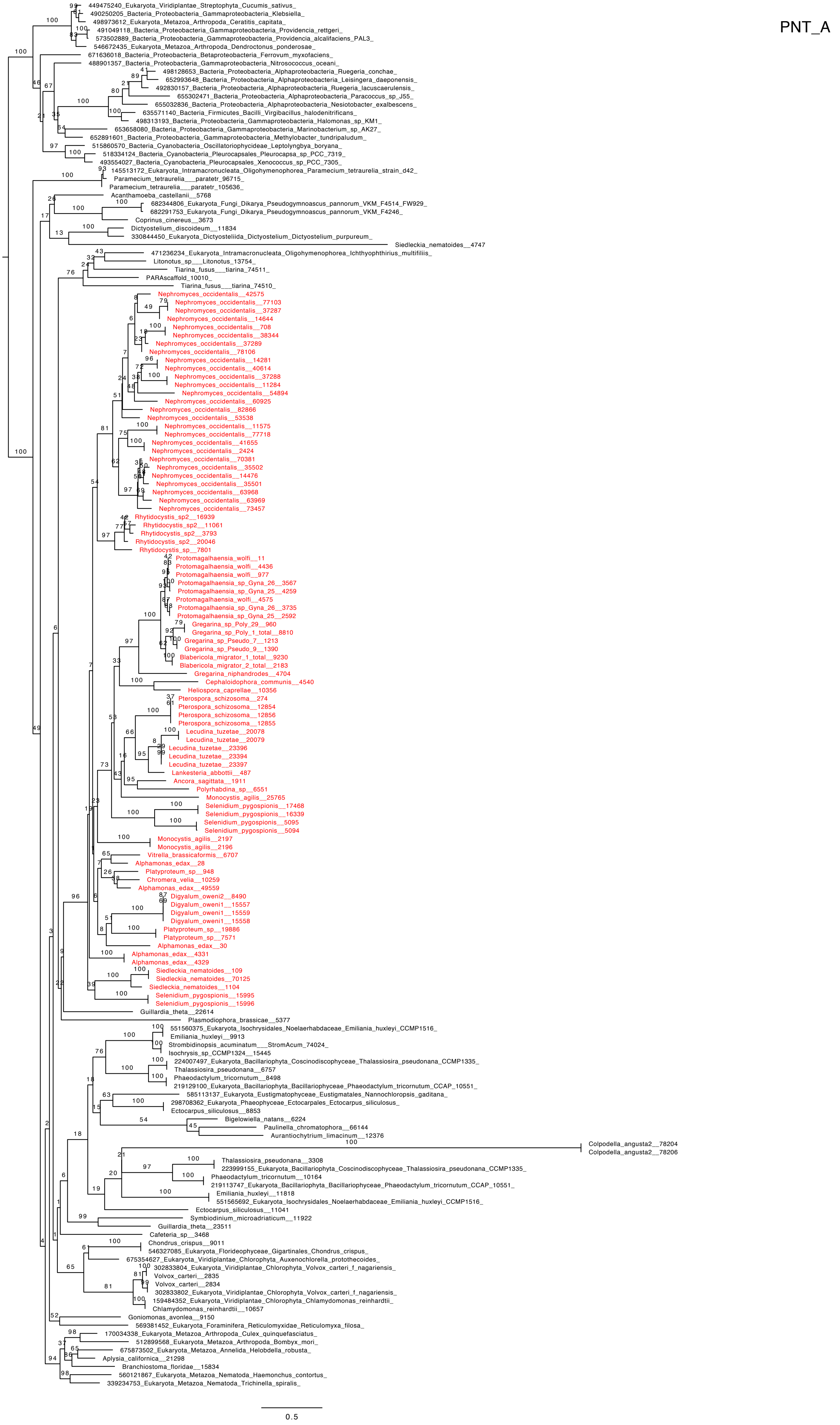
PNT_A
449475240_Eukaryota_Viridiplantae_Streptophyta_Cucumis_sativus_
490250205_Bacteria_Proteobacteria_Gammaproteobacteria_Klebsiella_
498973612_Eukaryota_Metazoa_Arthropoda_Ceratitis_capitata_
491049118_Bacteria_Proteobacteria_Gammaproteobacteria_Providencia_rettgeri_
573502889_Bacteria_Proteobacteria_Gammaproteobacteria_Providencia_alcalifaciens_PAL3_
546672435_Eukaryota_Metazoa_Arthropoda_Dendroctonus_ponderosae_
671636018_Bacteria_Proteobacteria_Betaproteobacteria_Ferrovum_myxofaciens_
488901357_Bacteria_Proteobacteria_Gammaproteobacteria_Nitrosococcus_oceani_
498128653_Bacteria_Proteobacteria_Alphaproteobacteria_Ruegeria_conchae_
652993648_Bacteria_Proteobacteria_Alphaproteobacteria_Leisingera_daeponensis_
492830157_Bacteria_Proteobacteria_Alphaproteobacteria_Ruegeria_lacuscaerulensis_
655302471_Bacteria_Proteobacteria_Alphaproteobacteria_Paracoccus_sp_J55_
655032836_Bacteria_Proteobacteria_Alphaproteobacteria_Nesiotobacter_exalbescens_
635571140_Bacteria_Firmicutes_Bacilli_Virgibacillus_halodenitrificans_
498313193_Bacteria_Proteobacteria_Gammaproteobacteria_Halomonas_sp_KM1_
653658080_Bacteria_Proteobacteria_Gammaproteobacteria_Marinobacterium_sp_AK27_
652891601_Bacteria_Proteobacteria_Gammaproteobacteria_Methylobacter_tundripaludum_
515860570_Bacteria_Cyanobacteria_Oscillatoriophycideae_Leptolyngbya_boryana_
518334124_Bacteria_Cyanobacteria_Pleurocapsales_Pleurocapsa_sp_PCC_7319_
493554027_Bacteria_Cyanobacteria_Pleurocapsales_Xenococcus_sp_PCC_7305_
145513172_Eukaryota_Intramacronucleata_Oligohymenophorea_Paramecium_tetraurelia_strain_d42_
Paramecium_tetraurelia___paratetr_96715_
Paramecium_tetraurelia___paratetr_105636_
Acanthamoeba_castellanii__5768
682344806_Eukaryota_Fungi_Dikarya_Pseudogymnoascus_pannorum_VKM_F4514_FW929_
682291753_Eukaryota_Fungi_Dikarya_Pseudogymnoascus_pannorum_VKM_F4246_
Coprinus_cinereus__3673
Dictyostelium_discoideum__11834
330844450_Eukaryota_Dictyosteliida_Dictyostelium_Dictyostelium_purpureum_
Siedleckia_nematoides__4747
471236234_Eukaryota_Intramacronucleata_Oligohymenophorea_Ichthyophthirius_multifiliis_
Litonotus_sp___Litonotus_13754_
Tiarina_fusus___tiarina_74511_
PARAscaffold_10010_
Tiarina_fusus___tiarina_74510_
Nephromyces_occidentalis__42575
Nephromyces_occidentalis__77103
Nephromyces_occidentalis__37287
Nephromyces_occidentalis__14644
Nephromyces_occidentalis__708
Nephromyces_occidentalis__38344
Nephromyces_occidentalis__37289
Nephromyces_occidentalis__78106
Nephromyces_occidentalis__14281
Nephromyces_occidentalis__40614
Nephromyces_occidentalis__37288
Nephromyces_occidentalis__11284
Nephromyces_occidentalis__54894
Nephromyces_occidentalis__60925
Nephromyces_occidentalis__82866
Nephromyces_occidentalis__53538
Nephromyces_occidentalis__11575
Nephromyces_occidentalis__77718
Nephromyces_occidentalis__41655
Nephromyces_occidentalis__2424
Nephromyces_occidentalis__70381
Nephromyces_occidentalis__35502
Nephromyces_occidentalis__14476
Nephromyces_occidentalis__35501
Nephromyces_occidentalis__63968
Nephromyces_occidentalis__63969
Nephromyces_occidentalis__73457
Rhytidocystis_sp2__16939
Rhytidocystis_sp2__11061
Rhytidocystis_sp2__3793
Rhytidocystis_sp2__20046
Rhytidocystis_sp__7801
Protomagalhaensia_wolfi__11
Protomagalhaensia_wolfi__4436
Protomagalhaensia_wolfi__977
Protomagalhaensia_sp_Gyna_26__3567
Protomagalhaensia_sp_Gyna_25__4259
Protomagalhaensia_wolfi__4575
Protomagalhaensia_sp_Gyna_26__3735
Protomagalhaensia_sp_Gyna_25__2592
Gregarina_sp_Poly_29__960
Gregarina_sp_Poly_1_total__8810
Gregarina_sp_Pseudo_7__1213
Gregarina_sp_Pseudo_9__1390
Blabericola_migrator_1_total__9230
Blabericola_migrator_2_total__2183
Gregarina_niphandrodes__4704
Cephaloidophora_communis__4540
Heliospora_caprellae__10356
Pterospora_schizosoma__274
Pterospora_schizosoma__12854
Pterospora_schizosoma__12856
Pterospora_schizosoma__12855
Lecudina_tuzetae__20078
Lecudina_tuzetae__20079
Lecudina_tuzetae__23396
Lecudina_tuzetae__23394
Lecudina_tuzetae__23397
Lankesteria_abbottii__487
Ancora_sagittata__1911
Polyrhabdina_sp__6551
Monocystis_agilis__25765
Selenidium_pygospionis__17468
Selenidium_pygospionis__16339
Selenidium_pygospionis__5095
Selenidium_pygospionis__5094
Monocystis_agilis__2197
Monocystis_agilis__2196
Vitrella_brassicaformis__6707
Alphamonas_edax__28
Platyproteum_sp__948
Chromera_velia__10259
Alphamonas_edax__49559
Digyalum_oweni2__8490
Digyalum_oweni1__15557
Digyalum_oweni1__15559
Digyalum_oweni1__15558
Platyproteum_sp__19886
Platyproteum_sp__7571
Alphamonas_edax__30
Alphamonas_edax__4331
Alphamonas_edax__4329
Siedleckia_nematoides__109
Siedleckia_nematoides__70125
Siedleckia_nematoides__1104
Selenidium_pygospionis__15995
Selenidium_pygospionis__15996
Guillardia_theta__22614
Plasmodiophora_brassicae__5377
551560375_Eukaryota_Isochrysidales_Noelaerhabdaceae_Emiliania_huxleyi_CCMP1516_
Emiliania_huxleyi__9913
Strombidinopsis_acuminatum___StromAcum_74024_
Isochrysis_sp_CCMP1324__15445
224007497_Eukaryota_Bacillariophyta_Coscinodiscophyceae_Thalassiosira_pseudonana_CCMP1335_
Thalassiosira_pseudonana__6757
Phaeodactylum_tricornutum__8498
219129100_Eukaryota_Bacillariophyta_Bacillariophyceae_Phaeodactylum_tricornutum_CCAP_10551_
585113137_Eukaryota_Eustigmatophyceae_Eustigmatales_Nannochloropsis_gaditana_
298708362_Eukaryota_Phaeophyceae_Ectocarpales_Ectocarpus_siliculosus_
Ectocarpus_siliculosus__8853
Bigelowiella_natans__6224
Paulinella_chromatophora__66144
Aurantiochytrium_limacinum__12376
Colpodella_angusta2__78204
Colpodella_angusta2__78206
Thalassiosira_pseudonana__3308
223999155_Eukaryota_Bacillariophyta_Coscinodiscophyceae_Thalassiosira_pseudonana_CCMP1335_
Phaeodactylum_tricornutum__10164
219113747_Eukaryota_Bacillariophyta_Bacillariophyceae_Phaeodactylum_tricornutum_CCAP_10551_
Emiliania_huxleyi__11818
551565692_Eukaryota_Isochrysidales_Noelaerhabdaceae_Emiliania_huxleyi_CCMP1516_
Ectocarpus_siliculosus__11041
Symbiodinium_microadriaticum__11922
Guillardia_theta__23511
Cafeteria_sp__3468
Chondrus_crispus__9011
546327085_Eukaryota_Florideophyceae_Gigartinales_Chondrus_crispus_
675354627_Eukaryota_Viridiplantae_Chlorophyta_Auxenochlorella_protothecoides_
302833804_Eukaryota_Viridiplantae_Chlorophyta_Volvox_carteri_f_nagariensis_
Volvox_carteri__2835
Volvox_carteri__2834
302833802_Eukaryota_Viridiplantae_Chlorophyta_Volvox_carteri_f_nagariensis_
159484352_Eukaryota_Viridiplantae_Chlorophyta_Chlamydomonas_reinhardtii_
Chlamydomonas_reinhardtii__10657
Goniomonas_avonlea__9150
569381452_Eukaryota_Foraminifera_Reticulomyxidae_Reticulomyxa_filosa_
170034338_Eukaryota_Metazoa_Arthropoda_Culex_quinquefasciatus_
512899568_Eukaryota_Metazoa_Arthropoda_Bombyx_mori_
675873502_Eukaryota_Metazoa_Annelida_Helobdella_robusta_
Aplysia_californica__21298
Branchiostoma_floridae__15834
560121867_Eukaryota_Metazoa_Nematoda_Haemonchus_contortus_
339234753_Eukaryota_Metazoa_Nematoda_Trichinella_spiralis_
0.5

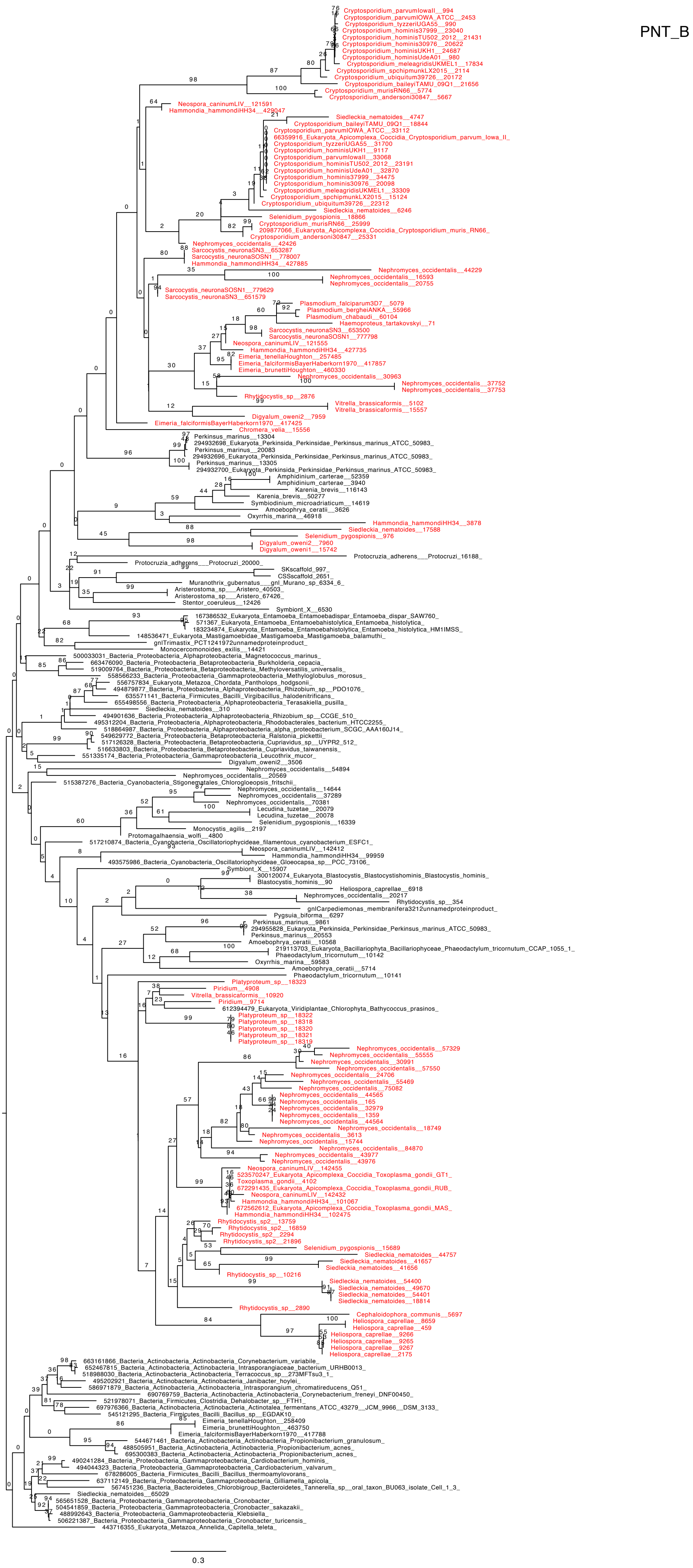
PNT_B
Cryptosporidium_parvumIowaII__994
Cryptosporidium_parvumIOWA_ATCC__2453
Cryptosporidium_tyzzeriUGA55__990
Cryptosporidium_hominis37999__23040
Cryptosporidium_hominisTU502_2012__21431
Cryptosporidium_hominis30976__20622
Cryptosporidium_hominisUKH1__24687
Cryptosporidium_hominisUdeA01__980
Cryptosporidium_meleagridisUKMEL1__17834
Cryptosporidium_spchipmunkLX2015__2114
Cryptosporidium_ubiquitum39726__20172
Cryptosporidium_baileyiTAMU_09Q1__21656
Cryptosporidium_murisRN66__5774
Cryptosporidium_andersoni30847__5667
Neospora_caninumLIV__121591
Hammondia_hammondiHH34__429047
Siedleckia_nematoides__4747
Cryptosporidium_baileyiTAMU_09Q1__18844
Cryptosporidium_parvumIOWA_ATCC__33112
66359916_Eukaryota_Apicomplexa_Coccidia_Cryptosporidium_parvum_Iowa_II_
Cryptosporidium_tyzzeriUGA55__31700
Cryptosporidium_hominisUKH1__9117
Cryptosporidium_parvumIowaII__33068
Cryptosporidium_hominisTU502_2012__23191
Cryptosporidium_hominisUdeA01__32870
Cryptosporidium_hominis37999__34475
Cryptosporidium_hominis30976__20098
Cryptosporidium_meleagridisUKMEL1__33309
Cryptosporidium_spchipmunkLX2015__15124
Cryptosporidium_ubiquitum39726__22312
Siedleckia_nematoides__6246
Selenidium_pygospionis__18866
Cryptosporidium_murisRN66__25999
209877066_Eukaryota_Apicomplexa_Coccidia_Cryptosporidium_muris_RN66_
Cryptosporidium_andersoni30847__25331
Nephromyces_occidentalis__42426
Sarcocystis_neuronaSN3__653287
Sarcocystis_neuronaSOSN1__778007
Hammondia_hammondiHH34__427885
Nephromyces_occidentalis__44229
Nephromyces_occidentalis__16593
Nephromyces_occidentalis__20755
Sarcocystis_neuronaSOSN1__779629
Sarcocystis_neuronaSN3__651579
Plasmodium_falciparum3D7__5079
Plasmodium_bergheiANKA__55966
Plasmodium_chabaudi__60104
Haemoproteus_tartakovskyi__71
Sarcocystis_neuronaSN3__653500
Sarcocystis_neuronaSOSN1__777798
Neospora_caninumLIV__121555
Hammondia_hammondiHH34__427735
Eimeria_tenellaHoughton__257485
Eimeria_falciformisBayerHaberkorn1970__417857
Eimeria_brunettiHoughton__460330
Nephromyces_occidentalis__30963
Nephromyces_occidentalis__37752
Nephromyces_occidentalis__37753
Rhytidocystis_sp__2876
Vitrella_brassicaformis__5102
Vitrella_brassicaformis__15557
Digyalum_oweni2__7959
Eimeria_falciformisBayerHaberkorn1970__417425
Chromera_velia__15556
Perkinsus_marinus__13304
294932698_Eukaryota_Perkinsida_Perkinsidae_Perkinsus_marinus_ATCC_50983_
Perkinsus_marinus__20083
294932696_Eukaryota_Perkinsida_Perkinsidae_Perkinsus_marinus_ATCC_50983_
Perkinsus_marinus__13305
294932700_Eukaryota_Perkinsida_Perkinsidae_Perkinsus_marinus_ATCC_50983_
Amphidinium_carterae__52359
Amphidinium_carterae__3940
Karenia_brevis__116143
Karenia_brevis__50277
Symbiodinium_microadriaticum__14619
Amoebophrya_ceratii__3626
Oxyrrhis_marina__46918
Hammondia_hammondiHH34__3878
Siedleckia_nematoides__17588
Selenidium_pygospionis__976
Digyalum_oweni2__7960
Digyalum_oweni1__15742
Protocruzia_adherens__Protocruzi_16188_
Protocruzia_adherens__Protocruzi_20000_
SKscaffold_997_
CSSscaffold_2651_
Muranothrix_gubernatus__gnl_Murano_sp_6334_6_
Aristerostoma_sp__Aristero_40503_
Aristerostoma_sp__Aristero_67426_
Stentor_coeruleus__12426
Symbiont_X__6530
167386532_Eukaryota_Entamoeba_Entamoebadispar_Entamoeba_dispar_SAW760_
571367_Eukaryota_Entamoeba_Entamoebahistolytica_Entamoeba_histolytica_
183234874_Eukaryota_Entamoeba_Entamoebahistolytica_Entamoeba_histolytica_HM1IMSS_
148536471_Eukaryota_Mastigamoebidae_Mastigamoeba_Mastigamoeba_balamuthi_
gnlTrimastix_PCT1241972unnamedproteinproduct_
Monocercomonoides_exilis__14421
500033031_Bacteria_Proteobacteria_Alphaproteobacteria_Magnetococcus_marinus_
663476090_Bacteria_Proteobacteria_Betaproteobacteria_Burkholderia_cepacia_
519009764_Bacteria_Proteobacteria_Betaproteobacteria_Methyloversatilis_universalis_
558566233_Bacteria_Proteobacteria_Gammaproteobacteria_Methyloglobulus_morosus_
556757834_Eukaryota_Metazoa_Chordata_Pantholops_hodgsonii_
494879877_Bacteria_Proteobacteria_Alphaproteobacteria_Rhizobium_sp__PDO1076_
635571141_Bacteria_Firmicutes_Bacilli_Virgibacillus_halodenitrificans_
655498556_Bacteria_Proteobacteria_Alphaproteobacteria_Terasakiella_pusilla_
Siedleckia_nematoides__310
494901636_Bacteria_Proteobacteria_Alphaproteobacteria_Rhizobium_sp__CCGE_510_
495312204_Bacteria_Proteobacteria_Alphaproteobacteria_Rhodobacterales_bacterium_HTCC2255_
518864987_Bacteria_Proteobacteria_Alphaproteobacteria_alpha_proteobacterium_SCGC_AAA160J14_
549629772_Bacteria_Proteobacteria_Betaproteobacteria_Ralstonia_pickettii_
517126328_Bacteria_Proteobacteria_Betaproteobacteria_Cupriavidus_sp__UYPR2_512_
516633803_Bacteria_Proteobacteria_Betaproteobacteria_Cupriavidus_taiwanensis_
551335174_Bacteria_Proteobacteria_Gammaproteobacteria_Leucothrix_mucor_
Digyalum_oweni2__3506
Nephromyces_occidentalis__54894
Nephromyces_occidentalis__20569
515387276_Bacteria_Cyanobacteria_Stigonematales_Chlorogloeopsis_fritschii_
Nephromyces_occidentalis__14644
Nephromyces_occidentalis__37289
Nephromyces_occidentalis__70381
Lecudina_tuzetae__20079
Lecudina_tuzetae__20078
Selenidium_pygospionis__16339
Monocystis_agilis__2197
Protomagalhaensia_wolfi__4800
517210874_Bacteria_Cyanobacteria_Oscillatoriophycideae_filamentous_cyanobacterium_ESFC1_
Neospora_caninumLIV__142412
Hammondia_hammondiHH34__99959
493575986_Bacteria_Cyanobacteria_Oscillatoriophycideae_Gloeocapsa_sp__PCC_73106_
Symbiont_X__15907
300120074_Eukaryota_Blastocystis_Blastocystishominis_Blastocystis_hominis_
Blastocystis_hominis__90
Heliospora_caprellae__6918
Nephromyces_occidentalis__20217
Rhytidocystis_sp__354
gnlCarpediemonas_membranifera3212unnamedproteinproduct_
Pygsuia_biforma__6297
Perkinsus_marinus__9861
294955828_Eukaryota_Perkinsida_Perkinsidae_Perkinsus_marinus_ATCC_50983_
Perkinsus_marinus__20553
Amoebophrya_ceratii__10568
219113703_Eukaryota_Bacillariophyta_Bacillariophyceae_Phaeodactylum_tricornutum_CCAP_1055_1_
Phaeodactylum_tricornutum__10142
Oxyrrhis_marina__59583
Amoebophrya_ceratii__5714
Phaeodactylum_tricornutum__10141
Platyproteum_sp__18323
Piridium__4908
Vitrella_brassicaformis__10920
Piridium__9714
612394479_Eukaryota_Viridiplantae_Chlorophyta_Bathycoccus_prasinos_
Platyproteum_sp__18322
Platyproteum_sp__18318
Platyproteum_sp__18320
Platyproteum_sp__18321
Platyproteum_sp__18319
Nephromyces_occidentalis__57329
Nephromyces_occidentalis__55555
Nephromyces_occidentalis__30991
Nephromyces_occidentalis__57550
Nephromyces_occidentalis__24706
Nephromyces_occidentalis__55469
Nephromyces_occidentalis__75082
Nephromyces_occidentalis__44565
Nephromyces_occidentalis__165
Nephromyces_occidentalis__32979
Nephromyces_occidentalis__1359
Nephromyces_occidentalis__44564
Nephromyces_occidentalis__18749
Nephromyces_occidentalis__3613
Nephromyces_occidentalis__15744
Nephromyces_occidentalis__84870
Nephromyces_occidentalis__43977
Nephromyces_occidentalis__43976
Neospora_caninumLIV__142455
523570247_Eukaryota_Apicomplexa_Coccidia_Toxoplasma_gondii_GT1_
Toxoplasma_gondii__4102
672291435_Eukaryota_Apicomplexa_Coccidia_Toxoplasma_gondii_RUB_
Neospora_caninumLIV__142432
Hammondia_hammondiHH34__101067
672562612_Eukaryota_Apicomplexa_Coccidia_Toxoplasma_gondii_MAS_
Hammondia_hammondiHH34__102475
Rhytidocystis_sp2__13759
Rhytidocystis_sp2__16859
Rhytidocystis_sp2__2294
Rhytidocystis_sp2__21896
Selenidium_pygospionis__15689
Siedleckia_nematoides__44757
Siedleckia_nematoides__41657
Siedleckia_nematoides__41656
Rhytidocystis_sp__10216
Siedleckia_nematoides__54400
Siedleckia_nematoides__49670
Siedleckia_nematoides__54401
Siedleckia_nematoides__18814
Rhytidocystis_sp__2890
Cephaloidophora_communis__5697
Heliospora_caprellae__8659
Heliospora_caprellae__459
Heliospora_caprellae__9266
Heliospora_caprellae__9265
Heliospora_caprellae__9267
Heliospora_caprellae__2175
663161866_Bacteria_Actinobacteria_Actinobacteria_Corynebacterium_variabile_
652467815_Bacteria_Actinobacteria_Actinobacteria_Intrasporangiaceae_bacterium_URHB0013_
518988030_Bacteria_Actinobacteria_Actinobacteria_Terracoccus_sp__273MFTsu3_1_
495202921_Bacteria_Actinobacteria_Actinobacteria_Janibacter_hoylei_
586971879_Bacteria_Actinobacteria_Actinobacteria_Intrasporangium_chromatireducens_Q5I_
690769759_Bacteria_Actinobacteria_Actinobacteria_Corynebacterium_freneyi_DNF00450_
521978071_Bacteria_Firmicutes_Clostridia_Dehalobacter_sp__FTH1_
697976366_Bacteria_Actinobacteria_Actinobacteria_Actinotalea_fermentans_ATCC_43279__JCM_9966__DSM_3133_
545121295_Bacteria_Firmicutes_Bacilli_Bacillus_sp__EGDAK10_
Eimeria_tenellaHoughton__258409
Eimeria_brunettiHoughton__463750
Eimeria_falciformisBayerHaberkorn1970__417788
544671461_Bacteria_Actinobacteria_Actinobacteria_Propionibacterium_granulosum_
488505951_Bacteria_Actinobacteria_Actinobacteria_Propionibacterium_acnes_
695300383_Bacteria_Actinobacteria_Actinobacteria_Propionibacterium_acnes_
490241284_Bacteria_Proteobacteria_Gammaproteobacteria_Cardiobacterium_hominis_
494044323_Bacteria_Proteobacteria_Gammaproteobacteria_Cardiobacterium_valvarum_
678286005_Bacteria_Firmicutes_Bacilli_Bacillus_thermoamylovorans_
637112149_Bacteria_Proteobacteria_Gammaproteobacteria_Gilliamella_apicola_
567451236_Bacteria_Bacteroidetes_Chlorobigroup_Bacteroidetes_Tannerella_sp__oral_taxon_BU063_isolate_Cell_1_3_
Siedleckia_nematoides__65029
565651528_Bacteria_Proteobacteria_Gammaproteobacteria_Cronobacter_
504541859_Bacteria_Proteobacteria_Gammaproteobacteria_Cronobacter_sakazakii_
488992643_Bacteria_Proteobacteria_Gammaproteobacteria_Klebsiella_
506221387_Bacteria_Proteobacteria_Gammaproteobacteria_Cronobacter_turicensis_
443716355_Eukaryota_Metazoa_Annelida_Capitella_teleta_
0.3

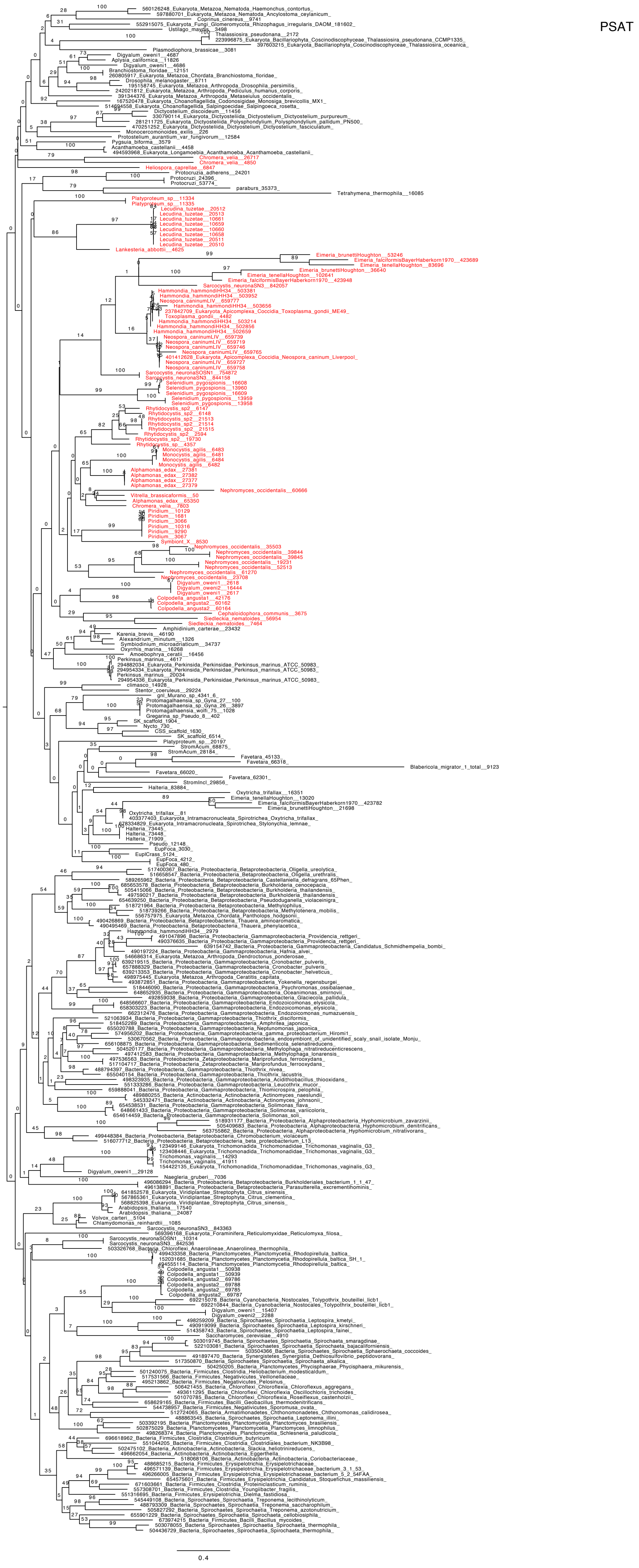
PSAT
0.4

PSD1

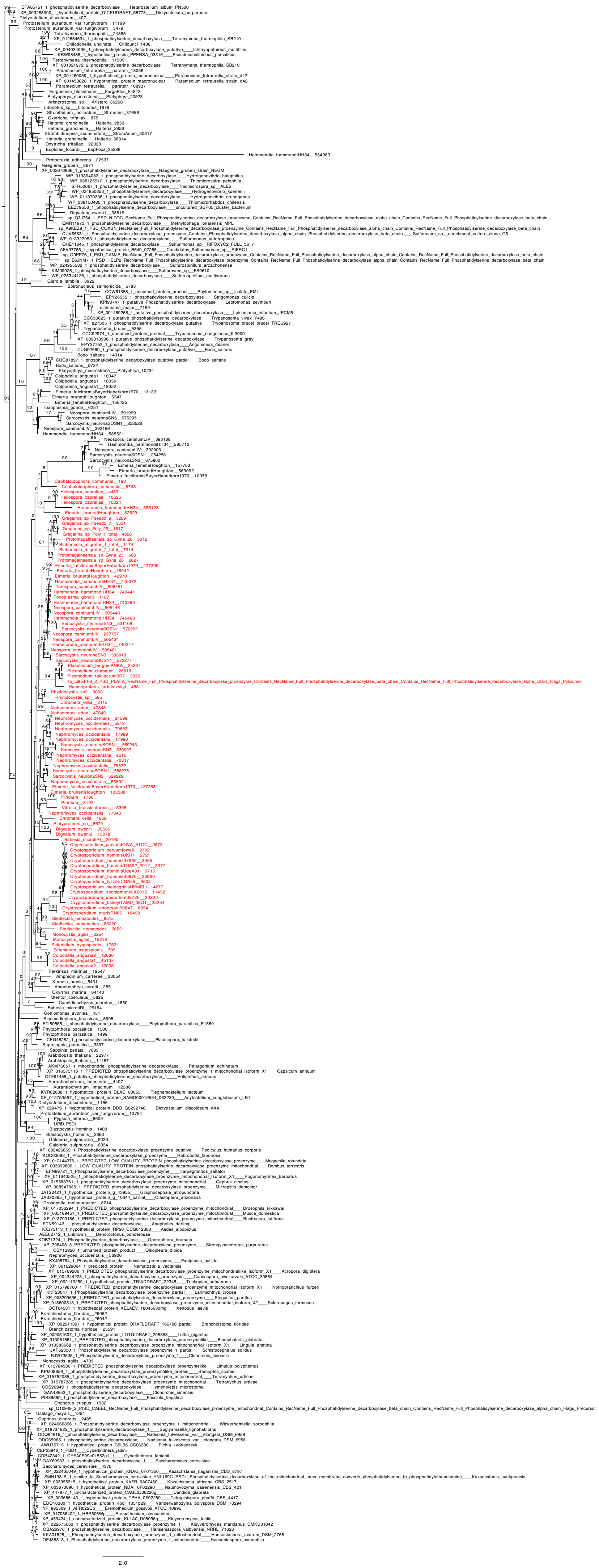

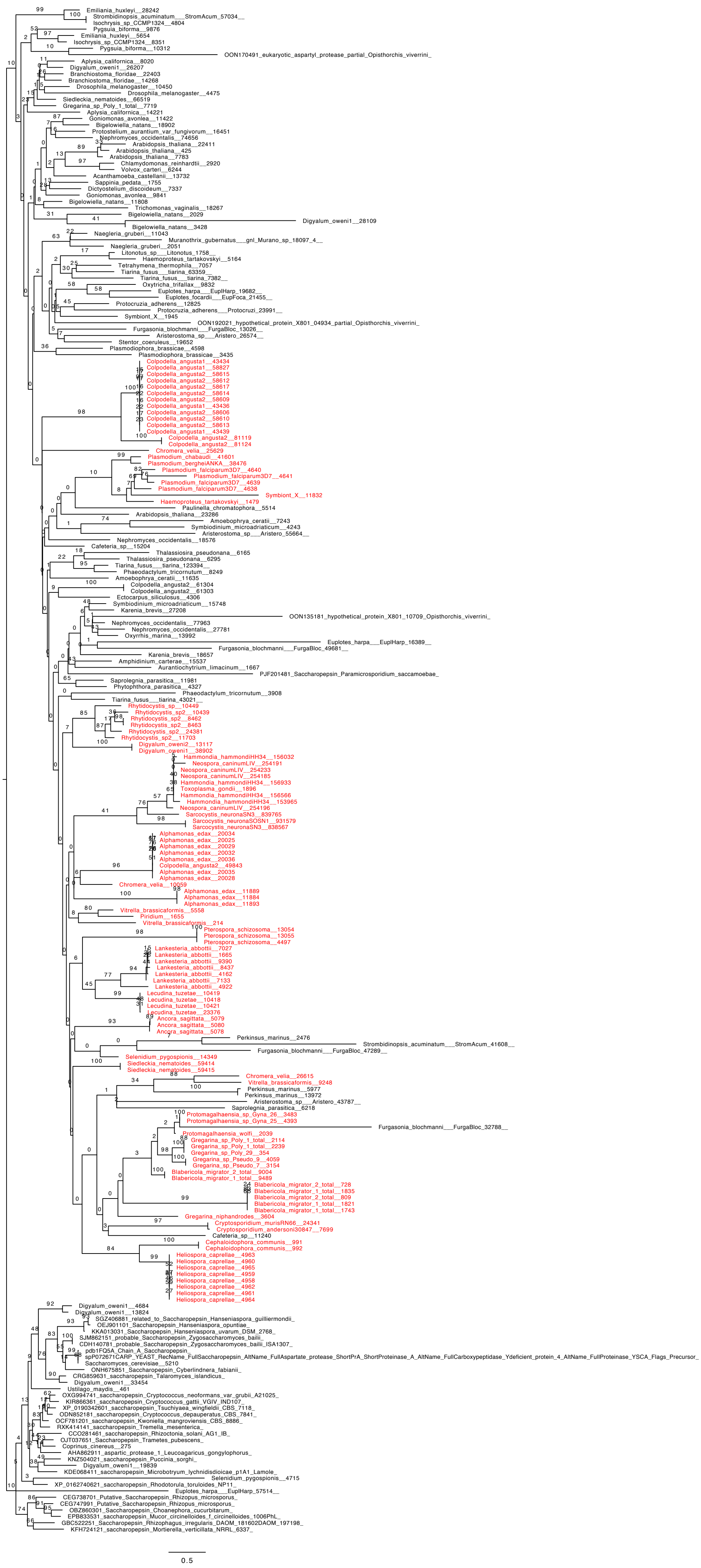

PrA
0.5

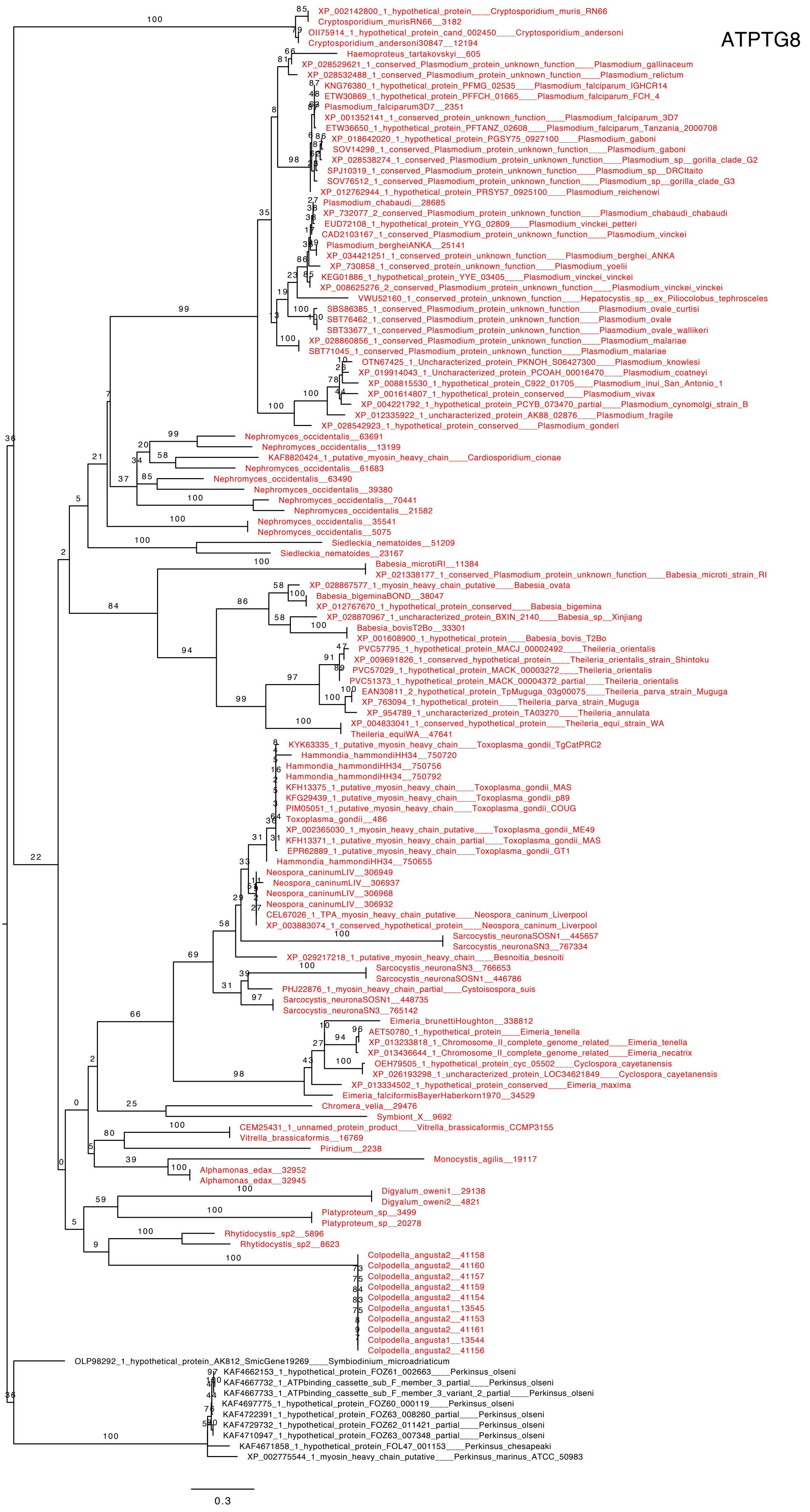
ATPTG8
XP_002142800_1_hypothetical_protein____Cryptosporidium_muris_RN66
Cryptosporidium_murisRN66__3182
OII75914_1_hypothetical_protein_cand_002450____Cryptosporidium_andersoni
Cryptosporidium_andersoni30847__12194
Haemoproteus_tartakovskyi__605
XP_028529621_1_conserved_Plasmodium_protein_unknown_function____Plasmodium_gallinaceum
XP_028532488_1_conserved_Plasmodium_protein_unknown_function____Plasmodium_relictum
KNG76380_1_hypothetical_protein_PFMG_02535____Plasmodium_falciparum_IGHCR14
ETW30869_1_hypothetical_protein_PFFCH_01665____Plasmodium_falciparum_FCH_4
Plasmodium_falciparum3D7__2351
XP_001352141_1_conserved_protein_unknown_function____Plasmodium_falciparum_3D7
ETW36650_1_hypothetical_protein_PFTANZ_02608____Plasmodium_falciparum_Tanzania_2000708
XP_018642020_1_hypothetical_protein_PGSY75_0927100____Plasmodium_gaboni
SOV14298_1_conserved_Plasmodium_protein_unknown_function____Plasmodium_gaboni
XP_028538274_1_conserved_Plasmodium_protein_unknown_function____Plasmodium_sp__gorilla_clade_G2
SPJ10319_1_conserved_Plasmodium_protein_unknown_function____Plasmodium_sp__DRCItaito
SOV76512_1_conserved_Plasmodium_protein_unknown_function____Plasmodium_sp__gorilla_clade_G3
XP_012762944_1_hypothetical_protein_PRSY57_0925100____Plasmodium_reichenowi
Plasmodium_chabaudi__28685
XP_732077_2_conserved_Plasmodium_protein_unknown_function____Plasmodium_chabaudi_chabaudi
EUD72108_1_hypothetical_protein_YYG_02809____Plasmodium_vinckei_petteri
CAD2103167_1_conserved_Plasmodium_protein_unknown_function____Plasmodium_vinckei
Plasmodium_bergheiANKA__25141
XP_034421251_1_conserved_protein_unknown_function____Plasmodium_berghei_ANKA
XP_730858_1_conserved_protein_unknown_function____Plasmodium_yoelii
KEG01886_1_hypothetical_protein_YYE_03405____Plasmodium_vinckei_vinckei
XP_008625276_2_conserved_Plasmodium_protein_unknown_function____Plasmodium_vinckei_vinckei
VWU52160_1_conserved_protein_unknown_function____Hepatocystis_sp__ex_Piliocolobus_tephrosceles
SBS86385_1_conserved_Plasmodium_protein_unknown_function____Plasmodium_ovale_curtisi
SBT76462_1_conserved_Plasmodium_protein_unknown_function____Plasmodium_ovale
SBT33677_1_conserved_Plasmodium_protein_unknown_function____Plasmodium_ovale_wallikeri
XP_028860856_1_conserved_Plasmodium_protein_unknown_function____Plasmodium_malariae
SBT71045_1_conserved_Plasmodium_protein_unknown_function____Plasmodium_malariae
OTN67425_1_Uncharacterized_protein_PKNOH_S06427300____Plasmodium_knowlesi
XP_019914043_1_Uncharacterized_protein_PCOAH_00016470____Plasmodium_coatneyi
XP_008815530_1_hypothetical_protein_C922_01705____Plasmodium_inui_San_Antonio_1
XP_001614807_1_hypothetical_protein____Plasmodium_vivax
XP_004221792_1_hypothetical_protein_PCYB_073470_partial____Plasmodium_cynomolgi_strain_B
XP_012335922_1_uncharacterized_protein_AK88_02876____Plasmodium_fragile
XP_028542923_1_hypothetical_protein_conserved____Plasmodium_gonderi
Nephromyces_occidentalis__63691
Nephromyces_occidentalis__13199
KAF8820424_1_putative_myosin_heavy_chain____Cardiosporidium_cionae
Nephromyces_occidentalis__61683
Nephromyces_occidentalis__63490
Nephromyces_occidentalis__39380
Nephromyces_occidentalis__70441
Nephromyces_occidentalis__21582
Nephromyces_occidentalis__35541
Nephromyces_occidentalis__5075
Siedleckia_nematoides__51209
Siedleckia_nematoides__23167
Babesia_microtiRI__11384
XP_021338177_1_conserved_Plasmodium_protein_unknown_function____Babesia_microti_strain_RI
XP_028867577_1_myosin_heavy_chain_putative____Babesia_ovata
Babesia_bigeminaBOND__38047
XP_012767670_1_hypothetical_protein_conserved____Babesia_bigemina
XP_028870967_1_uncharacterized_protein_BXIN_2140____Babesia_sp__Xinjiang
Babesia_bovisT2Bo__33301
XP_001608900_1_hypothetical_protein____Babesia_bovis_T2Bo
PVC57795_1_hypothetical_protein_MACJ_00002492____Theileria_orientalis
XP_009691826_1_conserved_hypothetical_protein____Theileria_orientalis_strain_Shintoku
PVC57029_1_hypothetical_protein_MACK_00003272____Theileria_orientalis
PVC51373_1_hypothetical_protein_MACK_00004372_partial____Theileria_orientalis
EAN30811_2_hypothetical_protein_TpMuguga_03g00075____Theileria_parva_strain_Muguga
XP_763094_1_hypothetical_protein____Theileria_parva_strain_Muguga
XP_954789_1_uncharacterized_protein_TA03270____Theileria_annulata
XP_004833041_1_conserved_hypothetical_protein____Theileria_equi_strain_WA
Theileria_equiWA__47641
KYK63335_1_putative_myosin_heavy_chain____Toxoplasma_gondii_TgCatPRC2
Hammondia_hammondiHH34__750720
Hammondia_hammondiHH34__750756
Hammondia_hammondiHH34__750792
KFH13375_1_putative_myosin_heavy_chain____Toxoplasma_gondii_MAS
KFG29439_1_putative_myosin_heavy_chain____Toxoplasma_gondii_p89
PIM05051_1_putative_myosin_heavy_chain____Toxoplasma_gondii_COUG
Toxoplasma_gondii__486
XP_002365030_1_myosin_heavy_chain_putative____Toxoplasma_gondii_ME49
KFH13371_1_putative_myosin_heavy_chain_partial____Toxoplasma_gondii_MAS
EPR62889_1_putative_myosin_heavy_chain____Toxoplasma_gondii_GT1
Hammondia_hammondiHH34__750655
Neospora_caninumLIV__306949
Neospora_caninumLIV__306937
Neospora_caninumLIV__306968
Neospora_caninumLIV__306932
CEL67026_1_TPA_myosin_heavy_chain_putative____Neospora_caninum_Liverpool
XP_003883074_1_conserved_hypothetical_protein____Neospora_caninum_Liverpool
Sarcocystis_neuronaSOSN1__445657
Sarcocystis_neuronaSN3__767334
XP_029217218_1_putative_myosin_heavy_chain____Besnoitia_besnoiti
Sarcocystis_neuronaSN3__766653
Sarcocystis_neuronaSOSN1__446786
PHJ22876_1_myosin_heavy_chain_partial____Cystoisospora_suis
Sarcocystis_neuronaSOSN1__448735
Sarcocystis_neuronaSN3__765142
Eimeria_brunettiHoughton__338812
AET50780_1_hypothetical_protein____Eimeria_tenella
XP_013233818_1_Chromosome_II_complete_genome_related____Eimeria_tenella
XP_013436644_1_Chromosome_II_complete_genome_related____Eimeria_necatrix
OEH79505_1_hypothetical_protein_cyc_05502____Cyclospora_cayetanensis
XP_026193298_1_uncharacterized_protein_LOC34621849____Cyclospora_cayetanensis
XP_013334502_1_hypothetical_protein_conserved____Eimeria_maxima
Eimeria_falciformisBayerHaberkorn1970__34529
Chromera_velia__29476
Symbiont_X__9692
CEM25431_1_unnamed_protein_product____Vitrella_brassicaformis_CCMP3155
Vitrella_brassicaformis__16769
Piridium__2238
Monocystis_agilis__19117
Alphamonas_edax__32952
Alphamonas_edax__32945
Digyalum_oweni1__29138
Digyalum_oweni2__4821
Platyproteum_sp__3499
Platyproteum_sp__20278
Rhytidocystis_sp2__5896
Rhytidocystis_sp2__8623
Colpodella_angusta2__41158
Colpodella_angusta2__41160
Colpodella_angusta2__41157
Colpodella_angusta2__41159
Colpodella_angusta2__41154
Colpodella_angusta1__13545
Colpodella_angusta2__41153
Colpodella_angusta2__41161
Colpodella_angusta1__13544
Colpodella_angusta2__41156
OLP98292_1_hypothetical_protein_AK812_SmicGene19269____Symbiodinium_microadriaticum
KAF4662153_1_hypothetical_protein_FOZ61_002663____Perkinsus_olseni
KAF4667732_1_ATPbinding_cassette_sub_F_member_3_partial____Perkinsus_olseni
KAF4667733_1_ATPbinding_cassette_sub_F_member_3_variant_2_partial____Perkinsus_olseni
KAF4697775_1_hypothetical_protein_FOZ60_000119____Perkinsus_olseni
KAF4722391_1_hypothetical_protein_FOZ63_008260_partial____Perkinsus_olseni
KAF4729732_1_hypothetical_protein_FOZ62_011421_partial____Perkinsus_olseni
KAF4710947_1_hypothetical_protein_FOZ63_007348_partial____Perkinsus_olseni
KAF4671858_1_hypothetical_protein_FOL47_001153____Perkinsus_chesapeaki
XP_002775544_1_myosin_heavy_chain_putative____Perkinsus_marinus_ATCC_50983
0.3

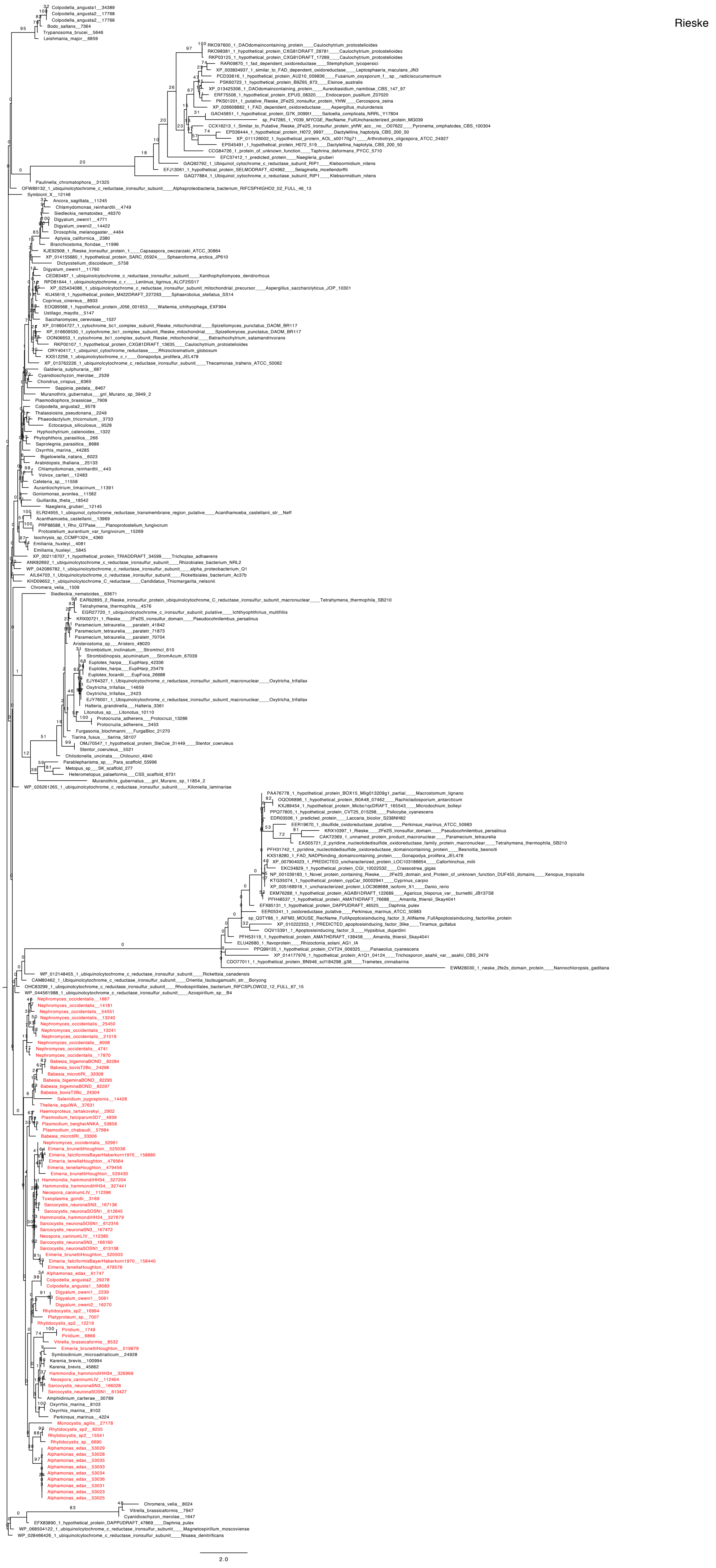
Rieske
2.0

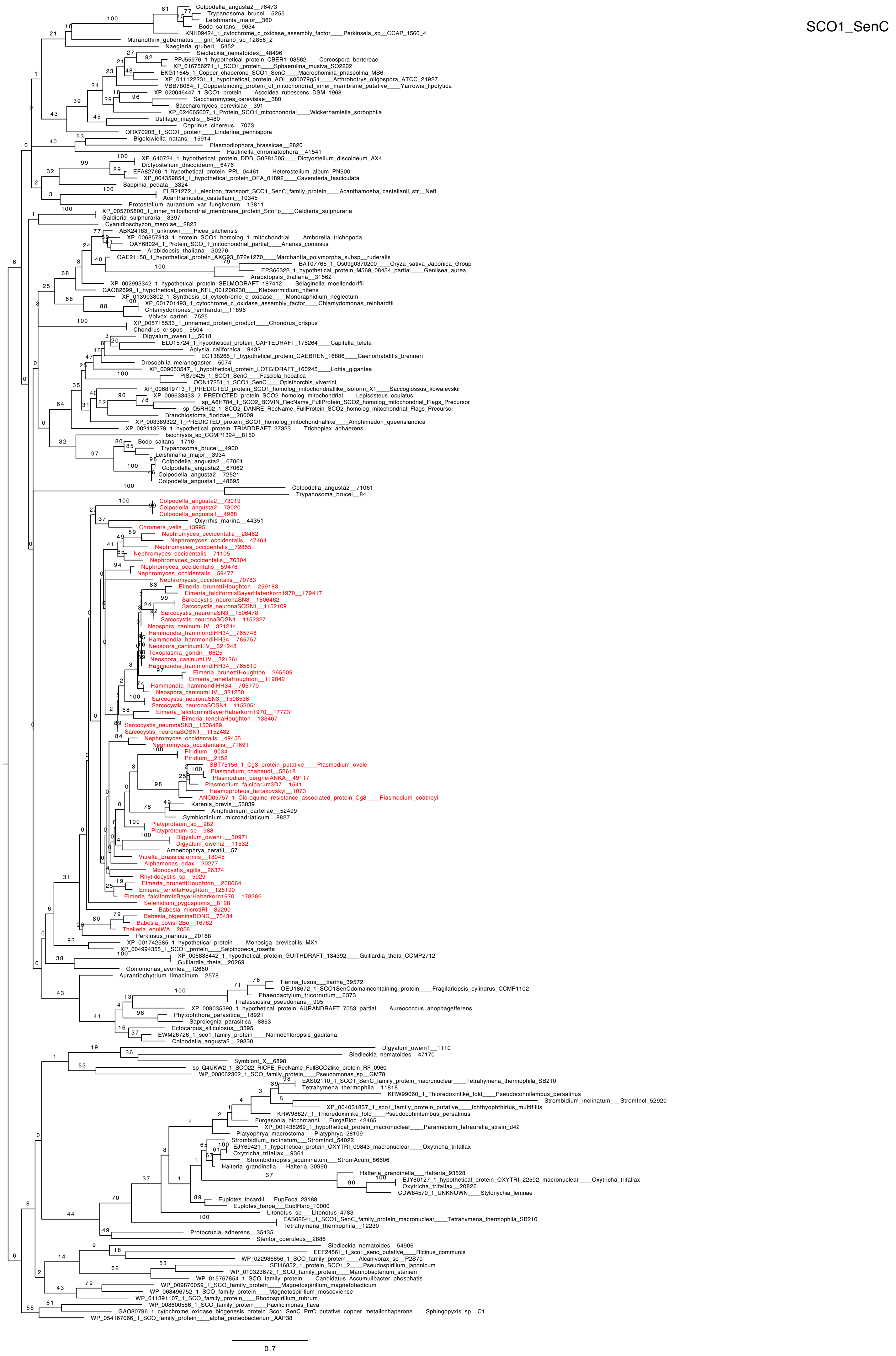
SCO1_SenC
Colpodella_angusta2__76473
Trypanosoma_brucei__5255
Leishmania_major__360
Bodo_saltans__9634
KNH09424_1_cytochrome_c_oxidase_assembly_factor____Perkinsela_sp__CCAP_1560_4
Muranothrix_gubernatus___gnl_Murano_sp_12856_2
Naegleria_gruberi__5452
Siedleckia_nematoides__48496
PPJ55976_1_hypothetical_protein_CBER1_03562____Cercospora_berteroae
XP_016756271_1_SCO1_protein____Sphaerulina_musiva_SO2202
EKG11645_1_Copper_chaperone_SCO1_SenC____Macrophomina_phaseolina_MS6
XP_011122231_1_hypothetical_protein_AOL_s00079g54____Arthrobotrys_oligospora_ATCC_24927
VBB78084_1_Copperbinding_protein_of_mitochondrial_inner_membrane_putative____Yarrowia_lipolytica
XP_020046447_1_SCO1_protein____Ascoidea_rubescens_DSM_1968
Saccharomyces_cerevisiae__380
Saccharomyces_cerevisiae__391
XP_024665607_1_Protein_SCO1_mitochondrial____Wickerhamiella_sorbophila
Ustilago_maydis__6480
Coprinus_cinereus__7073
ORX70203_1_SCO1_protein____Linderina_pennispora
Bigelowiella_natans__15914
Plasmodiophora_brassicae__2820
Paulinella_chromatophora__41541
XP_640724_1_hypothetical_protein_DDB_G0281505____Dictyostelium_discoideum_AX4
Dictyostelium_discoideum__6476
EFA82766_1_hypothetical_protein_PPL_04461____Heterostelium_album_PN500
XP_004359854_1_hypothetical_protein_DFA_01892____Cavenderia_fasciculata
Sappinia_pedata__3324
ELR21272_1_electron_transport_SCO1_SenC_family_protein____Acanthamoeba_castellanii_str__Neff
Acanthamoeba_castellanii__10345
Protostelium_aurantium_var_fungivorum__13811
XP_005705800_1_inner_mitochondrial_membrane_protein_Sco1p____Galdieria_sulphuraria
Galdieria_sulphuraria__3397
Cyanidioschyzon_merolae__2823
ABK24183_1_unknown____Picea_sitchensis
XP_006857913_1_protein_SCO1_homolog_1_mitochondrial____Amborella_trichopoda
OAY68024_1_Protein_SCO_1_mitochondrial_partial____Ananas_comosus
Arabidopsis_thaliana__30276
OAE21158_1_hypothetical_protein_AXG93_872s1270____Marchantia_polymorpha_subsp__ruderalis
BAT07765_1_Os09g0370200____Oryza_sativa_Japonica_Group
EPS66322_1_hypothetical_protein_M569_08454_partial____Genlisea_aurea
Arabidopsis_thaliana__31562
XP_002993342_1_hypothetical_protein_SELMODRAFT_187412____Selaginella_moellendorffii
GAQ82699_1_hypothetical_protein_KFL_001200230____Klebsormidium_nitens
XP_013903802_1_Synthesis_of_cytochrome_c_oxidase____Monoraphidium_neglectum
XP_001701493_1_cytochrome_c_oxidase_assembly_factor____Chlamydomonas_reinhardtii
Chlamydomonas_reinhardtii__11896
Volvox_carteri__7525
XP_005715533_1_unnamed_protein_product____Chondrus_crispus
Chondrus_crispus__5504
Digyalum_oweni1__5018
ELU15724_1_hypothetical_protein_CAPTEDRAFT_175264____Capitella_teleta
Aplysia_californica__9432
EGT38268_1_hypothetical_protein_CAEBREN_16886____Caenorhabditis_brenneri
Drosophila_melanogaster__5074
XP_009053547_1_hypothetical_protein_LOTGIDRAFT_160245____Lottia_gigantea
PIS79425_1_SCO1_SenC____Fasciola_hepatica
OON17251_1_SCO1_SenC____Opisthorchis_viverrini
XP_006819713_1_PREDICTED_protein_SCO1_homolog_mitochondriallike_isoform_X1____Saccoglossus_kowalevskii
XP_006633433_2_PREDICTED_protein_SCO2_homolog_mitochondrial____Lepisosteus_oculatus
sp_A6H784_1_SCO2_BOVIN_RecName_FullProtein_SCO2_homolog_mitochondrial_Flags_Precursor
sp_Q5RH02_1_SCO2_DANRE_RecName_FullProtein_SCO2_homolog_mitochondrial_Flags_Precursor
Branchiostoma_floridae__28009
XP_003389322_1_PREDICTED_protein_SCO1_homolog_mitochondriallike____Amphimedon_queenslandica
XP_002113379_1_hypothetical_protein_TRIADDRAFT_27323____Trichoplax_adhaerens
Isochrysis_sp_CCMP1324__8150
Bodo_saltans__1716
Trypanosoma_brucei__4900
Leishmania_major__3934
Colpodella_angusta2__67061
Colpodella_angusta2__67062
Colpodella_angusta2__72521
Colpodella_angusta1__48695
Colpodella_angusta2__71061
Trypanosoma_brucei__84
Colpodella_angusta2__73019
Colpodella_angusta2__73020
Colpodella_angusta1__4988
Oxyrrhis_marina__44351
Chromera_velia__13995
Nephromyces_occidentalis__28462
Nephromyces_occidentalis__47464
Nephromyces_occidentalis__72855
Nephromyces_occidentalis__71105
Nephromyces_occidentalis__76304
Nephromyces_occidentalis__59478
Nephromyces_occidentalis__59477
Nephromyces_occidentalis__70783
Eimeria_brunettiHoughton__259183
Eimeria_falciformisBayerHaberkorn1970__179417
Sarcocystis_neuronaSN3__1506462
Sarcocystis_neuronaSOSN1__1152109
Sarcocystis_neuronaSN3__1506478
Sarcocystis_neuronaSOSN1__1152327
Neospora_caninumLIV__321244
Hammondia_hammondiHH34__765748
Hammondia_hammondiHH34__765757
Neospora_caninumLIV__321248
Toxoplasma_gondii__6625
Neospora_caninumLIV__321261
Hammondia_hammondiHH34__765810
Eimeria_brunettiHoughton__265509
Eimeria_tenellaHoughton__119842
Hammondia_hammondiHH34__765770
Neospora_caninumLIV__321250
Sarcocystis_neuronaSN3__1506536
Sarcocystis_neuronaSOSN1__1153051
Eimeria_falciformisBayerHaberkorn1970__177231
Eimeria_tenellaHoughton__133467
Sarcocystis_neuronaSN3__1506489
Sarcocystis_neuronaSOSN1__1152482
Nephromyces_occidentalis__49455
Nephromyces_occidentalis__71691
Piridium__9034
Piridium__2152
SBT75156_1_Cg3_protein_putative____Plasmodium_ovale
Plasmodium_chabaudi__52618
Plasmodium_bergheiANKA__49117
Plasmodium_falciparum3D7__1541
Haemoproteus_tartakovskyi__1073
ANQ05757_1_Cloroquine_resistance_associated_protein_Cg3____Plasmodium_coatneyi
Karenia_brevis__53039
Amphidinium_carterae__52499
Symbiodinium_microadriaticum__8827
Platyproteum_sp__982
Platyproteum_sp__983
Digyalum_oweni1__30971
Digyalum_oweni2__11532
Amoebophrya_ceratii__57
Vitrella_brassicaformis__18045
Alphamonas_edax__20277
Monocystis_agilis__26374
Rhytidocystis_sp__5929
Eimeria_brunettiHoughton__268664
Eimeria_tenellaHoughton__126190
Eimeria_falciformisBayerHaberkorn1970__178386
Selenidium_pygospionis__8128
Babesia_microtiRI__32290
Babesia_bigeminaBOND__75434
Babesia_bovisT2Bo__16782
Theileria_equiWA__2058
Perkinsus_marinus__20168
XP_001742585_1_hypothetical_protein____Monosiga_brevicollis_MX1
XP_004994355_1_SCO1_protein____Salpingoeca_rosetta
XP_005838442_1_hypothetical_protein_GUITHDRAFT_134392____Guillardia_theta_CCMP2712
Guillardia_theta__20269
Goniomonas_avonlea__12660
Aurantiochytrium_limacinum__2578
Tiarina_fusus___tiarina_39572
OEU18672_1_SCO1SenCdomaincontaining_protein____Fragilariopsis_cylindrus_CCMP1102
Phaeodactylum_tricornutum__6373
Thalassiosira_pseudonana__995
XP_009035390_1_hypothetical_protein_AURANDRAFT_7053_partial____Aureococcus_anophagefferens
Phytophthora_parasitica__18921
Saprolegnia_parasitica__8853
Ectocarpus_siliculosus__3395
EWM26726_1_sco1_family_protein____Nannochloropsis_gaditana
Colpodella_angusta2__29830
Digyalum_oweni1__1110
Siedleckia_nematoides__47170
Symbiont_X__6898
sp_Q4UKW2_1_SCO22_RICFE_RecName_FullSCO2like_protein_RF_0960
WP_008062302_1_SCO_family_protein____Pseudomonas_sp__GM78
EAS02110_1_SCO1_SenC_family_protein_macronuclear____Tetrahymena_thermophila_SB210
Tetrahymena_thermophila__11818
KRW99060_1_Thioredoxinlike_fold____Pseudocohnilembus_persalinus
Strombidium_inclinatum___StromIncl_52920
XP_004031837_1_sco1_family_protein_putative____Ichthyophthirius_multifiliis
KRW98827_1_Thioredoxinlike_fold____Pseudocohnilembus_persalinus
Furgasonia_blochmanni___FurgaBloc_42465
XP_001438269_1_hypothetical_protein_macronuclear____Paramecium_tetraurelia_strain_d42
Platyophrya_macrostoma___Platyphrya_28109
Strombidium_inclinatum___StromIncl_54022
EJY69421_1_hypothetical_protein_OXYTRI_09843_macronuclear____Oxytricha_trifallax
Oxytricha_trifallax__9361
Strombidinopsis_acuminatum___StromAcum_86606
Halteria_grandinella___Halteria_30990
Halteria_grandinella___Halteria_93528
EJY80127_1_hypothetical_protein_OXYTRI_22592_macronuclear____Oxytricha_trifallax
Oxytricha_trifallax__20826
CDW84570_1_UNKNOWN____Stylonychia_lemnae
Euplotes_focardii___EupFoca_23188
Euplotes_harpa___EuplHarp_10000
Litonotus_sp___Litonotus_4783
EAS02641_1_SCO1_SenC_family_protein_macronuclear____Tetrahymena_thermophila_SB210
Tetrahymena_thermophila__12230
Protocruzia_adherens__35435
Stentor_coeruleus__2886
Siedleckia_nematoides__54906
EEF24561_1_sco1_senc_putative____Ricinus_communis
WP_022986856_1_SCO_family_protein____Alcanivorax_sp__P2S70
SEI46852_1_protein_SCO1_2____Pseudospirillum_japonicum
WP_010323672_1_SCO_family_protein____Marinobacterium_stanieri
WP_015767854_1_SCO_family_protein____Candidatus_Accumulibacter_phosphatis
WP_009870059_1_SCO_family_protein____Magnetospirillum_magnetotacticum
WP_068496752_1_SCO_family_protein____Magnetospirillum_moscoviense
WP_011391107_1_SCO_family_protein____Rhodospirillum_rubrum
WP_008600586_1_SCO_family_protein____Pacificimonas_flava
GAO80796_1_cytochrome_oxidase_biogenesis_protein_Sco1_SenC_PrrC_putative_copper_metallochaperone____Sphingopyxis_sp__C1
WP_054167066_1_SCO_family_protein____alpha_proteobacterium_AAP38
0.7

SCSa

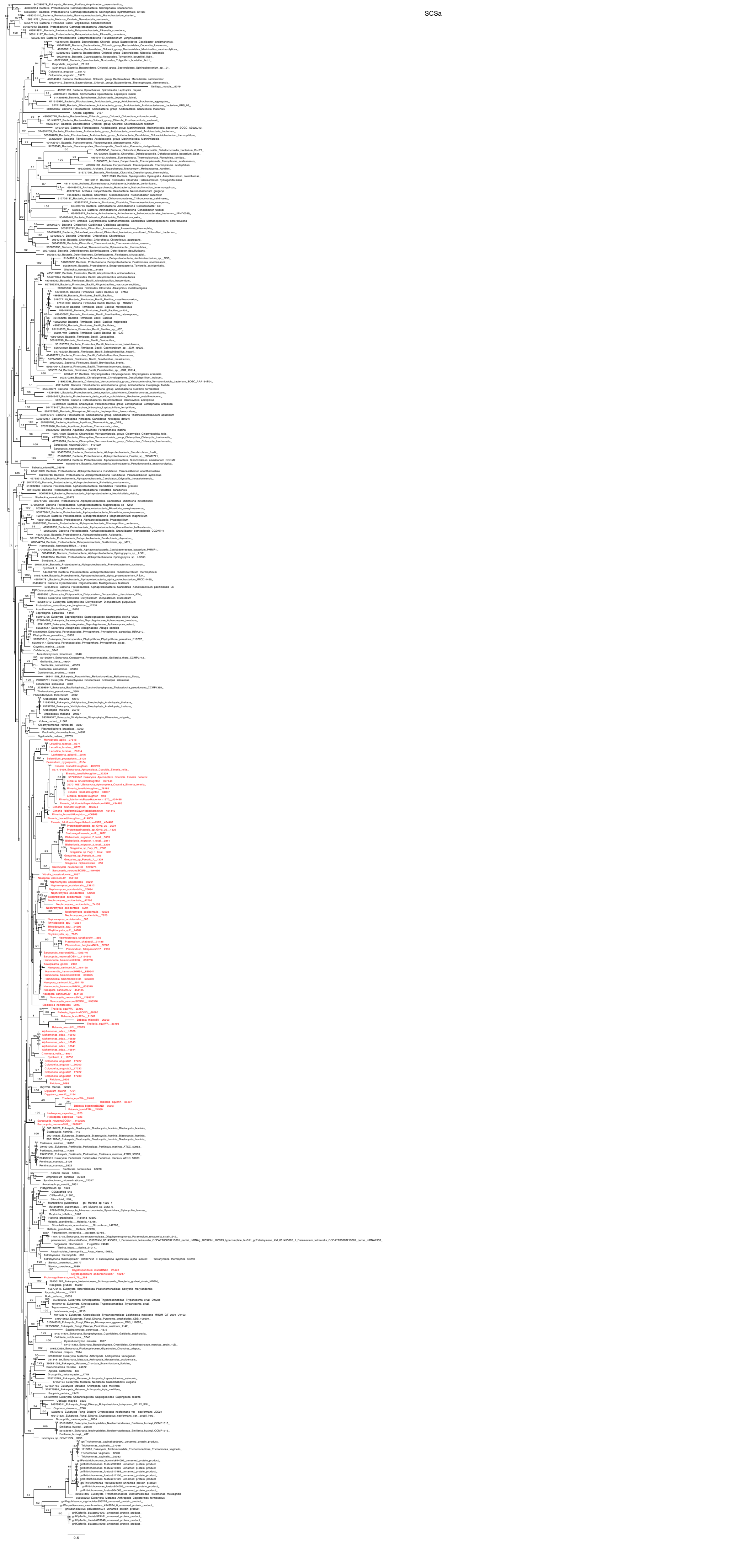

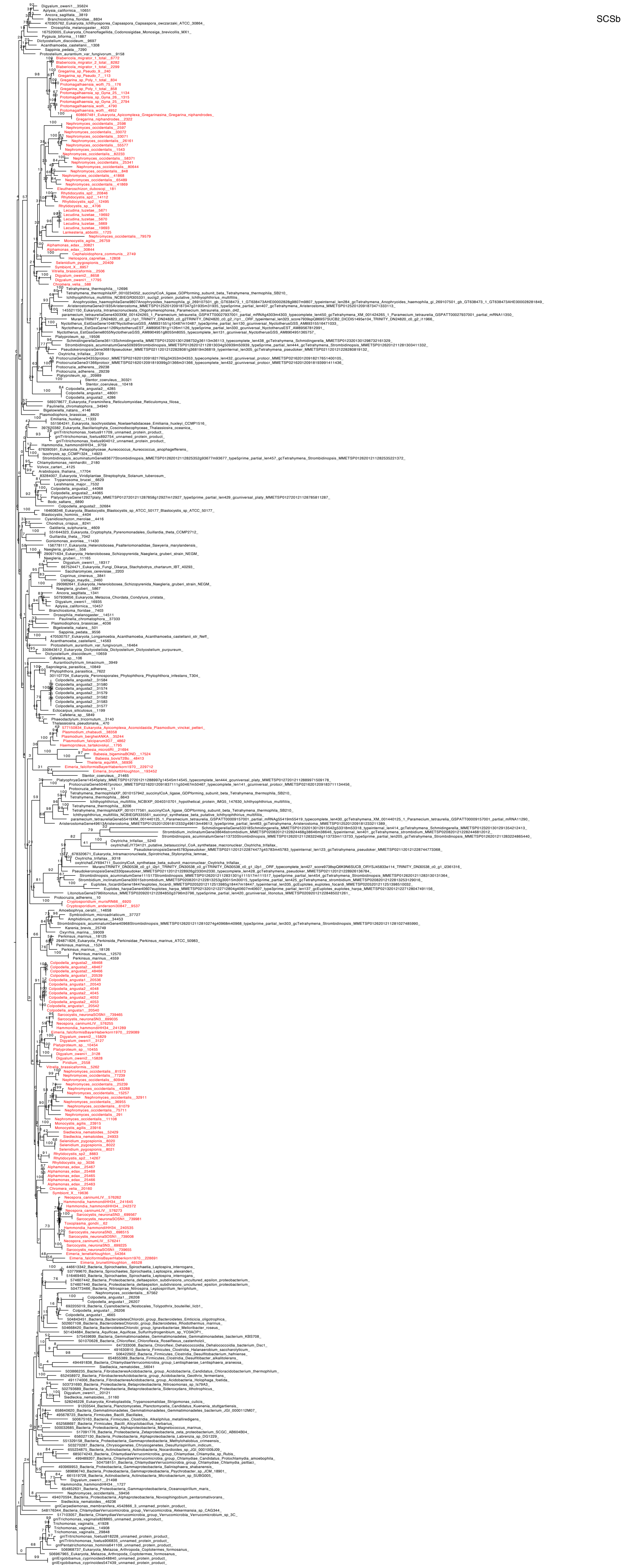
SCSb
0.6

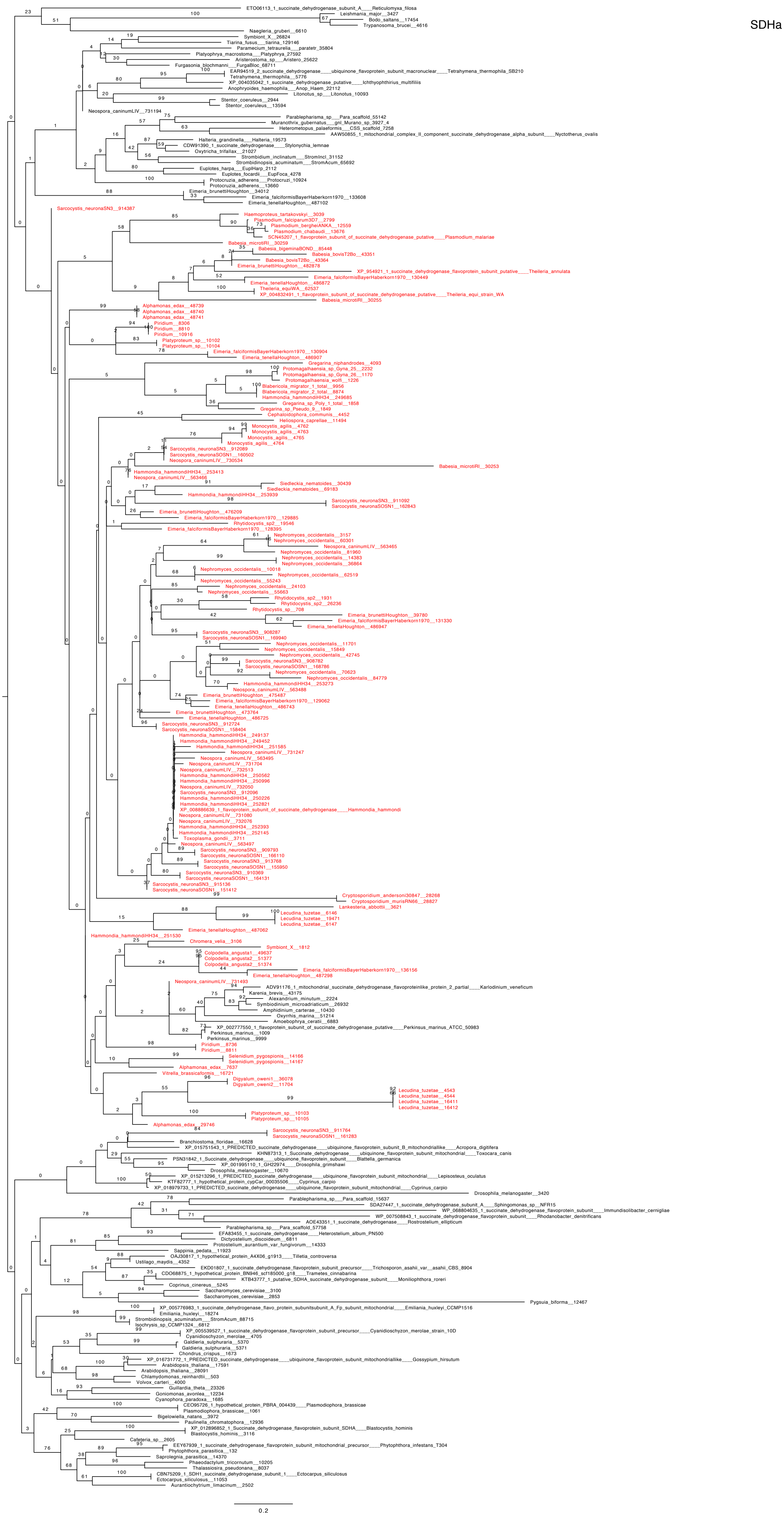
SDHa
0.2

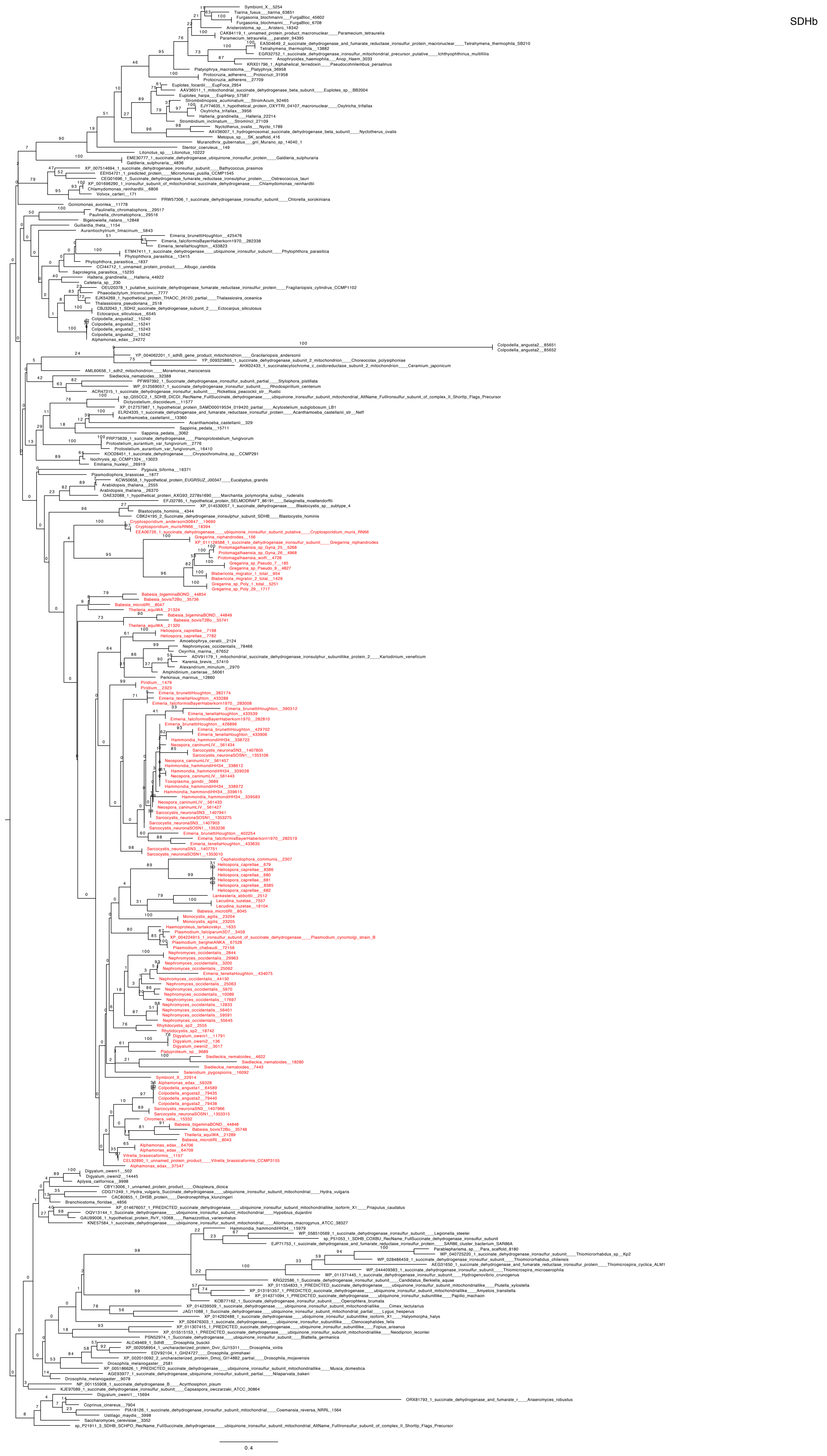
SDHb
0.4

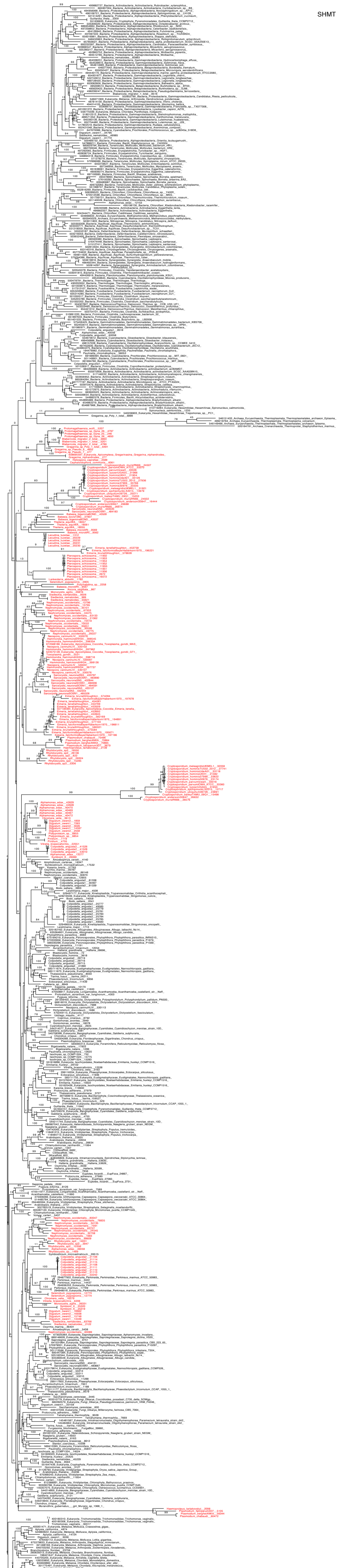
SHMT

YFH1

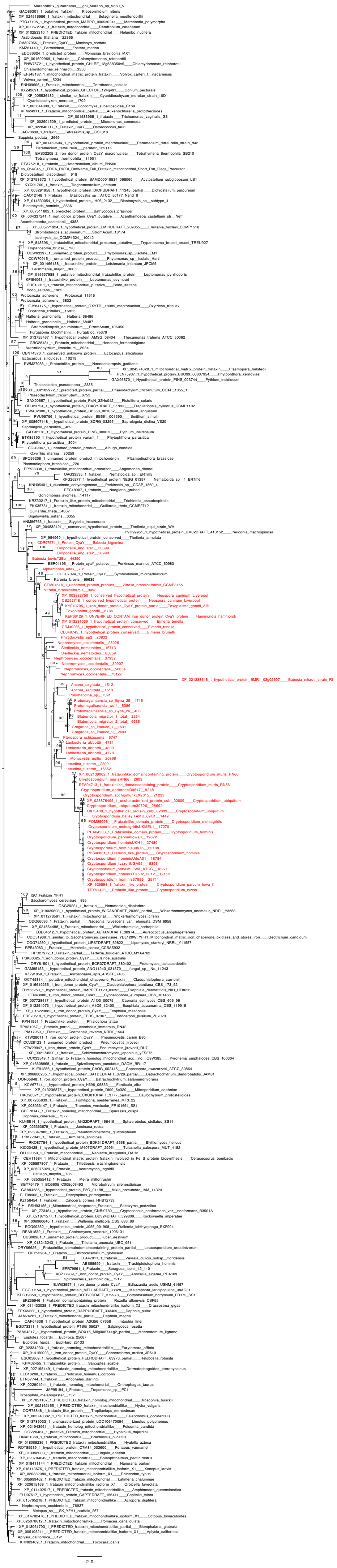

cob

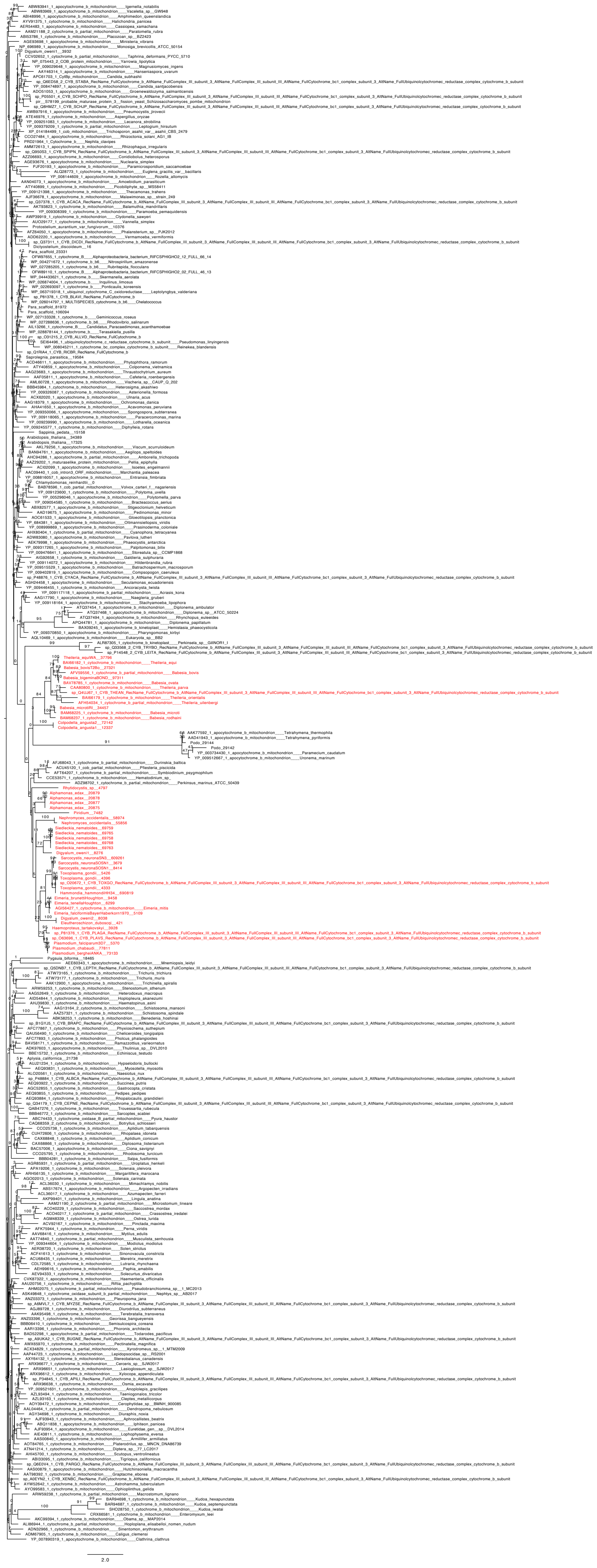

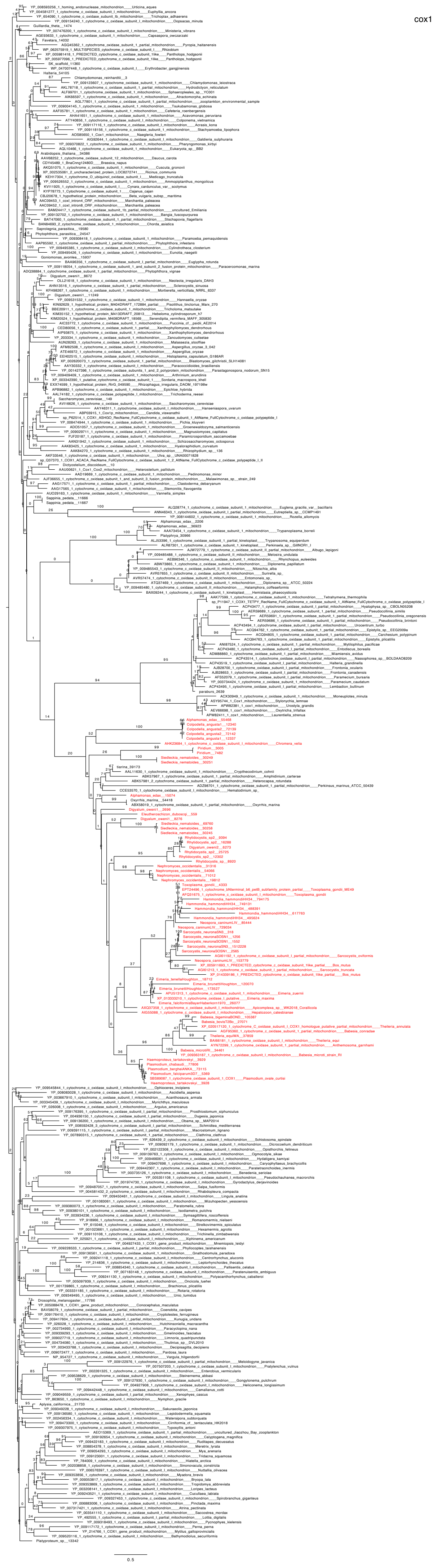
cox1
0.5

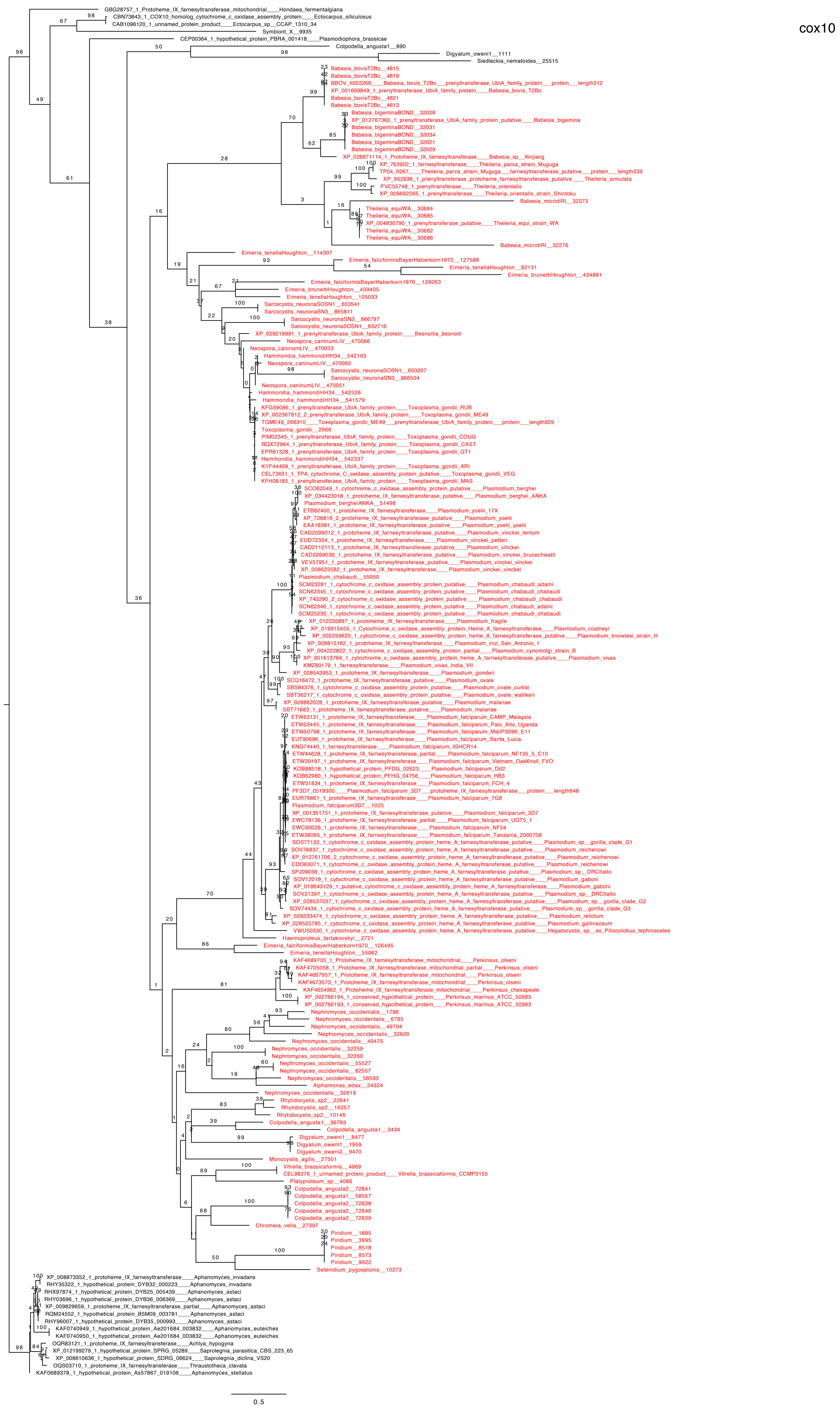

cox10
GBG28757_1_Protoheme_IX_farnesyltransferase_mitochondrial____Hondaea_fermentalgiana
CBN73843_1_COX10_homolog_cytochrome_c_oxidase_assembly_protein____Ectocarpus_siliculosus
CAB1096120_1_unnamed_protein_product____Ectocarpus_sp__CCAP_1310_34
Symbiont_X__9935
CEP00364_1_hypothetical_protein_PBRA_001418____Plasmodiophora_brassicae
Colpodella_angusta1__690
Digyalum_oweni1__1111
Siedleckia_nematoides__25515
Babesia_bovisT2Bo__4815
Babesia_bovisT2Bo__4819
BBOV_II003260____Babesia_bovis_T2Bo___prenyltransferase_UbiA_family_protein___protein___length312
XP_001609849_1_prenyltransferase_UbiA_family_protein____Babesia_bovis_T2Bo
Babesia_bovisT2Bo__4821
Babesia_bovisT2Bo__4813
Babesia_bigeminaBOND__32028
XP_012767360_1_prenyltransferase_UbiA_family_protein_putative____Babesia_bigemina
Babesia_bigeminaBOND__32031
Babesia_bigeminaBOND__32034
Babesia_bigeminaBOND__32021
Babesia_bigeminaBOND__32029
XP_028871114_1_Protoheme_IX_farnesyltransferase____Babesia_sp__Xinjiang
XP_763902_1_farnesyltransferase____Theileria_parva_strain_Muguga
TP04_0267____Theileria_parva_strain_Muguga___farnesyltransferase_putative___protein___length339
XP_952936_1_prenyltransferase_protoheme_farnesyltransferase_putative____Theileria_annulata
PVC55748_1_prenyltransferase____Theileria_orientalis
XP_009692565_1_prenyltransferase____Theileria_orientalis_strain_Shintoku
Babesia_microtiRI__32273
Theileria_equiWA__30684
Theileria_equiWA__30685
XP_004830790_1_prenyltransferase_putative____Theileria_equi_strain_WA
Theileria_equiWA__30682
Theileria_equiWA__30686
Babesia_microtiRI__32276
Eimeria_tenellaHoughton__114307
Eimeria_falciformisBayerHaberkorn1970__127586
Eimeria_tenellaHoughton__82131
Eimeria_brunettiHoughton__434881
Eimeria_falciformisBayerHaberkorn1970__129263
Eimeria_brunettiHoughton__439405
Eimeria_tenellaHoughton__105033
Sarcocystis_neuronaSOSN1__653541
Sarcocystis_neuronaSN3__865811
Sarcocystis_neuronaSN3__866797
Sarcocystis_neuronaSOSN1__652716
XP_029219991_1_prenyltransferase_UbiA_family_protein____Besnoitia_besnoiti
Neospora_caninumLIV__470066
Neospora_caninumLIV__470033
Hammondia_hammondiHH34__542105
Neospora_caninumLIV__470060
Sarcocystis_neuronaSOSN1__653207
Sarcocystis_neuronaSN3__866504
Neospora_caninumLIV__470051
Hammondia_hammondiHH34__542526
Hammondia_hammondiHH34__541579
KFG59086_1_prenyltransferase_UbiA_family_protein____Toxoplasma_gondii_RUB
XP_002367812_2_prenyltransferase_UbiA_family_protein____Toxoplasma_gondii_ME49
TGME49_206310____Toxoplasma_gondii_ME49___prenyltransferase_UbiA_family_protein___protein___length929
Toxoplasma_gondii__2066
PIM02345_1_prenyltransferase_UbiA_family_protein____Toxoplasma_gondii_COUG
RQX72964_1_prenyltransferase_UbiA_family_protein____Toxoplasma_gondii_CAST
EPR61328_1_prenyltransferase_UbiA_family_protein____Toxoplasma_gondii_GT1
Hammondia_hammondiHH34__542337
KYF44499_1_prenyltransferase_UbiA_family_protein____Toxoplasma_gondii_ARI
CEL73631_1_TPA_cytochrome_C_oxidase_assembly_protein_putative____Toxoplasma_gondii_VEG
KFH08185_1_prenyltransferase_UbiA_family_protein____Toxoplasma_gondii_MAS
SCO62049_1_cytochrome_c_oxidase_assembly_protein_putative____Plasmodium_berghei
XP_034423018_1_protoheme_IX_farnesyltransferase_putative____Plasmodium_berghei_ANKA
Plasmodium_bergheiANKA__51498
ETB62400_1_protoheme_IX_farnesyltransferase____Plasmodium_yoelii_17X
XP_726816_2_protoheme_IX_farnesyltransferase_putative____Plasmodium_yoelii
EAA18381_1_protoheme_IX_farnesyltransferase_putative____Plasmodium_yoelii_yoelii
CAD2099012_1_protoheme_IX_farnesyltransferase_putative____Plasmodium_vinckei_lentum
EUD72334_1_protoheme_IX_farnesyltransferase____Plasmodium_vinckei_petteri
CAD2110113_1_protoheme_IX_farnesyltransferase_putative____Plasmodium_vinckei
CAD2099039_1_protoheme_IX_farnesyltransferase_putative____Plasmodium_vinckei_brucechwatti
VEV57951_1_protoheme_IX_farnesyltransferase_putative____Plasmodium_vinckei_vinckei
XP_008623582_1_protoheme_IX_farnesyltransferase____Plasmodium_vinckei_vinckei
Plasmodium_chabaudi__55050
SCM23281_1_cytochrome_c_oxidase_assembly_protein_putative____Plasmodium_chabaudi_adami
SCN62345_1_cytochrome_c_oxidase_assembly_protein_putative____Plasmodium_chabaudi_chabaudi
XP_743290_2_cytochrome_c_oxidase_assembly_protein_putative____Plasmodium_chabaudi_chabaudi
SCN62346_1_cytochrome_c_oxidase_assembly_protein_putative____Plasmodium_chabaudi_adami
SCM25230_1_cytochrome_c_oxidase_assembly_protein_putative____Plasmodium_chabaudi_chabaudi
XP_012335897_1_protoheme_IX_farnesyltransferase____Plasmodium_fragile
XP_019915455_1_Cytochrome_c_oxidase_assembly_protein_Heme_A_farnesyltransferase____Plasmodium_coatneyi
XP_002259620_1_cytochrome_c_oxidase_assembly_protein_heme_A_farnesyltransferase_putative____Plasmodium_knowlesi_strain_H
XP_008815162_1_protoheme_IX_farnesyltransferase____Plasmodium_inui_San_Antonio_1
XP_004222822_1_cytochrome_c_oxidase_assembly_protein_partial____Plasmodium_cynomolgi_strain_B
XP_001613764_1_cytochrome_c_oxidase_assembly_protein_heme_A_farnesyltransferase_putative____Plasmodium_vivax
KMZ80179_1_farnesyltransferase____Plasmodium_vivax_India_VII
XP_028543953_1_protoheme_IX_farnesyltransferase____Plasmodium_gonderi
SCQ16472_1_protoheme_IX_farnesyltransferase_putative____Plasmodium_ovale
SBS84376_1_cytochrome_c_oxidase_assembly_protein_putative____Plasmodium_ovale_curtisi
SBT36217_1_cytochrome_c_oxidase_assembly_protein_putative____Plasmodium_ovale_wallikeri
XP_028862028_1_protoheme_IX_farnesyltransferase_putative____Plasmodium_malariae
SBT71683_1_protoheme_IX_farnesyltransferase_putative____Plasmodium_malariae
ETW63131_1_protoheme_IX_farnesyltransferase____Plasmodium_falciparum_CAMP_Malaysia
ETW53445_1_protoheme_IX_farnesyltransferase____Plasmodium_falciparum_Palo_Alto_Uganda
ETW50798_1_protoheme_IX_farnesyltransferase____Plasmodium_falciparum_MaliPS096_E11
EUT90696_1_protoheme_IX_farnesyltransferase____Plasmodium_falciparum_Santa_Lucia
KNG74440_1_farnesyltransferase____Plasmodium_falciparum_IGHCR14
ETW44628_1_protoheme_IX_farnesyltransferase_partial____Plasmodium_falciparum_NF135_5_C10
ETW20197_1_protoheme_IX_farnesyltransferase____Plasmodium_falciparum_Vietnam_OakKnoll_FVO
KOB88518_1_hypothetical_protein_PFDG_02623____Plasmodium_falciparum_Dd2
KOB62980_1_hypothetical_protein_PFHG_04756____Plasmodium_falciparum_HB3
ETW31834_1_protoheme_IX_farnesyltransferase____Plasmodium_falciparum_FCH_4
PF3D7_0519300____Plasmodium_falciparum_3D7___protoheme_IX_farnesyltransferase___protein___length648
EUR76661_1_protoheme_IX_farnesyltransferase____Plasmodium_falciparum_7G8
Plasmodium_falciparum3D7__1025
XP_001351751_1_protoheme_IX_farnesyltransferase_putative____Plasmodium_falciparum_3D7
EWC78136_1_protoheme_IX_farnesyltransferase_partial____Plasmodium_falciparum_UGT5_1
EWC90026_1_protoheme_IX_farnesyltransferase____Plasmodium_falciparum_NF54
ETW38065_1_protoheme_IX_farnesyltransferase____Plasmodium_falciparum_Tanzania_2000708
SOS77122_1_cytochrome_c_oxidase_assembly_protein_heme_A_farnesyltransferase_putative____Plasmodium_sp__gorilla_clade_G1
SOV76837_1_cytochrome_c_oxidase_assembly_protein_heme_A_farnesyltransferase_putative____Plasmodium_reichenowi
XP_012761706_2_cytochrome_c_oxidase_assembly_protein_heme_A_farnesyltransferase_putative____Plasmodium_reichenowi
CDO63071_1_cytochrome_c_oxidase_assembly_protein_heme_A_farnesyltransferase_putative____Plasmodium_reichenowi
SPJ09038_1_cytochrome_c_oxidase_assembly_protein_heme_A_farnesyltransferase_putative____Plasmodium_sp__DRC-Itaito
SOV12019_1_cytochrome_c_oxidase_assembly_protein_heme_A_farnesyltransferase_putative____Plasmodium_gaboni
XP_018643129_1_putative_cytochrome_c_oxidase_assembly_protein_heme_A_farnesyltransferase____Plasmodium_gaboni
SOV21397_1_cytochrome_c_oxidase_assembly_protein_heme_A_farnesyltransferase_putative____Plasmodium_sp__DRC-Itaito
XP_028537037_1_cytochrome_c_oxidase_assembly_protein_heme_A_farnesyltransferase_putative____Plasmodium_sp__gorilla_clade_G2
SOV74434_1_cytochrome_c_oxidase_assembly_protein_heme_A_farnesyltransferase_putative____Plasmodium_sp__gorilla_clade_G3
XP_028533474_1_cytochrome_c_oxidase_assembly_protein_heme_A_farnesyltransferase_putative____Plasmodium_relictum
XP_028525785_1_cytochrome_c_oxidase_assembly_protein_heme_A_farnesyltransferase_putative____Plasmodium_gallinaceum
VWU50330_1_cytochrome_c_oxidase_assembly_protein_heme_A_farnesyltransferase_putative____Hepatocystis_sp__ex_Piliocolobus_tephrosceles
Haemoproteus_tartakovskyi__2721
Eimeria_falciformisBayerHaberkorn1970__126495
Eimeria_tenellaHoughton__55062
KAF4689735_1_Protoheme_IX_farnesyltransferase_mitochondrial____Perkinsus_olseni
KAF4705058_1_Protoheme_IX_farnesyltransferase_mitochondrial_partial____Perkinsus_olseni
KAF4667957_1_Protoheme_IX_farnesyltransferase_mitochondrial____Perkinsus_olseni
KAF4673573_1_Protoheme_IX_farnesyltransferase_mitochondrial____Perkinsus_olseni
KAF4654962_1_Protoheme_IX_farnesyltransferase_mitochondrial____Perkinsus_chesapeaki
XP_002766194_1_conserved_hypothetical_protein____Perkinsus_marinus_ATCC_50983
XP_002766193_1_conserved_hypothetical_protein____Perkinsus_marinus_ATCC_50983
Nephromyces_occidentalis__1796
Nephromyces_occidentalis__6785
Nephromyces_occidentalis__49704
Nephromyces_occidentalis__32620
Nephromyces_occidentalis__49475
Nephromyces_occidentalis__32259
Nephromyces_occidentalis__32260
Nephromyces_occidentalis__55527
Nephromyces_occidentalis__82557
Nephromyces_occidentalis__58593
Alphamonas_edax__34324
Nephromyces_occidentalis__32619
Rhytidocystis_sp2__22641
Rhytidocystis_sp2__16257
Rhytidocystis_sp2__10146
Colpodella_angusta1__36793
Colpodella_angusta1__3434
Digyalum_oweni1__8477
Digyalum_oweni1__1959
Digyalum_oweni2__9470
Monocystis_agilis__27501
Vitrella_brassicaformis__4969
CEL98276_1_unnamed_protein_product____Vitrella_brassicaformis_CCMP3155
Platyproteum_sp__4066
Colpodella_angusta2__72641
Colpodella_angusta1__58557
Colpodella_angusta2__72638
Colpodella_angusta2__72640
Colpodella_angusta2__72639
Chromera_velia__27397
Piridium__1885
Piridium__3995
Piridium__8518
Piridium__8573
Piridium__9022
Selenidium_pygospionis__10273
XP_008873352_1_protoheme_IX_farnesyltransferase____Aphanomyces_invadans
RHY35322_1_hypothetical_protein_DYB32_000223____Aphanomyces_invadans
RHX97874_1_hypothetical_protein_DYB25_005439____Aphanomyces_astaci
RHY03696_1_hypothetical_protein_DYB36_006369____Aphanomyces_astaci
XP_009829659_1_protoheme_IX_farnesyltransferase_partial____Aphanomyces_astaci
RQM24552_1_hypothetical_protein_B5M09_003781____Aphanomyces_astaci
RHY96007_1_hypothetical_protein_DYB35_000993____Aphanomyces_astaci
KAF0740949_1_hypothetical_protein_Ae201684_003832____Aphanomyces_euteiches
KAF0740950_1_hypothetical_protein_Ae201684_003832____Aphanomyces_euteiches
OQR83121_1_protoheme_IX_farnesyltransferase____Achlya_hypogyna
XP_012199279_1_hypothetical_protein_SPRG_05289____Saprolegnia_parasitica_CBS_223_65
XP_008610636_1_hypothetical_protein_SDRG_06624____Saprolegnia_diclina_VS20
OQS03710_1_protoheme_IX_farnesyltransferase____Thraustotheca_clavata
KAF0689378_1_hypothetical_protein_As57867_019106____Aphanomyces_stellatus
0.5

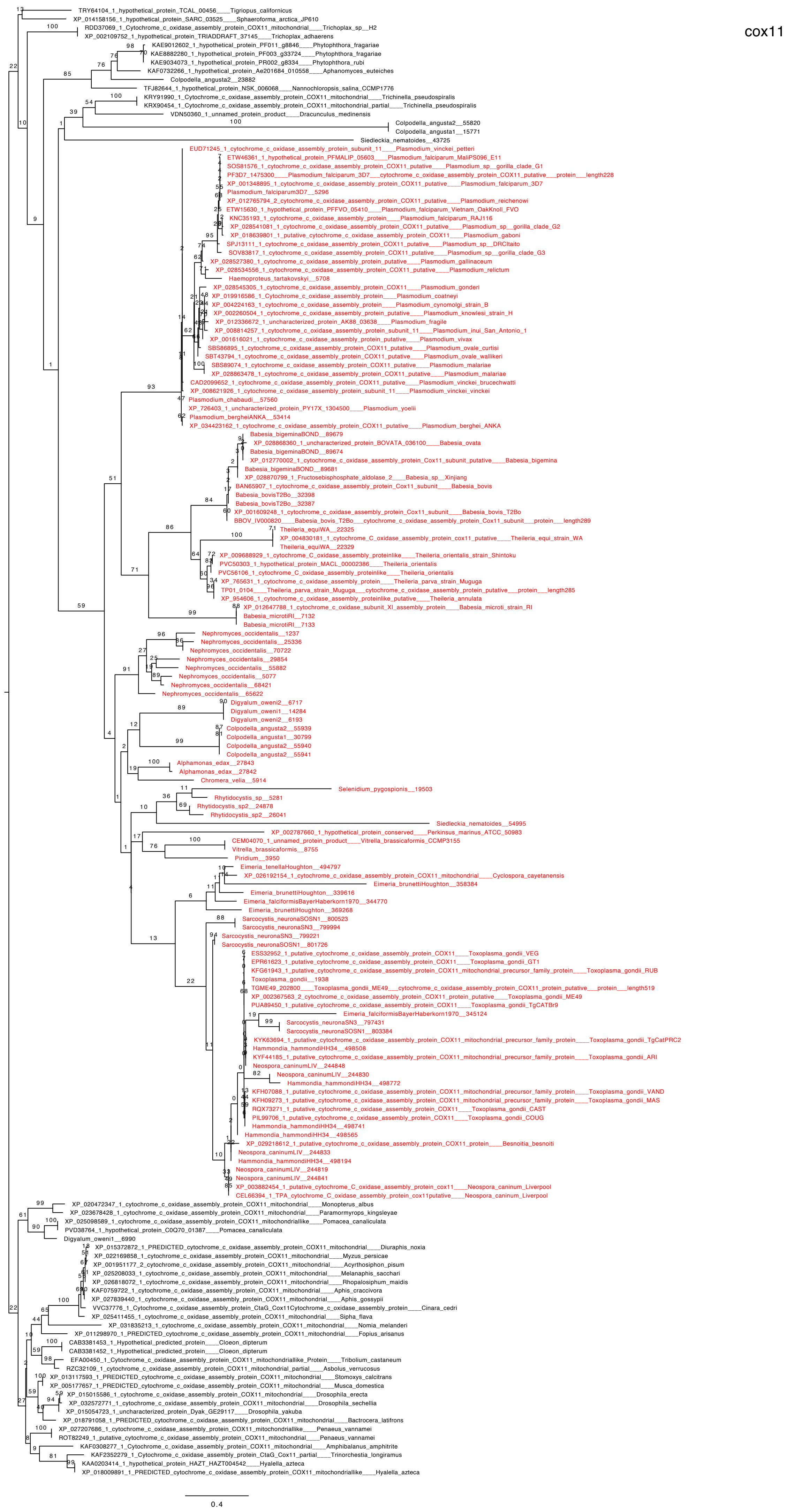

cox11
TRY64104_1_hypothetical_protein_TCAL_00456____Tigriopus_californicus
XP_014158156_1_hypothetical_protein_SARC_03525____Sphaeroforma_arctica_JP610
RDD37069_1_Cytochrome_c_oxidase_assembly_protein_COX11_mitochondrial____Trichoplax_sp__H2
XP_002109752_1_hypothetical_protein_TRIADDRAFT_37145____Trichoplax_adhaerens
KAE9012602_1_hypothetical_protein_PF011_g8846____Phytophthora_fragariae
KAE8882280_1_hypothetical_protein_PF003_g33724____Phytophthora_fragariae
KAE9034073_1_hypothetical_protein_PR002_g8334____Phytophthora_rubi
KAF0732266_1_hypothetical_protein_Ae201684_010558____Aphanomyces_euteiches
Colpodella_angusta2__23882
TFJ82644_1_hypothetical_protein_NSK_006068____Nannochloropsis_salina_CCMP1776
KRY91990_1_Cytochrome_c_oxidase_assembly_protein_COX11_mitochondrial____Trichinella_pseudospiralis
KRX90454_1_Cytochrome_c_oxidase_assembly_protein_COX11_mitochondrial_partial____Trichinella_pseudospiralis
VDN50360_1_unnamed_protein_product____Dracunculus_medinensis
Colpodella_angusta2__55820
Colpodella_angusta1__15771
Siedleckia_nematoides__43725
EUD71245_1_cytochrome_c_oxidase_assembly_protein_subunit_11____Plasmodium_vinckei_petteri
ETW46361_1_hypothetical_protein_PFMALIP_05603____Plasmodium_falciparum_MaliPS096_E11
SOS81576_1_cytochrome_c_oxidase_assembly_protein_COX11_putative____Plasmodium_sp__gorilla_clade_G1
PF3D7_1475300____Plasmodium_falciparum_3D7___cytochrome_c_oxidase_assembly_protein_COX11_putative___protein___length228
XP_001348895_1_cytochrome_c_oxidase_assembly_protein_COX11_putative____Plasmodium_falciparum_3D7
Plasmodium_falciparum3D7__5296
XP_012765794_2_cytochrome_c_oxidase_assembly_protein_COX11_putative____Plasmodium_reichenowi
ETW15630_1_hypothetical_protein_PFFVO_05410____Plasmodium_falciparum_Vietnam_OakKnoll_FVO
KNC35193_1_cytochrome_c_oxidase_assembly_protein____Plasmodium_falciparum_RAJ116
XP_028541081_1_cytochrome_c_oxidase_assembly_protein_COX11_putative____Plasmodium_sp__gorilla_clade_G2
XP_018639801_1_putative_cytochrome_c_oxidase_assembly_protein_COX11____Plasmodium_gaboni
SPJ13111_1_cytochrome_c_oxidase_assembly_protein_COX11_putative____Plasmodium_sp__DRC!taito
SOV83817_1_cytochrome_c_oxidase_assembly_protein_COX11_putative____Plasmodium_sp__gorilla_clade_G3
XP_028527380_1_cytochrome_c_oxidase_assembly_protein_putative____Plasmodium_gallinaceum
XP_028534556_1_cytochrome_c_oxidase_assembly_protein_COX11_putative____Plasmodium_relictum
Haemoproteus_tartakovskyi__5708
XP_028545305_1_cytochrome_c_oxidase_assembly_protein_COX11____Plasmodium_gonderi
XP_019916586_1_Cytochrome_c_oxidase_assembly_protein____Plasmodium_coatneyi
XP_004224163_1_cytochrome_c_oxidase_assembly_protein____Plasmodium_cynomolgi_strain_B
XP_002260504_1_cytochrome_c_oxidase_assembly_protein_putative____Plasmodium_knowlesi_strain_H
XP_012336672_1_uncharacterized_protein_AK88_03638____Plasmodium_fragile
XP_008814257_1_cytochrome_c_oxidase_assembly_protein_subunit_11____Plasmodium_inui_San_Antonio_1
XP_001616021_1_cytochrome_c_oxidase_assembly_protein_putative____Plasmodium_vivax
SBS86895_1_cytochrome_c_oxidase_assembly_protein_COX11_putative____Plasmodium_ovale_curtisi
SBT43794_1_cytochrome_c_oxidase_assembly_protein_COX11_putative____Plasmodium_ovale_wallikeri
SBS89074_1_cytochrome_c_oxidase_assembly_protein_COX11_putative____Plasmodium_malariae
XP_028863478_1_cytochrome_c_oxidase_assembly_protein_COX11_putative____Plasmodium_malariae
CAD2099652_1_cytochrome_c_oxidase_assembly_protein_COX11_putative____Plasmodium_vinckei_brucechwatti
XP_008621926_1_cytochrome_c_oxidase_assembly_protein_subunit_11____Plasmodium_vinckei_vinckei
Plasmodium_chabaudi__57560
XP_726403_1_uncharacterized_protein_PY17X_1304500____Plasmodium_yoelii
Plasmodium_bergheiANKA__53414
XP_034423162_1_cytochrome_c_oxidase_assembly_protein_COX11_putative____Plasmodium_berghei_ANKA
Babesia_bigeminaBOND__89679
XP_028868360_1_uncharacterized_protein_BOVATA_036100____Babesia_ovata
Babesia_bigeminaBOND__89674
XP_012770002_1_cytochrome_c_oxidase_assembly_protein_Cox11_subunit_putative____Babesia_bigemina
Babesia_bigeminaBOND__89681
XP_028870799_1_Fructosebisphosphate_aldolase_2____Babesia_sp__Xinjiang
BAN65907_1_cytochrome_c_oxidase_assembly_protein_Cox11_subunit____Babesia_bovis
Babesia_bovisT2Bo__32398
Babesia_bovisT2Bo__32387
XP_001609248_1_cytochrome_c_oxidase_assembly_protein_Cox11_subunit____Babesia_bovis_T2Bo
BBOV_IV000820____Babesia_bovis_T2Bo___cytochrome_c_oxidase_assembly_protein_Cox11_subunit___protein___length289
Theileria_equiWA__22325
XP_004830181_1_cytochrome_C_oxidase_assembly_protein_cox11_putative____Theileria_equi_strain_WA
Theileria_equiWA__22329
XP_009688929_1_cytochrome_C_oxidase_assembly_proteinlike____Theileria_orientalis_strain_Shintoku
PVC50303_1_hypothetical_protein_MACL_00002386____Theileria_orientalis
PVC56106_1_cytochrome_C_oxidase_assembly_proteinlike____Theileria_orientalis
XP_765631_1_cytochrome_c_oxidase_assembly_protein____Theileria_parva_strain_Muguga
TP01_0104____Theileria_parva_strain_Muguga___cytochrome_c_oxidase_assembly_protein_putative___protein___length285
XP_954606_1_cytochrome_c_oxidase_assembly_proteinlike_putative____Theileria_annulata
XP_012647788_1_cytochrome_c_oxidase_subunit_XI_assembly_protein____Babesia_microti_strain_RI
Babesia_microtiRI__7132
Babesia_microtiRI__7133
Nephromyces_occidentalis__1237
Nephromyces_occidentalis__25336
Nephromyces_occidentalis__70722
Nephromyces_occidentalis__29854
Nephromyces_occidentalis__55882
Nephromyces_occidentalis__5077
Nephromyces_occidentalis__68421
Nephromyces_occidentalis__65622
Digyalum_oweni2__6717
Digyalum_oweni1__14284
Digyalum_oweni2__6193
Colpodella_angusta2__55939
Colpodella_angusta1__30799
Colpodella_angusta2__55940
Colpodella_angusta2__55941
Alphamonas_edax__27843
Alphamonas_edax__27842
Chromera_velia__5914
Selenidium_pygospionis__19503
Rhytidocystis_sp__5281
Rhytidocystis_sp2__24878
Rhytidocystis_sp2__26041
Siedleckia_nematoides__54995
XP_002787660_1_hypothetical_protein_conserved____Perkinsus_marinus_ATCC_50983
CEM04070_1_unnamed_protein_product____Vitrella_brassicaformis_CCMP3155
Vitrella_brassicaformis__8755
Piridium__3950
Eimeria_tenellaHoughton__494797
XP_026192154_1_cytochrome_c_oxidase_assembly_protein_COX11_mitochondrial____Cyclospora_cayetanensis
Eimeria_brunettiHoughton__358384
Eimeria_brunettiHoughton__339616
Eimeria_falciformisBayerHaberkorn1970__344770
Eimeria_brunettiHoughton__369268
Sarcocystis_neuronaSOSN1__800523
Sarcocystis_neuronaSN3__799994
Sarcocystis_neuronaSN3__799221
Sarcocystis_neuronaSOSN1__801726
ESS32952_1_putative_cytochrome_c_oxidase_assembly_protein_COX11____Toxoplasma_gondii_VEG
EPR61623_1_putative_cytochrome_c_oxidase_assembly_protein_COX11____Toxoplasma_gondii_GT1
KFG61943_1_putative_cytochrome_c_oxidase_assembly_protein_COX11_mitochondrial_precursor_family_protein____Toxoplasma_gondii_RUB
Toxoplasma_gondii__1938
TGME49_202800____Toxoplasma_gondii_ME49___cytochrome_c_oxidase_assembly_protein_COX11_protein_putative___protein___length519
XP_002367563_2_cytochrome_c_oxidase_assembly_protein_COX11_protein_putative____Toxoplasma_gondii_ME49
PUA89450_1_putative_cytochrome_c_oxidase_assembly_protein_COX11____Toxoplasma_gondii_TgCATBr9
Eimeria_falciformisBayerHaberkorn1970__345124
Sarcocystis_neuronaSN3__797431
Sarcocystis_neuronaSOSN1__803384
KYK63694_1_putative_cytochrome_c_oxidase_assembly_protein_COX11_mitochondrial_precursor_family_protein____Toxoplasma_gondii_TgCatPRC2
Hammondia_hammondiHH34__498508
KYF44185_1_putative_cytochrome_c_oxidase_assembly_protein_COX11_mitochondrial_precursor_family_protein____Toxoplasma_gondii_ARI
Neospora_caninumLIV__244848
Neospora_caninumLIV__244830
Hammondia_hammondiHH34__498772
KFH07088_1_putative_cytochrome_c_oxidase_assembly_protein_COX11_mitochondrial_precursor_family_protein____Toxoplasma_gondii_VAND
KFH09273_1_putative_cytochrome_c_oxidase_assembly_protein_COX11_mitochondrial_precursor_family_protein____Toxoplasma_gondii_MAS
RQX73271_1_putative_cytochrome_c_oxidase_assembly_protein_COX11____Toxoplasma_gondii_CAST
PIL99706_1_putative_cytochrome_c_oxidase_assembly_protein_COX11____Toxoplasma_gondii_COUG
Hammondia_hammondiHH34__498741
Hammondia_hammondiHH34__498565
XP_029218612_1_putative_cytochrome_c_oxidase_assembly_protein_COX11_protein____Besnoitia_besnoiti
Neospora_caninumLIV__244833
Hammondia_hammondiHH34__498194
Neospora_caninumLIV__244819
Neospora_caninumLIV__244841
XP_003882454_1_putative_cytochrome_C_oxidase_assembly_protein_cox11____Neospora_caninum_Liverpool
CEL66394_1_TPA_cytochrome_C_oxidase_assembly_protein_cox11putative____Neospora_caninum_Liverpool
XP_020472347_1_cytochrome_c_oxidase_assembly_protein_COX11_mitochondrial____Monopterus_albus
XP_023678428_1_cytochrome_c_oxidase_assembly_protein_COX11_mitochondrial____Paramormyrops_kingsleyae
XP_025098589_1_cytochrome_c_oxidase_assembly_protein_COX11_mitochondriallike____Pomacea_canaliculata
PVD38764_1_hypothetical_protein_C0Q70_01387____Pomacea_canaliculata
Digyalum_oweni1__6990
XP_015372872_1_PREDICTED_cytochrome_c_oxidase_assembly_protein_COX11_mitochondrial____Diuraphis_noxia
XP_022169858_1_cytochrome_c_oxidase_assembly_protein_COX11_mitochondrial____Myzus_persicae
XP_001951177_2_cytochrome_c_oxidase_assembly_protein_COX11_mitochondrial____Acyrthosiphon_pisum
XP_025208033_1_cytochrome_c_oxidase_assembly_protein_COX11_mitochondrial____Melanaphis_sacchari
XP_026818072_1_cytochrome_c_oxidase_assembly_protein_COX11_mitochondrial____Rhopalosiphum_maidis
KAF0759722_1_cytochrome_c_oxidase_assembly_protein_COX11_mitochondrial____Aphis_craccivora
XP_027839440_1_cytochrome_c_oxidase_assembly_protein_COX11_mitochondrial____Aphis_gossypii
VVC37776_1_Cytochrome_c_oxidase_assembly_protein_CtaG_Cox11Cytochrome_c_oxidase_assembly_protein____Cinara_cedri
XP_025411455_1_cytochrome_c_oxidase_assembly_protein_COX11_mitochondrial____Sipha_flava
XP_031835213_1_cytochrome_c_oxidase_assembly_protein_COX11_mitochondrial____Nomia_melanderi
XP_011298970_1_PREDICTED_cytochrome_c_oxidase_assembly_protein_COX11_mitochondrial____Fopius_arisanus
CAB3381453_1_Hypothetical_predicted_protein____Cloeon_dipterum
CAB3381452_1_Hypothetical_predicted_protein____Cloeon_dipterum
EFA00450_1_Cytochrome_c_oxidase_assembly_protein_COX11_mitochondriallike_Protein____Tribolium_castaneum
RZC32109_1_cytochrome_c_oxidase_assembly_protein_COX11_mitochondrial_partial____Asbolus_verrucosus
XP_013117593_1_PREDICTED_cytochrome_c_oxidase_assembly_protein_COX11_mitochondrial____Stomoxys_calcitrans
XP_005177657_1_PREDICTED_cytochrome_c_oxidase_assembly_protein_COX11_mitochondrial____Musca_domestica
XP_015015586_1_cytochrome_c_oxidase_assembly_protein_COX11_mitochondrial____Drosophila_erecta
XP_032572771_1_cytochrome_c_oxidase_assembly_protein_COX11_mitochondrial____Drosophila_sechellia
XP_015054723_1_uncharacterized_protein_Dyak_GE29117____Drosophila_yakuba
XP_018791058_1_PREDICTED_cytochrome_c_oxidase_assembly_protein_COX11_mitochondrial____Bactrocera_latifrons
XP_027207686_1_cytochrome_c_oxidase_assembly_protein_COX11_mitochondriallike____Penaeus_vannamei
ROT82249_1_putative_cytochrome_c_oxidase_assembly_protein_COX11_mitochondrial____Penaeus_vannamei
KAF0308277_1_Cytochrome_c_oxidase_assembly_protein_COX11_mitochondrial____Amphibalanus_amphitrite
KAF2352279_1_Cytochrome_c_oxidase_assembly_protein_CtaG_Cox11_partial____Trinorchestia_longiramus
KAA0203414_1_hypothetical_protein_HAZT_HAZT004542____Hyalella_azteca
XP_018009891_1_PREDICTED_cytochrome_c_oxidase_assembly_protein_COX11_mitochondriallike____Hyalella_azteca
0.4

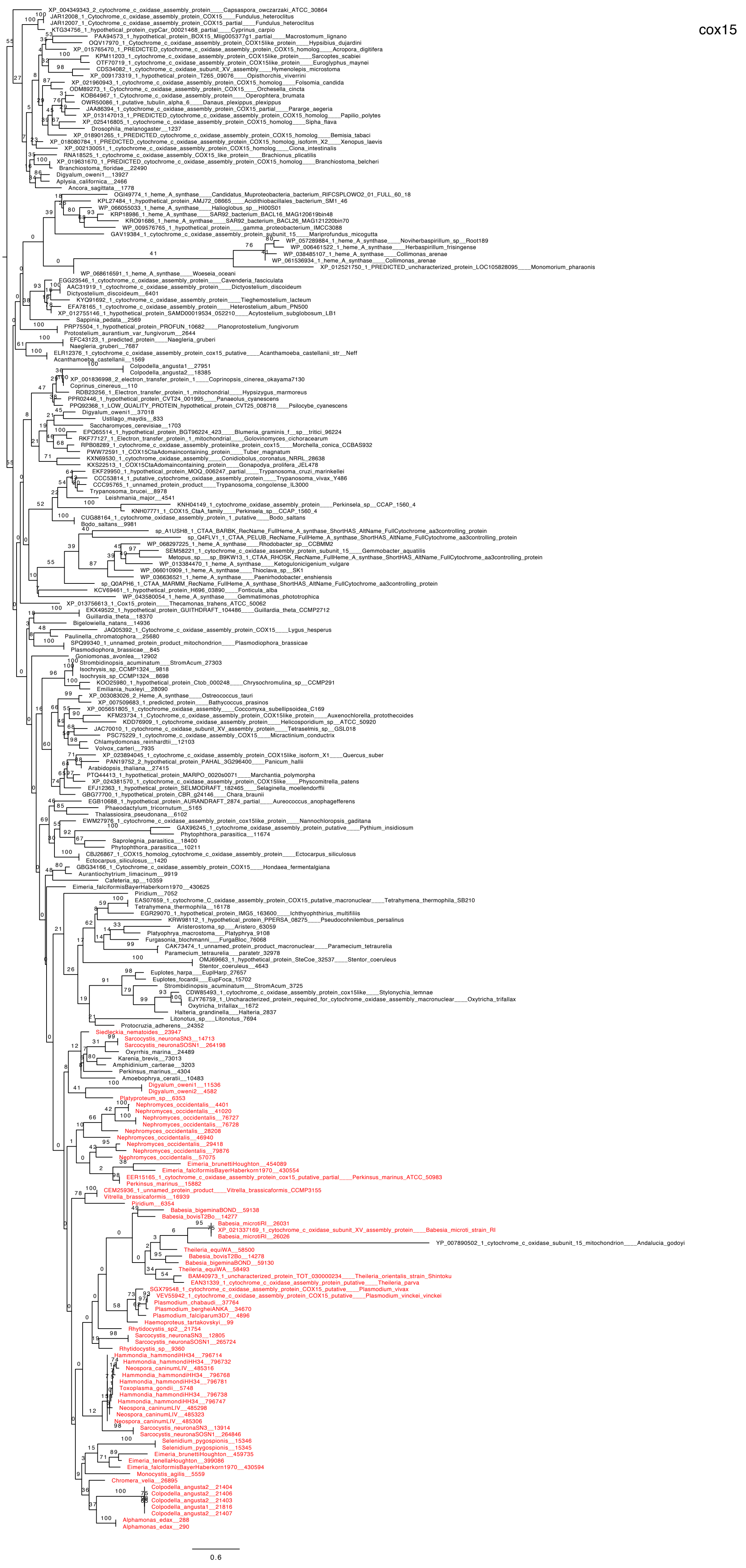
cox15
0.6

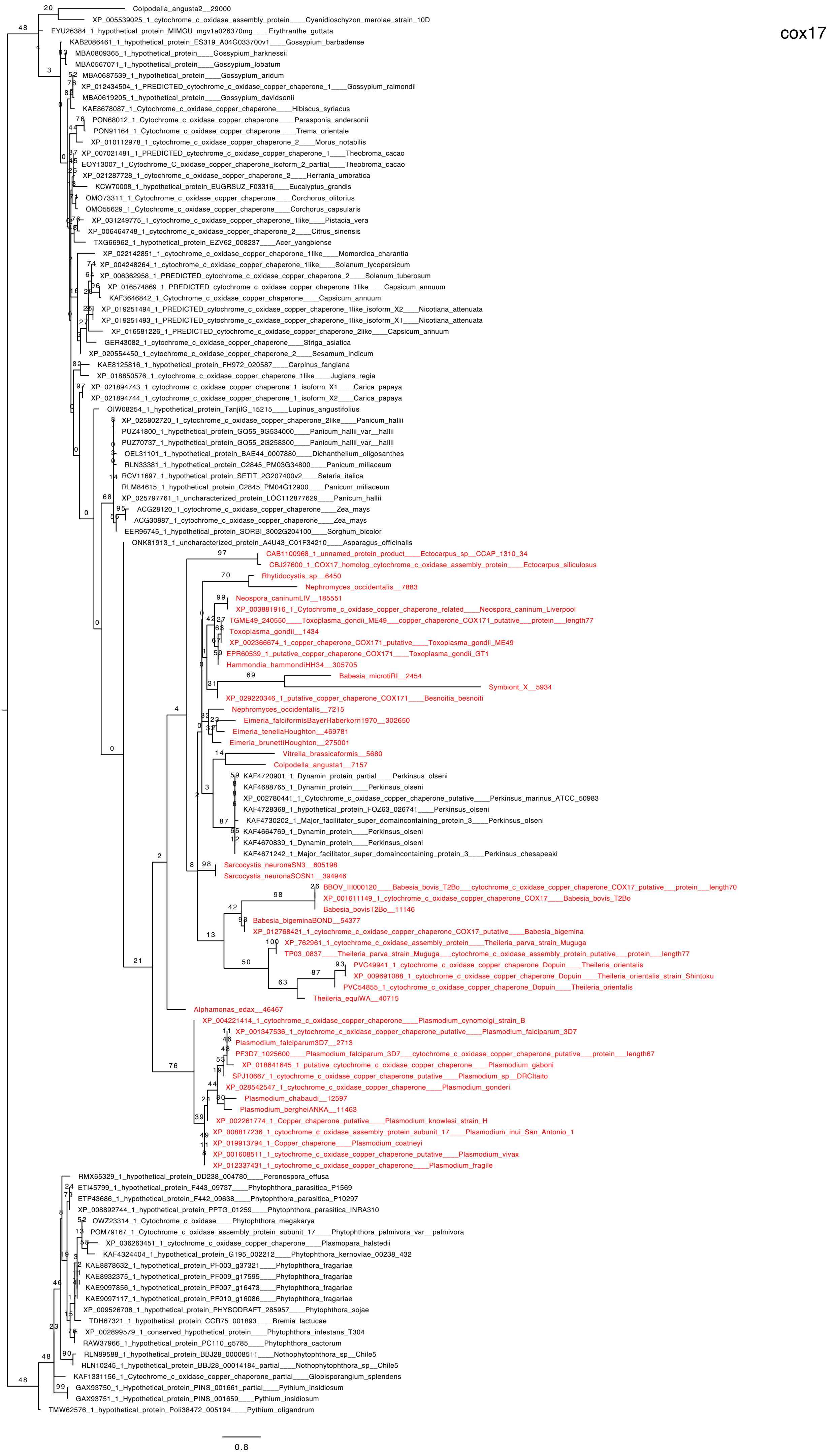
cox17
Colpodella_angusta2__29000
XP_005539025_1_cytochrome_c_oxidase_assembly_protein____Cyanidioschyzon_merolae_strain_10D
EYU26384_1_hypothetical_protein_MIMGU_mgv1a026370mg____Erythranthe_guttata
KAB2086461_1_hypothetical_protein_ES319_A04G033700v1____Gossypium_barbadense
MBA0809365_1_hypothetical_protein____Gossypium_harknessii
MBA0567071_1_hypothetical_protein____Gossypium_lobatum
MBA0687539_1_hypothetical_protein____Gossypium_aridum
XP_012434504_1_PREDICTED_cytochrome_c_oxidase_copper_chaperone_1____Gossypium_raimondii
MBA0619205_1_hypothetical_protein____Gossypium_davidsonii
KAE8678087_1_Cytochrome_c_oxidase_copper_chaperone____Hibiscus_syriacus
PON68012_1_Cytochrome_c_oxidase_copper_chaperone____Parasponia_andersonii
PON91164_1_Cytochrome_c_oxidase_copper_chaperone____Trema_orientale
XP_010112978_1_cytochrome_c_oxidase_copper_chaperone_2____Morus_notabilis
XP_007021481_1_PREDICTED_cytochrome_c_oxidase_copper_chaperone_1____Theobroma_cacao
EOY13007_1_Cytochrome_C_oxidase_copper_chaperone_isoform_2_partial____Theobroma_cacao
XP_021287728_1_cytochrome_c_oxidase_copper_chaperone_2____Herrania_umbratica
KCW70008_1_hypothetical_protein_EUGRSUZ_F03316____Eucalyptus_grandis
OMO73311_1_Cytochrome_c_oxidase_copper_chaperone____Corchorus_olitorius
OMO55629_1_Cytochrome_c_oxidase_copper_chaperone____Corchorus_capsularis
XP_031249775_1_cytochrome_c_oxidase_copper_chaperone_1like____Pistacia_vera
XP_006464748_1_cytochrome_c_oxidase_copper_chaperone_2____Citrus_sinensis
TXG66962_1_hypothetical_protein_EZV62_008237____Acer_yangbiense
XP_022142851_1_cytochrome_c_oxidase_copper_chaperone_1like____Momordica_charantia
XP_004248264_1_cytochrome_c_oxidase_copper_chaperone_1like____Solanum_lycopersicum
XP_006362958_1_PREDICTED_cytochrome_c_oxidase_copper_chaperone_2____Solanum_tuberosum
XP_016574869_1_PREDICTED_cytochrome_c_oxidase_copper_chaperone_1like____Capsicum_annuum
KAF3646842_1_Cytochrome_c_oxidase_copper_chaperone____Capsicum_annuum
XP_019251494_1_PREDICTED_cytochrome_c_oxidase_copper_chaperone_1like_isoform_X2____Nicotiana_attenuata
XP_019251493_1_PREDICTED_cytochrome_c_oxidase_copper_chaperone_1like_isoform_X1____Nicotiana_attenuata
XP_016581226_1_PREDICTED_cytochrome_c_oxidase_copper_chaperone_2like____Capsicum_annuum
GER43082_1_cytochrome_c_oxidase_copper_chaperone____Striga_asiatica
XP_020554450_1_cytochrome_c_oxidase_copper_chaperone_2____Sesamum_indicum
KAE8125816_1_hypothetical_protein_FH972_020587____Carpinus_fangiana
XP_018850576_1_cytochrome_c_oxidase_copper_chaperone_1like____Juglans_regia
XP_021894743_1_cytochrome_c_oxidase_copper_chaperone_1_isoform_X1____Carica_papaya
XP_021894744_1_cytochrome_c_oxidase_copper_chaperone_1_isoform_X2____Carica_papaya
OIW08254_1_hypothetical_protein_TanjilG_15215____Lupinus_angustifolius
XP_025802720_1_cytochrome_c_oxidase_copper_chaperone_2like____Panicum_hallii
PUZ41800_1_hypothetical_protein_GQ55_9G534000____Panicum_hallii_var__hallii
PUZ70737_1_hypothetical_protein_GQ55_2G258300____Panicum_hallii_var__hallii
OEL31101_1_hypothetical_protein_BAE44_0007880____Dichanthelium_oligosanthes
RLN33381_1_hypothetical_protein_C2845_PM03G34800____Panicum_miliaceum
RCV11697_1_hypothetical_protein_SETIT_2G207400v2____Setaria_italica
RLM84615_1_hypothetical_protein_C2845_PM04G12900____Panicum_miliaceum
XP_025797761_1_uncharacterized_protein_LOC112877629____Panicum_hallii
ACG28120_1_cytochrome_c_oxidase_copper_chaperone____Zea_mays
ACG30887_1_cytochrome_c_oxidase_copper_chaperone____Zea_mays
EER96745_1_hypothetical_protein_SORBI_3002G204100____Sorghum_bicolor
ONK81913_1_uncharacterized_protein_A4U43_C01F34210____Asparagus_officinalis
CAB1100968_1_unnamed_protein_product____Ectocarpus_sp__CCAP_1310_34
CBJ27600_1_COX17_homolog_cytochrome_c_oxidase_assembly_protein____Ectocarpus_siliculosus
Rhytidocystis_sp__6450
Nephromyces_occidentalis__7883
Neospora_caninumLIV__185551
XP_003881916_1_Cytochrome_c_oxidase_copper_chaperone_related____Neospora_caninum_Liverpool
TGME49_240550____Toxoplasma_gondii_ME49____copper_chaperone_COX171_putative___protein___length77
Toxoplasma_gondii__1434
XP_002366674_1_copper_chaperone_COX171_putative____Toxoplasma_gondii_ME49
EPR60539_1_putative_copper_chaperone_COX171____Toxoplasma_gondii_GT1
Hammondia_hammondiHH34__305705
Babesia_microtiRI__2454
Symbiont_X__5934
XP_029220346_1_putative_copper_chaperone_COX171____Besnoitia_besnoiti
Nephromyces_occidentalis__7215
Eimeria_falciformisBayerHaberkorn1970__302650
Eimeria_tenellaHoughton__469781
Eimeria_brunettiHoughton__275001
Vitrella_brassicaformis__5680
Colpodella_angusta1__7157
KAF4720901_1_Dynamin_protein_partial____Perkinsus_olseni
KAF4688765_1_Dynamin_protein____Perkinsus_olseni
XP_002780441_1_Cytochrome_c_oxidase_copper_chaperone_putative____Perkinsus_marinus_ATCC_50983
KAF4728368_1_hypothetical_protein_FOZ63_026741____Perkinsus_olseni
KAF4730202_1_Major_facilitator_super_domaincontaining_protein_3____Perkinsus_olseni
KAF4664769_1_Dynamin_protein____Perkinsus_olseni
KAF4670839_1_Dynamin_protein____Perkinsus_olseni
KAF4671242_1_Major_facilitator_super_domaincontaining_protein_3____Perkinsus_chesapeaki
Sarcocystis_neuronaSN3__605198
Sarcocystis_neuronaSOSN1__394946
BBOV_III000120____Babesia_bovis_T2Bo____cytochrome_c_oxidase_copper_chaperone_COX17_putative___protein___length70
XP_001611149_1_cytochrome_c_oxidase_copper_chaperone_COX17____Babesia_bovis_T2Bo
Babesia_bovisT2Bo__11146
Babesia_bigeminaBOND__54377
XP_012768421_1_cytochrome_c_oxidase_copper_chaperone_COX17_putative____Babesia_bigemina
XP_762961_1_cytochrome_c_oxidase_assembly_protein____Theileria_parva_strain_Muguga
TP03_0837____Theileria_parva_strain_Muguga____cytochrome_c_oxidase_assembly_protein_putative___protein___length77
PVC49941_1_cytochrome_c_oxidase_copper_chaperone_Dopuin____Theileria_orientalis
XP_009691088_1_cytochrome_c_oxidase_copper_chaperone_Dopuin____Theileria_orientalis_strain_Shintoku
PVC54855_1_cytochrome_c_oxidase_copper_chaperone_Dopuin____Theileria_orientalis
Theileria_equiWA__40715
Alphamonas_edax__46467
XP_004221414_1_cytochrome_c_oxidase_copper_chaperone____Plasmodium_cynomolgi_strain_B
XP_001347536_1_cytochrome_c_oxidase_copper_chaperone_putative____Plasmodium_falciparum_3D7
Plasmodium_falciparum3D7__2713
PF3D7_1025600____Plasmodium_falciparum_3D7____cytochrome_c_oxidase_copper_chaperone_putative___protein___length67
XP_018641645_1_putative_cytochrome_c_oxidase_copper_chaperone____Plasmodium_gaboni
SPJ10667_1_cytochrome_c_oxidase_copper_chaperone_putative____Plasmodium_sp__DRCItaito
XP_028542547_1_cytochrome_c_oxidase_copper_chaperone____Plasmodium_gonderi
Plasmodium_chabaudi__12597
Plasmodium_bergheiANKA__11463
XP_002261774_1_Copper_chaperone_putative____Plasmodium_knowlesi_strain_H
XP_008817236_1_cytochrome_c_oxidase_assembly_protein_subunit_17____Plasmodium_inui_San_Antonio_1
XP_019913794_1_Copper_chaperone____Plasmodium_coatneyi
XP_001608511_1_cytochrome_c_oxidase_copper_chaperone_putative____Plasmodium_vivax
XP_012337431_1_cytochrome_c_oxidase_copper_chaperone____Plasmodium_fragile
RMX65329_1_hypothetical_protein_DD238_004780____Peronospora_effusa
ETI45799_1_hypothetical_protein_F443_09737____Phytophthora_parasitica_P1569
ETP43686_1_hypothetical_protein_F442_09638____Phytophthora_parasitica_P10297
XP_008892744_1_hypothetical_protein_PPTG_01259____Phytophthora_parasitica_INRA310
OWZ23314_1_Cytochrome_c_oxidase____Phytophthora_megakarya
POM79167_1_Cytochrome_c_oxidase_assembly_protein_subunit_17____Phytophthora_palmivora_var__palmivora
XP_036263451_1_cytochrome_c_oxidase_copper_chaperone____Plasmopara_halstedii
KAF4324404_1_hypothetical_protein_G195_002212____Phytophthora_kernoviae_00238_432
KAE8878632_1_hypothetical_protein_PF003_g37321____Phytophthora_fragariae
KAE8932375_1_hypothetical_protein_PF009_g17595____Phytophthora_fragariae
KAE9097856_1_hypothetical_protein_PF007_g16473____Phytophthora_fragariae
KAE9097117_1_hypothetical_protein_PF010_g16086____Phytophthora_fragariae
XP_009526708_1_hypothetical_protein_PHYSODRAFT_285957____Phytophthora_sojae
TDH67321_1_hypothetical_protein_CCR75_001893____Bremia_lactucae
XP_002899579_1_conserved_hypothetical_protein____Phytophthora_infestans_T304
RAW37966_1_hypothetical_protein_PC110_g5785____Phytophthora_cactorum
RLN89588_1_hypothetical_protein_BBJ28_00008511____Nothophytophthora_sp__Chile5
RLN10245_1_hypothetical_protein_BBJ28_00014184_partial____Nothophytophthora_sp__Chile5
KAF1331156_1_Cytochrome_c_oxidase_copper_chaperone_partial____Globisporangium_splendens
GAX93750_1_Hypothetical_protein_PINS_001661_partial____Pythium_insidiosum
GAX93751_1_Hypothetical_protein_PINS_001659____Pythium_insidiosum
TMW62576_1_hypothetical_protein_Poli38472_005194____Pythium_oligandrum
0.8

cox19

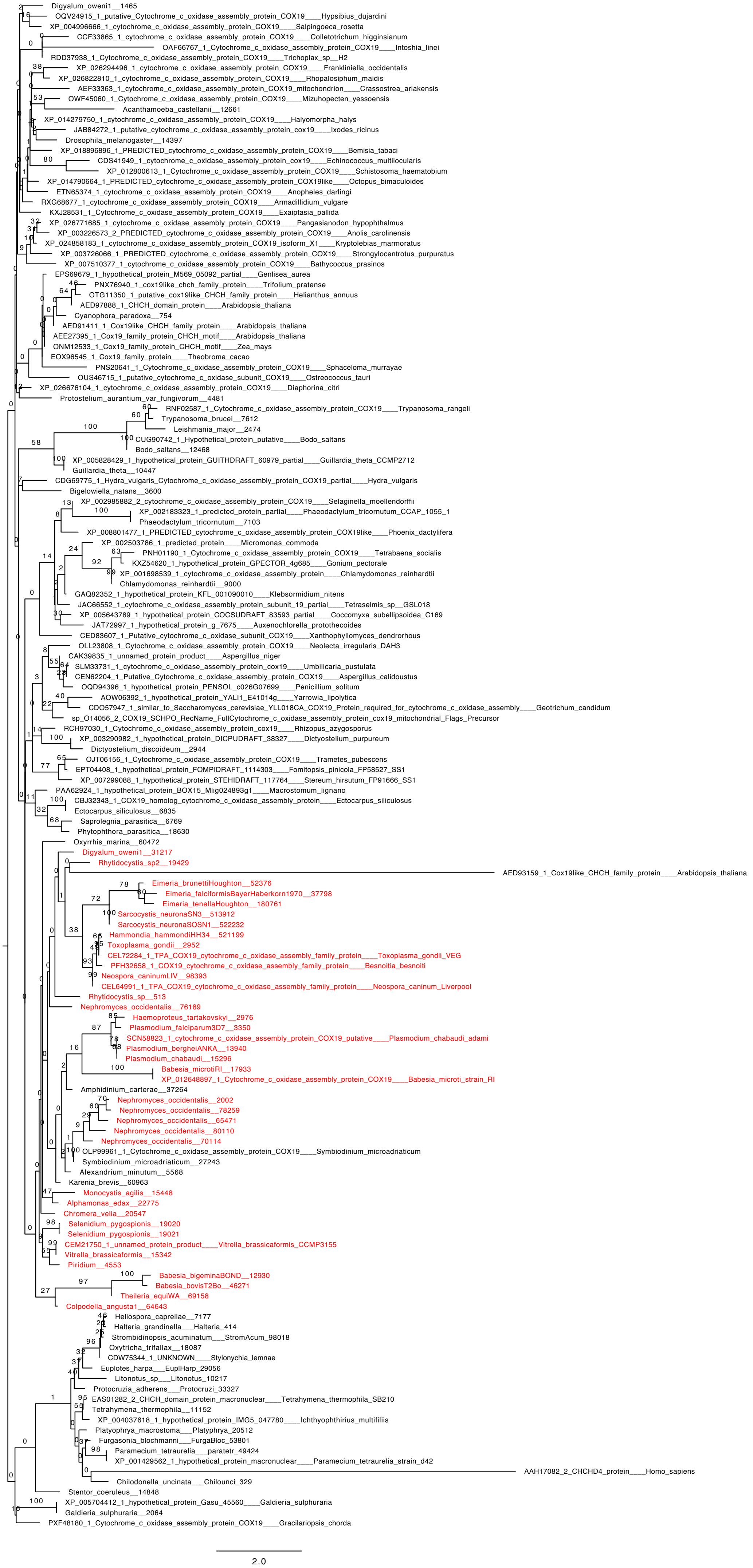

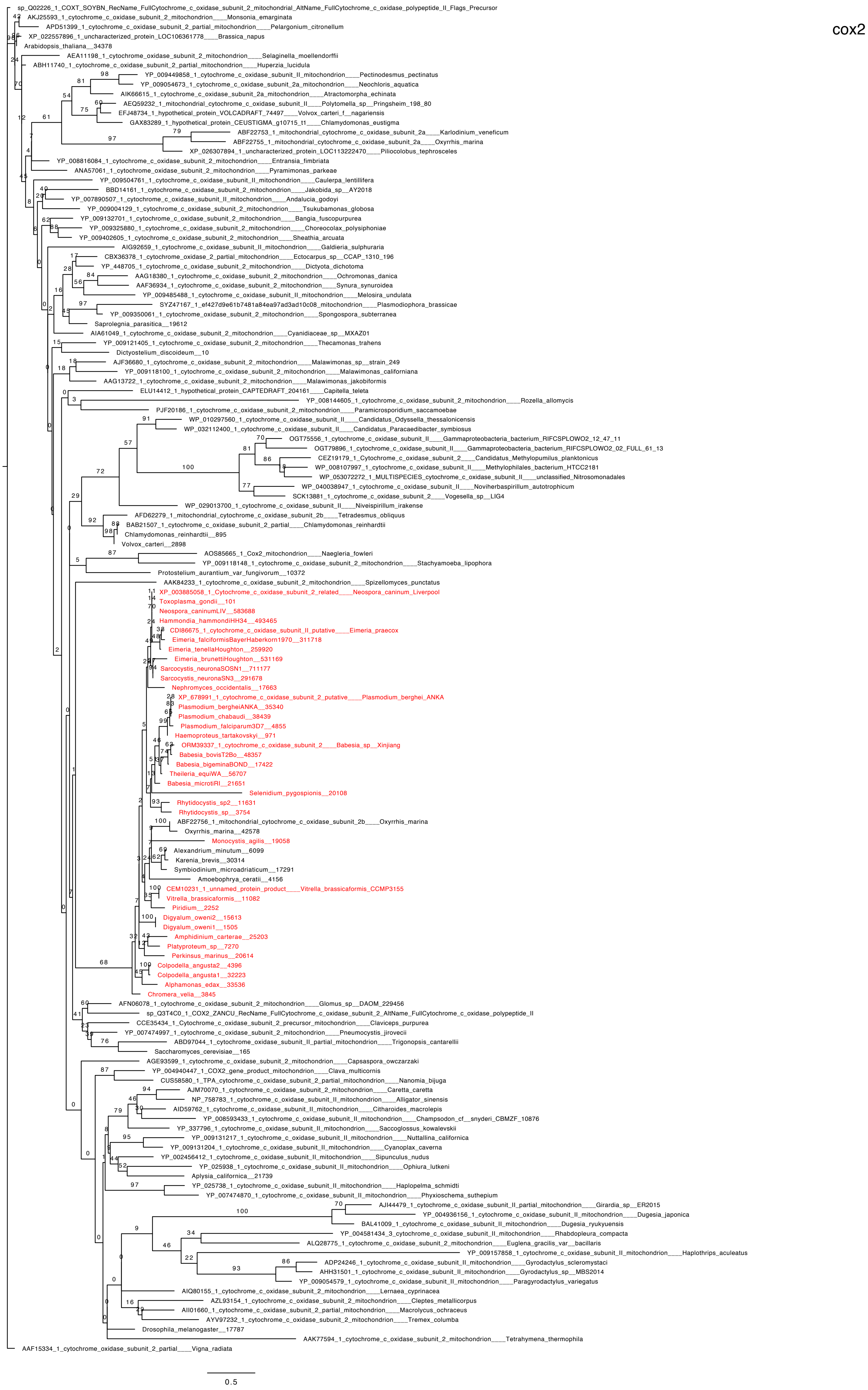
cox2
sp_Q02226_1_COXT_SOYBN_RecName_FullCytochrome_c_oxidase_subunit_2_mitochondrial_AltName_FullCytochrome_c_oxidase_polypeptide_II_Flags_Precursor
AKJ25593_1_cytochrome_c_oxidase_subunit_2_mitochondrion____Monsonia_emarginata
APD51399_1_cytochrome_c_oxidase_subunit_2_partial_mitochondrion____Pelargonium_citronellum
XP_022557896_1_uncharacterized_protein_LOC106361778____Brassica_napus
Arabidopsis_thaliana__34378
AEA11198_1_cytochrome_oxidase_subunit_2_mitochondrion____Selaginella_moellendorffii
ABH11740_1_cytochrome_c_oxidase_subunit_2_partial_mitochondrion____Huperzia_lucidula
YP_009449858_1_cytochrome_c_oxidase_subunit_II_mitochondrion____Pectinodesmus_pectinatus
YP_009054673_1_cytochrome_c_oxidase_subunit_2a_mitochondrion____Neochloris_aquatica
AIK66615_1_cytochrome_c_oxidase_subunit_2a_mitochondrion____Atractomorpha_echinata
AEQ59232_1_mitochondrial_cytochrome_c_oxidase_subunit_II____Polytomella_sp__Pringsheim_198_80
EFJ48734_1_hypothetical_protein_VOLCADRAFT_74497____Volvox_carteri_f__nagariensis
GAX83289_1_hypothetical_protein_CEUSTIGMA_g10715_t1____Chlamydomonas_eustigma
ABF22753_1_mitochondrial_cytochrome_c_oxidase_subunit_2a____Karlodinium_veneficum
ABF22755_1_mitochondrial_cytochrome_c_oxidase_subunit_2a____Oxyrrhis_marina
XP_026307894_1_uncharacterized_protein_LOC113222470____Piliocolobus_tephrosceles
YP_008816084_1_cytochrome_c_oxidase_subunit_2_mitochondrion____Entransia_fimbriata
ANA57061_1_cytochrome_oxidase_subunit_2_mitochondrion____Pyramimonas_parkeae
YP_009504761_1_cytochrome_c_oxidase_subunit_II_mitochondrion____Caulerpa_lentillifera
BBD14161_1_cytochrome_c_oxidase_subunit_2_mitochondrion____Jakobida_sp__AY2018
YP_007890507_1_cytochrome_c_oxidase_subunit_II_mitochondrion____Andalucia_godoyi
YP_009004129_1_cytochrome_c_oxidase_subunit_2_mitochondrion____Tsukubamonas_globosa
YP_009132701_1_cytochrome_c_oxidase_subunit_2_mitochondrion____Bangia_fuscopurpurea
YP_009325880_1_cytochrome_c_oxidase_subunit_2_mitochondrion____Choreocolax_polysiphoniae
YP_009402605_1_cytochrome_c_oxidase_subunit_2_mitochondrion____Sheathia_arcuata
AIG92659_1_cytochrome_c_oxidase_subunit_II_mitochondrion____Galdieria_sulphuraria
CBX36378_1_cytochrome_oxidase_2_partial_mitochondrion____Ectocarpus_sp__CCAP_1310_196
YP_448705_1_cytochrome_c_oxidase_subunit_2_mitochondrion____Dictyota_dichotoma
AAG18380_1_cytochrome_c_oxidase_subunit_2_mitochondrion____Ochromonas_danica
AAF36934_1_cytochrome_c_oxidase_subunit_2_mitochondrion____Synura_synuroidea
YP_009485488_1_cytochrome_c_oxidase_subunit_II_mitochondrion____Melosira_undulata
SYZ47167_1_ef427d9e61b7481a84ea97ad3ad10c08_mitochondrion____Plasmodiophora_brassicae
YP_009350061_1_cytochrome_oxidase_subunit_2_mitochondrion____Spongospora_subterranea
Saprolegnia_parasitica__19612
AIA61049_1_cytochrome_c_oxidase_subunit_2_mitochondrion____Cyanidiaceae_sp__MXAZ01
YP_009121405_1_cytochrome_c_oxidase_subunit_2_mitochondrion____Thecamonas_trahens
Dictyostelium_discoideum__10
AJF36680_1_cytochrome_c_oxidase_subunit_2_mitochondrion____Malawimonas_sp__strain_249
YP_009118100_1_cytochrome_c_oxidase_subunit_2_mitochondrion____Malawimonas_californiana
AAG13722_1_cytochrome_c_oxidase_subunit_2_mitochondrion____Malawimonas_jakobiformis
ELU14412_1_hypothetical_protein_CAPTEDRAFT_204161____Capitella_teleta
YP_008144605_1_cytochrome_c_oxidase_subunit_2_mitochondrion____Rozella_allomycis
PJF20186_1_cytochrome_c_oxidase_subunit_2_mitochondrion____Paramicrosporidium_saccamoebae
WP_010297560_1_cytochrome_c_oxidase_subunit_II____Candidatus_Odyssella_thessalonicensis
WP_032112400_1_cytochrome_c_oxidase_subunit_II____Candidatus_Paracaedibacter_symbiosus
OGT75556_1_cytochrome_c_oxidase_subunit_II____Gammaproteobacteria_bacterium_RIFCSPLOWO2_12_47_11
OGT79896_1_cytochrome_c_oxidase_subunit_II____Gammaproteobacteria_bacterium_RIFCSPLOWO2_02_FULL_61_13
CEZ19179_1_Cytochrome_c_oxidase_subunit_2____Candidatus_Methylopumilus_planktonicus
WP_008107997_1_cytochrome_c_oxidase_subunit_II____Methylophilales_bacterium_HTCC2181
WP_053072272_1_MULTISPECIES_cytochrome_c_oxidase_subunit_II____unclassified_Nitrosomonadales
WP_040038947_1_cytochrome_c_oxidase_subunit_II____Noviherbaspirillum_autotrophicum
SCK13881_1_cytochrome_c_oxidase_subunit_2____Vogesella_sp__LIG4
WP_029013700_1_cytochrome_c_oxidase_subunit_II____Niveispirillum_irakense
AFD62279_1_mitochondrial_cytochrome_c_oxidase_subunit_2b____Tetradesmus_obliquus
BAB21507_1_cytochrome_c_oxidase_subunit_2_partial____Chlamydomonas_reinhardtii
Chlamydomonas_reinhardtii__895
Volvox_carteri__2898
AOS85665_1_Cox2_mitochondrion____Naegleria_fowleri
YP_009118148_1_cytochrome_c_oxidase_subunit_2_mitochondrion____Stachyamoeba_lipophora
Protostelium_aurantium_var_fungivorum__10372
AAK84233_1_cytochrome_c_oxidase_subunit_2_mitochondrion____Spizellomyces_punctatus
XP_003885058_1_Cytochrome_c_oxidase_subunit_2_related____Neospora_caninum_Liverpool
Toxoplasma_gondii__101
Neospora_caninumLIV__583688
Hammondia_hammondiHH34__493465
CDI86675_1_cytochrome_c_oxidase_subunit_II_putative____Eimeria_praecox
Eimeria_falciformisBayerHaberkorn1970__311718
Eimeria_tenellaHoughton__259920
Eimeria_brunettiHoughton__531169
Sarcocystis_neuronaSOSN1__711177
Sarcocystis_neuronaSN3__291678
Nephromyces_occidentalis__17663
XP_678991_1_cytochrome_c_oxidase_subunit_2_putative____Plasmodium_berghei_ANKA
Plasmodium_bergheiANKA__35340
Plasmodium_chabaudi__38439
Plasmodium_falciparum3D7__4855
Haemoproteus_tartakovskyi__971
ORM39337_1_cytochrome_c_oxidase_subunit_2____Babesia_sp__Xinjiang
Babesia_bovisT2Bo__48357
Babesia_bigeminaBOND__17422
Theileria_equiWA__56707
Babesia_microtiRI__21651
Selenidium_pygospionis__20108
Rhytidocystis_sp2__11631
Rhytidocystis_sp__3754
ABF22756_1_mitochondrial_cytochrome_c_oxidase_subunit_2b____Oxyrrhis_marina
Oxyrrhis_marina__42578
Monocystis_agilis__19058
Alexandrium_minutum__6099
Karenia_brevis__30314
Symbiodinium_microadriaticum__17291
Amoebophrya_ceratii__4156
CEM10231_1_unnamed_protein_product____Vitrella_brassicaformis_CCMP3155
Vitrella_brassicaformis__11082
Piridium__2252
Digyalum_oweni2__15613
Digyalum_oweni1__1505
Amphidinium_carterae__25203
Platyproteum_sp__7270
Perkinsus_marinus__20614
Colpodella_angusta2__4396
Colpodella_angusta1__32223
Alphamonas_edax__33536
Chromera_velia__3845
AFN06078_1_cytochrome_c_oxidase_subunit_2_mitochondrion____Glomus_sp__DAOM_229456
sp_Q3T4C0_1_COX2_ZANCU_RecName_FullCytochrome_c_oxidase_subunit_2_AltName_FullCytochrome_c_oxidase_polypeptide_II
CCE35434_1_Cytochrome_c_oxidase_subunit_2_precursor_mitochondrion____Claviceps_purpurea
YP_007474997_1_cytochrome_c_oxidase_subunit_2_mitochondrion____Pneumocystis_jirovecii
ABD97044_1_cytochrome_oxidase_subunit_II_partial_mitochondrion____Trigonopsis_cantarellii
Saccharomyces_cerevisiae__165
AGE93599_1_cytochrome_c_oxidase_subunit_2_mitochondrion____Capsaspora_owczarzaki
YP_004940447_1_COX2_gene_product_mitochondrion____Clava_multicornis
CUS58580_1_TPA_cytochrome_c_oxidase_subunit_2_partial_mitochondrion____Nanomia_bijuga
AJM70070_1_cytochrome_c_oxidase_subunit_2_mitochondrion____Caretta_caretta
NP_758783_1_cytochrome_c_oxidase_subunit_II_mitochondrion____Alligator_sinensis
AID59762_1_cytochrome_c_oxidase_subunit_II_mitochondrion____Citharoides_macrolepis
YP_008593433_1_cytochrome_c_oxidase_subunit_II_mitochondrion____Champsodon_cf__snyderi_CBMZF_10876
YP_337796_1_cytochrome_c_oxidase_subunit_II_mitochondrion____Saccoglossus_kowalevskii
YP_009131217_1_cytochrome_c_oxidase_subunit_II_mitochondrion____Nuttallina_californica
YP_009131204_1_cytochrome_c_oxidase_subunit_II_mitochondrion____Cyanoplax_caverna
YP_002456412_1_cytochrome_c_oxidase_subunit_II_mitochondrion____Sipunculus_nudus
YP_025938_1_cytochrome_c_oxidase_subunit_II_mitochondrion____Ophiura_lutkeni
Aplysia_californica__21739
YP_025738_1_cytochrome_c_oxidase_subunit_II_mitochondrion____Haplopelma_schmidti
YP_007474870_1_cytochrome_c_oxidase_subunit_II_mitochondrion____Phyxioschema_suthepium
AJI44479_1_cytochrome_c_oxidase_subunit_II_partial_mitochondrion____Girardia_sp__ER2015
YP_004936156_1_cytochrome_c_oxidase_subunit_II_mitochondrion____Dugesia_japonica
BAL41009_1_cytochrome_c_oxidase_subunit_II_mitochondrion____Dugesia_ryukyuensis
YP_004581434_3_cytochrome_c_oxidase_subunit_II_mitochondrion____Rhabdopleura_compacta
ALQ28775_1_cytochrome_c_oxidase_subunit_2_mitochondrion____Euglena_gracilis_var__bacillaris
YP_009157858_1_cytochrome_c_oxidase_subunit_II_mitochondrion____Haplothrips_aculeatus
ADP24246_1_cytochrome_c_oxidase_subunit_II_mitochondrion____Gyrodactylus_sclerotystaci
AHH31501_1_cytochrome_c_oxidase_subunit_II_mitochondrion____Gyrodactylus_sp__MBS2014
YP_009054579_1_cytochrome_c_oxidase_subunit_II_mitochondrion____Paragyrodactylus_variegatus
AIQ80155_1_cytochrome_c_oxidase_subunit_2_mitochondrion____Lernaea_cyprinacea
AZL93154_1_cytochrome_c_oxidase_subunit_2_mitochondrion____Cleptes_metallicorpus
AII01660_1_cytochrome_c_oxidase_subunit_2_partial_mitochondrion____Macrolycus_ochraceus
AYV97232_1_cytochrome_c_oxidase_subunit_2_mitochondrion____Tremex_columba
Drosophila_melanogaster__17787
AAK77594_1_cytochrome_c_oxidase_subunit_2_mitochondrion____Tetrahymena_thermophila
AAF15334_1_cytochrome_oxidase_subunit_2_partial____Vigna_radiata
0.5

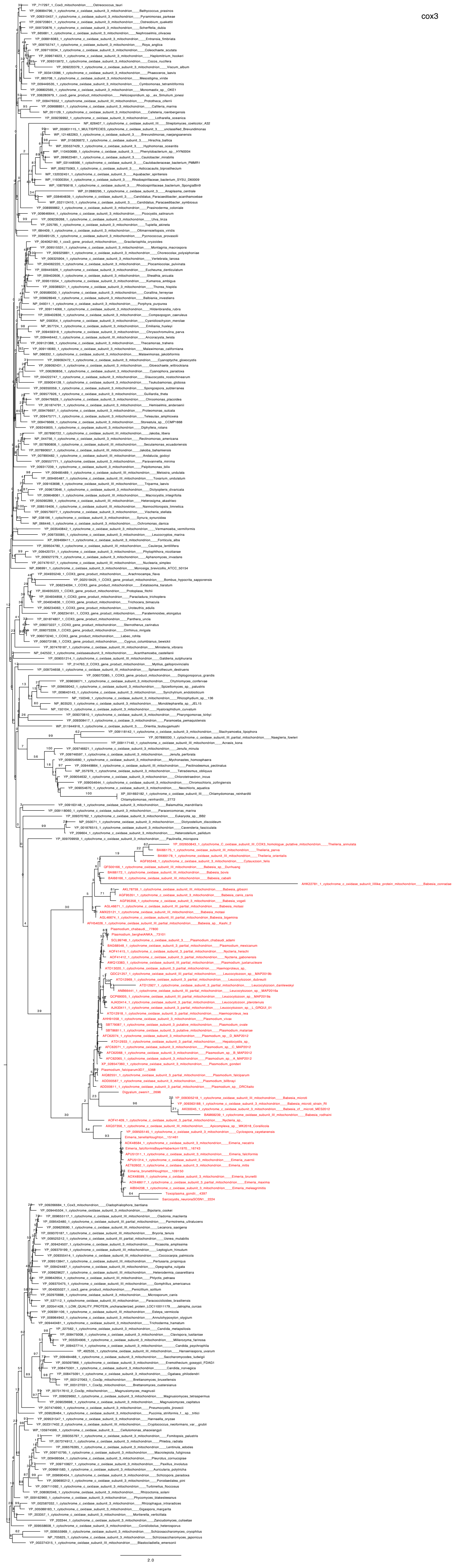
cox3
2.0

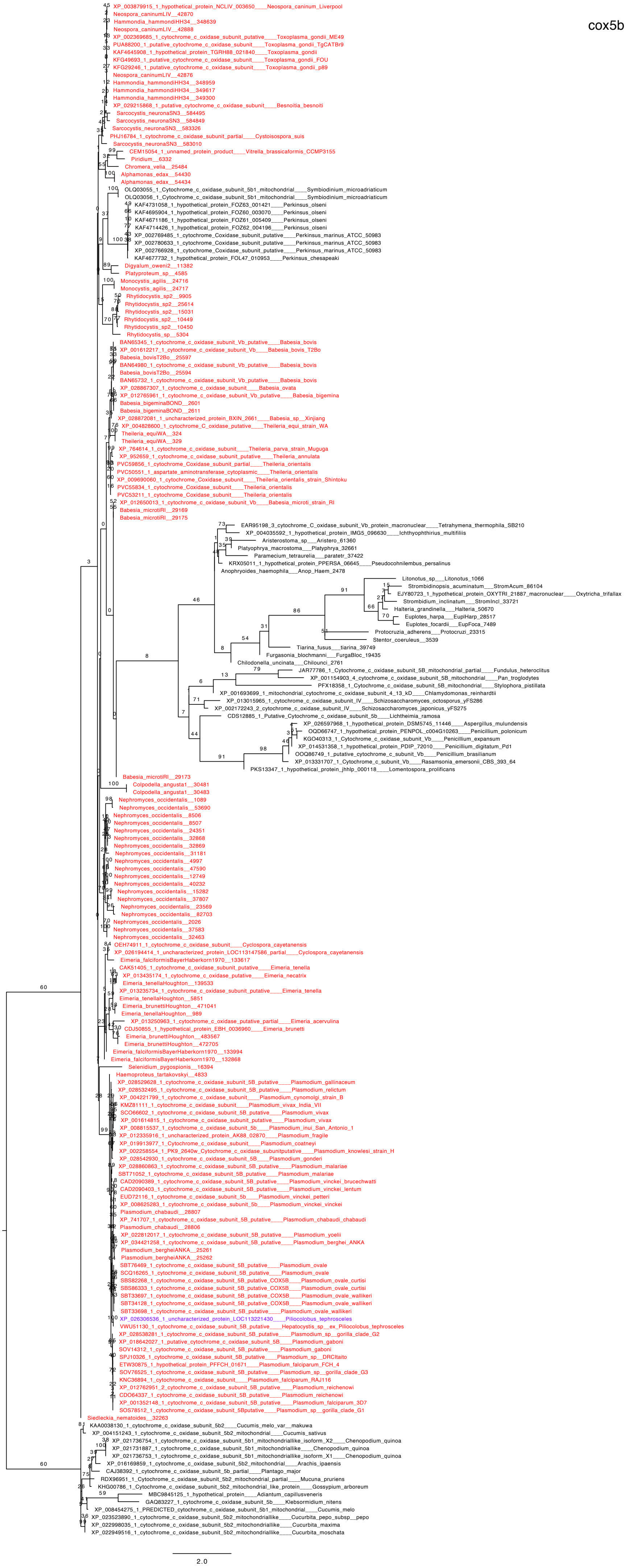
cox5b
XP_003879915_1_hypothetical_protein_NCLIV_003650____Neospora_caninum_Liverpool
Neospora_caninumLIV__42870
Hammondia_hammondiHH34__348639
Neospora_caninumLIV__42888
XP_002369685_1_cytochrome_c_oxidase_subunit_putative____Toxoplasma_gondii_ME49
PUA88200_1_putative_cytochrome_c_oxidase_subunit____Toxoplasma_gondii_TgCATBr9
KAF4645908_1_hypothetical_protein_TGRH88_021840____Toxoplasma_gondii
KFG49693_1_putative_cytochrome_c_oxidase_subunit____Toxoplasma_gondii_FOU
KFG29246_1_putative_cytochrome_c_oxidase_subunit____Toxoplasma_gondii_p89
Neospora_caninumLIV__42876
Hammondia_hammondiHH34__348959
Hammondia_hammondiHH34__349617
Hammondia_hammondiHH34__349300
XP_029215868_1_putative_cytochrome_c_oxidase_subunit____Besnoitia_besnoiti
Sarcocystis_neuronaSN3__584495
Sarcocystis_neuronaSN3__584849
Sarcocystis_neuronaSN3__583326
PHJ16784_1_cytochrome_c_oxidase_subunit_partial____Cystoisospora_suis
Sarcocystis_neuronaSN3__583010
CEM15054_1_unnamed_protein_product____Vitrella_brassicaformis_CCMP3155
Piridium__6332
Chromera_velia__25484
Alphamonas_edax__54430
Alphamonas_edax__54434
OLQ03055_1_Cytochrome_c_oxidase_subunit_5b1_mitochondrial____Symbiodinium_microadriaticum
OLQ03056_1_Cytochrome_c_oxidase_subunit_5b1_mitochondrial____Symbiodinium_microadriaticum
KAF4731058_1_hypothetical_protein_FOZ63_001421____Perkinsus_olseni
KAF4695904_1_hypothetical_protein_FOZ60_003070____Perkinsus_olseni
KAF4671186_1_hypothetical_protein_FOZ61_005409____Perkinsus_olseni
KAF4714426_1_hypothetical_protein_FOZ62_004196____Perkinsus_olseni
XP_002769485_1_cytochrome_Coxidase_subunit_putative____Perkinsus_marinus_ATCC_50983
XP_002780633_1_cytochrome_Coxidase_subunit_putative____Perkinsus_marinus_ATCC_50983
XP_002766928_1_cytochrome_Coxidase_subunit_putative____Perkinsus_marinus_ATCC_50983
KAF4677732_1_hypothetical_protein_FOL47_010953____Perkinsus_chesapeaki
Digyalum_oweni2__11382
Platyproteum_sp__4585
Monocystis_agilis__24716
Monocystis_agilis__24717
Rhytidocystis_sp2__9905
Rhytidocystis_sp2__25614
Rhytidocystis_sp2__15031
Rhytidocystis_sp2__10449
Rhytidocystis_sp2__10450
Rhytidocystis_sp__5304
BAN65345_1_cytochrome_c_oxidase_subunit_Vb_putative____Babesia_bovis
XP_001612217_1_cytochrome_c_oxidase_subunit_Vb____Babesia_bovis_T2Bo
Babesia_bovisT2Bo__25597
BAN64980_1_cytochrome_c_oxidase_subunit_Vb_putative____Babesia_bovis
Babesia_bovisT2Bo__25594
BAN65732_1_cytochrome_c_oxidase_subunit_Vb_putative____Babesia_bovis
XP_028867307_1_cytochrome_c_oxidase_subunit____Babesia_ovata
XP_012765961_1_cytochrome_c_oxidase_subunit_Vb_putative____Babesia_bigemina
Babesia_bigeminaBOND__2601
Babesia_bigeminaBOND__2611
XP_028872081_1_uncharacterized_protein_BXIN_2661____Babesia_sp__Xinjiang
XP_004828600_1_cytochrome_C_oxidase_putative____Theileria_equi_strain_WA
Theileria_equiWA__324
Theileria_equiWA__329
XP_764614_1_cytochrome_c_oxidase_subunit____Theileria_parva_strain_Muguga
XP_952659_1_cytochrome_c_oxidase_subunit_putative____Theileria_annulata
PVC59856_1_cytochrome_Coxidase_subunit_partial____Theileria_orientalis
PVC50551_1_aspartate_aminotransferase_cytoplasmic____Theileria_orientalis
XP_009690060_1_cytochrome_Coxidase_subunit____Theileria_orientalis_strain_Shintoku
PVC55834_1_cytochrome_Coxidase_subunit____Theileria_orientalis
PVC53211_1_cytochrome_Coxidase_subunit____Theileria_orientalis
XP_012650013_1_cytochrome_c_oxidase_subunit_Vb____Babesia_microti_strain_RI
Babesia_microtiRI__29169
Babesia_microtiRI__29175
EAR95198_3_cytochrome_C_oxidase_subunit_Vb_protein_macronuclear____Tetrahymena_thermophila_SB210
XP_004035592_1_hypothetical_protein_IMG5_096630____Ichthyophthirius_multifiliis
Aristerostoma_sp___Aristero_61360
Platyophrya_macrostoma___Platyphrya_32661
Paramecium_tetraurelia___paratetr_37422
KRX05011_1_hypothetical_protein_PPERSA_06645____Pseudocohnilembus_persalinus
Anophryoides_haemophila___Anop_Haem_2478
Litonotus_sp___Litonotus_1066
Strombidinopsis_acuminatum___StromAcum_86104
EJY80723_1_hypothetical_protein_OXYTRI_21887_macronuclear____Oxytricha_trifallax
Strombidium_inclinatum___StromIncl_33721
Halteria_grandinella___Halteria_50670
Euplotes_harpa___EuplHarp_28517
Euplotes_focardii___EupFoca_7489
Protocruzia_adherens___Protocruzi_23315
Stentor_coeruleus__3539
Tiarina_fusus___tiarina_39749
Furgasonia_blochmanni___FurgaBloc_19435
Chilodonella_uncinata___Chilounci_2761
JAR77786_1_Cytochrome_c_oxidase_subunit_5B_mitochondrial_partial____Fundulus_heteroclitus
XP_001154903_4_cytochrome_c_oxidase_subunit_5B_mitochondrial____Pan_troglodytes
PFX18358_1_Cytochrome_c_oxidase_subunit_5B_mitochondrial____Stylophora_pistillata
XP_001693699_1_mitochondrial_cytochrome_c_oxidase_subunit_4_13_kD____Chlamydomonas_reinhardtii
XP_013015965_1_cytochrome_c_oxidase_subunit_IV____Schizosaccharomyces_octosporus_yFS286
XP_002172243_2_cytochrome_c_oxidase_subunit_IV____Schizosaccharomyces_japonicus_yFS275
CDS12885_1_Putative_Cytochrome_c_oxidase_subunit_5b____Lichtheimia_ramosa
XP_026597968_1_hypothetical_protein_DSM5745_11446____Aspergillus_mulundensis
OQD66747_1_hypothetical_protein_PENPOL_c004G10263____Penicillium_polonicum
KGO40313_1_Cytochrome_c_oxidase_subunit_Vb____Penicillium_expansum
XP_014531358_1_hypothetical_protein_PDIP_72010____Penicillium_digitatum_Pd1
OOQ86749_1_putative_cytochrome_c_subunit_Vb____Penicillium_brasilianum
XP_013331707_1_Cytochrome_c_oxidase_subunit_Vb____Rasamsonia_emersonii_CBS_393_64
PKS13347_1_hypothetical_protein_jhhlp_000118____Lomentospora_prolificans
Babesia_microtiRI__29173
Colpodella_angusta1__30481
Colpodella_angusta1__30483
Nephromyces_occidentalis__1089
Nephromyces_occidentalis__53690
Nephromyces_occidentalis__8506
Nephromyces_occidentalis__8507
Nephromyces_occidentalis__24351
Nephromyces_occidentalis__32868
Nephromyces_occidentalis__32869
Nephromyces_occidentalis__31181
Nephromyces_occidentalis__4997
Nephromyces_occidentalis__47590
Nephromyces_occidentalis__12749
Nephromyces_occidentalis__40232
Nephromyces_occidentalis__15282
Nephromyces_occidentalis__37807
Nephromyces_occidentalis__23569
Nephromyces_occidentalis__82703
Nephromyces_occidentalis__2026
Nephromyces_occidentalis__37583
Nephromyces_occidentalis__32463
OEH74911_1_cytochrome_c_oxidase_subunit____Cyclospora_cayetanensis
XP_026194414_1_uncharacterized_protein_LOC113147586_partial____Cyclospora_cayetanensis
Eimeria_falciformisBayerHaberkorn1970__133617
CAK51405_1_cytochrome_c_oxidase_subunit_putative____Eimeria_tenella
XP_013435174_1_cytochrome_c_oxidase_putative____Eimeria_necatrix
Eimeria_tenellaHoughton__139533
XP_013235734_1_cytochrome_c_oxidase_subunit_putative____Eimeria_tenella
Eimeria_tenellaHoughton__5851
Eimeria_brunettiHoughton__471041
Eimeria_tenellaHoughton__989
XP_013250963_1_cytochrome_c_oxidase_putative_partial____Eimeria_acervulina
CDJ50855_1_hypothetical_protein_EBH_0036960____Eimeria_brunetti
Eimeria_brunettiHoughton__483567
Eimeria_brunettiHoughton__472705
Eimeria_falciformisBayerHaberkorn1970__133994
Eimeria_falciformisBayerHaberkorn1970__132868
Selenidium_pygospionis__16394
Haemoproteus_tartakovskyi__4833
XP_028529628_1_cytochrome_c_oxidase_subunit_5B_putative____Plasmodium_gallinaceum
XP_028532495_1_cytochrome_c_oxidase_subunit_5B_putative____Plasmodium_relictum
XP_004221799_1_cytochrome_c_oxidase_subunit____Plasmodium_cynomolgi_strain_B
KMZ81111_1_cytochrome_c_oxidase_subunit____Plasmodium_vivax_India_VII
SCO66602_1_cytochrome_c_oxidase_subunit_5B_putative____Plasmodium_vivax
XP_001614815_1_cytochrome_c_oxidase_subunit_putative____Plasmodium_vivax
XP_008815537_1_cytochrome_c_oxidase_subunit_5b____Plasmodium_inui_San_Antonio_1
XP_012335916_1_uncharacterized_protein_AK88_02870____Plasmodium_fragile
XP_019913977_1_Cytochrome_c_oxidase_subunit____Plasmodium_coatneyi
XP_002258554_1_PK9_2640w_Cytochrome_c_oxidase_subunitputative____Plasmodium_knowlesi_strain_H
XP_028542930_1_cytochrome_c_oxidase_subunit_5B____Plasmodium_gonderi
XP_028860863_1_cytochrome_c_oxidase_subunit_5B_putative____Plasmodium_malariae
SBT71052_1_cytochrome_c_oxidase_subunit_5B_putative____Plasmodium_malariae
CAD2090389_1_cytochrome_c_oxidase_subunit_5B_putative____Plasmodium_vinckei_brucechwatti
CAD2090403_1_cytochrome_c_oxidase_subunit_5B_putative____Plasmodium_vinckei_lentum
EUD72116_1_cytochrome_c_oxidase_subunit_5b____Plasmodium_vinckei_petteri
XP_008625283_1_cytochrome_c_oxidase_subunit_5b____Plasmodium_vinckei_vinckei
Plasmodium_chabaudi__28807
XP_741707_1_cytochrome_c_oxidase_subunit_5B_putative____Plasmodium_chabaudi_chabaudi
Plasmodium_chabaudi__28806
XP_022812017_1_cytochrome_c_oxidase_subunit_5B_putative____Plasmodium_yoelii
XP_034421258_1_cytochrome_c_oxidase_subunit_5B_putative____Plasmodium_berghei_ANKA
Plasmodium_bergheiANKA__25261
Plasmodium_bergheiANKA__25262
SBT76469_1_cytochrome_c_oxidase_subunit_5B_putative____Plasmodium_ovale
SCQ16265_1_cytochrome_c_oxidase_subunit_5B_putative____Plasmodium_ovale
SBS82268_1_cytochrome_c_oxidase_subunit_5B_putative_COX5B____Plasmodium_ovale_curtisi
SBS86333_1_cytochrome_c_oxidase_subunit_5B_putative_COX5B____Plasmodium_ovale_curtisi
SBT33697_1_cytochrome_c_oxidase_subunit_5B_putative_COX5B____Plasmodium_ovale_wallikeri
SBT34128_1_cytochrome_c_oxidase_subunit_5B_putative_COX5B____Plasmodium_ovale_wallikeri
SBT33698_1_cytochrome_c_oxidase_subunit_5B_putative____Plasmodium_ovale_wallikeri
XP_026306536_1_uncharacterized_protein_LOC113221430____Piliocolobus_tephrosceles
VWU51130_1_cytochrome_c_oxidase_subunit_5B_putative____Hepatocystis_sp__ex_Piliocolobus_tephrosceles
XP_028538281_1_cytochrome_c_oxidase_subunit_5B_putative____Plasmodium_sp__gorilla_clade_G2
XP_018642027_1_putative_cytochrome_c_oxidase_subunit_5B____Plasmodium_gaboni
SOV14312_1_cytochrome_c_oxidase_subunit_5B_putative____Plasmodium_gaboni
SPJ10326_1_cytochrome_c_oxidase_subunit_5B_putative____Plasmodium_sp__DRC/taito
ETW30875_1_hypothetical_protein_PFFCH_01671____Plasmodium_falciparum_FCH_4
SOV76525_1_cytochrome_c_oxidase_subunit_5B_putative____Plasmodium_sp__gorilla_clade_G3
KNC36894_1_cytochrome_c_oxidase_subunit____Plasmodium_falciparum_RAJ116
XP_012762951_2_cytochrome_c_oxidase_subunit_5B_putative____Plasmodium_reichenowi
CDO64337_1_cytochrome_c_oxidase_subunit_5B_putative____Plasmodium_reichenowi
XP_001352148_1_cytochrome_c_oxidase_subunit_5B_putative____Plasmodium_falciparum_3D7
SOS78512_1_cytochrome_c_oxidase_subunit_5Bputative____Plasmodium_sp__gorilla_clade_G1
Siedleckia_nematoides__32263
KAA0038130_1_cytochrome_c_oxidase_subunit_5b2____Cucumis_melo_var__makuwa
XP_004151243_1_cytochrome_c_oxidase_subunit_5b2_mitochondrial____Cucumis_sativus
XP_021736754_1_cytochrome_c_oxidase_subunit_5b1_mitochondriallike_isoform_X2____Chenopodium_quinoa
XP_021731887_1_cytochrome_c_oxidase_subunit_5b1_mitochondriallike____Chenopodium_quinoa
XP_021736753_1_cytochrome_c_oxidase_subunit_5b1_mitochondriallike_isoform_X1____Chenopodium_quinoa
XP_016169859_1_cytochrome_c_oxidase_subunit_5b2_mitochondrial____Arachis_ipaensis
CAJ38392_1_cytochrome_c_oxidase_subunit_5b_partial____Plantago_major
RDX96951_1_Cytochrome_c_oxidase_subunit_5b2_mitochondrial_partial____Mucuna_pruriens
KHG00786_1_Cytochrome_c_oxidase_subunit_5b2_mitochondrial_like_protein____Gossypium_arboreum
MBC9845125_1_hypothetical_protein____Adiantum_capillusveneris
GAQ83227_1_cytochrome_c_oxidase_subunit_5b____Klebsormidium_nitens
XP_008454275_1_PREDICTED_cytochrome_c_oxidase_subunit_5b1_mitochondrial____Cucumis_melo
XP_023523890_1_cytochrome_c_oxidase_subunit_5b2_mitochondriallike____Cucurbita_pepo_subsp__pepo
XP_022998035_1_cytochrome_c_oxidase_subunit_5b2_mitochondriallike____Cucurbita_maxima
XP_022949516_1_cytochrome_c_oxidase_subunit_5b2_mitochondriallike____Cucurbita_moschata
2.0

cyt_c1

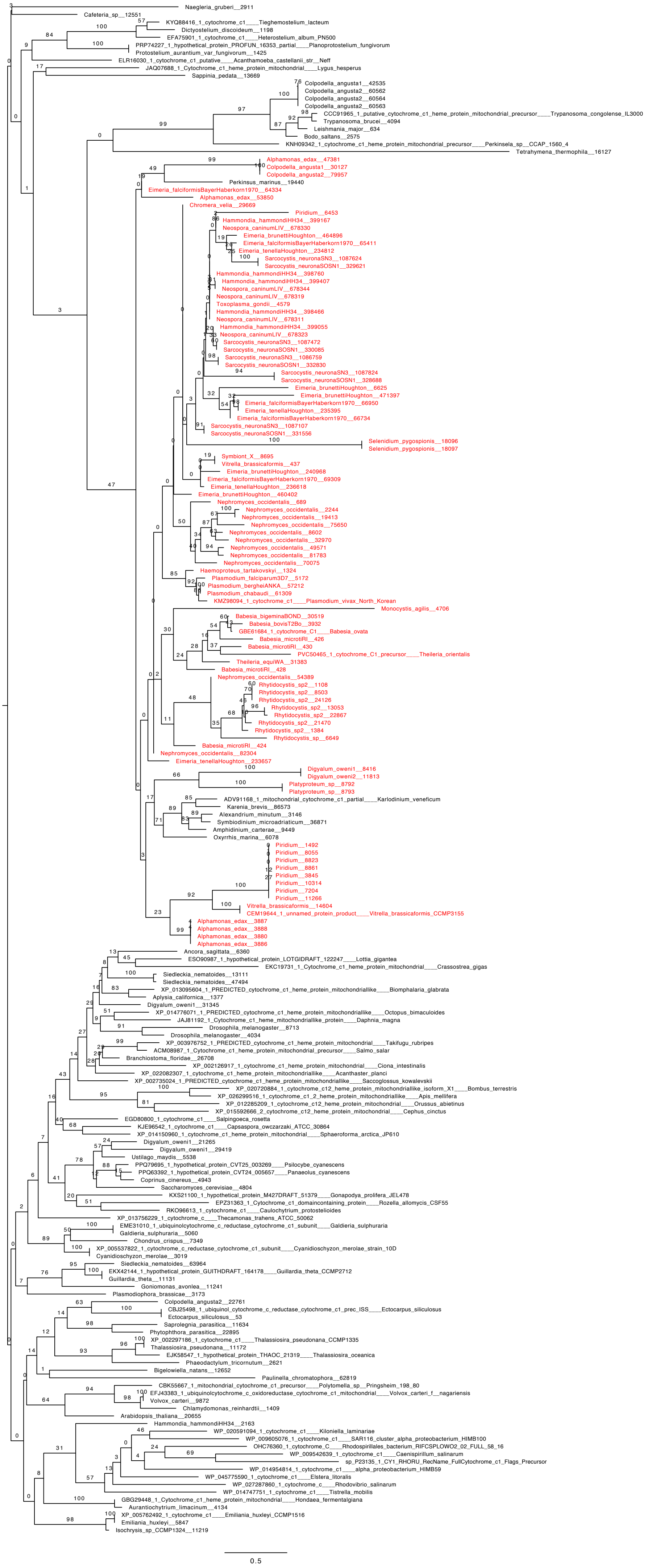

Naegleria_gruberi__2911
Cafeteria_sp__12551
KYQ88416_1_cytochrome_c1____Tieghemostelium_lacteum
Dictyostelium_discoideum__1198
EFA75901_1_cytochrome_c1____Heterostelium_album_PN500
PRP74227_1_hypothetical_protein_PROFUN_16353_partial____Planoprotostelium_fungivorum
Protostelium_aurantium_var_fungivorum__1425
ELR16030_1_cytochrome_c1_putative____Acanthamoeba_castellanii_str__Neff
JAQ07688_1_Cytochrome_c1_heme_protein_mitochondrial____Lygus_hesperus
Sappinia_pedata__13669
Colpodella_angusta1__42535
Colpodella_angusta2__60562
Colpodella_angusta2__60564
Colpodella_angusta2__60563
CCC91965_1_putative_cytochrome_c1_heme_protein_mitochondrial_precursor____Trypanosoma_congolense_IL3000
Trypanosoma_brucei__4094
Leishmania_major__634
Bodo_saltans__2575
KNH09342_1_cytochrome_c1_heme_protein_mitochondrial_precursor____Perkinsela_sp__CCAP_1560_4
Tetrahymena_thermophila__16127
Alphamonas_edax__47381
Colpodella_angusta1__30127
Colpodella_angusta2__79957
Perkinsus_marinus__19440
Eimeria_falciformisBayerHaberkorn1970__64334
Alphamonas_edax__53850
Chromera_velia__29669
Piridium__6453
Hammondia_hammondiHH34__399167
Neospora_caninumLIV__678330
Eimeria_brunettiHoughton__464896
Eimeria_falciformisBayerHaberkorn1970__65411
Eimeria_tenellaHoughton__234812
Sarcocystis_neuronaSN3__1087624
Sarcocystis_neuronaSOSN1__329621
Hammondia_hammondiHH34__398760
Hammondia_hammondiHH34__399407
Neospora_caninumLIV__678344
Neospora_caninumLIV__678319
Toxoplasma_gondii__4579
Hammondia_hammondiHH34__398466
Neospora_caninumLIV__678311
Hammondia_hammondiHH34__399055
Neospora_caninumLIV__678323
Sarcocystis_neuronaSN3__1087472
Sarcocystis_neuronaSOSN1__330085
Sarcocystis_neuronaSN3__1086759
Sarcocystis_neuronaSOSN1__332830
Sarcocystis_neuronaSN3__1087824
Sarcocystis_neuronaSOSN1__328688
Eimeria_brunettiHoughton__6625
Eimeria_brunettiHoughton__471397
Eimeria_falciformisBayerHaberkorn1970__66950
Eimeria_tenellaHoughton__235395
Eimeria_falciformisBayerHaberkorn1970__66734
Sarcocystis_neuronaSN3__1087107
Sarcocystis_neuronaSOSN1__331556
Selenidium_pygospionis__18096
Selenidium_pygospionis__18097
Symbiont_X__8695
Vitrella_brassicaformis__437
Eimeria_brunettiHoughton__240968
Eimeria_falciformisBayerHaberkorn1970__69309
Eimeria_tenellaHoughton__236618
Eimeria_brunettiHoughton__460402
Nephromyces_occidentalis__689
Nephromyces_occidentalis__2244
Nephromyces_occidentalis__19413
Nephromyces_occidentalis__75650
Nephromyces_occidentalis__8602
Nephromyces_occidentalis__32970
Nephromyces_occidentalis__49571
Nephromyces_occidentalis__81783
Nephromyces_occidentalis__70075
Haemoproteus_tartakovskyi__1324
Plasmodium_falciparum3D7__5172
Plasmodium_bergheiANKA__57212
Plasmodium_chabaudi__61309
KMZ98094_1_cytochrome_c1____Plasmodium_vivax_North_Korean
Monocystis_agilis__4706
Babesia_bigeminaBOND__30519
Babesia_bovisT2Bo__3932
GBE61684_1_cytochrome_C1____Babesia_ovata
Babesia_microtiRI__426
Babesia_microtiRI__430
PVC50465_1_cytochrome_C1_precursor____Theileria_orientalis
Theileria_equiWA__31383
Babesia_microtiRI__428
Nephromyces_occidentalis__54389
Rhytidocystis_sp2__1108
Rhytidocystis_sp2__8503
Rhytidocystis_sp2__24126
Rhytidocystis_sp2__13053
Rhytidocystis_sp2__22867
Rhytidocystis_sp2__21470
Rhytidocystis_sp2__1384
Rhytidocystis_sp__6649
Babesia_microtiRI__424
Nephromyces_occidentalis__82304
Eimeria_tenellaHoughton__233657
Digyalum_oweni1__8416
Digyalum_oweni2__11813
Platyproteum_sp__8792
Platyproteum_sp__8793
ADV91168_1_mitochondrial_cytochrome_c1_partial____Karlodinium_veneficum
Karenia_brevis__86573
Alexandrium_minutum__3146
Symbiodinium_microadriaticum__36871
Amphidinium_carterae__9449
Oxyrrhis_marina__6078
Piridium__1492
Piridium__8055
Piridium__8823
Piridium__8861
Piridium__3845
Piridium__10314
Piridium__7204
Piridium__11266
Vitrella_brassicaformis__14604
CEM19644_1_unnamed_protein_product____Vitrella_brassicaformis_CCMP3155
Alphamonas_edax__3887
Alphamonas_edax__3888
Alphamonas_edax__3880
Alphamonas_edax__3886
Ancora_sagittata__6360
ESO90987_1_hypothetical_protein_LOTGIDRAFT_122247____Lottia_gigantea
EKC19731_1_Cytochrome_c1_heme_protein_mitochondrial____Crassostrea_gigas
Siedleckia_nematoides__13111
Siedleckia_nematoides__47494
XP_013095604_1_PREDICTED_cytochrome_c1_heme_protein_mitochondriallike____Biomphalaria_glabrata
Aplysia_californica__1377
Digyalum_oweni1__31345
XP_014776071_1_PREDICTED_cytochrome_c1_heme_protein_mitochondriallike____Octopus_bimaculoides
JAJ81192_1_Cytochrome_c1_heme_mitochondriallike_protein____Daphnia_magna
Drosophila_melanogaster__8713
Drosophila_melanogaster__4034
XP_003976752_1_PREDICTED_cytochrome_c1_heme_protein_mitochondrial____Takifugu_rubripes
ACM08987_1_Cytochrome_c1_heme_protein_mitochondrial_precursor____Salmo_salar
Branchiostoma_floridae__26708
XP_002126917_1_cytochrome_c1_heme_protein_mitochondrial____Ciona_intestinalis
XP_022082307_1_cytochrome_c1_heme_protein_mitochondriallike____Acanthaster_planci
XP_002735024_1_PREDICTED_cytochrome_c1_heme_protein_mitochondriallike____Saccoglossus_kowalevskii
XP_020720884_1_cytochrome_c12_heme_protein_mitochondriallike_isoform_X1____Bombus_terrestris
XP_026299516_1_cytochrome_c1_2_heme_protein_mitochondriallike____Apis_mellifera
XP_012285209_1_cytochrome_c12_heme_protein_mitochondrial____Orussus_abietinus
XP_015592666_2_cytochrome_c12_heme_protein_mitochondrial____Cephus_cinctus
EGD80800_1_cytochrome_c1____Salpingoeca_rosetta
KJE96542_1_cytochrome_c1____Capsaspora_owczarzaki_ATCC_30864
XP_014150960_1_cytochrome_c1_heme_protein_mitochondrial____Sphaeroforma_arctica_JP610
Digyalum_oweni1__21265
Digyalum_oweni1__29419
Ustilago_maydis__5538
PPQ79695_1_hypothetical_protein_CVT25_003269____Psilocybe_cyanescens
PPQ63392_1_hypothetical_protein_CVT24_005657____Panaeolus_cyanescens
Coprinus_cinereus__4943
Saccharomyces_cerevisiae__4804
KXS21100_1_hypothetical_protein_M427DRAFT_51379____Gonapodya_prolifera_JEL478
EPZ31363_1_Cytochrome_c1_domaincontaining_protein____Rozella_allomycis_CSF55
RKO96613_1_cytochrome_c1____Caulochytrium_protostelioides
XP_013756229_1_cytochrome_c____Thecamonas_trahens_ATCC_50062
EME31010_1_ubiquinolcytochrome_c_reductase_cytochrome_c1_subunit____Galdieria_sulphuraria
Galdieria_sulphuraria__5060
Chondrus_crispus__7349
XP_005537822_1_cytochrome_c_reductase_cytochrome_c1_subunit____Cyanidioschyzon_merolae_strain_10D
Cyanidioschyzon_merolae__3019
Siedleckia_nematoides__63964
EKX42144_1_hypothetical_protein_GUITHDRAFT_164178____Guillardia_theta_CCMP2712
Guillardia_theta__11131
Goniomonas_avonlea__11241
Plasmodiophora_brassicae__3173
Colpodella_angusta2__22761
CBJ25498_1_ubiquinol_cytochrome_c_reductase_cytochrome_c1_prec_ISS____Ectocarpus_siliculosus
Ectocarpus_siliculosus__53
Saprolegnia_parasitica__11634
Phytophthora_parasitica__22895
XP_002297186_1_cytochrome_c1____Thalassiosira_pseudonana_CCMP1335
Thalassiosira_pseudonana__11172
EJK58547_1_hypothetical_protein_THAOC_21319____Thalassiosira_oceanica
Phaeodactylum_tricornutum__2621
Bigelowiella_natans__12652
Paulinella_chromatophora__62819
CBK55667_1_mitochondrial_cytochrome_c1_precursor____Polytomella_sp__Pringsheim_198_80
EFJ43383_1_ubiquinolcytochrome_c_oxidoreductase_cytochrome_c1_mitochondrial____Volvox_carteri_f__nagariensis
Volvox_carteri__9872
Chlamydomonas_reinhardtii__1409
Arabidopsis_thaliana__20655
Hammondia_hammondiHH34__2163
WP_020591094_1_cytochrome_c1____Kiloniella_laminariae
WP_009605076_1_cytochrome_c1____SAR116_cluster_alpha_proteobacterium_HIMB100
OHC76360_1_cytochrome_C____Rhodospirillales_bacterium_RIFCSPLOWO2_02_FULL_58_16
WP_009542639_1_cytochrome_c1____Caenispirillum_salinarum
sp_P23135_1_CY1_RHORU_RecName_FullCytochrome_c1_Flags_Precursor
WP_014954814_1_cytochrome_c1____alpha_proteobacterium_HIMB59
WP_045775590_1_cytochrome_c1____Elstera_litoralis
WP_027287860_1_cytochrome_c____Rhodovibrio_salinarum
WP_014747751_1_cytochrome_c1____Tistrella_mobilis
GBG29448_1_Cytochrome_c1_heme_protein_mitochondrial____Hondaea_fermentalgiana
Aurantiochytrium_limacinum__4134
XP_005762492_1_cytochrome_c1____Emiliania_huxleyi_CCMP1516
Emiliania_huxleyi__5847
Isochrysis_sp_CCMP1324__11219
0.5

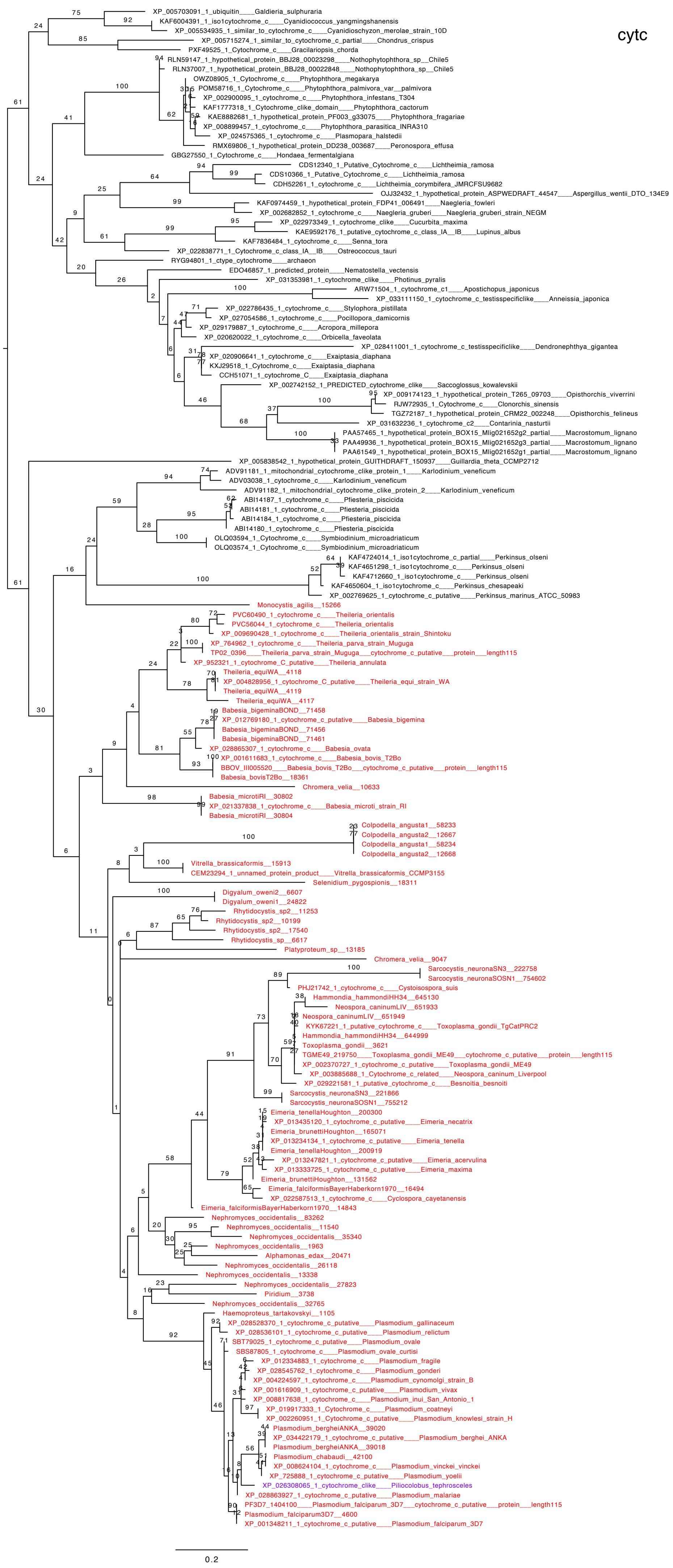

cytc
XP_005703091_1_ubiquitin____Galdieria_sulphuraria
KAF6004391_1_iso1cytochrome_c____Cyanidiococcus_yangmingshanensis
XP_005534935_1_similar_to_cytochrome_c____Cyanidioschyzon_merolae_strain_10D
XP_005715274_1_similar_to_cytochrome_c_partial____Chondrus_crispus
PXF49525_1_Cytochrome_c____Gracilariopsis_chorda
RLN59147_1_hypothetical_protein_BBJ28_00023298____Nothophytophthora_sp__Chile5
RLN37007_1_hypothetical_protein_BBJ28_00022848____Nothophytophthora_sp__Chile5
OWZ08905_1_Cytochrome_c____Phytophthora_megakarya
POM58716_1_Cytochrome_c____Phytophthora_palmivora_var__palmivora
XP_002900095_1_cytochrome_c____Phytophthora_infestans_T304
KAF1777318_1_Cytochrome_clike_domain____Phytophthora_cactorum
KAE8882681_1_hypothetical_protein_PF003_g33075____Phytophthora_fragariae
XP_008899457_1_cytochrome_c____Phytophthora_parasitica_INRA310
XP_024575365_1_cytochrome_c____Plasmopara_halstedii
RMX69806_1_hypothetical_protein_DD238_003687____Peronospora_effusa
GBG27550_1_Cytochrome_c____Hondaea_fermentalgiana
CDS12340_1_Putative_Cytochrome_c____Lichtheimia_ramosa
CDS10366_1_Putative_Cytochrome_c____Lichtheimia_ramosa
CDH52261_1_cytochrome_c____Lichtheimia_corymbifera_JMRCFSU9682
OJJ32432_1_hypothetical_protein_ASPWEDRAFT_44547____Aspergillus_wentii_DTO_134E9
KAF0974459_1_hypothetical_protein_FDP41_006491____Naegleria_fowleri
XP_002682852_1_cytochrome_c____Naegleria_gruberi____Naegleria_gruberi_strain_NEGM
XP_022973349_1_cytochrome_clike____Cucurbita_maxima
KAE9592176_1_putative_cytochrome_c_class_IA__IB____Lupinus_albus
KAF7836484_1_cytochrome_c____Senna_tora
XP_022838771_1_Cytochrome_c_class_IA__IB____Ostreococcus_tauri
RYG94801_1_ctype_cytochrome____archaeon
EDO46857_1_predicted_protein____Nematostella_vectensis
XP_031353981_1_cytochrome_clike____Photinus_pyralis
ARW71504_1_cytochrome_c1____Apostichopus_japonicus
XP_033111150_1_cytochrome_c_testisspecificlike____Anneissia_japonica
XP_022786435_1_cytochrome_c____Stylophora_pistillata
XP_027054586_1_cytochrome_c____Pocillopora_damicornis
XP_029179887_1_cytochrome_c____Acropora_millepora
XP_020620022_1_cytochrome_c____Orbicella_faveolata
XP_028411001_1_cytochrome_c_testisspecificlike____Dendronephthya_gigantea
XP_020906641_1_cytochrome_c____Exaiptasia_diaphana
KXJ29518_1_Cytochrome_c____Exaiptasia_diaphana
CCH51071_1_cytochrome_C____Exaiptasia_diaphana
XP_002742152_1_PREDICTED_cytochrome_clike____Saccoglossus_kowalevskii
XP_009174123_1_hypothetical_protein_T265_09703____Opisthorchis_viverrini
RJW72935_1_Cytochrome_c____Clonorchis_sinensis
TGZ72187_1_hypothetical_protein_CRM22_002248____Opisthorchis_felineus
XP_031632236_1_cytochrome_c2____Contarinia_nasturtii
PAA57465_1_hypothetical_protein_BOX15_Mlig021652g2_partial____Macrostomum_lignano
PAA49936_1_hypothetical_protein_BOX15_Mlig021652g3_partial____Macrostomum_lignano
PAA61549_1_hypothetical_protein_BOX15_Mlig021652g1_partial____Macrostomum_lignano
XP_005838542_1_hypothetical_protein_GUITHDRAFT_150937____Guillardia_theta_CCMP2712
ADV91181_1_mitochondrial_cytochrome_clike_protein_1____Karlodinium_veneficum
ADV03038_1_cytochrome_c____Karlodinium_veneficum
ADV91182_1_mitochondrial_cytochrome_clike_protein_2____Karlodinium_veneficum
ABI14187_1_cytochrome_c____Pfiesteria_piscicida
ABI14181_1_cytochrome_c____Pfiesteria_piscicida
ABI14184_1_cytochrome_c____Pfiesteria_piscicida
ABI14180_1_cytochrome_c____Pfiesteria_piscicida
OLQ03594_1_Cytochrome_c____Symbiodinium_microadriaticum
OLQ03574_1_Cytochrome_c____Symbiodinium_microadriaticum
KAF4724014_1_iso1cytochrome_c_partial____Perkinsus_olseni
KAF4651298_1_cytochrome_c____Perkinsus_olseni
KAF4712660_1_iso1cytochrome_c____Perkinsus_olseni
KAF4650604_1_iso1cytochrome_c____Perkinsus_chesapeaki
XP_002769625_1_cytochrome_c_putative____Perkinsus_marinus_ATCC_50983
Monocystis_agilis__15266
PVC60490_1_cytochrome_c____Theileria_orientalis
PVC56044_1_cytochrome_c____Theileria_orientalis
XP_009690428_1_cytochrome_c____Theileria_orientalis_strain_Shintoku
XP_764962_1_cytochrome_c____Theileria_parva_strain_Muguga
TP02_0396____Theileria_parva_strain_Muguga___cytochrome_c_putative___protein___length115
XP_952321_1_cytochrome_C_putative____Theileria_annulata
Theileria_equiWA__4118
XP_004828956_1_cytochrome_C_putative____Theileria_equi_strain_WA
Theileria_equiWA__4119
Theileria_equiWA__4117
Babesia_bigeminaBOND__71458
XP_012769180_1_cytochrome_c_putative____Babesia_bigemina
Babesia_bigeminaBOND__71456
Babesia_bigeminaBOND__71461
XP_028865307_1_cytochrome_c____Babesia_ovata
XP_001611683_1_cytochrome_c____Babesia_bovis_T2Bo
BBOV_III005520____Babesia_bovis_T2Bo___cytochrome_c_putative___protein___length115
Babesia_bovisT2Bo__18361
Chromera_velia__10633
Babesia_microtiRI__30802
XP_021337838_1_cytochrome_c____Babesia_microti_strain_RI
Babesia_microtiRI__30804
Colpodella_angusta1__58233
Colpodella_angusta2__12667
Colpodella_angusta1__58234
Colpodella_angusta2__12668
Vitrella_brassicaformis__15913
CEM23294_1_unnamed_protein_product____Vitrella_brassicaformis_CCMP3155
Selenidium_pygospionis__18311
Digyalum_oweni2__6607
Digyalum_oweni1__24822
Rhytidocystis_sp2__11253
Rhytidocystis_sp2__10199
Rhytidocystis_sp2__17540
Rhytidocystis_sp__6617
Platyproteum_sp__13185
Chromera_velia__9047
Sarcocystis_neuronaSN3__222758
Sarcocystis_neuronaSOSN1__754602
PHJ21742_1_cytochrome_c____Cystoisospora_suis
Hammondia_hammondiHH34__645130
Neospora_caninumLIV__651933
Neospora_caninumLIV__651949
KYK67221_1_putative_cytochrome_c____Toxoplasma_gondii_TgCatPRC2
Hammondia_hammondiHH34__644999
Toxoplasma_gondii__3621
TGME49_219750____Toxoplasma_gondii_ME49___cytochrome_c_putative___protein___length115
XP_002370727_1_cytochrome_c_putative____Toxoplasma_gondii_ME49
XP_003885688_1_Cytochrome_c_related____Neospora_caninum_Liverpool
XP_029221581_1_putative_cytochrome_c____Besnoitia_besnoiti
Sarcocystis_neuronaSN3__221866
Sarcocystis_neuronaSOSN1__755212
Eimeria_tenellaHoughton__200300
XP_013435120_1_cytochrome_c_putative____Eimeria_necatrix
Eimeria_brunettiHoughton__165071
XP_013234134_1_cytochrome_c_putative____Eimeria_tenella
Eimeria_tenellaHoughton__200919
XP_013247821_1_cytochrome_c_putative____Eimeria_acervulina
XP_013333725_1_cytochrome_c_putative____Eimeria_maxima
Eimeria_brunettiHoughton__131562
Eimeria_falciformisBayerHaberkorn1970__16494
XP_022587513_1_cytochrome_c____Cyclospora_cayetanensis
Eimeria_falciformisBayerHaberkorn1970__14843
Nephromyces_occidentalis__83262
Nephromyces_occidentalis__11540
Nephromyces_occidentalis__35340
Nephromyces_occidentalis__1963
Alphamonas_edax__20471
Nephromyces_occidentalis__26118
Nephromyces_occidentalis__13338
Nephromyces_occidentalis__27823
Piridium__3738
Nephromyces_occidentalis__32765
Haemoproteus_tartakovskyi__1105
XP_028528370_1_cytochrome_c_putative____Plasmodium_gallinaceum
XP_028536101_1_cytochrome_c_putative____Plasmodium_relictum
SBT79025_1_cytochrome_c_putative____Plasmodium_ovale
SBS87805_1_cytochrome_c____Plasmodium_ovale_curtisi
XP_012334883_1_cytochrome_c____Plasmodium_fragile
XP_028545762_1_cytochrome_c____Plasmodium_gonderi
XP_004224597_1_cytochrome_c____Plasmodium_cynomolgi_strain_B
XP_001616909_1_cytochrome_c_putative____Plasmodium_vivax
XP_008817638_1_cytochrome_c____Plasmodium_inui_San_Antonio_1
XP_019917333_1_Cytochrome_c____Plasmodium_coatneyi
XP_002260951_1_Cytochrome_c_putative____Plasmodium_knowlesi_strain_H
Plasmodium_bergheiANKA__39020
XP_034422179_1_cytochrome_c_putative____Plasmodium_berghei_ANKA
Plasmodium_bergheiANKA__39018
Plasmodium_chabaudi__42100
XP_008624104_1_cytochrome_c____Plasmodium_vinckei_vinckei
XP_725888_1_cytochrome_c_putative____Plasmodium_yoelii
XP_026308065_1_cytochrome_clike____Piliocolobus_tephrosceles
XP_028863927_1_cytochrome_c_putative____Plasmodium_malariae
PF3D7_1404100____Plasmodium_falciparum_3D7___cytochrome_c_putative___protein___length115
Plasmodium_falciparum3D7__4600
XP_001348211_1_cytochrome_c_putative____Plasmodium_falciparum_3D7
0.2

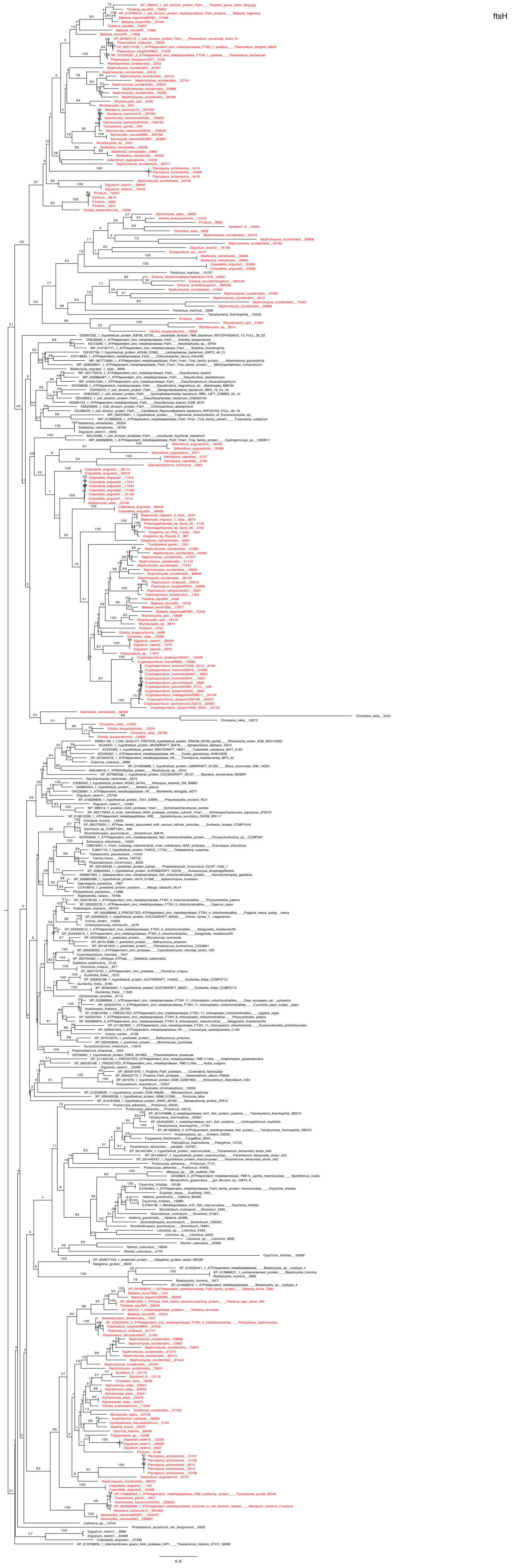
ftsH
0.6

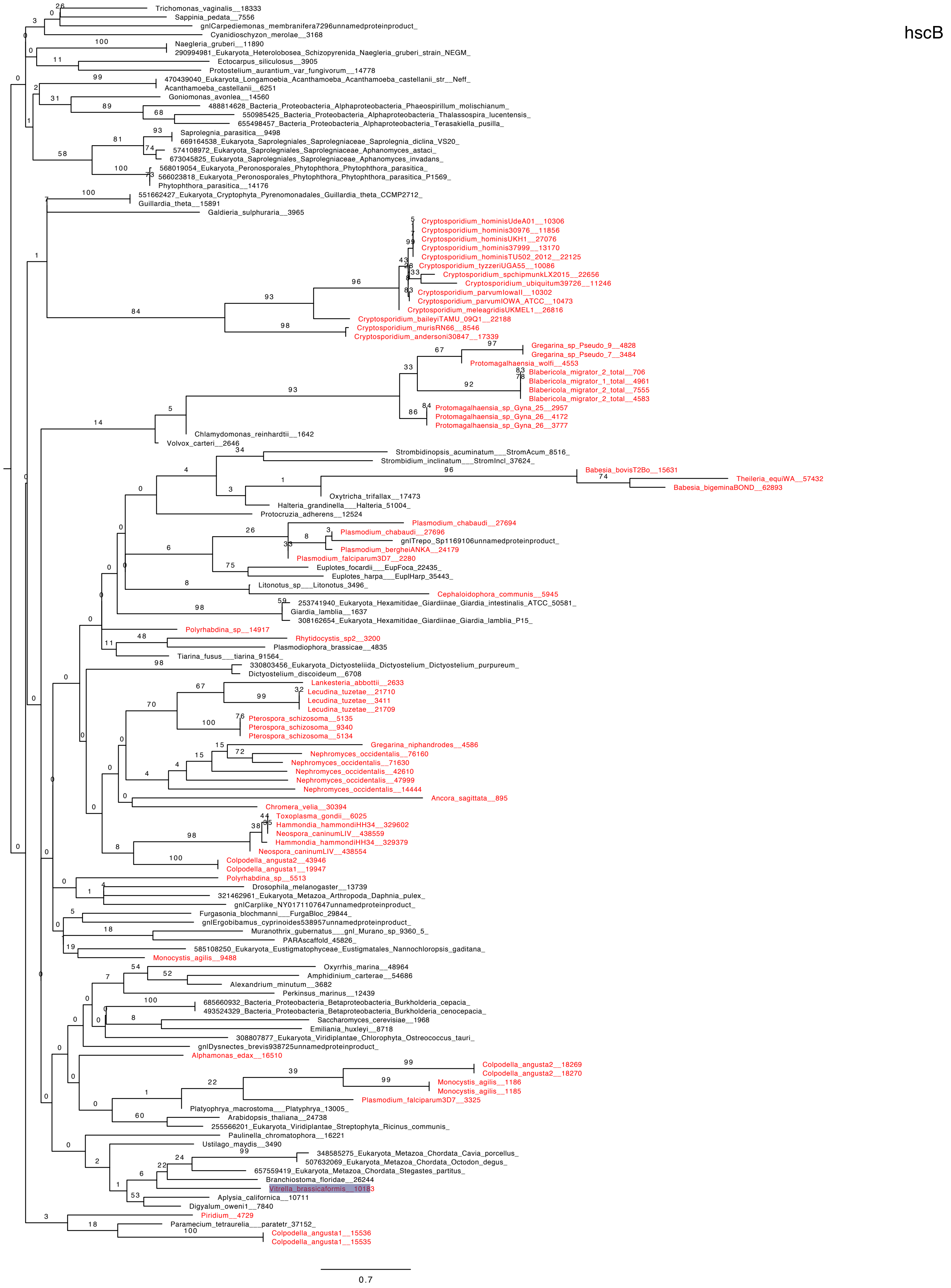
hscB
0.7

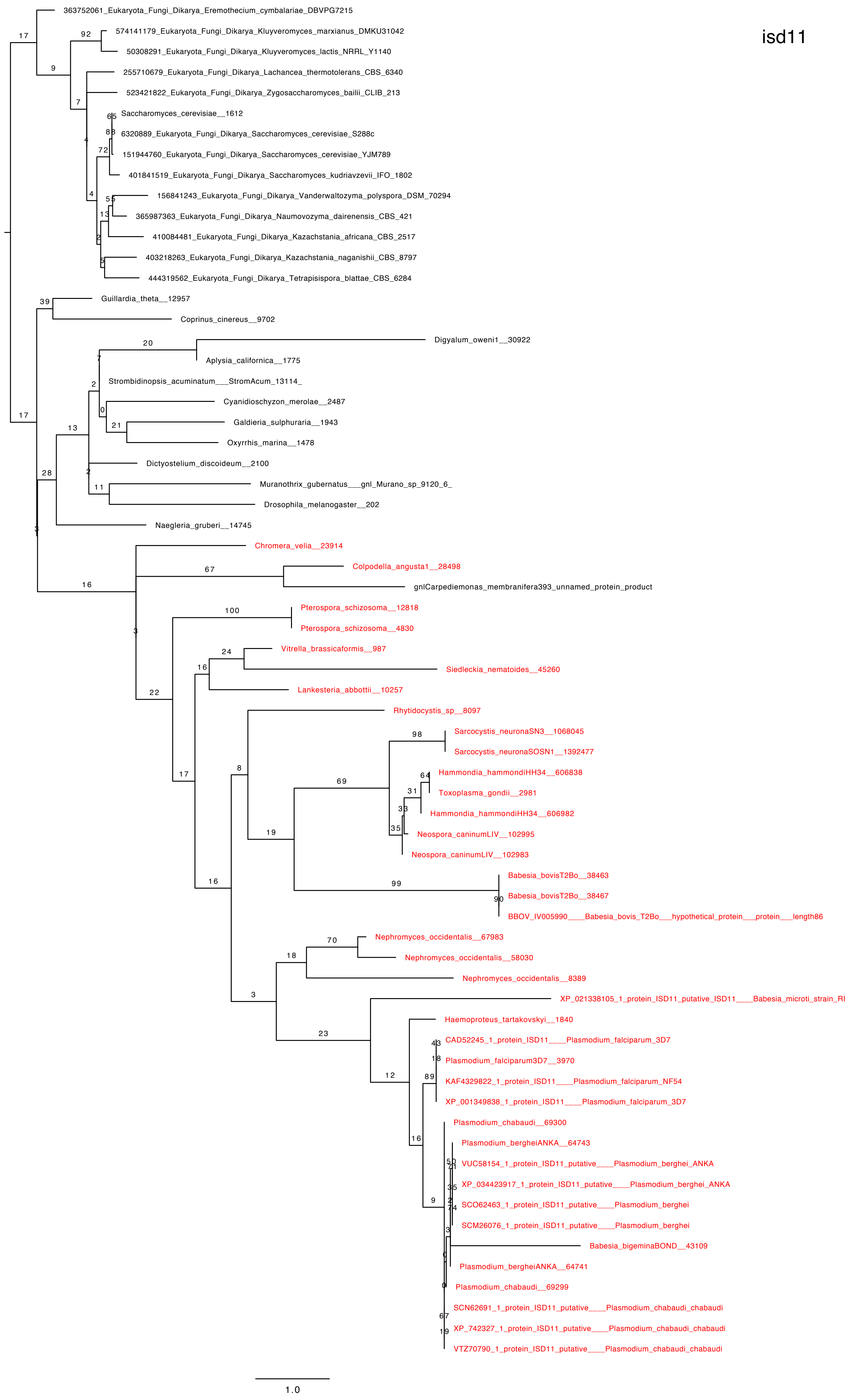
isd11
363752061_Eukaryota_Fungi_Dikarya_Eremothecium_cymbalariae_DBVPG7215
574141179_Eukaryota_Fungi_Dikarya_Kluyveromyces_marxianus_DMKU31042
50308291_Eukaryota_Fungi_Dikarya_Kluyveromyces_lactis_NRRL_Y1140
255710679_Eukaryota_Fungi_Dikarya_Lachancea_thermotolerans_CBS_6340
523421822_Eukaryota_Fungi_Dikarya_Zygosaccharomyces_bailii_CLIB_213
Saccharomyces_cerevisiae__1612
6320889_Eukaryota_Fungi_Dikarya_Saccharomyces_cerevisiae_S288c
151944760_Eukaryota_Fungi_Dikarya_Saccharomyces_cerevisiae_YJM789
401841519_Eukaryota_Fungi_Dikarya_Saccharomyces_kudriavzevii_IFO_1802
156841243_Eukaryota_Fungi_Dikarya_Vanderwaltozyma_polyspora_DSM_70294
365987363_Eukaryota_Fungi_Dikarya_Naumovozyma_dairenensis_CBS_421
410084481_Eukaryota_Fungi_Dikarya_Kazachstania_africana_CBS_2517
403218263_Eukaryota_Fungi_Dikarya_Kazachstania_naganishii_CBS_8797
444319562_Eukaryota_Fungi_Dikarya_Tetrapisispora_blattae_CBS_6284
Guillardia_theta__12957
Coprinus_cinereus__9702
Digyalum_oweni1__30922
Aplysia_californica__1775
Strombidinopsis_acuminatum___StromAcum_13114_
Cyanidioschyzon_merolae__2487
Galdieria_sulphuraria__1943
Oxyrrhis_marina__1478
Dictyostelium_discoideum__2100
Muranothrix_gubernatus___gnl_Murano_sp_9120_6_
Drosophila_melanogaster__202
Naegleria_gruberi__14745
Chromera_velia__23914
Colpodella_angusta1__28498
gnlCarpediemonas_membranifera393_unnamed_protein_product
Pterospora_schizosoma__12818
Pterospora_schizosoma__4830
Vitrella_brassicaformis__987
Siedleckia_nematoides__45260
Lankesteria_abbottii__10257
Rhytidocystis_sp__8097
Sarcocystis_neuronaSN3__1068045
Sarcocystis_neuronaSOSN1__1392477
Hammondia_hammondiHH34__606838
Toxoplasma_gondii__2981
Hammondia_hammondiHH34__606982
Neospora_caninumLIV__102995
Neospora_caninumLIV__102983
Babesia_bovisT2Bo__38463
Babesia_bovisT2Bo__38467
BBOV_IV005990____Babesia_bovis_T2Bo___hypothetical_protein___protein___length86
Nephromyces_occidentalis__67983
Nephromyces_occidentalis__58030
Nephromyces_occidentalis__8389
XP_021338105_1_protein_ISD11_putative_ISD11____Babesia_microti_strain_RI
Haemoproteus_tartakovskyi__1840
CAD52245_1_protein_ISD11____Plasmodium_falciparum_3D7
Plasmodium_falciparum3D7__3970
KAF4329822_1_protein_ISD11____Plasmodium_falciparum_NF54
XP_001349838_1_protein_ISD11____Plasmodium_falciparum_3D7
Plasmodium_chabaudi__69300
Plasmodium_bergheiANKA__64743
VUC58154_1_protein_ISD11_putative____Plasmodium_berghei_ANKA
XP_034423917_1_protein_ISD11_putative____Plasmodium_berghei_ANKA
SCO62463_1_protein_ISD11_putative____Plasmodium_berghei
SCM26076_1_protein_ISD11_putative____Plasmodium_berghei
Babesia_bigeminaBOND__43109
Plasmodium_bergheiANKA__64741
Plasmodium_chabaudi__69299
SCN62691_1_protein_ISD11_putative____Plasmodium_chabaudi_chabaudi
XP_742327_1_protein_ISD11_putative____Plasmodium_chabaudi_chabaudi
VTZ70790_1_protein_ISD11_putative____Plasmodium_chabaudi_chabaudi
1.0
